# Supplementary figures and images for: Systematically testing human HMBS missense variants to reveal mechanism and pathogenic variation
Source: bioRxiv. 2023 Feb 6:2023.02.06.527353. Preprint. [Version 1] doi: 10.1101/2023.02.06.527353 (PMC9934555; doi:10.1101/2023.02.06.527353)

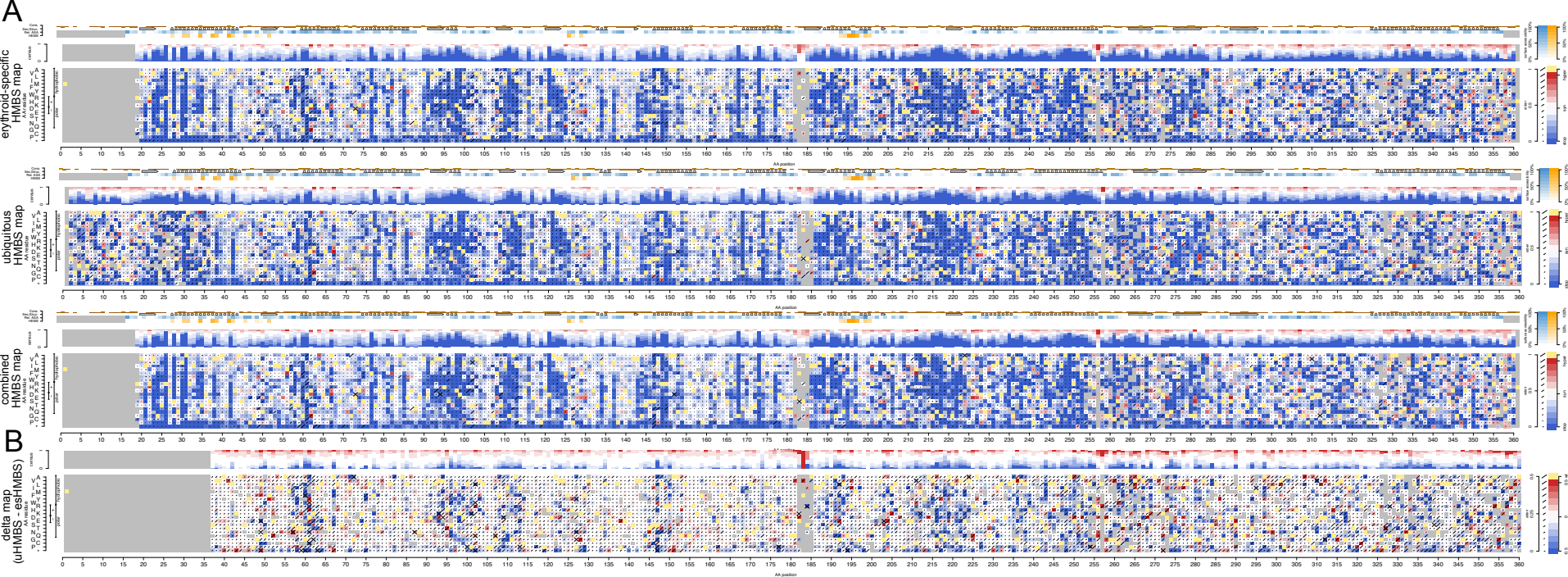

Supplement: Supplement 1 [file media-1.pdf]
