# Supplementary material for: Systematically testing human HMBS missense variants to reveal mechanism and pathogenic variation
Source: bioRxiv. 2023 Feb 6:2023.02.06.527353. Preprint. [Version 1] doi: 10.1101/2023.02.06.527353 (PMC9934555; doi:10.1101/2023.02.06.527353)
Supplement: Supplement 2 [file media-2.pdf]

Table S7: Curated reference set of disease- and non-disease-associated variants

protein consequence	nucleotide change	wt aa	pos	mut aa
p.Ala219Asp	c.0656C>A	Ala	219	Asp
p.Ala31Pro	c.0091G>C	Ala	31	Pro
p.Ala31Thr	c.0091G>A	Ala	31	Thr
p.Arg116Gln	c.0347G>A	Arg	116	Gln
p.Arg116Trp	c.0346C>T	Arg	116	Trp
p.Arg149Gln	c.0446G>A	Arg	149	Gln
p.Arg149Leu	c.0446G>T	Arg	149	Leu
p.Arg167Gln	c.0500G>A	Arg	167	Gln
p.Arg167Trp	c.0499C>T	Arg	167	Trp
p.Arg173Gln	c.0518G>A; c.0578G>A	Arg	173	Gln
p.Arg173Trp	c.0517C>T	Arg	173	Trp
p.Arg195Cys	c.0583C>T	Arg	195	Cys
p.Arg195His	c.0584G>A	Arg	195	His
p.Arg201Trp	c.0601C>T	Arg	201	Trp
p.Arg225Gln	c.0674G>A	Arg	225	Gln
p.Arg246Cys	c.0736C>T	Arg	246	Cys
p.Arg255Ser	c.0765G>C	Arg	255	Ser
p.Arg26Cys	c.0076C>T	Arg	26	Cys
p.Arg26His	c.0077G>A	Arg	26	His
p.Arg321His	c.0962G>A	Arg	321	His
p.Arg32His	c.0095G>A	Arg	32	His
p.Asp230Tyr	c.0688G>T	Asp	230	Tyr
p.Asp359Asn	c.1075G>A	Asp	359	Asn
p.Asp61Tyr	c.0181G>A	Asp	61	Tyr
p.Asp99Gly	c.0296A>G	Asp	99	Gly
p.Cys247Arg	c.0739T>C	Cys	247	Arg
p.Gln296Arg	c.0887A>G	Gln	296	Arg
p.Glu250Ala	c.0749A>C	Glu	250	Ala
p.Glu250Lys	c.0748G>A	Glu	250	Lys
p.Glu86Val	c.0257A>T	Glu	86	Val
p.Gly111Arg	c.0331G>A	Gly	111	Arg
p.Gly168Arg	c.0502G>A	Gly	168	Arg
p.Gly216Asp	c.0647G>A	Gly	216	Asp
p.Gly280Glu	c.0839G>A	Gly	280	Glu
p.Gly335Asp	c.1004G>A	Gly	335	Asp
p.Ile113Thr	c.0338T>C	Ile	113	Thr
p.Leu177Arg	c.0530T>G	Leu	177	Arg
p.Leu234Pro	c.0701T>C	Leu	234	Pro
p.Leu238Pro	c.0713T>C	Leu	238	Pro
p.Leu254Pro	c.0761T>C	Leu	254	Pro
p.Leu276Arg	c.0827T>G	Leu	276	Arg
p.Leu278Arg	c.0833T>G	Leu	278	Arg
p.Leu30Ile	c.0088C>A	Leu	30	Ile

p.Leu30Phe	c.0088C>T	Leu	30	Phe
p.Leu30Pro	c.0089T>C	Leu	30	Pro
p.Leu343Pro	c.1028T>C	Leu	343	Pro
p.Leu42Ser	c.0125T>C	Leu	42	Ser
p.Leu85Arg	c.0254T>G	Leu	85	Arg
p.Leu92Pro	c.0275T>C	Leu	92	Pro
p.Lys132Asn	c.0396G>C	Lys	132	Asn
p.Lys348Arg	c.1043A>G	Lys	348	Arg
p.Pro119Leu	c.0356C>T	Pro	119	Leu
p.Pro127Gln	c.0380C>A	Pro	127	Gln
p.Pro159Leu	c.0476C>T	Pro	159	Leu
p.Pro309Ser	c.0925C>T	Pro	309	Ser
p.Ser147Pro	c.0439T>C	Ser	147	Pro
p.Ser28Asn	c.0083G>A	Ser	28	Asn
p.Ser45Leu	c.0134C>T	Ser	45	Leu
p.Ser96Phe	c.0287C>T	Ser	96	Phe
p.Thr269Ile	c.0806C>T	Thr	269	Ile
p.Thr35Met	c.0104C>T	Thr	35	Met
p.Val124Asp	c.0371T>A	Val	124	Asp
p.Val215Glu	c.0644T>A	Val	215	Glu
p.Val267Leu	c.0799G>C	Val	267	Leu
p.Val267Met	c.0799G>A	Val	267	Met
p.Val90Gly	c.0269T>G	Val	90	Gly

clinical annotation	combined map	eHMBS map	uHMBS map	VARITY	PROVEAN
Pathogenic	0.000	0.000	0.371	0.993	-5.48
Pathogenic	0.000	0.000	0.581	0.994	-4.6
Pathogenic	0.000	0.000	0.000	0.978	-3.68
Pathogenic	0.264	NA	0.264	0.984	-3.71
Pathogenic	0.184	0.161	0.202	0.97	-7.35
Pathogenic	0.004	0.002	0.005	0.981	-3.72
Pathogenic	0.000	0.145	0.100	0.986	-6.5
Pathogenic	0.733	0.726	0.750	0.961	-3.85
Pathogenic	0.522	0.519	0.576	0.955	-7.68
Pathogenic	0.000	0.000	0.000	0.976	-3.85
Pathogenic	0.000	0.000	0.000	0.963	-7.72
Pathogenic	0.248	0.149	0.543	0.982	-7.72
Pathogenic	0.783	0.719	0.854	0.989	-4.82
Pathogenic	0.719	0.714	0.724	0.961	-7.74
Pathogenic	0.000	0.585	0.000	0.913	-3.42
Benign	0.281	0.306	0.272	0.824	-5.75
Benign	0.883	0.954	0.841	0.765	-2.98
Pathogenic	0.000	0.000	0.000	0.967	-7.03
Pathogenic	0.000	0.000	0.287	0.936	-4.39
Benign	0.863	NA	0.863	0.115	-0.82
Benign	0.595	0.595	1.005	0.862	-1.85
Pathogenic	0.512	0.459	0.581	0.458	-2.08
Benign	0.706	0.723	0.650	0.377	-1.51
Pathogenic	0.335	0.617	0.083	0.976	-8.33
Pathogenic	0.130	0.121	0.289	0.989	-6.5
Pathogenic	0.000	0.025	0.159	0.997	-8.65
Benign	1.061	1.073	1.045	0.846	-1.64
Pathogenic	0.000	0.526	0.000	0.983	-5.36
Pathogenic	0.018	0.153	0.104	0.985	-3.57
Benign	0.737	0.737	0.737	0.455	-1.47
Pathogenic	0.015	0.093	0.131	0.976	-6.26
Pathogenic	0.000	0.049	0.009	0.987	-7.72
Pathogenic	0.000	0.000	0.390	0.992	-6.1
Pathogenic	0.000	0.053	0.016	0.993	-7.14
Pathogenic	0.432	0.219	0.522	0.968	-5.69
Pathogenic	0.471	0.434	0.561	0.777	-3.83
Pathogenic	0.121	0.108	0.144	0.987	-5.77
Pathogenic	0.000	0.000	0.201	0.99	-5.15
Pathogenic	0.555	NA	0.555	0.99	-5.75
Pathogenic	0.432	0.000	0.445	0.998	-5.71
Pathogenic	0.000	0.060	0.005	0.986	-5.04
Pathogenic	0.094	0.106	0.083	0.994	-5.45
Pathogenic	0.000	0.000	0.675	0.957	-1.84

Pathogenic	0.000	0.000	0.000	0.972	-3.68
Pathogenic	0.000	0.000	0.000	0.992	-6.45
Pathogenic	0.300	0.331	0.211	0.915	-3.71
Pathogenic	0.000	0.000	0.000	0.985	-5.53
Pathogenic	0.000	0.025	0.028	0.992	-4.96
Pathogenic	0.000	0.000	0.019	0.982	-5.12
Benign	1.202	1.227	1.186	0.665	-2.71
Benign	1.317	1.364	1.230	0.355	-1.03
Pathogenic	0.021	0.097	0.128	0.973	-9.28
Benign	1.174	1.190	1.162	0.571	-1.96
Benign	0.785	0.727	0.828	0.835	-8.55
Benign	0.779	0.807	0.751	0.244	-1.44
Pathogenic	0.000	0.000	0.000	0.994	-4.64
Pathogenic	0.000	0.000	0.000	0.928	-2.73
Benign	0.630	0.621	0.647	0.083	1.89
Pathogenic	0.000	0.026	0.000	0.971	-5.56
Pathogenic	0.000	0.048	0.026	0.957	-4.28
Pathogenic	0.800	0.800	0.853	0.938	-5.53
Pathogenic	0.000	0.000	0.000	0.989	-6.33
Pathogenic	0.647	0.647	NA	0.993	-5.83
Pathogenic	0.848	0.879	0.779	0.949	-2.61
Pathogenic	1.328	1.344	1.247	0.921	-2.65
Pathogenic	0.032	0.165	0.029	0.972	-6.07

SIFT	Investigator(s) or Source
0	Sh. Wh.
0	Sh. Wh.
0	Ed.Fr.
0	Sh. Wh.
0	Ed.Fr.; JTF; Ca.Sc; Sh. Wh.; AKA
0	Ca.Sc
0	Ca.Sc
0	Sh. Wh.; Ed.Fr.
0	Sh. Wh.; AKA; Ed.Fr.
0	JTF; Sh. Wh.; Ca.Sc
0	JTF; Sh. Wh.; AKA
0	Sh. Wh.
0	Sh. Wh.
0	Sh. Wh.
0.008	Yl.Fl
0.097	ClinVar
0.016	C.R
0	E.Di.Pi.; Ca.Sc; Sh. Wh.
0.001	E.Di.Pi.; JTF; Ca.Sc; Sh. Wh.
0.341	Yl.Fl
0.003	gnomAD v2.1.1
0.266	Sh. Wh.
0.003	Clinvar
0	Sh. Wh.
0	Yl.Fl
0.001	Sh. Wh.
0.336	RJ.D
0	Ca.Sc
0	Sh. Wh.
0.223	Yl.Fl
0.001	E.Di.Pi.; JTF; Ca.Sc; Sh. Wh.
0	Sh. Wh.
0.001	Sh. Wh.; Yl.Fl
0	Sh. Wh.
0	Ca.Sc
0.001	Yl.Fl
0	Sh. Wh.
0.001	Sh. Wh.
0	JTF; Sh. Wh.
0	Sh. Wh.
0	Sh. Wh.
0	JTF
0	Sh. Wh.

0	Ca.Sc
0	AKA
0.01	Yl.Fl
0	Sh. Wh.
0	Sh. Wh.
0.003	Yl.Fl
0.269	AKA
0.399	Yl.Fl
0.001	Yl.Fl
0.186	ClinVar
0.001	gnomAD v2.1.1
0.151	RJ.D
0	Sh. Wh.
0.001	Ca.Sc
1	ClinVar
0	Ca.Sc
0.005	Sh. Wh.
0	Ca.Sc
0	Ca.Sc
0	AKA
0.005	Sh. Wh.
0.002	Ca.Sc
0	Sh. Wh.

Table S7: Curated percentages of enzymatic activity expressed as a ratio between variant anc

HGVS	wt aa	pos	mut aa	activity relative to wt (%)
p.Arg26Cys	Arg	26	Cys	5
p.Arg26His	Arg	26	His	3
p.Ser28Asn	Ser	28	Asn	4
p.Leu30Phe	Leu	30	Phe	3
p.Thr35Met	Thr	35	Met	11
p.Ser96Phe	Ser	96	Phe	3
p.Gly111Arg	Gly	111	Arg	4
p.Arg116Trp	Arg	116	Trp	4
p.Val124Asp	Val	124	Asp	4
p.Arg149Gln	Arg	149	Gln	5
p.Arg149Leu	Arg	149	Leu	5
p.Arg173Gln	Arg	173	Gln	4
p.Glu250Ala	Glu	250	Ala	4
p.Val267Met	Val	267	Met	4
p.Val215Glu	Val	215	Glu	30
p.Arg173Trp	Arg	173	Trp	0.6
p.Arg116Trp	Arg	116	Trp	0.5
p.Arg167Trp	Arg	167	Trp	4.2
p.Leu30Pro	Leu	30	Pro	0.2
p.Lys132Asn	Lys	132	Asn	95
p.Lys132Asn	Lys	132	Asn	0.66
p.Gly24Ser	Gly	24	Ser	0.07
p.Arg26Cys	Arg	26	Cys	0
p.Arg26His	Arg	26	His	0
p.Ser28Asn	Ser	28	Asn	0.01
p.Leu30Pro	Leu	30	Pro	0
p.Ala31Pro	Ala	31	Pro	0.01
p.Arg32Pro	Arg	32	Pro	1
p.Gln34Arg	Gln	34	Arg	0.01
p.Gln34Lys	Gln	34	Lys	0
p.Gln34Pro	Gln	34	Pro	0.01
p.Thr35Met	Thr	35	Met	0.04
p.Thr59Ile	Thr	59	Ile	81
p.Phe77Leu	Phe	77	Leu	0
p.Thr78Pro	Thr	78	Pro	0.03
p.Glu80Gly	Glu	80	Gly	0.02
p.Glu86Val	Glu	86	Val	0.93
p.Val93Phe	Val	93	Phe	0.01
p.Ser96Phe	Ser	96	Phe	0.01
p.Lys98Asn	Lys	98	Asn	1
p.Lys98Arg	Lys	98	Arg	0.01
p.Asp99Gly	Asp	99	Gly	0.03
p.Asp99His	Asp	99	His	0.03

p.Ala112Pro	Ala	112	Pro	0
p.Arg116Trp	Arg	116	Trp	0.01
p.Ala122Asp	Ala	122	Asp	0
p.Lys132Asn	Lys	132	Asn	0.97
p.Thr145Ile	Thr	145	Ile	0
p.Ser147Pro	Ser	147	Pro	0
p.Arg149Leu	Arg	149	Leu	0
p.Arg149Gln	Arg	149	Gln	0.01
p.Arg149Leu	Arg	149	Leu	0.05
p.Leu154Pro	Leu	154	Pro	0
p.Arg167Gln	Arg	167	Gln	0.01
p.Arg167Trp	Arg	167	Trp	0.02
p.Leu170Pro	Leu	170	Pro	1
p.Arg173Gln	Arg	173	Gln	0.01
p.Arg173Gln	Arg	173	Gln	0
p.Asp178Asn	Asp	178	Asn	0.81
p.Arg195Cys	Arg	195	Cys	0.03
p.Arg195Cys	Arg	195	Cys	0
p.Met212Val	Met	212	Val	0.02
p.Val215Met	Val	215	Met	0.19
p.Gln217His	Gln	217	His	0.05
p.Gly218Arg	Gly	218	Arg	0
p.Val224Glu	Val	224	Glu	0.01
p.Val235Glu	Val	235	Glu	0.03
p.Asp240Gly	Asp	240	Gly	0.05
p.Cys247Arg	Cys	247	Arg	0.02
p.Cys247Phe	Cys	247	Phe	0.11
p.Glu250Asp	Glu	250	Asp	0.01
p.Glu250Gln	Glu	250	Gln	0.02
p.His256Tyr	His	256	Tyr	0.05
p.leu278Pro	leu	278	Pro	1.22
p.Arg321His	Arg	321	His	0.96
p.Gly335Ser	Gly	335	Ser	0.03
p.Ala347Pro	Ala	347	Pro	51
p.Asp359Asn	Asp	359	Asn	0.93
p.Met1Ile	Met	1	Ile	1
p.Arg167Gln	Arg	167	Gln	1
p.Arg167Trp	Arg	167	Trp	3
p.Arg195Cysys	Arg	195	Cysys	3
p.Ala252Val	Ala	252	Val	61
p.Ala331Val	Ala	331	Val	62
p.Thr59Ile	Thr	59	Ile	77
p.Asp230Tyr	Asp	230	Tyr	88
p.Asp359Asn	Asp	359	Asn	86
p.Glylu86Val	Glylu	86	Val	94

p.Arg225Gln	Arg	225	Gln	102
p.Arg321His	Arg	321	His	122
p.Leu161Pro	Leu	161	Pro	1
p.Arg251Ser	Arg	251	Ser	1
p.Ala122Pro	Ala	122	Pro	2
p.Asn118Lys	Asn	118	Lys	73
p.Gly3Val	Gly	3	Val	34
p.Arg164Gly	Arg	164	Gly	61
p.Ile71Thr	Ile	71	Thr	62
p.Gly72Arg	Gly	72	Arg	69
p.Gly221Ser	Gly	221	Ser	69
p.Ile54Leu	Ile	54	Leu	71
p.Gly274Ala	Gly	274	Ala	72
p.Val282Leu	Val	282	Leu	73
p.Lys115Gln	Lys	115	Gln	76
p.Gly111Ala	Gly	111	Ala	76
p.Ala297Ser	Ala	297	Ser	76
p.Arg321Cys	Arg	321	Cys	77
p.Asp273Gly	Asp	273	Gly	78
p.Ala9Glylu	Ala	9	Glylu	79
p.Pro309Ser	Pro	309	Ser	79
p.Thr10Met	Thr	10	Met	79
p.Arg355Trp	Arg	355	Trp	80
p.Arg22Cys	Arg	22	Cys	80
p.Arg164Ser	Arg	164	Ser	81
p.Pro159Leu	Pro	159	Leu	81
p.Ile205Met	Ile	205	Met	83
p.Glu242Lys	Glu	242	Lys	83
p.Pro127Ala	Pro	127	Ala	84
p.Thr10Lys	Thr	10	Lys	86
p.Pro127Gln	Pro	127	Gln	87
p.Lys132Asn	Lys	132	Asn	87
p.Ser45Leu	Ser	45	Leu	88
p.Arg325Gln	Arg	325	Gln	88
p.Gln296Arg	Gln	296	Arg	92
p.Asp65His	Asp	65	His	93
p.His256Gln	His	256	Gln	93
p.His120Asn	His	120	Asn	95
p.Ser262Asn	Ser	262	Asn	96
p.Arg32Cysys	Arg	32	Cysys	98
p.Arg355Gln	Arg	355	Gln	98
p.Lys348Arg	Lys	348	Arg	100
p.Glu86Lys	Glu	86	Lys	104
p.Glu12Ala	Glu	12	Ala	106
p.Arg246His	Arg	246	His	122

p.Val237Met	Val	237	Met	128
p.Arg246Cys	Arg	246	Cys	130
p.Gly3Ser	Gly	3	Ser	211

d wild-type HMBS activity

Sources and References

Lenglet, H. et al. From a dominant to an oligogenic model of inheritance with environmental
Lenglet, H. et al. From a dominant to an oligogenic model of inheritance with environmental
Lenglet, H. et al. From a dominant to an oligogenic model of inheritance with environmental
Lenglet, H. et al. From a dominant to an oligogenic model of inheritance with environmental
Lenglet, H. et al. From a dominant to an oligogenic model of inheritance with environmental
Lenglet, H. et al. From a dominant to an oligogenic model of inheritance with environmental
Lenglet, H. et al. From a dominant to an oligogenic model of inheritance with environmental
Lenglet, H. et al. From a dominant to an oligogenic model of inheritance with environmental
Lenglet, H. et al. From a dominant to an oligogenic model of inheritance with environmental
Lenglet, H. et al. From a dominant to an oligogenic model of inheritance with environmental
Lenglet, H. et al. From a dominant to an oligogenic model of inheritance with environmental
Bung, N. et al. Human hydroxymethylbilane synthase: Molecular dynamics of the pyrrole chai
Lenglet, H. et al. From a dominant to an oligogenic model of inheritance with environmental
Lenglet, H. et al. From a dominant to an oligogenic model of inheritance with environmental
Bustad, H.J., et al. Conformational stability and activity analysis of two hydroxymethylbilane s
Bustad, H.J., et al. Conformational stability and activity analysis of two hydroxymethylbilane s
Bustad, H.J., et al. Conformational stability and activity analysis of two hydroxymethylbilane s
Bustad, H.J., et al. Conformational stability and activity analysis of two hydroxymethylbilane s
Chen, B. et al. Identification and characterization of 40 novel hydroxymethylbilane synthase r
Bustad, H.J., et al. Conformational stability and activity analysis of two hydroxymethylbilane s
Bustad, H.J., et al. Conformational stability and activity analysis of two hydroxymethylbilane s
Bustad, H.J. et al. Acute intermittent porphyria: an overview of therapy developments and fu
Bustad, H.J. et al. Acute intermittent porphyria: an overview of therapy developments and fu
Bustad, H.J. et al. Acute intermittent porphyria: an overview of therapy developments and fu
Bustad, H.J. et al. Acute intermittent porphyria: an overview of therapy developments and fu
Bustad, H.J. et al. Acute intermittent porphyria: an overview of therapy developments and fu
Bustad, H.J. et al. Acute intermittent porphyria: an overview of therapy developments and fu
Bustad, H.J. et al. Acute intermittent porphyria: an overview of therapy developments and fu
Bustad, H.J. et al. Acute intermittent porphyria: an overview of therapy developments and fu
Bustad, H.J. et al. Acute intermittent porphyria: an overview of therapy developments and fu
Bustad, H.J. et al. Acute intermittent porphyria: an overview of therapy developments and fu
Bustad, H.J. et al. Acute intermittent porphyria: an overview of therapy developments and fu
Bustad, H.J. et al. Acute intermittent porphyria: an overview of therapy developments and fu
Bustad, H.J. et al. Acute intermittent porphyria: an overview of therapy developments and fu
Bustad, H.J. et al. Acute intermittent porphyria: an overview of therapy developments and fu
Bustad, H.J. et al. Acute intermittent porphyria: an overview of therapy developments and fu
Bustad, H.J. et al. Acute intermittent porphyria: an overview of therapy developments and fu
Bustad, H.J. et al. Acute intermittent porphyria: an overview of therapy developments and fu
Bustad, H.J. et al. Acute intermittent porphyria: an overview of therapy developments and fu
Bustad, H.J. et al. Acute intermittent porphyria: an overview of therapy developments and fu
Bustad, H.J. et al. Acute intermittent porphyria: an overview of therapy developments and fu
Bustad, H.J. et al. Acute intermittent porphyria: an overview of therapy developments and fu

Bustad, H.J. et al. Acute intermittent porphyria: an overview of therapy developments and fu
Bustad, H.J. et al. Acute intermittent porphyria: an overview of therapy developments and fu
Bustad, H.J. et al. Acute intermittent porphyria: an overview of therapy developments and fu
Bustad, H.J. et al. Acute intermittent porphyria: an overview of therapy developments and fu
Bustad, H.J. et al. Acute intermittent porphyria: an overview of therapy developments and fu
Bustad, H.J. et al. Acute intermittent porphyria: an overview of therapy developments and fu
Bustad, H.J. et al. Acute intermittent porphyria: an overview of therapy developments and fu
Bustad, H.J. et al. Acute intermittent porphyria: an overview of therapy developments and fu
Bustad, H.J. et al. Acute intermittent porphyria: an overview of therapy developments and fu
Bustad, H.J. et al. Acute intermittent porphyria: an overview of therapy developments and fu
Bustad, H.J. et al. Acute intermittent porphyria: an overview of therapy developments and fu
Bustad, H.J. et al. Acute intermittent porphyria: an overview of therapy developments and fu
Bustad, H.J. et al. Acute intermittent porphyria: an overview of therapy developments and fu
Bustad, H.J. et al. Acute intermittent porphyria: an overview of therapy developments and fu
Bustad, H.J. et al. Acute intermittent porphyria: an overview of therapy developments and fu
Bustad, H.J. et al. Acute intermittent porphyria: an overview of therapy developments and fu
Bustad, H.J. et al. Acute intermittent porphyria: an overview of therapy developments and fu
Bustad, H.J. et al. Acute intermittent porphyria: an overview of therapy developments and fu
Bustad, H.J. et al. Acute intermittent porphyria: an overview of therapy developments and fu
Bustad, H.J. et al. Acute intermittent porphyria: an overview of therapy developments and fu
Bustad, H.J. et al. Acute intermittent porphyria: an overview of therapy developments and fu
Bustad, H.J. et al. Acute intermittent porphyria: an overview of therapy developments and fu
Bustad, H.J. et al. Acute intermittent porphyria: an overview of therapy developments and fu
Bustad, H.J. et al. Acute intermittent porphyria: an overview of therapy developments and fu
Bustad, H.J. et al. Acute intermittent porphyria: an overview of therapy developments and fu
Bustad, H.J. et al. Acute intermittent porphyria: an overview of therapy developments and fu
Bustad, H.J. et al. Acute intermittent porphyria: an overview of therapy developments and fu
Bustad, H.J. et al. Acute intermittent porphyria: an overview of therapy developments and fu
Bustad, H.J. et al. Acute intermittent porphyria: an overview of therapy developments and fu
Bustad, H.J. et al. Acute intermittent porphyria: an overview of therapy developments and fu
Bustad, H.J. et al. Acute intermittent porphyria: an overview of therapy developments and fu
Bustad, H.J. et al. Acute intermittent porphyria: an overview of therapy developments and fu
Bustad, H.J. et al. Acute intermittent porphyria: an overview of therapy developments and fu
Bustad, H.J. et al. Acute intermittent porphyria: an overview of therapy developments and fu
Bustad, H.J. et al. Acute intermittent porphyria: an overview of therapy developments and fu
Chen, B. et al. Acute Intermittent Porphyria: Predicted Pathogenicity of HMBS Variants Indica
Chen, B. et al. Acute Intermittent Porphyria: Predicted Pathogenicity of HMBS Variants Indica
Chen, B. et al. Acute Intermittent Porphyria: Predicted Pathogenicity of HMBS Variants Indica
Chen, B. et al. Acute Intermittent Porphyria: Predicted Pathogenicity of HMBS Variants Indica
Chen, B. et al. Acute Intermittent Porphyria: Predicted Pathogenicity of HMBS Variants Indica
Chen, B. et al. Acute Intermittent Porphyria: Predicted Pathogenicity of HMBS Variants Indica
Chen, B. et al. Acute Intermittent Porphyria: Predicted Pathogenicity of HMBS Variants Indica
Chen, B. et al. Acute Intermittent Porphyria: Predicted Pathogenicity of HMBS Variants Indica
Chen, B. et al. Acute Intermittent Porphyria: Predicted Pathogenicity of HMBS Variants Indica
Chen, B. et al. Acute Intermittent Porphyria: Predicted Pathogenicity of HMBS Variants Indica

Chen, B. et al. Acute Intermittent Porphyria: Predicted Pathogenicity of HMBS Variants Indica
Chen, B. et al. Acute Intermittent Porphyria: Predicted Pathogenicity of HMBS Variants Indica
Chen, B. et al. Acute Intermittent Porphyria: Predicted Pathogenicity of HMBS Variants Indica
Chen, B. et al. Acute Intermittent Porphyria: Predicted Pathogenicity of HMBS Variants Indica
Chen, B. et al. Acute Intermittent Porphyria: Predicted Pathogenicity of HMBS Variants Indica
Chen, B. et al. Acute Intermittent Porphyria: Predicted Pathogenicity of HMBS Variants Indica
Chen, B. et al. Acute Intermittent Porphyria: Predicted Pathogenicity of HMBS Variants Indica
Chen, B. et al. Acute Intermittent Porphyria: Predicted Pathogenicity of HMBS Variants Indica
Chen, B. et al. Acute Intermittent Porphyria: Predicted Pathogenicity of HMBS Variants Indica
Chen, B. et al. Acute Intermittent Porphyria: Predicted Pathogenicity of HMBS Variants Indica
Chen, B. et al. Acute Intermittent Porphyria: Predicted Pathogenicity of HMBS Variants Indica
Chen, B. et al. Acute Intermittent Porphyria: Predicted Pathogenicity of HMBS Variants Indica
Chen, B. et al. Acute Intermittent Porphyria: Predicted Pathogenicity of HMBS Variants Indica
Chen, B. et al. Acute Intermittent Porphyria: Predicted Pathogenicity of HMBS Variants Indica
Chen, B. et al. Acute Intermittent Porphyria: Predicted Pathogenicity of HMBS Variants Indica
Chen, B. et al. Acute Intermittent Porphyria: Predicted Pathogenicity of HMBS Variants Indica
Chen, B. et al. Acute Intermittent Porphyria: Predicted Pathogenicity of HMBS Variants Indica
Chen, B. et al. Acute Intermittent Porphyria: Predicted Pathogenicity of HMBS Variants Indica
Chen, B. et al. Acute Intermittent Porphyria: Predicted Pathogenicity of HMBS Variants Indica
Chen, B. et al. Acute Intermittent Porphyria: Predicted Pathogenicity of HMBS Variants Indica
Chen, B. et al. Acute Intermittent Porphyria: Predicted Pathogenicity of HMBS Variants Indica
Chen, B. et al. Acute Intermittent Porphyria: Predicted Pathogenicity of HMBS Variants Indica
Chen, B. et al. Acute Intermittent Porphyria: Predicted Pathogenicity of HMBS Variants Indica
Chen, B. et al. Acute Intermittent Porphyria: Predicted Pathogenicity of HMBS Variants Indica
Chen, B. et al. Acute Intermittent Porphyria: Predicted Pathogenicity of HMBS Variants Indica
Chen, B. et al. Acute Intermittent Porphyria: Predicted Pathogenicity of HMBS Variants Indica
Chen, B. et al. Acute Intermittent Porphyria: Predicted Pathogenicity of HMBS Variants Indica
Chen, B. et al. Acute Intermittent Porphyria: Predicted Pathogenicity of HMBS Variants Indica
Chen, B. et al. Acute Intermittent Porphyria: Predicted Pathogenicity of HMBS Variants Indica
Chen, B. et al. Acute Intermittent Porphyria: Predicted Pathogenicity of HMBS Variants Indica
Chen, B. et al. Acute Intermittent Porphyria: Predicted Pathogenicity of HMBS Variants Indica
Chen, B. et al. Acute Intermittent Porphyria: Predicted Pathogenicity of HMBS Variants Indica
Chen, B. et al. Acute Intermittent Porphyria: Predicted Pathogenicity of HMBS Variants Indica
Chen, B. et al. Acute Intermittent Porphyria: Predicted Pathogenicity of HMBS Variants Indica
Chen, B. et al. Acute Intermittent Porphyria: Predicted Pathogenicity of HMBS Variants Indica
Chen, B. et al. Acute Intermittent Porphyria: Predicted Pathogenicity of HMBS Variants Indica
Chen, B. et al. Acute Intermittent Porphyria: Predicted Pathogenicity of HMBS Variants Indica
Chen, B. et al. Acute Intermittent Porphyria: Predicted Pathogenicity of HMBS Variants Indica
Chen, B. et al. Acute Intermittent Porphyria: Predicted Pathogenicity of HMBS Variants Indica
Chen, B. et al. Acute Intermittent Porphyria: Predicted Pathogenicity of HMBS Variants Indica
Chen, B. et al. Acute Intermittent Porphyria: Predicted Pathogenicity of HMBS Variants Indica
Chen, B. et al. Acute Intermittent Porphyria: Predicted Pathogenicity of HMBS Variants Indica
Chen, B. et al. Acute Intermittent Porphyria: Predicted Pathogenicity of HMBS Variants Indica
Chen, B. et al. Acute Intermittent Porphyria: Predicted Pathogenicity of HMBS Variants Indica
Chen, B. et al. Acute Intermittent Porphyria: Predicted Pathogenicity of HMBS Variants Indica

Chen, B. et al. Acute Intermittent Porphyria: Predicted Pathogenicity of HMBS Variants Indica
Chen, B. et al. Acute Intermittent Porphyria: Predicted Pathogenicity of HMBS Variants Indica
Chen, B. et al. Acute Intermittent Porphyria: Predicted Pathogenicity of HMBS Variants Indica

modifiers in acute intermittent porphyria. Hum. Mol. Genet. 27, 1164-1173 (2018)
modifiers in acute intermittent porphyria. Hum. Mol. Genet. 27, 1164-1173 (2018)
modifiers in acute intermittent porphyria. Hum. Mol. Genet. 27, 1164-1173 (2018)
modifiers in acute intermittent porphyria. Hum. Mol. Genet. 27, 1164-1173 (2018)
modifiers in acute intermittent porphyria. Hum. Mol. Genet. 27, 1164-1173 (2018)
modifiers in acute intermittent porphyria. Hum. Mol. Genet. 27, 1164-1173 (2018)
modifiers in acute intermittent porphyria. Hum. Mol. Genet. 27, 1164-1173 (2018)
modifiers in acute intermittent porphyria. Hum. Mol. Genet. 27, 1164-1173 (2018)
modifiers in acute intermittent porphyria. Hum. Mol. Genet. 27, 1164-1173 (2018)
modifiers in acute intermittent porphyria. Hum. Mol. Genet. 27, 1164-1173 (2018)
modifiers in acute intermittent porphyria. Hum. Mol. Genet. 27, 1164-1173 (2018)
n elongation identifies step-specific residues that cause AIP. Proc. Natl. Acad. Sci. U.S.A. 115,
modifiers in acute intermittent porphyria. Hum. Mol. Genet. 27, 1164-1173 (2018)
modifiers in acute intermittent porphyria. Hum. Mol. Genet. 27, 1164-1173 (2018)
synthase mutants, K132N and V215E, with different phenotypic association with acute interm
synthase mutants, K132N and V215E, with different phenotypic association with acute interm
synthase mutants, K132N and V215E, with different phenotypic association with acute interm
synthase mutants, K132N and V215E, with different phenotypic association with acute interm
nutations that cause acute intermittent porphyria. J. Inherit. Metab. Dis. 42, 186,Äì194 (2019)
synthase mutants, K132N and V215E, with different phenotypic association with acute interm
synthase mutants, K132N and V215E, with different phenotypic association with acute interm
ture perspectives focusing on stabilisation of HMBS and proteostasis regulators. Int. J. Mol. 2
ture perspectives focusing on stabilisation of HMBS and proteostasis regulators. Int. J. Mol. 2
ture perspectives focusing on stabilisation of HMBS and proteostasis regulators. Int. J. Mol. 2
ture perspectives focusing on stabilisation of HMBS and proteostasis regulators. Int. J. Mol. 2
ture perspectives focusing on stabilisation of HMBS and proteostasis regulators. Int. J. Mol. 2
ture perspectives focusing on stabilisation of HMBS and proteostasis regulators. Int. J. Mol. 2
ture perspectives focusing on stabilisation of HMBS and proteostasis regulators. Int. J. Mol. 2
ture perspectives focusing on stabilisation of HMBS and proteostasis regulators. Int. J. Mol. 2
ture perspectives focusing on stabilisation of HMBS and proteostasis regulators. Int. J. Mol. 2
ture perspectives focusing on stabilisation of HMBS and proteostasis regulators. Int. J. Mol. 2
ture perspectives focusing on stabilisation of HMBS and proteostasis regulators. Int. J. Mol. 2
ture perspectives focusing on stabilisation of HMBS and proteostasis regulators. Int. J. Mol. 2
ture perspectives focusing on stabilisation of HMBS and proteostasis regulators. Int. J. Mol. 2
ture perspectives focusing on stabilisation of HMBS and proteostasis regulators. Int. J. Mol. 2
ture perspectives focusing on stabilisation of HMBS and proteostasis regulators. Int. J. Mol. 2
ture perspectives focusing on stabilisation of HMBS and proteostasis regulators. Int. J. Mol. 2
ture perspectives focusing on stabilisation of HMBS and proteostasis regulators. Int. J. Mol. 2
ture perspectives focusing on stabilisation of HMBS and proteostasis regulators. Int. J. Mol. 2
ture perspectives focusing on stabilisation of HMBS and proteostasis regulators. Int. J. Mol. 2
ture perspectives focusing on stabilisation of HMBS and proteostasis regulators. Int. J. Mol. 2
ture perspectives focusing on stabilisation of HMBS and proteostasis regulators. Int. J. Mol. 2
ture perspectives focusing on stabilisation of HMBS and proteostasis regulators. Int. J. Mol. 2

ture perspectives focusing on stabilisation of HMBS and proteostasis regulators. Int. J. Mol. 2
ture perspectives focusing on stabilisation of HMBS and proteostasis regulators. Int. J. Mol. 2
ture perspectives focusing on stabilisation of HMBS and proteostasis regulators. Int. J. Mol. 2
ture perspectives focusing on stabilisation of HMBS and proteostasis regulators. Int. J. Mol. 2
ture perspectives focusing on stabilisation of HMBS and proteostasis regulators. Int. J. Mol. 2
ture perspectives focusing on stabilisation of HMBS and proteostasis regulators. Int. J. Mol. 2
ture perspectives focusing on stabilisation of HMBS and proteostasis regulators. Int. J. Mol. 2
ture perspectives focusing on stabilisation of HMBS and proteostasis regulators. Int. J. Mol. 2
ture perspectives focusing on stabilisation of HMBS and proteostasis regulators. Int. J. Mol. 2
ture perspectives focusing on stabilisation of HMBS and proteostasis regulators. Int. J. Mol. 2
ture perspectives focusing on stabilisation of HMBS and proteostasis regulators. Int. J. Mol. 2
ture perspectives focusing on stabilisation of HMBS and proteostasis regulators. Int. J. Mol. 2
ture perspectives focusing on stabilisation of HMBS and proteostasis regulators. Int. J. Mol. 2
ture perspectives focusing on stabilisation of HMBS and proteostasis regulators. Int. J. Mol. 2
ture perspectives focusing on stabilisation of HMBS and proteostasis regulators. Int. J. Mol. 2
ture perspectives focusing on stabilisation of HMBS and proteostasis regulators. Int. J. Mol. 2
ture perspectives focusing on stabilisation of HMBS and proteostasis regulators. Int. J. Mol. 2
ture perspectives focusing on stabilisation of HMBS and proteostasis regulators. Int. J. Mol. 2
ture perspectives focusing on stabilisation of HMBS and proteostasis regulators. Int. J. Mol. 2
ture perspectives focusing on stabilisation of HMBS and proteostasis regulators. Int. J. Mol. 2
ture perspectives focusing on stabilisation of HMBS and proteostasis regulators. Int. J. Mol. 2
ture perspectives focusing on stabilisation of HMBS and proteostasis regulators. Int. J. Mol. 2
ture perspectives focusing on stabilisation of HMBS and proteostasis regulators. Int. J. Mol. 2
ture perspectives focusing on stabilisation of HMBS and proteostasis regulators. Int. J. Mol. 2
ture perspectives focusing on stabilisation of HMBS and proteostasis regulators. Int. J. Mol. 2
ture perspectives focusing on stabilisation of HMBS and proteostasis regulators. Int. J. Mol. 2
ture perspectives focusing on stabilisation of HMBS and proteostasis regulators. Int. J. Mol. 2
ture perspectives focusing on stabilisation of HMBS and proteostasis regulators. Int. J. Mol. 2
ture perspectives focusing on stabilisation of HMBS and proteostasis regulators. Int. J. Mol. 2
ture perspectives focusing on stabilisation of HMBS and proteostasis regulators. Int. J. Mol. 2
ture perspectives focusing on stabilisation of HMBS and proteostasis regulators. Int. J. Mol. 2
ture perspectives focusing on stabilisation of HMBS and proteostasis regulators. Int. J. Mol. 2
ture perspectives focusing on stabilisation of HMBS and proteostasis regulators. Int. J. Mol. 2
ture perspectives focusing on stabilisation of HMBS and proteostasis regulators. Int. J. Mol. 2
ture perspectives focusing on stabilisation of HMBS and proteostasis regulators. Int. J. Mol. 2
tes Extremely Low Penetrance of the Autosomal Dominant Disease. Hum. Mutat. 37, 1215,Äì
tes Extremely Low Penetrance of the Autosomal Dominant Disease. Hum. Mutat. 37, 1215,Äì
tes Extremely Low Penetrance of the Autosomal Dominant Disease. Hum. Mutat. 37, 1215,Äì
tes Extremely Low Penetrance of the Autosomal Dominant Disease. Hum. Mutat. 37, 1215,Äì
tes Extremely Low Penetrance of the Autosomal Dominant Disease. Hum. Mutat. 37, 1215,Äì
tes Extremely Low Penetrance of the Autosomal Dominant Disease. Hum. Mutat. 37, 1215,Äì
tes Extremely Low Penetrance of the Autosomal Dominant Disease. Hum. Mutat. 37, 1215,Äì
tes Extremely Low Penetrance of the Autosomal Dominant Disease. Hum. Mutat. 37, 1215,Äì
tes Extremely Low Penetrance of the Autosomal Dominant Disease. Hum. Mutat. 37, 1215,Äì
tes Extremely Low Penetrance of the Autosomal Dominant Disease. Hum. Mutat. 37, 1215,Äì

tes Extremely Low Penetrance of the Autosomal Dominant Disease. Hum. Mutat. 37, 1215,Äì
tes Extremely Low Penetrance of the Autosomal Dominant Disease. Hum. Mutat. 37, 1215,Äì
tes Extremely Low Penetrance of the Autosomal Dominant Disease. Hum. Mutat. 37, 1215,Äì
tes Extremely Low Penetrance of the Autosomal Dominant Disease. Hum. Mutat. 37, 1215,Äì
tes Extremely Low Penetrance of the Autosomal Dominant Disease. Hum. Mutat. 37, 1215,Äì
tes Extremely Low Penetrance of the Autosomal Dominant Disease. Hum. Mutat. 37, 1215,Äì
tes Extremely Low Penetrance of the Autosomal Dominant Disease. Hum. Mutat. 37, 1215,Äì
tes Extremely Low Penetrance of the Autosomal Dominant Disease. Hum. Mutat. 37, 1215,Äì
tes Extremely Low Penetrance of the Autosomal Dominant Disease. Hum. Mutat. 37, 1215,Äì
tes Extremely Low Penetrance of the Autosomal Dominant Disease. Hum. Mutat. 37, 1215,Äì
tes Extremely Low Penetrance of the Autosomal Dominant Disease. Hum. Mutat. 37, 1215,Äì
tes Extremely Low Penetrance of the Autosomal Dominant Disease. Hum. Mutat. 37, 1215,Äì
tes Extremely Low Penetrance of the Autosomal Dominant Disease. Hum. Mutat. 37, 1215,Äì
tes Extremely Low Penetrance of the Autosomal Dominant Disease. Hum. Mutat. 37, 1215,Äì
tes Extremely Low Penetrance of the Autosomal Dominant Disease. Hum. Mutat. 37, 1215,Äì
tes Extremely Low Penetrance of the Autosomal Dominant Disease. Hum. Mutat. 37, 1215,Äì
tes Extremely Low Penetrance of the Autosomal Dominant Disease. Hum. Mutat. 37, 1215,Äì
tes Extremely Low Penetrance of the Autosomal Dominant Disease. Hum. Mutat. 37, 1215,Äì
tes Extremely Low Penetrance of the Autosomal Dominant Disease. Hum. Mutat. 37, 1215,Äì
tes Extremely Low Penetrance of the Autosomal Dominant Disease. Hum. Mutat. 37, 1215,Äì
tes Extremely Low Penetrance of the Autosomal Dominant Disease. Hum. Mutat. 37, 1215,Äì
tes Extremely Low Penetrance of the Autosomal Dominant Disease. Hum. Mutat. 37, 1215,Äì
tes Extremely Low Penetrance of the Autosomal Dominant Disease. Hum. Mutat. 37, 1215,Äì
tes Extremely Low Penetrance of the Autosomal Dominant Disease. Hum. Mutat. 37, 1215,Äì
tes Extremely Low Penetrance of the Autosomal Dominant Disease. Hum. Mutat. 37, 1215,Äì
tes Extremely Low Penetrance of the Autosomal Dominant Disease. Hum. Mutat. 37, 1215,Äì
tes Extremely Low Penetrance of the Autosomal Dominant Disease. Hum. Mutat. 37, 1215,Äì
tes Extremely Low Penetrance of the Autosomal Dominant Disease. Hum. Mutat. 37, 1215,Äì
tes Extremely Low Penetrance of the Autosomal Dominant Disease. Hum. Mutat. 37, 1215,Äì
tes Extremely Low Penetrance of the Autosomal Dominant Disease. Hum. Mutat. 37, 1215,Äì
tes Extremely Low Penetrance of the Autosomal Dominant Disease. Hum. Mutat. 37, 1215,Äì
tes Extremely Low Penetrance of the Autosomal Dominant Disease. Hum. Mutat. 37, 1215,Äì
tes Extremely Low Penetrance of the Autosomal Dominant Disease. Hum. Mutat. 37, 1215,Äì
tes Extremely Low Penetrance of the Autosomal Dominant Disease. Hum. Mutat. 37, 1215,Äì
tes Extremely Low Penetrance of the Autosomal Dominant Disease. Hum. Mutat. 37, 1215,Äì
tes Extremely Low Penetrance of the Autosomal Dominant Disease. Hum. Mutat. 37, 1215,Äì
tes Extremely Low Penetrance of the Autosomal Dominant Disease. Hum. Mutat. 37, 1215,Äì
tes Extremely Low Penetrance of the Autosomal Dominant Disease. Hum. Mutat. 37, 1215,Äì
tes Extremely Low Penetrance of the Autosomal Dominant Disease. Hum. Mutat. 37, 1215,Äì
tes Extremely Low Penetrance of the Autosomal Dominant Disease. Hum. Mutat. 37, 1215,Äì
tes Extremely Low Penetrance of the Autosomal Dominant Disease. Hum. Mutat. 37, 1215,Äì
tes Extremely Low Penetrance of the Autosomal Dominant Disease. Hum. Mutat. 37, 1215,Äì
tes Extremely Low Penetrance of the Autosomal Dominant Disease. Hum. Mutat. 37, 1215,Äì
tes Extremely Low Penetrance of the Autosomal Dominant Disease. Hum. Mutat. 37, 1215,Äì
tes Extremely Low Penetrance of the Autosomal Dominant Disease. Hum. Mutat. 37, 1215,Äì

tes Extremely Low Penetrance of the Autosomal Dominant Disease. Hum. Mutat. 37, 1215,Äì
tes Extremely Low Penetrance of the Autosomal Dominant Disease. Hum. Mutat. 37, 1215,Äì
tes Extremely Low Penetrance of the Autosomal Dominant Disease. Hum. Mutat. 37, 1215,Äì

, E4071,ÄìE4080 (2018)

ıittent porphyria. Bioscience reports. 33 (2013)
ıittent porphyria. Bioscience reports. 33 (2013)
ıittent porphyria. Bioscience reports. 33 (2013)
ıittent porphyria. Bioscience reports. 33 (2013)
)
ıittent porphyria. Bioscience reports. 33 (2013)
ıittent porphyria. Bioscience reports. 33 (2013)
2, 675 (2021)
2, 675 (2021)
2, 675 (2021)
2, 675 (2021)
2, 675 (2021)
2, 675 (2021)
2, 675 (2021)
2, 675 (2021)
2, 675 (2021)
2, 675 (2021)
2, 675 (2021)
2, 675 (2021)
2, 675 (2021)
2, 675 (2021)
2, 675 (2021)
2, 675 (2021)
2, 675 (2021)
2, 675 (2021)
2, 675 (2021)
2, 675 (2021)
2, 675 (2021)
2, 675 (2021)

2, 675 (2021)
2, 675 (2021)
2, 675 (2021)
2, 675 (2021)
2, 675 (2021)
2, 675 (2021)
2, 675 (2021)
2, 675 (2021)
2, 675 (2021)
2, 675 (2021)
2, 675 (2021)
2, 675 (2021)
2, 675 (2021)
2, 675 (2021)
2, 675 (2021)
2, 675 (2021)
2, 675 (2021)
2, 675 (2021)
2, 675 (2021)
2, 675 (2021)
2, 675 (2021)
2, 675 (2021)
2, 675 (2021)
2, 675 (2021)
2, 675 (2021)
2, 675 (2021)
2, 675 (2021)
2, 675 (2021)
2, 675 (2021)
2, 675 (2021)
2, 675 (2021)
2, 675 (2021)
2, 675 (2021)
2, 675 (2021)
2, 675 (2021)
1222 (2016)
1222 (2016)
1222 (2016)
1222 (2016)
1222 (2016)
1222 (2016)
1222 (2016)
1222 (2016)
1222 (2016)
1222 (2016)

1222 (2016)
1222 (2016)
1222 (2016)
1222 (2016)
1222 (2016)
1222 (2016)
1222 (2016)
1222 (2016)
1222 (2016)
1222 (2016)
1222 (2016)
1222 (2016)
1222 (2016)
1222 (2016)
1222 (2016)
1222 (2016)
1222 (2016)
1222 (2016)
1222 (2016)
1222 (2016)
1222 (2016)
1222 (2016)
1222 (2016)
1222 (2016)
1222 (2016)
1222 (2016)
1222 (2016)
1222 (2016)
1222 (2016)
1222 (2016)
1222 (2016)
1222 (2016)
1222 (2016)
1222 (2016)
1222 (2016)
1222 (2016)
1222 (2016)
1222 (2016)
1222 (2016)
1222 (2016)
1222 (2016)
1222 (2016)
1222 (2016)
1222 (2016)
1222 (2016)

1222 (2016)
1222 (2016)
1222 (2016)
