# Supplementary material for: Systematically testing human HMBS missense variants to reveal mechanism and pathogenic variation
Source: bioRxiv. 2023 Feb 6:2023.02.06.527353. Preprint. [Version 1] doi: 10.1101/2023.02.06.527353 (PMC9934555; doi:10.1101/2023.02.06.527353)
Supplement: Supplement 3 [file media-3.pdf]

Table S8: Log likelihood ratios of pathogenicity derived from the combine HMBS map.

Variant					Combined Map	
**HGVS**	**type**	**pos**	**wt aa**	**mut aa**	**score**	**stderr**
p.Ala112=	synonymous	112	Ala	NA	1.015	0.003
p.Ala112Arg	substitution	112	Ala	Arg	0.000	0.151
p.Ala112Asn	substitution	112	Ala	Asn	0.000	0.542
p.Ala112Asp	substitution	112	Ala	Asp	0.122	0.048
p.Ala112Cys	substitution	112	Ala	Cys	1.107	0.020
p.Ala112Gln	substitution	112	Ala	Gln	0.548	0.021
p.Ala112Glu	substitution	112	Ala	Glu	0.000	0.077
p.Ala112Gly	substitution	112	Ala	Gly	0.597	0.035
p.Ala112His	substitution	112	Ala	His	0.000	0.030
p.Ala112Ile	substitution	112	Ala	Ile	0.000	0.024
p.Ala112Leu	substitution	112	Ala	Leu	0.598	0.064
p.Ala112Lys	substitution	112	Ala	Lys	0.000	0.040
p.Ala112Met	substitution	112	Ala	Met	0.000	0.027
p.Ala112Phe	substitution	112	Ala	Phe	0.000	0.028
p.Ala112Pro	substitution	112	Ala	Pro	0.319	0.058
p.Ala112Ser	substitution	112	Ala	Ser	0.737	0.028
p.Ala112Ter	nonsense	112	Ala	Ter	0.318	0.018
p.Ala112Thr	substitution	112	Ala	Thr	0.760	0.020
p.Ala112Trp	substitution	112	Ala	Trp	0.000	0.034
p.Ala112Tyr	substitution	112	Ala	Tyr	0.227	0.032
p.Ala112Val	substitution	112	Ala	Val	0.508	0.057
p.Ala122=	synonymous	122	Ala	NA	1.115	0.022
p.Ala122Arg	substitution	122	Ala	Arg	0.000	0.027
p.Ala122Asn	substitution	122	Ala	Asn	0.875	0.015
p.Ala122Asp	substitution	122	Ala	Asp	0.306	0.009
p.Ala122Gln	substitution	122	Ala	Gln	0.396	0.020
p.Ala122Glu	substitution	122	Ala	Glu	0.277	0.024
p.Ala122Gly	substitution	122	Ala	Gly	0.854	0.008
p.Ala122His	substitution	122	Ala	His	0.205	0.012
p.Ala122Ile	substitution	122	Ala	Ile	0.106	0.031
p.Ala122Leu	substitution	122	Ala	Leu	0.254	0.036
p.Ala122Lys	substitution	122	Ala	Lys	0.109	0.034
p.Ala122Met	substitution	122	Ala	Met	0.609	0.022
p.Ala122Phe	substitution	122	Ala	Phe	0.059	0.029
p.Ala122Pro	substitution	122	Ala	Pro	0.231	0.029
p.Ala122Ser	substitution	122	Ala	Ser	0.783	0.060
p.Ala122Ter	nonsense	122	Ala	Ter	0.000	0.024
p.Ala122Thr	substitution	122	Ala	Thr	0.800	0.011
p.Ala122Trp	substitution	122	Ala	Trp	0.000	0.021
p.Ala122Tyr	substitution	122	Ala	Tyr	0.083	0.015

p.Ala122Val	substitution	122	Ala	Val		0.997	0.003
p.Ala151=	synonymous	151	Ala	NA		0.795	0.008
p.Ala151Arg	substitution	151	Ala	Arg		0.898	0.008
p.Ala151Asn	substitution	151	Ala	Asn		0.946	0.011
p.Ala151Asp	substitution	151	Ala	Asp		0.947	0.009
p.Ala151Cys	substitution	151	Ala	Cys		0.849	0.008
p.Ala151Gln	substitution	151	Ala	Gln		1.170	0.014
p.Ala151Glu	substitution	151	Ala	Glu		0.000	0.067
p.Ala151Gly	substitution	151	Ala	Gly		0.900	0.009
p.Ala151His	substitution	151	Ala	His		0.880	0.007
p.Ala151Ile	substitution	151	Ala	Ile		0.358	0.131
p.Ala151Leu	substitution	151	Ala	Leu		0.907	0.022
p.Ala151Lys	substitution	151	Ala	Lys		0.642	0.010
p.Ala151Met	substitution	151	Ala	Met		0.113	0.033
p.Ala151Phe	substitution	151	Ala	Phe		0.773	0.023
p.Ala151Pro	substitution	151	Ala	Pro		0.180	0.093
p.Ala151Ser	substitution	151	Ala	Ser		0.785	0.061
p.Ala151Ter	nonsense	151	Ala	Ter		0.000	0.099
p.Ala151Thr	substitution	151	Ala	Thr		0.658	0.019
p.Ala151Trp	substitution	151	Ala	Trp		0.884	0.009
p.Ala151Tyr	substitution	151	Ala	Tyr		0.728	0.008
p.Ala151Val	substitution	151	Ala	Val		0.849	0.004
p.Ala152=	synonymous	152	Ala	NA		0.907	0.015
p.Ala152Arg	substitution	152	Ala	Arg		0.903	0.029
p.Ala152Asn	substitution	152	Ala	Asn		0.527	0.010
p.Ala152Asp	substitution	152	Ala	Asp		0.193	1.069
p.Ala152Cys	substitution	152	Ala	Cys		0.811	0.028
p.Ala152Gln	substitution	152	Ala	Gln		0.219	0.012
p.Ala152Glu	substitution	152	Ala	Glu		0.297	0.015
p.Ala152Gly	substitution	152	Ala	Gly		0.774	0.021
p.Ala152His	substitution	152	Ala	His		1.161	0.013
p.Ala152Ile	substitution	152	Ala	Ile		0.969	0.011
p.Ala152Leu	substitution	152	Ala	Leu		0.996	0.016
p.Ala152Lys	substitution	152	Ala	Lys		0.598	0.043
p.Ala152Met	substitution	152	Ala	Met		0.795	0.014
p.Ala152Phe	substitution	152	Ala	Phe		0.155	0.025
p.Ala152Pro	substitution	152	Ala	Pro		0.527	0.045
p.Ala152Ser	substitution	152	Ala	Ser		0.523	0.089
p.Ala152Thr	substitution	152	Ala	Thr		1.055	0.033
p.Ala152Trp	substitution	152	Ala	Trp		0.801	0.006
p.Ala152Tyr	substitution	152	Ala	Tyr		0.708	0.026
p.Ala152Val	substitution	152	Ala	Val		1.054	0.030
p.Ala185=	synonymous	185	Ala	NA		1.357	0.094

p.Ala185Asp	substitution	185	Ala	Asp	0.455	0.127
p.Ala185Gly	substitution	185	Ala	Gly	1.508	0.185
p.Ala185Ser	substitution	185	Ala	Ser	0.372	0.110
p.Ala185Thr	substitution	185	Ala	Thr	1.150	0.025
p.Ala185Val	substitution	185	Ala	Val	0.748	0.193
p.Ala189=	synonymous	189	Ala	NA	0.981	0.112
p.Ala189Arg	substitution	189	Ala	Arg	0.000	0.023
p.Ala189Asn	substitution	189	Ala	Asn	0.028	0.027
p.Ala189Asp	substitution	189	Ala	Asp	0.000	0.028
p.Ala189Cys	substitution	189	Ala	Cys	0.800	0.007
p.Ala189Gln	substitution	189	Ala	Gln	0.036	0.061
p.Ala189Glu	substitution	189	Ala	Glu	0.045	0.063
p.Ala189Gly	substitution	189	Ala	Gly	0.740	0.015
p.Ala189His	substitution	189	Ala	His	0.000	0.017
p.Ala189Ile	substitution	189	Ala	Ile	0.000	0.090
p.Ala189Leu	substitution	189	Ala	Leu	0.000	0.031
p.Ala189Lys	substitution	189	Ala	Lys	0.312	0.026
p.Ala189Met	substitution	189	Ala	Met	0.047	0.030
p.Ala189Phe	substitution	189	Ala	Phe	0.000	0.072
p.Ala189Pro	substitution	189	Ala	Pro	0.077	0.087
p.Ala189Ser	substitution	189	Ala	Ser	0.652	0.130
p.Ala189Ter	nonsense	189	Ala	Ter	0.328	0.202
p.Ala189Thr	substitution	189	Ala	Thr	0.333	0.088
p.Ala189Trp	substitution	189	Ala	Trp	0.000	0.127
p.Ala189Tyr	substitution	189	Ala	Tyr	0.000	0.019
p.Ala189Val	substitution	189	Ala	Val	0.000	0.010
p.Ala191=	synonymous	191	Ala	NA	0.802	0.039
p.Ala191Arg	substitution	191	Ala	Arg	0.000	0.057
p.Ala191Asp	substitution	191	Ala	Asp	0.201	0.021
p.Ala191Cys	substitution	191	Ala	Cys	0.194	0.031
p.Ala191Gln	substitution	191	Ala	Gln	0.000	0.231
p.Ala191Glu	substitution	191	Ala	Glu	0.000	0.049
p.Ala191Gly	substitution	191	Ala	Gly	0.416	0.008
p.Ala191His	substitution	191	Ala	His	0.000	0.217
p.Ala191Ile	substitution	191	Ala	Ile	0.000	0.031
p.Ala191Leu	substitution	191	Ala	Leu	0.000	0.019
p.Ala191Lys	substitution	191	Ala	Lys	0.198	0.084
p.Ala191Met	substitution	191	Ala	Met	0.000	0.021
p.Ala191Phe	substitution	191	Ala	Phe	0.133	0.014
p.Ala191Pro	substitution	191	Ala	Pro	0.000	0.033
p.Ala191Ser	substitution	191	Ala	Ser	0.656	0.043
p.Ala191Thr	substitution	191	Ala	Thr	0.711	0.005
p.Ala191Trp	substitution	191	Ala	Trp	0.000	0.050

p.Ala191Tyr	substitution	191	Ala	Tyr		0.125	0.087
p.Ala191Val	substitution	191	Ala	Val		0.000	0.066
p.Ala214=	synonymous	214	Ala	NA		0.707	0.022
p.Ala214Arg	substitution	214	Ala	Arg		0.000	0.012
p.Ala214Asn	substitution	214	Ala	Asn		0.426	0.005
p.Ala214Asp	substitution	214	Ala	Asp		0.427	0.012
p.Ala214Cys	substitution	214	Ala	Cys		0.783	0.007
p.Ala214Gln	substitution	214	Ala	Gln		0.000	0.038
p.Ala214Glu	substitution	214	Ala	Glu		0.254	0.005
p.Ala214Gly	substitution	214	Ala	Gly		0.675	0.008
p.Ala214Ile	substitution	214	Ala	Ile		0.114	0.007
p.Ala214Leu	substitution	214	Ala	Leu		0.002	0.017
p.Ala214Lys	substitution	214	Ala	Lys		0.000	0.012
p.Ala214Met	substitution	214	Ala	Met		1.168	0.001
p.Ala214Phe	substitution	214	Ala	Phe		0.364	0.011
p.Ala214Pro	substitution	214	Ala	Pro		0.692	0.011
p.Ala214Ser	substitution	214	Ala	Ser		0.516	0.063
p.Ala214Ter	nonsense	214	Ala	Ter		0.000	0.018
p.Ala214Thr	substitution	214	Ala	Thr		0.610	0.002
p.Ala214Trp	substitution	214	Ala	Trp		0.000	0.028
p.Ala214Tyr	substitution	214	Ala	Tyr		0.000	0.017
p.Ala214Val	substitution	214	Ala	Val		0.000	0.007
p.Ala219=	synonymous	219	Ala	NA		0.850	0.017
p.Ala219Arg	substitution	219	Ala	Arg		0.034	0.137
p.Ala219Asn	substitution	219	Ala	Asn		0.000	0.030
p.Ala219Asp	substitution	219	Ala	Asp		0.000	0.099
p.Ala219Cys	substitution	219	Ala	Cys		0.000	0.041
p.Ala219Glu	substitution	219	Ala	Glu		0.307	0.079
p.Ala219Gly	substitution	219	Ala	Gly		0.693	0.088
p.Ala219His	substitution	219	Ala	His		0.000	0.042
p.Ala219Leu	substitution	219	Ala	Leu		0.000	0.011
p.Ala219Lys	substitution	219	Ala	Lys		0.000	0.215
p.Ala219Phe	substitution	219	Ala	Phe		0.000	0.172
p.Ala219Pro	substitution	219	Ala	Pro		0.000	0.021
p.Ala219Ser	substitution	219	Ala	Ser		0.704	0.068
p.Ala219Thr	substitution	219	Ala	Thr		0.492	0.016
p.Ala219Tyr	substitution	219	Ala	Tyr		0.197	0.051
p.Ala219Val	substitution	219	Ala	Val		1.027	0.156
p.Ala226=	synonymous	226	Ala	NA		1.084	0.002
p.Ala226Arg	substitution	226	Ala	Arg		0.587	0.015
p.Ala226Asn	substitution	226	Ala	Asn		0.074	0.016
p.Ala226Asp	substitution	226	Ala	Asp		0.863	0.013
p.Ala226Cys	substitution	226	Ala	Cys		0.947	0.017

p.Ala226Gln	substitution	226	Ala	Gln	0.963	0.004
p.Ala226Glu	substitution	226	Ala	Glu	1.907	0.001
p.Ala226Gly	substitution	226	Ala	Gly	0.906	0.012
p.Ala226His	substitution	226	Ala	His	0.964	0.009
p.Ala226Ile	substitution	226	Ala	Ile	0.562	0.005
p.Ala226Leu	substitution	226	Ala	Leu	0.675	0.025
p.Ala226Lys	substitution	226	Ala	Lys	1.417	0.150
p.Ala226Met	substitution	226	Ala	Met	1.290	0.008
p.Ala226Phe	substitution	226	Ala	Phe	0.791	0.010
p.Ala226Pro	substitution	226	Ala	Pro	0.503	0.021
p.Ala226Ser	substitution	226	Ala	Ser	0.663	0.015
p.Ala226Ter	nonsense	226	Ala	Ter	0.028	0.018
p.Ala226Thr	substitution	226	Ala	Thr	0.707	0.049
p.Ala226Trp	substitution	226	Ala	Trp	1.563	0.001
p.Ala226Tyr	substitution	226	Ala	Tyr	0.996	0.057
p.Ala226Val	substitution	226	Ala	Val	0.982	0.020
p.Ala249=	synonymous	249	Ala	NA	1.317	0.018
p.Ala249Arg	substitution	249	Ala	Arg	0.000	0.023
p.Ala249Asn	substitution	249	Ala	Asn	0.000	0.075
p.Ala249Asp	substitution	249	Ala	Asp	0.140	0.014
p.Ala249Gln	substitution	249	Ala	Gln	0.000	0.039
p.Ala249Glu	substitution	249	Ala	Glu	0.030	0.064
p.Ala249Gly	substitution	249	Ala	Gly	0.000	0.010
p.Ala249His	substitution	249	Ala	His	0.000	0.027
p.Ala249Ile	substitution	249	Ala	Ile	0.000	0.075
p.Ala249Leu	substitution	249	Ala	Leu	0.199	0.043
p.Ala249Lys	substitution	249	Ala	Lys	0.166	0.019
p.Ala249Met	substitution	249	Ala	Met	0.000	0.041
p.Ala249Phe	substitution	249	Ala	Phe	0.000	0.018
p.Ala249Pro	substitution	249	Ala	Pro	0.191	0.084
p.Ala249Ser	substitution	249	Ala	Ser	1.076	0.030
p.Ala249Ter	nonsense	249	Ala	Ter	0.000	0.080
p.Ala249Thr	substitution	249	Ala	Thr	1.199	0.012
p.Ala249Trp	substitution	249	Ala	Trp	0.000	0.029
p.Ala249Tyr	substitution	249	Ala	Tyr	0.000	0.015
p.Ala249Val	substitution	249	Ala	Val	0.546	0.023
p.Ala252=	synonymous	252	Ala	NA	0.952	0.060
p.Ala252Arg	substitution	252	Ala	Arg	0.085	0.074
p.Ala252Asn	substitution	252	Ala	Asn	0.000	0.013
p.Ala252Asp	substitution	252	Ala	Asp	0.000	0.093
p.Ala252Cys	substitution	252	Ala	Cys	0.000	0.226
p.Ala252Glu	substitution	252	Ala	Glu	0.668	0.006
p.Ala252Gly	substitution	252	Ala	Gly	0.419	0.137

p.Ala252His	substitution	252	Ala	His	0.000	0.184
p.Ala252Leu	substitution	252	Ala	Leu	0.060	0.026
p.Ala252Phe	substitution	252	Ala	Phe	0.000	0.091
p.Ala252Pro	substitution	252	Ala	Pro	0.000	0.072
p.Ala252Ser	substitution	252	Ala	Ser	0.000	0.041
p.Ala252Ter	nonsense	252	Ala	Ter	1.334	0.304
p.Ala252Thr	substitution	252	Ala	Thr	1.196	0.105
p.Ala252Trp	substitution	252	Ala	Trp	0.000	0.097
p.Ala252Tyr	substitution	252	Ala	Tyr	0.000	0.037
p.Ala252Val	substitution	252	Ala	Val	1.218	0.026
p.Ala266=	synonymous	266	Ala	NA	0.844	0.045
p.Ala266Arg	substitution	266	Ala	Arg	0.118	0.011
p.Ala266Asp	substitution	266	Ala	Asp	0.701	0.026
p.Ala266Cys	substitution	266	Ala	Cys	0.000	0.129
p.Ala266Gln	substitution	266	Ala	Gln	0.000	0.102
p.Ala266Glu	substitution	266	Ala	Glu	0.222	0.056
p.Ala266Gly	substitution	266	Ala	Gly	0.175	0.077
p.Ala266Ile	substitution	266	Ala	Ile	0.131	0.214
p.Ala266Leu	substitution	266	Ala	Leu	0.050	0.040
p.Ala266Lys	substitution	266	Ala	Lys	0.627	0.008
p.Ala266Pro	substitution	266	Ala	Pro	0.000	0.119
p.Ala266Ser	substitution	266	Ala	Ser	0.000	0.192
p.Ala266Ter	nonsense	266	Ala	Ter	0.060	0.122
p.Ala266Thr	substitution	266	Ala	Thr	0.514	0.509
p.Ala266Val	substitution	266	Ala	Val	1.316	0.079
p.Ala270=	synonymous	270	Ala	NA	0.329	0.008
p.Ala270Arg	substitution	270	Ala	Arg	1.004	0.032
p.Ala270Asn	substitution	270	Ala	Asn	0.091	0.014
p.Ala270Asp	substitution	270	Ala	Asp	0.951	0.042
p.Ala270Gln	substitution	270	Ala	Gln	1.070	0.005
p.Ala270Glu	substitution	270	Ala	Glu	1.187	0.006
p.Ala270Gly	substitution	270	Ala	Gly	0.399	0.033
p.Ala270His	substitution	270	Ala	His	0.857	0.004
p.Ala270Ile	substitution	270	Ala	Ile	0.558	0.006
p.Ala270Leu	substitution	270	Ala	Leu	0.000	0.004
p.Ala270Lys	substitution	270	Ala	Lys	1.756	0.004
p.Ala270Met	substitution	270	Ala	Met	1.207	0.005
p.Ala270Phe	substitution	270	Ala	Phe	1.138	0.006
p.Ala270Pro	substitution	270	Ala	Pro	0.000	0.053
p.Ala270Ser	substitution	270	Ala	Ser	0.534	0.163
p.Ala270Ter	nonsense	270	Ala	Ter	0.436	0.034
p.Ala270Thr	substitution	270	Ala	Thr	0.516	0.015
p.Ala270Trp	substitution	270	Ala	Trp	0.519	0.008

p.Ala270Tyr	substitution	270	Ala	Tyr	0.386	0.008
p.Ala270Val	substitution	270	Ala	Val	0.965	0.006
p.Ala297=	synonymous	297	Ala	NA	1.039	0.018
p.Ala297Arg	substitution	297	Ala	Arg	0.000	0.055
p.Ala297Asn	substitution	297	Ala	Asn	0.000	0.079
p.Ala297Asp	substitution	297	Ala	Asp	0.844	0.114
p.Ala297Cys	substitution	297	Ala	Cys	1.461	0.006
p.Ala297Gln	substitution	297	Ala	Gln	0.245	0.013
p.Ala297Glu	substitution	297	Ala	Glu	0.272	0.013
p.Ala297Gly	substitution	297	Ala	Gly	0.815	0.124
p.Ala297Ile	substitution	297	Ala	Ile	0.000	0.125
p.Ala297Leu	substitution	297	Ala	Leu	0.299	0.134
p.Ala297Lys	substitution	297	Ala	Lys	0.000	0.012
p.Ala297Met	substitution	297	Ala	Met	0.000	0.221
p.Ala297Phe	substitution	297	Ala	Phe	0.000	0.040
p.Ala297Pro	substitution	297	Ala	Pro	0.000	0.235
p.Ala297Ser	substitution	297	Ala	Ser	0.395	0.076
p.Ala297Ter	nonsense	297	Ala	Ter	0.000	0.016
p.Ala297Thr	substitution	297	Ala	Thr	0.929	0.040
p.Ala297Trp	substitution	297	Ala	Trp	0.912	0.007
p.Ala297Tyr	substitution	297	Ala	Tyr	0.184	0.062
p.Ala297Val	substitution	297	Ala	Val	0.527	0.223
p.Ala303=	synonymous	303	Ala	NA	0.698	0.011
p.Ala303Arg	substitution	303	Ala	Arg	1.014	0.130
p.Ala303Asn	substitution	303	Ala	Asn	1.306	0.077
p.Ala303Asp	substitution	303	Ala	Asp	1.442	0.139
p.Ala303Gln	substitution	303	Ala	Gln	0.000	0.024
p.Ala303Glu	substitution	303	Ala	Glu	0.788	0.003
p.Ala303Gly	substitution	303	Ala	Gly	0.119	0.041
p.Ala303His	substitution	303	Ala	His	0.016	0.075
p.Ala303Ile	substitution	303	Ala	Ile	1.272	0.004
p.Ala303Leu	substitution	303	Ala	Leu	1.263	0.051
p.Ala303Lys	substitution	303	Ala	Lys	1.136	0.003
p.Ala303Met	substitution	303	Ala	Met	0.101	0.219
p.Ala303Phe	substitution	303	Ala	Phe	1.547	0.002
p.Ala303Pro	substitution	303	Ala	Pro	0.000	0.053
p.Ala303Ser	substitution	303	Ala	Ser	0.796	0.098
p.Ala303Ter	nonsense	303	Ala	Ter	0.732	0.034
p.Ala303Thr	substitution	303	Ala	Thr	0.961	0.021
p.Ala303Trp	substitution	303	Ala	Trp	0.000	0.022
p.Ala303Tyr	substitution	303	Ala	Tyr	0.418	0.048
p.Ala303Val	substitution	303	Ala	Val	0.245	0.053
p.Ala31=	synonymous	31	Ala	NA	0.634	0.011

p.Ala31Arg	substitution	31	Ala	Arg	0.000	0.006
p.Ala31Asn	substitution	31	Ala	Asn	0.000	0.008
p.Ala31Asp	substitution	31	Ala	Asp	0.000	0.003
p.Ala31Cys	substitution	31	Ala	Cys	0.000	0.002
p.Ala31Gln	substitution	31	Ala	Gln	0.000	0.009
p.Ala31Glu	substitution	31	Ala	Glu	0.000	0.001
p.Ala31Gly	substitution	31	Ala	Gly	0.664	0.000
p.Ala31His	substitution	31	Ala	His	0.000	0.005
p.Ala31Ile	substitution	31	Ala	Ile	0.000	0.003
p.Ala31Leu	substitution	31	Ala	Leu	0.000	0.008
p.Ala31Lys	substitution	31	Ala	Lys	0.000	0.006
p.Ala31Met	substitution	31	Ala	Met	0.000	0.021
p.Ala31Phe	substitution	31	Ala	Phe	0.000	0.003
p.Ala31Pro	substitution	31	Ala	Pro	0.000	0.013
p.Ala31Ser	substitution	31	Ala	Ser	0.846	0.012
p.Ala31Ter	nonsense	31	Ala	Ter	0.000	0.009
p.Ala31Thr	substitution	31	Ala	Thr	0.000	0.094
p.Ala31Trp	substitution	31	Ala	Trp	0.000	0.003
p.Ala31Tyr	substitution	31	Ala	Tyr	0.000	0.006
p.Ala31Val	substitution	31	Ala	Val	0.000	0.015
p.Ala320=	synonymous	320	Ala	NA	0.820	0.028
p.Ala320Arg	substitution	320	Ala	Arg	0.110	0.021
p.Ala320Asn	substitution	320	Ala	Asn	0.000	0.138
p.Ala320Asp	substitution	320	Ala	Asp	0.723	0.009
p.Ala320Cys	substitution	320	Ala	Cys	0.468	0.006
p.Ala320Gln	substitution	320	Ala	Gln	0.042	0.189
p.Ala320Glu	substitution	320	Ala	Glu	0.880	0.002
p.Ala320Gly	substitution	320	Ala	Gly	1.157	0.020
p.Ala320His	substitution	320	Ala	His	0.000	0.046
p.Ala320Ile	substitution	320	Ala	Ile	0.000	0.038
p.Ala320Leu	substitution	320	Ala	Leu	0.643	0.237
p.Ala320Lys	substitution	320	Ala	Lys	0.228	0.007
p.Ala320Met	substitution	320	Ala	Met	0.257	0.232
p.Ala320Phe	substitution	320	Ala	Phe	0.009	0.013
p.Ala320Pro	substitution	320	Ala	Pro	0.170	0.139
p.Ala320Ser	substitution	320	Ala	Ser	0.000	0.034
p.Ala320Ter	nonsense	320	Ala	Ter	0.000	0.222
p.Ala320Thr	substitution	320	Ala	Thr	0.962	0.137
p.Ala320Trp	substitution	320	Ala	Trp	1.118	0.012
p.Ala320Tyr	substitution	320	Ala	Tyr	0.000	0.054
p.Ala320Val	substitution	320	Ala	Val	0.829	0.003
p.Ala330=	synonymous	330	Ala	NA	0.484	0.095
p.Ala330Arg	substitution	330	Ala	Arg	1.237	0.001

p.Ala330Asn	substitution	330	Ala	Asn		1.052	0.004
p.Ala330Asp	substitution	330	Ala	Asp		0.931	0.006
p.Ala330Cys	substitution	330	Ala	Cys		1.699	0.006
p.Ala330Gly	substitution	330	Ala	Gly		0.742	0.405
p.Ala330His	substitution	330	Ala	His		0.023	0.076
p.Ala330Ile	substitution	330	Ala	Ile		0.000	0.106
p.Ala330Leu	substitution	330	Ala	Leu		1.325	0.129
p.Ala330Met	substitution	330	Ala	Met		0.000	0.089
p.Ala330Phe	substitution	330	Ala	Phe		0.544	0.028
p.Ala330Pro	substitution	330	Ala	Pro		0.459	0.049
p.Ala330Ser	substitution	330	Ala	Ser		0.614	0.146
p.Ala330Thr	substitution	330	Ala	Thr		0.794	0.156
p.Ala330Tyr	substitution	330	Ala	Tyr		0.000	0.090
p.Ala330Val	substitution	330	Ala	Val		1.198	0.009
p.Ala331=	synonymous	331	Ala	NA		0.810	0.806
p.Ala331Arg	substitution	331	Ala	Arg		0.000	0.148
p.Ala331Asn	substitution	331	Ala	Asn		0.000	0.121
p.Ala331Asp	substitution	331	Ala	Asp		0.548	0.221
p.Ala331Cys	substitution	331	Ala	Cys		1.009	0.008
p.Ala331Glu	substitution	331	Ala	Glu		0.125	0.015
p.Ala331Gly	substitution	331	Ala	Gly		1.589	0.047
p.Ala331Leu	substitution	331	Ala	Leu		0.000	0.028
p.Ala331Met	substitution	331	Ala	Met		0.099	0.012
p.Ala331Pro	substitution	331	Ala	Pro		0.000	0.019
p.Ala331Ser	substitution	331	Ala	Ser		0.199	0.068
p.Ala331Ter	nonsense	331	Ala	Ter		0.177	0.014
p.Ala331Thr	substitution	331	Ala	Thr		0.686	0.116
p.Ala331Val	substitution	331	Ala	Val		0.265	0.040
p.Ala339=	synonymous	339	Ala	NA		0.913	0.013
p.Ala339Arg	substitution	339	Ala	Arg		0.128	0.092
p.Ala339Asp	substitution	339	Ala	Asp		0.489	0.033
p.Ala339Cys	substitution	339	Ala	Cys		0.000	0.018
p.Ala339Glu	substitution	339	Ala	Glu		0.000	0.111
p.Ala339Gly	substitution	339	Ala	Gly		1.296	0.025
p.Ala339Ile	substitution	339	Ala	Ile		0.000	0.035
p.Ala339Met	substitution	339	Ala	Met		0.000	0.186
p.Ala339Pro	substitution	339	Ala	Pro		0.000	0.188
p.Ala339Ser	substitution	339	Ala	Ser		0.523	0.585
p.Ala339Ter	nonsense	339	Ala	Ter		0.000	0.257
p.Ala339Thr	substitution	339	Ala	Thr		1.049	0.011
p.Ala339Tyr	substitution	339	Ala	Tyr		0.183	0.200
p.Ala339Val	substitution	339	Ala	Val		0.839	0.074
p.Ala347=	synonymous	347	Ala	NA		1.194	0.018

p.Ala347Arg	substitution	347	Ala	Arg	1.450	0.021
p.Ala347Asp	substitution	347	Ala	Asp	0.000	0.128
p.Ala347Cys	substitution	347	Ala	Cys	0.132	0.143
p.Ala347Gln	substitution	347	Ala	Gln	1.076	0.046
p.Ala347Gly	substitution	347	Ala	Gly	0.000	0.078
p.Ala347His	substitution	347	Ala	His	0.255	0.099
p.Ala347Ile	substitution	347	Ala	Ile	0.011	0.258
p.Ala347Lys	substitution	347	Ala	Lys	0.000	0.268
p.Ala347Met	substitution	347	Ala	Met	0.374	0.053
p.Ala347Phe	substitution	347	Ala	Phe	0.023	0.073
p.Ala347Pro	substitution	347	Ala	Pro	1.638	0.117
p.Ala347Ser	substitution	347	Ala	Ser	0.918	0.080
p.Ala347Thr	substitution	347	Ala	Thr	1.147	0.063
p.Ala347Trp	substitution	347	Ala	Trp	0.000	0.058
p.Ala347Tyr	substitution	347	Ala	Tyr	1.499	0.010
p.Ala347Val	substitution	347	Ala	Val	0.013	0.030
p.Ala354=	synonymous	354	Ala	NA	1.566	0.245
p.Ala354Arg	substitution	354	Ala	Arg	0.034	0.267
p.Ala354Asn	substitution	354	Ala	Asn	0.000	0.199
p.Ala354Asp	substitution	354	Ala	Asp	1.285	0.006
p.Ala354Glu	substitution	354	Ala	Glu	1.001	0.080
p.Ala354Gly	substitution	354	Ala	Gly	1.246	0.389
p.Ala354Ile	substitution	354	Ala	Ile	0.000	0.127
p.Ala354Leu	substitution	354	Ala	Leu	0.121	0.028
p.Ala354Met	substitution	354	Ala	Met	1.060	0.006
p.Ala354Pro	substitution	354	Ala	Pro	1.173	0.018
p.Ala354Ser	substitution	354	Ala	Ser	0.203	0.054
p.Ala354Thr	substitution	354	Ala	Thr	1.096	0.102
p.Ala354Tyr	substitution	354	Ala	Tyr	0.000	0.078
p.Ala354Val	substitution	354	Ala	Val	0.313	0.060
p.Ala360=	synonymous	360	Ala	NA	1.173	0.032
p.Ala360Arg	substitution	360	Ala	Arg	0.380	0.051
p.Ala360Asn	substitution	360	Ala	Asn	0.000	0.072
p.Ala360Asp	substitution	360	Ala	Asp	0.930	0.015
p.Ala360Cys	substitution	360	Ala	Cys	0.000	0.219
p.Ala360Gln	substitution	360	Ala	Gln	0.000	0.026
p.Ala360Gly	substitution	360	Ala	Gly	1.113	0.085
p.Ala360Ile	substitution	360	Ala	Ile	0.266	0.154
p.Ala360Leu	substitution	360	Ala	Leu	0.534	0.083
p.Ala360Lys	substitution	360	Ala	Lys	0.000	0.021
p.Ala360Met	substitution	360	Ala	Met	0.000	0.053
p.Ala360Pro	substitution	360	Ala	Pro	0.000	0.123
p.Ala360Ser	substitution	360	Ala	Ser	0.910	0.022

p.Ala360Thr	substitution	360	Ala	Thr	1.064	0.154
p.Ala360Trp	substitution	360	Ala	Trp	0.000	0.027
p.Ala360Val	substitution	360	Ala	Val	1.311	0.009
p.Ala40=	synonymous	40	Ala	NA	0.959	0.019
p.Ala40Arg	substitution	40	Ala	Arg	0.586	0.022
p.Ala40Asn	substitution	40	Ala	Asn	0.558	0.008
p.Ala40Asp	substitution	40	Ala	Asp	0.891	0.007
p.Ala40Cys	substitution	40	Ala	Cys	1.208	0.004
p.Ala40Gln	substitution	40	Ala	Gln	0.591	0.125
p.Ala40Glu	substitution	40	Ala	Glu	0.800	0.007
p.Ala40Gly	substitution	40	Ala	Gly	0.944	0.002
p.Ala40His	substitution	40	Ala	His	0.658	0.006
p.Ala40Ile	substitution	40	Ala	Ile	0.615	0.010
p.Ala40Leu	substitution	40	Ala	Leu	0.728	0.011
p.Ala40Lys	substitution	40	Ala	Lys	1.049	0.012
p.Ala40Met	substitution	40	Ala	Met	1.085	0.006
p.Ala40Phe	substitution	40	Ala	Phe	0.554	0.007
p.Ala40Pro	substitution	40	Ala	Pro	0.061	0.077
p.Ala40Ser	substitution	40	Ala	Ser	0.775	0.017
p.Ala40Ter	nonsense	40	Ala	Ter	0.248	0.077
p.Ala40Thr	substitution	40	Ala	Thr	0.945	0.030
p.Ala40Trp	substitution	40	Ala	Trp	0.820	0.006
p.Ala40Tyr	substitution	40	Ala	Tyr	1.097	0.004
p.Ala40Val	substitution	40	Ala	Val	0.967	0.021
p.Ala44=	synonymous	44	Ala	NA	0.977	0.002
p.Ala44Arg	substitution	44	Ala	Arg	1.006	0.008
p.Ala44Asn	substitution	44	Ala	Asn	1.035	0.013
p.Ala44Asp	substitution	44	Ala	Asp	0.726	0.005
p.Ala44Cys	substitution	44	Ala	Cys	0.333	0.031
p.Ala44Gln	substitution	44	Ala	Gln	0.789	0.005
p.Ala44Glu	substitution	44	Ala	Glu	0.987	0.009
p.Ala44Gly	substitution	44	Ala	Gly	0.904	0.009
p.Ala44His	substitution	44	Ala	His	0.767	0.018
p.Ala44Ile	substitution	44	Ala	Ile	0.754	0.163
p.Ala44Leu	substitution	44	Ala	Leu	0.792	0.012
p.Ala44Lys	substitution	44	Ala	Lys	1.073	0.004
p.Ala44Met	substitution	44	Ala	Met	0.110	0.010
p.Ala44Phe	substitution	44	Ala	Phe	0.980	0.015
p.Ala44Pro	substitution	44	Ala	Pro	0.464	0.014
p.Ala44Ser	substitution	44	Ala	Ser	0.934	0.007
p.Ala44Ter	nonsense	44	Ala	Ter	0.000	0.086
p.Ala44Thr	substitution	44	Ala	Thr	0.964	0.015
p.Ala44Trp	substitution	44	Ala	Trp	0.796	0.002

p.Ala44Tyr	substitution	44	Ala	Tyr		1.015	0.014
p.Ala44Val	substitution	44	Ala	Val		1.059	0.022
p.Ala55=	synonymous	55	Ala	NA		1.114	0.020
p.Ala55Arg	substitution	55	Ala	Arg		0.807	0.024
p.Ala55Asn	substitution	55	Ala	Asn		0.595	0.008
p.Ala55Asp	substitution	55	Ala	Asp		0.884	0.003
p.Ala55Cys	substitution	55	Ala	Cys		0.630	0.009
p.Ala55Gln	substitution	55	Ala	Gln		0.819	0.017
p.Ala55Glu	substitution	55	Ala	Glu		1.611	0.004
p.Ala55Gly	substitution	55	Ala	Gly		0.686	0.003
p.Ala55His	substitution	55	Ala	His		0.965	0.008
p.Ala55Ile	substitution	55	Ala	Ile		0.670	0.005
p.Ala55Leu	substitution	55	Ala	Leu		0.823	0.004
p.Ala55Lys	substitution	55	Ala	Lys		0.584	0.007
p.Ala55Met	substitution	55	Ala	Met		0.578	0.009
p.Ala55Phe	substitution	55	Ala	Phe		1.213	0.007
p.Ala55Pro	substitution	55	Ala	Pro		0.896	0.018
p.Ala55Ser	substitution	55	Ala	Ser		0.966	0.009
p.Ala55Ter	nonsense	55	Ala	Ter		0.053	0.017
p.Ala55Thr	substitution	55	Ala	Thr		0.683	0.046
p.Ala55Trp	substitution	55	Ala	Trp		0.680	0.010
p.Ala55Tyr	substitution	55	Ala	Tyr		0.892	0.006
p.Ala55Val	substitution	55	Ala	Val		0.939	0.010
p.Ala67=	synonymous	67	Ala	NA		1.199	0.972
p.Ala67Arg	substitution	67	Ala	Arg		1.126	0.121
p.Ala67Asn	substitution	67	Ala	Asn		0.230	0.090
p.Ala67Asp	substitution	67	Ala	Asp		1.857	0.037
p.Ala67Gln	substitution	67	Ala	Gln		0.665	0.068
p.Ala67Glu	substitution	67	Ala	Glu		0.000	0.374
p.Ala67Gly	substitution	67	Ala	Gly		0.759	0.047
p.Ala67His	substitution	67	Ala	His		0.109	0.390
p.Ala67Ile	substitution	67	Ala	Ile		1.271	0.013
p.Ala67Leu	substitution	67	Ala	Leu		0.708	0.195
p.Ala67Lys	substitution	67	Ala	Lys		1.575	0.029
p.Ala67Met	substitution	67	Ala	Met		0.000	0.058
p.Ala67Phe	substitution	67	Ala	Phe		0.000	0.160
p.Ala67Pro	substitution	67	Ala	Pro		0.100	0.374
p.Ala67Ser	substitution	67	Ala	Ser		0.000	0.091
p.Ala67Ter	nonsense	67	Ala	Ter		0.000	0.307
p.Ala67Thr	substitution	67	Ala	Thr		1.139	0.022
p.Ala67Trp	substitution	67	Ala	Trp		0.000	0.042
p.Ala67Val	substitution	67	Ala	Val		0.845	0.094
p.Ala84=	synonymous	84	Ala	NA		0.816	0.007

p.Ala84Arg	substitution	84	Ala	Arg		0.377	0.023
p.Ala84Asn	substitution	84	Ala	Asn		0.557	0.015
p.Ala84Asp	substitution	84	Ala	Asp		0.637	0.007
p.Ala84Cys	substitution	84	Ala	Cys		0.596	0.018
p.Ala84Gln	substitution	84	Ala	Gln		0.445	0.012
p.Ala84Glu	substitution	84	Ala	Glu		0.505	0.007
p.Ala84Gly	substitution	84	Ala	Gly		0.565	0.019
p.Ala84His	substitution	84	Ala	His		0.680	0.035
p.Ala84Ile	substitution	84	Ala	Ile		0.354	0.084
p.Ala84Leu	substitution	84	Ala	Leu		0.507	0.071
p.Ala84Lys	substitution	84	Ala	Lys		0.677	0.016
p.Ala84Met	substitution	84	Ala	Met		1.114	0.024
p.Ala84Phe	substitution	84	Ala	Phe		0.904	0.028
p.Ala84Pro	substitution	84	Ala	Pro		0.445	0.100
p.Ala84Ser	substitution	84	Ala	Ser		0.763	0.051
p.Ala84Ter	nonsense	84	Ala	Ter		0.055	0.077
p.Ala84Thr	substitution	84	Ala	Thr		0.895	0.038
p.Ala84Trp	substitution	84	Ala	Trp		0.410	0.021
p.Ala84Tyr	substitution	84	Ala	Tyr		0.680	0.590
p.Ala84Val	substitution	84	Ala	Val		0.597	0.017
p.Arg116=	synonymous	116	Arg	NA		1.080	0.020
p.Arg116Ala	substitution	116	Arg	Ala		0.624	0.004
p.Arg116Asn	substitution	116	Arg	Asn		0.125	0.008
p.Arg116Asp	substitution	116	Arg	Asp		0.180	0.006
p.Arg116Cys	substitution	116	Arg	Cys		0.524	0.004
p.Arg116Gln	substitution	116	Arg	Gln		0.264	0.016
p.Arg116Glu	substitution	116	Arg	Glu		0.315	0.010
p.Arg116Gly	substitution	116	Arg	Gly		0.470	0.014
p.Arg116His	substitution	116	Arg	His		0.146	0.010
p.Arg116Ile	substitution	116	Arg	Ile		0.000	0.006
p.Arg116Leu	substitution	116	Arg	Leu		0.107	0.005
p.Arg116Lys	substitution	116	Arg	Lys		0.059	0.005
p.Arg116Met	substitution	116	Arg	Met		0.032	0.009
p.Arg116Pro	substitution	116	Arg	Pro		0.163	0.027
p.Arg116Ser	substitution	116	Arg	Ser		0.611	0.020
p.Arg116Ter	nonsense	116	Arg	Ter		0.103	0.041
p.Arg116Thr	substitution	116	Arg	Thr		0.203	0.004
p.Arg116Trp	substitution	116	Arg	Trp		0.184	0.005
p.Arg116Tyr	substitution	116	Arg	Tyr		0.169	0.006
p.Arg116Val	substitution	116	Arg	Val		0.000	0.007
p.Arg149=	synonymous	149	Arg	NA		0.967	0.021
p.Arg149Ala	substitution	149	Arg	Ala		0.000	0.030
p.Arg149Asn	substitution	149	Arg	Asn		0.000	0.157

p.Arg149Asp	substitution	149	Arg	Asp	0.150	0.021
p.Arg149Cys	substitution	149	Arg	Cys	0.018	0.020
p.Arg149Gln	substitution	149	Arg	Gln	0.004	0.014
p.Arg149Glu	substitution	149	Arg	Glu	0.000	0.302
p.Arg149Gly	substitution	149	Arg	Gly	0.000	0.036
p.Arg149His	substitution	149	Arg	His	0.433	0.010
p.Arg149Ile	substitution	149	Arg	Ile	0.149	0.180
p.Arg149Leu	substitution	149	Arg	Leu	0.000	0.031
p.Arg149Lys	substitution	149	Arg	Lys	0.029	0.018
p.Arg149Met	substitution	149	Arg	Met	0.000	0.140
p.Arg149Phe	substitution	149	Arg	Phe	0.501	0.136
p.Arg149Pro	substitution	149	Arg	Pro	0.000	0.028
p.Arg149Ser	substitution	149	Arg	Ser	0.000	0.014
p.Arg149Ter	nonsense	149	Arg	Ter	0.000	0.104
p.Arg149Thr	substitution	149	Arg	Thr	0.000	0.031
p.Arg149Trp	substitution	149	Arg	Trp	0.047	0.013
p.Arg149Tyr	substitution	149	Arg	Tyr	0.565	0.236
p.Arg149Val	substitution	149	Arg	Val	0.000	0.136
p.Arg150=	synonymous	150	Arg	NA	0.849	0.012
p.Arg150Asn	substitution	150	Arg	Asn	0.000	0.542
p.Arg150Cys	substitution	150	Arg	Cys	0.437	0.011
p.Arg150Gln	substitution	150	Arg	Gln	0.000	0.105
p.Arg150Glu	substitution	150	Arg	Glu	0.000	0.016
p.Arg150Gly	substitution	150	Arg	Gly	0.163	0.067
p.Arg150His	substitution	150	Arg	His	0.266	0.028
p.Arg150Ile	substitution	150	Arg	Ile	0.000	0.045
p.Arg150Leu	substitution	150	Arg	Leu	0.000	0.028
p.Arg150Lys	substitution	150	Arg	Lys	0.000	0.024
p.Arg150Met	substitution	150	Arg	Met	0.374	0.014
p.Arg150Phe	substitution	150	Arg	Phe	0.000	0.034
p.Arg150Pro	substitution	150	Arg	Pro	0.073	0.137
p.Arg150Ser	substitution	150	Arg	Ser	0.090	0.043
p.Arg150Ter	nonsense	150	Arg	Ter	0.032	0.185
p.Arg150Thr	substitution	150	Arg	Thr	0.000	0.201
p.Arg150Trp	substitution	150	Arg	Trp	0.093	0.017
p.Arg150Tyr	substitution	150	Arg	Tyr	0.000	0.014
p.Arg150Val	substitution	150	Arg	Val	0.398	0.290
p.Arg156=	synonymous	156	Arg	NA	1.009	0.016
p.Arg156Ala	substitution	156	Arg	Ala	1.087	0.007
p.Arg156Asn	substitution	156	Arg	Asn	0.933	0.017
p.Arg156Asp	substitution	156	Arg	Asp	0.556	0.025
p.Arg156Cys	substitution	156	Arg	Cys	0.724	0.006
p.Arg156Gln	substitution	156	Arg	Gln	0.918	0.018

p.Arg156Glu	substitution	156	Arg	Glu	0.861	0.008
p.Arg156Gly	substitution	156	Arg	Gly	0.614	0.008
p.Arg156His	substitution	156	Arg	His	1.067	0.006
p.Arg156Ile	substitution	156	Arg	Ile	1.131	0.011
p.Arg156Leu	substitution	156	Arg	Leu	0.763	0.017
p.Arg156Lys	substitution	156	Arg	Lys	0.968	0.053
p.Arg156Met	substitution	156	Arg	Met	1.122	0.008
p.Arg156Phe	substitution	156	Arg	Phe	0.257	0.008
p.Arg156Pro	substitution	156	Arg	Pro	0.996	0.030
p.Arg156Ser	substitution	156	Arg	Ser	1.048	0.019
p.Arg156Ter	nonsense	156	Arg	Ter	0.026	0.037
p.Arg156Thr	substitution	156	Arg	Thr	0.977	0.024
p.Arg156Trp	substitution	156	Arg	Trp	0.901	0.005
p.Arg156Tyr	substitution	156	Arg	Tyr	0.372	0.013
p.Arg156Val	substitution	156	Arg	Val	0.961	0.007
p.Arg164=	synonymous	164	Arg	NA	1.118	0.010
p.Arg164Ala	substitution	164	Arg	Ala	0.902	0.017
p.Arg164Asn	substitution	164	Arg	Asn	0.881	0.004
p.Arg164Asp	substitution	164	Arg	Asp	0.968	0.004
p.Arg164Cys	substitution	164	Arg	Cys	0.821	0.004
p.Arg164Gln	substitution	164	Arg	Gln	0.697	0.005
p.Arg164Glu	substitution	164	Arg	Glu	1.129	0.004
p.Arg164Gly	substitution	164	Arg	Gly	0.832	0.004
p.Arg164His	substitution	164	Arg	His	1.078	0.006
p.Arg164Ile	substitution	164	Arg	Ile	0.747	0.006
p.Arg164Leu	substitution	164	Arg	Leu	0.839	0.005
p.Arg164Lys	substitution	164	Arg	Lys	0.985	0.005
p.Arg164Met	substitution	164	Arg	Met	0.921	0.011
p.Arg164Phe	substitution	164	Arg	Phe	0.702	0.004
p.Arg164Pro	substitution	164	Arg	Pro	0.531	0.025
p.Arg164Ser	substitution	164	Arg	Ser	0.889	0.006
p.Arg164Ter	nonsense	164	Arg	Ter	0.000	0.015
p.Arg164Thr	substitution	164	Arg	Thr	0.954	0.005
p.Arg164Trp	substitution	164	Arg	Trp	0.901	0.005
p.Arg164Tyr	substitution	164	Arg	Tyr	0.542	0.006
p.Arg164Val	substitution	164	Arg	Val	0.811	0.007
p.Arg167=	synonymous	167	Arg	NA	1.064	0.024
p.Arg167Ala	substitution	167	Arg	Ala	0.800	0.005
p.Arg167Asn	substitution	167	Arg	Asn	0.625	0.008
p.Arg167Asp	substitution	167	Arg	Asp	0.286	0.008
p.Arg167Cys	substitution	167	Arg	Cys	0.223	0.006
p.Arg167Gln	substitution	167	Arg	Gln	0.733	0.016
p.Arg167Glu	substitution	167	Arg	Glu	0.000	0.009

p.Arg167Gly	substitution	167	Arg	Gly	0.771	0.045
p.Arg167His	substitution	167	Arg	His	0.543	0.008
p.Arg167Ile	substitution	167	Arg	Ile	0.683	0.007
p.Arg167Leu	substitution	167	Arg	Leu	0.702	0.007
p.Arg167Lys	substitution	167	Arg	Lys	0.448	0.013
p.Arg167Met	substitution	167	Arg	Met	0.532	0.006
p.Arg167Phe	substitution	167	Arg	Phe	0.325	0.008
p.Arg167Pro	substitution	167	Arg	Pro	0.136	0.006
p.Arg167Ser	substitution	167	Arg	Ser	0.662	0.011
p.Arg167Ter	nonsense	167	Arg	Ter	0.122	0.008
p.Arg167Thr	substitution	167	Arg	Thr	0.919	0.006
p.Arg167Trp	substitution	167	Arg	Trp	0.522	0.088
p.Arg167Tyr	substitution	167	Arg	Tyr	0.700	0.003
p.Arg167Val	substitution	167	Arg	Val	0.693	0.008
p.Arg173=	synonymous	173	Arg	NA	0.983	0.109
p.Arg173Ala	substitution	173	Arg	Ala	0.000	0.013
p.Arg173Asn	substitution	173	Arg	Asn	0.048	0.009
p.Arg173Asp	substitution	173	Arg	Asp	0.000	0.046
p.Arg173Cys	substitution	173	Arg	Cys	0.000	0.011
p.Arg173Gln	substitution	173	Arg	Gln	0.000	0.300
p.Arg173Gly	substitution	173	Arg	Gly	0.000	0.008
p.Arg173His	substitution	173	Arg	His	0.063	0.012
p.Arg173Ile	substitution	173	Arg	Ile	0.000	0.024
p.Arg173Leu	substitution	173	Arg	Leu	0.046	0.024
p.Arg173Lys	substitution	173	Arg	Lys	0.152	0.013
p.Arg173Met	substitution	173	Arg	Met	0.000	0.010
p.Arg173Phe	substitution	173	Arg	Phe	0.000	0.012
p.Arg173Pro	substitution	173	Arg	Pro	0.000	0.022
p.Arg173Ser	substitution	173	Arg	Ser	0.000	0.020
p.Arg173Ter	nonsense	173	Arg	Ter	0.000	0.039
p.Arg173Thr	substitution	173	Arg	Thr	0.000	0.030
p.Arg173Trp	substitution	173	Arg	Trp	0.000	0.100
p.Arg173Tyr	substitution	173	Arg	Tyr	0.000	0.029
p.Arg173Val	substitution	173	Arg	Val	0.000	0.011
p.Arg175=	synonymous	175	Arg	NA	0.883	0.011
p.Arg175Ala	substitution	175	Arg	Ala	0.882	0.021
p.Arg175Asn	substitution	175	Arg	Asn	0.872	0.014
p.Arg175Asp	substitution	175	Arg	Asp	0.509	0.045
p.Arg175Cys	substitution	175	Arg	Cys	0.909	0.012
p.Arg175Gln	substitution	175	Arg	Gln	1.048	0.015
p.Arg175Glu	substitution	175	Arg	Glu	0.378	0.008
p.Arg175Gly	substitution	175	Arg	Gly	0.793	0.029
p.Arg175His	substitution	175	Arg	His	0.342	0.021

p.Arg175Leu	substitution	175	Arg	Leu	0.756	0.010
p.Arg175Lys	substitution	175	Arg	Lys	1.012	0.009
p.Arg175Met	substitution	175	Arg	Met	1.032	0.006
p.Arg175Phe	substitution	175	Arg	Phe	0.077	0.013
p.Arg175Pro	substitution	175	Arg	Pro	0.160	0.106
p.Arg175Ser	substitution	175	Arg	Ser	0.875	0.070
p.Arg175Ter	nonsense	175	Arg	Ter	0.092	0.008
p.Arg175Thr	substitution	175	Arg	Thr	0.688	0.045
p.Arg175Trp	substitution	175	Arg	Trp	0.897	0.025
p.Arg175Tyr	substitution	175	Arg	Tyr	0.429	0.030
p.Arg175Val	substitution	175	Arg	Val	1.018	0.032
p.Arg195=	synonymous	195	Arg	NA	1.065	0.032
p.Arg195Ala	substitution	195	Arg	Ala	0.100	0.018
p.Arg195Asn	substitution	195	Arg	Asn	0.000	0.016
p.Arg195Asp	substitution	195	Arg	Asp	0.000	0.246
p.Arg195Cys	substitution	195	Arg	Cys	0.248	0.093
p.Arg195Gln	substitution	195	Arg	Gln	0.152	0.007
p.Arg195Glu	substitution	195	Arg	Glu	0.112	0.018
p.Arg195Gly	substitution	195	Arg	Gly	0.122	0.043
p.Arg195His	substitution	195	Arg	His	0.783	0.058
p.Arg195Ile	substitution	195	Arg	Ile	1.280	0.149
p.Arg195Leu	substitution	195	Arg	Leu	0.868	0.043
p.Arg195Lys	substitution	195	Arg	Lys	0.005	0.029
p.Arg195Met	substitution	195	Arg	Met	0.000	0.036
p.Arg195Phe	substitution	195	Arg	Phe	0.197	0.059
p.Arg195Pro	substitution	195	Arg	Pro	0.117	0.022
p.Arg195Ser	substitution	195	Arg	Ser	0.000	0.105
p.Arg195Ter	nonsense	195	Arg	Ter	0.000	0.013
p.Arg195Thr	substitution	195	Arg	Thr	0.000	0.019
p.Arg195Trp	substitution	195	Arg	Trp	0.328	0.009
p.Arg195Tyr	substitution	195	Arg	Tyr	0.805	0.013
p.Arg195Val	substitution	195	Arg	Val	0.151	0.177
p.Arg19=	synonymous	19	Arg	NA	0.687	0.000
p.Arg19Cys	substitution	19	Arg	Cys	0.222	0.363
p.Arg19Gly	substitution	19	Arg	Gly	1.024	0.066
p.Arg19Ile	substitution	19	Arg	Ile	0.949	0.005
p.Arg19Lys	substitution	19	Arg	Lys	1.015	0.008
p.Arg19Ser	substitution	19	Arg	Ser	0.631	0.000
p.Arg19Thr	substitution	19	Arg	Thr	0.810	0.056
p.Arg201=	synonymous	201	Arg	NA	1.087	0.014
p.Arg201Ala	substitution	201	Arg	Ala	0.000	0.006
p.Arg201Asn	substitution	201	Arg	Asn	0.520	0.010
p.Arg201Asp	substitution	201	Arg	Asp	0.952	0.005

p.Arg201Cys	substitution	201	Arg	Cys		0.540	0.005
p.Arg201Gln	substitution	201	Arg	Gln		0.724	0.015
p.Arg201Glu	substitution	201	Arg	Glu		0.000	0.023
p.Arg201Gly	substitution	201	Arg	Gly		0.673	0.031
p.Arg201His	substitution	201	Arg	His		0.000	0.027
p.Arg201Ile	substitution	201	Arg	Ile		1.755	0.012
p.Arg201Leu	substitution	201	Arg	Leu		1.092	0.007
p.Arg201Lys	substitution	201	Arg	Lys		0.755	0.004
p.Arg201Met	substitution	201	Arg	Met		0.000	0.136
p.Arg201Phe	substitution	201	Arg	Phe		0.231	0.007
p.Arg201Pro	substitution	201	Arg	Pro		0.205	0.044
p.Arg201Ser	substitution	201	Arg	Ser		0.000	0.110
p.Arg201Ter	nonsense	201	Arg	Ter		0.043	0.041
p.Arg201Thr	substitution	201	Arg	Thr		0.473	0.023
p.Arg201Trp	substitution	201	Arg	Trp		0.719	0.004
p.Arg201Tyr	substitution	201	Arg	Tyr		1.345	0.003
p.Arg201Val	substitution	201	Arg	Val		0.798	0.018
p.Arg225=	synonymous	225	Arg	NA		0.935	0.020
p.Arg225Ala	substitution	225	Arg	Ala		0.028	0.048
p.Arg225Asn	substitution	225	Arg	Asn		0.096	0.004
p.Arg225Asp	substitution	225	Arg	Asp		0.496	0.002
p.Arg225Cys	substitution	225	Arg	Cys		0.859	0.001
p.Arg225Gln	substitution	225	Arg	Gln		0.000	0.137
p.Arg225Glu	substitution	225	Arg	Glu		0.607	0.007
p.Arg225Gly	substitution	225	Arg	Gly		0.000	0.093
p.Arg225Ile	substitution	225	Arg	Ile		0.245	0.159
p.Arg225Leu	substitution	225	Arg	Leu		0.758	0.009
p.Arg225Lys	substitution	225	Arg	Lys		0.133	0.038
p.Arg225Met	substitution	225	Arg	Met		0.985	0.001
p.Arg225Phe	substitution	225	Arg	Phe		0.311	0.003
p.Arg225Pro	substitution	225	Arg	Pro		1.063	0.030
p.Arg225Ser	substitution	225	Arg	Ser		0.633	0.032
p.Arg225Ter	nonsense	225	Arg	Ter		0.000	0.104
p.Arg225Thr	substitution	225	Arg	Thr		0.541	0.015
p.Arg225Tyr	substitution	225	Arg	Tyr		0.712	0.002
p.Arg225Val	substitution	225	Arg	Val		0.577	0.017
p.Arg22=	synonymous	22	Arg	NA		1.200	0.012
p.Arg22Ala	substitution	22	Arg	Ala		0.836	0.009
p.Arg22Asn	substitution	22	Arg	Asn		0.830	0.008
p.Arg22Asp	substitution	22	Arg	Asp		0.297	0.002
p.Arg22Cys	substitution	22	Arg	Cys		0.933	0.009
p.Arg22Gln	substitution	22	Arg	Gln		0.839	0.001
p.Arg22Glu	substitution	22	Arg	Glu		0.786	0.002

p.Arg22Gly	substitution	22	Arg	Gly		0.631	0.003
p.Arg22His	substitution	22	Arg	His		1.713	0.021
p.Arg22Ile	substitution	22	Arg	Ile		1.159	0.003
p.Arg22Leu	substitution	22	Arg	Leu		1.161	0.001
p.Arg22Lys	substitution	22	Arg	Lys		1.006	0.002
p.Arg22Met	substitution	22	Arg	Met		0.239	0.003
p.Arg22Phe	substitution	22	Arg	Phe		0.783	0.002
p.Arg22Pro	substitution	22	Arg	Pro		0.379	0.002
p.Arg22Ser	substitution	22	Arg	Ser		0.914	0.002
p.Arg22Ter	nonsense	22	Arg	Ter		0.000	0.023
p.Arg22Thr	substitution	22	Arg	Thr		0.872	0.013
p.Arg22Trp	substitution	22	Arg	Trp		1.210	0.001
p.Arg22Tyr	substitution	22	Arg	Tyr		0.711	0.005
p.Arg22Val	substitution	22	Arg	Val		1.123	0.007
p.Arg246=	synonymous	246	Arg	NA		1.054	0.006
p.Arg246Ala	substitution	246	Arg	Ala		1.189	0.054
p.Arg246Asn	substitution	246	Arg	Asn		0.606	0.018
p.Arg246Asp	substitution	246	Arg	Asp		0.000	0.017
p.Arg246Cys	substitution	246	Arg	Cys		0.281	0.032
p.Arg246Gln	substitution	246	Arg	Gln		1.152	0.001
p.Arg246Glu	substitution	246	Arg	Glu		0.000	0.104
p.Arg246Gly	substitution	246	Arg	Gly		0.804	0.152
p.Arg246His	substitution	246	Arg	His		0.316	0.012
p.Arg246Ile	substitution	246	Arg	Ile		0.428	0.029
p.Arg246Leu	substitution	246	Arg	Leu		0.799	0.030
p.Arg246Lys	substitution	246	Arg	Lys		0.405	0.009
p.Arg246Met	substitution	246	Arg	Met		0.718	0.001
p.Arg246Phe	substitution	246	Arg	Phe		0.240	0.068
p.Arg246Pro	substitution	246	Arg	Pro		0.283	0.049
p.Arg246Ser	substitution	246	Arg	Ser		0.998	0.047
p.Arg246Ter	nonsense	246	Arg	Ter		0.000	0.038
p.Arg246Thr	substitution	246	Arg	Thr		0.647	0.004
p.Arg246Trp	substitution	246	Arg	Trp		0.387	0.004
p.Arg246Tyr	substitution	246	Arg	Tyr		0.388	0.013
p.Arg246Val	substitution	246	Arg	Val		1.016	0.124
p.Arg251=	synonymous	251	Arg	NA		0.902	0.039
p.Arg251Ala	substitution	251	Arg	Ala		0.000	0.217
p.Arg251Asn	substitution	251	Arg	Asn		0.332	0.365
p.Arg251Asp	substitution	251	Arg	Asp		0.000	0.013
p.Arg251Cys	substitution	251	Arg	Cys		0.176	0.238
p.Arg251Gln	substitution	251	Arg	Gln		0.000	0.119
p.Arg251Glu	substitution	251	Arg	Glu		0.072	0.020
p.Arg251Gly	substitution	251	Arg	Gly		0.000	0.029

p.Arg251His	substitution	251	Arg	His	0.000	0.044
p.Arg251Ile	substitution	251	Arg	Ile	0.053	0.022
p.Arg251Leu	substitution	251	Arg	Leu	0.000	0.008
p.Arg251Lys	substitution	251	Arg	Lys	0.953	0.002
p.Arg251Met	substitution	251	Arg	Met	0.835	0.006
p.Arg251Phe	substitution	251	Arg	Phe	0.032	0.150
p.Arg251Pro	substitution	251	Arg	Pro	0.363	0.028
p.Arg251Ser	substitution	251	Arg	Ser	0.418	0.090
p.Arg251Ter	nonsense	251	Arg	Ter	0.000	0.011
p.Arg251Thr	substitution	251	Arg	Thr	0.423	0.082
p.Arg251Trp	substitution	251	Arg	Trp	0.827	0.005
p.Arg251Tyr	substitution	251	Arg	Tyr	0.000	0.007
p.Arg251Val	substitution	251	Arg	Val	0.641	0.252
p.Arg255=	synonymous	255	Arg	NA	0.549	0.066
p.Arg255Ala	substitution	255	Arg	Ala	0.311	0.099
p.Arg255Asn	substitution	255	Arg	Asn	0.113	0.013
p.Arg255Gly	substitution	255	Arg	Gly	1.094	0.010
p.Arg255Leu	substitution	255	Arg	Leu	0.000	0.217
p.Arg255Lys	substitution	255	Arg	Lys	0.370	0.176
p.Arg255Met	substitution	255	Arg	Met	0.996	0.003
p.Arg255Pro	substitution	255	Arg	Pro	0.076	0.214
p.Arg255Ser	substitution	255	Arg	Ser	0.883	0.036
p.Arg255Trp	substitution	255	Arg	Trp	0.973	0.168
p.Arg26=	synonymous	26	Arg	NA	0.916	0.004
p.Arg26Ala	substitution	26	Arg	Ala	0.121	0.039
p.Arg26Asn	substitution	26	Arg	Asn	0.000	0.002
p.Arg26Asp	substitution	26	Arg	Asp	0.000	0.002
p.Arg26Cys	substitution	26	Arg	Cys	0.000	0.001
p.Arg26Gln	substitution	26	Arg	Gln	0.000	0.018
p.Arg26Glu	substitution	26	Arg	Glu	0.000	0.048
p.Arg26Gly	substitution	26	Arg	Gly	0.000	0.022
p.Arg26His	substitution	26	Arg	His	0.000	0.005
p.Arg26Ile	substitution	26	Arg	Ile	0.000	0.017
p.Arg26Leu	substitution	26	Arg	Leu	0.000	0.004
p.Arg26Lys	substitution	26	Arg	Lys	0.000	0.005
p.Arg26Met	substitution	26	Arg	Met	0.000	0.050
p.Arg26Phe	substitution	26	Arg	Phe	0.000	0.005
p.Arg26Pro	substitution	26	Arg	Pro	0.000	0.008
p.Arg26Ser	substitution	26	Arg	Ser	0.000	0.013
p.Arg26Ter	nonsense	26	Arg	Ter	0.000	0.004
p.Arg26Thr	substitution	26	Arg	Thr	0.000	0.026
p.Arg26Trp	substitution	26	Arg	Trp	0.000	0.004
p.Arg26Tyr	substitution	26	Arg	Tyr	0.000	0.006

p.Arg26Val	substitution	26	Arg	Val	0.000	0.026
p.Arg321=	synonymous	321	Arg	NA	1.074	0.025
p.Arg321Ala	substitution	321	Arg	Ala	0.955	0.044
p.Arg321Asn	substitution	321	Arg	Asn	0.431	0.007
p.Arg321Asp	substitution	321	Arg	Asp	0.189	0.006
p.Arg321Cys	substitution	321	Arg	Cys	0.583	0.469
p.Arg321Gln	substitution	321	Arg	Gln	0.440	0.004
p.Arg321Gly	substitution	321	Arg	Gly	0.993	0.018
p.Arg321His	substitution	321	Arg	His	0.863	0.005
p.Arg321Ile	substitution	321	Arg	Ile	0.818	0.016
p.Arg321Leu	substitution	321	Arg	Leu	0.886	0.005
p.Arg321Lys	substitution	321	Arg	Lys	1.366	0.003
p.Arg321Met	substitution	321	Arg	Met	0.537	0.005
p.Arg321Phe	substitution	321	Arg	Phe	1.127	0.001
p.Arg321Pro	substitution	321	Arg	Pro	0.546	0.223
p.Arg321Ser	substitution	321	Arg	Ser	0.962	0.045
p.Arg321Ter	nonsense	321	Arg	Ter	0.023	0.014
p.Arg321Thr	substitution	321	Arg	Thr	0.752	0.160
p.Arg321Trp	substitution	321	Arg	Trp	0.000	0.005
p.Arg321Val	substitution	321	Arg	Val	0.052	0.030
p.Arg325=	synonymous	325	Arg	NA	0.819	0.068
p.Arg325Ala	substitution	325	Arg	Ala	0.731	0.060
p.Arg325Asn	substitution	325	Arg	Asn	0.294	0.015
p.Arg325Asp	substitution	325	Arg	Asp	0.936	0.002
p.Arg325Cys	substitution	325	Arg	Cys	0.491	0.049
p.Arg325Gln	substitution	325	Arg	Gln	1.128	0.042
p.Arg325Glu	substitution	325	Arg	Glu	0.886	0.008
p.Arg325Gly	substitution	325	Arg	Gly	1.070	0.004
p.Arg325His	substitution	325	Arg	His	1.049	0.003
p.Arg325Ile	substitution	325	Arg	Ile	1.240	0.052
p.Arg325Leu	substitution	325	Arg	Leu	1.210	0.007
p.Arg325Lys	substitution	325	Arg	Lys	0.622	0.007
p.Arg325Met	substitution	325	Arg	Met	0.000	0.152
p.Arg325Phe	substitution	325	Arg	Phe	1.134	0.001
p.Arg325Pro	substitution	325	Arg	Pro	1.168	0.047
p.Arg325Ser	substitution	325	Arg	Ser	0.724	0.050
p.Arg325Ter	nonsense	325	Arg	Ter	0.033	0.048
p.Arg325Thr	substitution	325	Arg	Thr	0.693	0.012
p.Arg325Trp	substitution	325	Arg	Trp	1.010	0.016
p.Arg325Tyr	substitution	325	Arg	Tyr	0.567	0.004
p.Arg325Val	substitution	325	Arg	Val	0.022	0.071
p.Arg32=	synonymous	32	Arg	NA	0.937	0.001
p.Arg32Ala	substitution	32	Arg	Ala	0.660	0.003

p.Arg32Asn	substitution	32	Arg	Asn	1.049	0.001
p.Arg32Asp	substitution	32	Arg	Asp	0.264	0.001
p.Arg32Cys	substitution	32	Arg	Cys	0.908	0.008
p.Arg32Gln	substitution	32	Arg	Gln	1.140	0.000
p.Arg32Glu	substitution	32	Arg	Glu	0.586	0.001
p.Arg32Gly	substitution	32	Arg	Gly	0.896	0.000
p.Arg32His	substitution	32	Arg	His	0.595	0.004
p.Arg32Ile	substitution	32	Arg	Ile	1.087	0.004
p.Arg32Leu	substitution	32	Arg	Leu	0.689	0.009
p.Arg32Lys	substitution	32	Arg	Lys	0.673	0.001
p.Arg32Met	substitution	32	Arg	Met	0.944	0.001
p.Arg32Phe	substitution	32	Arg	Phe	0.555	0.001
p.Arg32Pro	substitution	32	Arg	Pro	0.000	0.010
p.Arg32Ser	substitution	32	Arg	Ser	0.691	0.008
p.Arg32Ter	nonsense	32	Arg	Ter	0.000	0.002
p.Arg32Thr	substitution	32	Arg	Thr	0.507	0.006
p.Arg32Trp	substitution	32	Arg	Trp	0.747	0.000
p.Arg32Tyr	substitution	32	Arg	Tyr	1.143	0.001
p.Arg32Val	substitution	32	Arg	Val	0.754	0.011
p.Arg355=	synonymous	355	Arg	NA	1.042	0.015
p.Arg355Ala	substitution	355	Arg	Ala	0.104	0.213
p.Arg355Asp	substitution	355	Arg	Asp	1.423	0.079
p.Arg355Cys	substitution	355	Arg	Cys	0.000	0.128
p.Arg355Gln	substitution	355	Arg	Gln	1.034	0.007
p.Arg355Gly	substitution	355	Arg	Gly	1.242	0.357
p.Arg355Leu	substitution	355	Arg	Leu	0.815	0.009
p.Arg355Lys	substitution	355	Arg	Lys	0.347	0.166
p.Arg355Met	substitution	355	Arg	Met	1.396	0.003
p.Arg355Pro	substitution	355	Arg	Pro	1.415	0.148
p.Arg355Ser	substitution	355	Arg	Ser	1.266	0.013
p.Arg355Thr	substitution	355	Arg	Thr	0.267	0.139
p.Arg355Trp	substitution	355	Arg	Trp	1.369	0.004
p.Arg355Tyr	substitution	355	Arg	Tyr	0.630	0.012
p.Arg355Val	substitution	355	Arg	Val	1.050	0.084
p.Asn118=	synonymous	118	Asn	NA	0.980	0.004
p.Asn118Ala	substitution	118	Asn	Ala	1.112	0.014
p.Asn118Arg	substitution	118	Asn	Arg	0.890	0.013
p.Asn118Asp	substitution	118	Asn	Asp	1.249	0.029
p.Asn118Cys	substitution	118	Asn	Cys	1.067	0.012
p.Asn118Gln	substitution	118	Asn	Gln	0.710	0.015
p.Asn118Glu	substitution	118	Asn	Glu	1.160	0.013
p.Asn118Gly	substitution	118	Asn	Gly	0.210	0.035
p.Asn118His	substitution	118	Asn	His	0.916	0.071

p.Asn118Ile	substitution	118	Asn	Ile	1.000	0.069
p.Asn118Leu	substitution	118	Asn	Leu	0.459	0.103
p.Asn118Lys	substitution	118	Asn	Lys	0.658	0.022
p.Asn118Me	substitution	118	Asn	Met	0.910	0.010
p.Asn118Phe	substitution	118	Asn	Phe	0.777	0.128
p.Asn118Pro	substitution	118	Asn	Pro	0.850	0.046
p.Asn118Ser	substitution	118	Asn	Ser	1.121	0.027
p.Asn118Ter	nonsense	118	Asn	Ter	0.158	0.018
p.Asn118Thr	substitution	118	Asn	Thr	1.173	0.009
p.Asn118Trp	substitution	118	Asn	Trp	0.812	0.010
p.Asn118Val	substitution	118	Asn	Val	0.948	0.007
p.Asn169=	synonymous	169	Asn	NA	1.080	0.007
p.Asn169Ala	substitution	169	Asn	Ala	0.445	0.012
p.Asn169Arg	substitution	169	Asn	Arg	0.841	0.006
p.Asn169Asp	substitution	169	Asn	Asp	0.471	0.017
p.Asn169Cys	substitution	169	Asn	Cys	0.239	0.032
p.Asn169Gln	substitution	169	Asn	Gln	0.269	0.018
p.Asn169Glu	substitution	169	Asn	Glu	0.375	0.006
p.Asn169Gly	substitution	169	Asn	Gly	0.372	0.028
p.Asn169His	substitution	169	Asn	His	0.906	0.031
p.Asn169Ile	substitution	169	Asn	Ile	0.100	0.099
p.Asn169Leu	substitution	169	Asn	Leu	0.733	0.034
p.Asn169Lys	substitution	169	Asn	Lys	0.893	0.027
p.Asn169Phe	substitution	169	Asn	Phe	0.363	0.083
p.Asn169Pro	substitution	169	Asn	Pro	0.000	0.067
p.Asn169Ser	substitution	169	Asn	Ser	0.920	0.010
p.Asn169Ter	nonsense	169	Asn	Ter	0.001	0.008
p.Asn169Thr	substitution	169	Asn	Thr	0.673	0.015
p.Asn169Trp	substitution	169	Asn	Trp	0.309	0.015
p.Asn169Tyr	substitution	169	Asn	Tyr	0.753	0.050
p.Asn169Val	substitution	169	Asn	Val	0.944	0.016
p.Asn171=	synonymous	171	Asn	NA	1.041	0.006
p.Asn171Ala	substitution	171	Asn	Ala	0.792	0.016
p.Asn171Arg	substitution	171	Asn	Arg	0.689	0.009
p.Asn171Asp	substitution	171	Asn	Asp	1.177	0.053
p.Asn171Gln	substitution	171	Asn	Gln	0.946	0.009
p.Asn171Glu	substitution	171	Asn	Glu	1.002	0.005
p.Asn171Gly	substitution	171	Asn	Gly	0.869	0.011
p.Asn171His	substitution	171	Asn	His	0.829	0.052
p.Asn171Ile	substitution	171	Asn	Ile	0.890	0.025
p.Asn171Leu	substitution	171	Asn	Leu	0.988	0.010
p.Asn171Lys	substitution	171	Asn	Lys	1.038	0.019
p.Asn171Me	substitution	171	Asn	Met	0.853	0.016

p.Asn171Phe	substitution	171	Asn	Phe	0.203	0.012
p.Asn171Pro	substitution	171	Asn	Pro	0.516	0.129
p.Asn171Ser	substitution	171	Asn	Ser	0.932	0.006
p.Asn171Ter	nonsense	171	Asn	Ter	0.000	0.008
p.Asn171Thr	substitution	171	Asn	Thr	0.918	0.014
p.Asn171Trp	substitution	171	Asn	Trp	1.101	0.007
p.Asn171Tyr	substitution	171	Asn	Tyr	1.065	0.010
p.Asn171Val	substitution	171	Asn	Val	0.550	0.323
p.Asn200=	synonymous	200	Asn	NA	0.789	0.002
p.Asn200Ala	substitution	200	Asn	Ala	0.914	0.022
p.Asn200Arg	substitution	200	Asn	Arg	0.101	0.017
p.Asn200Asp	substitution	200	Asn	Asp	1.153	0.001
p.Asn200Cys	substitution	200	Asn	Cys	0.950	0.032
p.Asn200Gln	substitution	200	Asn	Gln	0.000	0.064
p.Asn200Glu	substitution	200	Asn	Glu	0.586	0.003
p.Asn200Gly	substitution	200	Asn	Gly	0.731	0.033
p.Asn200His	substitution	200	Asn	His	1.187	0.046
p.Asn200Ile	substitution	200	Asn	Ile	0.998	0.021
p.Asn200Leu	substitution	200	Asn	Leu	0.288	0.022
p.Asn200Lys	substitution	200	Asn	Lys	1.110	0.002
p.Asn200Me	substitution	200	Asn	Met	0.837	0.003
p.Asn200Phe	substitution	200	Asn	Phe	1.281	0.010
p.Asn200Ser	substitution	200	Asn	Ser	0.744	0.073
p.Asn200Ter	nonsense	200	Asn	Ter	0.000	0.014
p.Asn200Thr	substitution	200	Asn	Thr	1.043	0.107
p.Asn200Trp	substitution	200	Asn	Trp	1.001	0.008
p.Asn200Tyr	substitution	200	Asn	Tyr	0.673	0.034
p.Asn200Val	substitution	200	Asn	Val	0.494	0.033
p.Asn322=	synonymous	322	Asn	NA	1.204	0.002
p.Asn322Ala	substitution	322	Asn	Ala	0.000	0.074
p.Asn322Arg	substitution	322	Asn	Arg	0.997	0.195
p.Asn322Asp	substitution	322	Asn	Asp	1.645	0.148
p.Asn322Cys	substitution	322	Asn	Cys	1.251	0.011
p.Asn322Gln	substitution	322	Asn	Gln	0.804	0.005
p.Asn322Glu	substitution	322	Asn	Glu	1.013	0.003
p.Asn322Gly	substitution	322	Asn	Gly	0.000	0.066
p.Asn322His	substitution	322	Asn	His	0.711	0.221
p.Asn322Ile	substitution	322	Asn	Ile	0.689	0.045
p.Asn322Leu	substitution	322	Asn	Leu	0.440	0.071
p.Asn322Lys	substitution	322	Asn	Lys	0.777	0.013
p.Asn322Me	substitution	322	Asn	Met	0.786	0.004
p.Asn322Phe	substitution	322	Asn	Phe	1.117	0.006
p.Asn322Pro	substitution	322	Asn	Pro	0.694	0.008

p.Asn322Ser	substitution	322	Asn	Ser	0.536	0.036
p.Asn322Ter	nonsense	322	Asn	Ter	0.000	0.062
p.Asn322Thr	substitution	322	Asn	Thr	0.519	0.109
p.Asn322Trp	substitution	322	Asn	Trp	1.152	0.003
p.Asn322Tyr	substitution	322	Asn	Tyr	0.676	0.018
p.Asn322Val	substitution	322	Asn	Val	0.865	0.008
p.Asn333=	synonymous	333	Asn	NA	1.431	0.008
p.Asn333Asp	substitution	333	Asn	Asp	0.000	0.063
p.Asn333Cys	substitution	333	Asn	Cys	0.000	0.035
p.Asn333His	substitution	333	Asn	His	1.178	0.143
p.Asn333Ile	substitution	333	Asn	Ile	1.239	0.006
p.Asn333Lys	substitution	333	Asn	Lys	1.202	0.022
p.Asn333Ser	substitution	333	Asn	Ser	0.000	0.126
p.Asn333Thr	substitution	333	Asn	Thr	0.000	0.224
p.Asn333Tyr	substitution	333	Asn	Tyr	0.301	0.167
p.Asn340=	synonymous	340	Asn	NA	0.944	0.005
p.Asn340Ala	substitution	340	Asn	Ala	0.138	0.019
p.Asn340Arg	substitution	340	Asn	Arg	0.625	0.136
p.Asn340Asp	substitution	340	Asn	Asp	0.953	0.220
p.Asn340Cys	substitution	340	Asn	Cys	0.000	0.027
p.Asn340Gln	substitution	340	Asn	Gln	1.254	0.003
p.Asn340Glu	substitution	340	Asn	Glu	0.000	0.143
p.Asn340Gly	substitution	340	Asn	Gly	0.906	0.086
p.Asn340His	substitution	340	Asn	His	0.000	0.036
p.Asn340Ile	substitution	340	Asn	Ile	1.290	0.005
p.Asn340Leu	substitution	340	Asn	Leu	0.675	0.038
p.Asn340Lys	substitution	340	Asn	Lys	0.974	0.005
p.Asn340Me	substitution	340	Asn	Met	1.346	0.053
p.Asn340Phe	substitution	340	Asn	Phe	0.556	0.014
p.Asn340Pro	substitution	340	Asn	Pro	0.000	0.021
p.Asn340Ser	substitution	340	Asn	Ser	0.720	0.330
p.Asn340Ter	nonsense	340	Asn	Ter	1.508	0.033
p.Asn340Thr	substitution	340	Asn	Thr	0.800	0.293
p.Asn340Trp	substitution	340	Asn	Trp	1.480	0.002
p.Asn340Tyr	substitution	340	Asn	Tyr	0.880	0.059
p.Asn340Val	substitution	340	Asn	Val	0.114	0.045
p.Asn349=	synonymous	349	Asn	NA	1.337	0.003
p.Asn349Ala	substitution	349	Asn	Ala	1.353	0.023
p.Asn349Arg	substitution	349	Asn	Arg	1.198	0.075
p.Asn349Asp	substitution	349	Asn	Asp	0.951	0.009
p.Asn349Cys	substitution	349	Asn	Cys	0.467	0.082
p.Asn349Gln	substitution	349	Asn	Gln	1.172	0.003
p.Asn349Glu	substitution	349	Asn	Glu	0.764	0.004

p.Asn349Gly	substitution	349	Asn	Gly	0.900	0.285
p.Asn349His	substitution	349	Asn	His	0.236	0.113
p.Asn349Ile	substitution	349	Asn	Ile	0.430	0.023
p.Asn349Leu	substitution	349	Asn	Leu	1.017	0.012
p.Asn349Lys	substitution	349	Asn	Lys	1.048	0.024
p.Asn349Met	substitution	349	Asn	Met	0.894	0.009
p.Asn349Pro	substitution	349	Asn	Pro	0.820	0.285
p.Asn349Ser	substitution	349	Asn	Ser	0.517	0.123
p.Asn349Ter	nonsense	349	Asn	Ter	0.997	0.003
p.Asn349Thr	substitution	349	Asn	Thr	0.948	0.007
p.Asn349Trp	substitution	349	Asn	Trp	1.671	0.008
p.Asn349Tyr	substitution	349	Asn	Tyr	0.439	0.018
p.Asn349Val	substitution	349	Asn	Val	0.777	0.156
p.Asn358=	synonymous	358	Asn	NA	1.102	0.021
p.Asn358Ala	substitution	358	Asn	Ala	1.305	0.022
p.Asn358Arg	substitution	358	Asn	Arg	1.239	0.017
p.Asn358Asp	substitution	358	Asn	Asp	0.000	0.288
p.Asn358Gly	substitution	358	Asn	Gly	0.120	0.017
p.Asn358Ile	substitution	358	Asn	Ile	1.300	0.065
p.Asn358Lys	substitution	358	Asn	Lys	1.413	0.003
p.Asn358Met	substitution	358	Asn	Met	1.456	0.008
p.Asn358Pro	substitution	358	Asn	Pro	1.271	0.003
p.Asn358Ser	substitution	358	Asn	Ser	1.219	0.062
p.Asn358Ter	nonsense	358	Asn	Ter	1.424	0.046
p.Asn358Thr	substitution	358	Asn	Thr	1.403	0.077
p.Asn358Tyr	substitution	358	Asn	Tyr	1.123	0.350
p.Asn88Ala	substitution	88	Asn	Ala	0.806	0.022
p.Asn88Arg	substitution	88	Asn	Arg	0.885	0.009
p.Asn88Asp	substitution	88	Asn	Asp	0.499	0.009
p.Asn88Cys	substitution	88	Asn	Cys	1.054	0.005
p.Asn88Glu	substitution	88	Asn	Glu	0.671	0.006
p.Asn88Gly	substitution	88	Asn	Gly	0.700	0.043
p.Asn88His	substitution	88	Asn	His	0.841	0.006
p.Asn88Ile	substitution	88	Asn	Ile	0.879	0.003
p.Asn88Leu	substitution	88	Asn	Leu	0.955	0.015
p.Asn88Lys	substitution	88	Asn	Lys	1.130	0.004
p.Asn88Met	substitution	88	Asn	Met	1.004	0.006
p.Asn88Phe	substitution	88	Asn	Phe	0.944	0.002
p.Asn88Pro	substitution	88	Asn	Pro	0.548	0.012
p.Asn88Ser	substitution	88	Asn	Ser	0.900	0.010
p.Asn88Ter	nonsense	88	Asn	Ter	0.000	0.026
p.Asn88Thr	substitution	88	Asn	Thr	1.044	0.032
p.Asn88Trp	substitution	88	Asn	Trp	1.046	0.003

p.Asn88Tyr	substitution	88	Asn	Tyr	0.985	0.003
p.Asn88Val	substitution	88	Asn	Val	0.476	0.019
p.Asp121Ala	substitution	121	Asp	Ala	0.000	0.009
p.Asp121Arg	substitution	121	Asp	Arg	0.000	0.007
p.Asp121Asn	substitution	121	Asp	Asn	0.091	0.008
p.Asp121Cys	substitution	121	Asp	Cys	0.789	0.003
p.Asp121Gln	substitution	121	Asp	Gln	0.000	0.017
p.Asp121Glu	substitution	121	Asp	Glu	0.406	0.038
p.Asp121Gly	substitution	121	Asp	Gly	0.127	0.009
p.Asp121His	substitution	121	Asp	His	0.009	0.011
p.Asp121Ile	substitution	121	Asp	Ile	0.000	0.007
p.Asp121Leu	substitution	121	Asp	Leu	0.034	0.004
p.Asp121Lys	substitution	121	Asp	Lys	0.000	0.011
p.Asp121Me	substitution	121	Asp	Met	0.000	0.006
p.Asp121Phe	substitution	121	Asp	Phe	0.000	0.010
p.Asp121Pro	substitution	121	Asp	Pro	0.011	0.012
p.Asp121Ser	substitution	121	Asp	Ser	0.103	0.024
p.Asp121Ter	nonsense	121	Asp	Ter	0.002	0.017
p.Asp121Thr	substitution	121	Asp	Thr	0.000	0.011
p.Asp121Trp	substitution	121	Asp	Trp	0.000	0.009
p.Asp121Tyr	substitution	121	Asp	Tyr	0.039	0.008
p.Asp121Val	substitution	121	Asp	Val	0.000	0.024
p.Asp178=	synonymous	178	Asp	NA	0.756	0.015
p.Asp178Ala	substitution	178	Asp	Ala	0.502	0.058
p.Asp178Arg	substitution	178	Asp	Arg	0.050	0.027
p.Asp178Asn	substitution	178	Asp	Asn	0.699	0.021
p.Asp178Cys	substitution	178	Asp	Cys	1.084	0.007
p.Asp178Gln	substitution	178	Asp	Gln	0.391	0.021
p.Asp178Glu	substitution	178	Asp	Glu	0.967	0.009
p.Asp178Gly	substitution	178	Asp	Gly	0.995	0.019
p.Asp178His	substitution	178	Asp	His	0.935	0.007
p.Asp178Ile	substitution	178	Asp	Ile	0.000	0.034
p.Asp178Leu	substitution	178	Asp	Leu	0.836	0.038
p.Asp178Lys	substitution	178	Asp	Lys	0.190	0.034
p.Asp178Me	substitution	178	Asp	Met	0.899	0.007
p.Asp178Phe	substitution	178	Asp	Phe	0.383	0.011
p.Asp178Pro	substitution	178	Asp	Pro	0.000	0.168
p.Asp178Ser	substitution	178	Asp	Ser	0.793	0.026
p.Asp178Ter	nonsense	178	Asp	Ter	0.000	0.088
p.Asp178Thr	substitution	178	Asp	Thr	0.560	0.014
p.Asp178Trp	substitution	178	Asp	Trp	0.396	0.010
p.Asp178Tyr	substitution	178	Asp	Tyr	1.001	0.011
p.Asp178Val	substitution	178	Asp	Val	0.838	0.008

p.Asp228=	synonymous	228	Asp	NA	0.740	0.002
p.Asp228Ala	substitution	228	Asp	Ala	0.932	0.149
p.Asp228Arg	substitution	228	Asp	Arg	1.064	0.047
p.Asp228Asn	substitution	228	Asp	Asn	1.061	0.165
p.Asp228Cys	substitution	228	Asp	Cys	0.960	0.004
p.Asp228Gln	substitution	228	Asp	Gln	1.363	0.006
p.Asp228Glu	substitution	228	Asp	Glu	1.268	0.002
p.Asp228Gly	substitution	228	Asp	Gly	0.935	0.099
p.Asp228His	substitution	228	Asp	His	0.000	0.092
p.Asp228Ile	substitution	228	Asp	Ile	0.000	0.007
p.Asp228Leu	substitution	228	Asp	Leu	0.081	0.009
p.Asp228Lys	substitution	228	Asp	Lys	0.037	0.025
p.Asp228Pro	substitution	228	Asp	Pro	1.525	0.033
p.Asp228Ser	substitution	228	Asp	Ser	0.689	0.104
p.Asp228Ter	nonsense	228	Asp	Ter	0.000	0.013
p.Asp228Thr	substitution	228	Asp	Thr	0.041	0.039
p.Asp228Trp	substitution	228	Asp	Trp	0.000	0.189
p.Asp228Tyr	substitution	228	Asp	Tyr	0.000	0.042
p.Asp228Val	substitution	228	Asp	Val	0.166	0.012
p.Asp230=	synonymous	230	Asp	NA	0.149	0.447
p.Asp230Ala	substitution	230	Asp	Ala	0.317	0.029
p.Asp230Arg	substitution	230	Asp	Arg	0.994	0.009
p.Asp230Asn	substitution	230	Asp	Asn	0.853	0.143
p.Asp230Cys	substitution	230	Asp	Cys	0.000	0.083
p.Asp230Gln	substitution	230	Asp	Gln	1.379	0.001
p.Asp230Glu	substitution	230	Asp	Glu	1.044	0.006
p.Asp230Gly	substitution	230	Asp	Gly	0.505	0.039
p.Asp230His	substitution	230	Asp	His	0.000	0.197
p.Asp230Ile	substitution	230	Asp	Ile	1.325	0.004
p.Asp230Leu	substitution	230	Asp	Leu	0.000	0.026
p.Asp230Lys	substitution	230	Asp	Lys	0.715	0.003
p.Asp230Me	substitution	230	Asp	Met	1.483	0.003
p.Asp230Phe	substitution	230	Asp	Phe	0.000	0.120
p.Asp230Pro	substitution	230	Asp	Pro	0.885	0.164
p.Asp230Ser	substitution	230	Asp	Ser	1.074	0.023
p.Asp230Ter	nonsense	230	Asp	Ter	0.227	0.014
p.Asp230Thr	substitution	230	Asp	Thr	0.000	0.030
p.Asp230Trp	substitution	230	Asp	Trp	1.010	0.003
p.Asp230Tyr	substitution	230	Asp	Tyr	0.512	0.038
p.Asp230Val	substitution	230	Asp	Val	0.908	0.014
p.Asp233Ala	substitution	233	Asp	Ala	0.532	0.021
p.Asp233Arg	substitution	233	Asp	Arg	0.693	0.008
p.Asp233Asn	substitution	233	Asp	Asn	1.066	0.003

p.Asp233Cys	substitution	233	Asp	Cys		0.856	0.003
p.Asp233Gln	substitution	233	Asp	Gln		0.703	0.002
p.Asp233Glu	substitution	233	Asp	Glu		0.908	0.002
p.Asp233Gly	substitution	233	Asp	Gly		0.537	0.010
p.Asp233His	substitution	233	Asp	His		1.003	0.003
p.Asp233Ile	substitution	233	Asp	Ile		0.722	0.002
p.Asp233Leu	substitution	233	Asp	Leu		0.054	0.043
p.Asp233Lys	substitution	233	Asp	Lys		0.776	0.004
p.Asp233Me	substitution	233	Asp	Met		0.556	0.013
p.Asp233Pro	substitution	233	Asp	Pro		0.387	0.094
p.Asp233Ser	substitution	233	Asp	Ser		0.864	0.106
p.Asp233Ter	nonsense	233	Asp	Ter		0.000	0.006
p.Asp233Thr	substitution	233	Asp	Thr		0.676	0.002
p.Asp233Trp	substitution	233	Asp	Trp		0.029	0.006
p.Asp233Tyr	substitution	233	Asp	Tyr		0.597	0.001
p.Asp233Val	substitution	233	Asp	Val		0.561	0.076
p.Asp240Ala	substitution	240	Asp	Ala		0.627	0.090
p.Asp240Arg	substitution	240	Asp	Arg		0.000	0.066
p.Asp240Asn	substitution	240	Asp	Asn		1.283	0.004
p.Asp240Cys	substitution	240	Asp	Cys		0.968	0.002
p.Asp240Gln	substitution	240	Asp	Gln		1.083	0.004
p.Asp240Glu	substitution	240	Asp	Glu		1.271	0.001
p.Asp240Gly	substitution	240	Asp	Gly		0.000	0.032
p.Asp240Ile	substitution	240	Asp	Ile		0.425	0.005
p.Asp240Leu	substitution	240	Asp	Leu		0.000	0.033
p.Asp240Lys	substitution	240	Asp	Lys		0.853	0.011
p.Asp240Me	substitution	240	Asp	Met		0.145	0.019
p.Asp240Phe	substitution	240	Asp	Phe		0.810	0.015
p.Asp240Pro	substitution	240	Asp	Pro		0.134	0.049
p.Asp240Ser	substitution	240	Asp	Ser		0.000	0.035
p.Asp240Ter	nonsense	240	Asp	Ter		0.030	0.077
p.Asp240Thr	substitution	240	Asp	Thr		0.421	0.008
p.Asp240Trp	substitution	240	Asp	Trp		0.000	0.022
p.Asp240Tyr	substitution	240	Asp	Tyr		1.072	0.006
p.Asp240Val	substitution	240	Asp	Val		0.930	0.191
p.Asp273Ala	substitution	273	Asp	Ala		0.146	0.244
p.Asp273Arg	substitution	273	Asp	Arg		1.126	0.033
p.Asp273Asn	substitution	273	Asp	Asn		1.436	0.013
p.Asp273Glu	substitution	273	Asp	Glu		0.904	0.026
p.Asp273Gly	substitution	273	Asp	Gly		0.000	0.532
p.Asp273His	substitution	273	Asp	His		0.469	0.344
p.Asp273Ile	substitution	273	Asp	Ile		0.381	0.042
p.Asp273Phe	substitution	273	Asp	Phe		0.000	0.028

p.Asp273Ser	substitution	273	Asp	Ser		0.158	0.039
p.Asp273Tyr	substitution	273	Asp	Tyr		0.891	0.009
p.Asp286=	synonymous	286	Asp	NA		0.925	0.007
p.Asp286Ala	substitution	286	Asp	Ala		0.285	0.033
p.Asp286Arg	substitution	286	Asp	Arg		0.922	0.005
p.Asp286Asn	substitution	286	Asp	Asn		1.098	0.010
p.Asp286Cys	substitution	286	Asp	Cys		0.591	0.009
p.Asp286Gln	substitution	286	Asp	Gln		0.884	0.004
p.Asp286Glu	substitution	286	Asp	Glu		1.066	0.011
p.Asp286Gly	substitution	286	Asp	Gly		0.556	0.006
p.Asp286His	substitution	286	Asp	His		0.708	0.020
p.Asp286Ile	substitution	286	Asp	Ile		1.520	0.033
p.Asp286Leu	substitution	286	Asp	Leu		0.656	0.006
p.Asp286Lys	substitution	286	Asp	Lys		0.366	0.006
p.Asp286Phe	substitution	286	Asp	Phe		0.506	0.020
p.Asp286Pro	substitution	286	Asp	Pro		0.652	0.069
p.Asp286Ser	substitution	286	Asp	Ser		0.621	0.073
p.Asp286Ter	nonsense	286	Asp	Ter		0.000	0.097
p.Asp286Thr	substitution	286	Asp	Thr		0.613	0.031
p.Asp286Trp	substitution	286	Asp	Trp		0.729	0.005
p.Asp286Tyr	substitution	286	Asp	Tyr		0.227	0.070
p.Asp286Val	substitution	286	Asp	Val		0.898	0.014
p.Asp289Ala	substitution	289	Asp	Ala		1.043	0.004
p.Asp289Arg	substitution	289	Asp	Arg		0.758	0.028
p.Asp289Asn	substitution	289	Asp	Asn		0.448	0.036
p.Asp289Cys	substitution	289	Asp	Cys		0.181	0.018
p.Asp289Gln	substitution	289	Asp	Gln		1.423	0.015
p.Asp289Glu	substitution	289	Asp	Glu		0.752	0.004
p.Asp289Gly	substitution	289	Asp	Gly		0.619	0.078
p.Asp289Ile	substitution	289	Asp	Ile		1.398	0.006
p.Asp289Leu	substitution	289	Asp	Leu		0.685	0.228
p.Asp289Lys	substitution	289	Asp	Lys		0.151	0.011
p.Asp289Phe	substitution	289	Asp	Phe		1.079	0.010
p.Asp289Pro	substitution	289	Asp	Pro		1.523	0.042
p.Asp289Ser	substitution	289	Asp	Ser		0.786	0.095
p.Asp289Ter	nonsense	289	Asp	Ter		0.000	0.013
p.Asp289Thr	substitution	289	Asp	Thr		1.217	0.143
p.Asp289Trp	substitution	289	Asp	Trp		1.067	0.007
p.Asp289Tyr	substitution	289	Asp	Tyr		0.865	0.008
p.Asp289Val	substitution	289	Asp	Val		1.052	0.042
p.Asp307Ala	substitution	307	Asp	Ala		0.000	0.138
p.Asp307Arg	substitution	307	Asp	Arg		0.291	0.157
p.Asp307Asn	substitution	307	Asp	Asn		1.183	0.009

p.Asp307Cys	substitution	307	Asp	Cys	0.812	0.003
p.Asp307Gln	substitution	307	Asp	Gln	0.146	0.258
p.Asp307Glu	substitution	307	Asp	Glu	0.523	0.052
p.Asp307Gly	substitution	307	Asp	Gly	1.223	0.027
p.Asp307His	substitution	307	Asp	His	1.323	0.003
p.Asp307Ile	substitution	307	Asp	Ile	0.000	0.166
p.Asp307Leu	substitution	307	Asp	Leu	0.000	0.183
p.Asp307Lys	substitution	307	Asp	Lys	1.907	0.007
p.Asp307Phe	substitution	307	Asp	Phe	1.475	0.002
p.Asp307Ser	substitution	307	Asp	Ser	0.639	0.017
p.Asp307Ter	nonsense	307	Asp	Ter	0.264	0.044
p.Asp307Thr	substitution	307	Asp	Thr	0.403	0.229
p.Asp307Trp	substitution	307	Asp	Trp	1.323	0.002
p.Asp307Tyr	substitution	307	Asp	Tyr	0.808	0.004
p.Asp307Val	substitution	307	Asp	Val	0.672	0.210
p.Asp311Ala	substitution	311	Asp	Ala	1.232	0.062
p.Asp311Arg	substitution	311	Asp	Arg	0.832	0.014
p.Asp311Asn	substitution	311	Asp	Asn	0.925	0.002
p.Asp311Cys	substitution	311	Asp	Cys	0.000	0.022
p.Asp311Gln	substitution	311	Asp	Gln	0.875	0.003
p.Asp311Glu	substitution	311	Asp	Glu	1.148	0.014
p.Asp311Gly	substitution	311	Asp	Gly	1.087	0.064
p.Asp311His	substitution	311	Asp	His	1.129	0.013
p.Asp311Ile	substitution	311	Asp	Ile	0.163	0.009
p.Asp311Leu	substitution	311	Asp	Leu	0.237	0.059
p.Asp311Lys	substitution	311	Asp	Lys	0.000	0.024
p.Asp311Met	substitution	311	Asp	Met	1.162	0.002
p.Asp311Phe	substitution	311	Asp	Phe	0.012	0.015
p.Asp311Pro	substitution	311	Asp	Pro	0.705	0.022
p.Asp311Ser	substitution	311	Asp	Ser	0.370	0.030
p.Asp311Ter	nonsense	311	Asp	Ter	0.000	0.014
p.Asp311Thr	substitution	311	Asp	Thr	0.372	0.031
p.Asp311Trp	substitution	311	Asp	Trp	0.680	0.008
p.Asp311Tyr	substitution	311	Asp	Tyr	0.523	0.005
p.Asp311Val	substitution	311	Asp	Val	0.901	0.027
p.Asp312=	synonymous	312	Asp	NA	1.109	0.003
p.Asp312Ala	substitution	312	Asp	Ala	0.925	0.092
p.Asp312Arg	substitution	312	Asp	Arg	0.957	0.074
p.Asp312Asn	substitution	312	Asp	Asn	1.251	0.009
p.Asp312Cys	substitution	312	Asp	Cys	1.128	0.034
p.Asp312Glu	substitution	312	Asp	Glu	0.804	0.004
p.Asp312Gly	substitution	312	Asp	Gly	0.758	1.048
p.Asp312His	substitution	312	Asp	His	0.994	0.020

p.Asp312Ile	substitution	312	Asp	Ile	0.000	0.029
p.Asp312Leu	substitution	312	Asp	Leu	0.410	0.041
p.Asp312Lys	substitution	312	Asp	Lys	0.000	0.037
p.Asp312Me	substitution	312	Asp	Met	0.610	0.011
p.Asp312Phe	substitution	312	Asp	Phe	0.367	0.108
p.Asp312Pro	substitution	312	Asp	Pro	0.032	0.060
p.Asp312Ser	substitution	312	Asp	Ser	0.000	0.035
p.Asp312Ter	nonsense	312	Asp	Ter	0.000	0.020
p.Asp312Thr	substitution	312	Asp	Thr	0.000	0.010
p.Asp312Trp	substitution	312	Asp	Trp	0.363	0.008
p.Asp312Tyr	substitution	312	Asp	Tyr	0.919	0.009
p.Asp312Val	substitution	312	Asp	Val	0.427	0.063
p.Asp352Ala	substitution	352	Asp	Ala	0.976	0.012
p.Asp352Arg	substitution	352	Asp	Arg	0.833	0.012
p.Asp352Asn	substitution	352	Asp	Asn	0.440	0.014
p.Asp352Cys	substitution	352	Asp	Cys	0.000	0.029
p.Asp352Gln	substitution	352	Asp	Gln	0.868	0.012
p.Asp352Glu	substitution	352	Asp	Glu	0.193	0.026
p.Asp352Gly	substitution	352	Asp	Gly	0.000	0.574
p.Asp352His	substitution	352	Asp	His	1.225	0.004
p.Asp352Ile	substitution	352	Asp	Ile	0.000	0.036
p.Asp352Leu	substitution	352	Asp	Leu	0.935	0.015
p.Asp352Lys	substitution	352	Asp	Lys	0.782	0.004
p.Asp352Me	substitution	352	Asp	Met	0.723	0.005
p.Asp352Phe	substitution	352	Asp	Phe	1.159	0.002
p.Asp352Pro	substitution	352	Asp	Pro	1.119	0.021
p.Asp352Ser	substitution	352	Asp	Ser	0.773	0.003
p.Asp352Ter	nonsense	352	Asp	Ter	0.796	0.062
p.Asp352Thr	substitution	352	Asp	Thr	0.528	0.100
p.Asp352Trp	substitution	352	Asp	Trp	0.729	0.003
p.Asp352Tyr	substitution	352	Asp	Tyr	1.081	0.004
p.Asp352Val	substitution	352	Asp	Val	0.659	0.012
p.Asp359Ala	substitution	359	Asp	Ala	1.528	0.007
p.Asp359Arg	substitution	359	Asp	Arg	1.316	0.004
p.Asp359Asn	substitution	359	Asp	Asn	0.706	0.036
p.Asp359Cys	substitution	359	Asp	Cys	1.114	0.006
p.Asp359Gln	substitution	359	Asp	Gln	0.256	0.068
p.Asp359Glu	substitution	359	Asp	Glu	0.884	0.048
p.Asp359Gly	substitution	359	Asp	Gly	1.009	0.046
p.Asp359His	substitution	359	Asp	His	1.048	0.006
p.Asp359Leu	substitution	359	Asp	Leu	0.425	0.059
p.Asp359Lys	substitution	359	Asp	Lys	0.212	0.012
p.Asp359Me	substitution	359	Asp	Met	0.000	0.106

p.Asp359Phe	substitution	359	Asp	Phe		0.564	0.029
p.Asp359Ser	substitution	359	Asp	Ser		0.050	0.021
p.Asp359Ter	nonsense	359	Asp	Ter		1.328	0.003
p.Asp359Trp	substitution	359	Asp	Trp		1.041	0.008
p.Asp359Tyr	substitution	359	Asp	Tyr		1.067	0.013
p.Asp359Val	substitution	359	Asp	Val		0.080	0.124
p.Asp36=	synonymous	36	Asp	NA		0.930	0.001
p.Asp36Ala	substitution	36	Asp	Ala		0.755	0.026
p.Asp36Arg	substitution	36	Asp	Arg		0.574	0.012
p.Asp36Asn	substitution	36	Asp	Asn		0.836	0.017
p.Asp36Cys	substitution	36	Asp	Cys		0.502	0.002
p.Asp36Gln	substitution	36	Asp	Gln		1.333	0.003
p.Asp36Glu	substitution	36	Asp	Glu		0.868	0.002
p.Asp36Gly	substitution	36	Asp	Gly		1.036	0.010
p.Asp36His	substitution	36	Asp	His		1.048	0.003
p.Asp36Ile	substitution	36	Asp	Ile		0.527	0.003
p.Asp36Leu	substitution	36	Asp	Leu		0.714	0.040
p.Asp36Lys	substitution	36	Asp	Lys		1.108	0.004
p.Asp36Met	substitution	36	Asp	Met		1.169	0.002
p.Asp36Pro	substitution	36	Asp	Pro		0.000	0.063
p.Asp36Ser	substitution	36	Asp	Ser		0.954	0.018
p.Asp36Ter	nonsense	36	Asp	Ter		0.141	0.006
p.Asp36Thr	substitution	36	Asp	Thr		0.903	0.002
p.Asp36Trp	substitution	36	Asp	Trp		0.562	0.002
p.Asp36Tyr	substitution	36	Asp	Tyr		0.835	0.012
p.Asp36Val	substitution	36	Asp	Val		0.985	0.008
p.Asp61=	synonymous	61	Asp	NA		0.874	0.042
p.Asp61Ala	substitution	61	Asp	Ala		0.000	0.484
p.Asp61Arg	substitution	61	Asp	Arg		0.000	0.880
p.Asp61Asn	substitution	61	Asp	Asn		0.000	0.579
p.Asp61Cys	substitution	61	Asp	Cys		0.000	0.772
p.Asp61Gln	substitution	61	Asp	Gln		0.000	0.159
p.Asp61Glu	substitution	61	Asp	Glu		1.091	0.241
p.Asp61Gly	substitution	61	Asp	Gly		0.040	0.203
p.Asp61His	substitution	61	Asp	His		0.256	0.497
p.Asp61Leu	substitution	61	Asp	Leu		0.897	0.447
p.Asp61Lys	substitution	61	Asp	Lys		0.358	0.044
p.Asp61Phe	substitution	61	Asp	Phe		0.000	0.038
p.Asp61Pro	substitution	61	Asp	Pro		0.061	0.243
p.Asp61Ser	substitution	61	Asp	Ser		0.000	0.340
p.Asp61Ter	nonsense	61	Asp	Ter		0.000	0.179
p.Asp61Thr	substitution	61	Asp	Thr		0.000	0.135
p.Asp61Trp	substitution	61	Asp	Trp		0.427	0.831

p.Asp61Tyr	substitution	61	Asp	Tyr	0.335	0.127
p.Asp61Val	substitution	61	Asp	Val	0.000	0.245
p.Asp65Ala	substitution	65	Asp	Ala	0.528	0.004
p.Asp65Arg	substitution	65	Asp	Arg	0.987	0.002
p.Asp65Asn	substitution	65	Asp	Asn	0.316	0.012
p.Asp65Cys	substitution	65	Asp	Cys	0.391	0.008
p.Asp65Gln	substitution	65	Asp	Gln	0.585	0.022
p.Asp65Glu	substitution	65	Asp	Glu	1.210	0.011
p.Asp65Gly	substitution	65	Asp	Gly	0.710	0.017
p.Asp65His	substitution	65	Asp	His	0.838	0.011
p.Asp65Ile	substitution	65	Asp	Ile	0.645	0.013
p.Asp65Leu	substitution	65	Asp	Leu	0.171	0.015
p.Asp65Lys	substitution	65	Asp	Lys	1.134	0.004
p.Asp65Met	substitution	65	Asp	Met	0.121	0.011
p.Asp65Phe	substitution	65	Asp	Phe	0.784	0.004
p.Asp65Pro	substitution	65	Asp	Pro	0.761	0.053
p.Asp65Ser	substitution	65	Asp	Ser	0.824	0.030
p.Asp65Ter	nonsense	65	Asp	Ter	0.000	0.015
p.Asp65Thr	substitution	65	Asp	Thr	0.763	0.014
p.Asp65Trp	substitution	65	Asp	Trp	0.655	0.005
p.Asp65Tyr	substitution	65	Asp	Tyr	0.778	0.004
p.Asp65Val	substitution	65	Asp	Val	0.767	0.010
p.Asp91=	synonymous	91	Asp	NA	1.333	0.002
p.Asp91Ala	substitution	91	Asp	Ala	0.616	0.014
p.Asp91Arg	substitution	91	Asp	Arg	0.126	0.132
p.Asp91Asn	substitution	91	Asp	Asn	0.956	0.010
p.Asp91Cys	substitution	91	Asp	Cys	0.303	0.021
p.Asp91Gln	substitution	91	Asp	Gln	0.464	0.017
p.Asp91Glu	substitution	91	Asp	Glu	1.065	0.039
p.Asp91Gly	substitution	91	Asp	Gly	0.312	0.013
p.Asp91His	substitution	91	Asp	His	0.147	0.082
p.Asp91Ile	substitution	91	Asp	Ile	0.133	0.055
p.Asp91Leu	substitution	91	Asp	Leu	0.029	0.054
p.Asp91Lys	substitution	91	Asp	Lys	0.965	0.015
p.Asp91Met	substitution	91	Asp	Met	0.060	0.012
p.Asp91Phe	substitution	91	Asp	Phe	0.000	0.046
p.Asp91Pro	substitution	91	Asp	Pro	0.235	0.084
p.Asp91Ser	substitution	91	Asp	Ser	0.711	0.027
p.Asp91Ter	nonsense	91	Asp	Ter	0.000	0.033
p.Asp91Thr	substitution	91	Asp	Thr	0.045	0.017
p.Asp91Trp	substitution	91	Asp	Trp	0.000	0.044
p.Asp91Tyr	substitution	91	Asp	Tyr	0.248	0.120
p.Asp91Val	substitution	91	Asp	Val	0.199	0.007

p.Asp99=	synonymous	99	Asp	NA		1.127	0.012
p.Asp99Ala	substitution	99	Asp	Ala		0.125	0.045
p.Asp99Arg	substitution	99	Asp	Arg		0.000	0.033
p.Asp99Asn	substitution	99	Asp	Asn		0.192	0.092
p.Asp99Cys	substitution	99	Asp	Cys		0.000	0.037
p.Asp99Gln	substitution	99	Asp	Gln		0.000	0.114
p.Asp99Glu	substitution	99	Asp	Glu		0.473	0.433
p.Asp99Gly	substitution	99	Asp	Gly		0.130	0.057
p.Asp99His	substitution	99	Asp	His		0.505	0.063
p.Asp99Ile	substitution	99	Asp	Ile		0.000	0.095
p.Asp99Leu	substitution	99	Asp	Leu		0.000	0.023
p.Asp99Lys	substitution	99	Asp	Lys		0.000	0.165
p.Asp99Met	substitution	99	Asp	Met		0.188	0.021
p.Asp99Phe	substitution	99	Asp	Phe		0.000	0.155
p.Asp99Pro	substitution	99	Asp	Pro		0.329	0.014
p.Asp99Ser	substitution	99	Asp	Ser		0.000	0.026
p.Asp99Ter	nonsense	99	Asp	Ter		0.352	0.021
p.Asp99Thr	substitution	99	Asp	Thr		0.000	0.050
p.Asp99Trp	substitution	99	Asp	Trp		0.000	0.013
p.Asp99Tyr	substitution	99	Asp	Tyr		0.000	0.064
p.Asp99Val	substitution	99	Asp	Val		0.000	0.056
p.Cys114=	synonymous	114	Cys	NA		0.942	0.004
p.Cys114Ala	substitution	114	Cys	Ala		0.362	0.084
p.Cys114Arg	substitution	114	Cys	Arg		0.726	0.032
p.Cys114Asn	substitution	114	Cys	Asn		0.780	0.025
p.Cys114Asp	substitution	114	Cys	Asp		0.813	0.022
p.Cys114Gln	substitution	114	Cys	Gln		0.956	0.007
p.Cys114Glu	substitution	114	Cys	Glu		0.000	0.017
p.Cys114Gly	substitution	114	Cys	Gly		0.561	0.021
p.Cys114His	substitution	114	Cys	His		0.270	0.078
p.Cys114Ile	substitution	114	Cys	Ile		1.030	0.015
p.Cys114Leu	substitution	114	Cys	Leu		0.796	0.005
p.Cys114Lys	substitution	114	Cys	Lys		0.597	0.015
p.Cys114Phe	substitution	114	Cys	Phe		0.633	0.010
p.Cys114Pro	substitution	114	Cys	Pro		0.836	0.117
p.Cys114Ser	substitution	114	Cys	Ser		0.971	0.029
p.Cys114Ter	nonsense	114	Cys	Ter		0.010	0.064
p.Cys114Thr	substitution	114	Cys	Thr		1.112	0.012
p.Cys114Trp	substitution	114	Cys	Trp		0.682	0.010
p.Cys114Tyr	substitution	114	Cys	Tyr		0.405	0.138
p.Cys114Val	substitution	114	Cys	Val		0.386	0.043
p.Cys211=	synonymous	211	Cys	NA		1.073	0.004
p.Cys211Ala	substitution	211	Cys	Ala		0.845	0.045

p.Cys211Arg	substitution	211	Cys	Arg	0.000	0.053
p.Cys211Glu	substitution	211	Cys	Glu	0.000	0.014
p.Cys211Gly	substitution	211	Cys	Gly	0.580	0.054
p.Cys211Leu	substitution	211	Cys	Leu	0.838	0.141
p.Cys211Lys	substitution	211	Cys	Lys	0.000	0.032
p.Cys211Met	substitution	211	Cys	Met	1.436	0.003
p.Cys211Phe	substitution	211	Cys	Phe	1.298	0.017
p.Cys211Pro	substitution	211	Cys	Pro	0.000	0.066
p.Cys211Ser	substitution	211	Cys	Ser	0.908	0.086
p.Cys211Ter	nonsense	211	Cys	Ter	0.057	0.169
p.Cys211Thr	substitution	211	Cys	Thr	0.288	0.119
p.Cys211Trp	substitution	211	Cys	Trp	0.535	0.087
p.Cys211Tyr	substitution	211	Cys	Tyr	0.748	0.119
p.Cys211Val	substitution	211	Cys	Val	0.932	0.638
p.Cys247=	synonymous	247	Cys	NA	1.344	0.004
p.Cys247Ala	substitution	247	Cys	Ala	1.288	0.064
p.Cys247Arg	substitution	247	Cys	Arg	0.000	0.035
p.Cys247Asn	substitution	247	Cys	Asn	0.475	0.011
p.Cys247Gln	substitution	247	Cys	Gln	0.000	0.058
p.Cys247Glu	substitution	247	Cys	Glu	0.225	0.009
p.Cys247Gly	substitution	247	Cys	Gly	0.461	0.053
p.Cys247His	substitution	247	Cys	His	0.000	0.189
p.Cys247Ile	substitution	247	Cys	Ile	0.000	0.120
p.Cys247Leu	substitution	247	Cys	Leu	0.263	0.022
p.Cys247Met	substitution	247	Cys	Met	0.144	0.221
p.Cys247Phe	substitution	247	Cys	Phe	0.387	0.134
p.Cys247Ser	substitution	247	Cys	Ser	0.785	0.057
p.Cys247Ter	nonsense	247	Cys	Ter	0.000	0.081
p.Cys247Thr	substitution	247	Cys	Thr	0.409	0.020
p.Cys247Trp	substitution	247	Cys	Trp	0.128	0.009
p.Cys247Tyr	substitution	247	Cys	Tyr	0.822	0.086
p.Cys247Val	substitution	247	Cys	Val	0.716	0.008
p.Cys261=	synonymous	261	Cys	NA	1.305	0.005
p.Cys261Ala	substitution	261	Cys	Ala	0.000	0.054
p.Cys261Arg	substitution	261	Cys	Arg	0.036	0.029
p.Cys261Asn	substitution	261	Cys	Asn	0.000	0.130
p.Cys261Asp	substitution	261	Cys	Asp	0.274	0.031
p.Cys261Gln	substitution	261	Cys	Gln	0.057	0.128
p.Cys261Glu	substitution	261	Cys	Glu	0.000	0.015
p.Cys261Gly	substitution	261	Cys	Gly	0.218	0.315
p.Cys261His	substitution	261	Cys	His	0.000	0.027
p.Cys261Ile	substitution	261	Cys	Ile	0.000	0.062
p.Cys261Leu	substitution	261	Cys	Leu	0.000	0.105

p.Cys261Lys	substitution	261	Cys	Lys	0.000	0.030
p.Cys261Met	substitution	261	Cys	Met	0.000	0.114
p.Cys261Phe	substitution	261	Cys	Phe	0.265	0.035
p.Cys261Pro	substitution	261	Cys	Pro	0.000	0.098
p.Cys261Ser	substitution	261	Cys	Ser	0.000	0.090
p.Cys261Ter	nonsense	261	Cys	Ter	0.061	0.100
p.Cys261Trp	substitution	261	Cys	Trp	0.600	0.402
p.Cys261Tyr	substitution	261	Cys	Tyr	0.576	0.149
p.Cys261Val	substitution	261	Cys	Val	0.000	0.011
p.Gln153=	synonymous	153	Gln	NA	1.072	0.009
p.Gln153Ala	substitution	153	Gln	Ala	0.276	0.269
p.Gln153Arg	substitution	153	Gln	Arg	0.061	0.023
p.Gln153Asn	substitution	153	Gln	Asn	0.000	0.042
p.Gln153Asp	substitution	153	Gln	Asp	0.000	0.035
p.Gln153Cys	substitution	153	Gln	Cys	0.592	0.013
p.Gln153Glu	substitution	153	Gln	Glu	0.288	0.069
p.Gln153Gly	substitution	153	Gln	Gly	0.605	0.006
p.Gln153His	substitution	153	Gln	His	0.447	0.048
p.Gln153Ile	substitution	153	Gln	Ile	0.075	0.019
p.Gln153Leu	substitution	153	Gln	Leu	0.548	0.022
p.Gln153Lys	substitution	153	Gln	Lys	0.123	0.025
p.Gln153Me	substitution	153	Gln	Met	0.552	0.008
p.Gln153Phe	substitution	153	Gln	Phe	0.000	0.313
p.Gln153Pro	substitution	153	Gln	Pro	0.884	0.015
p.Gln153Ser	substitution	153	Gln	Ser	0.671	0.007
p.Gln153Ter	nonsense	153	Gln	Ter	0.088	0.315
p.Gln153Thr	substitution	153	Gln	Thr	0.271	0.136
p.Gln153Tyr	substitution	153	Gln	Tyr	0.981	0.012
p.Gln153Val	substitution	153	Gln	Val	0.388	0.223
p.Gln155=	synonymous	155	Gln	NA	1.403	0.029
p.Gln155Ala	substitution	155	Gln	Ala	1.112	0.004
p.Gln155Arg	substitution	155	Gln	Arg	0.880	0.011
p.Gln155Asp	substitution	155	Gln	Asp	0.496	0.014
p.Gln155Cys	substitution	155	Gln	Cys	1.029	0.140
p.Gln155Glu	substitution	155	Gln	Glu	0.813	0.007
p.Gln155Gly	substitution	155	Gln	Gly	0.860	0.005
p.Gln155His	substitution	155	Gln	His	0.959	0.009
p.Gln155Ile	substitution	155	Gln	Ile	1.263	0.005
p.Gln155Leu	substitution	155	Gln	Leu	0.808	0.010
p.Gln155Lys	substitution	155	Gln	Lys	1.030	0.007
p.Gln155Me	substitution	155	Gln	Met	0.864	0.005
p.Gln155Phe	substitution	155	Gln	Phe	0.879	0.009
p.Gln155Pro	substitution	155	Gln	Pro	0.797	0.046

p.Gln155Ser	substitution	155	Gln	Ser	1.054	0.011
p.Gln155Ter	nonsense	155	Gln	Ter	0.000	0.028
p.Gln155Thr	substitution	155	Gln	Thr	0.859	0.008
p.Gln155Trp	substitution	155	Gln	Trp	0.859	0.005
p.Gln155Tyr	substitution	155	Gln	Tyr	0.704	0.007
p.Gln155Val	substitution	155	Gln	Val	0.803	0.006
p.Gln180=	synonymous	180	Gln	NA	0.823	0.015
p.Gln180Ala	substitution	180	Gln	Ala	1.151	0.007
p.Gln180Arg	substitution	180	Gln	Arg	1.048	0.009
p.Gln180Asn	substitution	180	Gln	Asn	1.507	0.010
p.Gln180Asp	substitution	180	Gln	Asp	0.623	0.019
p.Gln180Cys	substitution	180	Gln	Cys	1.308	0.009
p.Gln180Gly	substitution	180	Gln	Gly	0.805	0.006
p.Gln180His	substitution	180	Gln	His	0.917	0.033
p.Gln180Ile	substitution	180	Gln	Ile	0.095	0.012
p.Gln180Leu	substitution	180	Gln	Leu	0.914	0.019
p.Gln180Lys	substitution	180	Gln	Lys	1.070	0.009
p.Gln180Met	substitution	180	Gln	Met	0.806	0.011
p.Gln180Phe	substitution	180	Gln	Phe	0.068	0.018
p.Gln180Pro	substitution	180	Gln	Pro	0.750	0.011
p.Gln180Ser	substitution	180	Gln	Ser	0.885	0.079
p.Gln180Ter	nonsense	180	Gln	Ter	0.268	0.019
p.Gln180Thr	substitution	180	Gln	Thr	0.835	0.005
p.Gln180Trp	substitution	180	Gln	Trp	0.435	0.019
p.Gln180Tyr	substitution	180	Gln	Tyr	0.897	0.017
p.Gln180Val	substitution	180	Gln	Val	0.733	0.010
p.Gln181=	synonymous	181	Gln	NA	0.660	0.057
p.Gln181Ala	substitution	181	Gln	Ala	0.589	0.031
p.Gln181Arg	substitution	181	Gln	Arg	1.029	0.018
p.Gln181Asn	substitution	181	Gln	Asn	0.430	0.008
p.Gln181Asp	substitution	181	Gln	Asp	0.614	0.008
p.Gln181Cys	substitution	181	Gln	Cys	0.000	0.047
p.Gln181Glu	substitution	181	Gln	Glu	1.183	0.005
p.Gln181Gly	substitution	181	Gln	Gly	0.880	0.087
p.Gln181His	substitution	181	Gln	His	0.721	0.011
p.Gln181Ile	substitution	181	Gln	Ile	0.595	0.006
p.Gln181Leu	substitution	181	Gln	Leu	0.735	0.014
p.Gln181Lys	substitution	181	Gln	Lys	0.913	0.009
p.Gln181Met	substitution	181	Gln	Met	0.803	0.006
p.Gln181Phe	substitution	181	Gln	Phe	0.802	0.006
p.Gln181Pro	substitution	181	Gln	Pro	0.374	0.038
p.Gln181Ser	substitution	181	Gln	Ser	0.801	0.015
p.Gln181Ter	nonsense	181	Gln	Ter	0.055	0.027

p.Gln181Thr	substitution	181	Gln	Thr	1.016	0.005
p.Gln181Trp	substitution	181	Gln	Trp	0.807	0.007
p.Gln181Tyr	substitution	181	Gln	Tyr	1.164	0.005
p.Gln181Val	substitution	181	Gln	Val	0.442	0.055
p.Gln194=	synonymous	194	Gln	NA	1.117	0.011
p.Gln194Ala	substitution	194	Gln	Ala	1.000	0.019
p.Gln194Arg	substitution	194	Gln	Arg	1.065	0.098
p.Gln194Asn	substitution	194	Gln	Asn	0.458	0.033
p.Gln194Asp	substitution	194	Gln	Asp	0.801	0.005
p.Gln194Cys	substitution	194	Gln	Cys	0.083	0.018
p.Gln194Glu	substitution	194	Gln	Glu	0.389	0.007
p.Gln194Gly	substitution	194	Gln	Gly	0.375	0.012
p.Gln194His	substitution	194	Gln	His	0.350	0.033
p.Gln194Ile	substitution	194	Gln	Ile	0.000	0.251
p.Gln194Leu	substitution	194	Gln	Leu	0.769	0.038
p.Gln194Lys	substitution	194	Gln	Lys	1.001	0.003
p.Gln194Me	substitution	194	Gln	Met	1.360	0.006
p.Gln194Phe	substitution	194	Gln	Phe	0.000	0.036
p.Gln194Pro	substitution	194	Gln	Pro	0.780	0.199
p.Gln194Ser	substitution	194	Gln	Ser	0.120	0.019
p.Gln194Ter	nonsense	194	Gln	Ter	0.193	0.833
p.Gln194Thr	substitution	194	Gln	Thr	0.111	0.042
p.Gln194Tyr	substitution	194	Gln	Tyr	0.264	0.049
p.Gln194Val	substitution	194	Gln	Val	1.123	0.095
p.Gln204=	synonymous	204	Gln	NA	0.830	0.036
p.Gln204Ala	substitution	204	Gln	Ala	0.147	0.023
p.Gln204Arg	substitution	204	Gln	Arg	0.722	0.061
p.Gln204Asn	substitution	204	Gln	Asn	0.000	0.065
p.Gln204Asp	substitution	204	Gln	Asp	0.044	0.013
p.Gln204Cys	substitution	204	Gln	Cys	0.000	0.038
p.Gln204Glu	substitution	204	Gln	Glu	0.745	0.160
p.Gln204Gly	substitution	204	Gln	Gly	0.512	0.193
p.Gln204His	substitution	204	Gln	His	0.987	0.003
p.Gln204Ile	substitution	204	Gln	Ile	0.000	0.035
p.Gln204Leu	substitution	204	Gln	Leu	1.160	0.055
p.Gln204Lys	substitution	204	Gln	Lys	0.586	0.006
p.Gln204Me	substitution	204	Gln	Met	0.000	0.115
p.Gln204Pro	substitution	204	Gln	Pro	0.641	0.010
p.Gln204Ser	substitution	204	Gln	Ser	1.590	0.177
p.Gln204Ter	nonsense	204	Gln	Ter	0.306	0.010
p.Gln204Thr	substitution	204	Gln	Thr	0.000	0.051
p.Gln204Trp	substitution	204	Gln	Trp	0.000	0.008
p.Gln204Tyr	substitution	204	Gln	Tyr	0.629	0.005

p.Gln204Val	substitution	204	Gln	Val		0.995	0.005
p.Gln217=	synonymous	217	Gln	NA		0.386	0.743
p.Gln217Ala	substitution	217	Gln	Ala		0.427	0.099
p.Gln217Arg	substitution	217	Gln	Arg		0.000	0.081
p.Gln217Asn	substitution	217	Gln	Asn		0.000	0.039
p.Gln217Asp	substitution	217	Gln	Asp		0.000	0.264
p.Gln217Cys	substitution	217	Gln	Cys		0.034	0.018
p.Gln217Glu	substitution	217	Gln	Glu		0.119	0.022
p.Gln217Gly	substitution	217	Gln	Gly		0.000	0.023
p.Gln217His	substitution	217	Gln	His		0.394	0.007
p.Gln217Ile	substitution	217	Gln	Ile		0.035	0.008
p.Gln217Leu	substitution	217	Gln	Leu		0.000	0.066
p.Gln217Lys	substitution	217	Gln	Lys		0.215	0.016
p.Gln217Met	substitution	217	Gln	Met		0.000	0.031
p.Gln217Phe	substitution	217	Gln	Phe		0.000	0.050
p.Gln217Pro	substitution	217	Gln	Pro		0.585	0.153
p.Gln217Ser	substitution	217	Gln	Ser		0.712	0.193
p.Gln217Ter	nonsense	217	Gln	Ter		0.124	0.218
p.Gln217Thr	substitution	217	Gln	Thr		0.000	0.011
p.Gln217Trp	substitution	217	Gln	Trp		0.000	0.008
p.Gln217Tyr	substitution	217	Gln	Tyr		0.441	0.097
p.Gln217Val	substitution	217	Gln	Val		0.000	0.017
p.Gln229Ala	substitution	229	Gln	Ala		0.501	0.031
p.Gln229Arg	substitution	229	Gln	Arg		0.949	0.017
p.Gln229Asn	substitution	229	Gln	Asn		1.076	0.002
p.Gln229Asp	substitution	229	Gln	Asp		1.072	0.002
p.Gln229Cys	substitution	229	Gln	Cys		1.199	0.002
p.Gln229Glu	substitution	229	Gln	Glu		1.059	0.002
p.Gln229Gly	substitution	229	Gln	Gly		1.101	0.011
p.Gln229His	substitution	229	Gln	His		0.912	0.002
p.Gln229Ile	substitution	229	Gln	Ile		0.252	0.028
p.Gln229Leu	substitution	229	Gln	Leu		0.707	0.005
p.Gln229Lys	substitution	229	Gln	Lys		0.646	0.003
p.Gln229Met	substitution	229	Gln	Met		0.924	0.002
p.Gln229Phe	substitution	229	Gln	Phe		1.042	0.001
p.Gln229Pro	substitution	229	Gln	Pro		0.502	0.074
p.Gln229Ser	substitution	229	Gln	Ser		0.082	0.033
p.Gln229Ter	nonsense	229	Gln	Ter		0.000	0.289
p.Gln229Thr	substitution	229	Gln	Thr		0.581	0.046
p.Gln229Tyr	substitution	229	Gln	Tyr		1.086	0.002
p.Gln229Val	substitution	229	Gln	Val		0.383	0.019
p.Gln275=	synonymous	275	Gln	NA		0.756	0.312
p.Gln275Ala	substitution	275	Gln	Ala		0.000	0.024

p.Gln275Arg	substitution	275	Gln	Arg		1.085	0.041
p.Gln275Asn	substitution	275	Gln	Asn		0.083	0.007
p.Gln275Asp	substitution	275	Gln	Asp		0.008	0.049
p.Gln275Cys	substitution	275	Gln	Cys		0.000	0.033
p.Gln275Glu	substitution	275	Gln	Glu		0.304	0.074
p.Gln275Gly	substitution	275	Gln	Gly		1.539	0.007
p.Gln275His	substitution	275	Gln	His		1.149	0.077
p.Gln275Ile	substitution	275	Gln	Ile		0.299	0.094
p.Gln275Leu	substitution	275	Gln	Leu		0.000	0.052
p.Gln275Lys	substitution	275	Gln	Lys		0.990	0.030
p.Gln275Met	substitution	275	Gln	Met		1.434	0.004
p.Gln275Phe	substitution	275	Gln	Phe		0.402	0.013
p.Gln275Pro	substitution	275	Gln	Pro		0.601	0.230
p.Gln275Ser	substitution	275	Gln	Ser		0.000	0.009
p.Gln275Ter	nonsense	275	Gln	Ter		0.000	0.073
p.Gln275Thr	substitution	275	Gln	Thr		0.000	0.017
p.Gln275Trp	substitution	275	Gln	Trp		0.000	0.006
p.Gln275Tyr	substitution	275	Gln	Tyr		0.000	0.073
p.Gln275Val	substitution	275	Gln	Val		1.000	0.080
p.Gln292=	synonymous	292	Gln	NA		1.255	0.004
p.Gln292Ala	substitution	292	Gln	Ala		0.621	0.043
p.Gln292Arg	substitution	292	Gln	Arg		0.761	0.024
p.Gln292Asn	substitution	292	Gln	Asn		0.851	0.004
p.Gln292Asp	substitution	292	Gln	Asp		0.791	0.010
p.Gln292Cys	substitution	292	Gln	Cys		0.951	0.004
p.Gln292Glu	substitution	292	Gln	Glu		1.224	0.012
p.Gln292Gly	substitution	292	Gln	Gly		0.653	0.015
p.Gln292His	substitution	292	Gln	His		1.149	0.013
p.Gln292Ile	substitution	292	Gln	Ile		1.447	0.003
p.Gln292Leu	substitution	292	Gln	Leu		1.001	0.009
p.Gln292Lys	substitution	292	Gln	Lys		0.300	0.003
p.Gln292Met	substitution	292	Gln	Met		1.033	0.005
p.Gln292Phe	substitution	292	Gln	Phe		0.705	0.004
p.Gln292Pro	substitution	292	Gln	Pro		0.519	0.005
p.Gln292Ser	substitution	292	Gln	Ser		1.134	0.027
p.Gln292Ter	nonsense	292	Gln	Ter		0.384	0.062
p.Gln292Thr	substitution	292	Gln	Thr		0.815	0.031
p.Gln292Trp	substitution	292	Gln	Trp		0.476	0.005
p.Gln292Tyr	substitution	292	Gln	Tyr		0.392	0.004
p.Gln292Val	substitution	292	Gln	Val		0.552	0.017
p.Gln296=	synonymous	296	Gln	NA		1.067	0.042
p.Gln296Ala	substitution	296	Gln	Ala		0.000	0.010
p.Gln296Arg	substitution	296	Gln	Arg		1.061	0.003

p.Gln296Asn	substitution	296	Gln	Asn	0.941	0.005
p.Gln296Asp	substitution	296	Gln	Asp	0.122	0.009
p.Gln296Cys	substitution	296	Gln	Cys	0.000	0.011
p.Gln296Glu	substitution	296	Gln	Glu	0.865	0.004
p.Gln296Gly	substitution	296	Gln	Gly	0.973	0.004
p.Gln296His	substitution	296	Gln	His	1.110	0.041
p.Gln296Ile	substitution	296	Gln	Ile	0.506	0.007
p.Gln296Leu	substitution	296	Gln	Leu	1.020	0.050
p.Gln296Lys	substitution	296	Gln	Lys	0.957	0.010
p.Gln296Met	substitution	296	Gln	Met	0.000	0.008
p.Gln296Pro	substitution	296	Gln	Pro	0.576	0.040
p.Gln296Ser	substitution	296	Gln	Ser	0.632	0.030
p.Gln296Ter	nonsense	296	Gln	Ter	0.152	0.363
p.Gln296Thr	substitution	296	Gln	Thr	0.367	0.061
p.Gln296Trp	substitution	296	Gln	Trp	0.547	0.010
p.Gln296Tyr	substitution	296	Gln	Tyr	0.814	0.012
p.Gln296Val	substitution	296	Gln	Val	0.971	0.013
p.Gln29=	synonymous	29	Gln	NA	1.197	0.001
p.Gln29Ala	substitution	29	Gln	Ala	0.971	0.012
p.Gln29Arg	substitution	29	Gln	Arg	1.066	0.000
p.Gln29Asn	substitution	29	Gln	Asn	1.353	0.001
p.Gln29Asp	substitution	29	Gln	Asp	0.945	0.001
p.Gln29Cys	substitution	29	Gln	Cys	1.202	0.000
p.Gln29Glu	substitution	29	Gln	Glu	0.511	0.002
p.Gln29Gly	substitution	29	Gln	Gly	1.161	0.001
p.Gln29His	substitution	29	Gln	His	0.818	0.001
p.Gln29Ile	substitution	29	Gln	Ile	0.503	0.001
p.Gln29Leu	substitution	29	Gln	Leu	0.874	0.002
p.Gln29Lys	substitution	29	Gln	Lys	0.951	0.001
p.Gln29Met	substitution	29	Gln	Met	1.026	0.000
p.Gln29Phe	substitution	29	Gln	Phe	0.499	0.003
p.Gln29Pro	substitution	29	Gln	Pro	0.826	0.001
p.Gln29Ser	substitution	29	Gln	Ser	0.980	0.005
p.Gln29Ter	nonsense	29	Gln	Ter	0.000	0.008
p.Gln29Thr	substitution	29	Gln	Thr	0.770	0.005
p.Gln29Trp	substitution	29	Gln	Trp	0.850	0.000
p.Gln29Tyr	substitution	29	Gln	Tyr	0.668	0.001
p.Gln29Val	substitution	29	Gln	Val	0.522	0.001
p.Gln304=	synonymous	304	Gln	NA	1.188	0.004
p.Gln304Ala	substitution	304	Gln	Ala	1.270	0.016
p.Gln304Arg	substitution	304	Gln	Arg	1.020	0.010
p.Gln304Asn	substitution	304	Gln	Asn	1.315	0.059
p.Gln304Asp	substitution	304	Gln	Asp	0.000	0.025

p.Gln304Cys	substitution	304	Gln	Cys	1.038	0.002
p.Gln304Glu	substitution	304	Gln	Glu	1.071	0.001
p.Gln304Gly	substitution	304	Gln	Gly	0.452	0.044
p.Gln304His	substitution	304	Gln	His	0.686	0.027
p.Gln304Ile	substitution	304	Gln	Ile	0.053	0.159
p.Gln304Leu	substitution	304	Gln	Leu	0.269	0.081
p.Gln304Lys	substitution	304	Gln	Lys	1.134	0.002
p.Gln304Met	substitution	304	Gln	Met	0.471	0.006
p.Gln304Pro	substitution	304	Gln	Pro	0.377	0.084
p.Gln304Ser	substitution	304	Gln	Ser	1.094	0.073
p.Gln304Ter	nonsense	304	Gln	Ter	0.014	0.123
p.Gln304Thr	substitution	304	Gln	Thr	0.520	0.008
p.Gln304Trp	substitution	304	Gln	Trp	0.458	0.004
p.Gln304Val	substitution	304	Gln	Val	0.000	0.057
p.Gln314=	synonymous	314	Gln	NA	1.253	0.388
p.Gln314Ala	substitution	314	Gln	Ala	0.171	0.013
p.Gln314Arg	substitution	314	Gln	Arg	0.000	0.020
p.Gln314Asn	substitution	314	Gln	Asn	1.140	0.006
p.Gln314Asp	substitution	314	Gln	Asp	0.000	0.017
p.Gln314Cys	substitution	314	Gln	Cys	1.012	0.002
p.Gln314Glu	substitution	314	Gln	Glu	1.109	0.003
p.Gln314Gly	substitution	314	Gln	Gly	0.000	0.026
p.Gln314His	substitution	314	Gln	His	1.031	0.037
p.Gln314Ile	substitution	314	Gln	Ile	0.499	0.115
p.Gln314Leu	substitution	314	Gln	Leu	1.002	0.032
p.Gln314Met	substitution	314	Gln	Met	0.000	0.162
p.Gln314Phe	substitution	314	Gln	Phe	1.066	0.003
p.Gln314Pro	substitution	314	Gln	Pro	0.854	0.094
p.Gln314Ser	substitution	314	Gln	Ser	0.314	0.421
p.Gln314Ter	nonsense	314	Gln	Ter	0.207	0.022
p.Gln314Thr	substitution	314	Gln	Thr	0.809	0.224
p.Gln314Trp	substitution	314	Gln	Trp	0.810	0.003
p.Gln314Tyr	substitution	314	Gln	Tyr	0.000	0.016
p.Gln314Val	substitution	314	Gln	Val	0.850	0.022
p.Gln328=	synonymous	328	Gln	NA	1.337	0.460
p.Gln328Ala	substitution	328	Gln	Ala	0.462	0.007
p.Gln328Arg	substitution	328	Gln	Arg	0.000	0.794
p.Gln328Asn	substitution	328	Gln	Asn	0.000	0.230
p.Gln328Glu	substitution	328	Gln	Glu	1.044	0.010
p.Gln328Gly	substitution	328	Gln	Gly	0.700	0.003
p.Gln328His	substitution	328	Gln	His	0.913	0.042
p.Gln328Leu	substitution	328	Gln	Leu	0.691	0.075
p.Gln328Lys	substitution	328	Gln	Lys	1.041	0.005

p.Gln328Met	substitution	328	Gln	Met		1.301	0.004
p.Gln328Pro	substitution	328	Gln	Pro		0.808	0.019
p.Gln328Ser	substitution	328	Gln	Ser		1.511	0.142
p.Gln328Ter	nonsense	328	Gln	Ter		0.072	0.016
p.Gln328Trp	substitution	328	Gln	Trp		1.134	0.005
p.Gln328Val	substitution	328	Gln	Val		0.302	0.056
p.Gln332=	synonymous	332	Gln	NA		0.000	1.175
p.Gln332Arg	substitution	332	Gln	Arg		0.857	0.200
p.Gln332Asn	substitution	332	Gln	Asn		0.455	0.040
p.Gln332Asp	substitution	332	Gln	Asp		0.584	0.044
p.Gln332Cys	substitution	332	Gln	Cys		0.000	0.226
p.Gln332Glu	substitution	332	Gln	Glu		0.980	0.078
p.Gln332Gly	substitution	332	Gln	Gly		0.632	0.012
p.Gln332His	substitution	332	Gln	His		1.252	0.021
p.Gln332Ile	substitution	332	Gln	Ile		0.000	0.082
p.Gln332Leu	substitution	332	Gln	Leu		0.662	0.173
p.Gln332Lys	substitution	332	Gln	Lys		1.241	0.002
p.Gln332Met	substitution	332	Gln	Met		1.367	0.002
p.Gln332Phe	substitution	332	Gln	Phe		0.271	0.006
p.Gln332Pro	substitution	332	Gln	Pro		0.362	0.070
p.Gln332Ser	substitution	332	Gln	Ser		1.294	0.068
p.Gln332Ter	nonsense	332	Gln	Ter		0.121	0.320
p.Gln332Thr	substitution	332	Gln	Thr		1.061	0.112
p.Gln332Trp	substitution	332	Gln	Trp		1.056	0.007
p.Gln332Tyr	substitution	332	Gln	Tyr		1.074	0.006
p.Gln34=	synonymous	34	Gln	NA		1.146	0.029
p.Gln34Ala	substitution	34	Gln	Ala		0.623	0.024
p.Gln34Arg	substitution	34	Gln	Arg		0.000	0.001
p.Gln34Asn	substitution	34	Gln	Asn		0.755	0.001
p.Gln34Asp	substitution	34	Gln	Asp		0.086	0.001
p.Gln34Cys	substitution	34	Gln	Cys		0.387	0.001
p.Gln34Glu	substitution	34	Gln	Glu		0.000	0.002
p.Gln34Gly	substitution	34	Gln	Gly		0.567	0.001
p.Gln34His	substitution	34	Gln	His		0.332	0.001
p.Gln34Ile	substitution	34	Gln	Ile		0.424	0.001
p.Gln34Leu	substitution	34	Gln	Leu		0.281	0.002
p.Gln34Lys	substitution	34	Gln	Lys		0.000	0.010
p.Gln34Met	substitution	34	Gln	Met		0.614	0.001
p.Gln34Phe	substitution	34	Gln	Phe		0.183	0.001
p.Gln34Pro	substitution	34	Gln	Pro		0.000	0.063
p.Gln34Ser	substitution	34	Gln	Ser		0.825	0.001
p.Gln34Ter	nonsense	34	Gln	Ter		0.000	0.025
p.Gln34Thr	substitution	34	Gln	Thr		0.334	0.002

p.Gln34Trp	substitution	34	Gln	Trp	0.000	0.002
p.Gln34Tyr	substitution	34	Gln	Tyr	0.079	0.001
p.Gln34Val	substitution	34	Gln	Val	0.419	0.018
p.Gln356Asn	substitution	356	Gln	Asn	1.544	0.011
p.Gln356Cys	substitution	356	Gln	Cys	0.000	0.202
p.Gln356Glu	substitution	356	Gln	Glu	1.074	0.201
p.Gln356His	substitution	356	Gln	His	1.069	0.021
p.Gln356Ile	substitution	356	Gln	Ile	0.000	0.253
p.Gln356Leu	substitution	356	Gln	Leu	0.772	0.142
p.Gln356Lys	substitution	356	Gln	Lys	0.903	0.154
p.Gln356Met	substitution	356	Gln	Met	0.000	0.123
p.Gln356Phe	substitution	356	Gln	Phe	0.083	0.078
p.Gln356Pro	substitution	356	Gln	Pro	1.471	0.003
p.Gln356Ter	nonsense	356	Gln	Ter	0.672	0.016
p.Gln356Thr	substitution	356	Gln	Thr	0.036	0.082
p.Gln356Tyr	substitution	356	Gln	Tyr	0.350	0.053
p.Gln50=	synonymous	50	Gln	NA	1.337	0.014
p.Gln50Arg	substitution	50	Gln	Arg	0.853	0.087
p.Gln50Asp	substitution	50	Gln	Asp	0.044	0.208
p.Gln50Cys	substitution	50	Gln	Cys	0.000	0.174
p.Gln50Glu	substitution	50	Gln	Glu	1.358	0.004
p.Gln50Gly	substitution	50	Gln	Gly	0.375	0.102
p.Gln50His	substitution	50	Gln	His	0.953	0.068
p.Gln50Ile	substitution	50	Gln	Ile	0.681	0.081
p.Gln50Leu	substitution	50	Gln	Leu	1.009	0.013
p.Gln50Lys	substitution	50	Gln	Lys	1.187	0.041
p.Gln50Met	substitution	50	Gln	Met	1.266	0.022
p.Gln50Phe	substitution	50	Gln	Phe	0.661	0.030
p.Gln50Pro	substitution	50	Gln	Pro	1.306	0.072
p.Gln50Ser	substitution	50	Gln	Ser	0.000	0.300
p.Gln50Ter	nonsense	50	Gln	Ter	0.091	0.009
p.Gln50Thr	substitution	50	Gln	Thr	0.785	0.061
p.Gln50Trp	substitution	50	Gln	Trp	0.000	0.159
p.Gln50Tyr	substitution	50	Gln	Tyr	0.986	0.036
p.Gln50Val	substitution	50	Gln	Val	0.000	0.126
p.Glu117=	synonymous	117	Glu	NA	0.814	0.017
p.Glu117Ala	substitution	117	Glu	Ala	0.513	0.077
p.Glu117Arg	substitution	117	Glu	Arg	0.505	0.054
p.Glu117Asn	substitution	117	Glu	Asn	0.390	0.028
p.Glu117Asp	substitution	117	Glu	Asp	0.971	0.036
p.Glu117Cys	substitution	117	Glu	Cys	1.026	0.008
p.Glu117Gln	substitution	117	Glu	Gln	0.764	0.130
p.Glu117Gly	substitution	117	Glu	Gly	0.897	0.012

p.Glu117His	substitution	117	Glu	His	0.876	0.014
p.Glu117Ile	substitution	117	Glu	Ile	1.342	0.012
p.Glu117Leu	substitution	117	Glu	Leu	0.783	0.026
p.Glu117Lys	substitution	117	Glu	Lys	0.817	0.235
p.Glu117Met	substitution	117	Glu	Met	0.449	0.019
p.Glu117Phe	substitution	117	Glu	Phe	0.964	0.011
p.Glu117Pro	substitution	117	Glu	Pro	0.000	0.245
p.Glu117Ser	substitution	117	Glu	Ser	0.404	0.033
p.Glu117Ter	nonsense	117	Glu	Ter	0.074	0.029
p.Glu117Thr	substitution	117	Glu	Thr	0.421	0.018
p.Glu117Trp	substitution	117	Glu	Trp	0.887	0.039
p.Glu117Tyr	substitution	117	Glu	Tyr	0.896	0.015
p.Glu117Val	substitution	117	Glu	Val	1.108	0.033
p.Glu135=	synonymous	135	Glu	NA	0.833	0.005
p.Glu135Ala	substitution	135	Glu	Ala	1.043	0.010
p.Glu135Arg	substitution	135	Glu	Arg	1.039	0.017
p.Glu135Asn	substitution	135	Glu	Asn	0.610	0.006
p.Glu135Asp	substitution	135	Glu	Asp	0.916	0.011
p.Glu135Cys	substitution	135	Glu	Cys	0.717	0.007
p.Glu135Gln	substitution	135	Glu	Gln	1.118	0.043
p.Glu135Gly	substitution	135	Glu	Gly	0.707	0.020
p.Glu135His	substitution	135	Glu	His	0.133	0.022
p.Glu135Ile	substitution	135	Glu	Ile	0.873	0.015
p.Glu135Leu	substitution	135	Glu	Leu	0.992	0.033
p.Glu135Lys	substitution	135	Glu	Lys	0.226	0.022
p.Glu135Met	substitution	135	Glu	Met	1.113	0.007
p.Glu135Phe	substitution	135	Glu	Phe	0.871	0.005
p.Glu135Pro	substitution	135	Glu	Pro	0.865	0.015
p.Glu135Ser	substitution	135	Glu	Ser	0.630	0.013
p.Glu135Ter	nonsense	135	Glu	Ter	0.246	0.053
p.Glu135Thr	substitution	135	Glu	Thr	0.843	0.097
p.Glu135Trp	substitution	135	Glu	Trp	0.882	0.006
p.Glu135Tyr	substitution	135	Glu	Tyr	0.718	0.007
p.Glu135Val	substitution	135	Glu	Val	1.110	0.003
p.Glu139=	synonymous	139	Glu	NA	0.010	3.475
p.Glu139Ala	substitution	139	Glu	Ala	1.061	0.005
p.Glu139Arg	substitution	139	Glu	Arg	0.627	0.020
p.Glu139Asn	substitution	139	Glu	Asn	1.035	0.007
p.Glu139Asp	substitution	139	Glu	Asp	1.043	0.025
p.Glu139Cys	substitution	139	Glu	Cys	1.086	0.005
p.Glu139Gln	substitution	139	Glu	Gln	1.260	0.010
p.Glu139Gly	substitution	139	Glu	Gly	0.816	0.003
p.Glu139His	substitution	139	Glu	His	1.175	0.021

p.Glu139Ile	substitution	139	Glu	Ile	1.174	0.006
p.Glu139Leu	substitution	139	Glu	Leu	1.123	0.009
p.Glu139Lys	substitution	139	Glu	Lys	1.215	0.004
p.Glu139Met	substitution	139	Glu	Met	0.663	0.005
p.Glu139Phe	substitution	139	Glu	Phe	1.091	0.004
p.Glu139Pro	substitution	139	Glu	Pro	0.000	0.097
p.Glu139Ser	substitution	139	Glu	Ser	0.753	0.004
p.Glu139Ter	nonsense	139	Glu	Ter	0.422	0.008
p.Glu139Thr	substitution	139	Glu	Thr	0.614	0.013
p.Glu139Trp	substitution	139	Glu	Trp	1.206	0.005
p.Glu139Tyr	substitution	139	Glu	Tyr	0.982	0.005
p.Glu139Val	substitution	139	Glu	Val	0.621	0.005
p.Glu162=	synonymous	162	Glu	NA	1.175	0.024
p.Glu162Ala	substitution	162	Glu	Ala	0.560	0.022
p.Glu162Arg	substitution	162	Glu	Arg	0.929	0.072
p.Glu162Asn	substitution	162	Glu	Asn	0.346	0.018
p.Glu162Asp	substitution	162	Glu	Asp	0.842	0.026
p.Glu162Cys	substitution	162	Glu	Cys	1.387	0.015
p.Glu162Gln	substitution	162	Glu	Gln	0.502	0.048
p.Glu162Gly	substitution	162	Glu	Gly	0.694	0.163
p.Glu162His	substitution	162	Glu	His	0.394	0.216
p.Glu162Ile	substitution	162	Glu	Ile	0.914	0.010
p.Glu162Leu	substitution	162	Glu	Leu	0.985	0.042
p.Glu162Lys	substitution	162	Glu	Lys	1.117	0.017
p.Glu162Met	substitution	162	Glu	Met	0.887	0.014
p.Glu162Phe	substitution	162	Glu	Phe	1.165	0.009
p.Glu162Pro	substitution	162	Glu	Pro	0.000	0.273
p.Glu162Ser	substitution	162	Glu	Ser	0.409	0.032
p.Glu162Ter	nonsense	162	Glu	Ter	0.540	0.020
p.Glu162Thr	substitution	162	Glu	Thr	0.237	0.308
p.Glu162Trp	substitution	162	Glu	Trp	0.060	0.018
p.Glu162Tyr	substitution	162	Glu	Tyr	1.362	0.007
p.Glu162Val	substitution	162	Glu	Val	1.096	0.009
p.Glu179=	synonymous	179	Glu	NA	1.193	0.020
p.Glu179Ala	substitution	179	Glu	Ala	1.094	0.026
p.Glu179Arg	substitution	179	Glu	Arg	0.722	0.036
p.Glu179Asn	substitution	179	Glu	Asn	0.947	0.007
p.Glu179Asp	substitution	179	Glu	Asp	0.556	0.035
p.Glu179Cys	substitution	179	Glu	Cys	0.811	0.005
p.Glu179Gln	substitution	179	Glu	Gln	0.954	0.007
p.Glu179Gly	substitution	179	Glu	Gly	0.613	0.008
p.Glu179His	substitution	179	Glu	His	0.718	0.009
p.Glu179Ile	substitution	179	Glu	Ile	1.267	0.007

p.Glu179Leu	substitution	179	Glu	Leu	0.705	0.022
p.Glu179Lys	substitution	179	Glu	Lys	0.691	0.031
p.Glu179Met	substitution	179	Glu	Met	0.796	0.008
p.Glu179Phe	substitution	179	Glu	Phe	0.529	0.011
p.Glu179Pro	substitution	179	Glu	Pro	0.459	0.018
p.Glu179Ser	substitution	179	Glu	Ser	0.865	0.054
p.Glu179Ter	nonsense	179	Glu	Ter	0.363	0.014
p.Glu179Thr	substitution	179	Glu	Thr	0.864	0.011
p.Glu179Trp	substitution	179	Glu	Trp	0.758	0.008
p.Glu179Tyr	substitution	179	Glu	Tyr	0.812	0.012
p.Glu179Val	substitution	179	Glu	Val	1.074	0.007
p.Glu182=	synonymous	182	Glu	NA	0.909	0.022
p.Glu182Ala	substitution	182	Glu	Ala	1.006	0.039
p.Glu182Asp	substitution	182	Glu	Asp	0.720	0.082
p.Glu182Gly	substitution	182	Glu	Gly	1.529	0.039
p.Glu182Lys	substitution	182	Glu	Lys	0.509	0.532
p.Glu182Ter	nonsense	182	Glu	Ter	0.333	0.068
p.Glu182Val	substitution	182	Glu	Val	1.440	0.385
p.Glu209=	synonymous	209	Glu	NA	0.116	0.266
p.Glu209Ala	substitution	209	Glu	Ala	0.024	0.145
p.Glu209Arg	substitution	209	Glu	Arg	0.748	0.197
p.Glu209Asn	substitution	209	Glu	Asn	0.000	0.009
p.Glu209Asp	substitution	209	Glu	Asp	0.751	0.048
p.Glu209Cys	substitution	209	Glu	Cys	1.109	0.007
p.Glu209Gln	substitution	209	Glu	Gln	1.269	0.012
p.Glu209Gly	substitution	209	Glu	Gly	0.604	0.097
p.Glu209His	substitution	209	Glu	His	0.766	0.006
p.Glu209Ile	substitution	209	Glu	Ile	0.000	0.137
p.Glu209Leu	substitution	209	Glu	Leu	0.274	0.095
p.Glu209Met	substitution	209	Glu	Met	0.316	0.310
p.Glu209Phe	substitution	209	Glu	Phe	1.316	0.001
p.Glu209Pro	substitution	209	Glu	Pro	1.451	0.019
p.Glu209Ser	substitution	209	Glu	Ser	0.362	0.076
p.Glu209Ter	nonsense	209	Glu	Ter	0.113	0.017
p.Glu209Thr	substitution	209	Glu	Thr	0.063	0.056
p.Glu209Trp	substitution	209	Glu	Trp	0.000	0.010
p.Glu209Tyr	substitution	209	Glu	Tyr	1.108	0.063
p.Glu209Val	substitution	209	Glu	Val	0.064	0.039
p.Glu210=	synonymous	210	Glu	NA	0.713	0.268
p.Glu210Ala	substitution	210	Glu	Ala	0.587	0.058
p.Glu210Arg	substitution	210	Glu	Arg	1.004	0.029
p.Glu210Asn	substitution	210	Glu	Asn	0.994	0.013
p.Glu210Asp	substitution	210	Glu	Asp	0.702	0.106

p.Glu210Cys	substitution	210	Glu	Cys	1.325	0.002
p.Glu210Gln	substitution	210	Glu	Gln	0.335	0.041
p.Glu210Gly	substitution	210	Glu	Gly	0.160	0.079
p.Glu210His	substitution	210	Glu	His	0.000	0.062
p.Glu210Ile	substitution	210	Glu	Ile	1.627	0.007
p.Glu210Leu	substitution	210	Glu	Leu	1.064	0.015
p.Glu210Lys	substitution	210	Glu	Lys	0.000	0.011
p.Glu210Met	substitution	210	Glu	Met	0.000	0.015
p.Glu210Pro	substitution	210	Glu	Pro	0.000	0.015
p.Glu210Ser	substitution	210	Glu	Ser	0.264	0.027
p.Glu210Ter	nonsense	210	Glu	Ter	0.284	0.106
p.Glu210Thr	substitution	210	Glu	Thr	0.599	0.124
p.Glu210Trp	substitution	210	Glu	Trp	1.317	0.005
p.Glu210Tyr	substitution	210	Glu	Tyr	0.331	0.074
p.Glu210Val	substitution	210	Glu	Val	0.909	0.004
p.Glu223=	synonymous	223	Glu	NA	0.504	0.525
p.Glu223Ala	substitution	223	Glu	Ala	0.072	0.130
p.Glu223Arg	substitution	223	Glu	Arg	0.000	0.045
p.Glu223Asn	substitution	223	Glu	Asn	0.870	0.005
p.Glu223Asp	substitution	223	Glu	Asp	0.595	0.419
p.Glu223Cys	substitution	223	Glu	Cys	1.209	0.006
p.Glu223Gln	substitution	223	Glu	Gln	0.054	0.298
p.Glu223Gly	substitution	223	Glu	Gly	0.568	0.103
p.Glu223His	substitution	223	Glu	His	0.463	0.078
p.Glu223Ile	substitution	223	Glu	Ile	0.053	0.039
p.Glu223Leu	substitution	223	Glu	Leu	0.832	0.107
p.Glu223Lys	substitution	223	Glu	Lys	0.000	0.224
p.Glu223Met	substitution	223	Glu	Met	0.000	0.194
p.Glu223Phe	substitution	223	Glu	Phe	0.000	0.074
p.Glu223Pro	substitution	223	Glu	Pro	0.138	0.044
p.Glu223Ser	substitution	223	Glu	Ser	0.067	0.041
p.Glu223Ter	nonsense	223	Glu	Ter	0.452	0.194
p.Glu223Thr	substitution	223	Glu	Thr	0.568	0.138
p.Glu223Trp	substitution	223	Glu	Trp	0.000	0.013
p.Glu223Tyr	substitution	223	Glu	Tyr	0.000	0.013
p.Glu223Val	substitution	223	Glu	Val	0.280	0.181
p.Glu242Ala	substitution	242	Glu	Ala	1.100	0.026
p.Glu242Arg	substitution	242	Glu	Arg	0.728	0.125
p.Glu242Asn	substitution	242	Glu	Asn	0.000	0.182
p.Glu242Asp	substitution	242	Glu	Asp	0.958	0.003
p.Glu242Cys	substitution	242	Glu	Cys	0.009	0.079
p.Glu242Gln	substitution	242	Glu	Gln	0.794	0.004
p.Glu242Gly	substitution	242	Glu	Gly	0.025	0.024

p.Glu242His	substitution	242	Glu	His	1.335	0.003
p.Glu242Ile	substitution	242	Glu	Ile	0.000	0.011
p.Glu242Leu	substitution	242	Glu	Leu	0.856	0.330
p.Glu242Lys	substitution	242	Glu	Lys	0.834	0.004
p.Glu242Met	substitution	242	Glu	Met	0.302	0.005
p.Glu242Phe	substitution	242	Glu	Phe	1.484	0.008
p.Glu242Pro	substitution	242	Glu	Pro	1.076	0.223
p.Glu242Ser	substitution	242	Glu	Ser	0.134	0.026
p.Glu242Ter	nonsense	242	Glu	Ter	0.273	0.005
p.Glu242Thr	substitution	242	Glu	Thr	0.607	0.164
p.Glu242Tyr	substitution	242	Glu	Tyr	0.000	0.027
p.Glu242Val	substitution	242	Glu	Val	1.107	0.005
p.Glu250=	synonymous	250	Glu	NA	1.296	0.003
p.Glu250Ala	substitution	250	Glu	Ala	0.000	0.202
p.Glu250Arg	substitution	250	Glu	Arg	0.000	0.017
p.Glu250Asp	substitution	250	Glu	Asp	1.342	0.306
p.Glu250Cys	substitution	250	Glu	Cys	0.000	0.019
p.Glu250Gln	substitution	250	Glu	Gln	0.740	0.017
p.Glu250Gly	substitution	250	Glu	Gly	0.000	0.013
p.Glu250His	substitution	250	Glu	His	0.000	0.018
p.Glu250Ile	substitution	250	Glu	Ile	0.000	0.040
p.Glu250Leu	substitution	250	Glu	Leu	0.000	0.040
p.Glu250Lys	substitution	250	Glu	Lys	0.018	0.204
p.Glu250Met	substitution	250	Glu	Met	0.000	0.032
p.Glu250Phe	substitution	250	Glu	Phe	0.000	0.086
p.Glu250Pro	substitution	250	Glu	Pro	0.000	0.031
p.Glu250Ser	substitution	250	Glu	Ser	0.000	0.023
p.Glu250Ter	nonsense	250	Glu	Ter	0.000	0.066
p.Glu250Thr	substitution	250	Glu	Thr	0.068	0.069
p.Glu250Trp	substitution	250	Glu	Trp	0.000	0.008
p.Glu250Tyr	substitution	250	Glu	Tyr	0.000	0.042
p.Glu250Val	substitution	250	Glu	Val	0.000	0.064
p.Glu258=	synonymous	258	Glu	NA	0.703	0.007
p.Glu258Ala	substitution	258	Glu	Ala	0.119	0.069
p.Glu258Arg	substitution	258	Glu	Arg	0.000	0.146
p.Glu258Asn	substitution	258	Glu	Asn	0.000	0.023
p.Glu258Asp	substitution	258	Glu	Asp	1.263	0.360
p.Glu258Cys	substitution	258	Glu	Cys	1.326	0.002
p.Glu258Gly	substitution	258	Glu	Gly	1.303	0.048
p.Glu258Ile	substitution	258	Glu	Ile	1.007	0.009
p.Glu258Leu	substitution	258	Glu	Leu	0.027	0.072
p.Glu258Lys	substitution	258	Glu	Lys	1.169	0.124
p.Glu258Met	substitution	258	Glu	Met	0.007	0.010

p.Glu258Phe	substitution	258	Glu	Phe	0.475	0.013
p.Glu258Pro	substitution	258	Glu	Pro	0.391	0.038
p.Glu258Ser	substitution	258	Glu	Ser	0.580	0.045
p.Glu258Ter	nonsense	258	Glu	Ter	0.313	0.081
p.Glu258Thr	substitution	258	Glu	Thr	0.000	0.033
p.Glu258Trp	substitution	258	Glu	Trp	0.061	0.232
p.Glu258Tyr	substitution	258	Glu	Tyr	1.053	0.005
p.Glu258Val	substitution	258	Glu	Val	1.326	0.229
p.Glu293=	synonymous	293	Glu	NA	1.412	0.161
p.Glu293Ala	substitution	293	Glu	Ala	0.858	0.007
p.Glu293Arg	substitution	293	Glu	Arg	0.570	0.021
p.Glu293Asn	substitution	293	Glu	Asn	0.688	0.004
p.Glu293Asp	substitution	293	Glu	Asp	1.116	0.014
p.Glu293Cys	substitution	293	Glu	Cys	0.597	0.003
p.Glu293Gln	substitution	293	Glu	Gln	1.140	0.009
p.Glu293Gly	substitution	293	Glu	Gly	1.099	0.004
p.Glu293His	substitution	293	Glu	His	1.059	0.005
p.Glu293Ile	substitution	293	Glu	Ile	0.728	0.004
p.Glu293Leu	substitution	293	Glu	Leu	0.445	0.017
p.Glu293Lys	substitution	293	Glu	Lys	0.889	0.005
p.Glu293Phe	substitution	293	Glu	Phe	0.588	0.004
p.Glu293Pro	substitution	293	Glu	Pro	0.000	0.015
p.Glu293Ser	substitution	293	Glu	Ser	0.942	0.008
p.Glu293Ter	nonsense	293	Glu	Ter	0.203	0.011
p.Glu293Thr	substitution	293	Glu	Thr	0.079	0.037
p.Glu293Trp	substitution	293	Glu	Trp	1.136	0.004
p.Glu293Tyr	substitution	293	Glu	Tyr	0.540	0.005
p.Glu293Val	substitution	293	Glu	Val	0.535	0.005
p.Glu306=	synonymous	306	Glu	NA	1.123	0.003
p.Glu306Ala	substitution	306	Glu	Ala	1.235	0.005
p.Glu306Arg	substitution	306	Glu	Arg	0.951	0.017
p.Glu306Asn	substitution	306	Glu	Asn	1.459	0.002
p.Glu306Asp	substitution	306	Glu	Asp	0.524	0.003
p.Glu306Cys	substitution	306	Glu	Cys	0.135	0.015
p.Glu306Gln	substitution	306	Glu	Gln	1.006	0.003
p.Glu306Gly	substitution	306	Glu	Gly	0.498	0.057
p.Glu306His	substitution	306	Glu	His	0.953	0.005
p.Glu306Ile	substitution	306	Glu	Ile	0.912	0.006
p.Glu306Leu	substitution	306	Glu	Leu	1.114	0.022
p.Glu306Lys	substitution	306	Glu	Lys	1.330	0.015
p.Glu306Met	substitution	306	Glu	Met	0.172	0.008
p.Glu306Phe	substitution	306	Glu	Phe	1.441	0.003
p.Glu306Pro	substitution	306	Glu	Pro	0.524	0.066

p.Glu306Ser	substitution	306	Glu	Ser		0.844	0.165
p.Glu306Ter	nonsense	306	Glu	Ter		0.461	0.063
p.Glu306Thr	substitution	306	Glu	Thr		0.033	0.009
p.Glu306Trp	substitution	306	Glu	Trp		0.903	0.003
p.Glu306Tyr	substitution	306	Glu	Tyr		0.307	0.013
p.Glu306Val	substitution	306	Glu	Val		1.133	0.006
p.Glu310Ala	substitution	310	Glu	Ala		1.064	0.120
p.Glu310Arg	substitution	310	Glu	Arg		1.020	0.044
p.Glu310Asn	substitution	310	Glu	Asn		0.000	0.047
p.Glu310Asp	substitution	310	Glu	Asp		0.408	0.062
p.Glu310Gln	substitution	310	Glu	Gln		1.264	0.003
p.Glu310Gly	substitution	310	Glu	Gly		0.959	0.057
p.Glu310His	substitution	310	Glu	His		1.466	0.001
p.Glu310Ile	substitution	310	Glu	Ile		0.000	0.018
p.Glu310Leu	substitution	310	Glu	Leu		0.775	0.056
p.Glu310Lys	substitution	310	Glu	Lys		0.697	0.005
p.Glu310Met	substitution	310	Glu	Met		1.358	0.008
p.Glu310Phe	substitution	310	Glu	Phe		0.000	0.044
p.Glu310Pro	substitution	310	Glu	Pro		0.066	0.030
p.Glu310Ser	substitution	310	Glu	Ser		0.254	0.207
p.Glu310Ter	nonsense	310	Glu	Ter		0.425	0.004
p.Glu310Thr	substitution	310	Glu	Thr		0.000	0.316
p.Glu310Trp	substitution	310	Glu	Trp		0.087	0.009
p.Glu310Tyr	substitution	310	Glu	Tyr		0.000	0.020
p.Glu310Val	substitution	310	Glu	Val		0.714	1.008
p.Glu52=	synonymous	52	Glu	NA		0.525	0.029
p.Glu52Ala	substitution	52	Glu	Ala		1.080	0.037
p.Glu52Arg	substitution	52	Glu	Arg		0.989	0.044
p.Glu52Asn	substitution	52	Glu	Asn		0.184	0.014
p.Glu52Asp	substitution	52	Glu	Asp		0.904	0.004
p.Glu52Cys	substitution	52	Glu	Cys		1.045	0.011
p.Glu52Gln	substitution	52	Glu	Gln		0.000	0.293
p.Glu52Gly	substitution	52	Glu	Gly		0.921	0.012
p.Glu52His	substitution	52	Glu	His		0.267	0.058
p.Glu52Ile	substitution	52	Glu	Ile		0.992	0.008
p.Glu52Leu	substitution	52	Glu	Leu		0.259	0.048
p.Glu52Lys	substitution	52	Glu	Lys		0.342	0.026
p.Glu52Met	substitution	52	Glu	Met		0.000	0.024
p.Glu52Phe	substitution	52	Glu	Phe		0.892	0.025
p.Glu52Pro	substitution	52	Glu	Pro		0.561	0.071
p.Glu52Ser	substitution	52	Glu	Ser		0.910	0.101
p.Glu52Ter	nonsense	52	Glu	Ter		0.000	0.070
p.Glu52Thr	substitution	52	Glu	Thr		0.878	0.034

p.Glu52Trp	substitution	52	Glu	Trp		0.228	0.015
p.Glu52Tyr	substitution	52	Glu	Tyr		0.803	0.020
p.Glu52Val	substitution	52	Glu	Val		0.946	0.051
p.Glu73=	synonymous	73	Glu	NA		0.359	1.589
p.Glu73Ala	substitution	73	Glu	Ala		0.733	0.005
p.Glu73Arg	substitution	73	Glu	Arg		0.857	0.006
p.Glu73Asn	substitution	73	Glu	Asn		0.498	0.016
p.Glu73Asp	substitution	73	Glu	Asp		1.310	1.035
p.Glu73Cys	substitution	73	Glu	Cys		0.549	0.020
p.Glu73Gln	substitution	73	Glu	Gln		0.719	0.017
p.Glu73Gly	substitution	73	Glu	Gly		0.469	0.009
p.Glu73His	substitution	73	Glu	His		1.290	0.006
p.Glu73Ile	substitution	73	Glu	Ile		0.517	0.015
p.Glu73Leu	substitution	73	Glu	Leu		0.489	0.143
p.Glu73Lys	substitution	73	Glu	Lys		0.780	0.011
p.Glu73Met	substitution	73	Glu	Met		0.651	0.009
p.Glu73Phe	substitution	73	Glu	Phe		0.486	0.007
p.Glu73Pro	substitution	73	Glu	Pro		0.597	0.013
p.Glu73Ser	substitution	73	Glu	Ser		0.909	0.018
p.Glu73Ter	nonsense	73	Glu	Ter		0.236	0.009
p.Glu73Thr	substitution	73	Glu	Thr		1.172	0.004
p.Glu73Trp	substitution	73	Glu	Trp		0.186	0.010
p.Glu73Tyr	substitution	73	Glu	Tyr		0.819	0.005
p.Glu73Val	substitution	73	Glu	Val		0.727	0.018
p.Glu80=	synonymous	80	Glu	NA		0.631	0.017
p.Glu80Ala	substitution	80	Glu	Ala		0.869	0.002
p.Glu80Arg	substitution	80	Glu	Arg		0.730	0.004
p.Glu80Asn	substitution	80	Glu	Asn		0.438	0.006
p.Glu80Asp	substitution	80	Glu	Asp		1.128	0.012
p.Glu80Cys	substitution	80	Glu	Cys		0.893	0.002
p.Glu80Gln	substitution	80	Glu	Gln		0.772	0.003
p.Glu80Gly	substitution	80	Glu	Gly		0.647	0.002
p.Glu80His	substitution	80	Glu	His		0.568	0.018
p.Glu80Ile	substitution	80	Glu	Ile		0.703	0.005
p.Glu80Leu	substitution	80	Glu	Leu		0.490	0.009
p.Glu80Lys	substitution	80	Glu	Lys		0.739	0.004
p.Glu80Met	substitution	80	Glu	Met		0.816	0.003
p.Glu80Phe	substitution	80	Glu	Phe		0.000	0.043
p.Glu80Pro	substitution	80	Glu	Pro		0.625	0.005
p.Glu80Ser	substitution	80	Glu	Ser		0.757	0.014
p.Glu80Ter	nonsense	80	Glu	Ter		0.015	0.154
p.Glu80Thr	substitution	80	Glu	Thr		0.898	0.025
p.Glu80Trp	substitution	80	Glu	Trp		0.583	0.003

p.Glu80Tyr	substitution	80	Glu	Tyr	0.000	0.009
p.Glu80Val	substitution	80	Glu	Val	0.585	0.002
p.Glu82=	synonymous	82	Glu	NA	0.895	0.016
p.Glu82Ala	substitution	82	Glu	Ala	0.931	0.228
p.Glu82Arg	substitution	82	Glu	Arg	0.624	0.023
p.Glu82Asn	substitution	82	Glu	Asn	0.804	0.018
p.Glu82Asp	substitution	82	Glu	Asp	0.818	0.030
p.Glu82Cys	substitution	82	Glu	Cys	0.479	0.019
p.Glu82Gln	substitution	82	Glu	Gln	0.461	0.034
p.Glu82Gly	substitution	82	Glu	Gly	0.132	0.077
p.Glu82His	substitution	82	Glu	His	0.060	0.022
p.Glu82Ile	substitution	82	Glu	Ile	0.000	0.019
p.Glu82Leu	substitution	82	Glu	Leu	0.603	0.032
p.Glu82Lys	substitution	82	Glu	Lys	0.455	0.017
p.Glu82Met	substitution	82	Glu	Met	0.000	0.022
p.Glu82Phe	substitution	82	Glu	Phe	1.792	0.006
p.Glu82Pro	substitution	82	Glu	Pro	0.000	0.033
p.Glu82Ser	substitution	82	Glu	Ser	0.821	0.044
p.Glu82Ter	nonsense	82	Glu	Ter	0.298	0.077
p.Glu82Thr	substitution	82	Glu	Thr	0.602	0.245
p.Glu82Trp	substitution	82	Glu	Trp	0.413	0.013
p.Glu82Tyr	substitution	82	Glu	Tyr	0.151	0.016
p.Glu82Val	substitution	82	Glu	Val	0.417	0.097
p.Glu86=	synonymous	86	Glu	NA	1.415	0.020
p.Glu86Ala	substitution	86	Glu	Ala	0.874	0.020
p.Glu86Arg	substitution	86	Glu	Arg	1.022	0.012
p.Glu86Asn	substitution	86	Glu	Asn	0.426	0.012
p.Glu86Asp	substitution	86	Glu	Asp	0.969	0.008
p.Glu86Gln	substitution	86	Glu	Gln	0.916	0.009
p.Glu86Gly	substitution	86	Glu	Gly	0.613	0.010
p.Glu86His	substitution	86	Glu	His	1.039	0.008
p.Glu86Ile	substitution	86	Glu	Ile	1.012	0.006
p.Glu86Leu	substitution	86	Glu	Leu	0.806	0.032
p.Glu86Lys	substitution	86	Glu	Lys	1.129	0.014
p.Glu86Met	substitution	86	Glu	Met	0.786	0.016
p.Glu86Phe	substitution	86	Glu	Phe	0.694	0.012
p.Glu86Pro	substitution	86	Glu	Pro	0.000	0.057
p.Glu86Ser	substitution	86	Glu	Ser	0.884	0.032
p.Glu86Ter	nonsense	86	Glu	Ter	0.484	0.008
p.Glu86Thr	substitution	86	Glu	Thr	0.806	0.022
p.Glu86Trp	substitution	86	Glu	Trp	0.187	0.018
p.Glu86Tyr	substitution	86	Glu	Tyr	1.545	0.008
p.Glu86Val	substitution	86	Glu	Val	0.737	0.020

p.Glu89=	synonymous	89	Glu	NA		0.781	0.004
p.Glu89Ala	substitution	89	Glu	Ala		0.833	0.006
p.Glu89Arg	substitution	89	Glu	Arg		1.042	0.006
p.Glu89Asn	substitution	89	Glu	Asn		1.002	0.002
p.Glu89Asp	substitution	89	Glu	Asp		0.945	0.019
p.Glu89Cys	substitution	89	Glu	Cys		0.666	0.003
p.Glu89Gln	substitution	89	Glu	Gln		1.124	0.054
p.Glu89Gly	substitution	89	Glu	Gly		0.679	0.014
p.Glu89His	substitution	89	Glu	His		0.690	0.004
p.Glu89Ile	substitution	89	Glu	Ile		1.033	0.009
p.Glu89Leu	substitution	89	Glu	Leu		0.827	0.007
p.Glu89Lys	substitution	89	Glu	Lys		0.738	0.007
p.Glu89Met	substitution	89	Glu	Met		0.688	0.006
p.Glu89Phe	substitution	89	Glu	Phe		0.992	0.003
p.Glu89Pro	substitution	89	Glu	Pro		0.000	0.027
p.Glu89Ser	substitution	89	Glu	Ser		1.026	0.009
p.Glu89Ter	nonsense	89	Glu	Ter		0.332	0.043
p.Glu89Thr	substitution	89	Glu	Thr		0.829	0.007
p.Glu89Trp	substitution	89	Glu	Trp		0.631	0.004
p.Glu89Tyr	substitution	89	Glu	Tyr		1.020	0.003
p.Glu89Val	substitution	89	Glu	Val		0.906	0.019
p.Gly107=	synonymous	107	Gly	NA		0.958	0.004
p.Gly107Ala	substitution	107	Gly	Ala		0.754	0.027
p.Gly107Arg	substitution	107	Gly	Arg		0.754	0.029
p.Gly107Asn	substitution	107	Gly	Asn		0.563	0.021
p.Gly107Asp	substitution	107	Gly	Asp		0.947	0.062
p.Gly107Cys	substitution	107	Gly	Cys		0.962	0.008
p.Gly107Gln	substitution	107	Gly	Gln		1.120	0.511
p.Gly107Glu	substitution	107	Gly	Glu		0.808	0.021
p.Gly107His	substitution	107	Gly	His		0.684	0.052
p.Gly107Ile	substitution	107	Gly	Ile		0.000	0.323
p.Gly107Leu	substitution	107	Gly	Leu		0.736	0.069
p.Gly107Lys	substitution	107	Gly	Lys		0.571	0.016
p.Gly107Met	substitution	107	Gly	Met		1.064	0.015
p.Gly107Phe	substitution	107	Gly	Phe		1.048	0.008
p.Gly107Pro	substitution	107	Gly	Pro		0.031	0.245
p.Gly107Ser	substitution	107	Gly	Ser		0.820	0.020
p.Gly107Ter	nonsense	107	Gly	Ter		0.000	0.021
p.Gly107Thr	substitution	107	Gly	Thr		0.000	0.165
p.Gly107Trp	substitution	107	Gly	Trp		1.360	0.006
p.Gly107Tyr	substitution	107	Gly	Tyr		0.000	0.061
p.Gly107Val	substitution	107	Gly	Val		0.726	0.012
p.Gly111=	synonymous	111	Gly	NA		0.800	0.016

p.Gly111Ala	substitution	111	Gly	Ala	0.364	0.033
p.Gly111Arg	substitution	111	Gly	Arg	0.015	0.040
p.Gly111Asn	substitution	111	Gly	Asn	0.000	0.011
p.Gly111Asp	substitution	111	Gly	Asp	0.011	0.012
p.Gly111Cys	substitution	111	Gly	Cys	0.000	0.008
p.Gly111Gln	substitution	111	Gly	Gln	0.000	0.042
p.Gly111Glu	substitution	111	Gly	Glu	0.064	0.008
p.Gly111His	substitution	111	Gly	His	0.000	0.022
p.Gly111Ile	substitution	111	Gly	Ile	0.000	0.054
p.Gly111Leu	substitution	111	Gly	Leu	0.000	0.017
p.Gly111Lys	substitution	111	Gly	Lys	0.182	0.063
p.Gly111Met	substitution	111	Gly	Met	0.000	0.015
p.Gly111Phe	substitution	111	Gly	Phe	0.087	0.015
p.Gly111Pro	substitution	111	Gly	Pro	0.000	0.032
p.Gly111Ser	substitution	111	Gly	Ser	0.550	0.006
p.Gly111Ter	nonsense	111	Gly	Ter	0.119	0.024
p.Gly111Thr	substitution	111	Gly	Thr	0.037	0.014
p.Gly111Trp	substitution	111	Gly	Trp	0.000	0.009
p.Gly111Tyr	substitution	111	Gly	Tyr	0.000	0.024
p.Gly111Val	substitution	111	Gly	Val	0.179	0.007
p.Gly131=	synonymous	131	Gly	NA	0.922	0.038
p.Gly131Ala	substitution	131	Gly	Ala	0.956	0.012
p.Gly131Arg	substitution	131	Gly	Arg	0.821	0.006
p.Gly131Asn	substitution	131	Gly	Asn	0.750	0.008
p.Gly131Asp	substitution	131	Gly	Asp	0.786	0.007
p.Gly131Cys	substitution	131	Gly	Cys	0.505	0.010
p.Gly131Gln	substitution	131	Gly	Gln	1.092	0.009
p.Gly131Glu	substitution	131	Gly	Glu	0.725	0.012
p.Gly131His	substitution	131	Gly	His	1.061	0.008
p.Gly131Ile	substitution	131	Gly	Ile	0.229	0.007
p.Gly131Leu	substitution	131	Gly	Leu	0.634	0.007
p.Gly131Lys	substitution	131	Gly	Lys	0.667	0.014
p.Gly131Phe	substitution	131	Gly	Phe	0.805	0.006
p.Gly131Pro	substitution	131	Gly	Pro	0.416	0.021
p.Gly131Ser	substitution	131	Gly	Ser	0.877	0.011
p.Gly131Ter	nonsense	131	Gly	Ter	0.000	0.011
p.Gly131Thr	substitution	131	Gly	Thr	0.674	0.055
p.Gly131Trp	substitution	131	Gly	Trp	0.932	0.003
p.Gly131Tyr	substitution	131	Gly	Tyr	0.846	0.008
p.Gly131Val	substitution	131	Gly	Val	0.932	0.006
p.Gly144=	synonymous	144	Gly	NA	0.984	0.006
p.Gly144Ala	substitution	144	Gly	Ala	0.889	0.043
p.Gly144Arg	substitution	144	Gly	Arg	0.550	0.029

p.Gly144Asn	substitution	144	Gly	Asn	0.000	0.043
p.Gly144Asp	substitution	144	Gly	Asp	0.109	0.050
p.Gly144Cys	substitution	144	Gly	Cys	0.640	0.017
p.Gly144Gln	substitution	144	Gly	Gln	0.000	0.066
p.Gly144Glu	substitution	144	Gly	Glu	0.259	0.012
p.Gly144His	substitution	144	Gly	His	0.000	0.044
p.Gly144Ile	substitution	144	Gly	Ile	0.000	0.027
p.Gly144Leu	substitution	144	Gly	Leu	0.398	0.026
p.Gly144Lys	substitution	144	Gly	Lys	0.679	0.015
p.Gly144Met	substitution	144	Gly	Met	0.931	0.008
p.Gly144Phe	substitution	144	Gly	Phe	0.000	0.019
p.Gly144Pro	substitution	144	Gly	Pro	0.000	0.103
p.Gly144Ser	substitution	144	Gly	Ser	0.408	0.009
p.Gly144Ter	nonsense	144	Gly	Ter	0.402	0.096
p.Gly144Thr	substitution	144	Gly	Thr	0.762	0.016
p.Gly144Trp	substitution	144	Gly	Trp	0.101	0.006
p.Gly144Val	substitution	144	Gly	Val	0.346	0.012
p.Gly168=	synonymous	168	Gly	NA	0.902	0.024
p.Gly168Ala	substitution	168	Gly	Ala	0.000	0.037
p.Gly168Arg	substitution	168	Gly	Arg	0.000	0.022
p.Gly168Asn	substitution	168	Gly	Asn	0.137	0.016
p.Gly168Asp	substitution	168	Gly	Asp	0.000	0.009
p.Gly168Cys	substitution	168	Gly	Cys	0.000	0.012
p.Gly168Gln	substitution	168	Gly	Gln	0.000	0.026
p.Gly168Glu	substitution	168	Gly	Glu	0.000	0.036
p.Gly168His	substitution	168	Gly	His	0.024	0.010
p.Gly168Ile	substitution	168	Gly	Ile	0.000	0.042
p.Gly168Leu	substitution	168	Gly	Leu	0.110	0.010
p.Gly168Lys	substitution	168	Gly	Lys	0.000	0.055
p.Gly168Met	substitution	168	Gly	Met	0.000	0.024
p.Gly168Phe	substitution	168	Gly	Phe	0.000	0.015
p.Gly168Pro	substitution	168	Gly	Pro	0.000	0.031
p.Gly168Ser	substitution	168	Gly	Ser	0.000	0.016
p.Gly168Ter	nonsense	168	Gly	Ter	0.171	0.049
p.Gly168Thr	substitution	168	Gly	Thr	0.000	0.052
p.Gly168Trp	substitution	168	Gly	Trp	0.000	0.008
p.Gly168Tyr	substitution	168	Gly	Tyr	0.000	0.008
p.Gly168Val	substitution	168	Gly	Val	0.000	0.055
p.Gly192=	synonymous	192	Gly	NA	1.066	0.016
p.Gly192Ala	substitution	192	Gly	Ala	0.377	0.023
p.Gly192Arg	substitution	192	Gly	Arg	0.000	0.116
p.Gly192Asn	substitution	192	Gly	Asn	0.000	0.059
p.Gly192Asp	substitution	192	Gly	Asp	0.117	0.044

p.Gly192Cys	substitution	192	Gly	Cys	0.885	0.247
p.Gly192Gln	substitution	192	Gly	Gln	0.000	0.155
p.Gly192Glu	substitution	192	Gly	Glu	0.284	0.171
p.Gly192His	substitution	192	Gly	His	0.000	0.126
p.Gly192Leu	substitution	192	Gly	Leu	0.000	0.848
p.Gly192Met	substitution	192	Gly	Met	0.311	0.207
p.Gly192Phe	substitution	192	Gly	Phe	0.000	0.168
p.Gly192Ser	substitution	192	Gly	Ser	0.947	0.558
p.Gly192Ter	nonsense	192	Gly	Ter	0.000	0.223
p.Gly192Thr	substitution	192	Gly	Thr	0.150	0.022
p.Gly192Trp	substitution	192	Gly	Trp	0.000	0.037
p.Gly192Val	substitution	192	Gly	Val	0.507	0.088
p.Gly197=	synonymous	197	Gly	NA	0.518	0.003
p.Gly197Ala	substitution	197	Gly	Ala	0.217	0.031
p.Gly197Arg	substitution	197	Gly	Arg	0.775	0.061
p.Gly197Asn	substitution	197	Gly	Asn	0.176	0.014
p.Gly197Asp	substitution	197	Gly	Asp	0.025	0.055
p.Gly197Cys	substitution	197	Gly	Cys	1.134	0.018
p.Gly197Gln	substitution	197	Gly	Gln	0.000	0.046
p.Gly197Glu	substitution	197	Gly	Glu	0.258	0.003
p.Gly197His	substitution	197	Gly	His	0.000	0.006
p.Gly197Ile	substitution	197	Gly	Ile	0.000	0.107
p.Gly197Leu	substitution	197	Gly	Leu	0.175	0.062
p.Gly197Lys	substitution	197	Gly	Lys	0.071	0.034
p.Gly197Met	substitution	197	Gly	Met	0.000	0.029
p.Gly197Phe	substitution	197	Gly	Phe	0.526	0.021
p.Gly197Pro	substitution	197	Gly	Pro	0.145	0.142
p.Gly197Ser	substitution	197	Gly	Ser	0.983	0.100
p.Gly197Ter	nonsense	197	Gly	Ter	0.000	0.014
p.Gly197Thr	substitution	197	Gly	Thr	0.099	0.184
p.Gly197Trp	substitution	197	Gly	Trp	0.229	0.004
p.Gly197Tyr	substitution	197	Gly	Tyr	0.504	0.032
p.Gly197Val	substitution	197	Gly	Val	0.441	0.021
p.Gly203=	synonymous	203	Gly	NA	1.318	0.019
p.Gly203Ala	substitution	203	Gly	Ala	1.023	0.192
p.Gly203Arg	substitution	203	Gly	Arg	0.168	0.174
p.Gly203Cys	substitution	203	Gly	Cys	1.056	0.096
p.Gly203Glu	substitution	203	Gly	Glu	0.276	0.671
p.Gly203His	substitution	203	Gly	His	0.000	0.178
p.Gly203Ile	substitution	203	Gly	Ile	0.000	0.019
p.Gly203Leu	substitution	203	Gly	Leu	0.000	0.053
p.Gly203Met	substitution	203	Gly	Met	0.000	0.030
p.Gly203Phe	substitution	203	Gly	Phe	0.000	0.031

p.Gly203Pro	substitution	203	Gly	Pro	0.000	0.077
p.Gly203Ser	substitution	203	Gly	Ser	1.344	0.014
p.Gly203Ter	nonsense	203	Gly	Ter	0.000	0.220
p.Gly203Thr	substitution	203	Gly	Thr	0.000	0.100
p.Gly203Trp	substitution	203	Gly	Trp	1.142	0.002
p.Gly203Val	substitution	203	Gly	Val	1.119	0.003
p.Gly216=	synonymous	216	Gly	NA	0.888	0.005
p.Gly216Ala	substitution	216	Gly	Ala	0.184	0.130
p.Gly216Arg	substitution	216	Gly	Arg	0.000	0.068
p.Gly216Asp	substitution	216	Gly	Asp	0.000	0.141
p.Gly216Cys	substitution	216	Gly	Cys	1.179	0.009
p.Gly216Gln	substitution	216	Gly	Gln	0.000	0.229
p.Gly216Glu	substitution	216	Gly	Glu	0.000	0.129
p.Gly216His	substitution	216	Gly	His	0.000	0.230
p.Gly216Ile	substitution	216	Gly	Ile	0.000	0.072
p.Gly216Leu	substitution	216	Gly	Leu	0.000	0.053
p.Gly216Lys	substitution	216	Gly	Lys	0.000	0.041
p.Gly216Met	substitution	216	Gly	Met	0.040	0.055
p.Gly216Phe	substitution	216	Gly	Phe	0.276	0.008
p.Gly216Pro	substitution	216	Gly	Pro	0.000	0.097
p.Gly216Ser	substitution	216	Gly	Ser	0.942	0.124
p.Gly216Ter	nonsense	216	Gly	Ter	0.000	0.090
p.Gly216Thr	substitution	216	Gly	Thr	0.000	0.025
p.Gly216Trp	substitution	216	Gly	Trp	0.000	0.016
p.Gly216Tyr	substitution	216	Gly	Tyr	0.339	0.278
p.Gly216Val	substitution	216	Gly	Val	0.000	0.075
p.Gly218=	synonymous	218	Gly	NA	0.735	0.111
p.Gly218Ala	substitution	218	Gly	Ala	0.361	0.056
p.Gly218Arg	substitution	218	Gly	Arg	0.000	0.017
p.Gly218Asn	substitution	218	Gly	Asn	0.000	0.034
p.Gly218Asp	substitution	218	Gly	Asp	0.000	0.016
p.Gly218Cys	substitution	218	Gly	Cys	0.000	0.138
p.Gly218Gln	substitution	218	Gly	Gln	0.000	0.029
p.Gly218Glu	substitution	218	Gly	Glu	0.122	0.046
p.Gly218His	substitution	218	Gly	His	0.000	0.018
p.Gly218Ile	substitution	218	Gly	Ile	0.000	0.068
p.Gly218Leu	substitution	218	Gly	Leu	0.141	0.044
p.Gly218Lys	substitution	218	Gly	Lys	0.000	0.065
p.Gly218Met	substitution	218	Gly	Met	0.000	0.222
p.Gly218Phe	substitution	218	Gly	Phe	0.000	0.173
p.Gly218Pro	substitution	218	Gly	Pro	0.000	0.113
p.Gly218Ser	substitution	218	Gly	Ser	0.000	0.019
p.Gly218Ter	nonsense	218	Gly	Ter	0.194	0.080

p.Gly218Thr	substitution	218	Gly	Thr		0.000	0.075
p.Gly218Trp	substitution	218	Gly	Trp		0.094	0.006
p.Gly218Tyr	substitution	218	Gly	Tyr		0.000	0.011
p.Gly218Val	substitution	218	Gly	Val		0.247	0.022
p.Gly221=	synonymous	221	Gly	NA		0.508	0.056
p.Gly221Ala	substitution	221	Gly	Ala		0.541	0.074
p.Gly221Arg	substitution	221	Gly	Arg		0.000	0.010
p.Gly221Asn	substitution	221	Gly	Asn		0.000	0.075
p.Gly221Asp	substitution	221	Gly	Asp		0.000	0.026
p.Gly221Cys	substitution	221	Gly	Cys		0.840	0.152
p.Gly221Glu	substitution	221	Gly	Glu		0.000	0.016
p.Gly221His	substitution	221	Gly	His		0.380	0.012
p.Gly221Ile	substitution	221	Gly	Ile		1.066	0.003
p.Gly221Leu	substitution	221	Gly	Leu		0.231	0.019
p.Gly221Lys	substitution	221	Gly	Lys		0.000	0.069
p.Gly221Met	substitution	221	Gly	Met		0.000	0.019
p.Gly221Phe	substitution	221	Gly	Phe		0.072	0.058
p.Gly221Pro	substitution	221	Gly	Pro		0.202	0.042
p.Gly221Ser	substitution	221	Gly	Ser		0.564	0.065
p.Gly221Ter	nonsense	221	Gly	Ter		0.000	0.104
p.Gly221Thr	substitution	221	Gly	Thr		0.482	0.009
p.Gly221Trp	substitution	221	Gly	Trp		0.000	0.014
p.Gly221Tyr	substitution	221	Gly	Tyr		0.243	0.023
p.Gly221Val	substitution	221	Gly	Val		0.057	0.042
p.Gly236=	synonymous	236	Gly	NA		0.895	0.002
p.Gly236Ala	substitution	236	Gly	Ala		1.119	0.264
p.Gly236Arg	substitution	236	Gly	Arg		1.030	0.183
p.Gly236Asn	substitution	236	Gly	Asn		0.000	0.252
p.Gly236Asp	substitution	236	Gly	Asp		1.257	0.006
p.Gly236Cys	substitution	236	Gly	Cys		1.372	0.002
p.Gly236Glu	substitution	236	Gly	Glu		0.000	0.047
p.Gly236Leu	substitution	236	Gly	Leu		0.228	0.049
p.Gly236Met	substitution	236	Gly	Met		0.000	0.206
p.Gly236Phe	substitution	236	Gly	Phe		0.000	0.010
p.Gly236Ser	substitution	236	Gly	Ser		0.950	0.008
p.Gly236Ter	nonsense	236	Gly	Ter		0.000	0.012
p.Gly236Thr	substitution	236	Gly	Thr		0.770	0.236
p.Gly236Trp	substitution	236	Gly	Trp		0.000	0.032
p.Gly236Tyr	substitution	236	Gly	Tyr		0.000	0.067
p.Gly236Val	substitution	236	Gly	Val		0.129	0.065
p.Gly24=	synonymous	24	Gly	NA		0.964	0.000
p.Gly24Ala	substitution	24	Gly	Ala		0.771	0.002
p.Gly24Arg	substitution	24	Gly	Arg		0.000	0.003

p.Gly24Asn	substitution	24	Gly	Asn	0.000	0.004
p.Gly24Asp	substitution	24	Gly	Asp	0.000	0.029
p.Gly24Cys	substitution	24	Gly	Cys	0.706	0.000
p.Gly24Gln	substitution	24	Gly	Gln	0.000	0.002
p.Gly24Glu	substitution	24	Gly	Glu	0.000	0.001
p.Gly24His	substitution	24	Gly	His	0.000	0.002
p.Gly24Ile	substitution	24	Gly	Ile	0.158	0.002
p.Gly24Leu	substitution	24	Gly	Leu	0.751	0.002
p.Gly24Lys	substitution	24	Gly	Lys	0.000	0.002
p.Gly24Met	substitution	24	Gly	Met	0.485	0.001
p.Gly24Phe	substitution	24	Gly	Phe	0.000	0.002
p.Gly24Pro	substitution	24	Gly	Pro	0.000	0.020
p.Gly24Ser	substitution	24	Gly	Ser	0.461	0.008
p.Gly24Ter	nonsense	24	Gly	Ter	0.000	0.001
p.Gly24Thr	substitution	24	Gly	Thr	0.080	0.003
p.Gly24Trp	substitution	24	Gly	Trp	0.000	0.001
p.Gly24Tyr	substitution	24	Gly	Tyr	0.000	0.001
p.Gly24Val	substitution	24	Gly	Val	0.692	0.002
p.Gly259=	synonymous	259	Gly	NA	0.925	0.056
p.Gly259Ala	substitution	259	Gly	Ala	0.850	0.028
p.Gly259Arg	substitution	259	Gly	Arg	0.447	0.063
p.Gly259Asn	substitution	259	Gly	Asn	0.000	0.137
p.Gly259Asp	substitution	259	Gly	Asp	0.895	0.005
p.Gly259Cys	substitution	259	Gly	Cys	0.780	0.012
p.Gly259Gln	substitution	259	Gly	Gln	0.000	0.027
p.Gly259Glu	substitution	259	Gly	Glu	0.850	0.009
p.Gly259His	substitution	259	Gly	His	0.000	0.031
p.Gly259Leu	substitution	259	Gly	Leu	0.924	0.055
p.Gly259Lys	substitution	259	Gly	Lys	1.236	0.003
p.Gly259Phe	substitution	259	Gly	Phe	0.900	0.006
p.Gly259Pro	substitution	259	Gly	Pro	0.000	0.142
p.Gly259Ser	substitution	259	Gly	Ser	0.864	0.042
p.Gly259Ter	nonsense	259	Gly	Ter	0.552	0.273
p.Gly259Thr	substitution	259	Gly	Thr	1.059	0.054
p.Gly259Trp	substitution	259	Gly	Trp	0.000	0.095
p.Gly259Tyr	substitution	259	Gly	Tyr	0.330	0.010
p.Gly259Val	substitution	259	Gly	Val	0.356	0.089
p.Gly260=	synonymous	260	Gly	NA	0.429	0.042
p.Gly260Ala	substitution	260	Gly	Ala	0.210	0.045
p.Gly260Arg	substitution	260	Gly	Arg	0.000	0.118
p.Gly260Asp	substitution	260	Gly	Asp	1.387	0.003
p.Gly260Cys	substitution	260	Gly	Cys	1.258	0.005
p.Gly260Ser	substitution	260	Gly	Ser	0.000	0.085

p.Gly260Trp	substitution	260	Gly	Trp		0.256	0.201
p.Gly260Val	substitution	260	Gly	Val		1.211	0.335
p.Gly274=	synonymous	274	Gly	NA		1.201	0.014
p.Gly274Ala	substitution	274	Gly	Ala		1.563	0.264
p.Gly274Arg	substitution	274	Gly	Arg		0.273	0.130
p.Gly274Asn	substitution	274	Gly	Asn		0.000	0.011
p.Gly274Cys	substitution	274	Gly	Cys		0.191	0.047
p.Gly274Glu	substitution	274	Gly	Glu		1.747	0.088
p.Gly274His	substitution	274	Gly	His		0.278	0.015
p.Gly274Ile	substitution	274	Gly	Ile		0.000	0.057
p.Gly274Leu	substitution	274	Gly	Leu		0.710	0.040
p.Gly274Lys	substitution	274	Gly	Lys		0.652	0.065
p.Gly274Ser	substitution	274	Gly	Ser		0.000	0.077
p.Gly274Ter	nonsense	274	Gly	Ter		0.000	0.034
p.Gly274Thr	substitution	274	Gly	Thr		0.000	0.174
p.Gly274Trp	substitution	274	Gly	Trp		0.784	0.009
p.Gly274Tyr	substitution	274	Gly	Tyr		0.000	0.029
p.Gly274Val	substitution	274	Gly	Val		0.970	0.032
p.Gly280=	synonymous	280	Gly	NA		0.565	0.063
p.Gly280Ala	substitution	280	Gly	Ala		1.174	0.004
p.Gly280Arg	substitution	280	Gly	Arg		0.063	0.078
p.Gly280Asn	substitution	280	Gly	Asn		0.000	0.006
p.Gly280Asp	substitution	280	Gly	Asp		0.139	0.022
p.Gly280Cys	substitution	280	Gly	Cys		0.000	0.084
p.Gly280Gln	substitution	280	Gly	Gln		0.000	0.010
p.Gly280Glu	substitution	280	Gly	Glu		0.000	0.098
p.Gly280Ile	substitution	280	Gly	Ile		0.000	0.130
p.Gly280Leu	substitution	280	Gly	Leu		0.075	0.040
p.Gly280Lys	substitution	280	Gly	Lys		0.715	0.011
p.Gly280Met	substitution	280	Gly	Met		0.000	0.008
p.Gly280Phe	substitution	280	Gly	Phe		0.000	0.008
p.Gly280Pro	substitution	280	Gly	Pro		0.000	0.036
p.Gly280Ser	substitution	280	Gly	Ser		0.184	0.041
p.Gly280Ter	nonsense	280	Gly	Ter		0.090	0.072
p.Gly280Thr	substitution	280	Gly	Thr		0.000	0.013
p.Gly280Trp	substitution	280	Gly	Trp		0.000	0.007
p.Gly280Tyr	substitution	280	Gly	Tyr		0.000	0.012
p.Gly280Val	substitution	280	Gly	Val		0.514	0.060
p.Gly281=	synonymous	281	Gly	NA		1.227	0.019
p.Gly281Ala	substitution	281	Gly	Ala		0.929	0.107
p.Gly281Arg	substitution	281	Gly	Arg		0.679	0.132
p.Gly281Asn	substitution	281	Gly	Asn		0.000	0.020
p.Gly281Asp	substitution	281	Gly	Asp		0.000	0.056

p.Gly281Cys	substitution	281	Gly	Cys		0.795	0.092
p.Gly281Glu	substitution	281	Gly	Glu		1.608	0.018
p.Gly281His	substitution	281	Gly	His		0.251	0.021
p.Gly281Ile	substitution	281	Gly	Ile		0.000	0.137
p.Gly281Leu	substitution	281	Gly	Leu		0.625	0.087
p.Gly281Lys	substitution	281	Gly	Lys		0.000	0.024
p.Gly281Met	substitution	281	Gly	Met		0.000	0.016
p.Gly281Pro	substitution	281	Gly	Pro		0.000	0.030
p.Gly281Ser	substitution	281	Gly	Ser		1.915	0.008
p.Gly281Ter	nonsense	281	Gly	Ter		0.304	0.067
p.Gly281Trp	substitution	281	Gly	Trp		0.816	0.057
p.Gly281Tyr	substitution	281	Gly	Tyr		0.000	0.058
p.Gly281Val	substitution	281	Gly	Val		0.792	0.065
p.Gly287=	synonymous	287	Gly	NA		1.030	0.005
p.Gly287Ala	substitution	287	Gly	Ala		0.333	0.068
p.Gly287Arg	substitution	287	Gly	Arg		0.503	0.081
p.Gly287Asn	substitution	287	Gly	Asn		0.029	0.015
p.Gly287Asp	substitution	287	Gly	Asp		0.464	0.012
p.Gly287Cys	substitution	287	Gly	Cys		0.345	0.014
p.Gly287Gln	substitution	287	Gly	Gln		0.000	0.032
p.Gly287Glu	substitution	287	Gly	Glu		0.000	0.014
p.Gly287His	substitution	287	Gly	His		0.260	0.104
p.Gly287Ile	substitution	287	Gly	Ile		0.250	0.030
p.Gly287Leu	substitution	287	Gly	Leu		0.348	0.067
p.Gly287Lys	substitution	287	Gly	Lys		0.000	0.076
p.Gly287Met	substitution	287	Gly	Met		1.094	0.005
p.Gly287Phe	substitution	287	Gly	Phe		0.208	0.051
p.Gly287Pro	substitution	287	Gly	Pro		0.000	0.013
p.Gly287Ser	substitution	287	Gly	Ser		0.574	0.061
p.Gly287Ter	nonsense	287	Gly	Ter		0.071	0.048
p.Gly287Thr	substitution	287	Gly	Thr		0.000	0.023
p.Gly287Trp	substitution	287	Gly	Trp		0.000	0.083
p.Gly287Tyr	substitution	287	Gly	Tyr		0.399	0.062
p.Gly287Val	substitution	287	Gly	Val		1.290	0.016
p.Gly308=	synonymous	308	Gly	NA		0.779	0.040
p.Gly308Ala	substitution	308	Gly	Ala		0.905	0.008
p.Gly308Arg	substitution	308	Gly	Arg		0.567	0.013
p.Gly308Asn	substitution	308	Gly	Asn		1.073	0.006
p.Gly308Asp	substitution	308	Gly	Asp		1.049	0.029
p.Gly308Cys	substitution	308	Gly	Cys		0.834	0.003
p.Gly308Gln	substitution	308	Gly	Gln		1.292	0.003
p.Gly308Ile	substitution	308	Gly	Ile		1.185	0.010
p.Gly308Leu	substitution	308	Gly	Leu		0.890	0.012

p.Gly308Lys	substitution	308	Gly	Lys		0.000	0.048
p.Gly308Met	substitution	308	Gly	Met		0.097	0.007
p.Gly308Phe	substitution	308	Gly	Phe		0.000	0.184
p.Gly308Pro	substitution	308	Gly	Pro		0.316	0.036
p.Gly308Ser	substitution	308	Gly	Ser		0.764	0.070
p.Gly308Ter	nonsense	308	Gly	Ter		0.105	0.008
p.Gly308Thr	substitution	308	Gly	Thr		0.658	0.006
p.Gly308Trp	substitution	308	Gly	Trp		0.895	0.001
p.Gly308Tyr	substitution	308	Gly	Tyr		0.000	0.104
p.Gly308Val	substitution	308	Gly	Val		1.018	0.007
p.Gly317=	synonymous	317	Gly	NA		1.013	0.024
p.Gly317Ala	substitution	317	Gly	Ala		0.000	0.088
p.Gly317Arg	substitution	317	Gly	Arg		0.079	0.043
p.Gly317Asp	substitution	317	Gly	Asp		0.296	0.133
p.Gly317Cys	substitution	317	Gly	Cys		1.159	0.021
p.Gly317Gln	substitution	317	Gly	Gln		0.000	0.018
p.Gly317Glu	substitution	317	Gly	Glu		0.000	0.061
p.Gly317His	substitution	317	Gly	His		0.000	0.033
p.Gly317Ile	substitution	317	Gly	Ile		0.729	0.047
p.Gly317Leu	substitution	317	Gly	Leu		0.016	0.023
p.Gly317Lys	substitution	317	Gly	Lys		0.000	0.157
p.Gly317Met	substitution	317	Gly	Met		1.257	0.001
p.Gly317Phe	substitution	317	Gly	Phe		0.000	0.078
p.Gly317Pro	substitution	317	Gly	Pro		0.848	0.013
p.Gly317Ser	substitution	317	Gly	Ser		0.173	0.060
p.Gly317Ter	nonsense	317	Gly	Ter		0.000	0.187
p.Gly317Thr	substitution	317	Gly	Thr		0.531	0.053
p.Gly317Trp	substitution	317	Gly	Trp		0.937	0.003
p.Gly317Tyr	substitution	317	Gly	Tyr		0.519	0.023
p.Gly317Val	substitution	317	Gly	Val		0.457	0.056
p.Gly326=	synonymous	326	Gly	NA		0.306	0.613
p.Gly326Ala	substitution	326	Gly	Ala		1.265	0.004
p.Gly326Arg	substitution	326	Gly	Arg		0.653	0.046
p.Gly326Cys	substitution	326	Gly	Cys		1.042	0.098
p.Gly326Gln	substitution	326	Gly	Gln		0.000	0.087
p.Gly326Glu	substitution	326	Gly	Glu		1.072	0.002
p.Gly326Leu	substitution	326	Gly	Leu		0.239	0.124
p.Gly326Phe	substitution	326	Gly	Phe		0.000	0.077
p.Gly326Pro	substitution	326	Gly	Pro		0.900	0.054
p.Gly326Ser	substitution	326	Gly	Ser		0.864	0.032
p.Gly326Ter	nonsense	326	Gly	Ter		0.054	0.198
p.Gly326Trp	substitution	326	Gly	Trp		0.799	0.003
p.Gly326Tyr	substitution	326	Gly	Tyr		0.942	0.028

p.Gly326Val	substitution	326	Gly	Val		0.786	0.003
p.Gly335=	synonymous	335	Gly	NA		0.965	0.054
p.Gly335Ala	substitution	335	Gly	Ala		0.023	0.182
p.Gly335Arg	substitution	335	Gly	Arg		0.000	0.130
p.Gly335Asp	substitution	335	Gly	Asp		0.432	0.061
p.Gly335Cys	substitution	335	Gly	Cys		0.566	0.148
p.Gly335Gln	substitution	335	Gly	Gln		0.306	0.250
p.Gly335Glu	substitution	335	Gly	Glu		0.036	0.130
p.Gly335Ile	substitution	335	Gly	Ile		0.000	0.187
p.Gly335Leu	substitution	335	Gly	Leu		0.000	0.031
p.Gly335Lys	substitution	335	Gly	Lys		0.020	0.054
p.Gly335Met	substitution	335	Gly	Met		0.192	0.042
p.Gly335Phe	substitution	335	Gly	Phe		0.802	0.048
p.Gly335Pro	substitution	335	Gly	Pro		0.000	0.265
p.Gly335Ser	substitution	335	Gly	Ser		0.000	0.065
p.Gly335Ter	nonsense	335	Gly	Ter		0.438	0.050
p.Gly335Thr	substitution	335	Gly	Thr		0.113	0.014
p.Gly335Trp	substitution	335	Gly	Trp		0.064	0.012
p.Gly335Tyr	substitution	335	Gly	Tyr		0.000	0.077
p.Gly335Val	substitution	335	Gly	Val		0.000	0.089
p.Gly346=	synonymous	346	Gly	NA		0.037	0.503
p.Gly346Ala	substitution	346	Gly	Ala		0.361	0.057
p.Gly346Arg	substitution	346	Gly	Arg		0.863	0.018
p.Gly346Asn	substitution	346	Gly	Asn		0.577	0.204
p.Gly346Asp	substitution	346	Gly	Asp		0.054	0.123
p.Gly346Cys	substitution	346	Gly	Cys		1.266	0.003
p.Gly346Gln	substitution	346	Gly	Gln		0.000	0.066
p.Gly346Glu	substitution	346	Gly	Glu		1.334	0.063
p.Gly346His	substitution	346	Gly	His		0.271	0.023
p.Gly346Leu	substitution	346	Gly	Leu		0.704	0.173
p.Gly346Lys	substitution	346	Gly	Lys		0.000	0.021
p.Gly346Phe	substitution	346	Gly	Phe		0.511	0.005
p.Gly346Pro	substitution	346	Gly	Pro		0.000	0.113
p.Gly346Ser	substitution	346	Gly	Ser		0.000	0.075
p.Gly346Ter	nonsense	346	Gly	Ter		0.848	0.015
p.Gly346Thr	substitution	346	Gly	Thr		0.667	0.011
p.Gly346Tyr	substitution	346	Gly	Tyr		0.000	0.056
p.Gly346Val	substitution	346	Gly	Val		0.593	0.025
p.Gly48=	synonymous	48	Gly	NA		0.974	0.009
p.Gly48Ala	substitution	48	Gly	Ala		1.003	0.021
p.Gly48Arg	substitution	48	Gly	Arg		0.856	0.019
p.Gly48Asn	substitution	48	Gly	Asn		1.325	0.010
p.Gly48Asp	substitution	48	Gly	Asp		1.008	0.004

p.Gly48Cys	substitution	48	Gly	Cys	0.903	0.009
p.Gly48Gln	substitution	48	Gly	Gln	1.158	0.007
p.Gly48Glu	substitution	48	Gly	Glu	0.995	0.003
p.Gly48His	substitution	48	Gly	His	0.164	0.084
p.Gly48Ile	substitution	48	Gly	Ile	0.699	0.111
p.Gly48Leu	substitution	48	Gly	Leu	1.004	0.029
p.Gly48Lys	substitution	48	Gly	Lys	1.101	0.009
p.Gly48Met	substitution	48	Gly	Met	0.492	0.007
p.Gly48Phe	substitution	48	Gly	Phe	0.592	0.057
p.Gly48Pro	substitution	48	Gly	Pro	0.368	0.027
p.Gly48Ser	substitution	48	Gly	Ser	0.914	0.021
p.Gly48Ter	nonsense	48	Gly	Ter	0.000	0.016
p.Gly48Thr	substitution	48	Gly	Thr	0.947	0.020
p.Gly48Trp	substitution	48	Gly	Trp	0.818	0.005
p.Gly48Tyr	substitution	48	Gly	Tyr	0.721	0.015
p.Gly48Val	substitution	48	Gly	Val	0.844	0.018
p.Gly60=	synonymous	60	Gly	NA	1.223	0.052
p.Gly60Ala	substitution	60	Gly	Ala	0.204	0.137
p.Gly60Arg	substitution	60	Gly	Arg	0.231	0.056
p.Gly60Asn	substitution	60	Gly	Asn	0.632	0.040
p.Gly60Asp	substitution	60	Gly	Asp	0.099	0.100
p.Gly60Gln	substitution	60	Gly	Gln	0.594	0.055
p.Gly60Glu	substitution	60	Gly	Glu	0.000	0.631
p.Gly60His	substitution	60	Gly	His	0.040	0.488
p.Gly60Ile	substitution	60	Gly	Ile	0.000	0.160
p.Gly60Leu	substitution	60	Gly	Leu	0.000	0.123
p.Gly60Lys	substitution	60	Gly	Lys	0.000	0.061
p.Gly60Met	substitution	60	Gly	Met	0.000	0.115
p.Gly60Phe	substitution	60	Gly	Phe	0.000	0.869
p.Gly60Pro	substitution	60	Gly	Pro	0.000	0.084
p.Gly60Ser	substitution	60	Gly	Ser	0.000	0.077
p.Gly60Ter	nonsense	60	Gly	Ter	0.000	0.884
p.Gly60Thr	substitution	60	Gly	Thr	0.542	0.035
p.Gly60Trp	substitution	60	Gly	Trp	0.530	0.014
p.Gly60Tyr	substitution	60	Gly	Tyr	0.000	0.446
p.Gly60Val	substitution	60	Gly	Val	0.871	0.008
p.Gly72=	synonymous	72	Gly	NA	0.978	0.028
p.Gly72Ala	substitution	72	Gly	Ala	0.821	0.939
p.Gly72Arg	substitution	72	Gly	Arg	0.610	0.050
p.Gly72Asp	substitution	72	Gly	Asp	0.496	0.015
p.Gly72Cys	substitution	72	Gly	Cys	0.402	0.028
p.Gly72Gln	substitution	72	Gly	Gln	0.152	0.023
p.Gly72Glu	substitution	72	Gly	Glu	1.015	0.006

p.Gly72His	substitution	72	Gly	His		0.709	0.009
p.Gly72Ile	substitution	72	Gly	Ile		1.045	0.041
p.Gly72Leu	substitution	72	Gly	Leu		0.582	0.018
p.Gly72Lys	substitution	72	Gly	Lys		0.088	0.030
p.Gly72Met	substitution	72	Gly	Met		0.755	0.014
p.Gly72Phe	substitution	72	Gly	Phe		1.145	0.005
p.Gly72Pro	substitution	72	Gly	Pro		1.061	0.055
p.Gly72Ser	substitution	72	Gly	Ser		1.019	0.014
p.Gly72Ter	nonsense	72	Gly	Ter		0.703	0.039
p.Gly72Thr	substitution	72	Gly	Thr		0.716	0.010
p.Gly72Trp	substitution	72	Gly	Trp		0.380	0.013
p.Gly72Tyr	substitution	72	Gly	Tyr		0.837	0.003
p.Gly72Val	substitution	72	Gly	Val		0.742	0.009
p.His120Ala	substitution	120	His	Ala		0.307	0.102
p.His120Arg	substitution	120	His	Arg		1.012	0.008
p.His120Asn	substitution	120	His	Asn		0.964	0.003
p.His120Asp	substitution	120	His	Asp		0.781	0.004
p.His120Cys	substitution	120	His	Cys		0.962	0.003
p.His120Gln	substitution	120	His	Gln		0.712	0.041
p.His120Glu	substitution	120	His	Glu		1.090	0.004
p.His120Gly	substitution	120	His	Gly		0.742	0.005
p.His120Ile	substitution	120	His	Ile		0.396	0.006
p.His120Leu	substitution	120	His	Leu		1.061	0.008
p.His120Met	substitution	120	His	Met		0.989	0.005
p.His120Phe	substitution	120	His	Phe		1.235	0.003
p.His120Pro	substitution	120	His	Pro		0.121	0.006
p.His120Ser	substitution	120	His	Ser		0.966	0.005
p.His120Ter	nonsense	120	His	Ter		0.047	0.013
p.His120Thr	substitution	120	His	Thr		1.199	0.002
p.His120Trp	substitution	120	His	Trp		0.999	0.004
p.His120Tyr	substitution	120	His	Tyr		1.056	0.006
p.His120Val	substitution	120	His	Val		0.700	0.012
p.His126=	synonymous	126	His	NA		0.728	0.019
p.His126Ala	substitution	126	His	Ala		0.808	0.014
p.His126Arg	substitution	126	His	Arg		0.650	0.022
p.His126Asn	substitution	126	His	Asn		0.856	0.055
p.His126Asp	substitution	126	His	Asp		0.768	0.067
p.His126Cys	substitution	126	His	Cys		1.032	0.024
p.His126Gln	substitution	126	His	Gln		1.011	0.011
p.His126Glu	substitution	126	His	Glu		1.047	0.183
p.His126Gly	substitution	126	His	Gly		0.878	0.010
p.His126Ile	substitution	126	His	Ile		1.310	0.018
p.His126Leu	substitution	126	His	Leu		0.671	0.040

p.His126Lys	substitution	126	His	Lys		0.999	0.033
p.His126Met	substitution	126	His	Met		0.607	0.007
p.His126Phe	substitution	126	His	Phe		0.602	0.009
p.His126Pro	substitution	126	His	Pro		0.904	0.031
p.His126Ser	substitution	126	His	Ser		1.100	0.007
p.His126Ter	nonsense	126	His	Ter		0.168	0.023
p.His126Thr	substitution	126	His	Thr		0.679	0.037
p.His126Trp	substitution	126	His	Trp		0.341	0.017
p.His126Tyr	substitution	126	His	Tyr		0.396	0.042
p.His126Val	substitution	126	His	Val		0.543	0.016
p.His160Ala	substitution	160	His	Ala		0.359	0.034
p.His160Arg	substitution	160	His	Arg		1.097	0.012
p.His160Asn	substitution	160	His	Asn		0.986	0.009
p.His160Asp	substitution	160	His	Asp		0.956	0.008
p.His160Gln	substitution	160	His	Gln		1.046	0.004
p.His160Glu	substitution	160	His	Glu		0.980	0.005
p.His160Gly	substitution	160	His	Gly		0.891	0.021
p.His160Ile	substitution	160	His	Ile		0.821	0.006
p.His160Leu	substitution	160	His	Leu		0.839	0.005
p.His160Lys	substitution	160	His	Lys		1.125	0.006
p.His160Met	substitution	160	His	Met		1.186	0.010
p.His160Pro	substitution	160	His	Pro		0.834	0.010
p.His160Ser	substitution	160	His	Ser		0.826	0.033
p.His160Ter	nonsense	160	His	Ter		0.000	0.014
p.His160Thr	substitution	160	His	Thr		1.073	0.016
p.His160Trp	substitution	160	His	Trp		0.802	0.009
p.His160Val	substitution	160	His	Val		0.258	0.013
p.His199=	synonymous	199	His	NA		0.852	0.001
p.His199Ala	substitution	199	His	Ala		0.804	0.048
p.His199Arg	substitution	199	His	Arg		0.900	0.029
p.His199Asn	substitution	199	His	Asn		1.119	0.002
p.His199Asp	substitution	199	His	Asp		1.444	0.037
p.His199Gln	substitution	199	His	Gln		0.387	0.012
p.His199Glu	substitution	199	His	Glu		0.787	0.004
p.His199Gly	substitution	199	His	Gly		0.766	0.021
p.His199Ile	substitution	199	His	Ile		0.970	0.013
p.His199Leu	substitution	199	His	Leu		0.755	0.019
p.His199Lys	substitution	199	His	Lys		1.083	0.007
p.His199Met	substitution	199	His	Met		1.052	0.002
p.His199Phe	substitution	199	His	Phe		1.274	0.005
p.His199Pro	substitution	199	His	Pro		1.263	0.008
p.His199Ser	substitution	199	His	Ser		1.054	0.038
p.His199Ter	nonsense	199	His	Ter		0.290	0.234

p.His199Thr	substitution	199	His	Thr		0.417	0.017
p.His199Trp	substitution	199	His	Trp		0.807	0.008
p.His199Tyr	substitution	199	His	Tyr		1.076	0.004
p.His199Val	substitution	199	His	Val		1.126	0.004
p.His207=	synonymous	207	His	NA		0.206	0.080
p.His207Ala	substitution	207	His	Ala		1.087	0.100
p.His207Arg	substitution	207	His	Arg		0.616	0.104
p.His207Asn	substitution	207	His	Asn		0.346	0.006
p.His207Asp	substitution	207	His	Asp		0.315	0.153
p.His207Cys	substitution	207	His	Cys		0.900	0.010
p.His207Gln	substitution	207	His	Gln		0.974	0.009
p.His207Glu	substitution	207	His	Glu		1.133	0.002
p.His207Gly	substitution	207	His	Gly		0.849	0.013
p.His207Ile	substitution	207	His	Ile		0.525	0.012
p.His207Leu	substitution	207	His	Leu		0.855	0.011
p.His207Lys	substitution	207	His	Lys		1.039	0.002
p.His207Phe	substitution	207	His	Phe		0.620	0.014
p.His207Pro	substitution	207	His	Pro		0.905	0.032
p.His207Ser	substitution	207	His	Ser		1.036	0.023
p.His207Ter	nonsense	207	His	Ter		0.037	0.035
p.His207Thr	substitution	207	His	Thr		1.010	0.008
p.His207Trp	substitution	207	His	Trp		1.123	0.004
p.His207Tyr	substitution	207	His	Tyr		1.149	0.040
p.His207Val	substitution	207	His	Val		0.808	0.010
p.His239=	synonymous	239	His	NA		1.236	0.001
p.His239Ala	substitution	239	His	Ala		0.980	0.006
p.His239Arg	substitution	239	His	Arg		0.000	0.033
p.His239Asn	substitution	239	His	Asn		0.515	0.028
p.His239Asp	substitution	239	His	Asp		1.087	0.030
p.His239Cys	substitution	239	His	Cys		0.794	0.006
p.His239Gln	substitution	239	His	Gln		0.586	0.014
p.His239Glu	substitution	239	His	Glu		0.250	0.008
p.His239Gly	substitution	239	His	Gly		0.000	0.033
p.His239Ile	substitution	239	His	Ile		0.815	0.019
p.His239Leu	substitution	239	His	Leu		0.350	0.017
p.His239Lys	substitution	239	His	Lys		0.366	0.006
p.His239Met	substitution	239	His	Met		0.313	0.022
p.His239Phe	substitution	239	His	Phe		0.000	0.035
p.His239Pro	substitution	239	His	Pro		0.521	0.226
p.His239Ser	substitution	239	His	Ser		0.932	0.056
p.His239Ter	nonsense	239	His	Ter		0.000	0.015
p.His239Thr	substitution	239	His	Thr		1.104	0.090
p.His239Trp	substitution	239	His	Trp		0.000	0.222

p.His239Tyr	substitution	239	His	Tyr		0.387	0.059
p.His239Val	substitution	239	His	Val		0.084	0.032
p.His256=	synonymous	256	His	NA		0.906	0.006
p.His256Ala	substitution	256	His	Ala		1.403	0.010
p.His256Arg	substitution	256	His	Arg		0.897	0.012
p.His256Asn	substitution	256	His	Asn		0.273	0.014
p.His256Asp	substitution	256	His	Asp		0.075	0.062
p.His256Cys	substitution	256	His	Cys		0.937	0.006
p.His256Gln	substitution	256	His	Gln		0.325	0.014
p.His256Glu	substitution	256	His	Glu		1.371	0.002
p.His256Gly	substitution	256	His	Gly		1.253	0.264
p.His256Ile	substitution	256	His	Ile		0.163	0.209
p.His256Leu	substitution	256	His	Leu		0.754	0.054
p.His256Lys	substitution	256	His	Lys		0.371	0.006
p.His256Phe	substitution	256	His	Phe		1.213	0.006
p.His256Pro	substitution	256	His	Pro		0.276	0.036
p.His256Ser	substitution	256	His	Ser		0.918	0.212
p.His256Ter	nonsense	256	His	Ter		0.000	0.037
p.His256Thr	substitution	256	His	Thr		1.088	0.021
p.His256Trp	substitution	256	His	Trp		1.247	0.026
p.His256Tyr	substitution	256	His	Tyr		0.354	0.092
p.His256Val	substitution	256	His	Val		0.731	0.012
p.His268Ala	substitution	268	His	Ala		1.076	0.042
p.His268Arg	substitution	268	His	Arg		1.371	0.005
p.His268Asn	substitution	268	His	Asn		1.228	0.127
p.His268Asp	substitution	268	His	Asp		0.766	0.272
p.His268Cys	substitution	268	His	Cys		0.575	0.021
p.His268Gln	substitution	268	His	Gln		1.197	0.005
p.His268Glu	substitution	268	His	Glu		1.239	0.006
p.His268Gly	substitution	268	His	Gly		0.000	0.016
p.His268Ile	substitution	268	His	Ile		0.087	0.026
p.His268Leu	substitution	268	His	Leu		0.700	0.151
p.His268Lys	substitution	268	His	Lys		0.688	0.064
p.His268Met	substitution	268	His	Met		1.297	0.008
p.His268Phe	substitution	268	His	Phe		1.032	0.005
p.His268Pro	substitution	268	His	Pro		0.164	0.049
p.His268Ser	substitution	268	His	Ser		0.163	0.032
p.His268Thr	substitution	268	His	Thr		0.000	0.019
p.His268Tyr	substitution	268	His	Tyr		1.100	0.010
p.His268Val	substitution	268	His	Val		1.190	0.255
p.His300Ala	substitution	300	His	Ala		0.000	0.129
p.His300Arg	substitution	300	His	Arg		0.278	0.037
p.His300Asn	substitution	300	His	Asn		0.965	0.004

p.His300Asp	substitution	300	His	Asp		1.416	0.002
p.His300Cys	substitution	300	His	Cys		1.034	0.006
p.His300Gln	substitution	300	His	Gln		0.852	0.033
p.His300Glu	substitution	300	His	Glu		0.913	0.016
p.His300Gly	substitution	300	His	Gly		0.998	0.007
p.His300Ile	substitution	300	His	Ile		0.841	0.058
p.His300Leu	substitution	300	His	Leu		0.935	0.040
p.His300Lys	substitution	300	His	Lys		1.146	0.003
p.His300Met	substitution	300	His	Met		0.000	0.043
p.His300Phe	substitution	300	His	Phe		1.177	0.008
p.His300Pro	substitution	300	His	Pro		0.817	0.055
p.His300Ser	substitution	300	His	Ser		0.609	0.092
p.His300Ter	nonsense	300	His	Ter		0.000	0.018
p.His300Thr	substitution	300	His	Thr		1.201	0.002
p.His300Val	substitution	300	His	Val		1.097	0.266
p.His305=	synonymous	305	His	NA		1.175	0.686
p.His305Ala	substitution	305	His	Ala		0.000	0.022
p.His305Arg	substitution	305	His	Arg		0.310	0.056
p.His305Asn	substitution	305	His	Asn		1.026	0.003
p.His305Asp	substitution	305	His	Asp		1.125	0.002
p.His305Cys	substitution	305	His	Cys		1.132	0.003
p.His305Gln	substitution	305	His	Gln		1.025	0.045
p.His305Glu	substitution	305	His	Glu		1.295	0.004
p.His305Gly	substitution	305	His	Gly		1.083	0.007
p.His305Ile	substitution	305	His	Ile		1.104	0.002
p.His305Leu	substitution	305	His	Leu		0.835	0.158
p.His305Lys	substitution	305	His	Lys		0.038	0.015
p.His305Met	substitution	305	His	Met		0.548	0.003
p.His305Phe	substitution	305	His	Phe		0.000	0.021
p.His305Pro	substitution	305	His	Pro		0.000	0.015
p.His305Ser	substitution	305	His	Ser		0.439	0.071
p.His305Ter	nonsense	305	His	Ter		0.003	0.058
p.His305Thr	substitution	305	His	Thr		0.000	0.123
p.His305Trp	substitution	305	His	Trp		0.708	0.005
p.His305Tyr	substitution	305	His	Tyr		1.204	0.003
p.His305Val	substitution	305	His	Val		0.791	0.087
p.His83=	synonymous	83	His	NA		1.992	0.045
p.His83Ala	substitution	83	His	Ala		0.978	0.007
p.His83Arg	substitution	83	His	Arg		1.026	0.001
p.His83Asn	substitution	83	His	Asn		1.004	0.003
p.His83Asp	substitution	83	His	Asp		0.970	0.003
p.His83Cys	substitution	83	His	Cys		1.048	0.003
p.His83Gln	substitution	83	His	Gln		1.053	0.006

p.His83Glu	substitution	83	His	Glu		1.014	0.003
p.His83Gly	substitution	83	His	Gly		0.810	0.003
p.His83Ile	substitution	83	His	Ile		0.690	0.005
p.His83Leu	substitution	83	His	Leu		1.125	0.006
p.His83Lys	substitution	83	His	Lys		0.920	0.012
p.His83Met	substitution	83	His	Met		0.984	0.011
p.His83Phe	substitution	83	His	Phe		0.906	0.007
p.His83Pro	substitution	83	His	Pro		0.386	0.005
p.His83Ser	substitution	83	His	Ser		0.961	0.009
p.His83Ter	nonsense	83	His	Ter		0.040	0.016
p.His83Thr	substitution	83	His	Thr		1.004	0.029
p.His83Trp	substitution	83	His	Trp		1.076	0.004
p.His83Tyr	substitution	83	His	Tyr		1.129	0.003
p.His83Val	substitution	83	His	Val		0.847	0.002
p.His95=	synonymous	95	His	NA		0.908	0.004
p.His95Ala	substitution	95	His	Ala		0.699	0.011
p.His95Arg	substitution	95	His	Arg		0.000	0.018
p.His95Asn	substitution	95	His	Asn		1.065	0.004
p.His95Asp	substitution	95	His	Asp		0.144	0.069
p.His95Gln	substitution	95	His	Gln		0.849	0.052
p.His95Glu	substitution	95	His	Glu		0.090	0.019
p.His95Gly	substitution	95	His	Gly		0.211	0.013
p.His95Ile	substitution	95	His	Ile		0.000	0.068
p.His95Leu	substitution	95	His	Leu		0.704	0.027
p.His95Lys	substitution	95	His	Lys		0.000	0.045
p.His95Met	substitution	95	His	Met		0.423	0.007
p.His95Phe	substitution	95	His	Phe		0.947	0.023
p.His95Pro	substitution	95	His	Pro		0.000	0.015
p.His95Ser	substitution	95	His	Ser		0.284	0.017
p.His95Ter	nonsense	95	His	Ter		0.178	0.008
p.His95Thr	substitution	95	His	Thr		0.525	0.271
p.His95Trp	substitution	95	His	Trp		0.000	0.013
p.His95Tyr	substitution	95	His	Tyr		0.026	0.054
p.His95Val	substitution	95	His	Val		0.739	0.027
p.Ile110=	synonymous	110	Ile	NA		0.966	0.009
p.Ile110Ala	substitution	110	Ile	Ala		0.000	0.051
p.Ile110Arg	substitution	110	Ile	Arg		0.000	0.017
p.Ile110Asn	substitution	110	Ile	Asn		0.211	0.022
p.Ile110Asp	substitution	110	Ile	Asp		0.175	0.043
p.Ile110Cys	substitution	110	Ile	Cys		0.728	0.010
p.Ile110Gln	substitution	110	Ile	Gln		0.000	0.031
p.Ile110Glu	substitution	110	Ile	Glu		0.124	0.027
p.Ile110Gly	substitution	110	Ile	Gly		0.000	0.072

p.Ile110His	substitution	110	Ile	His	0.159	0.015
p.Ile110Leu	substitution	110	Ile	Leu	0.771	0.013
p.Ile110Lys	substitution	110	Ile	Lys	0.183	0.025
p.Ile110Phe	substitution	110	Ile	Phe	0.309	0.170
p.Ile110Pro	substitution	110	Ile	Pro	0.000	0.045
p.Ile110Ser	substitution	110	Ile	Ser	0.000	0.024
p.Ile110Ter	nonsense	110	Ile	Ter	0.000	0.014
p.Ile110Thr	substitution	110	Ile	Thr	0.311	0.017
p.Ile110Trp	substitution	110	Ile	Trp	0.000	0.017
p.Ile110Tyr	substitution	110	Ile	Tyr	0.095	0.033
p.Ile110Val	substitution	110	Ile	Val	0.824	0.065
p.Ile113=	synonymous	113	Ile	NA	1.089	0.010
p.Ile113Ala	substitution	113	Ile	Ala	0.759	0.102
p.Ile113Arg	substitution	113	Ile	Arg	0.000	0.126
p.Ile113Asn	substitution	113	Ile	Asn	0.000	0.051
p.Ile113Asp	substitution	113	Ile	Asp	0.186	0.039
p.Ile113Cys	substitution	113	Ile	Cys	0.867	0.025
p.Ile113Gln	substitution	113	Ile	Gln	0.235	0.110
p.Ile113Glu	substitution	113	Ile	Glu	0.000	0.861
p.Ile113Gly	substitution	113	Ile	Gly	0.073	0.122
p.Ile113His	substitution	113	Ile	His	0.000	0.564
p.Ile113Leu	substitution	113	Ile	Leu	1.062	0.016
p.Ile113Lys	substitution	113	Ile	Lys	0.000	0.022
p.Ile113Met	substitution	113	Ile	Met	0.000	0.053
p.Ile113Phe	substitution	113	Ile	Phe	0.000	0.249
p.Ile113Pro	substitution	113	Ile	Pro	0.000	0.849
p.Ile113Ser	substitution	113	Ile	Ser	0.075	0.050
p.Ile113Ter	nonsense	113	Ile	Ter	0.000	0.109
p.Ile113Thr	substitution	113	Ile	Thr	0.471	0.116
p.Ile113Trp	substitution	113	Ile	Trp	0.322	0.327
p.Ile113Tyr	substitution	113	Ile	Tyr	0.000	0.039
p.Ile113Val	substitution	113	Ile	Val	0.000	0.162
p.Ile166=	synonymous	166	Ile	NA	1.145	1.899
p.Ile166Ala	substitution	166	Ile	Ala	0.670	0.091
p.Ile166Arg	substitution	166	Ile	Arg	0.211	0.026
p.Ile166Asn	substitution	166	Ile	Asn	0.631	0.012
p.Ile166Asp	substitution	166	Ile	Asp	0.000	0.015
p.Ile166Cys	substitution	166	Ile	Cys	0.989	0.006
p.Ile166Gln	substitution	166	Ile	Gln	0.378	0.027
p.Ile166Glu	substitution	166	Ile	Glu	0.000	0.024
p.Ile166Gly	substitution	166	Ile	Gly	0.210	0.009
p.Ile166His	substitution	166	Ile	His	0.717	0.007
p.Ile166Leu	substitution	166	Ile	Leu	0.860	0.017

p.Ile166Lys	substitution	166	Ile	Lys	0.135	0.011
p.Ile166Met	substitution	166	Ile	Met	1.057	0.007
p.Ile166Phe	substitution	166	Ile	Phe	1.014	0.007
p.Ile166Pro	substitution	166	Ile	Pro	0.000	0.017
p.Ile166Ser	substitution	166	Ile	Ser	0.521	0.015
p.Ile166Ter	nonsense	166	Ile	Ter	0.050	0.015
p.Ile166Thr	substitution	166	Ile	Thr	0.418	0.048
p.Ile166Trp	substitution	166	Ile	Trp	0.955	0.012
p.Ile166Tyr	substitution	166	Ile	Tyr	0.561	0.016
p.Ile166Val	substitution	166	Ile	Val	0.483	0.086
p.Ile186=	synonymous	186	Ile	NA	0.903	0.002
p.Ile186Ala	substitution	186	Ile	Ala	0.877	0.015
p.Ile186Arg	substitution	186	Ile	Arg	0.000	0.019
p.Ile186Asn	substitution	186	Ile	Asn	0.421	0.002
p.Ile186Asp	substitution	186	Ile	Asp	0.199	0.078
p.Ile186Cys	substitution	186	Ile	Cys	0.735	0.005
p.Ile186Gln	substitution	186	Ile	Gln	0.943	0.002
p.Ile186Glu	substitution	186	Ile	Glu	1.240	0.001
p.Ile186Gly	substitution	186	Ile	Gly	0.360	0.025
p.Ile186His	substitution	186	Ile	His	0.574	0.013
p.Ile186Leu	substitution	186	Ile	Leu	0.688	0.013
p.Ile186Lys	substitution	186	Ile	Lys	0.085	0.014
p.Ile186Phe	substitution	186	Ile	Phe	0.596	0.002
p.Ile186Pro	substitution	186	Ile	Pro	0.000	0.012
p.Ile186Ser	substitution	186	Ile	Ser	0.404	0.014
p.Ile186Ter	nonsense	186	Ile	Ter	0.103	0.024
p.Ile186Thr	substitution	186	Ile	Thr	1.097	0.005
p.Ile186Trp	substitution	186	Ile	Trp	0.000	0.008
p.Ile186Tyr	substitution	186	Ile	Tyr	0.074	0.013
p.Ile186Val	substitution	186	Ile	Val	0.881	0.008
p.Ile187=	synonymous	187	Ile	NA	1.333	0.009
p.Ile187Ala	substitution	187	Ile	Ala	0.637	0.006
p.Ile187Arg	substitution	187	Ile	Arg	0.000	0.027
p.Ile187Asn	substitution	187	Ile	Asn	0.316	0.025
p.Ile187Asp	substitution	187	Ile	Asp	0.000	0.051
p.Ile187Cys	substitution	187	Ile	Cys	0.454	0.013
p.Ile187Gln	substitution	187	Ile	Gln	0.145	0.010
p.Ile187Glu	substitution	187	Ile	Glu	0.012	0.006
p.Ile187Gly	substitution	187	Ile	Gly	0.486	0.004
p.Ile187His	substitution	187	Ile	His	0.000	0.020
p.Ile187Leu	substitution	187	Ile	Leu	0.727	0.011
p.Ile187Lys	substitution	187	Ile	Lys	0.000	0.006
p.Ile187Met	substitution	187	Ile	Met	0.645	0.001

p.Ile187Phe	substitution	187	Ile	Phe	0.747	0.007
p.Ile187Pro	substitution	187	Ile	Pro	0.000	0.008
p.Ile187Ser	substitution	187	Ile	Ser	0.507	0.020
p.Ile187Ter	nonsense	187	Ile	Ter	0.004	0.005
p.Ile187Thr	substitution	187	Ile	Thr	0.270	0.142
p.Ile187Trp	substitution	187	Ile	Trp	0.000	0.010
p.Ile187Tyr	substitution	187	Ile	Tyr	0.000	0.018
p.Ile187Val	substitution	187	Ile	Val	0.730	0.015
p.Ile205=	synonymous	205	Ile	NA	1.307	0.007
p.Ile205Ala	substitution	205	Ile	Ala	0.638	0.040
p.Ile205Arg	substitution	205	Ile	Arg	0.530	0.058
p.Ile205Asn	substitution	205	Ile	Asn	0.783	0.026
p.Ile205Asp	substitution	205	Ile	Asp	1.052	0.007
p.Ile205Cys	substitution	205	Ile	Cys	1.031	0.009
p.Ile205Gln	substitution	205	Ile	Gln	1.221	0.012
p.Ile205Glu	substitution	205	Ile	Glu	1.163	0.001
p.Ile205Gly	substitution	205	Ile	Gly	0.992	0.005
p.Ile205Leu	substitution	205	Ile	Leu	1.114	0.020
p.Ile205Lys	substitution	205	Ile	Lys	1.114	0.143
p.Ile205Met	substitution	205	Ile	Met	0.767	0.002
p.Ile205Phe	substitution	205	Ile	Phe	0.000	0.218
p.Ile205Pro	substitution	205	Ile	Pro	0.000	0.063
p.Ile205Ser	substitution	205	Ile	Ser	0.451	0.077
p.Ile205Ter	nonsense	205	Ile	Ter	0.270	0.016
p.Ile205Thr	substitution	205	Ile	Thr	0.848	0.053
p.Ile205Trp	substitution	205	Ile	Trp	0.000	0.249
p.Ile205Tyr	substitution	205	Ile	Tyr	1.474	0.006
p.Ile205Val	substitution	205	Ile	Val	0.954	0.005
p.Ile21Ala	substitution	21	Ile	Ala	1.032	0.000
p.Ile21Arg	substitution	21	Ile	Arg	0.405	0.001
p.Ile21Asn	substitution	21	Ile	Asn	0.017	0.005
p.Ile21Asp	substitution	21	Ile	Asp	0.000	0.006
p.Ile21Cys	substitution	21	Ile	Cys	0.296	0.001
p.Ile21Gln	substitution	21	Ile	Gln	0.000	0.005
p.Ile21Glu	substitution	21	Ile	Glu	0.000	0.021
p.Ile21Gly	substitution	21	Ile	Gly	0.000	0.037
p.Ile21His	substitution	21	Ile	His	0.567	0.001
p.Ile21Leu	substitution	21	Ile	Leu	1.261	0.002
p.Ile21Lys	substitution	21	Ile	Lys	0.000	0.002
p.Ile21Met	substitution	21	Ile	Met	1.016	0.000
p.Ile21Phe	substitution	21	Ile	Phe	0.755	0.001
p.Ile21Pro	substitution	21	Ile	Pro	0.280	0.002
p.Ile21Ser	substitution	21	Ile	Ser	0.000	0.077

p.Ile21Ter	nonsense	21	Ile	Ter		0.000	0.059
p.Ile21Thr	substitution	21	Ile	Thr		0.702	0.004
p.Ile21Trp	substitution	21	Ile	Trp		0.819	0.000
p.Ile21Tyr	substitution	21	Ile	Tyr		0.747	0.001
p.Ile21Val	substitution	21	Ile	Val		1.010	0.002
p.Ile231=	synonymous	231	Ile	NA		0.751	0.009
p.Ile231Ala	substitution	231	Ile	Ala		1.340	0.135
p.Ile231Arg	substitution	231	Ile	Arg		0.161	0.050
p.Ile231Asn	substitution	231	Ile	Asn		0.516	0.074
p.Ile231Asp	substitution	231	Ile	Asp		0.965	0.005
p.Ile231Cys	substitution	231	Ile	Cys		0.004	0.071
p.Ile231Gln	substitution	231	Ile	Gln		0.000	0.039
p.Ile231Glu	substitution	231	Ile	Glu		0.000	0.015
p.Ile231Gly	substitution	231	Ile	Gly		0.473	0.017
p.Ile231His	substitution	231	Ile	His		0.000	0.020
p.Ile231Leu	substitution	231	Ile	Leu		0.144	0.021
p.Ile231Lys	substitution	231	Ile	Lys		0.283	0.009
p.Ile231Met	substitution	231	Ile	Met		1.043	0.014
p.Ile231Phe	substitution	231	Ile	Phe		0.000	0.038
p.Ile231Pro	substitution	231	Ile	Pro		0.000	0.021
p.Ile231Ser	substitution	231	Ile	Ser		0.787	0.026
p.Ile231Ter	nonsense	231	Ile	Ter		0.000	0.071
p.Ile231Thr	substitution	231	Ile	Thr		0.598	0.035
p.Ile231Trp	substitution	231	Ile	Trp		0.570	0.003
p.Ile231Val	substitution	231	Ile	Val		1.057	0.002
p.Ile248=	synonymous	248	Ile	NA		0.875	0.014
p.Ile248Ala	substitution	248	Ile	Ala		0.032	0.045
p.Ile248Arg	substitution	248	Ile	Arg		0.166	0.016
p.Ile248Asn	substitution	248	Ile	Asn		0.456	0.021
p.Ile248Cys	substitution	248	Ile	Cys		0.000	0.035
p.Ile248Glu	substitution	248	Ile	Glu		0.000	0.259
p.Ile248Gly	substitution	248	Ile	Gly		0.000	0.099
p.Ile248His	substitution	248	Ile	His		0.000	0.087
p.Ile248Leu	substitution	248	Ile	Leu		0.864	0.173
p.Ile248Lys	substitution	248	Ile	Lys		0.000	0.156
p.Ile248Met	substitution	248	Ile	Met		0.263	0.242
p.Ile248Phe	substitution	248	Ile	Phe		0.888	0.176
p.Ile248Pro	substitution	248	Ile	Pro		0.000	0.119
p.Ile248Ser	substitution	248	Ile	Ser		0.628	0.212
p.Ile248Ter	nonsense	248	Ile	Ter		0.000	0.202
p.Ile248Thr	substitution	248	Ile	Thr		0.272	0.097
p.Ile248Trp	substitution	248	Ile	Trp		0.287	0.034
p.Ile248Tyr	substitution	248	Ile	Tyr		0.000	0.025

p.Ile248Val	substitution	248	Ile	Val	0.576	0.069
p.Ile291=	synonymous	291	Ile	NA	1.103	0.005
p.Ile291Ala	substitution	291	Ile	Ala	1.018	0.008
p.Ile291Arg	substitution	291	Ile	Arg	1.384	0.015
p.Ile291Asn	substitution	291	Ile	Asn	0.744	0.009
p.Ile291Gln	substitution	291	Ile	Gln	1.013	0.007
p.Ile291Glu	substitution	291	Ile	Glu	1.013	0.008
p.Ile291Gly	substitution	291	Ile	Gly	0.590	0.020
p.Ile291His	substitution	291	Ile	His	0.798	0.005
p.Ile291Leu	substitution	291	Ile	Leu	0.876	0.010
p.Ile291Lys	substitution	291	Ile	Lys	1.059	0.029
p.Ile291Met	substitution	291	Ile	Met	1.067	0.005
p.Ile291Pro	substitution	291	Ile	Pro	0.751	0.008
p.Ile291Ser	substitution	291	Ile	Ser	0.556	0.026
p.Ile291Ter	nonsense	291	Ile	Ter	0.470	0.028
p.Ile291Thr	substitution	291	Ile	Thr	0.707	0.004
p.Ile291Trp	substitution	291	Ile	Trp	0.651	0.004
p.Ile291Val	substitution	291	Ile	Val	0.539	0.014
p.Ile299=	synonymous	299	Ile	NA	0.844	0.043
p.Ile299Ala	substitution	299	Ile	Ala	0.901	0.085
p.Ile299Arg	substitution	299	Ile	Arg	0.630	0.006
p.Ile299Asn	substitution	299	Ile	Asn	0.659	0.029
p.Ile299Asp	substitution	299	Ile	Asp	0.942	0.010
p.Ile299Cys	substitution	299	Ile	Cys	1.327	0.004
p.Ile299Glu	substitution	299	Ile	Glu	0.874	0.005
p.Ile299Gly	substitution	299	Ile	Gly	0.000	0.034
p.Ile299His	substitution	299	Ile	His	0.769	0.004
p.Ile299Leu	substitution	299	Ile	Leu	0.795	0.105
p.Ile299Lys	substitution	299	Ile	Lys	0.343	0.004
p.Ile299Met	substitution	299	Ile	Met	1.280	0.005
p.Ile299Phe	substitution	299	Ile	Phe	1.130	0.014
p.Ile299Ser	substitution	299	Ile	Ser	0.479	0.108
p.Ile299Ter	nonsense	299	Ile	Ter	0.000	0.010
p.Ile299Thr	substitution	299	Ile	Thr	1.121	0.008
p.Ile299Trp	substitution	299	Ile	Trp	0.223	0.029
p.Ile299Tyr	substitution	299	Ile	Tyr	0.966	0.011
p.Ile299Val	substitution	299	Ile	Val	0.961	0.112
p.Ile318=	synonymous	318	Ile	NA	1.028	0.048
p.Ile318Ala	substitution	318	Ile	Ala	0.000	0.174
p.Ile318Arg	substitution	318	Ile	Arg	0.281	0.106
p.Ile318Asn	substitution	318	Ile	Asn	0.444	0.103
p.Ile318Asp	substitution	318	Ile	Asp	0.131	0.027
p.Ile318Gln	substitution	318	Ile	Gln	0.000	0.139

p.Ile318Glu	substitution	318	Ile	Glu		0.246	0.163
p.Ile318Gly	substitution	318	Ile	Gly		0.577	0.014
p.Ile318Leu	substitution	318	Ile	Leu		0.354	0.026
p.Ile318Lys	substitution	318	Ile	Lys		1.430	0.003
p.Ile318Met	substitution	318	Ile	Met		0.658	0.009
p.Ile318Phe	substitution	318	Ile	Phe		1.218	0.013
p.Ile318Pro	substitution	318	Ile	Pro		0.000	0.133
p.Ile318Ser	substitution	318	Ile	Ser		0.843	0.076
p.Ile318Ter	nonsense	318	Ile	Ter		0.702	0.051
p.Ile318Thr	substitution	318	Ile	Thr		0.514	0.048
p.Ile318Trp	substitution	318	Ile	Trp		0.000	0.011
p.Ile318Tyr	substitution	318	Ile	Tyr		0.835	0.004
p.Ile318Val	substitution	318	Ile	Val		1.196	0.018
p.Ile323=	synonymous	323	Ile	NA		1.203	0.185
p.Ile323Ala	substitution	323	Ile	Ala		0.076	0.009
p.Ile323Arg	substitution	323	Ile	Arg		0.564	0.109
p.Ile323Asn	substitution	323	Ile	Asn		0.728	0.002
p.Ile323Asp	substitution	323	Ile	Asp		0.548	0.003
p.Ile323Cys	substitution	323	Ile	Cys		0.883	0.002
p.Ile323Gln	substitution	323	Ile	Gln		1.335	0.004
p.Ile323Glu	substitution	323	Ile	Glu		0.466	0.006
p.Ile323Gly	substitution	323	Ile	Gly		0.129	0.016
p.Ile323His	substitution	323	Ile	His		0.581	0.002
p.Ile323Leu	substitution	323	Ile	Leu		1.186	0.019
p.Ile323Lys	substitution	323	Ile	Lys		0.016	0.066
p.Ile323Met	substitution	323	Ile	Met		0.265	0.004
p.Ile323Phe	substitution	323	Ile	Phe		0.810	0.002
p.Ile323Pro	substitution	323	Ile	Pro		0.000	0.045
p.Ile323Ser	substitution	323	Ile	Ser		0.812	0.025
p.Ile323Ter	nonsense	323	Ile	Ter		0.229	0.030
p.Ile323Thr	substitution	323	Ile	Thr		0.656	0.004
p.Ile323Tyr	substitution	323	Ile	Tyr		0.820	0.002
p.Ile323Val	substitution	323	Ile	Val		0.902	0.002
p.Ile336=	synonymous	336	Ile	NA		1.224	0.003
p.Ile336Ala	substitution	336	Ile	Ala		0.592	0.004
p.Ile336Arg	substitution	336	Ile	Arg		1.351	0.005
p.Ile336Asp	substitution	336	Ile	Asp		0.000	0.126
p.Ile336Cys	substitution	336	Ile	Cys		0.000	0.037
p.Ile336Gly	substitution	336	Ile	Gly		0.000	0.070
p.Ile336Leu	substitution	336	Ile	Leu		0.271	0.014
p.Ile336Lys	substitution	336	Ile	Lys		0.000	0.038
p.Ile336Met	substitution	336	Ile	Met		0.230	0.017
p.Ile336Phe	substitution	336	Ile	Phe		0.818	0.005

p.Ile336Ser	substitution	336	Ile	Ser		1.318	0.015
p.Ile336Ter	nonsense	336	Ile	Ter		0.000	0.156
p.Ile336Thr	substitution	336	Ile	Thr		0.000	0.044
p.Ile336Trp	substitution	336	Ile	Trp		1.031	0.009
p.Ile336Tyr	substitution	336	Ile	Tyr		0.000	0.101
p.Ile336Val	substitution	336	Ile	Val		0.362	0.039
p.Ile33=	synonymous	33	Ile	NA		0.376	0.003
p.Ile33Ala	substitution	33	Ile	Ala		1.185	0.001
p.Ile33Arg	substitution	33	Ile	Arg		0.824	0.004
p.Ile33Asn	substitution	33	Ile	Asn		0.866	0.006
p.Ile33Asp	substitution	33	Ile	Asp		0.893	0.002
p.Ile33Gln	substitution	33	Ile	Gln		0.664	0.002
p.Ile33Glu	substitution	33	Ile	Glu		0.017	0.008
p.Ile33Gly	substitution	33	Ile	Gly		0.582	0.023
p.Ile33His	substitution	33	Ile	His		0.547	0.004
p.Ile33Leu	substitution	33	Ile	Leu		0.866	0.011
p.Ile33Lys	substitution	33	Ile	Lys		0.000	0.019
p.Ile33Met	substitution	33	Ile	Met		0.639	0.004
p.Ile33Phe	substitution	33	Ile	Phe		0.468	0.001
p.Ile33Pro	substitution	33	Ile	Pro		0.000	0.015
p.Ile33Ser	substitution	33	Ile	Ser		0.875	0.075
p.Ile33Ter	nonsense	33	Ile	Ter		0.000	0.012
p.Ile33Thr	substitution	33	Ile	Thr		0.956	0.018
p.Ile33Trp	substitution	33	Ile	Trp		0.944	0.006
p.Ile33Tyr	substitution	33	Ile	Tyr		1.331	0.001
p.Ile33Val	substitution	33	Ile	Val		0.678	0.001
p.Ile350=	synonymous	350	Ile	NA		0.996	0.008
p.Ile350Ala	substitution	350	Ile	Ala		0.242	0.053
p.Ile350Arg	substitution	350	Ile	Arg		0.512	0.224
p.Ile350Asn	substitution	350	Ile	Asn		0.393	0.160
p.Ile350Asp	substitution	350	Ile	Asp		0.188	0.062
p.Ile350Cys	substitution	350	Ile	Cys		0.388	0.027
p.Ile350Gln	substitution	350	Ile	Gln		0.180	0.063
p.Ile350Glu	substitution	350	Ile	Glu		0.000	0.199
p.Ile350Gly	substitution	350	Ile	Gly		0.000	0.192
p.Ile350His	substitution	350	Ile	His		1.469	0.004
p.Ile350Leu	substitution	350	Ile	Leu		0.130	0.052
p.Ile350Lys	substitution	350	Ile	Lys		0.000	0.115
p.Ile350Met	substitution	350	Ile	Met		0.711	0.251
p.Ile350Phe	substitution	350	Ile	Phe		1.199	0.040
p.Ile350Pro	substitution	350	Ile	Pro		0.000	0.082
p.Ile350Ser	substitution	350	Ile	Ser		0.142	0.027
p.Ile350Ter	nonsense	350	Ile	Ter		0.000	0.022

p.Ile350Thr	substitution	350	Ile	Thr	0.000	0.031
p.Ile350Trp	substitution	350	Ile	Trp	1.342	0.007
p.Ile350Tyr	substitution	350	Ile	Tyr	1.564	0.005
p.Ile350Val	substitution	350	Ile	Val	1.058	0.065
p.Ile53=	synonymous	53	Ile	NA	0.920	0.011
p.Ile53Ala	substitution	53	Ile	Ala	0.460	0.022
p.Ile53Arg	substitution	53	Ile	Arg	0.666	0.077
p.Ile53Asn	substitution	53	Ile	Asn	0.451	0.046
p.Ile53Asp	substitution	53	Ile	Asp	0.770	0.032
p.Ile53Cys	substitution	53	Ile	Cys	0.598	0.011
p.Ile53Gln	substitution	53	Ile	Gln	0.609	0.476
p.Ile53Glu	substitution	53	Ile	Glu	0.578	0.025
p.Ile53Gly	substitution	53	Ile	Gly	0.254	0.010
p.Ile53His	substitution	53	Ile	His	0.000	0.097
p.Ile53Leu	substitution	53	Ile	Leu	0.928	0.018
p.Ile53Lys	substitution	53	Ile	Lys	0.863	0.007
p.Ile53Met	substitution	53	Ile	Met	0.688	0.005
p.Ile53Phe	substitution	53	Ile	Phe	1.025	0.029
p.Ile53Pro	substitution	53	Ile	Pro	1.207	0.069
p.Ile53Ser	substitution	53	Ile	Ser	0.642	0.011
p.Ile53Ter	nonsense	53	Ile	Ter	0.456	0.011
p.Ile53Thr	substitution	53	Ile	Thr	0.573	0.011
p.Ile53Trp	substitution	53	Ile	Trp	0.353	0.010
p.Ile53Tyr	substitution	53	Ile	Tyr	1.056	0.005
p.Ile53Val	substitution	53	Ile	Val	0.899	0.060
p.Ile54=	synonymous	54	Ile	NA	1.039	1.346
p.Ile54Ala	substitution	54	Ile	Ala	0.537	0.024
p.Ile54Arg	substitution	54	Ile	Arg	0.707	0.005
p.Ile54Asn	substitution	54	Ile	Asn	0.738	0.009
p.Ile54Asp	substitution	54	Ile	Asp	0.429	0.009
p.Ile54Cys	substitution	54	Ile	Cys	0.060	0.022
p.Ile54Gln	substitution	54	Ile	Gln	0.031	0.014
p.Ile54Glu	substitution	54	Ile	Glu	1.124	0.004
p.Ile54Gly	substitution	54	Ile	Gly	0.383	0.009
p.Ile54His	substitution	54	Ile	His	0.300	0.009
p.Ile54Leu	substitution	54	Ile	Leu	0.803	0.007
p.Ile54Lys	substitution	54	Ile	Lys	0.909	0.007
p.Ile54Met	substitution	54	Ile	Met	0.710	0.006
p.Ile54Phe	substitution	54	Ile	Phe	0.979	0.004
p.Ile54Pro	substitution	54	Ile	Pro	0.000	0.012
p.Ile54Ser	substitution	54	Ile	Ser	0.596	0.006
p.Ile54Ter	nonsense	54	Ile	Ter	0.000	0.017
p.Ile54Thr	substitution	54	Ile	Thr	0.719	0.010

p.Ile54Trp	substitution	54	Ile	Trp		0.208	0.009
p.Ile54Tyr	substitution	54	Ile	Tyr		0.904	0.006
p.Ile54Val	substitution	54	Ile	Val		0.786	0.006
p.Ile63Ala	substitution	63	Ile	Ala		1.053	0.035
p.Ile63Arg	substitution	63	Ile	Arg		0.781	0.337
p.Ile63Asn	substitution	63	Ile	Asn		1.084	0.018
p.Ile63Asp	substitution	63	Ile	Asp		0.000	0.088
p.Ile63Cys	substitution	63	Ile	Cys		0.849	0.048
p.Ile63Gln	substitution	63	Ile	Gln		0.397	0.046
p.Ile63Glu	substitution	63	Ile	Glu		0.000	0.175
p.Ile63Gly	substitution	63	Ile	Gly		0.909	0.019
p.Ile63His	substitution	63	Ile	His		0.401	0.096
p.Ile63Leu	substitution	63	Ile	Leu		1.029	0.027
p.Ile63Lys	substitution	63	Ile	Lys		0.194	0.479
p.Ile63Met	substitution	63	Ile	Met		1.004	0.010
p.Ile63Phe	substitution	63	Ile	Phe		1.004	0.014
p.Ile63Pro	substitution	63	Ile	Pro		0.936	0.241
p.Ile63Ser	substitution	63	Ile	Ser		0.785	0.116
p.Ile63Ter	nonsense	63	Ile	Ter		0.000	0.928
p.Ile63Thr	substitution	63	Ile	Thr		0.000	0.275
p.Ile63Trp	substitution	63	Ile	Trp		0.685	0.029
p.Ile63Tyr	substitution	63	Ile	Tyr		0.372	0.053
p.Ile63Val	substitution	63	Ile	Val		0.633	0.137
p.Ile71=	synonymous	71	Ile	NA		1.315	0.159
p.Ile71Ala	substitution	71	Ile	Ala		0.000	0.044
p.Ile71Arg	substitution	71	Ile	Arg		0.289	0.055
p.Ile71Asn	substitution	71	Ile	Asn		0.821	0.009
p.Ile71Asp	substitution	71	Ile	Asp		0.547	0.023
p.Ile71Cys	substitution	71	Ile	Cys		0.769	0.009
p.Ile71Gln	substitution	71	Ile	Gln		1.561	0.013
p.Ile71Gly	substitution	71	Ile	Gly		0.720	0.009
p.Ile71His	substitution	71	Ile	His		0.657	0.012
p.Ile71Leu	substitution	71	Ile	Leu		0.979	0.011
p.Ile71Lys	substitution	71	Ile	Lys		0.929	0.015
p.Ile71Met	substitution	71	Ile	Met		1.248	0.007
p.Ile71Phe	substitution	71	Ile	Phe		0.861	0.021
p.Ile71Pro	substitution	71	Ile	Pro		0.671	0.015
p.Ile71Ser	substitution	71	Ile	Ser		0.745	0.009
p.Ile71Ter	nonsense	71	Ile	Ter		0.000	0.138
p.Ile71Thr	substitution	71	Ile	Thr		0.705	0.027
p.Ile71Trp	substitution	71	Ile	Trp		0.505	0.014
p.Ile71Tyr	substitution	71	Ile	Tyr		0.850	0.008
p.Ile71Val	substitution	71	Ile	Val		0.891	0.079

p.Leu100=	synonymous	100	Leu	NA		0.974	0.008
p.Leu100Ala	substitution	100	Leu	Ala		0.103	0.019
p.Leu100Arg	substitution	100	Leu	Arg		0.000	0.055
p.Leu100Asn	substitution	100	Leu	Asn		0.000	0.043
p.Leu100Asp	substitution	100	Leu	Asp		0.000	0.057
p.Leu100Cys	substitution	100	Leu	Cys		1.421	0.006
p.Leu100Gln	substitution	100	Leu	Gln		0.736	0.118
p.Leu100Glu	substitution	100	Leu	Glu		1.017	0.014
p.Leu100Gly	substitution	100	Leu	Gly		0.000	0.030
p.Leu100His	substitution	100	Leu	His		0.000	0.049
p.Leu100Ile	substitution	100	Leu	Ile		1.144	0.027
p.Leu100Lys	substitution	100	Leu	Lys		0.000	0.062
p.Leu100Met	substitution	100	Leu	Met		0.763	0.004
p.Leu100Phe	substitution	100	Leu	Phe		0.000	0.032
p.Leu100Pro	substitution	100	Leu	Pro		0.303	0.547
p.Leu100Ser	substitution	100	Leu	Ser		0.459	0.122
p.Leu100Ter	nonsense	100	Leu	Ter		0.000	0.095
p.Leu100Thr	substitution	100	Leu	Thr		0.487	0.093
p.Leu100Trp	substitution	100	Leu	Trp		0.304	0.020
p.Leu100Tyr	substitution	100	Leu	Tyr		0.970	0.024
p.Leu100Val	substitution	100	Leu	Val		0.638	0.088
p.Leu104=	synonymous	104	Leu	NA		0.700	0.006
p.Leu104Ala	substitution	104	Leu	Ala		0.685	0.082
p.Leu104Arg	substitution	104	Leu	Arg		0.536	0.037
p.Leu104Asn	substitution	104	Leu	Asn		0.023	0.021
p.Leu104Asp	substitution	104	Leu	Asp		0.324	0.012
p.Leu104Cys	substitution	104	Leu	Cys		0.706	0.006
p.Leu104Gln	substitution	104	Leu	Gln		0.635	0.006
p.Leu104Glu	substitution	104	Leu	Glu		0.281	0.012
p.Leu104Gly	substitution	104	Leu	Gly		0.649	0.018
p.Leu104His	substitution	104	Leu	His		0.808	0.008
p.Leu104Ile	substitution	104	Leu	Ile		1.006	0.002
p.Leu104Lys	substitution	104	Leu	Lys		0.000	0.087
p.Leu104Met	substitution	104	Leu	Met		1.059	0.006
p.Leu104Phe	substitution	104	Leu	Phe		0.782	0.003
p.Leu104Pro	substitution	104	Leu	Pro		0.664	0.014
p.Leu104Ser	substitution	104	Leu	Ser		0.781	0.014
p.Leu104Ter	nonsense	104	Leu	Ter		0.000	0.014
p.Leu104Thr	substitution	104	Leu	Thr		0.407	0.011
p.Leu104Tyr	substitution	104	Leu	Tyr		0.754	0.007
p.Leu104Val	substitution	104	Leu	Val		0.765	0.008
p.Leu134=	synonymous	134	Leu	NA		0.986	0.011
p.Leu134Ala	substitution	134	Leu	Ala		0.601	0.054

p.Leu134Arg	substitution	134	Leu	Arg	0.042	0.016
p.Leu134Asn	substitution	134	Leu	Asn	0.000	0.023
p.Leu134Asp	substitution	134	Leu	Asp	0.000	0.022
p.Leu134Cys	substitution	134	Leu	Cys	0.603	0.007
p.Leu134Gln	substitution	134	Leu	Gln	0.000	0.087
p.Leu134Glu	substitution	134	Leu	Glu	0.104	0.050
p.Leu134Gly	substitution	134	Leu	Gly	0.188	0.039
p.Leu134His	substitution	134	Leu	His	0.000	0.012
p.Leu134Ile	substitution	134	Leu	Ile	0.698	0.013
p.Leu134Lys	substitution	134	Leu	Lys	0.000	0.061
p.Leu134Met	substitution	134	Leu	Met	0.781	0.006
p.Leu134Phe	substitution	134	Leu	Phe	0.529	0.005
p.Leu134Pro	substitution	134	Leu	Pro	0.161	0.055
p.Leu134Ser	substitution	134	Leu	Ser	0.000	0.034
p.Leu134Ter	nonsense	134	Leu	Ter	0.325	0.045
p.Leu134Thr	substitution	134	Leu	Thr	0.352	0.024
p.Leu134Trp	substitution	134	Leu	Trp	0.408	0.006
p.Leu134Tyr	substitution	134	Leu	Tyr	0.000	0.016
p.Leu134Val	substitution	134	Leu	Val	0.719	0.591
p.Leu137=	synonymous	137	Leu	NA	0.990	0.010
p.Leu137Ala	substitution	137	Leu	Ala	0.414	0.023
p.Leu137Arg	substitution	137	Leu	Arg	0.261	0.031
p.Leu137Asn	substitution	137	Leu	Asn	0.210	0.006
p.Leu137Asp	substitution	137	Leu	Asp	0.073	0.013
p.Leu137Cys	substitution	137	Leu	Cys	0.460	0.003
p.Leu137Gln	substitution	137	Leu	Gln	0.364	0.004
p.Leu137Glu	substitution	137	Leu	Glu	0.009	0.010
p.Leu137Gly	substitution	137	Leu	Gly	0.042	0.002
p.Leu137His	substitution	137	Leu	His	0.314	0.009
p.Leu137Ile	substitution	137	Leu	Ile	1.178	0.003
p.Leu137Lys	substitution	137	Leu	Lys	0.000	0.009
p.Leu137Met	substitution	137	Leu	Met	0.894	0.002
p.Leu137Phe	substitution	137	Leu	Phe	0.553	0.003
p.Leu137Pro	substitution	137	Leu	Pro	0.000	0.010
p.Leu137Ser	substitution	137	Leu	Ser	0.486	0.005
p.Leu137Ter	nonsense	137	Leu	Ter	0.000	0.006
p.Leu137Thr	substitution	137	Leu	Thr	0.213	0.007
p.Leu137Trp	substitution	137	Leu	Trp	0.000	0.005
p.Leu137Tyr	substitution	137	Leu	Tyr	0.209	0.005
p.Leu137Val	substitution	137	Leu	Val	0.870	0.002
p.Leu148=	synonymous	148	Leu	NA	1.077	0.006
p.Leu148Ala	substitution	148	Leu	Ala	0.000	0.158
p.Leu148Arg	substitution	148	Leu	Arg	0.743	0.052

p.Leu148Asn	substitution	148	Leu	Asn	0.000	0.770
p.Leu148Asp	substitution	148	Leu	Asp	0.491	0.021
p.Leu148Cys	substitution	148	Leu	Cys	0.720	0.015
p.Leu148Gln	substitution	148	Leu	Gln	0.893	0.012
p.Leu148Glu	substitution	148	Leu	Glu	0.254	0.072
p.Leu148Gly	substitution	148	Leu	Gly	1.106	0.007
p.Leu148His	substitution	148	Leu	His	0.180	0.024
p.Leu148Ile	substitution	148	Leu	Ile	1.056	0.010
p.Leu148Lys	substitution	148	Leu	Lys	1.139	0.008
p.Leu148Met	substitution	148	Leu	Met	1.195	0.008
p.Leu148Phe	substitution	148	Leu	Phe	1.103	0.011
p.Leu148Pro	substitution	148	Leu	Pro	1.521	0.105
p.Leu148Ser	substitution	148	Leu	Ser	0.492	0.135
p.Leu148Ter	nonsense	148	Leu	Ter	0.000	0.017
p.Leu148Thr	substitution	148	Leu	Thr	0.519	0.256
p.Leu148Trp	substitution	148	Leu	Trp	0.459	0.012
p.Leu148Tyr	substitution	148	Leu	Tyr	0.000	0.014
p.Leu148Val	substitution	148	Leu	Val	0.905	0.004
p.Leu154=	synonymous	154	Leu	NA	0.972	0.009
p.Leu154Ala	substitution	154	Leu	Ala	0.429	0.069
p.Leu154Arg	substitution	154	Leu	Arg	0.197	0.012
p.Leu154Asn	substitution	154	Leu	Asn	0.209	0.010
p.Leu154Asp	substitution	154	Leu	Asp	0.302	0.022
p.Leu154Cys	substitution	154	Leu	Cys	0.988	0.005
p.Leu154Gln	substitution	154	Leu	Gln	0.695	0.012
p.Leu154Glu	substitution	154	Leu	Glu	0.000	0.007
p.Leu154Gly	substitution	154	Leu	Gly	0.804	0.003
p.Leu154His	substitution	154	Leu	His	0.752	0.005
p.Leu154Ile	substitution	154	Leu	Ile	0.335	0.021
p.Leu154Lys	substitution	154	Leu	Lys	0.279	0.009
p.Leu154Met	substitution	154	Leu	Met	0.764	0.010
p.Leu154Phe	substitution	154	Leu	Phe	0.527	0.007
p.Leu154Pro	substitution	154	Leu	Pro	0.000	0.047
p.Leu154Ser	substitution	154	Leu	Ser	0.459	0.070
p.Leu154Ter	nonsense	154	Leu	Ter	0.000	0.030
p.Leu154Thr	substitution	154	Leu	Thr	0.607	0.018
p.Leu154Trp	substitution	154	Leu	Trp	0.513	0.007
p.Leu154Tyr	substitution	154	Leu	Tyr	0.489	0.007
p.Leu154Val	substitution	154	Leu	Val	0.807	0.010
p.Leu161=	synonymous	161	Leu	NA	1.017	0.018
p.Leu161Ala	substitution	161	Leu	Ala	0.348	0.013
p.Leu161Arg	substitution	161	Leu	Arg	0.000	0.032
p.Leu161Asn	substitution	161	Leu	Asn	0.131	0.035

p.Leu161Asp	substitution	161	Leu	Asp	0.000	0.045
p.Leu161Cys	substitution	161	Leu	Cys	0.979	0.005
p.Leu161Gln	substitution	161	Leu	Gln	0.979	0.014
p.Leu161Glu	substitution	161	Leu	Glu	0.000	0.019
p.Leu161Gly	substitution	161	Leu	Gly	0.166	0.009
p.Leu161His	substitution	161	Leu	His	0.873	0.006
p.Leu161Ile	substitution	161	Leu	Ile	0.749	0.009
p.Leu161Lys	substitution	161	Leu	Lys	0.696	0.007
p.Leu161Met	substitution	161	Leu	Met	0.951	0.008
p.Leu161Phe	substitution	161	Leu	Phe	0.552	0.010
p.Leu161Pro	substitution	161	Leu	Pro	0.000	0.043
p.Leu161Ser	substitution	161	Leu	Ser	0.475	0.012
p.Leu161Ter	nonsense	161	Leu	Ter	0.025	0.010
p.Leu161Thr	substitution	161	Leu	Thr	0.656	0.022
p.Leu161Trp	substitution	161	Leu	Trp	0.519	0.007
p.Leu161Tyr	substitution	161	Leu	Tyr	0.902	0.006
p.Leu161Val	substitution	161	Leu	Val	0.568	0.008
p.Leu170=	synonymous	170	Leu	NA	0.918	0.008
p.Leu170Ala	substitution	170	Leu	Ala	0.551	0.037
p.Leu170Arg	substitution	170	Leu	Arg	0.000	0.018
p.Leu170Asn	substitution	170	Leu	Asn	0.781	0.019
p.Leu170Asp	substitution	170	Leu	Asp	0.000	0.045
p.Leu170Cys	substitution	170	Leu	Cys	0.691	0.014
p.Leu170Gln	substitution	170	Leu	Gln	1.097	0.007
p.Leu170Glu	substitution	170	Leu	Glu	0.527	0.019
p.Leu170Gly	substitution	170	Leu	Gly	0.958	0.031
p.Leu170His	substitution	170	Leu	His	0.720	0.040
p.Leu170Ile	substitution	170	Leu	Ile	0.790	0.042
p.Leu170Lys	substitution	170	Leu	Lys	0.038	0.027
p.Leu170Met	substitution	170	Leu	Met	1.074	0.008
p.Leu170Phe	substitution	170	Leu	Phe	0.904	0.008
p.Leu170Pro	substitution	170	Leu	Pro	0.000	0.014
p.Leu170Ser	substitution	170	Leu	Ser	0.520	0.034
p.Leu170Ter	nonsense	170	Leu	Ter	0.042	0.009
p.Leu170Thr	substitution	170	Leu	Thr	0.589	0.030
p.Leu170Trp	substitution	170	Leu	Trp	0.000	0.018
p.Leu170Tyr	substitution	170	Leu	Tyr	1.333	0.012
p.Leu170Val	substitution	170	Leu	Val	0.848	0.012
p.Leu174=	synonymous	174	Leu	NA	0.987	0.010
p.Leu174Ala	substitution	174	Leu	Ala	0.454	0.019
p.Leu174Arg	substitution	174	Leu	Arg	0.659	0.019
p.Leu174Asn	substitution	174	Leu	Asn	0.208	0.007
p.Leu174Asp	substitution	174	Leu	Asp	0.000	0.007

p.Leu174Cys	substitution	174	Leu	Cys	0.960	0.004
p.Leu174Gln	substitution	174	Leu	Gln	0.394	0.009
p.Leu174Glu	substitution	174	Leu	Glu	0.142	0.013
p.Leu174Gly	substitution	174	Leu	Gly	0.215	0.014
p.Leu174His	substitution	174	Leu	His	0.688	0.007
p.Leu174Ile	substitution	174	Leu	Ile	0.944	0.001
p.Leu174Met	substitution	174	Leu	Met	1.127	0.005
p.Leu174Phe	substitution	174	Leu	Phe	1.171	0.005
p.Leu174Pro	substitution	174	Leu	Pro	0.000	0.032
p.Leu174Ser	substitution	174	Leu	Ser	0.415	0.007
p.Leu174Ter	nonsense	174	Leu	Ter	0.000	0.012
p.Leu174Thr	substitution	174	Leu	Thr	0.588	0.049
p.Leu174Trp	substitution	174	Leu	Trp	0.404	0.010
p.Leu174Tyr	substitution	174	Leu	Tyr	0.695	0.007
p.Leu174Val	substitution	174	Leu	Val	0.814	0.017
p.Leu177=	synonymous	177	Leu	NA	1.116	0.014
p.Leu177Ala	substitution	177	Leu	Ala	0.294	0.007
p.Leu177Arg	substitution	177	Leu	Arg	0.121	0.007
p.Leu177Asn	substitution	177	Leu	Asn	0.554	0.011
p.Leu177Asp	substitution	177	Leu	Asp	0.169	0.035
p.Leu177Cys	substitution	177	Leu	Cys	0.509	0.007
p.Leu177Gln	substitution	177	Leu	Gln	0.284	0.009
p.Leu177Glu	substitution	177	Leu	Glu	0.000	0.009
p.Leu177Gly	substitution	177	Leu	Gly	0.000	0.008
p.Leu177His	substitution	177	Leu	His	0.000	0.010
p.Leu177Ile	substitution	177	Leu	Ile	0.527	0.012
p.Leu177Lys	substitution	177	Leu	Lys	0.000	0.015
p.Leu177Met	substitution	177	Leu	Met	0.988	0.006
p.Leu177Phe	substitution	177	Leu	Phe	0.354	0.005
p.Leu177Pro	substitution	177	Leu	Pro	0.000	0.751
p.Leu177Ser	substitution	177	Leu	Ser	0.000	0.003
p.Leu177Ter	nonsense	177	Leu	Ter	0.000	0.020
p.Leu177Thr	substitution	177	Leu	Thr	0.481	0.009
p.Leu177Trp	substitution	177	Leu	Trp	0.000	0.023
p.Leu177Tyr	substitution	177	Leu	Tyr	0.000	0.014
p.Leu177Val	substitution	177	Leu	Val	0.953	0.027
p.Leu188=	synonymous	188	Leu	NA	0.988	0.016
p.Leu188Ala	substitution	188	Leu	Ala	0.872	0.015
p.Leu188Arg	substitution	188	Leu	Arg	0.000	0.003
p.Leu188Asn	substitution	188	Leu	Asn	0.450	0.035
p.Leu188Asp	substitution	188	Leu	Asp	0.000	0.017
p.Leu188Cys	substitution	188	Leu	Cys	0.893	0.002
p.Leu188Gln	substitution	188	Leu	Gln	0.496	0.009

p.Leu188Glu	substitution	188	Leu	Glu	0.000	0.009
p.Leu188Gly	substitution	188	Leu	Gly	0.729	0.031
p.Leu188His	substitution	188	Leu	His	0.956	0.001
p.Leu188Ile	substitution	188	Leu	Ile	0.063	0.006
p.Leu188Lys	substitution	188	Leu	Lys	0.000	0.013
p.Leu188Met	substitution	188	Leu	Met	0.673	0.005
p.Leu188Phe	substitution	188	Leu	Phe	0.649	0.004
p.Leu188Pro	substitution	188	Leu	Pro	0.240	0.018
p.Leu188Ser	substitution	188	Leu	Ser	0.425	0.064
p.Leu188Ter	nonsense	188	Leu	Ter	0.231	0.005
p.Leu188Thr	substitution	188	Leu	Thr	0.845	0.040
p.Leu188Trp	substitution	188	Leu	Trp	0.000	0.008
p.Leu188Tyr	substitution	188	Leu	Tyr	0.000	0.018
p.Leu188Val	substitution	188	Leu	Val	0.000	0.014
p.Leu193=	synonymous	193	Leu	NA	0.933	0.016
p.Leu193Ala	substitution	193	Leu	Ala	0.000	0.062
p.Leu193Arg	substitution	193	Leu	Arg	0.000	0.004
p.Leu193Asn	substitution	193	Leu	Asn	0.451	0.016
p.Leu193Asp	substitution	193	Leu	Asp	0.000	0.011
p.Leu193Cys	substitution	193	Leu	Cys	0.000	0.019
p.Leu193Gln	substitution	193	Leu	Gln	0.522	0.006
p.Leu193Glu	substitution	193	Leu	Glu	0.000	0.021
p.Leu193Gly	substitution	193	Leu	Gly	1.029	0.002
p.Leu193His	substitution	193	Leu	His	0.123	0.008
p.Leu193Ile	substitution	193	Leu	Ile	0.292	0.010
p.Leu193Lys	substitution	193	Leu	Lys	0.000	0.021
p.Leu193Met	substitution	193	Leu	Met	1.094	0.001
p.Leu193Phe	substitution	193	Leu	Phe	0.424	0.009
p.Leu193Pro	substitution	193	Leu	Pro	0.622	0.170
p.Leu193Ser	substitution	193	Leu	Ser	0.637	0.007
p.Leu193Ter	nonsense	193	Leu	Ter	0.033	0.056
p.Leu193Thr	substitution	193	Leu	Thr	0.361	0.041
p.Leu193Trp	substitution	193	Leu	Trp	0.000	0.021
p.Leu193Tyr	substitution	193	Leu	Tyr	0.000	0.037
p.Leu193Val	substitution	193	Leu	Val	0.272	0.047
p.Leu206=	synonymous	206	Leu	NA	0.708	0.017
p.Leu206Ala	substitution	206	Leu	Ala	0.000	0.016
p.Leu206Arg	substitution	206	Leu	Arg	0.032	0.002
p.Leu206Asn	substitution	206	Leu	Asn	0.000	0.062
p.Leu206Asp	substitution	206	Leu	Asp	0.273	0.005
p.Leu206Cys	substitution	206	Leu	Cys	0.790	0.001
p.Leu206Gln	substitution	206	Leu	Gln	0.059	0.014
p.Leu206Glu	substitution	206	Leu	Glu	0.000	0.006

p.Leu206Gly	substitution	206	Leu	Gly	0.449	0.016
p.Leu206His	substitution	206	Leu	His	0.000	0.101
p.Leu206Ile	substitution	206	Leu	Ile	0.027	0.008
p.Leu206Lys	substitution	206	Leu	Lys	0.000	0.007
p.Leu206Met	substitution	206	Leu	Met	1.129	0.002
p.Leu206Phe	substitution	206	Leu	Phe	0.950	0.001
p.Leu206Pro	substitution	206	Leu	Pro	0.285	0.120
p.Leu206Ser	substitution	206	Leu	Ser	0.176	0.018
p.Leu206Ter	nonsense	206	Leu	Ter	0.000	0.022
p.Leu206Thr	substitution	206	Leu	Thr	0.432	0.193
p.Leu206Trp	substitution	206	Leu	Trp	0.207	0.002
p.Leu206Tyr	substitution	206	Leu	Tyr	0.000	0.014
p.Leu206Val	substitution	206	Leu	Val	0.499	0.152
p.Leu220=	synonymous	220	Leu	NA	0.755	0.036
p.Leu220Ala	substitution	220	Leu	Ala	1.124	0.015
p.Leu220Arg	substitution	220	Leu	Arg	0.042	0.014
p.Leu220Asn	substitution	220	Leu	Asn	0.004	0.019
p.Leu220Asp	substitution	220	Leu	Asp	0.149	0.031
p.Leu220Cys	substitution	220	Leu	Cys	0.762	0.004
p.Leu220Gln	substitution	220	Leu	Gln	0.000	0.008
p.Leu220Glu	substitution	220	Leu	Glu	0.000	0.009
p.Leu220Gly	substitution	220	Leu	Gly	0.088	0.049
p.Leu220His	substitution	220	Leu	His	0.059	0.009
p.Leu220Ile	substitution	220	Leu	Ile	0.000	0.060
p.Leu220Met	substitution	220	Leu	Met	1.001	0.003
p.Leu220Phe	substitution	220	Leu	Phe	0.475	0.012
p.Leu220Pro	substitution	220	Leu	Pro	0.000	0.076
p.Leu220Ser	substitution	220	Leu	Ser	0.399	0.144
p.Leu220Ter	nonsense	220	Leu	Ter	0.000	0.014
p.Leu220Thr	substitution	220	Leu	Thr	0.881	0.030
p.Leu220Trp	substitution	220	Leu	Trp	0.320	0.007
p.Leu220Tyr	substitution	220	Leu	Tyr	0.721	0.005
p.Leu220Val	substitution	220	Leu	Val	0.886	0.011
p.Leu232=	synonymous	232	Leu	NA	1.439	0.001
p.Leu232Ala	substitution	232	Leu	Ala	0.608	0.043
p.Leu232Arg	substitution	232	Leu	Arg	0.218	0.058
p.Leu232Asn	substitution	232	Leu	Asn	1.049	0.003
p.Leu232Asp	substitution	232	Leu	Asp	0.354	0.008
p.Leu232Cys	substitution	232	Leu	Cys	0.889	0.003
p.Leu232Gln	substitution	232	Leu	Gln	0.000	0.013
p.Leu232Glu	substitution	232	Leu	Glu	0.184	0.010
p.Leu232Gly	substitution	232	Leu	Gly	1.006	0.004
p.Leu232His	substitution	232	Leu	His	0.511	0.005

p.Leu232Ile	substitution	232	Leu	Ile	1.100	0.004
p.Leu232Lys	substitution	232	Leu	Lys	0.999	0.003
p.Leu232Met	substitution	232	Leu	Met	0.910	0.003
p.Leu232Phe	substitution	232	Leu	Phe	0.899	0.009
p.Leu232Pro	substitution	232	Leu	Pro	0.394	0.026
p.Leu232Ser	substitution	232	Leu	Ser	0.139	0.052
p.Leu232Ter	nonsense	232	Leu	Ter	0.384	0.011
p.Leu232Thr	substitution	232	Leu	Thr	0.980	0.001
p.Leu232Trp	substitution	232	Leu	Trp	0.645	0.002
p.Leu232Tyr	substitution	232	Leu	Tyr	0.278	0.052
p.Leu232Val	substitution	232	Leu	Val	1.133	0.033
p.Leu234=	synonymous	234	Leu	NA	0.962	0.011
p.Leu234Ala	substitution	234	Leu	Ala	1.135	0.002
p.Leu234Arg	substitution	234	Leu	Arg	0.569	0.223
p.Leu234Asn	substitution	234	Leu	Asn	1.180	0.003
p.Leu234Asp	substitution	234	Leu	Asp	0.226	0.019
p.Leu234Cys	substitution	234	Leu	Cys	0.909	0.002
p.Leu234Gln	substitution	234	Leu	Gln	0.665	0.006
p.Leu234Glu	substitution	234	Leu	Glu	0.000	0.010
p.Leu234Gly	substitution	234	Leu	Gly	0.110	0.011
p.Leu234His	substitution	234	Leu	His	0.609	0.012
p.Leu234Lys	substitution	234	Leu	Lys	0.939	0.004
p.Leu234Met	substitution	234	Leu	Met	0.501	0.005
p.Leu234Phe	substitution	234	Leu	Phe	0.593	0.003
p.Leu234Pro	substitution	234	Leu	Pro	0.000	0.068
p.Leu234Ser	substitution	234	Leu	Ser	0.047	0.027
p.Leu234Ter	nonsense	234	Leu	Ter	0.245	0.074
p.Leu234Thr	substitution	234	Leu	Thr	0.437	0.067
p.Leu234Trp	substitution	234	Leu	Trp	0.894	0.002
p.Leu234Tyr	substitution	234	Leu	Tyr	0.000	0.067
p.Leu234Val	substitution	234	Leu	Val	1.091	0.016
p.Leu238=	synonymous	238	Leu	NA	0.919	0.008
p.Leu238Ala	substitution	238	Leu	Ala	0.000	0.075
p.Leu238Arg	substitution	238	Leu	Arg	0.000	0.021
p.Leu238Asn	substitution	238	Leu	Asn	0.000	0.117
p.Leu238Asp	substitution	238	Leu	Asp	0.883	0.049
p.Leu238Cys	substitution	238	Leu	Cys	0.098	0.228
p.Leu238Gln	substitution	238	Leu	Gln	0.565	0.043
p.Leu238Glu	substitution	238	Leu	Glu	0.048	0.015
p.Leu238Gly	substitution	238	Leu	Gly	0.000	0.096
p.Leu238His	substitution	238	Leu	His	0.000	0.139
p.Leu238Lys	substitution	238	Leu	Lys	0.000	0.257
p.Leu238Met	substitution	238	Leu	Met	1.236	0.001

p.Leu238Phe	substitution	238	Leu	Phe		0.000	0.014
p.Leu238Pro	substitution	238	Leu	Pro		0.555	0.066
p.Leu238Ser	substitution	238	Leu	Ser		0.127	0.013
p.Leu238Ter	nonsense	238	Leu	Ter		0.000	0.125
p.Leu238Thr	substitution	238	Leu	Thr		0.000	0.145
p.Leu238Trp	substitution	238	Leu	Trp		0.098	0.009
p.Leu238Val	substitution	238	Leu	Val		0.583	0.047
p.Leu244=	synonymous	244	Leu	NA		0.990	0.014
p.Leu244Ala	substitution	244	Leu	Ala		0.123	0.031
p.Leu244Arg	substitution	244	Leu	Arg		0.088	0.007
p.Leu244Asn	substitution	244	Leu	Asn		0.298	0.015
p.Leu244Asp	substitution	244	Leu	Asp		0.000	0.200
p.Leu244Cys	substitution	244	Leu	Cys		0.969	0.003
p.Leu244Gln	substitution	244	Leu	Gln		1.413	0.003
p.Leu244Glu	substitution	244	Leu	Glu		0.000	0.078
p.Leu244Gly	substitution	244	Leu	Gly		0.000	0.040
p.Leu244His	substitution	244	Leu	His		0.082	0.012
p.Leu244Ile	substitution	244	Leu	Ile		0.000	0.011
p.Leu244Lys	substitution	244	Leu	Lys		0.000	0.021
p.Leu244Met	substitution	244	Leu	Met		0.719	0.005
p.Leu244Phe	substitution	244	Leu	Phe		0.000	0.006
p.Leu244Pro	substitution	244	Leu	Pro		0.142	0.035
p.Leu244Ser	substitution	244	Leu	Ser		1.131	0.137
p.Leu244Ter	nonsense	244	Leu	Ter		0.273	0.051
p.Leu244Thr	substitution	244	Leu	Thr		1.106	0.152
p.Leu244Trp	substitution	244	Leu	Trp		0.000	0.014
p.Leu244Tyr	substitution	244	Leu	Tyr		1.182	0.002
p.Leu244Val	substitution	244	Leu	Val		0.748	0.172
p.Leu245=	synonymous	245	Leu	NA		0.869	0.057
p.Leu245Ala	substitution	245	Leu	Ala		1.132	0.054
p.Leu245Arg	substitution	245	Leu	Arg		0.031	0.026
p.Leu245Asp	substitution	245	Leu	Asp		0.000	0.054
p.Leu245Cys	substitution	245	Leu	Cys		0.847	0.003
p.Leu245Gln	substitution	245	Leu	Gln		0.726	0.003
p.Leu245Glu	substitution	245	Leu	Glu		0.085	0.004
p.Leu245Gly	substitution	245	Leu	Gly		0.226	0.067
p.Leu245His	substitution	245	Leu	His		0.719	0.038
p.Leu245Ile	substitution	245	Leu	Ile		0.963	0.002
p.Leu245Lys	substitution	245	Leu	Lys		0.143	0.013
p.Leu245Met	substitution	245	Leu	Met		1.159	0.007
p.Leu245Phe	substitution	245	Leu	Phe		0.439	0.071
p.Leu245Pro	substitution	245	Leu	Pro		0.000	0.130
p.Leu245Ser	substitution	245	Leu	Ser		0.000	0.030

p.Leu245Thr	substitution	245	Leu	Thr	0.000	0.025
p.Leu245Trp	substitution	245	Leu	Trp	0.807	0.003
p.Leu245Tyr	substitution	245	Leu	Tyr	1.185	0.014
p.Leu245Val	substitution	245	Leu	Val	0.754	0.079
p.Leu254=	synonymous	254	Leu	NA	0.941	0.078
p.Leu254Ala	substitution	254	Leu	Ala	0.000	0.014
p.Leu254Arg	substitution	254	Leu	Arg	0.069	0.221
p.Leu254Cys	substitution	254	Leu	Cys	0.000	0.038
p.Leu254Gln	substitution	254	Leu	Gln	0.967	0.009
p.Leu254Gly	substitution	254	Leu	Gly	0.000	0.082
p.Leu254Ile	substitution	254	Leu	Ile	0.000	0.025
p.Leu254Lys	substitution	254	Leu	Lys	0.000	0.072
p.Leu254Met	substitution	254	Leu	Met	0.720	0.008
p.Leu254Phe	substitution	254	Leu	Phe	0.000	0.093
p.Leu254Pro	substitution	254	Leu	Pro	0.432	0.664
p.Leu254Ser	substitution	254	Leu	Ser	0.445	0.037
p.Leu254Ter	nonsense	254	Leu	Ter	0.000	0.132
p.Leu254Trp	substitution	254	Leu	Trp	1.293	0.002
p.Leu254Tyr	substitution	254	Leu	Tyr	0.000	0.164
p.Leu254Val	substitution	254	Leu	Val	0.845	0.158
p.Leu257=	synonymous	257	Leu	NA	2.078	0.123
p.Leu257Arg	substitution	257	Leu	Arg	1.340	0.162
p.Leu257Met	substitution	257	Leu	Met	0.963	0.005
p.Leu257Val	substitution	257	Leu	Val	1.129	0.030
p.Leu276=	synonymous	276	Leu	NA	0.935	0.034
p.Leu276Ala	substitution	276	Leu	Ala	0.193	0.026
p.Leu276Arg	substitution	276	Leu	Arg	0.000	0.017
p.Leu276Asn	substitution	276	Leu	Asn	0.000	0.145
p.Leu276Asp	substitution	276	Leu	Asp	0.039	0.009
p.Leu276Cys	substitution	276	Leu	Cys	0.853	0.006
p.Leu276Gln	substitution	276	Leu	Gln	0.207	0.239
p.Leu276Glu	substitution	276	Leu	Glu	0.008	0.015
p.Leu276Gly	substitution	276	Leu	Gly	0.307	0.084
p.Leu276His	substitution	276	Leu	His	0.000	0.017
p.Leu276Ile	substitution	276	Leu	Ile	0.316	0.042
p.Leu276Lys	substitution	276	Leu	Lys	0.000	0.012
p.Leu276Met	substitution	276	Leu	Met	1.189	0.005
p.Leu276Phe	substitution	276	Leu	Phe	0.156	0.005
p.Leu276Pro	substitution	276	Leu	Pro	0.000	0.088
p.Leu276Ser	substitution	276	Leu	Ser	0.014	0.012
p.Leu276Ter	nonsense	276	Leu	Ter	0.000	0.010
p.Leu276Thr	substitution	276	Leu	Thr	0.000	0.084
p.Leu276Trp	substitution	276	Leu	Trp	0.000	0.021

p.Leu276Tyr	substitution	276	Leu	Tyr	1.034	0.004
p.Leu276Val	substitution	276	Leu	Val	1.066	0.004
p.Leu278=	synonymous	278	Leu	NA	1.046	0.016
p.Leu278Ala	substitution	278	Leu	Ala	0.302	0.007
p.Leu278Arg	substitution	278	Leu	Arg	0.094	0.009
p.Leu278Asn	substitution	278	Leu	Asn	0.000	0.016
p.Leu278Asp	substitution	278	Leu	Asp	0.000	0.060
p.Leu278Cys	substitution	278	Leu	Cys	0.967	0.005
p.Leu278Gln	substitution	278	Leu	Gln	1.371	0.010
p.Leu278Glu	substitution	278	Leu	Glu	0.000	0.016
p.Leu278Gly	substitution	278	Leu	Gly	0.745	0.075
p.Leu278His	substitution	278	Leu	His	0.025	0.207
p.Leu278Lys	substitution	278	Leu	Lys	0.000	0.009
p.Leu278Met	substitution	278	Leu	Met	0.449	0.010
p.Leu278Phe	substitution	278	Leu	Phe	0.765	0.013
p.Leu278Pro	substitution	278	Leu	Pro	0.587	0.096
p.Leu278Ser	substitution	278	Leu	Ser	0.543	0.015
p.Leu278Ter	nonsense	278	Leu	Ter	0.000	0.011
p.Leu278Thr	substitution	278	Leu	Thr	0.483	0.115
p.Leu278Trp	substitution	278	Leu	Trp	0.162	0.007
p.Leu278Tyr	substitution	278	Leu	Tyr	0.000	0.018
p.Leu278Val	substitution	278	Leu	Val	0.381	0.041
p.Leu285=	synonymous	285	Leu	NA	0.932	0.011
p.Leu285Ala	substitution	285	Leu	Ala	1.003	0.011
p.Leu285Arg	substitution	285	Leu	Arg	0.802	0.011
p.Leu285Asn	substitution	285	Leu	Asn	1.418	0.004
p.Leu285Asp	substitution	285	Leu	Asp	1.174	0.006
p.Leu285Cys	substitution	285	Leu	Cys	0.471	0.004
p.Leu285Gln	substitution	285	Leu	Gln	0.595	0.040
p.Leu285Glu	substitution	285	Leu	Glu	0.238	0.055
p.Leu285Gly	substitution	285	Leu	Gly	1.211	0.006
p.Leu285His	substitution	285	Leu	His	0.648	0.004
p.Leu285Ile	substitution	285	Leu	Ile	0.766	0.011
p.Leu285Lys	substitution	285	Leu	Lys	0.739	0.042
p.Leu285Met	substitution	285	Leu	Met	0.962	0.004
p.Leu285Phe	substitution	285	Leu	Phe	1.076	0.002
p.Leu285Pro	substitution	285	Leu	Pro	1.171	0.004
p.Leu285Ser	substitution	285	Leu	Ser	0.000	0.048
p.Leu285Ter	nonsense	285	Leu	Ter	0.101	0.096
p.Leu285Thr	substitution	285	Leu	Thr	0.794	0.032
p.Leu285Trp	substitution	285	Leu	Trp	0.620	0.005
p.Leu285Tyr	substitution	285	Leu	Tyr	1.216	0.004
p.Leu285Val	substitution	285	Leu	Val	0.778	0.013

p.Leu30=	synonymous	30	Leu	NA	0.935	0.000
p.Leu30Ala	substitution	30	Leu	Ala	0.000	0.002
p.Leu30Arg	substitution	30	Leu	Arg	0.000	0.001
p.Leu30Asn	substitution	30	Leu	Asn	0.000	0.002
p.Leu30Asp	substitution	30	Leu	Asp	0.000	0.003
p.Leu30Cys	substitution	30	Leu	Cys	0.842	0.000
p.Leu30Gln	substitution	30	Leu	Gln	0.000	0.004
p.Leu30Glu	substitution	30	Leu	Glu	0.000	0.001
p.Leu30Gly	substitution	30	Leu	Gly	0.000	0.001
p.Leu30His	substitution	30	Leu	His	0.000	0.002
p.Leu30Ile	substitution	30	Leu	Ile	0.000	0.001
p.Leu30Lys	substitution	30	Leu	Lys	0.000	0.002
p.Leu30Met	substitution	30	Leu	Met	0.570	0.000
p.Leu30Phe	substitution	30	Leu	Phe	0.000	0.002
p.Leu30Pro	substitution	30	Leu	Pro	0.000	0.004
p.Leu30Ser	substitution	30	Leu	Ser	0.000	0.005
p.Leu30Ter	nonsense	30	Leu	Ter	0.000	0.003
p.Leu30Thr	substitution	30	Leu	Thr	0.000	0.001
p.Leu30Trp	substitution	30	Leu	Trp	0.000	0.001
p.Leu30Tyr	substitution	30	Leu	Tyr	0.208	0.001
p.Leu30Val	substitution	30	Leu	Val	0.616	0.004
p.Leu315=	synonymous	315	Leu	NA	0.925	0.063
p.Leu315Ala	substitution	315	Leu	Ala	0.502	0.204
p.Leu315Arg	substitution	315	Leu	Arg	0.161	0.308
p.Leu315Asn	substitution	315	Leu	Asn	0.204	0.018
p.Leu315Asp	substitution	315	Leu	Asp	0.109	0.011
p.Leu315Cys	substitution	315	Leu	Cys	1.428	0.004
p.Leu315Gln	substitution	315	Leu	Gln	0.068	0.035
p.Leu315Glu	substitution	315	Leu	Glu	0.000	0.024
p.Leu315Gly	substitution	315	Leu	Gly	0.264	0.104
p.Leu315Ile	substitution	315	Leu	Ile	0.000	0.023
p.Leu315Lys	substitution	315	Leu	Lys	0.557	0.018
p.Leu315Met	substitution	315	Leu	Met	0.731	0.006
p.Leu315Phe	substitution	315	Leu	Phe	1.254	0.002
p.Leu315Pro	substitution	315	Leu	Pro	0.754	0.245
p.Leu315Ser	substitution	315	Leu	Ser	1.229	0.117
p.Leu315Ter	nonsense	315	Leu	Ter	0.219	0.134
p.Leu315Thr	substitution	315	Leu	Thr	0.029	0.191
p.Leu315Trp	substitution	315	Leu	Trp	0.813	0.005
p.Leu315Tyr	substitution	315	Leu	Tyr	0.000	0.035
p.Leu315Val	substitution	315	Leu	Val	0.079	0.127
p.Leu329=	synonymous	329	Leu	NA	1.310	0.080
p.Leu329Arg	substitution	329	Leu	Arg	1.383	0.026

p.Leu329Asp	substitution	329	Leu	Asp		0.753	0.086
p.Leu329Cys	substitution	329	Leu	Cys		0.000	0.080
p.Leu329Gln	substitution	329	Leu	Gln		1.218	0.007
p.Leu329Glu	substitution	329	Leu	Glu		0.000	0.016
p.Leu329Ile	substitution	329	Leu	Ile		0.987	0.189
p.Leu329Met	substitution	329	Leu	Met		1.493	0.038
p.Leu329Phe	substitution	329	Leu	Phe		0.891	0.002
p.Leu329Pro	substitution	329	Leu	Pro		0.000	0.170
p.Leu329Ser	substitution	329	Leu	Ser		1.011	0.022
p.Leu329Ter	nonsense	329	Leu	Ter		0.934	0.305
p.Leu329Thr	substitution	329	Leu	Thr		1.232	0.324
p.Leu329Trp	substitution	329	Leu	Trp		0.243	0.028
p.Leu329Tyr	substitution	329	Leu	Tyr		0.348	0.016
p.Leu329Val	substitution	329	Leu	Val		1.365	0.025
p.Leu334=	synonymous	334	Leu	NA		1.439	0.007
p.Leu334Arg	substitution	334	Leu	Arg		0.000	0.033
p.Leu334Asn	substitution	334	Leu	Asn		1.903	0.010
p.Leu334Cys	substitution	334	Leu	Cys		1.071	0.004
p.Leu334Gln	substitution	334	Leu	Gln		0.000	0.019
p.Leu334Gly	substitution	334	Leu	Gly		1.296	0.011
p.Leu334His	substitution	334	Leu	His		0.000	0.019
p.Leu334Lys	substitution	334	Leu	Lys		0.000	0.016
p.Leu334Met	substitution	334	Leu	Met		1.058	0.005
p.Leu334Phe	substitution	334	Leu	Phe		0.821	0.036
p.Leu334Ser	substitution	334	Leu	Ser		0.509	0.225
p.Leu334Ter	nonsense	334	Leu	Ter		0.535	0.014
p.Leu334Thr	substitution	334	Leu	Thr		0.000	0.143
p.Leu334Trp	substitution	334	Leu	Trp		0.604	0.013
p.Leu334Tyr	substitution	334	Leu	Tyr		0.340	0.020
p.Leu334Val	substitution	334	Leu	Val		1.547	0.014
p.Leu338=	synonymous	338	Leu	NA		1.025	0.024
p.Leu338Ala	substitution	338	Leu	Ala		1.212	0.005
p.Leu338Arg	substitution	338	Leu	Arg		0.100	0.081
p.Leu338Asp	substitution	338	Leu	Asp		0.000	0.104
p.Leu338Cys	substitution	338	Leu	Cys		0.632	0.011
p.Leu338Gln	substitution	338	Leu	Gln		0.129	0.233
p.Leu338Gly	substitution	338	Leu	Gly		0.000	0.022
p.Leu338His	substitution	338	Leu	His		0.000	0.035
p.Leu338Ile	substitution	338	Leu	Ile		0.052	0.101
p.Leu338Lys	substitution	338	Leu	Lys		0.374	0.148
p.Leu338Met	substitution	338	Leu	Met		0.978	0.005
p.Leu338Phe	substitution	338	Leu	Phe		0.000	0.117
p.Leu338Pro	substitution	338	Leu	Pro		0.770	0.159

p.Leu338Ser	substitution	338	Leu	Ser	0.918	0.003
p.Leu338Ter	nonsense	338	Leu	Ter	0.000	0.134
p.Leu338Thr	substitution	338	Leu	Thr	0.726	0.012
p.Leu338Trp	substitution	338	Leu	Trp	0.000	0.084
p.Leu338Tyr	substitution	338	Leu	Tyr	0.000	0.161
p.Leu338Val	substitution	338	Leu	Val	0.000	0.142
p.Leu341=	synonymous	341	Leu	NA	0.797	0.035
p.Leu341Ala	substitution	341	Leu	Ala	0.000	0.266
p.Leu341Arg	substitution	341	Leu	Arg	0.603	0.048
p.Leu341Asp	substitution	341	Leu	Asp	1.203	0.004
p.Leu341Cys	substitution	341	Leu	Cys	0.529	0.003
p.Leu341Glu	substitution	341	Leu	Glu	0.873	0.008
p.Leu341Gly	substitution	341	Leu	Gly	0.674	0.061
p.Leu341His	substitution	341	Leu	His	0.427	0.008
p.Leu341Ile	substitution	341	Leu	Ile	0.000	0.104
p.Leu341Lys	substitution	341	Leu	Lys	0.967	0.003
p.Leu341Met	substitution	341	Leu	Met	0.850	0.008
p.Leu341Phe	substitution	341	Leu	Phe	0.523	0.014
p.Leu341Pro	substitution	341	Leu	Pro	0.135	0.055
p.Leu341Ser	substitution	341	Leu	Ser	0.352	0.129
p.Leu341Ter	nonsense	341	Leu	Ter	0.338	0.020
p.Leu341Thr	substitution	341	Leu	Thr	1.096	0.009
p.Leu341Trp	substitution	341	Leu	Trp	0.652	0.003
p.Leu341Tyr	substitution	341	Leu	Tyr	0.570	0.023
p.Leu341Val	substitution	341	Leu	Val	0.832	0.085
p.Leu342=	synonymous	342	Leu	NA	1.069	0.008
p.Leu342Arg	substitution	342	Leu	Arg	0.000	0.007
p.Leu342Asp	substitution	342	Leu	Asp	0.000	0.007
p.Leu342Cys	substitution	342	Leu	Cys	0.307	0.003
p.Leu342Gln	substitution	342	Leu	Gln	0.641	0.007
p.Leu342Glu	substitution	342	Leu	Glu	0.003	0.007
p.Leu342Gly	substitution	342	Leu	Gly	0.375	0.009
p.Leu342His	substitution	342	Leu	His	1.301	0.005
p.Leu342Ile	substitution	342	Leu	Ile	0.495	0.005
p.Leu342Phe	substitution	342	Leu	Phe	1.334	0.164
p.Leu342Pro	substitution	342	Leu	Pro	0.000	0.020
p.Leu342Ser	substitution	342	Leu	Ser	0.630	0.013
p.Leu342Ter	nonsense	342	Leu	Ter	0.174	0.009
p.Leu342Thr	substitution	342	Leu	Thr	0.597	0.016
p.Leu342Trp	substitution	342	Leu	Trp	0.037	0.005
p.Leu342Tyr	substitution	342	Leu	Tyr	1.014	0.002
p.Leu342Val	substitution	342	Leu	Val	1.446	0.019
p.Leu343=	synonymous	343	Leu	NA	1.065	0.014

p.Leu343Ala	substitution	343	Leu	Ala		0.659	0.098
p.Leu343Arg	substitution	343	Leu	Arg		1.102	0.010
p.Leu343Asn	substitution	343	Leu	Asn		1.219	0.002
p.Leu343Asp	substitution	343	Leu	Asp		1.181	0.004
p.Leu343Cys	substitution	343	Leu	Cys		0.460	0.005
p.Leu343Gln	substitution	343	Leu	Gln		0.520	0.008
p.Leu343Glu	substitution	343	Leu	Glu		0.562	0.007
p.Leu343Gly	substitution	343	Leu	Gly		0.786	0.005
p.Leu343His	substitution	343	Leu	His		1.079	0.004
p.Leu343Ile	substitution	343	Leu	Ile		0.000	0.011
p.Leu343Lys	substitution	343	Leu	Lys		0.303	0.014
p.Leu343Met	substitution	343	Leu	Met		1.218	0.005
p.Leu343Phe	substitution	343	Leu	Phe		0.828	0.003
p.Leu343Pro	substitution	343	Leu	Pro		0.300	0.134
p.Leu343Ser	substitution	343	Leu	Ser		0.449	0.043
p.Leu343Ter	nonsense	343	Leu	Ter		0.000	0.108
p.Leu343Thr	substitution	343	Leu	Thr		0.762	0.246
p.Leu343Trp	substitution	343	Leu	Trp		1.373	0.004
p.Leu343Tyr	substitution	343	Leu	Tyr		0.000	0.030
p.Leu343Val	substitution	343	Leu	Val		1.369	0.011
p.Leu351=	synonymous	351	Leu	NA		0.243	0.065
p.Leu351Ala	substitution	351	Leu	Ala		0.000	0.149
p.Leu351Arg	substitution	351	Leu	Arg		0.513	0.052
p.Leu351Asn	substitution	351	Leu	Asn		0.000	0.252
p.Leu351Asp	substitution	351	Leu	Asp		1.099	0.013
p.Leu351Cys	substitution	351	Leu	Cys		1.418	0.004
p.Leu351Gln	substitution	351	Leu	Gln		1.294	0.000
p.Leu351Glu	substitution	351	Leu	Glu		1.125	0.003
p.Leu351Gly	substitution	351	Leu	Gly		0.000	0.150
p.Leu351His	substitution	351	Leu	His		0.884	0.016
p.Leu351Ile	substitution	351	Leu	Ile		0.671	0.009
p.Leu351Lys	substitution	351	Leu	Lys		0.000	0.051
p.Leu351Met	substitution	351	Leu	Met		1.136	0.004
p.Leu351Pro	substitution	351	Leu	Pro		0.746	0.016
p.Leu351Ser	substitution	351	Leu	Ser		0.519	0.230
p.Leu351Ter	nonsense	351	Leu	Ter		0.063	0.021
p.Leu351Thr	substitution	351	Leu	Thr		1.019	0.140
p.Leu351Trp	substitution	351	Leu	Trp		0.340	0.008
p.Leu351Tyr	substitution	351	Leu	Tyr		0.913	0.269
p.Leu351Val	substitution	351	Leu	Val		0.349	0.042
p.Leu357=	synonymous	357	Leu	NA		1.267	0.297
p.Leu357Arg	substitution	357	Leu	Arg		1.086	0.262
p.Leu357Cys	substitution	357	Leu	Cys		0.000	0.097

p.Leu357His	substitution	357	Leu	His		1.485	0.036
p.Leu357Ile	substitution	357	Leu	Ile		1.112	0.110
p.Leu357Lys	substitution	357	Leu	Lys		0.820	0.016
p.Leu357Phe	substitution	357	Leu	Phe		1.160	0.005
p.Leu357Ser	substitution	357	Leu	Ser		1.376	0.029
p.Leu357Trp	substitution	357	Leu	Trp		0.000	0.216
p.Leu357Tyr	substitution	357	Leu	Tyr		0.865	0.004
p.Leu357Val	substitution	357	Leu	Val		0.796	0.122
p.Leu42=	synonymous	42	Leu	NA		0.902	0.004
p.Leu42Ala	substitution	42	Leu	Ala		0.448	0.007
p.Leu42Arg	substitution	42	Leu	Arg		0.000	0.009
p.Leu42Asn	substitution	42	Leu	Asn		0.000	0.019
p.Leu42Asp	substitution	42	Leu	Asp		0.000	0.009
p.Leu42Cys	substitution	42	Leu	Cys		0.611	0.003
p.Leu42Gln	substitution	42	Leu	Gln		0.000	0.022
p.Leu42Glu	substitution	42	Leu	Glu		0.038	0.012
p.Leu42Gly	substitution	42	Leu	Gly		0.000	0.006
p.Leu42His	substitution	42	Leu	His		0.000	0.015
p.Leu42Ile	substitution	42	Leu	Ile		0.776	0.004
p.Leu42Lys	substitution	42	Leu	Lys		0.000	0.012
p.Leu42Met	substitution	42	Leu	Met		0.677	0.004
p.Leu42Phe	substitution	42	Leu	Phe		0.708	0.003
p.Leu42Pro	substitution	42	Leu	Pro		0.000	0.008
p.Leu42Ser	substitution	42	Leu	Ser		0.000	0.007
p.Leu42Ter	nonsense	42	Leu	Ter		0.109	0.014
p.Leu42Thr	substitution	42	Leu	Thr		0.221	0.008
p.Leu42Tyr	substitution	42	Leu	Tyr		0.182	0.005
p.Leu42Val	substitution	42	Leu	Val		0.523	0.004
p.Leu49=	synonymous	49	Leu	NA		1.065	0.006
p.Leu49Ala	substitution	49	Leu	Ala		1.110	0.023
p.Leu49Arg	substitution	49	Leu	Arg		0.515	0.085
p.Leu49Asn	substitution	49	Leu	Asn		0.060	0.669
p.Leu49Asp	substitution	49	Leu	Asp		0.642	0.137
p.Leu49Cys	substitution	49	Leu	Cys		0.000	0.278
p.Leu49Gln	substitution	49	Leu	Gln		1.019	0.013
p.Leu49Glu	substitution	49	Leu	Glu		0.000	0.789
p.Leu49Gly	substitution	49	Leu	Gly		0.926	0.146
p.Leu49His	substitution	49	Leu	His		0.833	0.012
p.Leu49Ile	substitution	49	Leu	Ile		0.524	0.051
p.Leu49Lys	substitution	49	Leu	Lys		0.581	0.021
p.Leu49Met	substitution	49	Leu	Met		0.858	0.008
p.Leu49Phe	substitution	49	Leu	Phe		0.232	0.119
p.Leu49Pro	substitution	49	Leu	Pro		0.896	0.077

p.Leu49Ser	substitution	49	Leu	Ser		0.906	0.014
p.Leu49Ter	nonsense	49	Leu	Ter		0.210	0.769
p.Leu49Thr	substitution	49	Leu	Thr		1.007	0.034
p.Leu49Trp	substitution	49	Leu	Trp		0.618	0.010
p.Leu49Tyr	substitution	49	Leu	Tyr		1.683	0.033
p.Leu49Val	substitution	49	Leu	Val		1.046	0.033
p.Leu64=	synonymous	64	Leu	NA		0.900	0.099
p.Leu64Ala	substitution	64	Leu	Ala		1.755	0.011
p.Leu64Arg	substitution	64	Leu	Arg		0.730	0.079
p.Leu64Asp	substitution	64	Leu	Asp		0.641	0.016
p.Leu64Cys	substitution	64	Leu	Cys		0.964	0.029
p.Leu64Gln	substitution	64	Leu	Gln		0.656	0.013
p.Leu64Glu	substitution	64	Leu	Glu		0.000	0.160
p.Leu64Gly	substitution	64	Leu	Gly		1.092	0.094
p.Leu64His	substitution	64	Leu	His		1.300	0.012
p.Leu64Ile	substitution	64	Leu	Ile		0.858	0.009
p.Leu64Lys	substitution	64	Leu	Lys		0.996	0.019
p.Leu64Met	substitution	64	Leu	Met		0.687	0.013
p.Leu64Phe	substitution	64	Leu	Phe		0.759	0.006
p.Leu64Pro	substitution	64	Leu	Pro		0.977	0.038
p.Leu64Ser	substitution	64	Leu	Ser		0.479	0.063
p.Leu64Ter	nonsense	64	Leu	Ter		0.000	0.524
p.Leu64Thr	substitution	64	Leu	Thr		0.933	0.218
p.Leu64Trp	substitution	64	Leu	Trp		1.289	0.012
p.Leu64Tyr	substitution	64	Leu	Tyr		1.104	0.009
p.Leu64Val	substitution	64	Leu	Val		0.315	0.099
p.Leu68=	synonymous	68	Leu	NA		0.897	0.006
p.Leu68Ala	substitution	68	Leu	Ala		0.846	0.150
p.Leu68Arg	substitution	68	Leu	Arg		0.926	0.039
p.Leu68Asn	substitution	68	Leu	Asn		0.419	0.012
p.Leu68Asp	substitution	68	Leu	Asp		0.717	0.013
p.Leu68Cys	substitution	68	Leu	Cys		0.762	0.015
p.Leu68Gln	substitution	68	Leu	Gln		0.392	0.016
p.Leu68Glu	substitution	68	Leu	Glu		0.552	0.017
p.Leu68Gly	substitution	68	Leu	Gly		0.487	0.021
p.Leu68His	substitution	68	Leu	His		0.265	0.081
p.Leu68Ile	substitution	68	Leu	Ile		1.013	0.026
p.Leu68Lys	substitution	68	Leu	Lys		1.039	0.007
p.Leu68Met	substitution	68	Leu	Met		1.089	0.010
p.Leu68Phe	substitution	68	Leu	Phe		0.374	0.038
p.Leu68Pro	substitution	68	Leu	Pro		0.667	0.008
p.Leu68Ser	substitution	68	Leu	Ser		0.788	0.013
p.Leu68Ter	nonsense	68	Leu	Ter		0.305	0.018

p.Leu68Thr	substitution	68	Leu	Thr	0.375	0.019
p.Leu68Trp	substitution	68	Leu	Trp	0.588	0.016
p.Leu68Tyr	substitution	68	Leu	Tyr	0.852	0.012
p.Leu68Val	substitution	68	Leu	Val	0.913	0.029
p.Leu76=	synonymous	76	Leu	NA	1.055	0.018
p.Leu76Ala	substitution	76	Leu	Ala	0.426	0.014
p.Leu76Arg	substitution	76	Leu	Arg	0.904	0.007
p.Leu76Asn	substitution	76	Leu	Asn	0.926	0.011
p.Leu76Asp	substitution	76	Leu	Asp	0.916	0.010
p.Leu76Cys	substitution	76	Leu	Cys	1.159	0.005
p.Leu76Gln	substitution	76	Leu	Gln	0.808	0.006
p.Leu76Glu	substitution	76	Leu	Glu	0.351	0.009
p.Leu76Gly	substitution	76	Leu	Gly	0.771	0.069
p.Leu76Ile	substitution	76	Leu	Ile	0.432	0.022
p.Leu76Lys	substitution	76	Leu	Lys	1.112	0.004
p.Leu76Met	substitution	76	Leu	Met	0.984	0.003
p.Leu76Phe	substitution	76	Leu	Phe	0.544	0.007
p.Leu76Pro	substitution	76	Leu	Pro	0.777	0.009
p.Leu76Ser	substitution	76	Leu	Ser	0.850	0.047
p.Leu76Ter	nonsense	76	Leu	Ter	0.000	0.014
p.Leu76Thr	substitution	76	Leu	Thr	0.825	0.018
p.Leu76Trp	substitution	76	Leu	Trp	0.775	0.003
p.Leu76Tyr	substitution	76	Leu	Tyr	0.567	0.015
p.Leu76Val	substitution	76	Leu	Val	1.048	0.007
p.Leu81=	synonymous	81	Leu	NA	0.913	0.092
p.Leu81Ala	substitution	81	Leu	Ala	0.170	0.018
p.Leu81Arg	substitution	81	Leu	Arg	0.000	0.032
p.Leu81Asn	substitution	81	Leu	Asn	0.000	0.032
p.Leu81Asp	substitution	81	Leu	Asp	0.108	0.013
p.Leu81Cys	substitution	81	Leu	Cys	0.665	0.008
p.Leu81Gln	substitution	81	Leu	Gln	0.000	0.018
p.Leu81Glu	substitution	81	Leu	Glu	0.035	0.017
p.Leu81Gly	substitution	81	Leu	Gly	0.592	0.011
p.Leu81His	substitution	81	Leu	His	0.854	0.011
p.Leu81Ile	substitution	81	Leu	Ile	0.949	0.003
p.Leu81Lys	substitution	81	Leu	Lys	0.000	0.012
p.Leu81Met	substitution	81	Leu	Met	0.618	0.004
p.Leu81Phe	substitution	81	Leu	Phe	0.987	0.002
p.Leu81Pro	substitution	81	Leu	Pro	0.519	0.021
p.Leu81Ser	substitution	81	Leu	Ser	0.039	0.013
p.Leu81Ter	nonsense	81	Leu	Ter	0.000	0.012
p.Leu81Thr	substitution	81	Leu	Thr	0.778	0.013
p.Leu81Trp	substitution	81	Leu	Trp	0.724	0.006

p.Leu81Tyr	substitution	81	Leu	Tyr		0.100	0.020
p.Leu81Val	substitution	81	Leu	Val		0.655	0.007
p.Leu85=	synonymous	85	Leu	NA		1.125	0.010
p.Leu85Ala	substitution	85	Leu	Ala		0.486	0.031
p.Leu85Arg	substitution	85	Leu	Arg		0.000	0.015
p.Leu85Asn	substitution	85	Leu	Asn		0.000	0.043
p.Leu85Asp	substitution	85	Leu	Asp		0.000	0.037
p.Leu85Cys	substitution	85	Leu	Cys		0.864	0.004
p.Leu85Gln	substitution	85	Leu	Gln		0.436	0.005
p.Leu85Glu	substitution	85	Leu	Glu		0.021	0.007
p.Leu85Gly	substitution	85	Leu	Gly		0.000	0.019
p.Leu85His	substitution	85	Leu	His		0.000	0.011
p.Leu85Ile	substitution	85	Leu	Ile		0.556	0.011
p.Leu85Lys	substitution	85	Leu	Lys		0.066	0.024
p.Leu85Met	substitution	85	Leu	Met		0.850	0.003
p.Leu85Phe	substitution	85	Leu	Phe		0.182	0.006
p.Leu85Pro	substitution	85	Leu	Pro		0.000	0.062
p.Leu85Ser	substitution	85	Leu	Ser		0.000	0.006
p.Leu85Ter	nonsense	85	Leu	Ter		0.279	0.016
p.Leu85Thr	substitution	85	Leu	Thr		0.395	0.014
p.Leu85Trp	substitution	85	Leu	Trp		0.000	0.011
p.Leu85Tyr	substitution	85	Leu	Tyr		0.000	0.023
p.Leu85Val	substitution	85	Leu	Val		0.780	0.003
p.Leu92=	synonymous	92	Leu	NA		1.039	0.008
p.Leu92Ala	substitution	92	Leu	Ala		0.791	0.009
p.Leu92Arg	substitution	92	Leu	Arg		0.000	0.015
p.Leu92Asn	substitution	92	Leu	Asn		0.983	0.060
p.Leu92Asp	substitution	92	Leu	Asp		0.000	0.067
p.Leu92Cys	substitution	92	Leu	Cys		0.988	0.012
p.Leu92Gln	substitution	92	Leu	Gln		0.133	0.062
p.Leu92Glu	substitution	92	Leu	Glu		0.000	0.064
p.Leu92Gly	substitution	92	Leu	Gly		0.430	0.015
p.Leu92His	substitution	92	Leu	His		0.272	0.008
p.Leu92Ile	substitution	92	Leu	Ile		0.932	0.007
p.Leu92Lys	substitution	92	Leu	Lys		0.025	0.015
p.Leu92Met	substitution	92	Leu	Met		0.948	0.004
p.Leu92Phe	substitution	92	Leu	Phe		0.794	0.008
p.Leu92Pro	substitution	92	Leu	Pro		0.000	0.144
p.Leu92Ser	substitution	92	Leu	Ser		0.215	0.006
p.Leu92Ter	nonsense	92	Leu	Ter		0.330	0.016
p.Leu92Thr	substitution	92	Leu	Thr		0.600	0.108
p.Leu92Trp	substitution	92	Leu	Trp		0.000	0.039
p.Leu92Tyr	substitution	92	Leu	Tyr		0.000	0.015

p.Leu92Val	substitution	92	Leu	Val	0.516	0.155
p.Leu97=	synonymous	97	Leu	NA	1.077	0.009
p.Leu97Ala	substitution	97	Leu	Ala	0.834	0.005
p.Leu97Arg	substitution	97	Leu	Arg	0.000	0.024
p.Leu97Asn	substitution	97	Leu	Asn	0.190	0.008
p.Leu97Asp	substitution	97	Leu	Asp	0.000	0.012
p.Leu97Cys	substitution	97	Leu	Cys	0.527	0.015
p.Leu97Gln	substitution	97	Leu	Gln	0.000	0.014
p.Leu97Glu	substitution	97	Leu	Glu	0.133	0.010
p.Leu97Gly	substitution	97	Leu	Gly	0.000	0.009
p.Leu97His	substitution	97	Leu	His	0.000	0.015
p.Leu97Ile	substitution	97	Leu	Ile	0.635	0.005
p.Leu97Lys	substitution	97	Leu	Lys	0.183	0.009
p.Leu97Met	substitution	97	Leu	Met	0.647	0.012
p.Leu97Phe	substitution	97	Leu	Phe	0.796	0.006
p.Leu97Pro	substitution	97	Leu	Pro	0.000	0.028
p.Leu97Ser	substitution	97	Leu	Ser	0.353	0.048
p.Leu97Ter	nonsense	97	Leu	Ter	0.383	0.043
p.Leu97Thr	substitution	97	Leu	Thr	1.290	0.007
p.Leu97Trp	substitution	97	Leu	Trp	0.000	0.025
p.Leu97Tyr	substitution	97	Leu	Tyr	0.000	0.017
p.Leu97Val	substitution	97	Leu	Val	0.972	0.014
p.Lys115=	synonymous	115	Lys	NA	1.084	0.023
p.Lys115Ala	substitution	115	Lys	Ala	0.523	0.021
p.Lys115Arg	substitution	115	Lys	Arg	0.851	0.013
p.Lys115Asp	substitution	115	Lys	Asp	1.068	0.008
p.Lys115Cys	substitution	115	Lys	Cys	0.631	0.012
p.Lys115Gln	substitution	115	Lys	Gln	0.882	0.004
p.Lys115Glu	substitution	115	Lys	Glu	0.836	0.022
p.Lys115Gly	substitution	115	Lys	Gly	1.030	0.021
p.Lys115His	substitution	115	Lys	His	0.654	0.005
p.Lys115Ile	substitution	115	Lys	Ile	0.673	0.008
p.Lys115Leu	substitution	115	Lys	Leu	0.931	0.024
p.Lys115Phe	substitution	115	Lys	Phe	0.850	0.005
p.Lys115Pro	substitution	115	Lys	Pro	1.019	0.010
p.Lys115Ser	substitution	115	Lys	Ser	1.101	0.032
p.Lys115Ter	nonsense	115	Lys	Ter	0.069	0.012
p.Lys115Thr	substitution	115	Lys	Thr	0.957	0.003
p.Lys115Trp	substitution	115	Lys	Trp	0.368	0.007
p.Lys115Tyr	substitution	115	Lys	Tyr	0.446	0.006
p.Lys115Val	substitution	115	Lys	Val	0.748	0.006
p.Lys128=	synonymous	128	Lys	NA	1.189	0.017
p.Lys128Ala	substitution	128	Lys	Ala	0.901	0.091

p.Lys128Arg	substitution	128	Lys	Arg	0.642	0.027
p.Lys128Asn	substitution	128	Lys	Asn	1.104	0.049
p.Lys128Asp	substitution	128	Lys	Asp	0.657	0.010
p.Lys128Cys	substitution	128	Lys	Cys	0.701	0.009
p.Lys128Gln	substitution	128	Lys	Gln	0.377	0.030
p.Lys128Glu	substitution	128	Lys	Glu	1.412	0.013
p.Lys128Gly	substitution	128	Lys	Gly	0.383	0.093
p.Lys128His	substitution	128	Lys	His	0.903	0.033
p.Lys128Ile	substitution	128	Lys	Ile	0.939	0.014
p.Lys128Leu	substitution	128	Lys	Leu	0.902	0.041
p.Lys128Met	substitution	128	Lys	Met	1.244	0.006
p.Lys128Phe	substitution	128	Lys	Phe	0.513	0.030
p.Lys128Pro	substitution	128	Lys	Pro	0.000	0.102
p.Lys128Ser	substitution	128	Lys	Ser	0.757	0.025
p.Lys128Ter	nonsense	128	Lys	Ter	0.467	0.152
p.Lys128Thr	substitution	128	Lys	Thr	1.218	0.200
p.Lys128Trp	substitution	128	Lys	Trp	0.858	0.012
p.Lys128Tyr	substitution	128	Lys	Tyr	0.752	0.010
p.Lys128Val	substitution	128	Lys	Val	0.515	0.019
p.Lys132Ala	substitution	132	Lys	Ala	0.811	0.004
p.Lys132Arg	substitution	132	Lys	Arg	0.899	0.002
p.Lys132Asn	substitution	132	Lys	Asn	1.202	0.029
p.Lys132Cys	substitution	132	Lys	Cys	0.879	0.004
p.Lys132Gln	substitution	132	Lys	Gln	1.139	0.006
p.Lys132Glu	substitution	132	Lys	Glu	0.924	0.004
p.Lys132Gly	substitution	132	Lys	Gly	1.042	0.004
p.Lys132His	substitution	132	Lys	His	0.811	0.005
p.Lys132Ile	substitution	132	Lys	Ile	0.858	0.003
p.Lys132Leu	substitution	132	Lys	Leu	0.812	0.006
p.Lys132Met	substitution	132	Lys	Met	1.090	0.003
p.Lys132Phe	substitution	132	Lys	Phe	1.118	0.005
p.Lys132Pro	substitution	132	Lys	Pro	0.917	0.007
p.Lys132Ser	substitution	132	Lys	Ser	0.966	0.002
p.Lys132Ter	nonsense	132	Lys	Ter	0.375	0.007
p.Lys132Thr	substitution	132	Lys	Thr	0.796	0.003
p.Lys132Trp	substitution	132	Lys	Trp	0.990	0.003
p.Lys132Tyr	substitution	132	Lys	Tyr	0.912	0.003
p.Lys132Val	substitution	132	Lys	Val	0.739	0.007
p.Lys140=	synonymous	140	Lys	NA	1.218	0.040
p.Lys140Ala	substitution	140	Lys	Ala	0.909	0.008
p.Lys140Arg	substitution	140	Lys	Arg	0.856	0.042
p.Lys140Asn	substitution	140	Lys	Asn	0.900	0.037
p.Lys140Asp	substitution	140	Lys	Asp	1.054	0.004

p.Lys140Cys	substitution	140	Lys	Cys	0.814	0.011
p.Lys140Glu	substitution	140	Lys	Glu	1.083	0.003
p.Lys140Gly	substitution	140	Lys	Gly	0.903	0.017
p.Lys140His	substitution	140	Lys	His	0.606	0.006
p.Lys140Ile	substitution	140	Lys	Ile	0.592	0.004
p.Lys140Leu	substitution	140	Lys	Leu	0.871	0.005
p.Lys140Met	substitution	140	Lys	Met	0.816	0.003
p.Lys140Phe	substitution	140	Lys	Phe	0.787	0.006
p.Lys140Pro	substitution	140	Lys	Pro	0.930	0.045
p.Lys140Ser	substitution	140	Lys	Ser	0.996	0.002
p.Lys140Ter	nonsense	140	Lys	Ter	0.289	0.007
p.Lys140Thr	substitution	140	Lys	Thr	0.801	0.006
p.Lys140Trp	substitution	140	Lys	Trp	1.165	0.003
p.Lys140Tyr	substitution	140	Lys	Tyr	0.704	0.004
p.Lys140Val	substitution	140	Lys	Val	0.780	0.063
p.Lys157=	synonymous	157	Lys	NA	1.359	0.015
p.Lys157Ala	substitution	157	Lys	Ala	1.031	0.004
p.Lys157Arg	substitution	157	Lys	Arg	0.848	0.023
p.Lys157Asn	substitution	157	Lys	Asn	1.031	0.035
p.Lys157Asp	substitution	157	Lys	Asp	0.653	0.008
p.Lys157Cys	substitution	157	Lys	Cys	0.876	0.005
p.Lys157Gln	substitution	157	Lys	Gln	0.958	0.007
p.Lys157Glu	substitution	157	Lys	Glu	0.486	0.017
p.Lys157Gly	substitution	157	Lys	Gly	0.924	0.012
p.Lys157His	substitution	157	Lys	His	1.034	0.007
p.Lys157Ile	substitution	157	Lys	Ile	0.800	0.008
p.Lys157Leu	substitution	157	Lys	Leu	0.948	0.003
p.Lys157Met	substitution	157	Lys	Met	0.954	0.006
p.Lys157Phe	substitution	157	Lys	Phe	0.816	0.006
p.Lys157Pro	substitution	157	Lys	Pro	0.637	0.040
p.Lys157Ser	substitution	157	Lys	Ser	0.866	0.008
p.Lys157Ter	nonsense	157	Lys	Ter	0.291	0.011
p.Lys157Thr	substitution	157	Lys	Thr	0.542	0.059
p.Lys157Trp	substitution	157	Lys	Trp	0.303	0.007
p.Lys157Tyr	substitution	157	Lys	Tyr	0.943	0.005
p.Lys157Val	substitution	157	Lys	Val	0.827	0.005
p.Lys176=	synonymous	176	Lys	NA	1.446	0.027
p.Lys176Ala	substitution	176	Lys	Ala	0.681	0.073
p.Lys176Arg	substitution	176	Lys	Arg	0.892	0.008
p.Lys176Asn	substitution	176	Lys	Asn	0.604	0.010
p.Lys176Asp	substitution	176	Lys	Asp	0.717	0.005
p.Lys176Gln	substitution	176	Lys	Gln	1.057	0.008
p.Lys176Glu	substitution	176	Lys	Glu	0.693	0.019

p.Lys176Gly	substitution	176	Lys	Gly	0.592	0.139
p.Lys176His	substitution	176	Lys	His	0.697	0.005
p.Lys176Ile	substitution	176	Lys	Ile	0.903	0.004
p.Lys176Leu	substitution	176	Lys	Leu	0.950	0.006
p.Lys176Met	substitution	176	Lys	Met	0.955	0.004
p.Lys176Phe	substitution	176	Lys	Phe	1.004	0.005
p.Lys176Pro	substitution	176	Lys	Pro	0.000	0.054
p.Lys176Ser	substitution	176	Lys	Ser	0.815	0.009
p.Lys176Ter	nonsense	176	Lys	Ter	0.404	0.013
p.Lys176Thr	substitution	176	Lys	Thr	0.836	0.004
p.Lys176Trp	substitution	176	Lys	Trp	0.800	0.007
p.Lys176Tyr	substitution	176	Lys	Tyr	0.794	0.003
p.Lys176Val	substitution	176	Lys	Val	0.882	0.004
p.Lys227Ala	substitution	227	Lys	Ala	1.364	0.004
p.Lys227Arg	substitution	227	Lys	Arg	0.611	0.024
p.Lys227Asn	substitution	227	Lys	Asn	0.489	0.006
p.Lys227Asp	substitution	227	Lys	Asp	1.214	0.003
p.Lys227Cys	substitution	227	Lys	Cys	0.000	0.160
p.Lys227Gln	substitution	227	Lys	Gln	0.721	0.003
p.Lys227Glu	substitution	227	Lys	Glu	1.736	0.001
p.Lys227Gly	substitution	227	Lys	Gly	0.147	0.008
p.Lys227His	substitution	227	Lys	His	0.264	0.010
p.Lys227Ile	substitution	227	Lys	Ile	0.328	0.022
p.Lys227Leu	substitution	227	Lys	Leu	1.193	0.007
p.Lys227Met	substitution	227	Lys	Met	0.424	0.004
p.Lys227Phe	substitution	227	Lys	Phe	0.025	0.011
p.Lys227Pro	substitution	227	Lys	Pro	0.000	0.214
p.Lys227Ser	substitution	227	Lys	Ser	0.743	0.015
p.Lys227Ter	nonsense	227	Lys	Ter	0.427	0.135
p.Lys227Thr	substitution	227	Lys	Thr	0.539	0.074
p.Lys227Trp	substitution	227	Lys	Trp	0.479	0.004
p.Lys227Tyr	substitution	227	Lys	Tyr	1.299	0.003
p.Lys227Val	substitution	227	Lys	Val	0.000	0.015
p.Lys272=	synonymous	272	Lys	NA	1.425	0.014
p.Lys272Arg	substitution	272	Lys	Arg	0.784	0.032
p.Lys272Asn	substitution	272	Lys	Asn	1.175	0.021
p.Lys272Cys	substitution	272	Lys	Cys	0.413	0.005
p.Lys272Gln	substitution	272	Lys	Gln	0.726	0.160
p.Lys272Glu	substitution	272	Lys	Glu	0.599	0.606
p.Lys272Gly	substitution	272	Lys	Gly	0.000	0.101
p.Lys272Leu	substitution	272	Lys	Leu	0.627	0.095
p.Lys272Phe	substitution	272	Lys	Phe	0.985	0.008
p.Lys272Ser	substitution	272	Lys	Ser	1.115	0.036

p.Lys272Ter	nonsense	272	Lys	Ter	0.544	0.880
p.Lys272Thr	substitution	272	Lys	Thr	0.853	0.161
p.Lys272Trp	substitution	272	Lys	Trp	1.522	0.004
p.Lys272Tyr	substitution	272	Lys	Tyr	0.000	0.009
p.Lys272Val	substitution	272	Lys	Val	1.404	0.005
p.Lys27=	synonymous	27	Lys	NA	1.033	0.006
p.Lys27Ala	substitution	27	Lys	Ala	1.023	0.028
p.Lys27Arg	substitution	27	Lys	Arg	1.226	0.007
p.Lys27Asn	substitution	27	Lys	Asn	1.139	0.001
p.Lys27Asp	substitution	27	Lys	Asp	1.143	0.001
p.Lys27Cys	substitution	27	Lys	Cys	0.988	0.001
p.Lys27Gln	substitution	27	Lys	Gln	0.649	0.001
p.Lys27Glu	substitution	27	Lys	Glu	0.805	0.002
p.Lys27Gly	substitution	27	Lys	Gly	0.745	0.002
p.Lys27His	substitution	27	Lys	His	0.779	0.001
p.Lys27Ile	substitution	27	Lys	Ile	0.778	0.000
p.Lys27Leu	substitution	27	Lys	Leu	0.838	0.001
p.Lys27Met	substitution	27	Lys	Met	0.883	0.000
p.Lys27Phe	substitution	27	Lys	Phe	0.980	0.000
p.Lys27Pro	substitution	27	Lys	Pro	0.863	0.002
p.Lys27Ser	substitution	27	Lys	Ser	0.992	0.002
p.Lys27Ter	nonsense	27	Lys	Ter	0.000	0.003
p.Lys27Thr	substitution	27	Lys	Thr	0.727	0.046
p.Lys27Trp	substitution	27	Lys	Trp	0.993	0.001
p.Lys27Tyr	substitution	27	Lys	Tyr	0.667	0.001
p.Lys27Val	substitution	27	Lys	Val	1.126	0.013
p.Lys345=	synonymous	345	Lys	NA	1.305	0.003
p.Lys345Ala	substitution	345	Lys	Ala	1.071	0.158
p.Lys345Arg	substitution	345	Lys	Arg	0.104	0.149
p.Lys345Asn	substitution	345	Lys	Asn	0.899	0.028
p.Lys345Asp	substitution	345	Lys	Asp	0.281	0.015
p.Lys345Cys	substitution	345	Lys	Cys	0.294	0.108
p.Lys345Gln	substitution	345	Lys	Gln	0.256	0.312
p.Lys345Glu	substitution	345	Lys	Glu	0.000	0.250
p.Lys345Gly	substitution	345	Lys	Gly	0.311	0.071
p.Lys345Ile	substitution	345	Lys	Ile	1.046	0.020
p.Lys345Leu	substitution	345	Lys	Leu	0.878	0.124
p.Lys345Met	substitution	345	Lys	Met	1.110	0.002
p.Lys345Phe	substitution	345	Lys	Phe	1.437	0.003
p.Lys345Pro	substitution	345	Lys	Pro	0.000	0.130
p.Lys345Ter	nonsense	345	Lys	Ter	0.229	0.051
p.Lys345Thr	substitution	345	Lys	Thr	1.363	0.171
p.Lys345Tyr	substitution	345	Lys	Tyr	1.159	0.003

p.Lys345Val	substitution	345	Lys	Val	0.339	0.033
p.Lys348=	synonymous	348	Lys	NA	0.625	0.114
p.Lys348Arg	substitution	348	Lys	Arg	1.317	0.021
p.Lys348Asn	substitution	348	Lys	Asn	1.329	0.020
p.Lys348Asp	substitution	348	Lys	Asp	0.947	0.119
p.Lys348Cys	substitution	348	Lys	Cys	0.000	0.055
p.Lys348Gln	substitution	348	Lys	Gln	0.732	0.077
p.Lys348Glu	substitution	348	Lys	Glu	1.025	0.073
p.Lys348Gly	substitution	348	Lys	Gly	1.065	0.004
p.Lys348His	substitution	348	Lys	His	1.217	0.010
p.Lys348Ile	substitution	348	Lys	Ile	1.292	0.013
p.Lys348Leu	substitution	348	Lys	Leu	1.166	0.125
p.Lys348Met	substitution	348	Lys	Met	0.356	0.006
p.Lys348Phe	substitution	348	Lys	Phe	0.878	0.003
p.Lys348Pro	substitution	348	Lys	Pro	0.032	0.035
p.Lys348Ser	substitution	348	Lys	Ser	1.127	0.016
p.Lys348Ter	nonsense	348	Lys	Ter	0.184	0.089
p.Lys348Thr	substitution	348	Lys	Thr	0.287	0.081
p.Lys348Trp	substitution	348	Lys	Trp	1.061	0.004
p.Lys348Tyr	substitution	348	Lys	Tyr	0.000	0.035
p.Lys348Val	substitution	348	Lys	Val	0.000	0.093
p.Lys43=	synonymous	43	Lys	NA	0.750	0.005
p.Lys43Ala	substitution	43	Lys	Ala	0.755	0.009
p.Lys43Arg	substitution	43	Lys	Arg	0.797	0.010
p.Lys43Asn	substitution	43	Lys	Asn	0.859	0.032
p.Lys43Cys	substitution	43	Lys	Cys	0.673	0.006
p.Lys43Gln	substitution	43	Lys	Gln	0.855	0.047
p.Lys43Glu	substitution	43	Lys	Glu	0.720	0.026
p.Lys43Gly	substitution	43	Lys	Gly	0.749	0.013
p.Lys43His	substitution	43	Lys	His	0.754	0.004
p.Lys43Ile	substitution	43	Lys	Ile	0.987	0.005
p.Lys43Leu	substitution	43	Lys	Leu	0.669	0.016
p.Lys43Met	substitution	43	Lys	Met	0.691	0.002
p.Lys43Phe	substitution	43	Lys	Phe	0.383	0.011
p.Lys43Pro	substitution	43	Lys	Pro	0.038	0.020
p.Lys43Ser	substitution	43	Lys	Ser	0.817	0.008
p.Lys43Ter	nonsense	43	Lys	Ter	0.132	0.152
p.Lys43Thr	substitution	43	Lys	Thr	0.985	0.008
p.Lys43Trp	substitution	43	Lys	Trp	0.697	0.003
p.Lys43Tyr	substitution	43	Lys	Tyr	0.624	0.005
p.Lys43Val	substitution	43	Lys	Val	1.303	0.009
p.Lys62=	synonymous	62	Lys	NA	0.694	0.052
p.Lys62Ala	substitution	62	Lys	Ala	0.898	0.238

p.Lys62Arg	substitution	62	Lys	Arg		0.638	0.090
p.Lys62Asn	substitution	62	Lys	Asn		1.263	0.007
p.Lys62Asp	substitution	62	Lys	Asp		1.729	0.018
p.Lys62Cys	substitution	62	Lys	Cys		0.460	0.024
p.Lys62Gln	substitution	62	Lys	Gln		1.364	0.040
p.Lys62Glu	substitution	62	Lys	Glu		0.659	0.428
p.Lys62Gly	substitution	62	Lys	Gly		2.421	0.533
p.Lys62His	substitution	62	Lys	His		0.000	0.083
p.Lys62Ile	substitution	62	Lys	Ile		1.248	0.033
p.Lys62Leu	substitution	62	Lys	Leu		0.275	0.056
p.Lys62Met	substitution	62	Lys	Met		0.818	0.006
p.Lys62Phe	substitution	62	Lys	Phe		0.562	0.022
p.Lys62Pro	substitution	62	Lys	Pro		0.000	0.054
p.Lys62Ser	substitution	62	Lys	Ser		0.846	0.062
p.Lys62Ter	nonsense	62	Lys	Ter		0.784	0.006
p.Lys62Thr	substitution	62	Lys	Thr		0.000	0.225
p.Lys62Trp	substitution	62	Lys	Trp		0.561	0.026
p.Lys62Tyr	substitution	62	Lys	Tyr		0.951	0.009
p.Lys62Val	substitution	62	Lys	Val		1.025	0.163
p.Lys70=	synonymous	70	Lys	NA		1.209	0.014
p.Lys70Ala	substitution	70	Lys	Ala		0.754	0.019
p.Lys70Arg	substitution	70	Lys	Arg		0.631	0.046
p.Lys70Asn	substitution	70	Lys	Asn		1.204	0.006
p.Lys70Asp	substitution	70	Lys	Asp		0.771	0.012
p.Lys70Cys	substitution	70	Lys	Cys		0.657	0.007
p.Lys70Gln	substitution	70	Lys	Gln		0.581	0.015
p.Lys70Glu	substitution	70	Lys	Glu		0.724	0.103
p.Lys70Gly	substitution	70	Lys	Gly		0.872	0.005
p.Lys70His	substitution	70	Lys	His		0.747	0.016
p.Lys70Ile	substitution	70	Lys	Ile		1.039	0.006
p.Lys70Leu	substitution	70	Lys	Leu		0.508	0.013
p.Lys70Met	substitution	70	Lys	Met		1.080	0.007
p.Lys70Phe	substitution	70	Lys	Phe		0.794	0.009
p.Lys70Pro	substitution	70	Lys	Pro		0.686	0.023
p.Lys70Ser	substitution	70	Lys	Ser		0.578	0.120
p.Lys70Ter	nonsense	70	Lys	Ter		0.000	0.654
p.Lys70Thr	substitution	70	Lys	Thr		0.673	0.014
p.Lys70Trp	substitution	70	Lys	Trp		0.724	0.005
p.Lys70Tyr	substitution	70	Lys	Tyr		0.803	0.006
p.Lys70Val	substitution	70	Lys	Val		1.063	0.067
p.Lys74=	synonymous	74	Lys	NA		0.989	0.013
p.Lys74Ala	substitution	74	Lys	Ala		0.780	0.005
p.Lys74Arg	substitution	74	Lys	Arg		0.290	0.047

p.Lys74Asn	substitution	74	Lys	Asn	1.033	0.025
p.Lys74Asp	substitution	74	Lys	Asp	0.314	0.006
p.Lys74Cys	substitution	74	Lys	Cys	0.309	0.009
p.Lys74Gln	substitution	74	Lys	Gln	1.366	0.007
p.Lys74Glu	substitution	74	Lys	Glu	0.000	0.129
p.Lys74Gly	substitution	74	Lys	Gly	0.724	0.062
p.Lys74His	substitution	74	Lys	His	0.839	0.007
p.Lys74Ile	substitution	74	Lys	Ile	0.543	0.049
p.Lys74Leu	substitution	74	Lys	Leu	0.483	0.032
p.Lys74Met	substitution	74	Lys	Met	0.016	0.012
p.Lys74Phe	substitution	74	Lys	Phe	0.635	0.008
p.Lys74Pro	substitution	74	Lys	Pro	0.476	0.010
p.Lys74Ser	substitution	74	Lys	Ser	0.385	0.089
p.Lys74Ter	nonsense	74	Lys	Ter	0.000	0.060
p.Lys74Thr	substitution	74	Lys	Thr	0.592	0.037
p.Lys74Trp	substitution	74	Lys	Trp	0.000	0.069
p.Lys74Tyr	substitution	74	Lys	Tyr	0.543	0.010
p.Lys74Val	substitution	74	Lys	Val	0.155	0.009
p.Lys79=	synonymous	79	Lys	NA	1.339	0.012
p.Lys79Ala	substitution	79	Lys	Ala	0.796	0.018
p.Lys79Arg	substitution	79	Lys	Arg	0.864	0.027
p.Lys79Asn	substitution	79	Lys	Asn	1.010	0.105
p.Lys79Asp	substitution	79	Lys	Asp	0.841	0.004
p.Lys79Cys	substitution	79	Lys	Cys	0.831	0.005
p.Lys79Gln	substitution	79	Lys	Gln	0.834	0.005
p.Lys79Glu	substitution	79	Lys	Glu	0.933	0.010
p.Lys79Gly	substitution	79	Lys	Gly	0.919	0.010
p.Lys79His	substitution	79	Lys	His	0.958	0.009
p.Lys79Ile	substitution	79	Lys	Ile	0.710	0.007
p.Lys79Leu	substitution	79	Lys	Leu	0.667	0.006
p.Lys79Met	substitution	79	Lys	Met	0.735	0.010
p.Lys79Phe	substitution	79	Lys	Phe	0.354	0.008
p.Lys79Pro	substitution	79	Lys	Pro	0.663	0.006
p.Lys79Ser	substitution	79	Lys	Ser	1.101	0.002
p.Lys79Ter	nonsense	79	Lys	Ter	0.474	0.017
p.Lys79Thr	substitution	79	Lys	Thr	0.773	0.007
p.Lys79Trp	substitution	79	Lys	Trp	0.910	0.004
p.Lys79Tyr	substitution	79	Lys	Tyr	0.976	0.004
p.Lys79Val	substitution	79	Lys	Val	0.456	0.039
p.Lys87=	synonymous	87	Lys	NA	1.035	0.017
p.Lys87Ala	substitution	87	Lys	Ala	0.934	0.038
p.Lys87Arg	substitution	87	Lys	Arg	0.984	0.007
p.Lys87Asn	substitution	87	Lys	Asn	0.944	0.904

p.Lys87Asp	substitution	87	Lys	Asp	1.042	0.004
p.Lys87Cys	substitution	87	Lys	Cys	0.916	0.005
p.Lys87Gln	substitution	87	Lys	Gln	1.175	0.009
p.Lys87Glu	substitution	87	Lys	Glu	0.784	0.006
p.Lys87Gly	substitution	87	Lys	Gly	0.571	0.025
p.Lys87His	substitution	87	Lys	His	0.607	0.021
p.Lys87Ile	substitution	87	Lys	Ile	1.011	0.003
p.Lys87Leu	substitution	87	Lys	Leu	0.892	0.012
p.Lys87Met	substitution	87	Lys	Met	0.959	0.006
p.Lys87Phe	substitution	87	Lys	Phe	0.692	0.004
p.Lys87Pro	substitution	87	Lys	Pro	0.000	0.017
p.Lys87Ser	substitution	87	Lys	Ser	1.056	0.004
p.Lys87Ter	nonsense	87	Lys	Ter	0.285	0.030
p.Lys87Thr	substitution	87	Lys	Thr	0.962	0.006
p.Lys87Trp	substitution	87	Lys	Trp	0.971	0.005
p.Lys87Tyr	substitution	87	Lys	Tyr	1.266	0.003
p.Lys87Val	substitution	87	Lys	Val	0.504	0.011
p.Lys98=	synonymous	98	Lys	NA	0.842	0.028
p.Lys98Ala	substitution	98	Lys	Ala	0.105	0.033
p.Lys98Arg	substitution	98	Lys	Arg	0.000	0.015
p.Lys98Asn	substitution	98	Lys	Asn	0.466	0.011
p.Lys98Asp	substitution	98	Lys	Asp	0.000	0.021
p.Lys98Cys	substitution	98	Lys	Cys	0.325	0.008
p.Lys98Gln	substitution	98	Lys	Gln	0.000	0.012
p.Lys98Glu	substitution	98	Lys	Glu	0.153	0.224
p.Lys98Gly	substitution	98	Lys	Gly	0.000	0.018
p.Lys98His	substitution	98	Lys	His	0.000	0.028
p.Lys98Ile	substitution	98	Lys	Ile	0.218	0.014
p.Lys98Leu	substitution	98	Lys	Leu	0.000	0.007
p.Lys98Met	substitution	98	Lys	Met	0.000	0.017
p.Lys98Phe	substitution	98	Lys	Phe	0.000	0.024
p.Lys98Pro	substitution	98	Lys	Pro	0.024	0.032
p.Lys98Ser	substitution	98	Lys	Ser	0.000	0.015
p.Lys98Ter	nonsense	98	Lys	Ter	0.000	0.046
p.Lys98Thr	substitution	98	Lys	Thr	0.000	0.029
p.Lys98Trp	substitution	98	Lys	Trp	0.000	0.020
p.Lys98Tyr	substitution	98	Lys	Tyr	0.000	0.022
p.Lys98Val	substitution	98	Lys	Val	0.000	0.007
p.Met196Ala	substitution	196	Met	Ala	0.965	0.102
p.Met196Arg	substitution	196	Met	Arg	0.371	0.043
p.Met196Asr	substitution	196	Met	Asn	0.751	0.004
p.Met196Cys	substitution	196	Met	Cys	1.200	0.002
p.Met196Glr	substitution	196	Met	Gln	1.232	0.003

p.Met196Glu	substitution	196	Met	Glu	0.233	0.006
p.Met196Gly	substitution	196	Met	Gly	0.730	0.031
p.Met196His	substitution	196	Met	His	0.975	0.002
p.Met196Ile	substitution	196	Met	Ile	1.071	0.015
p.Met196Leu	substitution	196	Met	Leu	0.951	0.029
p.Met196Lys	substitution	196	Met	Lys	0.276	0.007
p.Met196Phe	substitution	196	Met	Phe	1.154	0.002
p.Met196Pro	substitution	196	Met	Pro	0.000	0.005
p.Met196Ser	substitution	196	Met	Ser	0.322	0.031
p.Met196Ter	nonsense	196	Met	Ter	0.000	0.017
p.Met196Thr	substitution	196	Met	Thr	0.409	0.007
p.Met196Trp	substitution	196	Met	Trp	0.268	0.002
p.Met196Tyr	substitution	196	Met	Tyr	0.952	0.003
p.Met196Val	substitution	196	Met	Val	0.864	0.007
p.Met212Ala	substitution	212	Met	Ala	0.000	0.082
p.Met212Arg	substitution	212	Met	Arg	0.000	0.026
p.Met212Asn	substitution	212	Met	Asn	0.000	0.031
p.Met212Asp	substitution	212	Met	Asp	0.000	0.063
p.Met212Cys	substitution	212	Met	Cys	0.000	0.171
p.Met212Gln	substitution	212	Met	Gln	0.019	0.029
p.Met212Glu	substitution	212	Met	Glu	0.296	0.140
p.Met212Gly	substitution	212	Met	Gly	1.195	0.022
p.Met212His	substitution	212	Met	His	1.105	0.003
p.Met212Ile	substitution	212	Met	Ile	1.209	0.025
p.Met212Leu	substitution	212	Met	Leu	1.120	0.014
p.Met212Lys	substitution	212	Met	Lys	0.000	0.609
p.Met212Phe	substitution	212	Met	Phe	2.044	0.001
p.Met212Pro	substitution	212	Met	Pro	0.291	0.131
p.Met212Ser	substitution	212	Met	Ser	0.744	0.004
p.Met212Ter	nonsense	212	Met	Ter	0.000	0.013
p.Met212Thr	substitution	212	Met	Thr	0.847	0.030
p.Met212Trp	substitution	212	Met	Trp	0.000	0.013
p.Met212Tyr	substitution	212	Met	Tyr	0.000	0.045
p.Met212Val	substitution	212	Met	Val	0.165	0.252
p.Met271Ala	substitution	271	Met	Ala	0.076	0.013
p.Met271Arg	substitution	271	Met	Arg	0.567	0.124
p.Met271Asn	substitution	271	Met	Asn	0.601	0.007
p.Met271Asp	substitution	271	Met	Asp	1.269	0.008
p.Met271Cys	substitution	271	Met	Cys	0.454	0.004
p.Met271Gln	substitution	271	Met	Gln	0.706	0.010
p.Met271Glu	substitution	271	Met	Glu	1.447	0.004
p.Met271Gly	substitution	271	Met	Gly	1.017	0.007
p.Met271His	substitution	271	Met	His	1.264	0.005

p.Met271Ile	substitution	271	Met	Ile	1.282	0.013
p.Met271Leu	substitution	271	Met	Leu	0.979	0.067
p.Met271Lys	substitution	271	Met	Lys	0.823	0.344
p.Met271Phe	substitution	271	Met	Phe	1.056	0.004
p.Met271Pro	substitution	271	Met	Pro	0.000	0.060
p.Met271Ser	substitution	271	Met	Ser	0.961	0.023
p.Met271Ter	nonsense	271	Met	Ter	0.000	0.008
p.Met271Thr	substitution	271	Met	Thr	0.446	0.035
p.Met271Trp	substitution	271	Met	Trp	0.398	0.029
p.Met271Tyr	substitution	271	Met	Tyr	1.225	0.005
p.Met271Val	substitution	271	Met	Val	0.785	0.229
p.Met295Ala	substitution	295	Met	Ala	0.971	0.012
p.Met295Arg	substitution	295	Met	Arg	1.126	0.008
p.Met295Asn	substitution	295	Met	Asn	0.000	0.008
p.Met295Asp	substitution	295	Met	Asp	0.687	0.003
p.Met295Cys	substitution	295	Met	Cys	0.103	0.005
p.Met295Gln	substitution	295	Met	Gln	0.292	0.008
p.Met295Glu	substitution	295	Met	Glu	0.515	0.003
p.Met295Gly	substitution	295	Met	Gly	1.115	0.003
p.Met295His	substitution	295	Met	His	0.055	0.021
p.Met295Ile	substitution	295	Met	Ile	0.916	0.571
p.Met295Leu	substitution	295	Met	Leu	1.083	0.013
p.Met295Lys	substitution	295	Met	Lys	0.932	0.011
p.Met295Phe	substitution	295	Met	Phe	0.525	0.005
p.Met295Pro	substitution	295	Met	Pro	0.000	0.017
p.Met295Ser	substitution	295	Met	Ser	0.173	0.005
p.Met295Ter	nonsense	295	Met	Ter	0.000	0.007
p.Met295Thr	substitution	295	Met	Thr	0.479	0.075
p.Met295Trp	substitution	295	Met	Trp	0.754	0.002
p.Met295Tyr	substitution	295	Met	Tyr	1.367	0.008
p.Met295Val	substitution	295	Met	Val	1.064	0.022
p.Met56Ala	substitution	56	Met	Ala	0.922	0.161
p.Met56Arg	substitution	56	Met	Arg	0.676	0.075
p.Met56Asn	substitution	56	Met	Asn	0.491	0.023
p.Met56Asp	substitution	56	Met	Asp	1.096	0.014
p.Met56Cys	substitution	56	Met	Cys	0.150	0.017
p.Met56Gln	substitution	56	Met	Gln	1.006	0.012
p.Met56Glu	substitution	56	Met	Glu	0.932	0.008
p.Met56Gly	substitution	56	Met	Gly	0.436	0.021
p.Met56His	substitution	56	Met	His	0.971	0.037
p.Met56Ile	substitution	56	Met	Ile	1.166	0.033
p.Met56Leu	substitution	56	Met	Leu	1.076	0.104
p.Met56Lys	substitution	56	Met	Lys	1.055	0.052

p.Met56Phe	substitution	56	Met	Phe	1.881	0.071
p.Met56Pro	substitution	56	Met	Pro	0.356	0.051
p.Met56Ser	substitution	56	Met	Ser	1.155	0.098
p.Met56Ter	nonsense	56	Met	Ter	0.000	0.026
p.Met56Thr	substitution	56	Met	Thr	0.977	0.134
p.Met56Trp	substitution	56	Met	Trp	0.628	0.005
p.Met56Tyr	substitution	56	Met	Tyr	0.000	0.097
p.Met56Val	substitution	56	Met	Val	0.570	0.105
p.Phe108=	synonymous	108	Phe	NA	1.018	0.218
p.Phe108Ala	substitution	108	Phe	Ala	0.000	0.254
p.Phe108Arg	substitution	108	Phe	Arg	1.094	0.049
p.Phe108Asn	substitution	108	Phe	Asn	0.684	0.012
p.Phe108Asp	substitution	108	Phe	Asp	0.000	0.026
p.Phe108Cys	substitution	108	Phe	Cys	0.784	0.075
p.Phe108Gln	substitution	108	Phe	Gln	1.452	0.015
p.Phe108Glu	substitution	108	Phe	Glu	1.513	0.013
p.Phe108Gly	substitution	108	Phe	Gly	0.538	0.129
p.Phe108His	substitution	108	Phe	His	0.000	0.207
p.Phe108Ile	substitution	108	Phe	Ile	1.127	0.007
p.Phe108Leu	substitution	108	Phe	Leu	1.071	0.052
p.Phe108Lys	substitution	108	Phe	Lys	0.000	0.037
p.Phe108Me	substitution	108	Phe	Met	0.000	0.028
p.Phe108Pro	substitution	108	Phe	Pro	1.183	0.161
p.Phe108Ser	substitution	108	Phe	Ser	0.283	0.066
p.Phe108Ter	nonsense	108	Phe	Ter	0.000	0.163
p.Phe108Thr	substitution	108	Phe	Thr	1.110	0.097
p.Phe108Trp	substitution	108	Phe	Trp	1.139	0.006
p.Phe108Tyr	substitution	108	Phe	Tyr	1.036	0.115
p.Phe108Val	substitution	108	Phe	Val	0.631	0.021
p.Phe125=	synonymous	125	Phe	NA	1.053	0.052
p.Phe125Ala	substitution	125	Phe	Ala	0.361	0.021
p.Phe125Arg	substitution	125	Phe	Arg	0.058	0.025
p.Phe125Asn	substitution	125	Phe	Asn	0.689	0.011
p.Phe125Asp	substitution	125	Phe	Asp	0.000	0.042
p.Phe125Cys	substitution	125	Phe	Cys	0.000	0.034
p.Phe125Gln	substitution	125	Phe	Gln	1.807	0.147
p.Phe125Glu	substitution	125	Phe	Glu	0.517	0.013
p.Phe125Gly	substitution	125	Phe	Gly	0.000	0.009
p.Phe125His	substitution	125	Phe	His	0.112	0.104
p.Phe125Ile	substitution	125	Phe	Ile	1.002	0.013
p.Phe125Leu	substitution	125	Phe	Leu	0.913	0.011
p.Phe125Lys	substitution	125	Phe	Lys	0.000	0.028
p.Phe125Me	substitution	125	Phe	Met	0.900	0.008

p.Phe125Pro	substitution	125	Phe	Pro	0.946	0.021
p.Phe125Ser	substitution	125	Phe	Ser	0.168	0.065
p.Phe125Ter	nonsense	125	Phe	Ter	0.403	0.033
p.Phe125Thr	substitution	125	Phe	Thr	0.109	0.068
p.Phe125Trp	substitution	125	Phe	Trp	0.934	0.006
p.Phe125Tyr	substitution	125	Phe	Tyr	1.379	0.007
p.Phe125Val	substitution	125	Phe	Val	0.772	0.024
p.Phe129=	synonymous	129	Phe	NA	0.510	0.736
p.Phe129Ala	substitution	129	Phe	Ala	0.524	0.022
p.Phe129Arg	substitution	129	Phe	Arg	0.376	0.092
p.Phe129Asn	substitution	129	Phe	Asn	0.000	0.103
p.Phe129Asp	substitution	129	Phe	Asp	0.660	0.012
p.Phe129Cys	substitution	129	Phe	Cys	0.584	0.017
p.Phe129Gln	substitution	129	Phe	Gln	1.067	0.011
p.Phe129Glu	substitution	129	Phe	Glu	1.588	0.023
p.Phe129Gly	substitution	129	Phe	Gly	0.630	0.013
p.Phe129His	substitution	129	Phe	His	0.845	0.019
p.Phe129Ile	substitution	129	Phe	Ile	0.615	0.194
p.Phe129Leu	substitution	129	Phe	Leu	0.955	0.009
p.Phe129Lys	substitution	129	Phe	Lys	0.180	0.066
p.Phe129Me	substitution	129	Phe	Met	1.274	0.015
p.Phe129Pro	substitution	129	Phe	Pro	0.000	0.114
p.Phe129Ser	substitution	129	Phe	Ser	0.610	0.036
p.Phe129Ter	nonsense	129	Phe	Ter	0.000	0.086
p.Phe129Thr	substitution	129	Phe	Thr	0.825	0.017
p.Phe129Trp	substitution	129	Phe	Trp	0.000	0.073
p.Phe129Tyr	substitution	129	Phe	Tyr	1.009	0.018
p.Phe129Val	substitution	129	Phe	Val	0.765	0.015
p.Phe158=	synonymous	158	Phe	NA	1.009	0.010
p.Phe158Ala	substitution	158	Phe	Ala	0.765	0.014
p.Phe158Arg	substitution	158	Phe	Arg	0.734	0.029
p.Phe158Asn	substitution	158	Phe	Asn	0.973	0.018
p.Phe158Asp	substitution	158	Phe	Asp	0.697	0.025
p.Phe158Cys	substitution	158	Phe	Cys	0.968	0.016
p.Phe158Gln	substitution	158	Phe	Gln	0.755	0.012
p.Phe158Glu	substitution	158	Phe	Glu	0.103	0.009
p.Phe158Gly	substitution	158	Phe	Gly	0.666	0.013
p.Phe158His	substitution	158	Phe	His	1.161	0.009
p.Phe158Ile	substitution	158	Phe	Ile	0.972	0.017
p.Phe158Leu	substitution	158	Phe	Leu	0.858	0.005
p.Phe158Lys	substitution	158	Phe	Lys	0.431	0.014
p.Phe158Pro	substitution	158	Phe	Pro	0.000	0.079
p.Phe158Ser	substitution	158	Phe	Ser	0.696	0.026

p.Phe158Ter	nonsense	158	Phe	Ter		0.111	0.010
p.Phe158Thr	substitution	158	Phe	Thr		1.083	0.009
p.Phe158Trp	substitution	158	Phe	Trp		0.994	0.003
p.Phe158Tyr	substitution	158	Phe	Tyr		1.078	0.006
p.Phe158Val	substitution	158	Phe	Val		0.355	0.060
p.Phe163=	synonymous	163	Phe	NA		1.009	0.013
p.Phe163Ala	substitution	163	Phe	Ala		0.949	0.008
p.Phe163Arg	substitution	163	Phe	Arg		0.732	0.006
p.Phe163Asn	substitution	163	Phe	Asn		1.171	0.160
p.Phe163Asp	substitution	163	Phe	Asp		0.131	0.056
p.Phe163Cys	substitution	163	Phe	Cys		0.851	0.004
p.Phe163Gln	substitution	163	Phe	Gln		0.802	0.004
p.Phe163Glu	substitution	163	Phe	Glu		0.603	0.004
p.Phe163Gly	substitution	163	Phe	Gly		0.692	0.012
p.Phe163His	substitution	163	Phe	His		0.845	0.017
p.Phe163Ile	substitution	163	Phe	Ile		1.023	0.018
p.Phe163Leu	substitution	163	Phe	Leu		1.110	0.017
p.Phe163Met	substitution	163	Phe	Met		0.877	0.005
p.Phe163Pro	substitution	163	Phe	Pro		0.900	0.006
p.Phe163Ser	substitution	163	Phe	Ser		0.651	0.011
p.Phe163Ter	nonsense	163	Phe	Ter		0.000	0.010
p.Phe163Thr	substitution	163	Phe	Thr		1.126	0.016
p.Phe163Trp	substitution	163	Phe	Trp		1.033	0.004
p.Phe163Tyr	substitution	163	Phe	Tyr		0.842	0.022
p.Phe163Val	substitution	163	Phe	Val		1.067	0.008
p.Phe183=	synonymous	183	Phe	NA		0.975	0.035
p.Phe183Cys	substitution	183	Phe	Cys		0.620	0.706
p.Phe183Ile	substitution	183	Phe	Ile		1.184	0.012
p.Phe183Leu	substitution	183	Phe	Leu		1.128	0.047
p.Phe183Ser	substitution	183	Phe	Ser		0.827	1.020
p.Phe183Val	substitution	183	Phe	Val		1.098	0.119
p.Phe253=	synonymous	253	Phe	NA		1.378	0.001
p.Phe253Ala	substitution	253	Phe	Ala		0.062	0.020
p.Phe253Arg	substitution	253	Phe	Arg		0.000	0.055
p.Phe253Asn	substitution	253	Phe	Asn		0.000	0.205
p.Phe253Asp	substitution	253	Phe	Asp		0.000	0.059
p.Phe253Cys	substitution	253	Phe	Cys		0.000	0.089
p.Phe253Gln	substitution	253	Phe	Gln		0.000	0.014
p.Phe253Glu	substitution	253	Phe	Glu		0.000	0.045
p.Phe253Gly	substitution	253	Phe	Gly		0.134	0.024
p.Phe253His	substitution	253	Phe	His		0.257	0.011
p.Phe253Ile	substitution	253	Phe	Ile		1.001	0.008
p.Phe253Leu	substitution	253	Phe	Leu		0.770	0.006

p.Phe253Lys	substitution	253	Phe	Lys	0.000	0.049
p.Phe253Me	substitution	253	Phe	Met	1.135	0.002
p.Phe253Pro	substitution	253	Phe	Pro	0.126	0.062
p.Phe253Ser	substitution	253	Phe	Ser	0.000	0.017
p.Phe253Ter	nonsense	253	Phe	Ter	0.000	0.013
p.Phe253Thr	substitution	253	Phe	Thr	0.205	0.022
p.Phe253Trp	substitution	253	Phe	Trp	0.193	0.020
p.Phe253Tyr	substitution	253	Phe	Tyr	1.006	0.006
p.Phe253Val	substitution	253	Phe	Val	0.304	0.088
p.Phe51=	synonymous	51	Phe	NA	0.346	1.140
p.Phe51Ala	substitution	51	Phe	Ala	0.586	0.017
p.Phe51Arg	substitution	51	Phe	Arg	0.000	0.008
p.Phe51Asn	substitution	51	Phe	Asn	0.318	0.013
p.Phe51Asp	substitution	51	Phe	Asp	0.551	0.013
p.Phe51Gln	substitution	51	Phe	Gln	0.439	0.008
p.Phe51Glu	substitution	51	Phe	Glu	0.126	0.015
p.Phe51Gly	substitution	51	Phe	Gly	0.677	0.020
p.Phe51His	substitution	51	Phe	His	0.735	0.012
p.Phe51Ile	substitution	51	Phe	Ile	0.947	0.006
p.Phe51Leu	substitution	51	Phe	Leu	0.888	0.043
p.Phe51Lys	substitution	51	Phe	Lys	0.317	0.013
p.Phe51Met	substitution	51	Phe	Met	0.750	0.010
p.Phe51Pro	substitution	51	Phe	Pro	0.965	0.029
p.Phe51Ser	substitution	51	Phe	Ser	0.451	0.009
p.Phe51Ter	nonsense	51	Phe	Ter	0.000	0.021
p.Phe51Thr	substitution	51	Phe	Thr	1.032	0.047
p.Phe51Trp	substitution	51	Phe	Trp	0.839	0.005
p.Phe51Tyr	substitution	51	Phe	Tyr	0.626	0.015
p.Phe51Val	substitution	51	Phe	Val	1.130	0.010
p.Phe77Ala	substitution	77	Phe	Ala	0.120	0.027
p.Phe77Arg	substitution	77	Phe	Arg	0.000	0.010
p.Phe77Asn	substitution	77	Phe	Asn	0.000	0.013
p.Phe77Asp	substitution	77	Phe	Asp	0.049	0.012
p.Phe77Cys	substitution	77	Phe	Cys	0.128	0.006
p.Phe77Gln	substitution	77	Phe	Gln	0.100	0.011
p.Phe77Glu	substitution	77	Phe	Glu	0.000	0.011
p.Phe77Gly	substitution	77	Phe	Gly	0.000	0.011
p.Phe77His	substitution	77	Phe	His	0.000	0.018
p.Phe77Ile	substitution	77	Phe	Ile	0.187	0.020
p.Phe77Leu	substitution	77	Phe	Leu	0.000	0.019
p.Phe77Lys	substitution	77	Phe	Lys	0.000	0.038
p.Phe77Met	substitution	77	Phe	Met	0.000	0.017
p.Phe77Pro	substitution	77	Phe	Pro	0.000	0.046

p.Phe77Ser	substitution	77	Phe	Ser		0.094	0.057
p.Phe77Ter	nonsense	77	Phe	Ter		0.176	0.006
p.Phe77Thr	substitution	77	Phe	Thr		0.000	0.032
p.Phe77Trp	substitution	77	Phe	Trp		0.823	0.003
p.Phe77Tyr	substitution	77	Phe	Tyr		0.685	0.005
p.Phe77Val	substitution	77	Phe	Val		0.000	0.010
p.Pro101=	synonymous	101	Pro	NA		0.932	0.003
p.Pro101Ala	substitution	101	Pro	Ala		0.965	0.020
p.Pro101Arg	substitution	101	Pro	Arg		0.000	0.283
p.Pro101Asn	substitution	101	Pro	Asn		0.587	0.038
p.Pro101Asp	substitution	101	Pro	Asp		0.000	0.099
p.Pro101Cys	substitution	101	Pro	Cys		0.000	0.055
p.Pro101Gln	substitution	101	Pro	Gln		0.661	0.063
p.Pro101Glu	substitution	101	Pro	Glu		0.658	0.009
p.Pro101Gly	substitution	101	Pro	Gly		0.323	0.031
p.Pro101His	substitution	101	Pro	His		0.980	0.021
p.Pro101Ile	substitution	101	Pro	Ile		0.784	0.010
p.Pro101Leu	substitution	101	Pro	Leu		0.490	0.079
p.Pro101Lys	substitution	101	Pro	Lys		0.000	0.978
p.Pro101Met	substitution	101	Pro	Met		0.341	0.097
p.Pro101Phe	substitution	101	Pro	Phe		0.458	0.013
p.Pro101Ser	substitution	101	Pro	Ser		0.822	0.050
p.Pro101Ter	nonsense	101	Pro	Ter		0.000	0.065
p.Pro101Thr	substitution	101	Pro	Thr		0.912	0.021
p.Pro101Trp	substitution	101	Pro	Trp		0.000	0.030
p.Pro101Tyr	substitution	101	Pro	Tyr		0.000	0.115
p.Pro101Val	substitution	101	Pro	Val		0.678	0.073
p.Pro105=	synonymous	105	Pro	NA		0.995	0.019
p.Pro105Ala	substitution	105	Pro	Ala		0.910	0.005
p.Pro105Arg	substitution	105	Pro	Arg		0.802	0.011
p.Pro105Asn	substitution	105	Pro	Asn		0.455	0.009
p.Pro105Asp	substitution	105	Pro	Asp		0.307	0.012
p.Pro105Cys	substitution	105	Pro	Cys		0.369	0.011
p.Pro105Gln	substitution	105	Pro	Gln		0.845	0.005
p.Pro105Glu	substitution	105	Pro	Glu		0.437	0.016
p.Pro105Gly	substitution	105	Pro	Gly		0.952	0.008
p.Pro105His	substitution	105	Pro	His		0.885	0.004
p.Pro105Ile	substitution	105	Pro	Ile		0.597	0.005
p.Pro105Leu	substitution	105	Pro	Leu		0.646	0.035
p.Pro105Lys	substitution	105	Pro	Lys		0.891	0.019
p.Pro105Met	substitution	105	Pro	Met		0.747	0.012
p.Pro105Phe	substitution	105	Pro	Phe		1.145	0.003
p.Pro105Ser	substitution	105	Pro	Ser		0.769	0.056

p.Pro105Ter	nonsense	105	Pro	Ter		0.002	0.029
p.Pro105Thr	substitution	105	Pro	Thr		0.911	0.023
p.Pro105Trp	substitution	105	Pro	Trp		0.669	0.020
p.Pro105Tyr	substitution	105	Pro	Tyr		0.007	0.010
p.Pro105Val	substitution	105	Pro	Val		0.410	0.005
p.Pro106=	synonymous	106	Pro	NA		0.964	0.033
p.Pro106Ala	substitution	106	Pro	Ala		0.925	0.010
p.Pro106Arg	substitution	106	Pro	Arg		0.966	0.004
p.Pro106Asn	substitution	106	Pro	Asn		1.014	0.007
p.Pro106Asp	substitution	106	Pro	Asp		0.889	0.005
p.Pro106Cys	substitution	106	Pro	Cys		1.078	0.011
p.Pro106Gln	substitution	106	Pro	Gln		0.874	0.012
p.Pro106Glu	substitution	106	Pro	Glu		1.140	0.008
p.Pro106Gly	substitution	106	Pro	Gly		0.772	0.025
p.Pro106His	substitution	106	Pro	His		1.005	0.005
p.Pro106Ile	substitution	106	Pro	Ile		0.779	0.007
p.Pro106Leu	substitution	106	Pro	Leu		0.983	0.029
p.Pro106Lys	substitution	106	Pro	Lys		1.082	0.009
p.Pro106Met	substitution	106	Pro	Met		0.381	0.018
p.Pro106Phe	substitution	106	Pro	Phe		1.061	0.005
p.Pro106Ser	substitution	106	Pro	Ser		1.019	0.003
p.Pro106Ter	nonsense	106	Pro	Ter		0.121	0.015
p.Pro106Thr	substitution	106	Pro	Thr		1.009	0.003
p.Pro106Trp	substitution	106	Pro	Trp		0.879	0.009
p.Pro106Tyr	substitution	106	Pro	Tyr		0.486	0.010
p.Pro106Val	substitution	106	Pro	Val		1.160	0.012
p.Pro119=	synonymous	119	Pro	NA		0.836	0.069
p.Pro119Ala	substitution	119	Pro	Ala		0.923	0.003
p.Pro119Arg	substitution	119	Pro	Arg		0.181	0.011
p.Pro119Asn	substitution	119	Pro	Asn		0.388	0.008
p.Pro119Asp	substitution	119	Pro	Asp		0.128	0.007
p.Pro119Cys	substitution	119	Pro	Cys		0.826	0.008
p.Pro119Glu	substitution	119	Pro	Glu		0.451	0.034
p.Pro119Gly	substitution	119	Pro	Gly		0.279	0.007
p.Pro119His	substitution	119	Pro	His		0.782	0.002
p.Pro119Ile	substitution	119	Pro	Ile		0.000	0.011
p.Pro119Leu	substitution	119	Pro	Leu		0.021	0.029
p.Pro119Lys	substitution	119	Pro	Lys		0.110	0.061
p.Pro119Met	substitution	119	Pro	Met		0.000	0.014
p.Pro119Phe	substitution	119	Pro	Phe		0.788	0.008
p.Pro119Ser	substitution	119	Pro	Ser		0.834	0.032
p.Pro119Ter	nonsense	119	Pro	Ter		0.000	0.424
p.Pro119Thr	substitution	119	Pro	Thr		0.981	0.033

p.Pro119Trp	substitution	119	Pro	Trp	0.837	0.009
p.Pro119Tyr	substitution	119	Pro	Tyr	0.697	0.005
p.Pro119Val	substitution	119	Pro	Val	0.787	0.008
p.Pro127=	synonymous	127	Pro	NA	0.965	0.075
p.Pro127Ala	substitution	127	Pro	Ala	0.703	0.073
p.Pro127Arg	substitution	127	Pro	Arg	1.025	0.035
p.Pro127Asn	substitution	127	Pro	Asn	0.000	0.875
p.Pro127Asp	substitution	127	Pro	Asp	0.404	0.350
p.Pro127Cys	substitution	127	Pro	Cys	0.084	0.034
p.Pro127Gln	substitution	127	Pro	Gln	1.174	0.037
p.Pro127Gly	substitution	127	Pro	Gly	0.568	0.648
p.Pro127His	substitution	127	Pro	His	0.266	0.150
p.Pro127Ile	substitution	127	Pro	Ile	1.073	0.012
p.Pro127Leu	substitution	127	Pro	Leu	1.155	0.068
p.Pro127Lys	substitution	127	Pro	Lys	0.820	0.131
p.Pro127Met	substitution	127	Pro	Met	0.067	0.044
p.Pro127Phe	substitution	127	Pro	Phe	0.827	0.066
p.Pro127Ser	substitution	127	Pro	Ser	0.844	0.057
p.Pro127Ter	nonsense	127	Pro	Ter	0.647	0.045
p.Pro127Thr	substitution	127	Pro	Thr	0.886	0.094
p.Pro127Trp	substitution	127	Pro	Trp	1.135	0.021
p.Pro127Tyr	substitution	127	Pro	Tyr	0.000	0.898
p.Pro127Val	substitution	127	Pro	Val	0.534	0.070
p.Pro138=	synonymous	138	Pro	NA	0.976	0.006
p.Pro138Ala	substitution	138	Pro	Ala	1.036	0.003
p.Pro138Arg	substitution	138	Pro	Arg	0.772	0.022
p.Pro138Asn	substitution	138	Pro	Asn	0.895	0.004
p.Pro138Asp	substitution	138	Pro	Asp	0.788	0.006
p.Pro138Cys	substitution	138	Pro	Cys	0.816	0.004
p.Pro138Gln	substitution	138	Pro	Gln	1.123	0.007
p.Pro138Glu	substitution	138	Pro	Glu	0.945	0.107
p.Pro138Gly	substitution	138	Pro	Gly	0.628	0.008
p.Pro138His	substitution	138	Pro	His	0.754	0.004
p.Pro138Ile	substitution	138	Pro	Ile	0.696	0.022
p.Pro138Leu	substitution	138	Pro	Leu	0.821	0.015
p.Pro138Lys	substitution	138	Pro	Lys	0.836	0.030
p.Pro138Met	substitution	138	Pro	Met	0.939	0.004
p.Pro138Phe	substitution	138	Pro	Phe	0.582	0.005
p.Pro138Ser	substitution	138	Pro	Ser	0.887	0.004
p.Pro138Ter	nonsense	138	Pro	Ter	0.121	0.049
p.Pro138Thr	substitution	138	Pro	Thr	0.808	0.006
p.Pro138Trp	substitution	138	Pro	Trp	0.339	0.008
p.Pro138Tyr	substitution	138	Pro	Tyr	0.511	0.005

p.Pro138Val	substitution	138	Pro	Val	0.695	0.006
p.Pro159=	synonymous	159	Pro	NA	1.074	0.076
p.Pro159Ala	substitution	159	Pro	Ala	0.951	0.015
p.Pro159Arg	substitution	159	Pro	Arg	0.805	0.009
p.Pro159Asn	substitution	159	Pro	Asn	1.075	0.006
p.Pro159Asp	substitution	159	Pro	Asp	0.571	0.008
p.Pro159Cys	substitution	159	Pro	Cys	1.001	0.005
p.Pro159Gln	substitution	159	Pro	Gln	0.834	0.014
p.Pro159Glu	substitution	159	Pro	Glu	0.694	0.008
p.Pro159Gly	substitution	159	Pro	Gly	0.726	0.006
p.Pro159His	substitution	159	Pro	His	1.015	0.006
p.Pro159Ile	substitution	159	Pro	Ile	0.663	0.008
p.Pro159Leu	substitution	159	Pro	Leu	0.785	0.029
p.Pro159Lys	substitution	159	Pro	Lys	0.786	0.008
p.Pro159Met	substitution	159	Pro	Met	0.635	0.018
p.Pro159Phe	substitution	159	Pro	Phe	0.652	0.030
p.Pro159Ser	substitution	159	Pro	Ser	0.723	0.065
p.Pro159Ter	nonsense	159	Pro	Ter	0.348	0.018
p.Pro159Thr	substitution	159	Pro	Thr	0.730	0.033
p.Pro159Trp	substitution	159	Pro	Trp	0.265	0.014
p.Pro159Tyr	substitution	159	Pro	Tyr	0.413	0.011
p.Pro159Val	substitution	159	Pro	Val	1.109	0.056
p.Pro208=	synonymous	208	Pro	NA	1.252	0.014
p.Pro208Ala	substitution	208	Pro	Ala	0.497	0.038
p.Pro208Arg	substitution	208	Pro	Arg	0.960	0.033
p.Pro208Asn	substitution	208	Pro	Asn	0.000	0.050
p.Pro208Asp	substitution	208	Pro	Asp	0.182	0.011
p.Pro208Cys	substitution	208	Pro	Cys	0.060	0.019
p.Pro208Gln	substitution	208	Pro	Gln	0.781	0.006
p.Pro208Glu	substitution	208	Pro	Glu	0.000	0.023
p.Pro208Gly	substitution	208	Pro	Gly	1.052	0.098
p.Pro208His	substitution	208	Pro	His	0.899	0.002
p.Pro208Ile	substitution	208	Pro	Ile	1.058	0.004
p.Pro208Leu	substitution	208	Pro	Leu	0.814	0.022
p.Pro208Lys	substitution	208	Pro	Lys	0.218	0.020
p.Pro208Met	substitution	208	Pro	Met	0.983	0.003
p.Pro208Phe	substitution	208	Pro	Phe	1.086	0.004
p.Pro208Ser	substitution	208	Pro	Ser	0.785	0.047
p.Pro208Ter	nonsense	208	Pro	Ter	0.000	0.042
p.Pro208Thr	substitution	208	Pro	Thr	0.649	0.003
p.Pro208Tyr	substitution	208	Pro	Tyr	1.171	0.004
p.Pro208Val	substitution	208	Pro	Val	0.414	0.028
p.Pro241=	synonymous	241	Pro	NA	1.083	0.005

p.Pro241Ala	substitution	241	Pro	Ala	0.467	0.050
p.Pro241Arg	substitution	241	Pro	Arg	0.000	0.102
p.Pro241Asp	substitution	241	Pro	Asp	0.555	0.017
p.Pro241Cys	substitution	241	Pro	Cys	1.079	0.011
p.Pro241Gln	substitution	241	Pro	Gln	1.231	0.004
p.Pro241His	substitution	241	Pro	His	0.681	0.035
p.Pro241Leu	substitution	241	Pro	Leu	0.000	0.057
p.Pro241Lys	substitution	241	Pro	Lys	0.000	0.017
p.Pro241Met	substitution	241	Pro	Met	1.480	0.004
p.Pro241Ser	substitution	241	Pro	Ser	1.136	0.091
p.Pro241Ter	nonsense	241	Pro	Ter	0.777	0.301
p.Pro241Thr	substitution	241	Pro	Thr	0.601	0.084
p.Pro241Trp	substitution	241	Pro	Trp	1.113	0.007
p.Pro241Tyr	substitution	241	Pro	Tyr	0.000	0.070
p.Pro264=	synonymous	264	Pro	NA	0.488	0.755
p.Pro264Ala	substitution	264	Pro	Ala	0.180	0.077
p.Pro264Cys	substitution	264	Pro	Cys	0.000	0.067
p.Pro264Gln	substitution	264	Pro	Gln	0.764	0.033
p.Pro264Glu	substitution	264	Pro	Glu	0.280	0.011
p.Pro264Leu	substitution	264	Pro	Leu	0.306	0.769
p.Pro264Lys	substitution	264	Pro	Lys	0.000	0.131
p.Pro264Met	substitution	264	Pro	Met	0.000	0.075
p.Pro264Ser	substitution	264	Pro	Ser	1.315	0.071
p.Pro264Ter	nonsense	264	Pro	Ter	0.788	0.063
p.Pro264Thr	substitution	264	Pro	Thr	1.610	0.062
p.Pro264Trp	substitution	264	Pro	Trp	0.013	0.165
p.Pro264Tyr	substitution	264	Pro	Tyr	0.000	0.019
p.Pro264Val	substitution	264	Pro	Val	0.000	0.124
p.Pro302=	synonymous	302	Pro	NA	0.804	0.099
p.Pro302Ala	substitution	302	Pro	Ala	0.945	0.109
p.Pro302Arg	substitution	302	Pro	Arg	0.227	0.019
p.Pro302Asn	substitution	302	Pro	Asn	0.000	0.032
p.Pro302Asp	substitution	302	Pro	Asp	0.923	0.005
p.Pro302Cys	substitution	302	Pro	Cys	0.896	0.003
p.Pro302Gln	substitution	302	Pro	Gln	0.295	0.003
p.Pro302Gly	substitution	302	Pro	Gly	1.414	0.004
p.Pro302Ile	substitution	302	Pro	Ile	0.000	0.032
p.Pro302Leu	substitution	302	Pro	Leu	0.915	0.061
p.Pro302Lys	substitution	302	Pro	Lys	0.332	0.018
p.Pro302Met	substitution	302	Pro	Met	1.064	0.010
p.Pro302Phe	substitution	302	Pro	Phe	0.227	0.019
p.Pro302Ser	substitution	302	Pro	Ser	0.347	0.031
p.Pro302Ter	nonsense	302	Pro	Ter	0.000	0.133

p.Pro302Thr	substitution	302	Pro	Thr	1.129	0.026
p.Pro302Trp	substitution	302	Pro	Trp	0.910	0.032
p.Pro302Tyr	substitution	302	Pro	Tyr	0.655	0.150
p.Pro302Val	substitution	302	Pro	Val	1.084	0.227
p.Pro309=	synonymous	309	Pro	NA	1.150	0.011
p.Pro309Ala	substitution	309	Pro	Ala	1.098	0.051
p.Pro309Arg	substitution	309	Pro	Arg	0.849	0.036
p.Pro309Asn	substitution	309	Pro	Asn	1.349	0.001
p.Pro309Cys	substitution	309	Pro	Cys	0.160	0.258
p.Pro309Gln	substitution	309	Pro	Gln	0.801	0.003
p.Pro309Glu	substitution	309	Pro	Glu	0.066	0.006
p.Pro309Gly	substitution	309	Pro	Gly	1.320	0.002
p.Pro309His	substitution	309	Pro	His	0.829	0.002
p.Pro309Ile	substitution	309	Pro	Ile	0.405	0.013
p.Pro309Leu	substitution	309	Pro	Leu	1.052	0.008
p.Pro309Lys	substitution	309	Pro	Lys	1.052	0.004
p.Pro309Met	substitution	309	Pro	Met	0.000	0.020
p.Pro309Phe	substitution	309	Pro	Phe	1.182	0.003
p.Pro309Ser	substitution	309	Pro	Ser	0.779	0.135
p.Pro309Ter	nonsense	309	Pro	Ter	0.000	0.009
p.Pro309Thr	substitution	309	Pro	Thr	0.592	0.013
p.Pro309Trp	substitution	309	Pro	Trp	0.346	0.035
p.Pro309Tyr	substitution	309	Pro	Tyr	0.000	0.028
p.Pro309Val	substitution	309	Pro	Val	1.268	0.016
p.Pro313=	synonymous	313	Pro	NA	1.152	0.031
p.Pro313Ala	substitution	313	Pro	Ala	1.108	0.011
p.Pro313Arg	substitution	313	Pro	Arg	1.055	0.008
p.Pro313Asn	substitution	313	Pro	Asn	0.665	0.004
p.Pro313Asp	substitution	313	Pro	Asp	0.022	0.010
p.Pro313Cys	substitution	313	Pro	Cys	0.469	0.006
p.Pro313Gln	substitution	313	Pro	Gln	0.115	0.118
p.Pro313Gly	substitution	313	Pro	Gly	0.835	0.057
p.Pro313His	substitution	313	Pro	His	0.389	0.031
p.Pro313Ile	substitution	313	Pro	Ile	1.354	0.002
p.Pro313Leu	substitution	313	Pro	Leu	0.935	0.110
p.Pro313Lys	substitution	313	Pro	Lys	0.677	0.018
p.Pro313Phe	substitution	313	Pro	Phe	0.000	0.105
p.Pro313Ser	substitution	313	Pro	Ser	1.151	0.005
p.Pro313Ter	nonsense	313	Pro	Ter	0.000	0.043
p.Pro313Thr	substitution	313	Pro	Thr	0.583	0.122
p.Pro313Trp	substitution	313	Pro	Trp	0.005	0.095
p.Pro313Tyr	substitution	313	Pro	Tyr	1.273	0.002
p.Pro313Val	substitution	313	Pro	Val	1.030	0.013

p.Pro324=	synonymous	324	Pro	NA	0.950	0.020
p.Pro324Ala	substitution	324	Pro	Ala	0.580	0.072
p.Pro324Arg	substitution	324	Pro	Arg	1.005	0.022
p.Pro324Asp	substitution	324	Pro	Asp	1.402	0.004
p.Pro324Cys	substitution	324	Pro	Cys	0.210	0.015
p.Pro324Gln	substitution	324	Pro	Gln	0.781	0.157
p.Pro324Glu	substitution	324	Pro	Glu	0.054	0.014
p.Pro324Gly	substitution	324	Pro	Gly	0.462	0.040
p.Pro324His	substitution	324	Pro	His	1.118	0.003
p.Pro324Ile	substitution	324	Pro	Ile	0.217	0.026
p.Pro324Leu	substitution	324	Pro	Leu	0.923	0.018
p.Pro324Lys	substitution	324	Pro	Lys	0.468	0.042
p.Pro324Met	substitution	324	Pro	Met	0.037	0.026
p.Pro324Phe	substitution	324	Pro	Phe	0.000	0.008
p.Pro324Ser	substitution	324	Pro	Ser	0.690	0.158
p.Pro324Ter	nonsense	324	Pro	Ter	0.000	0.102
p.Pro324Thr	substitution	324	Pro	Thr	1.095	0.003
p.Pro324Tyr	substitution	324	Pro	Tyr	0.521	0.005
p.Pro324Val	substitution	324	Pro	Val	0.729	0.103
p.Pro327=	synonymous	327	Pro	NA	0.888	0.015
p.Pro327Arg	substitution	327	Pro	Arg	0.000	0.129
p.Pro327Asn	substitution	327	Pro	Asn	0.000	0.140
p.Pro327Cys	substitution	327	Pro	Cys	0.000	0.035
p.Pro327Gln	substitution	327	Pro	Gln	1.206	0.008
p.Pro327His	substitution	327	Pro	His	0.940	0.004
p.Pro327Leu	substitution	327	Pro	Leu	0.317	0.071
p.Pro327Ser	substitution	327	Pro	Ser	0.318	0.621
p.Pro327Thr	substitution	327	Pro	Thr	0.595	0.688
p.Pro47=	synonymous	47	Pro	NA	1.147	0.032
p.Pro47Ala	substitution	47	Pro	Ala	0.459	0.048
p.Pro47Arg	substitution	47	Pro	Arg	0.919	0.016
p.Pro47Asn	substitution	47	Pro	Asn	1.003	0.004
p.Pro47Asp	substitution	47	Pro	Asp	0.739	0.037
p.Pro47Cys	substitution	47	Pro	Cys	0.475	0.008
p.Pro47Gln	substitution	47	Pro	Gln	0.893	0.004
p.Pro47Glu	substitution	47	Pro	Glu	0.000	0.501
p.Pro47Gly	substitution	47	Pro	Gly	0.598	0.012
p.Pro47His	substitution	47	Pro	His	0.807	0.006
p.Pro47Ile	substitution	47	Pro	Ile	0.136	0.014
p.Pro47Leu	substitution	47	Pro	Leu	0.394	0.009
p.Pro47Lys	substitution	47	Pro	Lys	1.273	0.005
p.Pro47Met	substitution	47	Pro	Met	0.000	0.031
p.Pro47Phe	substitution	47	Pro	Phe	0.643	0.008

p.Pro47Ser	substitution	47	Pro	Ser	0.846	0.031
p.Pro47Ter	nonsense	47	Pro	Ter	0.000	0.015
p.Pro47Thr	substitution	47	Pro	Thr	0.914	0.006
p.Pro47Trp	substitution	47	Pro	Trp	0.318	0.024
p.Pro47Tyr	substitution	47	Pro	Tyr	0.472	0.008
p.Pro47Val	substitution	47	Pro	Val	0.889	0.007
p.Ser141=	synonymous	141	Ser	NA	0.838	0.002
p.Ser141Ala	substitution	141	Ser	Ala	1.032	0.007
p.Ser141Arg	substitution	141	Ser	Arg	0.779	0.008
p.Ser141Asn	substitution	141	Ser	Asn	1.116	0.003
p.Ser141Asp	substitution	141	Ser	Asp	0.589	0.005
p.Ser141Gln	substitution	141	Ser	Gln	0.866	0.008
p.Ser141Glu	substitution	141	Ser	Glu	0.703	0.010
p.Ser141Gly	substitution	141	Ser	Gly	0.880	0.024
p.Ser141His	substitution	141	Ser	His	0.896	0.004
p.Ser141Ile	substitution	141	Ser	Ile	0.399	0.004
p.Ser141Leu	substitution	141	Ser	Leu	0.818	0.002
p.Ser141Lys	substitution	141	Ser	Lys	0.642	0.008
p.Ser141Met	substitution	141	Ser	Met	1.020	0.006
p.Ser141Phe	substitution	141	Ser	Phe	0.646	0.007
p.Ser141Pro	substitution	141	Ser	Pro	0.105	0.015
p.Ser141Ter	nonsense	141	Ser	Ter	0.000	0.010
p.Ser141Thr	substitution	141	Ser	Thr	0.743	0.005
p.Ser141Trp	substitution	141	Ser	Trp	0.399	0.007
p.Ser141Tyr	substitution	141	Ser	Tyr	0.624	0.007
p.Ser141Val	substitution	141	Ser	Val	0.740	0.004
p.Ser146=	synonymous	146	Ser	NA	0.889	0.078
p.Ser146Ala	substitution	146	Ser	Ala	0.722	0.040
p.Ser146Arg	substitution	146	Ser	Arg	0.836	0.022
p.Ser146Asn	substitution	146	Ser	Asn	1.034	0.036
p.Ser146Asp	substitution	146	Ser	Asp	0.282	0.041
p.Ser146Cys	substitution	146	Ser	Cys	0.267	0.448
p.Ser146Gln	substitution	146	Ser	Gln	1.121	0.010
p.Ser146Glu	substitution	146	Ser	Glu	0.000	0.011
p.Ser146Gly	substitution	146	Ser	Gly	0.941	0.012
p.Ser146His	substitution	146	Ser	His	0.445	0.159
p.Ser146Ile	substitution	146	Ser	Ile	0.650	0.075
p.Ser146Leu	substitution	146	Ser	Leu	0.188	0.009
p.Ser146Lys	substitution	146	Ser	Lys	0.191	0.015
p.Ser146Met	substitution	146	Ser	Met	0.494	0.008
p.Ser146Phe	substitution	146	Ser	Phe	0.813	0.025
p.Ser146Pro	substitution	146	Ser	Pro	0.118	0.011
p.Ser146Ter	nonsense	146	Ser	Ter	0.395	0.013

p.Ser146Thr	substitution	146	Ser	Thr	0.635	0.175
p.Ser146Trp	substitution	146	Ser	Trp	0.832	0.013
p.Ser146Tyr	substitution	146	Ser	Tyr	0.400	0.045
p.Ser146Val	substitution	146	Ser	Val	0.607	0.027
p.Ser147=	synonymous	147	Ser	NA	0.824	0.065
p.Ser147Ala	substitution	147	Ser	Ala	0.000	0.641
p.Ser147Arg	substitution	147	Ser	Arg	0.182	0.320
p.Ser147Asn	substitution	147	Ser	Asn	0.000	0.757
p.Ser147Asp	substitution	147	Ser	Asp	0.483	0.027
p.Ser147Cys	substitution	147	Ser	Cys	0.305	0.454
p.Ser147Glu	substitution	147	Ser	Glu	0.110	0.047
p.Ser147Gly	substitution	147	Ser	Gly	0.000	0.610
p.Ser147His	substitution	147	Ser	His	0.000	0.165
p.Ser147Ile	substitution	147	Ser	Ile	0.685	0.466
p.Ser147Leu	substitution	147	Ser	Leu	0.000	0.260
p.Ser147Phe	substitution	147	Ser	Phe	0.000	0.217
p.Ser147Pro	substitution	147	Ser	Pro	0.000	0.812
p.Ser147Ter	nonsense	147	Ser	Ter	0.000	0.812
p.Ser147Thr	substitution	147	Ser	Thr	0.316	0.126
p.Ser147Trp	substitution	147	Ser	Trp	0.566	0.129
p.Ser147Tyr	substitution	147	Ser	Tyr	0.000	0.135
p.Ser147Val	substitution	147	Ser	Val	0.000	0.137
p.Ser165=	synonymous	165	Ser	NA	0.611	0.032
p.Ser165Ala	substitution	165	Ser	Ala	0.507	0.110
p.Ser165Arg	substitution	165	Ser	Arg	0.737	0.015
p.Ser165Asn	substitution	165	Ser	Asn	0.841	0.006
p.Ser165Asp	substitution	165	Ser	Asp	0.000	0.016
p.Ser165Cys	substitution	165	Ser	Cys	0.959	0.009
p.Ser165Gln	substitution	165	Ser	Gln	0.963	0.008
p.Ser165Glu	substitution	165	Ser	Glu	0.481	0.014
p.Ser165Gly	substitution	165	Ser	Gly	1.008	0.038
p.Ser165His	substitution	165	Ser	His	0.887	0.007
p.Ser165Ile	substitution	165	Ser	Ile	0.697	0.008
p.Ser165Leu	substitution	165	Ser	Leu	0.342	0.228
p.Ser165Lys	substitution	165	Ser	Lys	0.298	0.018
p.Ser165Met	substitution	165	Ser	Met	0.406	0.012
p.Ser165Phe	substitution	165	Ser	Phe	0.579	0.013
p.Ser165Pro	substitution	165	Ser	Pro	1.090	0.010
p.Ser165Ter	nonsense	165	Ser	Ter	0.000	0.026
p.Ser165Thr	substitution	165	Ser	Thr	1.181	0.016
p.Ser165Trp	substitution	165	Ser	Trp	0.312	0.010
p.Ser165Tyr	substitution	165	Ser	Tyr	0.811	0.007
p.Ser165Val	substitution	165	Ser	Val	0.772	0.022

p.Ser184=	synonymous	184	Ser	NA		1.446	0.381
p.Ser184Arg	substitution	184	Ser	Arg		1.062	0.691
p.Ser184Asn	substitution	184	Ser	Asn		0.999	0.016
p.Ser184Cys	substitution	184	Ser	Cys		1.327	0.025
p.Ser184Gly	substitution	184	Ser	Gly		0.712	0.437
p.Ser184Ile	substitution	184	Ser	Ile		0.889	0.033
p.Ser184Thr	substitution	184	Ser	Thr		1.144	0.051
p.Ser262Ala	substitution	262	Ser	Ala		1.319	0.036
p.Ser262Arg	substitution	262	Ser	Arg		1.248	0.094
p.Ser262Asn	substitution	262	Ser	Asn		1.402	0.010
p.Ser262Cys	substitution	262	Ser	Cys		0.007	0.390
p.Ser262Gln	substitution	262	Ser	Gln		0.575	0.023
p.Ser262Glu	substitution	262	Ser	Glu		1.626	0.004
p.Ser262Gly	substitution	262	Ser	Gly		1.415	0.008
p.Ser262His	substitution	262	Ser	His		0.042	0.034
p.Ser262Ile	substitution	262	Ser	Ile		0.979	0.158
p.Ser262Leu	substitution	262	Ser	Leu		0.000	0.095
p.Ser262Met	substitution	262	Ser	Met		0.000	0.176
p.Ser262Phe	substitution	262	Ser	Phe		0.000	0.096
p.Ser262Thr	substitution	262	Ser	Thr		0.000	0.140
p.Ser262Trp	substitution	262	Ser	Trp		0.000	0.097
p.Ser262Val	substitution	262	Ser	Val		0.746	0.047
p.Ser284=	synonymous	284	Ser	NA		1.377	0.023
p.Ser284Ala	substitution	284	Ser	Ala		1.526	0.006
p.Ser284Arg	substitution	284	Ser	Arg		1.268	0.013
p.Ser284Asn	substitution	284	Ser	Asn		0.807	0.053
p.Ser284Asp	substitution	284	Ser	Asp		0.236	0.052
p.Ser284Cys	substitution	284	Ser	Cys		0.810	0.016
p.Ser284Gln	substitution	284	Ser	Gln		1.625	0.010
p.Ser284Glu	substitution	284	Ser	Glu		0.000	0.072
p.Ser284Gly	substitution	284	Ser	Gly		0.899	0.035
p.Ser284Ile	substitution	284	Ser	Ile		1.235	0.011
p.Ser284Leu	substitution	284	Ser	Leu		0.000	0.112
p.Ser284Pro	substitution	284	Ser	Pro		0.000	0.065
p.Ser284Thr	substitution	284	Ser	Thr		1.205	0.106
p.Ser284Trp	substitution	284	Ser	Trp		0.309	0.013
p.Ser284Val	substitution	284	Ser	Val		0.000	0.090
p.Ser288=	synonymous	288	Ser	NA		0.606	0.114
p.Ser288Ala	substitution	288	Ser	Ala		1.027	0.008
p.Ser288Arg	substitution	288	Ser	Arg		0.002	0.018
p.Ser288Asn	substitution	288	Ser	Asn		0.558	0.015
p.Ser288Asp	substitution	288	Ser	Asp		1.151	0.004
p.Ser288Cys	substitution	288	Ser	Cys		0.869	0.004

p.Ser288Gln	substitution	288	Ser	Gln	1.146	0.006
p.Ser288Glu	substitution	288	Ser	Glu	1.174	0.040
p.Ser288Gly	substitution	288	Ser	Gly	1.048	0.008
p.Ser288His	substitution	288	Ser	His	0.000	0.007
p.Ser288Ile	substitution	288	Ser	Ile	1.084	0.014
p.Ser288Leu	substitution	288	Ser	Leu	1.048	0.012
p.Ser288Lys	substitution	288	Ser	Lys	0.572	0.015
p.Ser288Met	substitution	288	Ser	Met	1.308	0.002
p.Ser288Phe	substitution	288	Ser	Phe	0.780	0.004
p.Ser288Pro	substitution	288	Ser	Pro	0.923	0.025
p.Ser288Ter	nonsense	288	Ser	Ter	0.036	0.053
p.Ser288Thr	substitution	288	Ser	Thr	0.764	0.042
p.Ser288Trp	substitution	288	Ser	Trp	1.142	0.004
p.Ser288Tyr	substitution	288	Ser	Tyr	0.467	0.010
p.Ser288Val	substitution	288	Ser	Val	0.964	0.013
p.Ser28=	synonymous	28	Ser	NA	0.985	0.006
p.Ser28Ala	substitution	28	Ser	Ala	0.551	0.029
p.Ser28Arg	substitution	28	Ser	Arg	0.000	0.015
p.Ser28Asn	substitution	28	Ser	Asn	0.000	0.009
p.Ser28Asp	substitution	28	Ser	Asp	0.000	0.005
p.Ser28Cys	substitution	28	Ser	Cys	0.112	0.167
p.Ser28Gln	substitution	28	Ser	Gln	0.000	0.055
p.Ser28Glu	substitution	28	Ser	Glu	0.000	0.002
p.Ser28Gly	substitution	28	Ser	Gly	0.901	0.007
p.Ser28His	substitution	28	Ser	His	0.000	0.061
p.Ser28Ile	substitution	28	Ser	Ile	0.000	0.004
p.Ser28Leu	substitution	28	Ser	Leu	0.000	0.037
p.Ser28Lys	substitution	28	Ser	Lys	0.000	0.003
p.Ser28Met	substitution	28	Ser	Met	0.000	0.008
p.Ser28Phe	substitution	28	Ser	Phe	0.000	0.005
p.Ser28Pro	substitution	28	Ser	Pro	0.000	0.046
p.Ser28Ter	nonsense	28	Ser	Ter	0.000	0.014
p.Ser28Thr	substitution	28	Ser	Thr	1.018	0.113
p.Ser28Trp	substitution	28	Ser	Trp	0.000	0.006
p.Ser28Tyr	substitution	28	Ser	Tyr	0.000	0.005
p.Ser28Val	substitution	28	Ser	Val	0.000	0.054
p.Ser290=	synonymous	290	Ser	NA	0.000	0.098
p.Ser290Ala	substitution	290	Ser	Ala	0.000	0.006
p.Ser290Arg	substitution	290	Ser	Arg	0.572	0.054
p.Ser290Asn	substitution	290	Ser	Asn	0.704	0.104
p.Ser290Asp	substitution	290	Ser	Asp	0.478	0.006
p.Ser290Cys	substitution	290	Ser	Cys	0.184	0.028
p.Ser290Gln	substitution	290	Ser	Gln	0.000	0.088

p.Ser290Glu	substitution	290	Ser	Glu	2.060	0.067
p.Ser290Gly	substitution	290	Ser	Gly	0.930	0.013
p.Ser290His	substitution	290	Ser	His	0.000	0.036
p.Ser290Ile	substitution	290	Ser	Ile	1.267	0.049
p.Ser290Leu	substitution	290	Ser	Leu	0.661	0.091
p.Ser290Lys	substitution	290	Ser	Lys	0.528	0.006
p.Ser290Met	substitution	290	Ser	Met	1.260	0.004
p.Ser290Phe	substitution	290	Ser	Phe	1.014	0.077
p.Ser290Pro	substitution	290	Ser	Pro	0.361	0.196
p.Ser290Ter	nonsense	290	Ser	Ter	0.000	0.008
p.Ser290Thr	substitution	290	Ser	Thr	0.000	0.070
p.Ser290Trp	substitution	290	Ser	Trp	1.375	0.004
p.Ser290Tyr	substitution	290	Ser	Tyr	0.971	0.005
p.Ser290Val	substitution	290	Ser	Val	0.603	0.003
p.Ser337=	synonymous	337	Ser	NA	1.184	0.011
p.Ser337Arg	substitution	337	Ser	Arg	1.074	0.076
p.Ser337Asn	substitution	337	Ser	Asn	1.222	0.006
p.Ser337Cys	substitution	337	Ser	Cys	0.869	0.188
p.Ser337Gln	substitution	337	Ser	Gln	0.000	0.205
p.Ser337Glu	substitution	337	Ser	Glu	0.369	0.012
p.Ser337Gly	substitution	337	Ser	Gly	0.906	0.084
p.Ser337Ile	substitution	337	Ser	Ile	0.831	0.022
p.Ser337Leu	substitution	337	Ser	Leu	1.236	0.003
p.Ser337Lys	substitution	337	Ser	Lys	0.370	0.092
p.Ser337Met	substitution	337	Ser	Met	0.318	0.029
p.Ser337Phe	substitution	337	Ser	Phe	0.000	0.098
p.Ser337Pro	substitution	337	Ser	Pro	0.005	0.015
p.Ser337Ter	nonsense	337	Ser	Ter	0.282	0.214
p.Ser337Thr	substitution	337	Ser	Thr	1.191	0.047
p.Ser337Trp	substitution	337	Ser	Trp	0.000	0.069
p.Ser337Val	substitution	337	Ser	Val	1.378	0.006
p.Ser344=	synonymous	344	Ser	NA	0.425	0.049
p.Ser344Ala	substitution	344	Ser	Ala	0.000	0.079
p.Ser344Arg	substitution	344	Ser	Arg	1.290	0.019
p.Ser344Asn	substitution	344	Ser	Asn	1.151	0.134
p.Ser344Asp	substitution	344	Ser	Asp	1.756	0.024
p.Ser344Cys	substitution	344	Ser	Cys	0.719	0.045
p.Ser344Gln	substitution	344	Ser	Gln	0.474	0.069
p.Ser344Glu	substitution	344	Ser	Glu	1.072	0.003
p.Ser344Gly	substitution	344	Ser	Gly	1.184	0.022
p.Ser344His	substitution	344	Ser	His	1.327	0.007
p.Ser344Ile	substitution	344	Ser	Ile	1.188	0.004
p.Ser344Leu	substitution	344	Ser	Leu	0.000	0.150

p.Ser344Met	substitution	344	Ser	Met	0.364	0.008
p.Ser344Pro	substitution	344	Ser	Pro	1.125	0.137
p.Ser344Ter	nonsense	344	Ser	Ter	0.000	0.138
p.Ser344Thr	substitution	344	Ser	Thr	1.007	0.095
p.Ser344Trp	substitution	344	Ser	Trp	0.000	0.128
p.Ser344Tyr	substitution	344	Ser	Tyr	0.608	0.073
p.Ser344Val	substitution	344	Ser	Val	0.491	0.007
p.Ser37=	synonymous	37	Ser	NA	0.957	0.011
p.Ser37Ala	substitution	37	Ser	Ala	1.104	0.032
p.Ser37Arg	substitution	37	Ser	Arg	0.865	0.027
p.Ser37Asn	substitution	37	Ser	Asn	1.127	0.007
p.Ser37Asp	substitution	37	Ser	Asp	0.304	0.015
p.Ser37Cys	substitution	37	Ser	Cys	0.908	0.004
p.Ser37Glu	substitution	37	Ser	Glu	0.828	0.016
p.Ser37Gly	substitution	37	Ser	Gly	0.664	0.009
p.Ser37His	substitution	37	Ser	His	0.960	0.009
p.Ser37Ile	substitution	37	Ser	Ile	0.982	0.004
p.Ser37Leu	substitution	37	Ser	Leu	0.914	0.025
p.Ser37Lys	substitution	37	Ser	Lys	0.999	0.004
p.Ser37Met	substitution	37	Ser	Met	1.148	0.005
p.Ser37Phe	substitution	37	Ser	Phe	0.887	0.003
p.Ser37Pro	substitution	37	Ser	Pro	0.439	0.011
p.Ser37Ter	nonsense	37	Ser	Ter	0.000	0.014
p.Ser37Thr	substitution	37	Ser	Thr	1.155	0.005
p.Ser37Trp	substitution	37	Ser	Trp	1.040	0.005
p.Ser37Tyr	substitution	37	Ser	Tyr	0.338	0.016
p.Ser37Val	substitution	37	Ser	Val	0.660	0.006
p.Ser45=	synonymous	45	Ser	NA	1.081	0.052
p.Ser45Ala	substitution	45	Ser	Ala	0.969	0.004
p.Ser45Arg	substitution	45	Ser	Arg	0.860	0.045
p.Ser45Asp	substitution	45	Ser	Asp	0.922	0.005
p.Ser45Cys	substitution	45	Ser	Cys	1.237	0.003
p.Ser45Gln	substitution	45	Ser	Gln	1.141	0.005
p.Ser45Glu	substitution	45	Ser	Glu	0.812	0.003
p.Ser45Gly	substitution	45	Ser	Gly	0.594	0.004
p.Ser45His	substitution	45	Ser	His	0.657	0.008
p.Ser45Ile	substitution	45	Ser	Ile	1.359	0.005
p.Ser45Leu	substitution	45	Ser	Leu	0.630	0.017
p.Ser45Lys	substitution	45	Ser	Lys	1.275	0.006
p.Ser45Met	substitution	45	Ser	Met	0.473	0.006
p.Ser45Phe	substitution	45	Ser	Phe	0.471	0.005
p.Ser45Pro	substitution	45	Ser	Pro	0.719	0.044
p.Ser45Ter	nonsense	45	Ser	Ter	0.326	0.006

p.Ser45Thr	substitution	45	Ser	Thr	1.056	0.004
p.Ser45Trp	substitution	45	Ser	Trp	0.837	0.004
p.Ser45Tyr	substitution	45	Ser	Tyr	1.015	0.007
p.Ser45Val	substitution	45	Ser	Val	0.746	0.028
p.Ser57=	synonymous	57	Ser	NA	1.105	0.010
p.Ser57Ala	substitution	57	Ser	Ala	0.403	0.061
p.Ser57Arg	substitution	57	Ser	Arg	0.868	0.036
p.Ser57Asn	substitution	57	Ser	Asn	0.325	0.683
p.Ser57Asp	substitution	57	Ser	Asp	1.213	0.023
p.Ser57Cys	substitution	57	Ser	Cys	0.790	0.008
p.Ser57Glu	substitution	57	Ser	Glu	0.383	0.025
p.Ser57Gly	substitution	57	Ser	Gly	0.711	0.014
p.Ser57His	substitution	57	Ser	His	0.150	0.044
p.Ser57Ile	substitution	57	Ser	Ile	0.000	0.394
p.Ser57Leu	substitution	57	Ser	Leu	0.752	0.170
p.Ser57Lys	substitution	57	Ser	Lys	0.000	0.044
p.Ser57Met	substitution	57	Ser	Met	0.000	0.162
p.Ser57Phe	substitution	57	Ser	Phe	1.037	0.164
p.Ser57Pro	substitution	57	Ser	Pro	0.408	0.287
p.Ser57Ter	nonsense	57	Ser	Ter	0.096	0.043
p.Ser57Thr	substitution	57	Ser	Thr	0.586	0.408
p.Ser57Trp	substitution	57	Ser	Trp	0.777	0.012
p.Ser57Tyr	substitution	57	Ser	Tyr	0.820	0.052
p.Ser57Val	substitution	57	Ser	Val	1.060	0.009
p.Ser69=	synonymous	69	Ser	NA	0.849	0.024
p.Ser69Ala	substitution	69	Ser	Ala	0.666	0.010
p.Ser69Arg	substitution	69	Ser	Arg	0.889	0.004
p.Ser69Asn	substitution	69	Ser	Asn	0.950	0.006
p.Ser69Asp	substitution	69	Ser	Asp	0.699	0.008
p.Ser69Cys	substitution	69	Ser	Cys	1.030	0.004
p.Ser69Gln	substitution	69	Ser	Gln	0.937	0.018
p.Ser69Glu	substitution	69	Ser	Glu	0.982	0.011
p.Ser69Gly	substitution	69	Ser	Gly	0.797	0.043
p.Ser69His	substitution	69	Ser	His	0.965	0.008
p.Ser69Ile	substitution	69	Ser	Ile	0.879	0.014
p.Ser69Leu	substitution	69	Ser	Leu	0.695	0.003
p.Ser69Lys	substitution	69	Ser	Lys	1.155	0.006
p.Ser69Met	substitution	69	Ser	Met	1.150	0.004
p.Ser69Phe	substitution	69	Ser	Phe	0.688	0.012
p.Ser69Pro	substitution	69	Ser	Pro	0.940	0.009
p.Ser69Ter	nonsense	69	Ser	Ter	0.005	0.014
p.Ser69Thr	substitution	69	Ser	Thr	0.758	0.017
p.Ser69Trp	substitution	69	Ser	Trp	0.742	0.005

p.Ser69Tyr	substitution	69	Ser	Tyr		0.797	0.005
p.Ser69Val	substitution	69	Ser	Val		0.797	0.004
p.Ser75=	synonymous	75	Ser	NA		0.678	0.013
p.Ser75Ala	substitution	75	Ser	Ala		1.045	0.011
p.Ser75Arg	substitution	75	Ser	Arg		0.930	0.046
p.Ser75Asn	substitution	75	Ser	Asn		1.236	0.051
p.Ser75Asp	substitution	75	Ser	Asp		0.460	0.036
p.Ser75Cys	substitution	75	Ser	Cys		1.243	0.033
p.Ser75Gln	substitution	75	Ser	Gln		0.712	0.015
p.Ser75Glu	substitution	75	Ser	Glu		0.279	0.049
p.Ser75Gly	substitution	75	Ser	Gly		0.935	0.036
p.Ser75His	substitution	75	Ser	His		0.630	0.024
p.Ser75Ile	substitution	75	Ser	Ile		0.695	0.029
p.Ser75Leu	substitution	75	Ser	Leu		0.844	0.097
p.Ser75Lys	substitution	75	Ser	Lys		1.561	0.005
p.Ser75Met	substitution	75	Ser	Met		0.470	0.014
p.Ser75Phe	substitution	75	Ser	Phe		0.000	0.387
p.Ser75Pro	substitution	75	Ser	Pro		0.953	0.040
p.Ser75Ter	nonsense	75	Ser	Ter		0.000	0.592
p.Ser75Thr	substitution	75	Ser	Thr		1.205	0.019
p.Ser75Tyr	substitution	75	Ser	Tyr		0.000	0.307
p.Ser75Val	substitution	75	Ser	Val		0.421	0.106
p.Ser96=	synonymous	96	Ser	NA		0.992	0.020
p.Ser96Ala	substitution	96	Ser	Ala		0.985	0.035
p.Ser96Arg	substitution	96	Ser	Arg		0.033	0.059
p.Ser96Asn	substitution	96	Ser	Asn		0.000	0.085
p.Ser96Asp	substitution	96	Ser	Asp		0.005	0.120
p.Ser96Cys	substitution	96	Ser	Cys		0.531	0.032
p.Ser96Gln	substitution	96	Ser	Gln		0.000	0.062
p.Ser96Gly	substitution	96	Ser	Gly		0.791	0.104
p.Ser96His	substitution	96	Ser	His		0.218	0.046
p.Ser96Ile	substitution	96	Ser	Ile		0.000	0.042
p.Ser96Leu	substitution	96	Ser	Leu		0.000	0.078
p.Ser96Lys	substitution	96	Ser	Lys		0.000	0.221
p.Ser96Met	substitution	96	Ser	Met		0.311	0.060
p.Ser96Phe	substitution	96	Ser	Phe		0.000	0.718
p.Ser96Pro	substitution	96	Ser	Pro		0.095	0.072
p.Ser96Ter	nonsense	96	Ser	Ter		0.000	0.032
p.Ser96Thr	substitution	96	Ser	Thr		0.000	0.018
p.Ser96Trp	substitution	96	Ser	Trp		0.000	0.582
p.Ser96Tyr	substitution	96	Ser	Tyr		0.128	0.052
p.Ser96Val	substitution	96	Ser	Val		0.000	0.190
p.Thr102=	synonymous	102	Thr	NA		0.849	0.034

p.Thr102Ala	substitution	102	Thr	Ala		0.921	0.015
p.Thr102Arg	substitution	102	Thr	Arg		0.154	0.154
p.Thr102Asn	substitution	102	Thr	Asn		0.856	0.007
p.Thr102Asp	substitution	102	Thr	Asp		0.370	0.097
p.Thr102Cys	substitution	102	Thr	Cys		0.133	0.409
p.Thr102Gln	substitution	102	Thr	Gln		0.537	0.718
p.Thr102Gly	substitution	102	Thr	Gly		0.706	0.011
p.Thr102His	substitution	102	Thr	His		1.210	0.008
p.Thr102Ile	substitution	102	Thr	Ile		0.998	0.015
p.Thr102Leu	substitution	102	Thr	Leu		0.449	1.127
p.Thr102Lys	substitution	102	Thr	Lys		0.000	0.359
p.Thr102Met	substitution	102	Thr	Met		0.604	0.024
p.Thr102Phe	substitution	102	Thr	Phe		0.072	0.124
p.Thr102Pro	substitution	102	Thr	Pro		0.819	0.045
p.Thr102Ser	substitution	102	Thr	Ser		1.068	0.015
p.Thr102Ter	nonsense	102	Thr	Ter		0.000	0.890
p.Thr102Trp	substitution	102	Thr	Trp		0.000	0.882
p.Thr102Tyr	substitution	102	Thr	Tyr		1.217	0.013
p.Thr102Val	substitution	102	Thr	Val		0.360	0.055
p.Thr109=	synonymous	109	Thr	NA		1.119	0.003
p.Thr109Ala	substitution	109	Thr	Ala		0.465	0.189
p.Thr109Arg	substitution	109	Thr	Arg		0.000	0.222
p.Thr109Asn	substitution	109	Thr	Asn		0.392	0.108
p.Thr109Asp	substitution	109	Thr	Asp		0.000	0.890
p.Thr109Cys	substitution	109	Thr	Cys		0.983	0.038
p.Thr109Gln	substitution	109	Thr	Gln		0.555	0.075
p.Thr109Glu	substitution	109	Thr	Glu		0.018	0.315
p.Thr109Gly	substitution	109	Thr	Gly		0.000	0.225
p.Thr109His	substitution	109	Thr	His		0.733	0.021
p.Thr109Ile	substitution	109	Thr	Ile		0.519	0.066
p.Thr109Leu	substitution	109	Thr	Leu		0.297	0.230
p.Thr109Lys	substitution	109	Thr	Lys		1.029	0.010
p.Thr109Met	substitution	109	Thr	Met		0.686	0.025
p.Thr109Phe	substitution	109	Thr	Phe		0.213	0.019
p.Thr109Pro	substitution	109	Thr	Pro		0.000	0.148
p.Thr109Ser	substitution	109	Thr	Ser		0.770	0.038
p.Thr109Ter	nonsense	109	Thr	Ter		0.000	0.185
p.Thr109Trp	substitution	109	Thr	Trp		0.492	0.027
p.Thr109Tyr	substitution	109	Thr	Tyr		0.179	0.036
p.Thr109Val	substitution	109	Thr	Val		1.229	0.199
p.Thr133=	synonymous	133	Thr	NA		1.003	0.009
p.Thr133Ala	substitution	133	Thr	Ala		0.582	0.064
p.Thr133Arg	substitution	133	Thr	Arg		1.048	0.022

p.Thr133Asn	substitution	133	Thr	Asn	1.075	0.022
p.Thr133Asp	substitution	133	Thr	Asp	1.027	0.024
p.Thr133Cys	substitution	133	Thr	Cys	0.943	0.022
p.Thr133Gln	substitution	133	Thr	Gln	0.435	0.013
p.Thr133Glu	substitution	133	Thr	Glu	0.938	0.013
p.Thr133Gly	substitution	133	Thr	Gly	0.453	0.141
p.Thr133His	substitution	133	Thr	His	0.502	0.048
p.Thr133Ile	substitution	133	Thr	Ile	0.747	0.017
p.Thr133Leu	substitution	133	Thr	Leu	0.704	0.052
p.Thr133Lys	substitution	133	Thr	Lys	0.719	0.015
p.Thr133Met	substitution	133	Thr	Met	0.957	0.005
p.Thr133Phe	substitution	133	Thr	Phe	0.317	0.030
p.Thr133Pro	substitution	133	Thr	Pro	0.368	0.101
p.Thr133Ser	substitution	133	Thr	Ser	0.782	0.018
p.Thr133Ter	nonsense	133	Thr	Ter	0.000	0.015
p.Thr133Trp	substitution	133	Thr	Trp	0.744	0.011
p.Thr133Tyr	substitution	133	Thr	Tyr	0.671	0.028
p.Thr133Val	substitution	133	Thr	Val	0.670	0.019
p.Thr136=	synonymous	136	Thr	NA	0.865	0.007
p.Thr136Ala	substitution	136	Thr	Ala	0.617	0.094
p.Thr136Arg	substitution	136	Thr	Arg	0.879	0.007
p.Thr136Asn	substitution	136	Thr	Asn	0.932	0.012
p.Thr136Asp	substitution	136	Thr	Asp	0.950	0.025
p.Thr136Cys	substitution	136	Thr	Cys	0.468	0.016
p.Thr136Gln	substitution	136	Thr	Gln	0.806	0.005
p.Thr136Glu	substitution	136	Thr	Glu	0.908	0.003
p.Thr136Gly	substitution	136	Thr	Gly	0.776	0.005
p.Thr136His	substitution	136	Thr	His	0.976	0.015
p.Thr136Ile	substitution	136	Thr	Ile	1.103	0.005
p.Thr136Leu	substitution	136	Thr	Leu	0.741	0.007
p.Thr136Lys	substitution	136	Thr	Lys	1.004	0.013
p.Thr136Met	substitution	136	Thr	Met	1.075	0.004
p.Thr136Phe	substitution	136	Thr	Phe	0.640	0.023
p.Thr136Pro	substitution	136	Thr	Pro	0.263	0.058
p.Thr136Ser	substitution	136	Thr	Ser	1.084	0.036
p.Thr136Ter	nonsense	136	Thr	Ter	0.238	0.010
p.Thr136Trp	substitution	136	Thr	Trp	1.082	0.004
p.Thr136Tyr	substitution	136	Thr	Tyr	0.875	0.015
p.Thr136Val	substitution	136	Thr	Val	0.811	0.022
p.Thr145=	synonymous	145	Thr	NA	0.691	0.018
p.Thr145Ala	substitution	145	Thr	Ala	1.183	0.090
p.Thr145Arg	substitution	145	Thr	Arg	0.301	0.037
p.Thr145Asn	substitution	145	Thr	Asn	0.227	0.075

p.Thr145Asp	substitution	145	Thr	Asp	0.000	0.033
p.Thr145Cys	substitution	145	Thr	Cys	0.275	0.044
p.Thr145Gln	substitution	145	Thr	Gln	0.168	0.015
p.Thr145Glu	substitution	145	Thr	Glu	0.000	0.022
p.Thr145Gly	substitution	145	Thr	Gly	0.483	0.047
p.Thr145His	substitution	145	Thr	His	0.131	0.067
p.Thr145Ile	substitution	145	Thr	Ile	0.214	0.035
p.Thr145Leu	substitution	145	Thr	Leu	0.596	0.019
p.Thr145Lys	substitution	145	Thr	Lys	0.000	0.010
p.Thr145Met	substitution	145	Thr	Met	0.562	0.009
p.Thr145Phe	substitution	145	Thr	Phe	0.805	0.020
p.Thr145Pro	substitution	145	Thr	Pro	0.000	0.080
p.Thr145Ser	substitution	145	Thr	Ser	0.869	0.020
p.Thr145Ter	nonsense	145	Thr	Ter	0.000	0.015
p.Thr145Trp	substitution	145	Thr	Trp	0.413	0.009
p.Thr145Tyr	substitution	145	Thr	Tyr	0.000	0.086
p.Thr145Val	substitution	145	Thr	Val	0.807	0.153
p.Thr172=	synonymous	172	Thr	NA	1.017	0.004
p.Thr172Ala	substitution	172	Thr	Ala	1.001	0.017
p.Thr172Arg	substitution	172	Thr	Arg	0.781	0.004
p.Thr172Asn	substitution	172	Thr	Asn	0.960	0.007
p.Thr172Asp	substitution	172	Thr	Asp	0.000	0.031
p.Thr172Cys	substitution	172	Thr	Cys	0.650	0.008
p.Thr172Gln	substitution	172	Thr	Gln	0.000	0.014
p.Thr172Glu	substitution	172	Thr	Glu	0.000	0.010
p.Thr172Gly	substitution	172	Thr	Gly	0.763	0.011
p.Thr172His	substitution	172	Thr	His	0.471	0.012
p.Thr172Ile	substitution	172	Thr	Ile	0.604	0.005
p.Thr172Leu	substitution	172	Thr	Leu	0.845	0.009
p.Thr172Lys	substitution	172	Thr	Lys	0.691	0.014
p.Thr172Met	substitution	172	Thr	Met	0.326	0.011
p.Thr172Phe	substitution	172	Thr	Phe	0.772	0.012
p.Thr172Pro	substitution	172	Thr	Pro	0.168	0.020
p.Thr172Ser	substitution	172	Thr	Ser	0.774	0.010
p.Thr172Ter	nonsense	172	Thr	Ter	0.000	0.015
p.Thr172Trp	substitution	172	Thr	Trp	0.201	0.006
p.Thr172Tyr	substitution	172	Thr	Tyr	0.766	0.011
p.Thr172Val	substitution	172	Thr	Val	0.444	0.063
p.Thr190=	synonymous	190	Thr	NA	1.090	0.014
p.Thr190Ala	substitution	190	Thr	Ala	1.024	0.037
p.Thr190Arg	substitution	190	Thr	Arg	0.560	0.072
p.Thr190Asn	substitution	190	Thr	Asn	1.316	0.002
p.Thr190Asp	substitution	190	Thr	Asp	0.699	0.007

p.Thr190Cys	substitution	190	Thr	Cys		1.129	0.012
p.Thr190Gln	substitution	190	Thr	Gln		1.597	0.101
p.Thr190Glu	substitution	190	Thr	Glu		0.083	0.049
p.Thr190Gly	substitution	190	Thr	Gly		0.879	0.007
p.Thr190His	substitution	190	Thr	His		1.215	0.003
p.Thr190Ile	substitution	190	Thr	Ile		1.243	0.009
p.Thr190Leu	substitution	190	Thr	Leu		1.003	0.018
p.Thr190Lys	substitution	190	Thr	Lys		0.839	0.005
p.Thr190Met	substitution	190	Thr	Met		0.033	0.013
p.Thr190Phe	substitution	190	Thr	Phe		0.000	0.171
p.Thr190Pro	substitution	190	Thr	Pro		0.173	0.051
p.Thr190Ser	substitution	190	Thr	Ser		0.149	0.173
p.Thr190Ter	nonsense	190	Thr	Ter		0.000	0.109
p.Thr190Trp	substitution	190	Thr	Trp		1.093	0.015
p.Thr190Tyr	substitution	190	Thr	Tyr		0.513	0.004
p.Thr190Val	substitution	190	Thr	Val		0.896	0.004
p.Thr243=	synonymous	243	Thr	NA		1.235	0.040
p.Thr243Ala	substitution	243	Thr	Ala		0.000	0.180
p.Thr243Arg	substitution	243	Thr	Arg		0.353	0.053
p.Thr243Asn	substitution	243	Thr	Asn		0.799	0.006
p.Thr243Cys	substitution	243	Thr	Cys		0.000	0.057
p.Thr243Gln	substitution	243	Thr	Gln		1.302	0.005
p.Thr243Glu	substitution	243	Thr	Glu		0.312	0.017
p.Thr243Gly	substitution	243	Thr	Gly		0.903	0.003
p.Thr243His	substitution	243	Thr	His		0.847	0.004
p.Thr243Ile	substitution	243	Thr	Ile		0.625	0.077
p.Thr243Leu	substitution	243	Thr	Leu		0.000	0.048
p.Thr243Lys	substitution	243	Thr	Lys		0.464	0.129
p.Thr243Met	substitution	243	Thr	Met		0.000	0.119
p.Thr243Phe	substitution	243	Thr	Phe		0.000	0.108
p.Thr243Pro	substitution	243	Thr	Pro		0.337	0.030
p.Thr243Ser	substitution	243	Thr	Ser		0.862	0.082
p.Thr243Ter	nonsense	243	Thr	Ter		0.000	0.167
p.Thr243Tyr	substitution	243	Thr	Tyr		0.000	0.036
p.Thr243Val	substitution	243	Thr	Val		0.000	0.051
p.Thr25=	synonymous	25	Thr	NA		0.859	0.003
p.Thr25Ala	substitution	25	Thr	Ala		0.857	0.006
p.Thr25Arg	substitution	25	Thr	Arg		0.678	0.022
p.Thr25Asn	substitution	25	Thr	Asn		0.385	0.005
p.Thr25Asp	substitution	25	Thr	Asp		0.000	0.004
p.Thr25Cys	substitution	25	Thr	Cys		0.465	0.001
p.Thr25Gln	substitution	25	Thr	Gln		0.032	0.003
p.Thr25Glu	substitution	25	Thr	Glu		0.746	0.001

p.Thr25Gly	substitution	25	Thr	Gly	0.304	0.005
p.Thr25His	substitution	25	Thr	His	0.843	0.001
p.Thr25Ile	substitution	25	Thr	Ile	0.340	0.038
p.Thr25Leu	substitution	25	Thr	Leu	0.000	0.048
p.Thr25Lys	substitution	25	Thr	Lys	0.320	0.001
p.Thr25Met	substitution	25	Thr	Met	0.566	0.000
p.Thr25Phe	substitution	25	Thr	Phe	0.236	0.001
p.Thr25Pro	substitution	25	Thr	Pro	0.000	0.002
p.Thr25Ser	substitution	25	Thr	Ser	0.881	0.001
p.Thr25Ter	nonsense	25	Thr	Ter	0.000	0.061
p.Thr25Trp	substitution	25	Thr	Trp	0.000	0.005
p.Thr25Tyr	substitution	25	Thr	Tyr	0.000	0.002
p.Thr25Val	substitution	25	Thr	Val	0.381	0.001
p.Thr269=	synonymous	269	Thr	NA	1.124	0.028
p.Thr269Ala	substitution	269	Thr	Ala	0.725	0.005
p.Thr269Arg	substitution	269	Thr	Arg	0.000	0.078
p.Thr269Asn	substitution	269	Thr	Asn	0.454	0.007
p.Thr269Cys	substitution	269	Thr	Cys	1.248	0.005
p.Thr269Gln	substitution	269	Thr	Gln	0.000	0.040
p.Thr269Glu	substitution	269	Thr	Glu	0.000	0.027
p.Thr269Gly	substitution	269	Thr	Gly	0.000	0.016
p.Thr269Ile	substitution	269	Thr	Ile	0.000	0.176
p.Thr269Leu	substitution	269	Thr	Leu	0.022	0.021
p.Thr269Lys	substitution	269	Thr	Lys	0.000	0.209
p.Thr269Met	substitution	269	Thr	Met	0.261	0.006
p.Thr269Phe	substitution	269	Thr	Phe	0.000	0.015
p.Thr269Pro	substitution	269	Thr	Pro	0.000	0.146
p.Thr269Ser	substitution	269	Thr	Ser	0.094	0.022
p.Thr269Ter	nonsense	269	Thr	Ter	0.034	0.033
p.Thr269Tyr	substitution	269	Thr	Tyr	0.000	0.019
p.Thr269Val	substitution	269	Thr	Val	0.000	0.027
p.Thr279=	synonymous	279	Thr	NA	1.101	0.028
p.Thr279Ala	substitution	279	Thr	Ala	0.998	0.033
p.Thr279Arg	substitution	279	Thr	Arg	0.392	0.006
p.Thr279Asn	substitution	279	Thr	Asn	1.075	0.005
p.Thr279Asp	substitution	279	Thr	Asp	0.208	0.006
p.Thr279Cys	substitution	279	Thr	Cys	0.000	0.006
p.Thr279Gln	substitution	279	Thr	Gln	0.691	0.005
p.Thr279Glu	substitution	279	Thr	Glu	0.587	0.008
p.Thr279Gly	substitution	279	Thr	Gly	0.767	0.004
p.Thr279His	substitution	279	Thr	His	0.337	0.005
p.Thr279Ile	substitution	279	Thr	Ile	1.016	0.006
p.Thr279Leu	substitution	279	Thr	Leu	0.401	0.007

p.Thr279Lys	substitution	279	Thr	Lys	0.187	0.008
p.Thr279Met	substitution	279	Thr	Met	0.269	0.004
p.Thr279Phe	substitution	279	Thr	Phe	0.749	0.004
p.Thr279Pro	substitution	279	Thr	Pro	0.079	0.019
p.Thr279Ser	substitution	279	Thr	Ser	0.824	0.010
p.Thr279Ter	nonsense	279	Thr	Ter	0.000	0.006
p.Thr279Trp	substitution	279	Thr	Trp	0.237	0.014
p.Thr279Tyr	substitution	279	Thr	Tyr	0.916	0.004
p.Thr279Val	substitution	279	Thr	Val	0.602	0.008
p.Thr294=	synonymous	294	Thr	NA	0.631	0.066
p.Thr294Ala	substitution	294	Thr	Ala	0.545	0.141
p.Thr294Arg	substitution	294	Thr	Arg	0.786	0.005
p.Thr294Asn	substitution	294	Thr	Asn	1.146	0.015
p.Thr294Asp	substitution	294	Thr	Asp	0.000	0.110
p.Thr294Cys	substitution	294	Thr	Cys	0.000	0.121
p.Thr294Gln	substitution	294	Thr	Gln	0.000	0.109
p.Thr294Glu	substitution	294	Thr	Glu	0.000	0.075
p.Thr294Gly	substitution	294	Thr	Gly	1.155	0.007
p.Thr294His	substitution	294	Thr	His	1.547	0.011
p.Thr294Ile	substitution	294	Thr	Ile	1.073	0.004
p.Thr294Leu	substitution	294	Thr	Leu	0.703	0.067
p.Thr294Lys	substitution	294	Thr	Lys	0.011	0.003
p.Thr294Phe	substitution	294	Thr	Phe	0.752	0.019
p.Thr294Pro	substitution	294	Thr	Pro	1.429	0.016
p.Thr294Ser	substitution	294	Thr	Ser	1.060	0.029
p.Thr294Ter	nonsense	294	Thr	Ter	0.000	0.059
p.Thr294Tyr	substitution	294	Thr	Tyr	0.000	0.202
p.Thr294Val	substitution	294	Thr	Val	0.951	0.245
p.Thr298=	synonymous	298	Thr	NA	1.086	0.047
p.Thr298Ala	substitution	298	Thr	Ala	0.676	0.155
p.Thr298Arg	substitution	298	Thr	Arg	1.073	0.036
p.Thr298Asn	substitution	298	Thr	Asn	0.590	0.039
p.Thr298Cys	substitution	298	Thr	Cys	0.607	0.009
p.Thr298Gln	substitution	298	Thr	Gln	0.620	0.019
p.Thr298Glu	substitution	298	Thr	Glu	0.824	0.012
p.Thr298Gly	substitution	298	Thr	Gly	0.047	0.052
p.Thr298His	substitution	298	Thr	His	0.670	0.006
p.Thr298Ile	substitution	298	Thr	Ile	0.193	0.136
p.Thr298Leu	substitution	298	Thr	Leu	0.504	0.100
p.Thr298Lys	substitution	298	Thr	Lys	0.720	0.012
p.Thr298Met	substitution	298	Thr	Met	0.754	0.004
p.Thr298Phe	substitution	298	Thr	Phe	0.480	0.057
p.Thr298Pro	substitution	298	Thr	Pro	0.312	0.107

p.Thr298Ser	substitution	298	Thr	Ser		1.054	0.051
p.Thr298Ter	nonsense	298	Thr	Ter		0.000	0.101
p.Thr298Trp	substitution	298	Thr	Trp		0.971	0.005
p.Thr298Tyr	substitution	298	Thr	Tyr		0.781	0.014
p.Thr298Val	substitution	298	Thr	Val		1.011	0.035
p.Thr319=	synonymous	319	Thr	NA		0.749	0.047
p.Thr319Ala	substitution	319	Thr	Ala		0.663	0.031
p.Thr319Arg	substitution	319	Thr	Arg		0.000	0.010
p.Thr319Asn	substitution	319	Thr	Asn		0.353	0.006
p.Thr319Asp	substitution	319	Thr	Asp		0.542	0.006
p.Thr319Cys	substitution	319	Thr	Cys		0.917	0.009
p.Thr319Gln	substitution	319	Thr	Gln		0.000	0.102
p.Thr319Glu	substitution	319	Thr	Glu		0.174	0.017
p.Thr319Gly	substitution	319	Thr	Gly		0.520	0.080
p.Thr319His	substitution	319	Thr	His		1.321	0.003
p.Thr319Ile	substitution	319	Thr	Ile		0.736	0.004
p.Thr319Leu	substitution	319	Thr	Leu		0.000	0.027
p.Thr319Lys	substitution	319	Thr	Lys		0.243	0.014
p.Thr319Met	substitution	319	Thr	Met		0.000	0.019
p.Thr319Phe	substitution	319	Thr	Phe		0.873	0.004
p.Thr319Pro	substitution	319	Thr	Pro		0.245	0.031
p.Thr319Ser	substitution	319	Thr	Ser		0.870	0.062
p.Thr319Ter	nonsense	319	Thr	Ter		0.000	0.203
p.Thr319Trp	substitution	319	Thr	Trp		0.000	0.016
p.Thr319Tyr	substitution	319	Thr	Tyr		1.194	0.007
p.Thr319Val	substitution	319	Thr	Val		0.000	0.021
p.Thr35=	synonymous	35	Thr	NA		0.970	0.002
p.Thr35Ala	substitution	35	Thr	Ala		0.861	0.000
p.Thr35Arg	substitution	35	Thr	Arg		0.000	0.001
p.Thr35Asn	substitution	35	Thr	Asn		0.201	0.001
p.Thr35Asp	substitution	35	Thr	Asp		0.000	0.003
p.Thr35Cys	substitution	35	Thr	Cys		0.624	0.000
p.Thr35Gln	substitution	35	Thr	Gln		0.000	0.001
p.Thr35Glu	substitution	35	Thr	Glu		0.000	0.002
p.Thr35Gly	substitution	35	Thr	Gly		0.838	0.001
p.Thr35His	substitution	35	Thr	His		0.000	0.005
p.Thr35Ile	substitution	35	Thr	Ile		0.000	0.001
p.Thr35Leu	substitution	35	Thr	Leu		0.456	0.008
p.Thr35Lys	substitution	35	Thr	Lys		0.000	0.021
p.Thr35Met	substitution	35	Thr	Met		0.800	0.000
p.Thr35Phe	substitution	35	Thr	Phe		0.000	0.002
p.Thr35Pro	substitution	35	Thr	Pro		0.000	0.003
p.Thr35Ser	substitution	35	Thr	Ser		0.753	0.017

p.Thr35Ter	nonsense	35	Thr	Ter	0.000	0.002
p.Thr35Trp	substitution	35	Thr	Trp	0.236	0.001
p.Thr35Tyr	substitution	35	Thr	Tyr	0.000	0.002
p.Thr35Val	substitution	35	Thr	Val	0.762	0.002
p.Thr41=	synonymous	41	Thr	NA	0.891	0.024
p.Thr41Ala	substitution	41	Thr	Ala	0.842	0.019
p.Thr41Arg	substitution	41	Thr	Arg	0.900	0.021
p.Thr41Asn	substitution	41	Thr	Asn	0.741	0.004
p.Thr41Asp	substitution	41	Thr	Asp	0.779	0.006
p.Thr41Cys	substitution	41	Thr	Cys	0.815	0.007
p.Thr41Gln	substitution	41	Thr	Gln	0.593	0.038
p.Thr41Glu	substitution	41	Thr	Glu	0.885	0.019
p.Thr41Gly	substitution	41	Thr	Gly	0.635	0.015
p.Thr41His	substitution	41	Thr	His	0.933	0.007
p.Thr41Ile	substitution	41	Thr	Ile	0.964	0.056
p.Thr41Leu	substitution	41	Thr	Leu	0.735	0.008
p.Thr41Lys	substitution	41	Thr	Lys	0.736	0.015
p.Thr41Met	substitution	41	Thr	Met	1.147	0.004
p.Thr41Phe	substitution	41	Thr	Phe	0.658	0.004
p.Thr41Pro	substitution	41	Thr	Pro	0.434	0.022
p.Thr41Ser	substitution	41	Thr	Ser	0.993	0.005
p.Thr41Ter	nonsense	41	Thr	Ter	0.294	0.142
p.Thr41Trp	substitution	41	Thr	Trp	0.669	0.007
p.Thr41Tyr	substitution	41	Thr	Tyr	0.834	0.005
p.Thr41Val	substitution	41	Thr	Val	0.364	0.035
p.Thr58=	synonymous	58	Thr	NA	1.073	0.022
p.Thr58Ala	substitution	58	Thr	Ala	0.695	0.178
p.Thr58Arg	substitution	58	Thr	Arg	0.000	0.019
p.Thr58Asn	substitution	58	Thr	Asn	0.903	0.022
p.Thr58Asp	substitution	58	Thr	Asp	0.124	0.263
p.Thr58Cys	substitution	58	Thr	Cys	0.974	0.016
p.Thr58Gln	substitution	58	Thr	Gln	0.142	0.052
p.Thr58Glu	substitution	58	Thr	Glu	0.818	0.012
p.Thr58Gly	substitution	58	Thr	Gly	0.000	0.343
p.Thr58His	substitution	58	Thr	His	0.000	0.180
p.Thr58Ile	substitution	58	Thr	Ile	0.338	0.124
p.Thr58Leu	substitution	58	Thr	Leu	0.253	0.038
p.Thr58Lys	substitution	58	Thr	Lys	0.000	0.052
p.Thr58Met	substitution	58	Thr	Met	0.900	0.012
p.Thr58Phe	substitution	58	Thr	Phe	0.000	0.141
p.Thr58Pro	substitution	58	Thr	Pro	0.053	0.067
p.Thr58Ser	substitution	58	Thr	Ser	0.821	0.099
p.Thr58Ter	nonsense	58	Thr	Ter	0.000	0.123

p.Thr58Trp	substitution	58	Thr	Trp	0.000	0.301
p.Thr58Tyr	substitution	58	Thr	Tyr	0.000	0.096
p.Thr58Val	substitution	58	Thr	Val	0.642	0.025
p.Thr59=	synonymous	59	Thr	NA	1.098	0.072
p.Thr59Ala	substitution	59	Thr	Ala	0.827	0.084
p.Thr59Arg	substitution	59	Thr	Arg	0.619	0.044
p.Thr59Asn	substitution	59	Thr	Asn	0.398	0.023
p.Thr59Asp	substitution	59	Thr	Asp	1.106	0.024
p.Thr59Gln	substitution	59	Thr	Gln	1.014	0.032
p.Thr59Gly	substitution	59	Thr	Gly	0.549	0.065
p.Thr59His	substitution	59	Thr	His	0.825	0.017
p.Thr59Ile	substitution	59	Thr	Ile	0.398	0.144
p.Thr59Leu	substitution	59	Thr	Leu	0.486	0.488
p.Thr59Lys	substitution	59	Thr	Lys	0.599	0.122
p.Thr59Met	substitution	59	Thr	Met	0.986	0.014
p.Thr59Phe	substitution	59	Thr	Phe	1.305	0.019
p.Thr59Pro	substitution	59	Thr	Pro	0.926	0.175
p.Thr59Ser	substitution	59	Thr	Ser	1.118	0.017
p.Thr59Ter	nonsense	59	Thr	Ter	0.307	0.783
p.Thr59Trp	substitution	59	Thr	Trp	0.110	0.034
p.Thr59Val	substitution	59	Thr	Val	0.586	0.061
p.Thr66=	synonymous	66	Thr	NA	0.967	0.175
p.Thr66Ala	substitution	66	Thr	Ala	1.095	0.018
p.Thr66Arg	substitution	66	Thr	Arg	0.761	0.006
p.Thr66Asn	substitution	66	Thr	Asn	0.807	0.025
p.Thr66Asp	substitution	66	Thr	Asp	0.261	0.024
p.Thr66Gln	substitution	66	Thr	Gln	0.463	0.026
p.Thr66Glu	substitution	66	Thr	Glu	1.107	0.011
p.Thr66Gly	substitution	66	Thr	Gly	0.913	0.020
p.Thr66His	substitution	66	Thr	His	0.658	0.012
p.Thr66Ile	substitution	66	Thr	Ile	1.134	0.007
p.Thr66Leu	substitution	66	Thr	Leu	0.759	0.024
p.Thr66Lys	substitution	66	Thr	Lys	0.000	0.030
p.Thr66Met	substitution	66	Thr	Met	0.838	0.007
p.Thr66Phe	substitution	66	Thr	Phe	0.731	0.008
p.Thr66Pro	substitution	66	Thr	Pro	0.962	0.051
p.Thr66Ser	substitution	66	Thr	Ser	1.057	0.014
p.Thr66Ter	nonsense	66	Thr	Ter	0.218	0.015
p.Thr66Trp	substitution	66	Thr	Trp	0.688	0.014
p.Thr66Tyr	substitution	66	Thr	Tyr	0.395	0.014
p.Thr66Val	substitution	66	Thr	Val	1.189	0.011
p.Thr78=	synonymous	78	Thr	NA	0.996	0.014
p.Thr78Ala	substitution	78	Thr	Ala	0.505	0.004

p.Thr78Arg	substitution	78	Thr	Arg	0.000	0.012
p.Thr78Asn	substitution	78	Thr	Asn	0.780	0.033
p.Thr78Asp	substitution	78	Thr	Asp	0.376	0.017
p.Thr78Cys	substitution	78	Thr	Cys	1.101	0.014
p.Thr78Gln	substitution	78	Thr	Gln	0.764	0.019
p.Thr78Glu	substitution	78	Thr	Glu	0.000	0.026
p.Thr78Gly	substitution	78	Thr	Gly	0.901	0.005
p.Thr78His	substitution	78	Thr	His	0.263	0.056
p.Thr78Ile	substitution	78	Thr	Ile	1.176	0.007
p.Thr78Leu	substitution	78	Thr	Leu	0.778	0.097
p.Thr78Lys	substitution	78	Thr	Lys	0.961	0.014
p.Thr78Met	substitution	78	Thr	Met	0.141	0.026
p.Thr78Phe	substitution	78	Thr	Phe	0.000	0.093
p.Thr78Pro	substitution	78	Thr	Pro	0.000	0.036
p.Thr78Ser	substitution	78	Thr	Ser	0.635	0.006
p.Thr78Ter	nonsense	78	Thr	Ter	0.000	0.025
p.Thr78Trp	substitution	78	Thr	Trp	0.117	0.145
p.Thr78Tyr	substitution	78	Thr	Tyr	0.403	0.026
p.Thr78Val	substitution	78	Thr	Val	0.798	0.010
p.Trp198Ala	substitution	198	Trp	Ala	1.033	0.003
p.Trp198Arg	substitution	198	Trp	Arg	0.677	0.010
p.Trp198Asn	substitution	198	Trp	Asn	0.989	0.001
p.Trp198Asp	substitution	198	Trp	Asp	0.688	0.002
p.Trp198Cys	substitution	198	Trp	Cys	1.038	0.013
p.Trp198Gln	substitution	198	Trp	Gln	0.485	0.003
p.Trp198Glu	substitution	198	Trp	Glu	0.982	0.001
p.Trp198Gly	substitution	198	Trp	Gly	0.653	0.018
p.Trp198His	substitution	198	Trp	His	0.851	0.003
p.Trp198Ile	substitution	198	Trp	Ile	0.579	0.023
p.Trp198Leu	substitution	198	Trp	Leu	0.963	0.004
p.Trp198Lys	substitution	198	Trp	Lys	0.887	0.002
p.Trp198Met	substitution	198	Trp	Met	1.224	0.001
p.Trp198Phe	substitution	198	Trp	Phe	0.882	0.002
p.Trp198Pro	substitution	198	Trp	Pro	0.422	0.067
p.Trp198Ser	substitution	198	Trp	Ser	0.785	0.038
p.Trp198Ter	nonsense	198	Trp	Ter	0.000	0.080
p.Trp198Thr	substitution	198	Trp	Thr	0.371	0.052
p.Trp198Tyr	substitution	198	Trp	Tyr	0.880	0.001
p.Trp198Val	substitution	198	Trp	Val	0.778	0.004
p.Trp283Ala	substitution	283	Trp	Ala	0.093	0.173
p.Trp283Arg	substitution	283	Trp	Arg	0.568	0.022
p.Trp283Asn	substitution	283	Trp	Asn	0.965	0.005
p.Trp283Cys	substitution	283	Trp	Cys	0.515	0.008

p.Trp283Gln	substitution	283	Trp	Gln	0.873	0.011
p.Trp283Glu	substitution	283	Trp	Glu	0.000	0.025
p.Trp283Gly	substitution	283	Trp	Gly	1.003	0.079
p.Trp283Ile	substitution	283	Trp	Ile	0.348	0.005
p.Trp283Leu	substitution	283	Trp	Leu	0.174	0.133
p.Trp283Lys	substitution	283	Trp	Lys	0.000	0.014
p.Trp283Met	substitution	283	Trp	Met	0.000	0.022
p.Trp283Phe	substitution	283	Trp	Phe	0.000	0.006
p.Trp283Pro	substitution	283	Trp	Pro	0.695	0.122
p.Trp283Ser	substitution	283	Trp	Ser	1.036	0.006
p.Trp283Ter	nonsense	283	Trp	Ter	0.167	0.494
p.Trp283Thr	substitution	283	Trp	Thr	0.360	0.052
p.Trp283Tyr	substitution	283	Trp	Tyr	1.037	0.003
p.Trp283Val	substitution	283	Trp	Val	0.000	0.051
p.Tyr213=	synonymous	213	Tyr	NA	1.185	0.619
p.Tyr213Ala	substitution	213	Tyr	Ala	0.000	0.031
p.Tyr213Arg	substitution	213	Tyr	Arg	0.000	0.024
p.Tyr213Asn	substitution	213	Tyr	Asn	0.819	0.006
p.Tyr213Asp	substitution	213	Tyr	Asp	0.000	0.014
p.Tyr213Cys	substitution	213	Tyr	Cys	0.500	0.018
p.Tyr213Gln	substitution	213	Tyr	Gln	0.418	0.168
p.Tyr213Glu	substitution	213	Tyr	Glu	1.083	0.004
p.Tyr213Gly	substitution	213	Tyr	Gly	0.731	0.034
p.Tyr213His	substitution	213	Tyr	His	1.142	0.006
p.Tyr213Ile	substitution	213	Tyr	Ile	0.000	0.011
p.Tyr213Leu	substitution	213	Tyr	Leu	0.051	0.030
p.Tyr213Lys	substitution	213	Tyr	Lys	0.000	0.013
p.Tyr213Phe	substitution	213	Tyr	Phe	0.202	0.005
p.Tyr213Pro	substitution	213	Tyr	Pro	0.653	0.118
p.Tyr213Ser	substitution	213	Tyr	Ser	0.186	0.037
p.Tyr213Ter	nonsense	213	Tyr	Ter	0.625	0.113
p.Tyr213Thr	substitution	213	Tyr	Thr	0.000	0.006
p.Tyr213Val	substitution	213	Tyr	Val	0.000	0.006
p.Tyr277=	synonymous	277	Tyr	NA	1.076	0.012
p.Tyr277Ala	substitution	277	Tyr	Ala	1.008	0.015
p.Tyr277Arg	substitution	277	Tyr	Arg	0.919	0.077
p.Tyr277Asn	substitution	277	Tyr	Asn	0.794	0.042
p.Tyr277Asp	substitution	277	Tyr	Asp	0.593	0.027
p.Tyr277Cys	substitution	277	Tyr	Cys	0.634	0.006
p.Tyr277Gln	substitution	277	Tyr	Gln	0.000	0.036
p.Tyr277Glu	substitution	277	Tyr	Glu	0.068	0.011
p.Tyr277Gly	substitution	277	Tyr	Gly	0.119	0.031
p.Tyr277His	substitution	277	Tyr	His	1.419	0.726

p.Tyr277Ile	substitution	277	Tyr	Ile	0.011	0.143
p.Tyr277Leu	substitution	277	Tyr	Leu	0.949	0.100
p.Tyr277Lys	substitution	277	Tyr	Lys	0.222	0.007
p.Tyr277Met	substitution	277	Tyr	Met	0.909	0.004
p.Tyr277Phe	substitution	277	Tyr	Phe	1.166	0.416
p.Tyr277Pro	substitution	277	Tyr	Pro	0.000	0.009
p.Tyr277Ser	substitution	277	Tyr	Ser	0.674	0.162
p.Tyr277Ter	nonsense	277	Tyr	Ter	0.110	0.034
p.Tyr277Thr	substitution	277	Tyr	Thr	0.396	0.054
p.Tyr277Trp	substitution	277	Tyr	Trp	0.000	0.024
p.Tyr277Val	substitution	277	Tyr	Val	0.126	0.025
p.Tyr46=	synonymous	46	Tyr	NA	1.031	0.002
p.Tyr46Ala	substitution	46	Tyr	Ala	0.606	0.046
p.Tyr46Arg	substitution	46	Tyr	Arg	0.632	0.021
p.Tyr46Asn	substitution	46	Tyr	Asn	1.126	0.006
p.Tyr46Asp	substitution	46	Tyr	Asp	0.795	0.012
p.Tyr46Cys	substitution	46	Tyr	Cys	0.754	0.026
p.Tyr46Gly	substitution	46	Tyr	Gly	0.467	0.007
p.Tyr46His	substitution	46	Tyr	His	1.018	0.013
p.Tyr46Ile	substitution	46	Tyr	Ile	0.676	0.026
p.Tyr46Leu	substitution	46	Tyr	Leu	0.605	0.004
p.Tyr46Lys	substitution	46	Tyr	Lys	0.775	0.006
p.Tyr46Met	substitution	46	Tyr	Met	1.024	0.003
p.Tyr46Phe	substitution	46	Tyr	Phe	0.960	0.058
p.Tyr46Pro	substitution	46	Tyr	Pro	0.000	0.057
p.Tyr46Ser	substitution	46	Tyr	Ser	0.700	0.010
p.Tyr46Ter	nonsense	46	Tyr	Ter	0.471	0.024
p.Tyr46Thr	substitution	46	Tyr	Thr	0.784	0.014
p.Tyr46Trp	substitution	46	Tyr	Trp	1.016	0.002
p.Tyr46Val	substitution	46	Tyr	Val	1.094	0.112
p.Val103=	synonymous	103	Val	NA	1.126	0.008
p.Val103Ala	substitution	103	Val	Ala	0.578	0.044
p.Val103Arg	substitution	103	Val	Arg	0.892	0.077
p.Val103Asn	substitution	103	Val	Asn	0.756	0.032
p.Val103Asp	substitution	103	Val	Asp	0.784	0.013
p.Val103Cys	substitution	103	Val	Cys	1.423	0.006
p.Val103Gln	substitution	103	Val	Gln	1.233	0.016
p.Val103Glu	substitution	103	Val	Glu	0.762	0.008
p.Val103Gly	substitution	103	Val	Gly	0.918	0.009
p.Val103His	substitution	103	Val	His	0.000	0.323
p.Val103Ile	substitution	103	Val	Ile	0.659	0.044
p.Val103Leu	substitution	103	Val	Leu	0.635	0.025
p.Val103Lys	substitution	103	Val	Lys	1.016	0.011

p.Val103Met	substitution	103	Val	Met	0.904	0.008
p.Val103Phe	substitution	103	Val	Phe	0.725	0.026
p.Val103Pro	substitution	103	Val	Pro	0.885	0.050
p.Val103Ser	substitution	103	Val	Ser	0.841	0.036
p.Val103Ter	nonsense	103	Val	Ter	0.000	0.023
p.Val103Thr	substitution	103	Val	Thr	1.224	0.279
p.Val103Trp	substitution	103	Val	Trp	1.203	0.016
p.Val103Tyr	substitution	103	Val	Tyr	0.000	0.130
p.Val123=	synonymous	123	Val	NA	1.050	0.018
p.Val123Ala	substitution	123	Val	Ala	0.885	0.007
p.Val123Arg	substitution	123	Val	Arg	0.000	0.217
p.Val123Asn	substitution	123	Val	Asn	0.000	0.145
p.Val123Asp	substitution	123	Val	Asp	0.338	0.342
p.Val123Cys	substitution	123	Val	Cys	0.523	0.016
p.Val123Gln	substitution	123	Val	Gln	0.036	0.038
p.Val123Glu	substitution	123	Val	Glu	0.000	0.019
p.Val123Gly	substitution	123	Val	Gly	0.540	0.015
p.Val123His	substitution	123	Val	His	0.011	0.068
p.Val123Ile	substitution	123	Val	Ile	1.037	0.006
p.Val123Leu	substitution	123	Val	Leu	0.888	0.067
p.Val123Lys	substitution	123	Val	Lys	0.000	0.137
p.Val123Phe	substitution	123	Val	Phe	0.121	0.099
p.Val123Pro	substitution	123	Val	Pro	0.303	0.018
p.Val123Ser	substitution	123	Val	Ser	0.685	0.006
p.Val123Ter	nonsense	123	Val	Ter	0.000	0.039
p.Val123Thr	substitution	123	Val	Thr	1.094	0.008
p.Val123Trp	substitution	123	Val	Trp	0.000	0.113
p.Val123Tyr	substitution	123	Val	Tyr	0.000	0.052
p.Val124=	synonymous	124	Val	NA	1.053	0.004
p.Val124Ala	substitution	124	Val	Ala	0.919	0.008
p.Val124Arg	substitution	124	Val	Arg	0.073	0.011
p.Val124Asn	substitution	124	Val	Asn	0.090	0.017
p.Val124Asp	substitution	124	Val	Asp	0.000	0.023
p.Val124Cys	substitution	124	Val	Cys	0.696	0.010
p.Val124Gln	substitution	124	Val	Gln	0.140	0.030
p.Val124Glu	substitution	124	Val	Glu	0.161	0.006
p.Val124Gly	substitution	124	Val	Gly	0.299	0.028
p.Val124His	substitution	124	Val	His	0.000	0.069
p.Val124Ile	substitution	124	Val	Ile	0.893	0.032
p.Val124Leu	substitution	124	Val	Leu	0.771	0.024
p.Val124Lys	substitution	124	Val	Lys	0.251	0.016
p.Val124Met	substitution	124	Val	Met	1.006	0.004
p.Val124Phe	substitution	124	Val	Phe	0.493	0.008

p.Val124Pro	substitution	124	Val	Pro	0.011	0.010
p.Val124Ser	substitution	124	Val	Ser	0.486	0.019
p.Val124Ter	nonsense	124	Val	Ter	0.000	0.014
p.Val124Thr	substitution	124	Val	Thr	0.401	0.023
p.Val124Trp	substitution	124	Val	Trp	0.273	0.013
p.Val124Tyr	substitution	124	Val	Tyr	0.000	0.045
p.Val130=	synonymous	130	Val	NA	0.861	0.042
p.Val130Ala	substitution	130	Val	Ala	0.906	0.019
p.Val130Arg	substitution	130	Val	Arg	1.159	0.027
p.Val130Asn	substitution	130	Val	Asn	0.976	0.034
p.Val130Asp	substitution	130	Val	Asp	1.432	0.018
p.Val130Cys	substitution	130	Val	Cys	1.136	0.010
p.Val130Gln	substitution	130	Val	Gln	0.855	0.017
p.Val130Glu	substitution	130	Val	Glu	1.632	0.081
p.Val130Gly	substitution	130	Val	Gly	0.543	0.056
p.Val130His	substitution	130	Val	His	1.011	0.019
p.Val130Ile	substitution	130	Val	Ile	1.073	0.009
p.Val130Leu	substitution	130	Val	Leu	0.589	0.113
p.Val130Lys	substitution	130	Val	Lys	0.515	0.103
p.Val130Met	substitution	130	Val	Met	0.719	0.155
p.Val130Phe	substitution	130	Val	Phe	0.668	0.015
p.Val130Pro	substitution	130	Val	Pro	0.944	0.013
p.Val130Ser	substitution	130	Val	Ser	0.199	0.078
p.Val130Ter	nonsense	130	Val	Ter	0.025	0.099
p.Val130Thr	substitution	130	Val	Thr	0.721	0.014
p.Val130Trp	substitution	130	Val	Trp	0.477	0.141
p.Val130Tyr	substitution	130	Val	Tyr	1.110	0.021
p.Val142=	synonymous	142	Val	NA	1.184	0.015
p.Val142Ala	substitution	142	Val	Ala	0.949	0.004
p.Val142Arg	substitution	142	Val	Arg	0.966	0.002
p.Val142Asn	substitution	142	Val	Asn	0.885	0.005
p.Val142Asp	substitution	142	Val	Asp	0.694	0.005
p.Val142Cys	substitution	142	Val	Cys	1.002	0.003
p.Val142Gln	substitution	142	Val	Gln	0.449	0.005
p.Val142Glu	substitution	142	Val	Glu	0.890	0.009
p.Val142Gly	substitution	142	Val	Gly	0.772	0.003
p.Val142His	substitution	142	Val	His	0.809	0.004
p.Val142Leu	substitution	142	Val	Leu	0.825	0.003
p.Val142Lys	substitution	142	Val	Lys	0.829	0.004
p.Val142Met	substitution	142	Val	Met	0.824	0.004
p.Val142Phe	substitution	142	Val	Phe	0.596	0.004
p.Val142Pro	substitution	142	Val	Pro	0.474	0.006
p.Val142Ser	substitution	142	Val	Ser	0.812	0.006

p.Val142Ter	nonsense	142	Val	Ter	0.000	0.016
p.Val142Thr	substitution	142	Val	Thr	0.836	0.004
p.Val142Trp	substitution	142	Val	Trp	0.753	0.004
p.Val142Tyr	substitution	142	Val	Tyr	0.840	0.003
p.Val143=	synonymous	143	Val	NA	1.054	0.005
p.Val143Ala	substitution	143	Val	Ala	1.052	0.006
p.Val143Arg	substitution	143	Val	Arg	0.000	0.005
p.Val143Asn	substitution	143	Val	Asn	0.016	0.009
p.Val143Asp	substitution	143	Val	Asp	0.122	0.015
p.Val143Cys	substitution	143	Val	Cys	1.180	0.004
p.Val143Gln	substitution	143	Val	Gln	0.141	0.012
p.Val143Glu	substitution	143	Val	Glu	0.199	0.009
p.Val143Gly	substitution	143	Val	Gly	0.143	0.005
p.Val143His	substitution	143	Val	His	0.041	0.009
p.Val143Ile	substitution	143	Val	Ile	1.000	0.004
p.Val143Leu	substitution	143	Val	Leu	0.857	0.001
p.Val143Lys	substitution	143	Val	Lys	0.135	0.008
p.Val143Met	substitution	143	Val	Met	0.787	0.004
p.Val143Pro	substitution	143	Val	Pro	0.055	0.006
p.Val143Ser	substitution	143	Val	Ser	0.013	0.006
p.Val143Ter	nonsense	143	Val	Ter	0.000	0.006
p.Val143Thr	substitution	143	Val	Thr	0.379	0.005
p.Val143Trp	substitution	143	Val	Trp	0.006	0.005
p.Val143Tyr	substitution	143	Val	Tyr	0.438	0.002
p.Val202=	synonymous	202	Val	NA	0.956	0.015
p.Val202Ala	substitution	202	Val	Ala	0.796	0.051
p.Val202Arg	substitution	202	Val	Arg	0.724	0.088
p.Val202Asn	substitution	202	Val	Asn	0.000	0.021
p.Val202Asp	substitution	202	Val	Asp	0.764	0.084
p.Val202Cys	substitution	202	Val	Cys	0.956	0.003
p.Val202Gln	substitution	202	Val	Gln	0.433	0.017
p.Val202Glu	substitution	202	Val	Glu	0.841	0.003
p.Val202Gly	substitution	202	Val	Gly	0.776	0.001
p.Val202His	substitution	202	Val	His	0.000	0.022
p.Val202Leu	substitution	202	Val	Leu	0.904	0.018
p.Val202Lys	substitution	202	Val	Lys	0.555	0.023
p.Val202Met	substitution	202	Val	Met	0.972	0.003
p.Val202Phe	substitution	202	Val	Phe	0.000	0.067
p.Val202Pro	substitution	202	Val	Pro	1.366	0.002
p.Val202Ser	substitution	202	Val	Ser	0.466	0.202
p.Val202Ter	nonsense	202	Val	Ter	0.000	0.050
p.Val202Thr	substitution	202	Val	Thr	1.164	0.085
p.Val202Trp	substitution	202	Val	Trp	0.278	0.010

p.Val202Tyr	substitution	202	Val	Tyr	0.637	0.012
p.Val20=	synonymous	20	Val	NA	1.249	0.001
p.Val20Ala	substitution	20	Val	Ala	0.751	0.011
p.Val20Arg	substitution	20	Val	Arg	0.919	0.008
p.Val20Asp	substitution	20	Val	Asp	0.283	0.001
p.Val20Gln	substitution	20	Val	Gln	0.945	0.002
p.Val20Glu	substitution	20	Val	Glu	0.478	0.002
p.Val20Gly	substitution	20	Val	Gly	0.589	0.001
p.Val20Ile	substitution	20	Val	Ile	0.821	0.002
p.Val20Leu	substitution	20	Val	Leu	0.627	0.013
p.Val20Lys	substitution	20	Val	Lys	1.197	0.001
p.Val20Met	substitution	20	Val	Met	0.906	0.002
p.Val20Phe	substitution	20	Val	Phe	0.753	0.001
p.Val20Pro	substitution	20	Val	Pro	0.839	0.001
p.Val20Ser	substitution	20	Val	Ser	1.262	0.001
p.Val20Ter	nonsense	20	Val	Ter	0.000	0.006
p.Val20Thr	substitution	20	Val	Thr	0.967	0.001
p.Val20Trp	substitution	20	Val	Trp	0.653	0.001
p.Val215=	synonymous	215	Val	NA	0.910	0.014
p.Val215Ala	substitution	215	Val	Ala	0.734	0.129
p.Val215Arg	substitution	215	Val	Arg	0.185	0.054
p.Val215Asn	substitution	215	Val	Asn	0.000	0.015
p.Val215Asp	substitution	215	Val	Asp	0.000	0.007
p.Val215Cys	substitution	215	Val	Cys	1.331	0.003
p.Val215Glu	substitution	215	Val	Glu	0.647	0.664
p.Val215Gly	substitution	215	Val	Gly	1.192	0.002
p.Val215Ile	substitution	215	Val	Ile	0.152	0.026
p.Val215Leu	substitution	215	Val	Leu	0.512	0.105
p.Val215Lys	substitution	215	Val	Lys	0.000	0.036
p.Val215Met	substitution	215	Val	Met	0.791	0.025
p.Val215Phe	substitution	215	Val	Phe	0.251	0.159
p.Val215Pro	substitution	215	Val	Pro	0.523	0.005
p.Val215Ser	substitution	215	Val	Ser	1.110	0.215
p.Val215Trp	substitution	215	Val	Trp	0.000	0.068
p.Val215Tyr	substitution	215	Val	Tyr	0.000	0.124
p.Val222=	synonymous	222	Val	NA	0.998	0.003
p.Val222Ala	substitution	222	Val	Ala	0.836	0.027
p.Val222Arg	substitution	222	Val	Arg	0.000	0.012
p.Val222Asn	substitution	222	Val	Asn	0.000	0.034
p.Val222Asp	substitution	222	Val	Asp	0.000	0.051
p.Val222Cys	substitution	222	Val	Cys	0.784	0.002
p.Val222Gln	substitution	222	Val	Gln	0.000	0.008
p.Val222Glu	substitution	222	Val	Glu	0.356	0.326

p.Val222Gly	substitution	222	Val	Gly	0.000	0.004
p.Val222His	substitution	222	Val	His	0.000	0.073
p.Val222Ile	substitution	222	Val	Ile	1.303	0.001
p.Val222Leu	substitution	222	Val	Leu	1.165	0.010
p.Val222Lys	substitution	222	Val	Lys	0.000	0.006
p.Val222Met	substitution	222	Val	Met	0.657	0.006
p.Val222Phe	substitution	222	Val	Phe	0.000	0.010
p.Val222Pro	substitution	222	Val	Pro	0.000	0.035
p.Val222Ser	substitution	222	Val	Ser	0.000	0.010
p.Val222Ter	nonsense	222	Val	Ter	0.000	0.078
p.Val222Thr	substitution	222	Val	Thr	0.000	0.051
p.Val222Trp	substitution	222	Val	Trp	0.000	0.015
p.Val222Tyr	substitution	222	Val	Tyr	0.000	0.006
p.Val224=	synonymous	224	Val	NA	0.982	0.249
p.Val224Ala	substitution	224	Val	Ala	0.598	1.015
p.Val224Arg	substitution	224	Val	Arg	0.046	0.082
p.Val224Asp	substitution	224	Val	Asp	0.000	0.063
p.Val224Cys	substitution	224	Val	Cys	1.094	0.002
p.Val224Glu	substitution	224	Val	Glu	0.296	0.016
p.Val224Gly	substitution	224	Val	Gly	0.304	0.025
p.Val224His	substitution	224	Val	His	0.000	0.129
p.Val224Leu	substitution	224	Val	Leu	1.373	0.007
p.Val224Met	substitution	224	Val	Met	1.272	0.004
p.Val224Phe	substitution	224	Val	Phe	1.091	0.071
p.Val224Pro	substitution	224	Val	Pro	0.250	0.105
p.Val224Ser	substitution	224	Val	Ser	1.456	0.003
p.Val224Ter	nonsense	224	Val	Ter	0.000	0.013
p.Val224Thr	substitution	224	Val	Thr	1.381	0.003
p.Val224Trp	substitution	224	Val	Trp	0.059	0.011
p.Val224Tyr	substitution	224	Val	Tyr	0.121	0.021
p.Val235=	synonymous	235	Val	NA	0.942	0.023
p.Val235Ala	substitution	235	Val	Ala	0.067	0.088
p.Val235Arg	substitution	235	Val	Arg	0.000	0.065
p.Val235Asp	substitution	235	Val	Asp	0.000	0.014
p.Val235Cys	substitution	235	Val	Cys	0.000	0.004
p.Val235Glu	substitution	235	Val	Glu	0.388	0.014
p.Val235Gly	substitution	235	Val	Gly	0.160	0.035
p.Val235His	substitution	235	Val	His	0.000	0.084
p.Val235Ile	substitution	235	Val	Ile	0.013	0.024
p.Val235Leu	substitution	235	Val	Leu	0.790	0.016
p.Val235Lys	substitution	235	Val	Lys	0.000	0.027
p.Val235Met	substitution	235	Val	Met	0.908	0.002
p.Val235Phe	substitution	235	Val	Phe	0.000	0.099

p.Val235Pro	substitution	235	Val	Pro	0.000	0.035
p.Val235Ser	substitution	235	Val	Ser	0.000	0.039
p.Val235Ter	nonsense	235	Val	Ter	0.000	0.070
p.Val235Thr	substitution	235	Val	Thr	0.000	0.027
p.Val235Trp	substitution	235	Val	Trp	0.000	0.007
p.Val235Tyr	substitution	235	Val	Tyr	0.208	0.010
p.Val237=	synonymous	237	Val	NA	0.997	0.030
p.Val237Ala	substitution	237	Val	Ala	0.000	0.167
p.Val237Arg	substitution	237	Val	Arg	0.815	0.015
p.Val237Asp	substitution	237	Val	Asp	0.000	0.049
p.Val237Cys	substitution	237	Val	Cys	0.126	0.074
p.Val237Gln	substitution	237	Val	Gln	0.128	0.036
p.Val237Glu	substitution	237	Val	Glu	0.154	0.030
p.Val237Gly	substitution	237	Val	Gly	0.253	0.030
p.Val237His	substitution	237	Val	His	0.000	0.053
p.Val237Leu	substitution	237	Val	Leu	1.052	0.012
p.Val237Met	substitution	237	Val	Met	0.655	0.039
p.Val237Phe	substitution	237	Val	Phe	0.553	0.081
p.Val237Pro	substitution	237	Val	Pro	1.123	0.002
p.Val237Ser	substitution	237	Val	Ser	0.900	0.065
p.Val237Ter	nonsense	237	Val	Ter	0.180	0.023
p.Val237Trp	substitution	237	Val	Trp	0.000	0.009
p.Val237Tyr	substitution	237	Val	Tyr	0.209	0.024
p.Val23=	synonymous	23	Val	NA	0.967	0.001
p.Val23Ala	substitution	23	Val	Ala	1.271	0.032
p.Val23Arg	substitution	23	Val	Arg	0.000	0.070
p.Val23Asn	substitution	23	Val	Asn	0.000	0.005
p.Val23Asp	substitution	23	Val	Asp	0.000	0.009
p.Val23Cys	substitution	23	Val	Cys	0.367	0.001
p.Val23Glu	substitution	23	Val	Glu	0.000	0.027
p.Val23Gly	substitution	23	Val	Gly	0.000	0.012
p.Val23His	substitution	23	Val	His	0.193	0.011
p.Val23Ile	substitution	23	Val	Ile	0.122	0.003
p.Val23Leu	substitution	23	Val	Leu	0.817	0.001
p.Val23Lys	substitution	23	Val	Lys	0.000	0.005
p.Val23Met	substitution	23	Val	Met	0.949	0.001
p.Val23Phe	substitution	23	Val	Phe	1.032	0.001
p.Val23Pro	substitution	23	Val	Pro	0.000	0.049
p.Val23Ser	substitution	23	Val	Ser	0.000	0.028
p.Val23Ter	nonsense	23	Val	Ter	0.000	0.093
p.Val23Thr	substitution	23	Val	Thr	0.055	0.051
p.Val23Trp	substitution	23	Val	Trp	0.000	0.151
p.Val23Tyr	substitution	23	Val	Tyr	0.237	0.006

p.Val263=	synonymous	263	Val	NA	0.814	0.011
p.Val263Ala	substitution	263	Val	Ala	1.358	0.004
p.Val263Arg	substitution	263	Val	Arg	0.320	0.144
p.Val263Asp	substitution	263	Val	Asp	0.079	0.110
p.Val263Gln	substitution	263	Val	Gln	1.479	0.005
p.Val263Glu	substitution	263	Val	Glu	1.053	0.014
p.Val263Gly	substitution	263	Val	Gly	0.000	0.197
p.Val263His	substitution	263	Val	His	0.073	0.021
p.Val263Leu	substitution	263	Val	Leu	0.948	0.070
p.Val263Lys	substitution	263	Val	Lys	1.412	0.005
p.Val263Met	substitution	263	Val	Met	0.463	0.048
p.Val263Phe	substitution	263	Val	Phe	0.600	0.053
p.Val263Ser	substitution	263	Val	Ser	0.693	0.008
p.Val263Ter	nonsense	263	Val	Ter	0.010	0.019
p.Val263Trp	substitution	263	Val	Trp	0.000	0.021
p.Val265=	synonymous	265	Val	NA	1.039	0.159
p.Val265Ala	substitution	265	Val	Ala	1.214	0.147
p.Val265Arg	substitution	265	Val	Arg	0.000	0.035
p.Val265Asn	substitution	265	Val	Asn	0.000	0.012
p.Val265Asp	substitution	265	Val	Asp	0.000	0.027
p.Val265Gln	substitution	265	Val	Gln	0.000	0.039
p.Val265Gly	substitution	265	Val	Gly	0.213	0.012
p.Val265His	substitution	265	Val	His	0.000	0.083
p.Val265Ile	substitution	265	Val	Ile	0.872	0.170
p.Val265Leu	substitution	265	Val	Leu	0.363	0.130
p.Val265Met	substitution	265	Val	Met	0.103	0.013
p.Val265Phe	substitution	265	Val	Phe	0.000	0.027
p.Val265Pro	substitution	265	Val	Pro	0.315	0.013
p.Val265Ser	substitution	265	Val	Ser	0.000	0.057
p.Val265Ter	nonsense	265	Val	Ter	0.000	0.036
p.Val265Thr	substitution	265	Val	Thr	0.255	0.028
p.Val267=	synonymous	267	Val	NA	1.589	0.036
p.Val267Ala	substitution	267	Val	Ala	0.331	0.074
p.Val267Arg	substitution	267	Val	Arg	0.000	0.067
p.Val267Asn	substitution	267	Val	Asn	1.290	0.028
p.Val267Asp	substitution	267	Val	Asp	0.000	0.140
p.Val267Cys	substitution	267	Val	Cys	1.159	0.007
p.Val267Gln	substitution	267	Val	Gln	0.000	0.094
p.Val267Glu	substitution	267	Val	Glu	0.263	0.367
p.Val267Gly	substitution	267	Val	Gly	0.380	0.014
p.Val267Ile	substitution	267	Val	Ile	0.229	0.077
p.Val267Leu	substitution	267	Val	Leu	0.848	0.021
p.Val267Lys	substitution	267	Val	Lys	0.601	0.028

p.Val267Met	substitution	267	Val	Met	1.328	0.051
p.Val267Phe	substitution	267	Val	Phe	1.108	0.025
p.Val267Pro	substitution	267	Val	Pro	0.000	0.037
p.Val267Ser	substitution	267	Val	Ser	0.277	0.048
p.Val267Ter	nonsense	267	Val	Ter	0.000	0.007
p.Val267Thr	substitution	267	Val	Thr	1.476	0.006
p.Val267Trp	substitution	267	Val	Trp	0.000	0.013
p.Val267Tyr	substitution	267	Val	Tyr	0.000	0.029
p.Val282=	synonymous	282	Val	NA	0.923	0.004
p.Val282Ala	substitution	282	Val	Ala	0.757	0.086
p.Val282Arg	substitution	282	Val	Arg	0.000	0.017
p.Val282Asn	substitution	282	Val	Asn	0.126	0.018
p.Val282Asp	substitution	282	Val	Asp	0.114	0.095
p.Val282Cys	substitution	282	Val	Cys	0.622	0.021
p.Val282Gln	substitution	282	Val	Gln	0.237	0.010
p.Val282Glu	substitution	282	Val	Glu	0.000	0.011
p.Val282Gly	substitution	282	Val	Gly	0.547	0.041
p.Val282His	substitution	282	Val	His	0.249	0.015
p.Val282Ile	substitution	282	Val	Ile	0.401	0.015
p.Val282Leu	substitution	282	Val	Leu	0.463	0.052
p.Val282Lys	substitution	282	Val	Lys	0.177	0.009
p.Val282Met	substitution	282	Val	Met	0.650	0.004
p.Val282Phe	substitution	282	Val	Phe	0.742	0.016
p.Val282Pro	substitution	282	Val	Pro	0.000	0.019
p.Val282Ser	substitution	282	Val	Ser	0.304	0.030
p.Val282Ter	nonsense	282	Val	Ter	0.000	0.055
p.Val282Thr	substitution	282	Val	Thr	0.593	0.125
p.Val282Tyr	substitution	282	Val	Tyr	0.041	0.026
p.Val301=	synonymous	301	Val	NA	0.945	0.046
p.Val301Ala	substitution	301	Val	Ala	0.953	0.046
p.Val301Arg	substitution	301	Val	Arg	0.767	0.094
p.Val301Asn	substitution	301	Val	Asn	0.000	0.023
p.Val301Asp	substitution	301	Val	Asp	0.941	0.011
p.Val301Cys	substitution	301	Val	Cys	0.611	0.010
p.Val301Gly	substitution	301	Val	Gly	0.979	0.080
p.Val301His	substitution	301	Val	His	0.031	0.261
p.Val301Ile	substitution	301	Val	Ile	0.999	0.337
p.Val301Leu	substitution	301	Val	Leu	0.000	0.334
p.Val301Lys	substitution	301	Val	Lys	0.000	0.082
p.Val301Met	substitution	301	Val	Met	0.314	0.062
p.Val301Phe	substitution	301	Val	Phe	1.005	0.010
p.Val301Pro	substitution	301	Val	Pro	0.000	0.026
p.Val301Ser	substitution	301	Val	Ser	0.480	0.129

p.Val301Thr	substitution	301	Val	Thr	0.000	0.034
p.Val301Tyr	substitution	301	Val	Tyr	0.712	0.010
p.Val316=	synonymous	316	Val	NA	0.900	0.029
p.Val316Ala	substitution	316	Val	Ala	0.857	0.011
p.Val316Arg	substitution	316	Val	Arg	0.987	0.184
p.Val316Asn	substitution	316	Val	Asn	0.000	0.029
p.Val316Asp	substitution	316	Val	Asp	0.825	0.006
p.Val316Gln	substitution	316	Val	Gln	0.000	0.069
p.Val316Glu	substitution	316	Val	Glu	0.290	0.199
p.Val316Gly	substitution	316	Val	Gly	0.677	0.034
p.Val316His	substitution	316	Val	His	0.105	0.032
p.Val316Ile	substitution	316	Val	Ile	0.795	0.279
p.Val316Leu	substitution	316	Val	Leu	0.994	0.029
p.Val316Lys	substitution	316	Val	Lys	0.000	0.233
p.Val316Met	substitution	316	Val	Met	0.000	0.129
p.Val316Phe	substitution	316	Val	Phe	1.001	0.006
p.Val316Pro	substitution	316	Val	Pro	0.386	0.026
p.Val316Ser	substitution	316	Val	Ser	0.705	0.178
p.Val316Ter	nonsense	316	Val	Ter	0.030	0.106
p.Val316Thr	substitution	316	Val	Thr	0.064	0.038
p.Val316Tyr	substitution	316	Val	Tyr	0.000	0.007
p.Val353=	synonymous	353	Val	NA	1.205	0.009
p.Val353Ala	substitution	353	Val	Ala	0.712	0.051
p.Val353Arg	substitution	353	Val	Arg	0.476	0.017
p.Val353Asn	substitution	353	Val	Asn	1.252	0.002
p.Val353Asp	substitution	353	Val	Asp	1.389	0.164
p.Val353Cys	substitution	353	Val	Cys	0.000	0.072
p.Val353Gln	substitution	353	Val	Gln	1.385	0.005
p.Val353Gly	substitution	353	Val	Gly	0.555	0.026
p.Val353His	substitution	353	Val	His	1.392	0.007
p.Val353Ile	substitution	353	Val	Ile	0.432	0.017
p.Val353Leu	substitution	353	Val	Leu	0.463	0.040
p.Val353Lys	substitution	353	Val	Lys	0.000	0.109
p.Val353Met	substitution	353	Val	Met	1.197	0.003
p.Val353Phe	substitution	353	Val	Phe	0.000	0.188
p.Val353Pro	substitution	353	Val	Pro	0.613	0.231
p.Val353Ser	substitution	353	Val	Ser	0.782	0.087
p.Val353Ter	nonsense	353	Val	Ter	0.000	0.123
p.Val353Thr	substitution	353	Val	Thr	1.380	0.003
p.Val38=	synonymous	38	Val	NA	0.982	0.027
p.Val38Ala	substitution	38	Val	Ala	0.691	0.035
p.Val38Arg	substitution	38	Val	Arg	0.083	0.038
p.Val38Asn	substitution	38	Val	Asn	0.141	0.032

p.Val38Asp	substitution	38	Val	Asp	0.000	0.018
p.Val38Cys	substitution	38	Val	Cys	0.700	0.009
p.Val38Gln	substitution	38	Val	Gln	0.000	0.054
p.Val38Glu	substitution	38	Val	Glu	0.160	0.120
p.Val38Gly	substitution	38	Val	Gly	0.000	0.012
p.Val38His	substitution	38	Val	His	0.072	0.107
p.Val38Ile	substitution	38	Val	Ile	1.199	0.008
p.Val38Leu	substitution	38	Val	Leu	0.641	0.007
p.Val38Lys	substitution	38	Val	Lys	0.000	0.025
p.Val38Met	substitution	38	Val	Met	0.510	0.030
p.Val38Phe	substitution	38	Val	Phe	0.334	0.011
p.Val38Pro	substitution	38	Val	Pro	0.000	0.097
p.Val38Ser	substitution	38	Val	Ser	0.370	0.065
p.Val38Ter	nonsense	38	Val	Ter	0.000	0.012
p.Val38Thr	substitution	38	Val	Thr	0.970	0.005
p.Val38Trp	substitution	38	Val	Trp	0.000	0.018
p.Val38Tyr	substitution	38	Val	Tyr	0.000	0.078
p.Val39=	synonymous	39	Val	NA	0.874	0.019
p.Val39Ala	substitution	39	Val	Ala	1.000	0.023
p.Val39Arg	substitution	39	Val	Arg	0.637	0.017
p.Val39Asn	substitution	39	Val	Asn	0.681	0.009
p.Val39Asp	substitution	39	Val	Asp	0.000	0.012
p.Val39Cys	substitution	39	Val	Cys	0.816	0.003
p.Val39Gln	substitution	39	Val	Gln	0.603	0.006
p.Val39Glu	substitution	39	Val	Glu	0.569	0.006
p.Val39Gly	substitution	39	Val	Gly	0.537	0.008
p.Val39His	substitution	39	Val	His	0.419	0.007
p.Val39Ile	substitution	39	Val	Ile	0.417	0.013
p.Val39Leu	substitution	39	Val	Leu	0.726	0.003
p.Val39Lys	substitution	39	Val	Lys	0.583	0.007
p.Val39Met	substitution	39	Val	Met	0.573	0.009
p.Val39Phe	substitution	39	Val	Phe	0.517	0.010
p.Val39Pro	substitution	39	Val	Pro	0.000	0.013
p.Val39Ser	substitution	39	Val	Ser	0.614	0.026
p.Val39Ter	nonsense	39	Val	Ter	0.000	0.008
p.Val39Thr	substitution	39	Val	Thr	0.632	0.016
p.Val39Trp	substitution	39	Val	Trp	0.445	0.005
p.Val39Tyr	substitution	39	Val	Tyr	0.802	0.005
p.Val90=	synonymous	90	Val	NA	1.024	0.003
p.Val90Ala	substitution	90	Val	Ala	0.510	0.015
p.Val90Arg	substitution	90	Val	Arg	0.000	0.005
p.Val90Asn	substitution	90	Val	Asn	0.122	0.034
p.Val90Asp	substitution	90	Val	Asp	0.000	0.022

p.Val90Cys	substitution	90	Val	Cys		0.862	0.004
p.Val90Gln	substitution	90	Val	Gln		0.305	0.006
p.Val90Glu	substitution	90	Val	Glu		0.387	0.010
p.Val90Gly	substitution	90	Val	Gly		0.032	0.016
p.Val90His	substitution	90	Val	His		0.375	0.018
p.Val90Ile	substitution	90	Val	Ile		0.863	0.005
p.Val90Leu	substitution	90	Val	Leu		0.990	0.016
p.Val90Lys	substitution	90	Val	Lys		0.000	0.023
p.Val90Met	substitution	90	Val	Met		0.762	0.005
p.Val90Phe	substitution	90	Val	Phe		0.286	0.014
p.Val90Pro	substitution	90	Val	Pro		0.000	0.031
p.Val90Ser	substitution	90	Val	Ser		0.505	0.006
p.Val90Ter	nonsense	90	Val	Ter		0.000	0.013
p.Val90Thr	substitution	90	Val	Thr		0.549	0.005
p.Val90Trp	substitution	90	Val	Trp		0.199	0.006
p.Val90Tyr	substitution	90	Val	Tyr		0.346	0.019
p.Val93=	synonymous	93	Val	NA		1.140	0.004
p.Val93Ala	substitution	93	Val	Ala		0.891	0.006
p.Val93Arg	substitution	93	Val	Arg		0.845	0.257
p.Val93Asn	substitution	93	Val	Asn		0.657	0.012
p.Val93Asp	substitution	93	Val	Asp		0.507	0.076
p.Val93Cys	substitution	93	Val	Cys		0.675	0.010
p.Val93Glu	substitution	93	Val	Glu		0.000	0.059
p.Val93Gly	substitution	93	Val	Gly		0.603	0.011
p.Val93His	substitution	93	Val	His		1.020	0.008
p.Val93Ile	substitution	93	Val	Ile		1.148	0.003
p.Val93Leu	substitution	93	Val	Leu		0.703	0.033
p.Val93Lys	substitution	93	Val	Lys		0.000	0.986
p.Val93Met	substitution	93	Val	Met		0.727	0.048
p.Val93Phe	substitution	93	Val	Phe		0.410	0.010
p.Val93Pro	substitution	93	Val	Pro		0.032	0.087
p.Val93Ser	substitution	93	Val	Ser		0.172	0.177
p.Val93Ter	nonsense	93	Val	Ter		0.000	0.029
p.Val93Thr	substitution	93	Val	Thr		0.407	0.022
p.Val93Trp	substitution	93	Val	Trp		0.000	0.040
p.Val93Tyr	substitution	93	Val	Tyr		0.020	0.017
p.Val94=	synonymous	94	Val	NA		0.944	0.024
p.Val94Ala	substitution	94	Val	Ala		0.000	0.139
p.Val94Arg	substitution	94	Val	Arg		0.000	0.059
p.Val94Asn	substitution	94	Val	Asn		0.122	0.154
p.Val94Asp	substitution	94	Val	Asp		0.080	1.429
p.Val94Cys	substitution	94	Val	Cys		1.100	0.007
p.Val94Gln	substitution	94	Val	Gln		0.345	0.040

p.Val94Glu	substitution	94	Val	Glu	0.088	0.021
p.Val94Gly	substitution	94	Val	Gly	0.174	0.017
p.Val94His	substitution	94	Val	His	0.471	0.347
p.Val94Ile	substitution	94	Val	Ile	0.543	0.068
p.Val94Leu	substitution	94	Val	Leu	0.606	0.061
p.Val94Lys	substitution	94	Val	Lys	0.040	0.120
p.Val94Met	substitution	94	Val	Met	0.000	0.267
p.Val94Phe	substitution	94	Val	Phe	0.190	0.019
p.Val94Pro	substitution	94	Val	Pro	0.363	0.128
p.Val94Ser	substitution	94	Val	Ser	0.000	0.058
p.Val94Ter	nonsense	94	Val	Ter	0.505	0.054
p.Val94Thr	substitution	94	Val	Thr	0.468	0.086
p.Val94Trp	substitution	94	Val	Trp	0.000	0.037
p.Val94Tyr	substitution	94	Val	Tyr	0.061	0.109

Log Likelihood Ratio of Pathogenicity

LLR	95% CI	Evidence Strength for Variant Class.
-1.096	[-1.099;-1.09	benign supporting
1.276	[0.200;5.105]	pathogenic strong
1.276	[-1.089;5.062	pathogenic strong
1.116	[0.804;1.237]	pathogenic moderate
-1.107	[-1.162;-1.08	benign supporting
-0.397	[-0.451;-0.34	benign supporting
1.276	[1.042;1.871]	pathogenic strong
-0.462	[-0.579;-0.37	benign supporting
1.276	[1.195;1.389]	pathogenic strong
1.276	[1.209;1.360]	pathogenic strong
-0.463	[-0.666;-0.28	benign supporting
1.276	[1.167;1.443]	pathogenic strong
1.276	[1.200;1.374]	pathogenic strong
1.276	[1.199;1.377]	pathogenic strong
0.293	[-0.206;0.838	indeterminate
-0.693	[-0.846;-0.60	benign supporting
0.304	[0.016;0.513]	indeterminate
-0.754	[-0.860;-0.66	benign supporting
1.276	[1.184;1.405]	pathogenic strong
0.764	[0.469;1.003]	pathogenic moderate
-0.345	[-0.505;-0.11	benign supporting
-1.116	[-1.168;-1.08	benign supporting
1.276	[1.201;1.377]	pathogenic strong
-1.015	[-1.065;-0.96	benign supporting
0.378	[0.264;0.480]	pathogenic supporting
-0.107	[-0.207;0.000	indeterminate
0.547	[0.262;0.756]	pathogenic supporting
-0.977	[-1.007;-0.94	benign supporting
0.844	[0.759;0.936]	pathogenic moderate
1.134	[1.004;1.213]	pathogenic moderate
0.656	[0.243;0.940]	pathogenic moderate
1.131	[0.965;1.214]	pathogenic moderate
-0.483	[-0.554;-0.41	benign supporting
1.194	[1.123;1.275]	pathogenic moderate
0.750	[0.490;0.971]	pathogenic moderate
-0.827	[-1.055;-0.57	benign supporting
1.276	[1.209;1.360]	pathogenic strong
-0.863	[-0.913;-0.81	benign supporting
1.276	[1.217;1.351]	pathogenic strong
1.162	[1.127;1.202]	pathogenic moderate

-1.105	[-1.108;-1.10	benign supporting
-0.852	[-0.888;-0.81	benign supporting
-1.054	[-1.070;-1.02	benign supporting
-1.090	[-1.107;-1.07	benign supporting
-1.091	[-1.104;-1.08	benign supporting
-0.965	[-0.999;-0.93	benign supporting
-1.164	[-1.169;-1.15	benign supporting
1.276	[1.109;1.687]	pathogenic strong
-1.056	[-1.072;-1.02	benign supporting
-1.023	[-1.045;-1.00	benign supporting
-0.003	[-0.494;1.14(	indeterminate
-1.066	[-1.094;-1.00	benign supporting
-0.538	[-0.570;-0.50	benign supporting
1.127	[0.963;1.207]	pathogenic moderate
-0.794	[-0.906;-0.67	benign supporting
0.947	[-0.005;1.27[illegible]	pathogenic moderate
-0.831	[-1.065;-0.57	benign supporting
1.276	[0.892;5.022]	pathogenic strong
-0.564	[-0.622;-0.50	benign supporting
-1.030	[-1.059;-1.00	benign supporting
-0.674	[-0.707;-0.64	benign supporting
-0.966	[-0.986;-0.94	benign supporting
-1.066	[-1.084;-1.02	benign supporting
-1.063	[-1.101;-0.95	benign supporting
-0.370	[-0.396;-0.34	benign supporting
0.892	[-1.165;4.95(	pathogenic moderate
-0.888	[-1.000;-0.74	benign supporting
0.792	[0.704;0.885]	pathogenic moderate
0.428	[0.237;0.585]	pathogenic supporting
-0.797	[-0.896;-0.68	benign supporting
-1.165	[-1.170;-1.14	benign supporting
-1.108	[-1.115;-1.09	benign supporting
-1.105	[-1.115;-1.09	benign supporting
-0.463	[-0.605;-0.34	benign supporting
-0.852	[-0.917;-0.77	benign supporting
1.036	[0.849;1.137]	pathogenic moderate
-0.371	[-0.494;-0.21	benign supporting
-0.365	[-0.621;0.01[illegible]	benign supporting
-1.084	[-1.121;-1.08	benign supporting
-0.864	[-0.891;-0.83	benign supporting
-0.639	[-0.751;-0.56	benign supporting
-1.084	[-1.115;-1.08	benign supporting
-0.957	[-1.165;-0.83	benign supporting

-0.253	[-0.638;0.867	indeterminate
-0.852	[-3.763;0.000	benign supporting
-0.050	[-0.444;1.028	indeterminate
-1.170	[-1.169;-1.10	benign supporting
-0.720	[-1.113;-0.04	benign supporting
-1.114	[-1.165;-0.75	benign supporting
1.276	[1.213;1.359	pathogenic strong
1.235	[1.165;1.316	pathogenic moderate
1.276	[1.199;1.377	pathogenic strong
-0.863	[-0.898;-0.83	benign supporting
1.224	[1.040;1.456	pathogenic moderate
1.213	[0.992;1.444	pathogenic moderate
-0.697	[-0.780;-0.64	benign supporting
1.276	[1.228;1.333	pathogenic strong
1.276	[0.966;2.770	pathogenic strong
1.276	[1.191;1.392	pathogenic strong
0.339	[-0.019;0.618	pathogenic supporting
1.209	[1.135;1.294	pathogenic moderate
1.276	[1.065;1.790	pathogenic strong
1.170	[0.686;1.492	pathogenic moderate
-0.557	[-1.056;-0.11	benign supporting
0.225	[-0.659;1.404	indeterminate
0.187	[-0.335;1.016	indeterminate
1.276	[0.622;5.087	pathogenic strong
1.276	[1.224;1.340	pathogenic strong
1.276	[1.246;1.309	pathogenic strong
-0.867	[-1.020;-0.67	benign supporting
1.276	[1.129;1.565	pathogenic strong
0.860	[0.711;1.019	pathogenic moderate
0.889	[0.650;1.102	pathogenic moderate
1.276	[-0.239;5.100	pathogenic strong
1.276	[1.148;1.496	pathogenic strong
-0.158	[-0.200;-0.11	indeterminate
1.276	[-0.194;5.108	pathogenic strong
1.276	[1.190;1.392	pathogenic strong
1.276	[1.223;1.340	pathogenic strong
0.871	[-0.019;1.225	pathogenic moderate
1.276	[1.217;1.349	pathogenic strong
1.102	[1.020;1.135	pathogenic moderate
1.276	[1.188;1.401	pathogenic strong
-0.562	[-0.694;-0.42	benign supporting
-0.643	[-0.660;-0.62	benign supporting
1.276	[1.146;1.509	pathogenic strong

1.112	[0.425;1.359	pathogenic moderate
1.276	[1.108;1.680	pathogenic strong
-0.637	[-0.728;-0.57	benign supporting
1.276	[1.243;1.314	pathogenic strong
-0.183	[-0.206;-0.16	indeterminate
-0.187	[-0.243;-0.12	indeterminate
-0.826	[-0.854;-0.78	benign supporting
1.276	[1.174;1.424	pathogenic strong
0.657	[0.613;0.700	pathogenic moderate
-0.592	[-0.615;-0.56	benign supporting
1.125	[1.110;1.141	pathogenic moderate
1.273	[1.226;1.327	pathogenic strong
1.276	[1.242;1.316	pathogenic strong
-1.164	[-1.164;-1.16	benign supporting
-0.025	[-0.085;0.10	indeterminate
-0.617	[-0.647;-0.58	benign supporting
-0.355	[-0.542;-0.09	benign supporting
1.276	[1.225;1.336	pathogenic strong
-0.486	[-0.494;-0.47	benign supporting
1.276	[1.202;1.373	pathogenic strong
1.276	[1.229;1.333	pathogenic strong
1.276	[1.255;1.298	pathogenic strong
-0.968	[-1.028;-0.90	benign supporting
1.227	[0.321;5.064	pathogenic moderate
1.276	[1.196;1.387	pathogenic strong
1.276	[0.909;5.021	pathogenic strong
1.276	[1.166;1.441	pathogenic strong
0.371	[-0.269;1.04	pathogenic supporting
-0.618	[-0.998;-0.35	benign supporting
1.276	[1.164;1.455	pathogenic strong
1.276	[1.246;1.309	pathogenic strong
1.276	[-0.156;5.11	pathogenic strong
1.276	[0.000;5.107	pathogenic strong
1.276	[1.218;1.347	pathogenic strong
-0.633	[-0.946;-0.42	benign supporting
-0.321	[-0.368;-0.26	benign supporting
0.875	[0.444;1.144	pathogenic moderate
-1.091	[-1.168;-0.65	benign supporting
-1.090	[-1.092;-1.08	benign supporting
-0.447	[-0.497;-0.40	benign supporting
1.174	[1.137;1.214	pathogenic moderate
-0.997	[-1.037;-0.94	benign supporting
-1.091	[-1.114;-1.07	benign supporting

-1.103	[-1.110;-1.09	benign supporting
0.000	[0.000;0.000	indeterminate
-1.065	[-1.079;-1.02	benign supporting
-1.104	[-1.115;-1.09	benign supporting
-0.413	[-0.426;-0.40	benign supporting
-0.591	[-0.666;-0.51	benign supporting
-0.920	[-3.548;-0.83	benign supporting
-1.018	[-1.037;-0.99	benign supporting
-0.844	[-0.891;-0.78	benign supporting
-0.337	[-0.393;-0.26	benign supporting
-0.573	[-0.616;-0.52	benign supporting
1.236	[1.188;1.289	pathogenic moderate
-0.637	[-0.870;-0.49	benign supporting
-0.839	[-0.839;-0.83	benign supporting
-1.105	[-1.115;-1.02	benign supporting
-1.113	[-1.115;-1.08	benign supporting
-0.986	[-1.028;-0.96	benign supporting
1.276	[1.213;1.357	pathogenic strong
1.276	[1.059;1.835	pathogenic strong
1.082	[0.999;1.128	pathogenic moderate
1.276	[1.172;1.428	pathogenic strong
1.234	[1.043;1.497	pathogenic moderate
1.276	[1.248;1.307	pathogenic strong
1.276	[1.202;1.373	pathogenic strong
1.276	[1.064;1.816	pathogenic strong
0.867	[0.503;1.123	pathogenic moderate
1.002	[0.848;1.108	pathogenic moderate
1.276	[1.167;1.449	pathogenic strong
1.276	[1.226;1.336	pathogenic strong
0.901	[0.012;1.237	pathogenic moderate
-1.088	[-1.142;-1.08	benign supporting
1.276	[1.020;1.887	pathogenic strong
-1.168	[-1.169;-1.11	benign supporting
1.276	[1.196;1.383	pathogenic strong
1.276	[1.234;1.325	pathogenic strong
-0.394	[-0.454;-0.33	benign supporting
-1.095	[-1.115;-0.93	benign supporting
1.159	[0.743;1.394	pathogenic moderate
1.276	[1.241;1.317	pathogenic strong
1.276	[0.935;4.832	pathogenic strong
1.276	[-0.221;5.10	pathogenic strong
-0.579	[-0.597;-0.56	benign supporting
-0.165	[-0.605;1.03	indeterminate

1.276	[-0.031;5.098	pathogenic strong
1.192	[1.128;1.262]	pathogenic moderate
1.276	[0.948;4.851]	pathogenic strong
1.276	[1.064;1.747]	pathogenic strong
1.276	[1.165;1.445]	pathogenic strong
-0.971	[-3.655;0.000	benign supporting
-1.169	[-1.169;-0.93	benign supporting
1.276	[0.902;5.036]	pathogenic strong
1.276	[1.175;1.420]	pathogenic strong
-1.127	[-1.169;-1.04	benign supporting
-0.956	[-1.080;-0.74	benign supporting
1.121	[1.086;1.145]	pathogenic moderate
-0.629	[-0.729;-0.55	benign supporting
1.276	[0.622;5.097]	pathogenic strong
1.276	[0.864;5.019]	pathogenic strong
0.782	[0.179;1.129]	pathogenic moderate
0.969	[0.230;1.238]	pathogenic moderate
1.104	[-0.392;5.037	pathogenic moderate
1.206	[1.111;1.324]	pathogenic moderate
-0.514	[-0.540;-0.48	benign supporting
1.276	[0.734;5.084]	pathogenic strong
1.276	[-0.079;5.103	pathogenic strong
1.193	[0.422;4.045]	pathogenic moderate
-0.352	[-1.164;4.819	benign supporting
-0.987	[-1.167;-0.88	benign supporting
0.217	[0.081;0.327]	indeterminate
-1.101	[-1.115;-1.08	benign supporting
1.152	[1.118;1.188]	pathogenic moderate
-1.094	[-1.115;-1.00	benign supporting
-1.086	[-1.089;-1.08	benign supporting
-1.166	[-1.169;-1.16	benign supporting
-0.115	[-0.271;0.163	indeterminate
-0.983	[-1.000;-0.96	benign supporting
-0.409	[-0.424;-0.39	benign supporting
1.276	[1.264;1.290]	pathogenic strong
-3.211	[-3.338;-3.02	benign strong
-1.151	[-1.169;-1.12	benign supporting
-1.148	[-1.168;-1.13	benign supporting
1.276	[1.136;1.525]	pathogenic strong
-0.380	[-0.985;0.800	benign supporting
-0.209	[-0.337;-0.03	indeterminate
-0.355	[-0.393;-0.31	benign supporting
-0.359	[-0.380;-0.33	benign supporting

-0.086	[-0.123;-0.04	indeterminate
-1.105	[-1.114;-1.09	benign supporting
-1.087	[-1.101;-1.08	benign supporting
1.276	[1.130;1.558	pathogenic strong
1.276	[1.022;1.942	pathogenic strong
-0.957	[-1.113;-0.49	benign supporting
-0.891	[-0.899;-0.88	benign supporting
0.698	[0.579;0.790	pathogenic moderate
0.573	[0.432;0.692	pathogenic supporting
-0.899	[-1.111;-0.42	benign supporting
1.276	[0.695;5.085	pathogenic strong
0.417	[-0.413;1.219	pathogenic supporting
1.276	[1.242;1.314	pathogenic strong
1.276	[-0.228;5.106	pathogenic strong
1.276	[1.171;1.437	pathogenic strong
1.276	[-0.268;5.105	pathogenic strong
-0.105	[-0.394;0.708	indeterminate
1.276	[1.229;1.331	pathogenic strong
-1.079	[-1.114;-0.96	benign supporting
-1.069	[-1.077;-1.05	benign supporting
0.929	[0.390;1.191	pathogenic moderate
-0.371	[-1.090;1.149	benign supporting
-0.625	[-0.659;-0.59	benign supporting
-1.096	[-1.167;-0.74	benign supporting
-1.000	[-1.167;-0.89	benign supporting
-0.904	[-3.594;-0.83	benign supporting
1.276	[1.210;1.361	pathogenic strong
-0.837	[-0.848;-0.82	benign supporting
1.119	[0.868;1.221	pathogenic moderate
1.253	[1.013;1.676	pathogenic moderate
-1.039	[-1.049;-1.03	benign supporting
-1.050	[-1.168;-0.95	benign supporting
-1.145	[-1.155;-1.13	benign supporting
1.141	[-0.375;5.056	pathogenic moderate
-0.837	[-0.837;-0.83	benign supporting
1.276	[1.137;1.529	pathogenic strong
-0.854	[-1.105;-0.47	benign supporting
-0.682	[-0.860;-0.57	benign supporting
-1.101	[-1.115;-1.07	benign supporting
1.276	[1.216;1.352	pathogenic strong
-0.163	[-0.351;0.249	indeterminate
0.697	[0.044;1.077	pathogenic moderate
-0.525	[-0.560;-0.49	benign supporting

1.276	[1.259;1.295	pathogenic strong
1.276	[1.253;1.301	pathogenic strong
1.276	[1.267;1.286	pathogenic strong
1.276	[1.270;1.283	pathogenic strong
1.276	[1.251;1.303	pathogenic strong
1.276	[1.274;1.278	pathogenic strong
-0.574	[-0.574;-0.57	benign supporting
1.276	[1.263;1.290	pathogenic strong
1.276	[1.267;1.286	pathogenic strong
1.276	[1.252;1.302	pathogenic strong
1.276	[1.258;1.296	pathogenic strong
1.276	[1.220;1.346	pathogenic strong
1.276	[1.267;1.286	pathogenic strong
1.276	[1.238;1.319	pathogenic strong
-0.960	[-1.007;-0.91	benign supporting
1.276	[1.251;1.304	pathogenic strong
1.276	[0.932;4.959	pathogenic strong
1.276	[1.268;1.285	pathogenic strong
1.276	[1.260;1.293	pathogenic strong
1.276	[1.235;1.324	pathogenic strong
-0.912	[-1.015;-0.76	benign supporting
1.131	[1.049;1.181	pathogenic moderate
1.276	[0.525;5.090	pathogenic strong
-0.663	[-0.698;-0.63	benign supporting
-0.277	[-0.301;-0.25	indeterminate
1.216	[-0.145;5.08	pathogenic moderate
-1.023	[-1.030;-1.01	benign supporting
-1.167	[-1.170;-1.12	benign supporting
1.276	[1.154;1.476	pathogenic strong
1.276	[1.174;1.426	pathogenic strong
-0.540	[-1.110;0.99	benign supporting
0.759	[0.708;0.811	pathogenic moderate
0.639	[-0.628;4.26	pathogenic moderate
1.263	[1.227;1.301	pathogenic moderate
0.988	[-0.218;1.51	pathogenic moderate
1.276	[1.184;1.404	pathogenic strong
1.276	[-0.202;5.10	pathogenic strong
-1.102	[-1.166;-0.61	benign supporting
-1.120	[-1.153;-1.09	benign supporting
1.276	[1.138;1.530	pathogenic strong
-0.928	[-0.939;-0.91	benign supporting
-0.308	[-0.584;0.45	indeterminate
-1.086	[-1.092;-1.08	benign supporting

-1.084	[-1.086;-1.08	benign supporting
-1.080	[-1.088;-1.07	benign supporting
-3.739	[-3.790;-3.67	benign strong
-0.703	[-1.165;1.354	benign supporting
1.243	[0.986;1.614]	pathogenic moderate
1.276	[0.837;5.060]	pathogenic strong
-0.977	[-1.168;-0.83	benign supporting
1.276	[0.954;2.454]	pathogenic strong
-0.392	[-0.465;-0.31	benign supporting
-0.260	[-0.408;-0.01	indeterminate
-0.493	[-1.050;0.215	benign supporting
-0.850	[-1.113;-0.32	benign supporting
1.276	[0.956;4.756]	pathogenic strong
-1.169	[-1.169;-1.13	benign supporting
-0.886	[-2.612;4.622	benign supporting
1.276	[0.245;5.101]	pathogenic strong
1.276	[0.721;5.096]	pathogenic strong
-0.397	[-1.094;1.131	benign supporting
-1.099	[-1.107;-1.09	benign supporting
1.112	[1.038;1.147]	pathogenic moderate
-0.845	[-3.693;-0.83	benign supporting
1.276	[1.196;1.380]	pathogenic strong
1.143	[1.115;1.173]	pathogenic moderate
1.276	[1.223;1.340]	pathogenic strong
0.866	[0.193;1.187]	pathogenic moderate
0.957	[0.846;1.054]	pathogenic moderate
-0.609	[-1.068;-0.25	benign supporting
0.603	[0.062;0.913]	pathogenic supporting
-1.069	[-1.084;-1.03	benign supporting
1.108	[0.352;1.367]	pathogenic moderate
-0.316	[-0.402;-0.17	indeterminate
1.276	[1.225;1.336]	pathogenic strong
1.276	[0.799;5.068]	pathogenic strong
-1.011	[-1.068;-0.96	benign supporting
1.276	[1.179;1.416]	pathogenic strong
1.276	[-0.009;5.110	pathogenic strong
1.276	[-0.049;5.101	pathogenic strong
-0.366	[-1.164;4.934	benign supporting
1.276	[-0.321;5.094	pathogenic strong
-1.085	[-1.091;-1.08	benign supporting
0.934	[-0.439;4.745	pathogenic moderate
-0.946	[-1.105;-0.62	benign supporting
-1.168	[-1.169;-1.10	benign supporting

-0.899	[-0.925;-0.86	benign supporting
1.276	[0.638;5.090	pathogenic strong
1.104	[-0.170;1.83	pathogenic moderate
-1.088	[-1.167;-1.08	benign supporting
1.276	[1.032;1.857	pathogenic strong
0.651	[-0.244;1.19	pathogenic moderate
1.260	[-0.343;5.09	pathogenic moderate
1.276	[-0.354;5.09	pathogenic strong
-0.056	[-0.295;0.58	indeterminate
1.243	[1.004;1.635	pathogenic moderate
-0.899	[-3.807;0.00	benign supporting
-1.072	[-1.114;-0.76	benign supporting
-1.165	[-1.169;-1.03	benign supporting
1.276	[1.128;1.579	pathogenic strong
-0.860	[-0.877;-0.84	benign supporting
1.258	[1.177;1.363	pathogenic moderate
-0.839	[-3.759;0.00	benign supporting
1.227	[-0.417;5.09	pathogenic moderate
1.276	[-0.080;5.11	pathogenic strong
-1.024	[-1.038;-1.00	benign supporting
-1.102	[-1.164;-0.96	benign supporting
-1.070	[-3.591;0.00	benign supporting
1.276	[0.686;5.095	pathogenic strong
1.117	[0.967;1.184	pathogenic moderate
-1.084	[-1.086;-1.08	benign supporting
-1.164	[-1.169;-1.13	benign supporting
0.851	[0.351;1.144	pathogenic moderate
-1.097	[-1.168;-0.99	benign supporting
1.276	[1.033;1.881	pathogenic strong
0.336	[-0.202;0.87	pathogenic supporting
-1.164	[-1.169;-1.08	benign supporting
-0.071	[-0.303;0.51	indeterminate
1.276	[1.082;1.739	pathogenic strong
-1.080	[-1.100;-1.05	benign supporting
1.276	[-0.190;5.09	pathogenic strong
1.276	[1.204;1.365	pathogenic strong
-1.114	[-1.169;-1.02	benign supporting
0.601	[-0.422;1.34	pathogenic supporting
-0.381	[-0.613;-0.04	benign supporting
1.276	[1.219;1.346	pathogenic strong
1.276	[1.138;1.521	pathogenic strong
1.276	[0.650;5.085	pathogenic strong
-1.068	[-1.096;-1.00	benign supporting

-1.085	[-1.168;-0.76	benign supporting
1.276	[1.203;1.372	pathogenic strong
-0.994	[-1.014;-0.97	benign supporting
-1.100	[-1.115;-1.07	benign supporting
-0.447	[-0.517;-0.39	benign supporting
-0.408	[-0.428;-0.39	benign supporting
-1.041	[-1.065;-1.01	benign supporting
-1.148	[-1.166;-1.12	benign supporting
-0.454	[-0.941;0.03	benign supporting
-0.863	[-0.895;-0.83	benign supporting
-1.089	[-1.092;-1.08	benign supporting
-0.565	[-0.581;-0.54	benign supporting
-0.494	[-0.526;-0.46	benign supporting
-0.674	[-0.724;-0.63	benign supporting
-1.085	[-1.092;-1.08	benign supporting
-1.091	[-1.098;-1.08	benign supporting
-0.403	[-0.420;-0.38	benign supporting
1.191	[0.808;1.480	pathogenic moderate
-0.799	[-0.881;-0.70	benign supporting
0.684	[-0.108;1.14	pathogenic moderate
-1.090	[-1.115;-1.03	benign supporting
-0.911	[-0.933;-0.88	benign supporting
-1.098	[-1.106;-1.09	benign supporting
-1.106	[-1.115;-1.07	benign supporting
-1.114	[-1.116;-1.11	benign supporting
-1.100	[-1.108;-1.09	benign supporting
-1.088	[-1.098;-1.08	benign supporting
-0.669	[-0.687;-0.65	benign supporting
0.186	[-0.101;0.56	indeterminate
-0.838	[-0.861;-0.81	benign supporting
-1.110	[-1.116;-1.10	benign supporting
-1.064	[-1.075;-1.03	benign supporting
-0.775	[-0.865;-0.68	benign supporting
-0.735	[-1.110;-0.21	benign supporting
-0.844	[-0.898;-0.77	benign supporting
-1.087	[-1.089;-1.08	benign supporting
1.130	[1.107;1.153	pathogenic moderate
-1.115	[-1.116;-1.09	benign supporting
-0.270	[-0.319;-0.21	indeterminate
-1.082	[-1.091;-1.07	benign supporting
1.276	[0.995;2.450	pathogenic strong
-1.104	[-1.115;-1.08	benign supporting
-0.853	[-0.864;-0.84	benign supporting

-1.096	[-1.110;-1.08	benign supporting
-1.084	[-1.103;-1.08	benign supporting
-1.115	[-1.168;-1.08	benign supporting
-0.878	[-0.975;-0.75	benign supporting
-0.459	[-0.486;-0.43	benign supporting
-1.029	[-1.040;-1.01	benign supporting
-0.518	[-0.549;-0.48	benign supporting
-0.908	[-0.973;-0.83	benign supporting
-0.855	[-0.861;-0.85	benign supporting
-0.609	[-0.617;-0.59	benign supporting
-1.105	[-1.115;-1.09	benign supporting
-0.583	[-0.600;-0.56	benign supporting
-0.917	[-0.930;-0.90	benign supporting
-0.443	[-0.462;-0.42	benign supporting
-0.435	[-0.460;-0.41	benign supporting
-1.137	[-1.165;-1.10	benign supporting
-1.049	[-1.081;-0.99	benign supporting
-1.106	[-1.115;-1.09	benign supporting
1.202	[1.158;1.248	pathogenic moderate
-0.604	[-0.802;-0.45	benign supporting
-0.599	[-0.625;-0.56	benign supporting
-1.043	[-1.063;-1.02	benign supporting
-1.086	[-1.100;-1.07	benign supporting
-1.166	[-3.422;1.872	benign supporting
-1.130	[-1.169;-0.94	benign supporting
0.753	[-0.135;1.205	pathogenic moderate
0.000	[0.000;0.000	indeterminate
-0.576	[-0.859;-0.37	benign supporting
1.276	[-0.736;5.098	pathogenic strong
-0.751	[-0.971;-0.57	benign supporting
1.132	[-1.006;5.070	pathogenic moderate
-1.041	[-1.071;-1.01	benign supporting
-0.639	[-1.112;0.242	benign supporting
-0.841	[-0.874;-0.83	benign supporting
1.276	[1.128;1.583	pathogenic strong
1.276	[0.000;5.102	pathogenic strong
1.141	[-0.928;5.062	pathogenic moderate
1.276	[0.959;3.298	pathogenic strong
1.276	[-0.463;5.098	pathogenic strong
-1.150	[-1.169;-1.09	benign supporting
1.276	[1.164;1.450	pathogenic strong
-0.957	[-1.110;-0.56	benign supporting
-0.902	[-0.930;-0.86	benign supporting

-0.063	[-0.170;0.181	indeterminate
-0.408	[-0.448;-0.37	benign supporting
-0.530	[-0.556;-0.50	benign supporting
-0.460	[-0.519;-0.41	benign supporting
-0.232	[-0.277;-0.17	indeterminate
-0.340	[-0.358;-0.32	benign supporting
-0.418	[-0.470;-0.37	benign supporting
-0.600	[-0.724;-0.48	benign supporting
0.011	[-0.357;0.903	indeterminate
-0.344	[-0.550;-0.04	benign supporting
-0.595	[-0.639;-0.54	benign supporting
-1.115	[-1.168;-1.08	benign supporting
-1.064	[-1.099;-0.96	benign supporting
-0.233	[-0.533;0.672	indeterminate
-0.761	[-0.997;-0.57	benign supporting
1.200	[0.840;1.510	pathogenic moderate
-1.048	[-1.106;-0.91	benign supporting
-0.142	[-0.246;-0.04	indeterminate
-0.599	[-1.167;4.633	benign supporting
-0.462	[-0.517;-0.41	benign supporting
-1.089	[-1.120;-1.08	benign supporting
-0.509	[-0.524;-0.49	benign supporting
1.112	[1.078;1.131	pathogenic moderate
0.947	[0.894;0.999	pathogenic moderate
-0.366	[-0.378;-0.35	benign supporting
0.611	[0.446;0.745	pathogenic supporting
0.321	[0.163;0.443	pathogenic supporting
-0.281	[-0.330;-0.22	indeterminate
1.061	[1.000;1.111	pathogenic moderate
1.276	[1.260;1.293	pathogenic strong
1.133	[1.122;1.145	pathogenic moderate
1.194	[1.181;1.207	pathogenic moderate
1.230	[1.207;1.255	pathogenic moderate
1.009	[0.798;1.130	pathogenic moderate
-0.488	[-0.553;-0.42	benign supporting
1.138	[0.932;1.239	pathogenic moderate
0.851	[0.819;0.884	pathogenic moderate
0.928	[0.890;0.968	pathogenic moderate
0.994	[0.943;1.029	pathogenic moderate
1.276	[1.256;1.298	pathogenic strong
-1.106	[-1.115;-1.07	benign supporting
1.276	[1.195;1.388	pathogenic strong
1.276	[0.061;5.107	pathogenic strong

1.050	[0.899;1.130	pathogenic moderate
1.250	[1.197;1.310	pathogenic moderate
1.271	[1.231;1.315	pathogenic moderate
1.276	[-0.453;5.090	pathogenic strong
1.276	[1.179;1.418	pathogenic strong
-0.203	[-0.249;-0.15	indeterminate
1.052	[-0.328;4.889	pathogenic moderate
1.276	[1.194;1.396	pathogenic strong
1.234	[1.187;1.287	pathogenic moderate
1.276	[0.489;5.097	pathogenic strong
-0.335	[-0.798;0.737	benign supporting
1.276	[1.199;1.381	pathogenic strong
1.276	[1.237;1.322	pathogenic strong
1.276	[0.854;5.064	pathogenic strong
1.276	[1.193;1.392	pathogenic strong
1.210	[1.176;1.244	pathogenic moderate
-0.418	[-1.102;1.138	benign supporting
1.276	[0.553;5.100	pathogenic strong
-0.966	[-1.011;-0.92	benign supporting
1.276	[-1.086;5.070	pathogenic strong
-0.213	[-0.261;-0.15	indeterminate
1.276	[0.834;5.045	pathogenic strong
1.276	[1.230;1.331	pathogenic strong
1.012	[0.449;1.232	pathogenic moderate
0.597	[0.272;0.823	pathogenic supporting
1.276	[1.156;1.471	pathogenic strong
1.276	[1.200;1.377	pathogenic strong
1.276	[1.212;1.360	pathogenic strong
-0.055	[-0.118;0.046	indeterminate
1.276	[1.188;1.404	pathogenic strong
1.175	[0.046;4.871	pathogenic moderate
1.154	[0.965;1.269	pathogenic moderate
1.231	[-0.080;5.095	pathogenic moderate
1.276	[-0.126;5.106	pathogenic strong
1.150	[1.111;1.192	pathogenic moderate
1.276	[1.236;1.321	pathogenic strong
-0.113	[-1.087;1.903	indeterminate
-1.099	[-1.114;-1.08	benign supporting
-1.092	[-1.101;-1.08	benign supporting
-1.081	[-1.106;-1.05	benign supporting
-0.407	[-0.475;-0.34	benign supporting
-0.666	[-0.687;-0.64	benign supporting
-1.072	[-1.096;-1.02	benign supporting

-0.994	[-1.016;-0.96	benign supporting
-0.493	[-0.517;-0.46	benign supporting
-1.085	[-1.089;-1.08	benign supporting
-1.137	[-1.169;-1.11	benign supporting
-0.761	[-0.854;-0.67	benign supporting
-1.107	[-1.115;-0.99	benign supporting
-1.125	[-1.149;-1.10	benign supporting
0.644	[0.573;0.711	pathogenic moderate
-1.105	[-1.115;-1.08	benign supporting
-1.085	[-1.098;-1.08	benign supporting
1.239	[1.143;1.361	pathogenic moderate
-1.115	[-1.115;-1.07	benign supporting
-1.059	[-1.068;-1.04	benign supporting
-0.049	[-0.113;0.06	indeterminate
-1.102	[-1.113;-1.09	benign supporting
-1.120	[-1.148;-1.10	benign supporting
-1.060	[-1.082;-1.00	benign supporting
-1.024	[-1.038;-1.01	benign supporting
-1.107	[-1.114;-1.10	benign supporting
-0.914	[-0.928;-0.89	benign supporting
-0.623	[-0.637;-0.61	benign supporting
-1.134	[-1.146;-1.12	benign supporting
-0.932	[-0.945;-0.91	benign supporting
-1.088	[-1.093;-1.08	benign supporting
-0.715	[-0.750;-0.68	benign supporting
-0.946	[-0.964;-0.92	benign supporting
-1.111	[-1.116;-1.10	benign supporting
-1.074	[-1.087;-1.05	benign supporting
-0.629	[-0.642;-0.61	benign supporting
-0.376	[-0.438;-0.30	benign supporting
-1.037	[-1.059;-1.01	benign supporting
1.276	[1.234;1.325	pathogenic strong
-1.096	[-1.104;-1.08	benign supporting
-1.059	[-1.069;-1.04	benign supporting
-0.390	[-0.405;-0.37	benign supporting
-0.888	[-0.920;-0.85	benign supporting
-1.085	[-1.111;-1.08	benign supporting
-0.862	[-0.887;-0.83	benign supporting
-0.510	[-0.537;-0.48	benign supporting
0.495	[0.402;0.581	pathogenic supporting
0.777	[0.733;0.823	pathogenic moderate
-0.683	[-0.767;-0.62	benign supporting
1.276	[1.251;1.303	pathogenic strong

-0.789	[-0.993;-0.60	benign supporting
-0.391	[-0.410;-0.37	benign supporting
-0.604	[-0.624;-0.58	benign supporting
-0.630	[-0.650;-0.61	benign supporting
-0.239	[-0.291;-0.17	indeterminate
-0.378	[-0.391;-0.36	benign supporting
0.251	[0.113;0.360	indeterminate
1.095	[1.057;1.113	pathogenic moderate
-0.571	[-0.607;-0.53	benign supporting
1.116	[1.093;1.133	pathogenic moderate
-1.073	[-1.080;-1.06	benign supporting
-0.364	[-0.622;0.03	benign supporting
-0.628	[-0.637;-0.61	benign supporting
-0.618	[-0.639;-0.59	benign supporting
-1.113	[-1.166;-0.78	benign supporting
1.276	[1.239;1.318	pathogenic strong
1.208	[1.184;1.233	pathogenic moderate
1.276	[1.154;1.477	pathogenic strong
1.276	[1.244;1.312	pathogenic strong
1.276	[-0.458;5.09	pathogenic strong
1.276	[1.253;1.302	pathogenic strong
1.188	[1.158;1.219	pathogenic moderate
1.276	[1.211;1.357	pathogenic strong
1.211	[1.149;1.278	pathogenic moderate
1.043	[0.953;1.111	pathogenic moderate
1.276	[1.249;1.307	pathogenic strong
1.276	[1.243;1.314	pathogenic strong
1.276	[1.216;1.352	pathogenic strong
1.276	[1.220;1.343	pathogenic strong
1.276	[1.172;1.434	pathogenic strong
1.276	[1.193;1.384	pathogenic strong
1.276	[0.899;5.019	pathogenic strong
1.276	[1.200;1.379	pathogenic strong
1.276	[1.245;1.312	pathogenic strong
-1.028	[-1.065;-0.99	benign supporting
-1.027	[-1.075;-0.95	benign supporting
-1.010	[-1.055;-0.95	benign supporting
-0.346	[-0.464;-0.16	benign supporting
-1.067	[-1.081;-1.03	benign supporting
-1.085	[-1.094;-1.08	benign supporting
-0.066	[-0.103;-0.01	indeterminate
-0.848	[-0.967;-0.68	benign supporting
0.105	[-0.080;0.41	indeterminate

-0.743	[-0.805;-0.69	benign supporting
-1.097	[-1.106;-1.09	benign supporting
-1.090	[-1.094;-1.08	benign supporting
1.170	[1.139;1.203	pathogenic moderate
1.021	[-0.034;1.362	pathogenic moderate
-1.016	[-1.111;-0.69	benign supporting
1.152	[1.133;1.173	pathogenic moderate
-0.611	[-0.804;-0.46	benign supporting
-1.052	[-1.091;-0.96	benign supporting
-0.192	[-0.316;-0.04	indeterminate
-1.094	[-1.114;-1.08	benign supporting
-1.085	[-1.128;-1.08	benign supporting
1.142	[1.094;1.184	pathogenic moderate
1.276	[1.231;1.328	pathogenic strong
1.276	[-0.305;5.102	pathogenic strong
0.686	[-0.205;1.184	pathogenic moderate
1.045	[1.002;1.090	pathogenic moderate
1.128	[1.063;1.170	pathogenic moderate
1.115	[0.840;1.224	pathogenic moderate
-0.827	[-1.053;-0.57	benign supporting
-1.030	[-1.168;-0.83	benign supporting
-1.005	[-1.096;-0.82	benign supporting
1.268	[1.188;1.374	pathogenic moderate
1.276	[1.177;1.423	pathogenic strong
0.876	[0.328;1.165	pathogenic moderate
1.122	[1.021;1.175	pathogenic moderate
1.276	[0.847;5.046	pathogenic strong
1.276	[1.238;1.320	pathogenic strong
1.276	[1.223;1.338	pathogenic strong
0.228	[0.072;0.351	indeterminate
-0.874	[-0.930;-0.81	benign supporting
1.048	[-0.334;4.598	pathogenic moderate
-0.610	[-0.610;-0.60	benign supporting
0.783	[-1.082;5.044	pathogenic moderate
-1.092	[-1.164;-1.04	benign supporting
-1.092	[-1.100;-1.08	benign supporting
-1.096	[-1.103;-1.09	benign supporting
-0.520	[-0.521;-0.52	benign supporting
-0.887	[-1.074;-0.63	benign supporting
-1.091	[-1.115;-1.08	benign supporting
1.276	[1.258;1.296	pathogenic strong
-0.361	[-0.387;-0.33	benign supporting
-1.095	[-1.103;-1.08	benign supporting

-0.388	[-0.399;-0.37	benign supporting
-0.666	[-0.731;-0.62	benign supporting
1.276	[1.213;1.357	pathogenic strong
-0.587	[-0.688;-0.48	benign supporting
1.276	[1.203;1.371	pathogenic strong
-3.225	[-3.519;0.00(	benign strong
-1.094	[-1.105;-1.08	benign supporting
-0.739	[-0.764;-0.71	benign supporting
1.276	[0.584;5.097	pathogenic strong
0.749	[0.696;0.802	pathogenic moderate
0.845	[0.465;1.116	pathogenic moderate
1.276	[0.804;5.064	pathogenic strong
1.215	[1.112;1.344	pathogenic moderate
-0.288	[-0.358;-0.18	indeterminate
-0.657	[-0.672;-0.64	benign supporting
-0.965	[-0.969;-0.96	benign supporting
-0.858	[-0.935;-0.76	benign supporting
-1.083	[-1.111;-1.05	benign supporting
1.236	[1.113;1.406	pathogenic moderate
1.147	[1.137;1.156	pathogenic moderate
-0.327	[-0.332;-0.32	benign supporting
-0.989	[-0.995;-0.98	benign supporting
1.276	[0.447;5.096	pathogenic strong
-0.480	[-0.503;-0.45	benign supporting
1.276	[0.951;4.946	pathogenic strong
0.699	[-0.412;1.41!	pathogenic moderate
-0.746	[-0.805;-0.69	benign supporting
1.102	[0.835;1.197	pathogenic moderate
-1.111	[-1.113;-1.11	benign supporting
0.348	[0.309;0.386	pathogenic supporting
-1.085	[-1.124;-1.08	benign supporting
-0.523	[-0.622;-0.42	benign supporting
1.276	[0.853;5.064	pathogenic strong
-0.388	[-0.425;-0.34	benign supporting
-0.645	[-0.652;-0.63	benign supporting
-0.433	[-0.485;-0.39	benign supporting
-1.164	[-1.169;-1.11	benign supporting
-0.940	[-0.977;-0.90	benign supporting
-0.930	[-0.960;-0.89	benign supporting
0.430	[0.407;0.453	pathogenic supporting
-1.082	[-1.093;-1.07	benign supporting
-0.946	[-0.950;-0.94	benign supporting
-0.832	[-0.841;-0.82	benign supporting

-0.520	[-0.530;-0.50	benign supporting
-3.662	[-3.821;-1.43	benign strong
-1.166	[-1.168;-1.16	benign supporting
-1.165	[-1.166;-1.16	benign supporting
-1.100	[-1.102;-1.09	benign supporting
0.719	[0.699;0.739	pathogenic moderate
-0.827	[-0.835;-0.81	benign supporting
-0.069	[-0.078;-0.05	indeterminate
-1.070	[-1.072;-1.06	benign supporting
1.276	[1.212;1.358	pathogenic strong
-1.010	[-1.053;-0.96	benign supporting
-1.144	[-1.148;-1.14	benign supporting
-0.643	[-0.662;-0.62	benign supporting
-1.126	[-1.145;-1.11	benign supporting
-1.084	[-1.087;-1.08	benign supporting
-1.167	[-1.169;-1.01	benign supporting
-0.477	[-0.537;-0.42	benign supporting
1.276	[1.227;1.334	pathogenic strong
0.525	[0.077;0.796	pathogenic supporting
-1.169	[-1.170;-1.16	benign supporting
1.276	[0.861;5.027	pathogenic strong
-0.872	[-1.114;-0.34	benign supporting
0.312	[0.134;0.452	indeterminate
-0.190	[-0.308;-0.05	indeterminate
-0.861	[-0.983;-0.70	benign supporting
-0.128	[-0.173;-0.08	indeterminate
-0.655	[-0.660;-0.65	benign supporting
0.715	[-0.053;1.135	pathogenic moderate
0.513	[-0.065;0.916	pathogenic supporting
-1.104	[-1.115;-1.06	benign supporting
1.276	[1.171;1.425	pathogenic strong
-0.548	[-0.559;-0.53	benign supporting
-0.088	[-0.107;-0.06	indeterminate
-0.089	[-0.150;-0.02	indeterminate
-1.095	[-1.167;-0.78	benign supporting
-1.060	[-1.110;-0.92	benign supporting
1.276	[-0.166;5.101	pathogenic strong
0.189	[-1.095;4.948	indeterminate
1.276	[1.240;1.318	pathogenic strong
0.964	[-0.519;5.026	pathogenic moderate
1.276	[0.721;5.084	pathogenic strong
1.177	[1.130;1.229	pathogenic moderate
1.276	[1.196;1.384	pathogenic strong

1.276	[1.160;1.463	pathogenic strong
1.202	[1.148;1.261	pathogenic moderate
1.276	[1.254;1.301	pathogenic strong
-1.095	[-1.098;-1.09	benign supporting
-0.938	[-0.961;-0.91	benign supporting
1.230	[0.125;5.076	pathogenic moderate
-0.021	[-0.166;0.363	indeterminate
-0.164	[-0.452;0.692	indeterminate
1.276	[1.244;1.312	pathogenic strong
-0.176	[-0.439;0.609	indeterminate
-0.924	[-0.941;-0.90	benign supporting
1.276	[1.257;1.297	pathogenic strong
-0.537	[-1.113;1.038	benign supporting
-0.398	[-0.599;-0.16	benign supporting
0.347	[-0.340;1.121	pathogenic supporting
1.126	[1.088;1.155	pathogenic moderate
-1.095	[-1.113;-1.08	benign supporting
1.276	[-0.183;5.105	pathogenic strong
-0.045	[-0.650;1.230	indeterminate
-1.105	[-1.108;-1.10	benign supporting
1.171	[-0.312;5.077	pathogenic moderate
-1.027	[-1.096;-0.88	benign supporting
-1.112	[-1.167;-0.54	benign supporting
-1.071	[-1.076;-1.06	benign supporting
1.116	[0.875;1.212	pathogenic moderate
1.276	[1.270;1.283	pathogenic strong
1.276	[1.271;1.281	pathogenic strong
1.276	[1.272;1.280	pathogenic strong
1.276	[1.226;1.337	pathogenic strong
1.276	[1.149;1.491	pathogenic strong
1.276	[1.215;1.353	pathogenic strong
1.276	[1.263;1.290	pathogenic strong
1.276	[1.228;1.333	pathogenic strong
1.276	[1.264;1.289	pathogenic strong
1.276	[1.261;1.292	pathogenic strong
1.276	[1.141;1.514	pathogenic strong
1.276	[1.261;1.292	pathogenic strong
1.276	[1.253;1.301	pathogenic strong
1.276	[1.240;1.320	pathogenic strong
1.276	[1.264;1.289	pathogenic strong
1.276	[1.204;1.368	pathogenic strong
1.276	[1.264;1.288	pathogenic strong
1.276	[1.258;1.296	pathogenic strong

1.276	[1.204;1.368]	pathogenic strong
-1.087	[-1.126;-1.08	benign supporting
-1.097	[-1.115;-1.00	benign supporting
-0.197	[-0.230;-0.16	indeterminate
0.909	[0.863;0.960]	pathogenic moderate
-0.442	[-1.164;4.557	benign supporting
-0.221	[-0.240;-0.20	indeterminate
-1.107	[-1.115;-1.09	benign supporting
-0.997	[-1.012;-0.97	benign supporting
-0.907	[-0.966;-0.83	benign supporting
-1.033	[-1.051;-1.01	benign supporting
-0.951	[-0.955;-0.94	benign supporting
-0.384	[-0.395;-0.37	benign supporting
-1.132	[-1.134;-1.13	benign supporting
-0.395	[-1.091;1.135	benign supporting
-1.102	[-1.115;-1.01	benign supporting
1.243	[1.204;1.284]	pathogenic moderate
-0.729	[-1.111;-0.21	benign supporting
1.276	[1.262;1.290]	pathogenic strong
1.203	[1.127;1.286]	pathogenic moderate
-0.908	[-1.093;-0.61	benign supporting
-0.679	[-0.967;-0.48	benign supporting
0.448	[0.254;0.606]	pathogenic supporting
-1.084	[-1.086;-1.08	benign supporting
-0.320	[-0.449;-0.10	benign supporting
-1.133	[-1.169;-1.08	benign supporting
-1.032	[-1.058;-1.00	benign supporting
-1.086	[-1.088;-1.08	benign supporting
-1.085	[-1.086;-1.08	benign supporting
-1.081	[-1.169;-0.96	benign supporting
-1.144	[-1.169;-1.11	benign supporting
-0.505	[-0.526;-0.48	benign supporting
1.276	[0.174;5.102]	pathogenic strong
-1.141	[-1.145;-1.13	benign supporting
-1.164	[-1.169;-1.05	benign supporting
-0.666	[-0.907;-0.51	benign supporting
1.229	[1.108;1.396]	pathogenic moderate
-0.618	[-0.652;-0.58	benign supporting
-1.098	[-1.114;-1.08	benign supporting
-0.420	[-0.430;-0.41	benign supporting
1.244	[1.013;1.577]	pathogenic moderate
-1.084	[-1.086;-1.08	benign supporting
-0.567	[-0.575;-0.55	benign supporting

-1.085	[-1.085;-1.08	benign supporting
0.610	[0.599;0.620]	pathogenic supporting
-1.067	[-1.076;-1.04	benign supporting
-1.151	[-1.152;-1.15	benign supporting
-0.446	[-0.448;-0.44	benign supporting
-1.050	[-1.051;-1.04	benign supporting
-0.459	[-0.472;-0.44	benign supporting
-1.092	[-1.097;-1.08	benign supporting
-0.613	[-0.636;-0.58	benign supporting
-0.589	[-0.592;-0.58	benign supporting
-1.089	[-1.090;-1.08	benign supporting
-0.405	[-0.408;-0.40	benign supporting
1.276	[1.248;1.307]	pathogenic strong
-0.616	[-0.638;-0.59	benign supporting
1.276	[1.271;1.282]	pathogenic strong
-0.342	[-0.358;-0.32	benign supporting
-0.715	[-0.717;-0.71	benign supporting
-1.157	[-1.160;-1.15	benign supporting
-0.737	[-0.803;-0.68	benign supporting
-1.086	[-1.098;-1.08	benign supporting
1.137	[-0.371;5.06(	pathogenic moderate
-0.916	[-1.046;-0.83	benign supporting
1.276	[0.585;5.094]	pathogenic strong
-1.089	[-1.093;-1.08	benign supporting
-1.078	[-3.543;0.00(	benign supporting
-0.899	[-0.935;-0.85	benign supporting
0.053	[-0.582;1.23	indeterminate
-0.933	[-0.937;-0.92	benign supporting
-0.921	[-2.976;-0.83	benign supporting
-1.046	[-1.081;-1.01	benign supporting
0.594	[-0.387;1.27	pathogenic supporting
-0.950	[-0.954;-0.94	benign supporting
-0.518	[-0.559;-0.47	benign supporting
-1.084	[-1.167;-1.02	benign supporting
-1.114	[-1.116;-1.10	benign supporting
-1.112	[-1.149;-1.09	benign supporting
-1.039	[-1.071;-1.00	benign supporting
-1.066	[-1.167;-0.99	benign supporting
-1.085	[-1.093;-1.08	benign supporting
-0.641	[-0.696;-0.60	benign supporting
-1.166	[-1.170;-1.14	benign supporting
0.827	[0.538;1.078]	pathogenic moderate
-1.071	[-1.114;-0.80	benign supporting

-1.103	[-1.143;-0.99	benign supporting
-0.261	[-0.567;0.61	indeterminate
-0.564	[-0.629;-0.49	benign supporting
-1.068	[-1.080;-1.04	benign supporting
-0.809	[-1.107;-0.38	benign supporting
-0.967	[-1.086;-0.75	benign supporting
-1.123	[-1.169;-1.08	benign supporting
1.025	[0.891;1.114	pathogenic moderate
-1.164	[-1.168;-1.16	benign supporting
-0.892	[-0.932;-0.84	benign supporting
-1.092	[-1.101;-1.08	benign supporting
-1.089	[-1.096;-1.08	benign supporting
-0.232	[-0.281;-0.17	indeterminate
-0.950	[-0.975;-0.92	benign supporting
-0.285	[-0.340;-0.21	indeterminate
0.720	[0.396;0.966	pathogenic moderate
0.586	[0.382;0.742	pathogenic supporting
-0.058	[-0.089;-0.02	indeterminate
-0.050	[-0.187;0.30	indeterminate
-1.066	[-1.105;-0.96	benign supporting
1.143	[0.457;1.498	pathogenic moderate
-0.684	[-0.861;-0.58	benign supporting
-1.045	[-1.090;-0.94	benign supporting
-0.020	[-0.366;0.86	indeterminate
1.276	[1.099;1.685	pathogenic strong
-1.073	[-1.086;-1.05	benign supporting
1.275	[1.252;1.299	pathogenic strong
-0.588	[-0.630;-0.54	benign supporting
0.360	[0.141;0.529	pathogenic supporting
-0.734	[-0.966;-0.56	benign supporting
-1.089	[-1.112;-1.06	benign supporting
-1.087	[-1.091;-1.08	benign supporting
-0.844	[-0.916;-0.75	benign supporting
-0.612	[-0.635;-0.58	benign supporting
-1.165	[-1.169;-1.03	benign supporting
-1.090	[-1.103;-1.07	benign supporting
-1.102	[-1.107;-1.09	benign supporting
-1.005	[-1.040;-0.96	benign supporting
-0.928	[-1.081;-0.67	benign supporting
-1.039	[-1.085;-0.94	benign supporting
-1.109	[-1.115;-1.09	benign supporting
-1.088	[-1.102;-1.08	benign supporting
-0.974	[-1.028;-0.91	benign supporting

0.854	[0.765;0.954	pathogenic moderate
-0.355	[-0.770;0.61(	benign supporting
-1.081	[-1.088;-1.07	benign supporting
1.276	[1.254;1.300	pathogenic strong
-1.073	[-1.090;-1.04	benign supporting
-1.102	[-1.115;-1.09	benign supporting
-1.085	[-1.091;-1.08	benign supporting
-0.399	[-1.114;1.40	benign supporting
-0.840	[-0.849;-0.83	benign supporting
-1.070	[-1.099;-1.01	benign supporting
1.140	[1.097;1.183	pathogenic moderate
-1.168	[-1.169;-1.16	benign supporting
-1.093	[-1.115;-1.03	benign supporting
1.276	[1.113;1.630	pathogenic strong
-0.446	[-0.454;-0.43	benign supporting
-0.679	[-0.849;-0.57	benign supporting
-1.166	[-1.169;-1.03	benign supporting
-1.104	[-1.115;-1.08	benign supporting
0.486	[0.213;0.697	pathogenic supporting
-1.110	[-1.116;-1.10	benign supporting
-0.942	[-0.954;-0.93	benign supporting
-1.029	[-1.052;-1.00	benign supporting
-0.707	[-1.037;-0.47	benign supporting
1.276	[1.237;1.321	pathogenic strong
-1.086	[-1.167;-0.92	benign supporting
-1.102	[-1.111;-1.09	benign supporting
-0.588	[-0.694;-0.47	benign supporting
-0.324	[-0.410;-0.19	benign supporting
-1.156	[-1.166;-1.14	benign supporting
1.276	[1.076;1.803	pathogenic strong
-1.104	[-1.167;-0.49	benign supporting
-0.942	[-3.799;0.00(	benign supporting
-1.064	[-1.102;-1.03	benign supporting
-0.872	[-0.894;-0.85	benign supporting
-1.097	[-1.099;-1.09	benign supporting
1.276	[1.104;1.660	pathogenic strong
-0.642	[-1.115;0.571	benign supporting
-0.612	[-0.810;-0.46	benign supporting
-0.220	[-0.436;0.41	indeterminate
-0.809	[-0.870;-0.73	benign supporting
-0.832	[-0.847;-0.81	benign supporting
-1.119	[-1.135;-1.10	benign supporting
-0.620	[-0.641;-0.59	benign supporting

-0.383	[-0.479;-0.27	benign supporting
1.276	[1.119;1.608]	pathogenic strong
-0.359	[-0.677;0.382	benign supporting
-1.169	[-1.170;-1.16	benign supporting
-0.592	[-0.644;-0.53	benign supporting
-1.000	[-1.025;-0.96	benign supporting
-0.911	[-0.921;-0.90	benign supporting
1.276	[1.112;1.618]	pathogenic strong
1.276	[1.182;1.411]	pathogenic strong
-1.165	[-1.168;-0.88	benign supporting
-1.081	[-1.105;-1.06	benign supporting
-1.160	[-1.169;-1.07	benign supporting
1.276	[0.635;5.087]	pathogenic strong
1.276	[-0.207;5.103	pathogenic strong
0.406	[-0.518;1.308	pathogenic supporting
-1.089	[-1.096;-1.08	benign supporting
1.088	[0.966;1.141]	pathogenic moderate
-0.511	[-1.046;-0.01	benign supporting
-1.095	[-1.166;-0.37	benign supporting
1.276	[1.202;1.374]	pathogenic strong
-1.061	[-1.067;-1.05	benign supporting
1.276	[0.407;5.095]	pathogenic strong
-1.066	[-1.114;-0.68	benign supporting
1.276	[1.177;1.418]	pathogenic strong
-1.018	[-1.030;-1.00	benign supporting
-0.592	[-0.727;-0.46	benign supporting
-1.112	[-1.116;-1.10	benign supporting
-0.964	[-1.081;-0.89	benign supporting
-0.407	[-0.443;-0.37	benign supporting
1.276	[1.219;1.349]	pathogenic strong
-0.658	[-1.164;1.159	benign supporting
-0.852	[-0.902;-0.83	benign supporting
-0.862	[-1.164;0.774	benign supporting
-0.876	[-0.880;-0.87	benign supporting
-1.023	[-1.110;-0.76	benign supporting
1.125	[0.858;1.241]	pathogenic moderate
-0.970	[-0.973;-0.96	benign supporting
-0.959	[-0.995;-0.93	benign supporting
-1.170	[-1.169;-0.96	benign supporting
-1.094	[-1.108;-1.08	benign supporting
-0.275	[-0.512;0.389	indeterminate
-1.164	[-1.165;-1.16	benign supporting
-0.764	[-0.789;-0.74	benign supporting

-1.056	[-1.166;0.184	benign supporting
0.731	[-0.259;1.262	pathogenic moderate
-0.194	[-0.291;-0.08	indeterminate
-1.095	[-1.107;-1.08	benign supporting
-1.085	[-1.105;-1.08	benign supporting
-1.046	[-1.069;-1.01	benign supporting
-0.911	[-1.165;0.629	benign supporting
-0.356	[-0.748;0.559	benign supporting
-1.104	[-1.108;-1.10	benign supporting
-1.092	[-1.102;-1.08	benign supporting
-1.547	[-3.834;-1.05	benign strong
-0.216	[-0.288;-0.12	indeterminate
-0.809	[-1.112;-0.30	benign supporting
-1.103	[-1.158;-1.08	benign supporting
-1.000	[-1.051;-0.96	benign supporting
-1.082	[-1.152;-1.03	benign supporting
1.276	[-0.422;5.091	pathogenic strong
1.118	[1.040;1.159]	pathogenic moderate
-1.007	[-1.166;-0.91	benign supporting
-0.923	[-0.927;-0.91	benign supporting
-0.895	[-0.905;-0.88	benign supporting
-1.040	[-1.048;-1.03	benign supporting
-1.124	[-1.169;-0.96	benign supporting
-0.916	[-0.971;-0.84	benign supporting
-0.928	[-1.063;-0.83	benign supporting
-1.126	[-2.892;0.000	benign supporting
-0.877	[-0.969;-0.76	benign supporting
-1.030	[-1.061;-1.00	benign supporting
-0.332	[-0.356;-0.30	benign supporting
-1.084	[-1.086;-1.08	benign supporting
-0.585	[-0.607;-0.56	benign supporting
-0.628	[-0.830;-0.49	benign supporting
-0.950	[-0.973;-0.92	benign supporting
-1.022	[-1.031;-1.01	benign supporting
-1.097	[-1.115;-1.07	benign supporting
-1.135	[-1.148;-1.12	benign supporting
-1.101	[-1.108;-1.09	benign supporting
-1.089	[-1.092;-1.08	benign supporting
-0.397	[-0.425;-0.36	benign supporting
-1.057	[-1.073;-1.02	benign supporting
1.276	[1.204;1.371]	pathogenic strong
-1.086	[-1.114;-1.08	benign supporting
-1.085	[-1.087;-1.08	benign supporting

-1.112	[-1.115;-1.10	benign supporting
-0.294	[-0.353;-0.21	indeterminate
1.276	[1.251;1.304	pathogenic strong
1.276	[1.256;1.297	pathogenic strong
1.153	[1.134;1.172	pathogenic moderate
-0.838	[-0.852;-0.82	benign supporting
1.276	[1.229;1.331	pathogenic strong
-0.133	[-0.304;0.203	indeterminate
1.109	[1.064;1.132	pathogenic moderate
1.263	[1.232;1.295	pathogenic moderate
1.276	[1.256;1.297	pathogenic strong
1.228	[1.216;1.239	pathogenic moderate
1.276	[1.244;1.313	pathogenic strong
1.276	[1.258;1.295	pathogenic strong
1.276	[1.249;1.306	pathogenic strong
1.261	[1.226;1.297	pathogenic moderate
1.138	[1.049;1.196	pathogenic moderate
1.274	[1.226;1.329	pathogenic strong
1.276	[1.244;1.311	pathogenic strong
1.276	[1.251;1.304	pathogenic strong
1.221	[1.200;1.243	pathogenic moderate
1.276	[1.210;1.360	pathogenic strong
-0.742	[-0.832;-0.67	benign supporting
-0.336	[-0.497;-0.09	benign supporting
1.206	[1.140;1.277	pathogenic moderate
-0.626	[-0.700;-0.56	benign supporting
-1.090	[-1.099;-1.08	benign supporting
-0.098	[-0.200;0.029	indeterminate
-1.106	[-1.115;-1.09	benign supporting
-1.106	[-1.115;-1.08	benign supporting
-1.083	[-1.093;-1.07	benign supporting
1.276	[1.183;1.408	pathogenic strong
-0.941	[-1.069;-0.76	benign supporting
0.905	[0.649;1.114	pathogenic moderate
-1.055	[-1.069;-1.03	benign supporting
-0.077	[-0.126;-0.01	indeterminate
1.276	[0.000;5.102	pathogenic strong
-0.848	[-0.958;-0.70	benign supporting
1.276	[0.979;2.367	pathogenic strong
-0.412	[-0.450;-0.37	benign supporting
-0.107	[-0.161;-0.05	indeterminate
-1.102	[-1.114;-1.09	benign supporting
-0.944	[-0.974;-0.91	benign supporting

-0.698	[-0.707;-0.69	benign supporting
-1.081	[-1.165;-0.53	benign supporting
-1.085	[-1.165;-1.08	benign supporting
-1.084	[-1.168;-0.67	benign supporting
-1.101	[-1.106;-1.09	benign supporting
-0.953	[-0.960;-0.94	benign supporting
-1.044	[-1.049;-1.04	benign supporting
-1.083	[-1.132;-0.71	benign supporting
1.276	[0.948;3.564	pathogenic strong
1.276	[1.256;1.298	pathogenic strong
1.164	[1.144;1.187	pathogenic moderate
1.223	[1.158;1.295	pathogenic moderate
-0.837	[-0.891;-0.83	benign supporting
-0.612	[-1.041;-0.30	benign supporting
1.276	[1.238;1.318	pathogenic strong
1.217	[1.121;1.332	pathogenic moderate
1.276	[-0.040;5.10	pathogenic strong
1.276	[1.163;1.443	pathogenic strong
1.001	[0.903;1.075	pathogenic moderate
1.054	[-1.088;5.04	pathogenic moderate
0.307	[-0.052;0.63	indeterminate
-1.106	[-1.115;-1.09	benign supporting
-0.974	[-1.128;-0.41	benign supporting
1.276	[1.024;2.083	pathogenic strong
-0.943	[-0.945;-0.94	benign supporting
-1.086	[-1.089;-1.08	benign supporting
-0.340	[-0.439;-0.18	benign supporting
1.276	[-0.101;5.10	pathogenic strong
-0.977	[-0.986;-0.97	benign supporting
1.276	[1.201;1.373	pathogenic strong
-0.649	[-0.659;-0.64	benign supporting
-0.874	[-0.879;-0.86	benign supporting
1.276	[0.706;5.079	pathogenic strong
-1.030	[-1.164;-0.41	benign supporting
-1.087	[-1.120;-1.08	benign supporting
0.763	[0.653;0.863	pathogenic moderate
1.276	[1.193;1.389	pathogenic strong
-1.098	[-1.100;-1.09	benign supporting
-0.350	[-0.448;-0.21	benign supporting
-1.066	[-1.084;-1.02	benign supporting
-0.378	[-0.430;-0.31	benign supporting
-0.617	[-0.640;-0.59	benign supporting
-1.085	[-1.086;-1.08	benign supporting

-0.982	[-0.995;-0.97	benign supporting
-0.631	[-0.638;-0.62	benign supporting
-1.067	[-1.069;-1.06	benign supporting
-0.384	[-0.407;-0.35	benign supporting
-1.101	[-1.104;-1.09	benign supporting
-0.662	[-0.670;-0.65	benign supporting
1.201	[1.091;1.324	pathogenic moderate
-0.804	[-0.827;-0.78	benign supporting
-0.406	[-0.439;-0.37	benign supporting
-0.087	[-0.425;0.85	indeterminate
-0.999	[-1.112;-0.56	benign supporting
1.276	[1.258;1.295	pathogenic strong
-0.593	[-0.600;-0.58	benign supporting
1.234	[1.219;1.248	pathogenic moderate
-0.463	[-0.465;-0.46	benign supporting
-0.413	[-0.647;-0.14	benign supporting
-0.514	[-0.870;-0.24	benign supporting
1.276	[1.107;1.655	pathogenic strong
-1.027	[-1.036;-1.01	benign supporting
-1.107	[-1.110;-1.10	benign supporting
-1.090	[-1.093;-1.08	benign supporting
-1.041	[-1.043;-1.03	benign supporting
1.276	[1.189;1.394	pathogenic strong
-0.182	[-0.205;-0.15	indeterminate
1.276	[1.183;1.404	pathogenic strong
-0.974	[-1.015;-0.93	benign supporting
1.064	[0.941;1.131	pathogenic moderate
-0.885	[-0.945;-0.81	benign supporting
1.100	[0.751;1.223	pathogenic moderate
1.276	[1.179;1.411	pathogenic strong
1.234	[0.935;1.610	pathogenic moderate
-0.173	[-0.212;-0.13	indeterminate
1.276	[1.214;1.356	pathogenic strong
-1.086	[-1.090;-1.08	benign supporting
-1.079	[-1.166;-0.40	benign supporting
1.061	[-0.503;5.04	pathogenic moderate
-1.130	[-1.169;-1.08	benign supporting
-0.908	[-0.924;-0.89	benign supporting
-1.064	[-1.096;-0.97	benign supporting
1.276	[-1.089;5.06	pathogenic strong
-0.281	[-1.109;1.95	indeterminate
-0.073	[-0.270;0.42	indeterminate
1.276	[1.198;1.379	pathogenic strong

1.026	[0.741;1.163]	pathogenic moderate
-1.040	[-1.067;-1.01	benign supporting
-1.076	[-1.085;-1.06	benign supporting
0.501	[0.021;0.786]	pathogenic supporting
-1.075	[-1.081;-1.06	benign supporting
-1.099	[-1.120;-1.08	benign supporting
-0.453	[-0.482;-0.42	benign supporting
-1.029	[-1.042;-1.01	benign supporting
-1.085	[-1.091;-1.08	benign supporting
-0.407	[-0.421;-0.39	benign supporting
-0.638	[-0.716;-0.57	benign supporting
-0.840	[-0.896;-0.83	benign supporting
-0.562	[-0.579;-0.54	benign supporting
-0.031	[-0.066;0.013	indeterminate
-0.342	[-0.395;-0.27	benign supporting
-0.556	[-0.839;-0.36	benign supporting
-0.504	[-0.764;-0.29	benign supporting
1.276	[0.904;4.996]	pathogenic strong
-0.492	[-0.589;-0.40	benign supporting
-0.675	[-0.695;-0.65	benign supporting
0.764	[-0.021;1.153	pathogenic moderate
-1.053	[-1.077;-1.00	benign supporting
-1.086	[-1.088;-1.08	benign supporting
-0.748	[-0.891;-0.63	benign supporting
-0.239	[-0.360;-0.06	indeterminate
0.942	[0.802;1.063]	pathogenic moderate
-0.916	[-0.934;-0.89	benign supporting
-0.729	[-0.751;-0.70	benign supporting
-0.501	[-0.786;-0.27	benign supporting
-0.932	[-0.939;-0.92	benign supporting
-0.608	[-1.113;0.739	benign supporting
1.046	[0.977;1.106]	pathogenic moderate
-1.089	[-1.099;-1.08	benign supporting
-0.837	[-0.906;-0.83	benign supporting
-0.832	[-1.100;-0.46	benign supporting
1.276	[1.240;1.317]	pathogenic strong
-1.130	[-1.169;-0.87	benign supporting
-1.085	[-1.089;-1.08	benign supporting
-1.001	[-1.024;-0.96	benign supporting
-1.084	[-1.144;-1.08	benign supporting
1.276	[0.520;5.098]	pathogenic strong
0.465	[-0.464;1.300	pathogenic supporting
-1.165	[-1.169;-1.16	benign supporting

-0.891	[-0.907;-0.87	benign supporting
1.061	[-0.565;5.049	pathogenic moderate
-0.365	[-0.507;-0.16	benign supporting
-1.115	[-1.169;-1.03	benign supporting
-0.979	[-0.987;-0.97	benign supporting
1.276	[0.000;5.105]	pathogenic strong
1.276	[-0.006;5.100	pathogenic strong
0.000	[0.000;0.000]	indeterminate
-0.880	[-0.882;-0.87	benign supporting
-0.533	[-0.586;-0.47	benign supporting
0.610	[0.057;0.951]	pathogenic supporting
-0.123	[-0.971;1.353	indeterminate
-0.979	[-0.984;-0.97	benign supporting
-0.881	[-0.902;-0.86	benign supporting
-0.586	[-1.111;0.638	benign supporting
-1.098	[-1.169;-0.95	benign supporting
-0.933	[-0.988;-0.87	benign supporting
-1.076	[-1.079;-1.07	benign supporting
1.276	[1.216;1.349]	pathogenic strong
-1.015	[-1.024;-1.00	benign supporting
-1.166	[-1.170;-1.12	benign supporting
-1.091	[-1.168;-1.08	benign supporting
-1.134	[-1.169;-1.10	benign supporting
1.010	[0.941;1.064]	pathogenic moderate
0.729	[0.013;1.117]	pathogenic moderate
1.276	[1.208;1.359]	pathogenic strong
-1.165	[-1.166;-1.16	benign supporting
1.259	[1.217;1.306]	pathogenic moderate
-0.634	[-0.717;-0.57	benign supporting
-0.043	[-0.193;0.350	indeterminate
1.276	[1.237;1.319]	pathogenic strong
-0.049	[-0.198;0.333	indeterminate
-0.599	[-0.621;-0.57	benign supporting
-0.365	[-0.378;-0.35	benign supporting
-1.059	[-1.096;-0.96	benign supporting
-1.110	[-1.115;-1.10	benign supporting
-1.077	[-1.115;-0.70	benign supporting
-1.099	[-1.115;-0.88	benign supporting
-1.065	[-1.093;-1.04	benign supporting
-1.133	[-1.169;-1.08	benign supporting
-0.872	[-0.891;-0.85	benign supporting
-0.749	[-2.095;4.795	benign supporting
-1.106	[-1.115;-1.08	benign supporting

1.276	[1.196;1.384]	pathogenic strong
-0.142	[-0.320;0.219	indeterminate
1.276	[1.176;1.420]	pathogenic strong
-0.485	[-0.520;-0.45	benign supporting
-0.036	[-0.442;1.036	indeterminate
1.230	[1.050;1.456]	pathogenic moderate
1.276	[1.180;1.407]	pathogenic strong
1.276	[1.220;1.343]	pathogenic strong
1.276	[1.248;1.308]	pathogenic strong
-0.022	[-0.067;0.040	indeterminate
-1.073	[-1.084;-1.06	benign supporting
-0.186	[-0.401;0.385	indeterminate
-1.114	[-1.116;-1.09	benign supporting
-0.935	[-0.984;-0.88	benign supporting
-0.220	[-0.275;-0.15	indeterminate
1.276	[1.194;1.386]	pathogenic strong
-1.005	[-1.043;-0.95	benign supporting
0.893	[0.701;1.079]	pathogenic moderate
1.276	[-1.096;5.050	pathogenic strong
-1.113	[-1.127;-1.09	benign supporting
1.276	[1.177;1.416]	pathogenic strong
-1.083	[-1.104;-1.06	benign supporting
-0.825	[-0.841;-0.80	benign supporting
-0.663	[-0.684;-0.64	benign supporting
-1.166	[-1.168;-1.16	benign supporting
-1.120	[-1.169;-1.08	benign supporting
-0.793	[-0.815;-0.77	benign supporting
-0.855	[-1.073;-0.59	benign supporting
-0.373	[-0.663;0.165	benign supporting
-0.676	[-0.686;-0.66	benign supporting
-1.089	[-1.093;-1.08	benign supporting
-0.566	[-0.602;-0.52	benign supporting
-0.837	[-0.845;-0.83	benign supporting
-0.988	[-0.997;-0.97	benign supporting
-0.636	[-0.801;-0.52	benign supporting
-1.114	[-1.129;-1.10	benign supporting
0.646	[-0.091;1.115	pathogenic moderate
-1.029	[-1.109;-0.83	benign supporting
-1.098	[-1.115;-1.07	benign supporting
-1.085	[-1.088;-1.08	benign supporting
-0.182	[-0.388;0.360	indeterminate
0.819	[0.733;0.910]	pathogenic moderate
1.276	[0.848;5.062]	pathogenic strong

-0.417	[-0.502;-0.34	benign supporting
1.206	[1.153;1.262	pathogenic moderate
-0.976	[-0.980;-0.97	benign supporting
-1.087	[-1.092;-1.08	benign supporting
-1.085	[-1.094;-1.08	benign supporting
1.166	[0.250;1.984	pathogenic moderate
-1.079	[-1.081;-1.07	benign supporting
-0.740	[-0.878;-0.63	benign supporting
-0.429	[-0.465;-0.39	benign supporting
-0.940	[-1.006;-0.86	benign supporting
-0.336	[-0.341;-0.33	benign supporting
-0.972	[-0.976;-0.96	benign supporting
-1.004	[-1.010;-0.99	benign supporting
-1.088	[-1.095;-1.08	benign supporting
-1.085	[-1.086;-1.08	benign supporting
-0.371	[-0.380;-0.36	benign supporting
-0.648	[-0.850;-0.52	benign supporting
-1.109	[-1.116;-1.10	benign supporting
-1.164	[-1.164;-1.16	benign supporting
1.276	[1.114;1.612	pathogenic strong
-1.096	[-1.115;-1.07	benign supporting
1.078	[1.039;1.108	pathogenic moderate
-1.062	[-1.066;-1.05	benign supporting
-0.413	[-0.419;-0.40	benign supporting
-0.939	[-0.986;-0.89	benign supporting
-1.112	[-1.116;-1.10	benign supporting
-1.014	[-1.098;-0.84	benign supporting
1.276	[-1.082;5.078	pathogenic strong
1.276	[-1.119;5.004	pathogenic strong
1.276	[-1.093;5.056	pathogenic strong
1.276	[-1.112;5.026	pathogenic strong
1.276	[0.000;5.102	pathogenic strong
-1.093	[-1.168;-0.47	benign supporting
1.219	[-0.194;5.095	pathogenic moderate
0.647	[-1.108;5.033	pathogenic moderate
-1.052	[-1.169;1.243	benign supporting
-0.002	[-0.234;0.578	indeterminate
1.276	[1.173;1.425	pathogenic strong
1.191	[-0.384;5.089	pathogenic moderate
1.276	[-0.593;5.094	pathogenic strong
1.276	[0.000;5.106	pathogenic strong
1.276	[0.542;5.099	pathogenic strong
-0.187	[-1.166;4.914	indeterminate

0.165	[-0.448;1.15(	indeterminate
1.276	[-0.294;5.104	pathogenic strong
-0.372	[-0.383;-0.36	benign supporting
-1.110	[-1.113;-1.10	benign supporting
0.313	[0.129;0.450]	indeterminate
-0.096	[-0.131;-0.06	indeterminate
-0.445	[-0.515;-0.38	benign supporting
-1.143	[-1.169;-1.09	benign supporting
-0.642	[-0.706;-0.59	benign supporting
-0.945	[-0.993;-0.90	benign supporting
-0.543	[-0.582;-0.50	benign supporting
0.986	[0.866;1.076]	pathogenic moderate
-1.141	[-1.156;-1.12	benign supporting
1.117	[1.074;1.143]	pathogenic moderate
-0.829	[-0.847;-0.80	benign supporting
-0.756	[-1.000;-0.56	benign supporting
-0.919	[-1.027;-0.77	benign supporting
1.276	[1.235;1.326]	pathogenic strong
-0.763	[-0.841;-0.68	benign supporting
-0.560	[-0.575;-0.54	benign supporting
-0.810	[-0.832;-0.78	benign supporting
-0.776	[-0.833;-0.71	benign supporting
-0.972	[-0.975;-0.96	benign supporting
-0.495	[-0.540;-0.44	benign supporting
1.110	[-0.088;1.655	pathogenic moderate
-1.098	[-1.113;-1.08	benign supporting
0.394	[0.077;0.613]	pathogenic supporting
-0.271	[-0.330;-0.19	indeterminate
-1.085	[-1.152;-1.08	benign supporting
0.340	[0.142;0.494]	pathogenic supporting
1.058	[0.354;1.299]	pathogenic moderate
1.102	[0.716;1.244]	pathogenic moderate
1.235	[1.094;1.439]	pathogenic moderate
-1.104	[-1.115;-1.08	benign supporting
1.192	[1.162;1.222]	pathogenic moderate
1.276	[1.154;1.480]	pathogenic strong
0.736	[-0.118;1.178	pathogenic moderate
-0.644	[-0.766;-0.56	benign supporting
1.276	[1.187;1.396]	pathogenic strong
1.212	[1.169;1.259]	pathogenic moderate
1.276	[1.160;1.465]	pathogenic strong
0.683	[-0.307;1.252	pathogenic moderate
0.867	[0.813;0.924]	pathogenic moderate

-1.131	[-1.168;-1.10	benign supporting
1.112	[0.816;1.223]	pathogenic moderate
1.276	[1.186;1.405]	pathogenic strong
0.898	[-0.047;1.255	pathogenic moderate
1.276	[1.177;1.422]	pathogenic strong
1.276	[0.762;5.084]	pathogenic strong
-0.289	[-1.131;4.803	indeterminate
1.106	[0.706;1.249]	pathogenic moderate
-0.341	[-0.514;-0.08	benign supporting
1.276	[0.915;4.967]	pathogenic strong
1.276	[1.212;1.358]	pathogenic strong
1.276	[0.000;5.105]	pathogenic strong
0.913	[0.754;1.061]	pathogenic moderate
1.276	[0.072;5.100]	pathogenic strong
0.213	[0.004;0.398]	indeterminate
1.276	[1.207;1.367]	pathogenic strong
0.019	[-0.103;0.348	indeterminate
1.276	[1.146;1.500]	pathogenic strong
1.276	[1.237;1.320]	pathogenic strong
1.276	[1.113;1.644]	pathogenic strong
1.276	[1.129;1.550]	pathogenic strong
-1.088	[-1.092;-1.08	benign supporting
-0.017	[-0.370;0.883	indeterminate
-0.670	[-0.840;-0.57	benign supporting
-0.818	[-0.927;-0.68	benign supporting
-0.895	[-0.980;-0.78	benign supporting
-1.098	[-1.108;-1.08	benign supporting
1.276	[1.229;1.331]	pathogenic strong
-0.413	[-0.469;-0.36	benign supporting
0.582	[-0.178;1.118	pathogenic supporting
-1.090	[-1.103;-1.08	benign supporting
-0.853	[-0.875;-0.83	benign supporting
-0.463	[-0.514;-0.42	benign supporting
-0.523	[-0.556;-0.49	benign supporting
-0.939	[-1.112;-0.47	benign supporting
-1.109	[-1.115;-1.07	benign supporting
1.262	[1.093;1.573]	pathogenic moderate
-1.113	[-1.144;-1.09	benign supporting
-0.604	[-0.630;-0.57	benign supporting
-0.130	[-0.597;1.093	indeterminate
-0.086	[-0.283;0.406	indeterminate
-1.087	[-1.089;-1.08	benign supporting
-0.959	[-1.082;-0.74	benign supporting

1.276	[1.138;1.535	pathogenic strong
1.276	[1.238;1.321	pathogenic strong
-0.437	[-0.606;-0.29	benign supporting
-0.945	[-1.115;-0.41	benign supporting
1.276	[1.187;1.397	pathogenic strong
-0.908	[-0.912;-0.90	benign supporting
-1.009	[-1.048;-0.97	benign supporting
1.276	[1.104;1.667	pathogenic strong
-1.066	[-1.114;-0.70	benign supporting
1.196	[-0.092;5.07	pathogenic moderate
0.483	[-0.355;1.19	pathogenic supporting
-0.382	[-0.635;-0.02	benign supporting
-0.720	[-1.098;-0.35	benign supporting
-1.081	[-3.300;1.58	benign supporting
-0.965	[-0.971;-0.96	benign supporting
-1.020	[-1.167;-0.92	benign supporting
1.276	[1.180;1.413	pathogenic strong
-0.292	[-0.328;-0.24	indeterminate
1.276	[1.126;1.578	pathogenic strong
0.771	[0.707;0.837	pathogenic moderate
-0.265	[-0.418;-0.00	indeterminate
1.276	[-0.059;5.10	pathogenic strong
1.276	[0.735;5.080	pathogenic strong
0.612	[0.376;0.789	pathogenic supporting
1.069	[-0.439;5.02	pathogenic moderate
-0.087	[-0.547;1.11	indeterminate
-0.831	[-1.050;-0.58	benign supporting
1.276	[1.008;2.107	pathogenic strong
-0.139	[-0.236;-0.04	indeterminate
1.108	[1.064;1.129	pathogenic moderate
-0.915	[-1.105;-0.55	benign supporting
-0.650	[-0.679;-0.62	benign supporting
-1.001	[-1.012;-0.98	benign supporting
1.276	[1.135;1.527	pathogenic strong
1.224	[1.150;1.309	pathogenic moderate
1.276	[0.618;5.098	pathogenic strong
0.561	[0.159;0.815	pathogenic supporting
1.196	[0.330;4.703	pathogenic moderate
1.276	[1.236;1.324	pathogenic strong
0.796	[-0.953;5.03	pathogenic moderate
1.276	[1.203;1.372	pathogenic strong
1.276	[1.115;1.630	pathogenic strong
1.276	[0.844;5.050	pathogenic strong

1.276	[1.192;1.388]	pathogenic strong
1.276	[0.789;5.064]	pathogenic strong
0.605	[0.174;0.878]	pathogenic supporting
1.276	[0.904;4.956]	pathogenic strong
1.276	[0.977;2.490]	pathogenic strong
1.192	[0.660;1.716]	pathogenic moderate
-0.466	[-1.151;1.679	benign supporting
-0.432	[-1.005;0.538	benign supporting
1.276	[1.244;1.312]	pathogenic strong
-1.087	[-1.093;-1.08	benign supporting
0.552	[-0.884;4.846	pathogenic supporting
1.192	[1.134;1.253]	pathogenic moderate
1.276	[1.163;1.453]	pathogenic strong
1.276	[1.182;1.413]	pathogenic strong
-0.455	[-0.498;-0.42	benign supporting
0.483	[-0.180;1.045	pathogenic supporting
-0.475	[-0.495;-0.45	benign supporting
-0.237	[-0.390;0.017	indeterminate
1.172	[1.127;1.222]	pathogenic moderate
-0.397	[-0.453;-0.34	benign supporting
1.114	[0.982;1.174]	pathogenic moderate
-0.401	[-0.422;-0.38	benign supporting
1.276	[-0.483;5.100	pathogenic strong
-1.030	[-1.070;-0.97	benign supporting
-0.585	[-0.608;-0.56	benign supporting
1.157	[-0.622;5.086	pathogenic moderate
0.577	[-0.381;1.275	pathogenic supporting
-1.114	[-1.116;-1.09	benign supporting
-0.089	[-0.921;1.363	indeterminate
-0.929	[-0.964;-0.89	benign supporting
-1.113	[-1.122;-1.10	benign supporting
-1.022	[-1.057;-0.98	benign supporting
-0.327	[-0.364;-0.28	benign supporting
-1.090	[-1.167;-0.74	benign supporting
-0.893	[-0.922;-0.86	benign supporting
-0.991	[-1.008;-0.96	benign supporting
-1.100	[-1.113;-1.08	benign supporting
-1.049	[-1.062;-1.03	benign supporting
-0.881	[-0.924;-0.83	benign supporting
-1.090	[-1.095;-1.08	benign supporting
-0.999	[-1.014;-0.97	benign supporting
-1.021	[-1.052;-0.99	benign supporting
-0.857	[-1.034;-0.63	benign supporting

-1.084	[-1.090;-1.08	benign supporting
1.276	[1.201;1.375	pathogenic strong
-0.989	[-1.015;-0.95	benign supporting
-0.988	[-1.007;-0.96	benign supporting
-0.634	[-0.655;-0.61	benign supporting
-0.869	[-0.895;-0.84	benign supporting
-0.917	[-0.973;-0.85	benign supporting
-1.170	[-1.170;-1.14	benign supporting
-1.085	[-1.090;-1.08	benign supporting
-0.852	[-0.869;-0.83	benign supporting
-0.507	[-0.567;-0.44	benign supporting
-0.997	[-1.018;-0.97	benign supporting
-0.874	[-0.904;-0.84	benign supporting
-1.071	[-1.111;-0.97	benign supporting
1.148	[1.120;1.176	pathogenic moderate
-1.070	[-1.093;-1.01	benign supporting
-1.086	[-1.092;-1.08	benign supporting
-0.876	[-0.926;-0.82	benign supporting
1.183	[1.139;1.230	pathogenic moderate
-0.723	[-0.789;-0.67	benign supporting
-1.030	[-1.112;-0.67	benign supporting
0.592	[0.391;0.753	pathogenic supporting
-0.938	[-0.958;-0.91	benign supporting
-0.207	[-0.286;-0.11	indeterminate
-1.052	[-1.080;-0.99	benign supporting
-0.684	[-0.731;-0.64	benign supporting
-0.567	[-0.786;-0.40	benign supporting
-0.451	[-0.552;-0.37	benign supporting
-1.090	[-1.106;-1.08	benign supporting
-0.195	[-0.236;-0.15	indeterminate
-0.493	[-0.519;-0.46	benign supporting
1.276	[1.153;1.485	pathogenic strong
-1.166	[-1.168;-1.16	benign supporting
-1.023	[-1.113;-0.64	benign supporting
-0.661	[-0.704;-0.62	benign supporting
-0.460	[-0.482;-0.44	benign supporting
-0.687	[-0.758;-0.63	benign supporting
-1.069	[-1.081;-1.04	benign supporting
-0.869	[-0.899;-0.84	benign supporting
-0.866	[-0.894;-0.84	benign supporting
-0.056	[-0.241;0.41	indeterminate
-0.865	[-0.928;-0.79	benign supporting
1.199	[1.133;1.272	pathogenic moderate

-1.095	[-1.100;-1.09	benign supporting
-0.878	[-0.913;-0.84	benign supporting
-1.165	[-1.168;-1.16	benign supporting
-0.224	[-0.396;0.20(	indeterminate
-1.118	[-1.148;-1.09	benign supporting
-1.103	[-1.115;-1.08	benign supporting
-1.085	[-1.168;-0.99	benign supporting
-0.259	[-0.365;-0.10	indeterminate
-0.865	[-0.886;-0.84	benign supporting
1.162	[1.120;1.209]	pathogenic moderate
-0.092	[-0.125;-0.05	indeterminate
-0.058	[-0.113;0.01{	indeterminate
0.025	[-0.158;0.50	indeterminate
1.276	[-0.312;5.10	pathogenic strong
-0.781	[-0.958;-0.62	benign supporting
-1.103	[-1.106;-1.09	benign supporting
-0.955	[-0.962;-0.94	benign supporting
1.276	[1.179;1.421]	pathogenic strong
-0.816	[-1.137;-0.11	benign supporting
1.118	[1.030;1.163]	pathogenic moderate
0.893	[-1.151;4.99	pathogenic moderate
1.129	[0.900;1.234]	pathogenic moderate
0.611	[-0.004;1.00	pathogenic supporting
-1.126	[-1.169;-0.99	benign supporting
-0.929	[-1.059;-0.75	benign supporting
1.060	[0.899;1.141]	pathogenic moderate
-0.662	[-0.943;-0.47	benign supporting
1.276	[1.111;1.670]	pathogenic strong
1.214	[1.180;1.251]	pathogenic moderate
1.276	[1.176;1.429]	pathogenic strong
-0.711	[-1.110;-0.20	benign supporting
-0.350	[-1.033;1.10	benign supporting
-1.110	[-1.114;-1.10	benign supporting
1.276	[1.182;1.409]	pathogenic strong
-1.166	[-1.169;-1.04	benign supporting
-0.446	[-0.462;-0.43	benign supporting
1.276	[0.765;5.075]	pathogenic strong
-0.537	[-0.569;-0.50	benign supporting
-0.845	[-3.784;0.00(	benign supporting
0.376	[0.234;0.496]	pathogenic supporting
1.276	[1.141;1.519]	pathogenic strong
1.276	[1.253;1.302]	pathogenic strong
-0.516	[-0.531;-0.50	benign supporting

-1.105	[-1.111;-1.10	benign supporting
-0.086	[-1.165;4.967	indeterminate
-0.186	[-0.506;0.746	indeterminate
1.276	[1.020;2.030	pathogenic strong
1.276	[1.173;1.430	pathogenic strong
1.276	[-0.354;5.096	pathogenic strong
1.227	[1.179;1.278	pathogenic moderate
1.119	[1.016;1.171	pathogenic moderate
1.276	[1.215;1.357	pathogenic strong
-0.102	[-0.133;-0.07	indeterminate
1.226	[1.205;1.247	pathogenic moderate
1.276	[1.105;1.667	pathogenic strong
0.809	[0.691;0.935	pathogenic moderate
1.276	[1.190;1.395	pathogenic strong
1.276	[1.143;1.517	pathogenic strong
-0.444	[-1.025;0.513	benign supporting
-0.645	[-1.111;0.251	benign supporting
1.113	[-0.409;5.038	pathogenic moderate
1.276	[1.246;1.310	pathogenic strong
1.276	[1.252;1.303	pathogenic strong
-0.223	[-0.518;0.676	indeterminate
1.276	[1.230;1.332	pathogenic strong
-0.335	[-0.415;-0.22	benign supporting
-1.093	[-1.114;-1.07	benign supporting
-1.088	[-1.089;-1.08	benign supporting
-1.087	[-1.088;-1.08	benign supporting
-1.167	[-1.170;-1.16	benign supporting
-1.084	[-1.085;-1.08	benign supporting
-1.102	[-1.127;-1.08	benign supporting
-1.069	[-1.071;-1.06	benign supporting
0.666	[0.372;0.878	pathogenic moderate
-0.638	[-0.652;-0.62	benign supporting
-0.546	[-0.557;-0.53	benign supporting
-1.076	[-1.078;-1.07	benign supporting
-1.086	[-1.087;-1.08	benign supporting
-0.336	[-0.542;-0.00	benign supporting
1.164	[1.062;1.250	pathogenic moderate
1.276	[-0.402;5.089	pathogenic strong
-0.439	[-0.585;-0.32	benign supporting
-1.091	[-1.093;-1.09	benign supporting
-0.077	[-0.169;0.077	indeterminate
-0.743	[-1.164;1.076	benign supporting
1.276	[1.209;1.361	pathogenic strong

-1.091	[-1.167;-1.08	benign supporting
1.163	[1.146;1.181	pathogenic moderate
1.264	[1.138;1.467	pathogenic moderate
1.276	[1.186;1.402	pathogenic strong
0.391	[-0.244;1.01	pathogenic supporting
-0.837	[-0.838;-0.83	benign supporting
-1.170	[-1.169;-1.00	benign supporting
0.419	[-0.303;1.12	pathogenic supporting
1.276	[1.139;1.526	pathogenic strong
-1.108	[-1.115;-1.08	benign supporting
-0.909	[-0.914;-0.90	benign supporting
-0.122	[-0.190;-0.06	indeterminate
-0.469	[-1.105;1.08	benign supporting
1.276	[1.250;1.305	pathogenic strong
1.276	[1.068;1.783	pathogenic strong
1.276	[1.230;1.333	pathogenic strong
1.276	[1.258;1.296	pathogenic strong
1.276	[1.074;1.798	pathogenic strong
-1.103	[-1.164;-0.95	benign supporting
-1.059	[-1.070;-1.04	benign supporting
-0.504	[-0.636;-0.38	benign supporting
-0.757	[-0.884;-0.64	benign supporting
-0.970	[-0.985;-0.95	benign supporting
-0.842	[-0.885;-0.78	benign supporting
-1.094	[-1.100;-1.08	benign supporting
-1.114	[-1.161;-1.06	benign supporting
-0.558	[-0.606;-0.50	benign supporting
-1.169	[-1.170;-1.12	benign supporting
-0.900	[-0.904;-0.89	benign supporting
-1.102	[-1.112;-1.09	benign supporting
0.413	[0.384;0.442	pathogenic supporting
-1.089	[-1.093;-1.08	benign supporting
-0.634	[-0.645;-0.62	benign supporting
-0.359	[-0.373;-0.34	benign supporting
-1.142	[-1.169;-1.08	benign supporting
-0.079	[-0.338;0.59	indeterminate
-0.898	[-1.016;-0.73	benign supporting
-0.294	[-0.311;-0.27	indeterminate
-0.099	[-0.119;-0.07	indeterminate
-0.402	[-0.446;-0.36	benign supporting
-1.085	[-1.164;-1.08	benign supporting
1.276	[1.248;1.309	pathogenic strong
-1.084	[-1.085;-1.08	benign supporting

-1.087	[-1.094;-1.08	benign supporting
1.115	[1.079;1.137	pathogenic moderate
1.276	[1.246;1.311	pathogenic strong
-1.000	[-1.011;-0.98	benign supporting
-1.111	[-1.116;-1.10	benign supporting
-1.110	[-1.169;-1.08	benign supporting
-0.342	[-0.360;-0.32	benign supporting
-1.094	[-1.119;-1.07	benign supporting
-1.098	[-1.113;-1.08	benign supporting
1.276	[1.253;1.302	pathogenic strong
-0.431	[-0.559;-0.33	benign supporting
-0.521	[-0.616;-0.42	benign supporting
1.042	[-0.983;5.06	pathogenic moderate
-0.034	[-0.311;0.68	indeterminate
-0.396	[-0.420;-0.37	benign supporting
-0.897	[-0.945;-0.84	benign supporting
-1.109	[-1.116;-1.09	benign supporting
-1.170	[-1.170;-1.16	benign supporting
-1.109	[-1.116;-1.09	benign supporting
-1.085	[-1.085;-1.08	benign supporting
-0.959	[-0.960;-0.95	benign supporting
-1.090	[-1.091;-1.08	benign supporting
-1.161	[-1.163;-1.15	benign supporting
-0.348	[-0.353;-0.34	benign supporting
-1.166	[-1.166;-1.16	benign supporting
-0.907	[-0.911;-0.90	benign supporting
-0.337	[-0.340;-0.33	benign supporting
-1.014	[-1.019;-1.00	benign supporting
-1.094	[-1.095;-1.09	benign supporting
-1.091	[-1.092;-1.09	benign supporting
-0.331	[-0.339;-0.32	benign supporting
-0.922	[-0.927;-0.91	benign supporting
-1.115	[-1.116;-1.10	benign supporting
1.276	[1.253;1.300	pathogenic strong
-0.784	[-0.818;-0.75	benign supporting
-0.969	[-0.970;-0.96	benign supporting
-0.580	[-0.584;-0.57	benign supporting
-0.364	[-0.366;-0.36	benign supporting
-1.167	[-1.169;-1.16	benign supporting
-1.042	[-1.085;-1.00	benign supporting
-1.094	[-1.102;-1.08	benign supporting
-0.988	[-1.160;-0.91	benign supporting
1.276	[1.207;1.361	pathogenic strong

-1.088	[-1.089;-1.08	benign supporting
-1.086	[-1.087;-1.08	benign supporting
-0.248	[-0.387;-0.03	indeterminate
-0.610	[-0.697;-0.52	benign supporting
1.202	[-0.026;5.074	pathogenic moderate
0.587	[-0.180;1.132	pathogenic supporting
-1.141	[-1.148;-1.13	benign supporting
-0.283	[-0.304;-0.26	indeterminate
-0.062	[-0.388;0.808	indeterminate
-1.095	[-1.169;-1.06	benign supporting
1.255	[0.658;5.080]	pathogenic moderate
-0.360	[-0.383;-0.33	benign supporting
-0.260	[-0.273;-0.24	indeterminate
1.276	[1.125;1.558]	pathogenic strong
-1.062	[-3.588;0.000	benign supporting
0.986	[0.879;1.065]	pathogenic moderate
1.276	[1.219;1.345]	pathogenic strong
-1.152	[-1.170;-1.13	benign supporting
1.276	[1.226;1.335]	pathogenic strong
-1.097	[-1.099;-1.09	benign supporting
-1.110	[-1.117;-1.10	benign supporting
1.276	[1.205;1.370]	pathogenic strong
-1.090	[-1.115;-1.08	benign supporting
-0.332	[-0.662;0.551	benign supporting
-1.102	[-1.115;-1.08	benign supporting
1.276	[0.000;5.100]	pathogenic strong
-1.085	[-1.086;-1.08	benign supporting
-0.977	[-1.111;-0.58	benign supporting
0.328	[-1.107;4.992	pathogenic supporting
0.839	[0.680;1.007]	pathogenic moderate
-0.884	[-1.164;-0.05	benign supporting
-0.886	[-0.899;-0.87	benign supporting
1.276	[1.231;1.330]	pathogenic strong
-0.968	[-1.047;-0.87	benign supporting
-0.970	[-3.626;0.000	benign supporting
-0.267	[-0.294;-0.24	indeterminate
1.276	[-1.114;5.004	pathogenic strong
1.276	[-0.253;5.104	pathogenic strong
-1.086	[-1.092;-1.08	benign supporting
-0.627	[-0.634;-0.62	benign supporting
-1.069	[-1.112;-0.93	benign supporting
-0.615	[-0.950;-0.39	benign supporting
-1.087	[-1.089;-1.08	benign supporting

-1.006	[-1.016;-0.99	benign supporting
-0.882	[-0.958;-0.79	benign supporting
-0.849	[-3.754;0.000	benign supporting
1.176	[1.138;1.216	pathogenic moderate
-1.142	[-1.160;-1.12	benign supporting
0.399	[-0.146;0.891	pathogenic supporting
1.276	[-1.150;4.927	pathogenic strong
-0.983	[-1.165;-0.27	benign supporting
-0.253	[-0.381;-0.06	indeterminate
-0.443	[-0.584;-0.32	benign supporting
1.276	[-0.223;5.101	pathogenic strong
-1.114	[-1.133;-0.92	benign supporting
-0.521	[-0.561;-0.48	benign supporting
-1.062	[-1.142;-1.01	benign supporting
1.276	[1.012;2.063	pathogenic strong
-0.570	[-1.101;0.332	benign supporting
-1.078	[-1.085;-1.07	benign supporting
-0.951	[-0.953;-0.94	benign supporting
0.576	[0.508;0.636	pathogenic supporting
-0.016	[-0.332;0.767	indeterminate
-1.013	[-1.167;-0.91	benign supporting
1.116	[-0.726;5.064	pathogenic moderate
-1.084	[-1.168;-0.94	benign supporting
-1.084	[-1.087;-1.08	benign supporting
-1.087	[-1.091;-1.08	benign supporting
-1.162	[-1.169;-1.09	benign supporting
-0.506	[-0.583;-0.43	benign supporting
1.276	[1.272;1.280	pathogenic strong
-0.738	[-0.741;-0.73	benign supporting
1.158	[1.156;1.161	pathogenic moderate
-0.087	[-0.092;-0.08	indeterminate
1.276	[1.271;1.281	pathogenic strong
-0.421	[-0.423;-0.41	benign supporting
0.189	[0.166;0.213	indeterminate
-0.178	[-0.183;-0.17	indeterminate
0.521	[0.502;0.541	pathogenic supporting
1.276	[1.247;1.309	pathogenic strong
-0.493	[-0.495;-0.49	benign supporting
0.934	[0.929;0.939	pathogenic moderate
1.276	[1.114;1.629	pathogenic strong
-0.920	[-0.925;-0.91	benign supporting
1.276	[1.207;1.364	pathogenic strong
0.176	[0.145;0.206	indeterminate

1.276	[1.269;1.284	pathogenic strong
1.168	[1.165;1.170	pathogenic moderate
-0.166	[-0.249;-0.08	indeterminate
-0.837	[-0.839;-0.83	benign supporting
1.276	[-0.098;5.10	pathogenic strong
-1.087	[-1.168;-0.60	benign supporting
-1.086	[-1.110;-1.08	benign supporting
1.276	[-0.320;5.09	pathogenic strong
-0.791	[-1.110;-0.31	benign supporting
-1.063	[-1.164;-0.47	benign supporting
1.276	[0.682;5.088	pathogenic strong
1.162	[0.735;1.416	pathogenic moderate
-0.883	[-0.887;-0.87	benign supporting
-0.586	[-0.632;-0.53	benign supporting
1.224	[0.875;1.620	pathogenic moderate
0.029	[-0.251;0.69	indeterminate
-0.969	[-0.995;-0.95	benign supporting
-0.975	[-1.110;-0.60	benign supporting
1.214	[-0.242;5.09	pathogenic moderate
1.276	[0.000;5.104	pathogenic strong
-0.956	[-0.961;-0.95	benign supporting
-0.057	[-0.433;0.95	indeterminate
-1.095	[-1.115;-0.90	benign supporting
-0.602	[-0.939;-0.36	benign supporting
-1.098	[-1.112;-1.08	benign supporting
-1.166	[-1.169;-1.04	benign supporting
-1.046	[-1.112;-0.99	benign supporting
-0.568	[-0.658;-0.47	benign supporting
-0.999	[-1.167;-0.90	benign supporting
1.276	[-0.433;5.09	pathogenic strong
1.152	[1.131;1.176	pathogenic moderate
-0.830	[-1.059;-0.57	benign supporting
1.276	[0.000;5.102	pathogenic strong
-1.111	[-1.115;-1.07	benign supporting
1.276	[0.636;5.093	pathogenic strong
-0.897	[-0.964;-0.81	benign supporting
-0.351	[-0.570;-0.03	benign supporting
-0.340	[-0.488;-0.11	benign supporting
-0.094	[-0.235;0.17	indeterminate
-1.109	[-1.115;-1.05	benign supporting
-1.091	[-1.097;-1.08	benign supporting
-0.767	[-1.106;-0.34	benign supporting
-1.051	[-1.074;-1.01	benign supporting

-1.016	[-1.062;-0.96	benign supporting
-0.966	[-0.983;-0.95	benign supporting
-0.826	[-0.934;-0.68	benign supporting
-0.904	[-1.164;-0.00	benign supporting
-0.241	[-0.312;-0.14	indeterminate
-1.104	[-1.115;-1.08	benign supporting
1.276	[-0.282;5.10	pathogenic strong
-0.127	[-0.281;0.13	indeterminate
1.174	[1.104;1.253	pathogenic moderate
-0.170	[-0.253;-0.08	indeterminate
-1.034	[-1.103;-0.88	benign supporting
-1.049	[-1.076;-1.00	benign supporting
-1.108	[-1.169;-1.08	benign supporting
-0.935	[-0.956;-0.91	benign supporting
-1.086	[-1.093;-1.08	benign supporting
-1.087	[-1.101;-1.08	benign supporting
-0.484	[-0.504;-0.46	benign supporting
-1.071	[-1.085;-1.04	benign supporting
-0.653	[-0.679;-0.63	benign supporting
-1.119	[-1.169;-1.08	benign supporting
-0.636	[-0.710;-0.58	benign supporting
1.103	[0.969;1.154	pathogenic moderate
-1.012	[-1.061;-0.95	benign supporting
-1.107	[-1.115;-1.07	benign supporting
0.769	[0.595;0.933	pathogenic moderate
-1.114	[-1.132;-1.10	benign supporting
-1.009	[-1.023;-0.99	benign supporting
-1.000	[-1.047;-0.93	benign supporting
-0.518	[-0.562;-0.47	benign supporting
0.693	[0.040;1.063	pathogenic moderate
-0.954	[-1.111;-0.55	benign supporting
-1.026	[-1.047;-1.00	benign supporting
-0.655	[-0.680;-0.63	benign supporting
-1.110	[-1.118;-1.10	benign supporting
1.261	[-1.116;1.55	pathogenic moderate
-1.084	[-1.086;-1.08	benign supporting
-0.513	[-0.578;-0.44	benign supporting
-1.088	[-1.093;-1.08	benign supporting
-1.086	[-1.108;-1.08	benign supporting
-1.091	[-1.096;-1.08	benign supporting
-1.054	[-1.080;-1.03	benign supporting
-0.901	[-0.914;-0.88	benign supporting
-1.164	[-1.169;-1.12	benign supporting

-1.164	[-1.166;-1.16	benign supporting
-1.126	[-1.153;-1.10	benign supporting
-1.133	[-1.151;-1.11	benign supporting
-0.573	[-0.590;-0.55	benign supporting
-1.094	[-1.100;-1.09	benign supporting
1.276	[0.888;4.986]	pathogenic strong
-0.733	[-0.755;-0.71	benign supporting
-0.174	[-0.214;-0.13	indeterminate
-0.492	[-0.536;-0.44	benign supporting
-1.152	[-1.169;-1.13	benign supporting
-1.113	[-1.116;-1.10	benign supporting
-0.503	[-0.520;-0.48	benign supporting
-1.164	[-1.169;-1.11	benign supporting
-0.411	[-0.472;-0.35	benign supporting
-1.079	[-1.114;-0.82	benign supporting
0.064	[-0.073;0.347	indeterminate
-0.951	[-1.040;-0.84	benign supporting
-0.939	[-0.958;-0.92	benign supporting
-0.336	[-0.465;-0.13	benign supporting
-0.620	[-1.102;-0.06	benign supporting
-0.103	[-0.884;1.315	indeterminate
-1.070	[-1.081;-1.04	benign supporting
-1.112	[-1.115;-1.06	benign supporting
-1.118	[-1.167;-1.09	benign supporting
-1.035	[-1.071;-0.99	benign supporting
-1.164	[-1.169;-1.16	benign supporting
1.276	[-0.370;5.095	pathogenic strong
-0.140	[-0.285;0.048	indeterminate
-0.388	[-0.437;-0.33	benign supporting
0.726	[-0.969;4.987	pathogenic moderate
1.192	[1.148;1.240]	pathogenic moderate
-0.954	[-0.962;-0.94	benign supporting
-1.097	[-1.114;-1.08	benign supporting
-1.168	[-1.169;-1.09	benign supporting
-1.096	[-1.162;-1.08	benign supporting
-0.662	[-0.848;-0.55	benign supporting
-1.091	[-1.100;-1.08	benign supporting
-0.407	[-0.510;-0.31	benign supporting
-0.889	[-0.915;-0.86	benign supporting
-1.097	[-1.107;-1.08	benign supporting
-0.490	[-0.516;-0.46	benign supporting
-0.655	[-0.688;-0.62	benign supporting
-1.045	[-1.062;-1.02	benign supporting

-0.634	[-0.721;-0.57	benign supporting
-0.616	[-0.724;-0.52	benign supporting
-0.853	[-0.890;-0.81	benign supporting
-0.374	[-0.401;-0.34	benign supporting
-0.261	[-0.325;-0.17	indeterminate
-1.001	[-1.105;-0.75	benign supporting
-0.022	[-0.097;0.158	indeterminate
-0.998	[-1.030;-0.95	benign supporting
-0.747	[-0.794;-0.70	benign supporting
-0.891	[-0.938;-0.83	benign supporting
-1.087	[-1.092;-1.08	benign supporting
-1.067	[-1.095;-1.00	benign supporting
-1.100	[-1.115;-1.08	benign supporting
-0.658	[-1.029;-0.41	benign supporting
-0.837	[-0.897;-0.83	benign supporting
-0.346	[-1.164;4.906	benign supporting
0.180	[-0.278;0.868	indeterminate
-0.905	[-3.701;0.000	benign supporting
1.122	[-0.536;5.058	pathogenic moderate
1.242	[0.258;5.089]	pathogenic moderate
-0.719	[-1.115;-0.04	benign supporting
1.276	[1.250;1.304]	pathogenic strong
-0.729	[-0.956;-0.56	benign supporting
-1.110	[-1.125;-1.09	benign supporting
-1.043	[-1.070;-1.01	benign supporting
-0.474	[-0.858;-0.14	benign supporting
-0.771	[-0.812;-0.73	benign supporting
1.276	[0.446;5.086]	pathogenic strong
0.562	[-0.266;1.155	pathogenic supporting
0.314	[-1.072;4.928	indeterminate
-0.987	[-0.990;-0.98	benign supporting
-0.898	[-0.922;-0.87	benign supporting
-0.018	[-0.350;0.816	indeterminate
1.126	[1.058;1.167]	pathogenic moderate
1.189	[0.984;1.360]	pathogenic moderate
1.276	[1.247;1.308]	pathogenic strong
-1.108	[-1.169;-1.08	benign supporting
1.188	[1.084;1.297]	pathogenic moderate
-0.646	[-1.147;0.871	benign supporting
-0.448	[-0.629;-0.29	benign supporting
-1.101	[-1.115;-1.08	benign supporting
-1.106	[-1.115;-1.09	benign supporting
-0.630	[-1.067;-0.32	benign supporting

-0.977	[-0.980;-0.97	benign supporting
0.167	[-0.160;0.657	indeterminate
1.021	[0.301;1.273	pathogenic moderate
1.276	[1.115;1.630	pathogenic strong
-0.872	[-0.917;-0.85	benign supporting
-1.085	[-1.096;-1.08	benign supporting
1.276	[1.243;1.313	pathogenic strong
1.276	[1.233;1.326	pathogenic strong
1.276	[1.233;1.324	pathogenic strong
0.608	[0.322;0.820	pathogenic supporting
0.504	[-0.320;1.173	pathogenic supporting
-0.465	[-0.959;0.023	benign supporting
-0.986	[-0.997;-0.97	benign supporting
0.203	[-0.292;0.917	indeterminate
-1.067	[-1.072;-1.06	benign supporting
-0.339	[-1.156;4.949	benign supporting
1.177	[0.178;4.070	pathogenic moderate
1.276	[1.155;1.473	pathogenic strong
-1.007	[-1.022;-0.99	benign supporting
-0.459	[-1.158;1.954	benign supporting
-1.145	[-1.169;-1.11	benign supporting
1.201	[-0.519;5.084	pathogenic moderate
-0.421	[-0.761;-0.03	benign supporting
-0.269	[-0.494;0.344	indeterminate
1.201	[1.105;1.318	pathogenic moderate
-0.933	[-1.111;-0.50	benign supporting
1.276	[-0.219;5.100	pathogenic strong
1.276	[-0.086;5.105	pathogenic strong
1.276	[1.065;1.797	pathogenic strong
1.087	[0.780;1.203	pathogenic moderate
1.183	[1.058;1.297	pathogenic moderate
-0.247	[-0.922;1.173	indeterminate
-0.422	[-0.943;0.422	benign supporting
1.276	[1.240;1.318	pathogenic strong
1.276	[1.240;1.318	pathogenic strong
0.530	[-0.525;1.422	pathogenic supporting
-1.101	[-1.165;-1.08	benign supporting
-0.673	[-1.097;-0.30	benign supporting
1.276	[0.000;5.102	pathogenic strong
-1.100	[-1.103;-1.09	benign supporting
1.263	[1.006;1.830	pathogenic moderate
-0.849	[-0.866;-0.83	benign supporting
1.239	[1.177;1.311	pathogenic moderate

-0.971	[-0.975;-0.96	benign supporting
1.276	[1.246;1.309]	pathogenic strong
-0.982	[-1.166;0.758	benign supporting
-0.937	[-0.951;-0.92	benign supporting
0.403	[0.345;0.459]	pathogenic supporting
-0.873	[-0.886;-0.85	benign supporting
-1.088	[-1.168;-0.52	benign supporting
1.101	[0.921;1.163]	pathogenic moderate
0.565	[0.514;0.608]	pathogenic supporting
-0.479	[-1.076;0.433	benign supporting
1.276	[1.201;1.377]	pathogenic strong
-1.107	[-1.119;-1.09	benign supporting
-1.010	[-1.016;-1.00	benign supporting
1.276	[-0.100;5.102	pathogenic strong
1.276	[1.227;1.336]	pathogenic strong
-0.967	[-3.647;0.000	benign supporting
1.276	[1.225;1.340]	pathogenic strong
-0.698	[-0.796;-0.63	benign supporting
1.276	[1.238;1.319]	pathogenic strong
1.276	[1.224;1.338]	pathogenic strong
1.276	[1.170;1.436]	pathogenic strong
1.276	[1.170;1.436]	pathogenic strong
1.250	[-0.195;5.100	pathogenic moderate
1.276	[1.187;1.401]	pathogenic strong
1.276	[0.993;2.208]	pathogenic strong
1.276	[1.191;1.393]	pathogenic strong
1.276	[1.212;1.357]	pathogenic strong
1.276	[1.106;1.647]	pathogenic strong
1.182	[0.856;1.404]	pathogenic moderate
1.276	[1.252;1.302]	pathogenic strong
1.276	[1.163;1.448]	pathogenic strong
1.276	[1.107;1.640]	pathogenic strong
-0.632	[-0.652;-0.61	benign supporting
1.119	[0.658;1.301]	pathogenic moderate
1.276	[0.362;5.104]	pathogenic strong
1.276	[1.214;1.355]	pathogenic strong
-1.050	[-3.580;0.000	benign supporting
-0.977	[-0.981;-0.97	benign supporting
-1.003	[-1.148;-0.93	benign supporting
-1.100	[-1.109;-1.09	benign supporting
1.238	[0.987;1.591]	pathogenic moderate
-1.164	[-1.169;-0.92	benign supporting
1.265	[1.238;1.295]	pathogenic moderate

-0.292	[-0.334;-0.24	indeterminate
-0.096	[-0.275;0.322	indeterminate
-0.438	[-0.580;-0.32	benign supporting
0.336	[-0.283;1.042	pathogenic supporting
1.276	[1.188;1.402	pathogenic strong
1.191	[-0.365;5.087	pathogenic moderate
-1.084	[-1.086;-1.08	benign supporting
-0.976	[-3.501;0.000	benign supporting
-0.923	[-3.534;-0.83	benign supporting
-0.986	[-1.009;-0.95	benign supporting
-0.424	[-0.490;-0.37	benign supporting
-0.611	[-0.620;-0.60	benign supporting
-1.117	[-1.158;-1.09	benign supporting
-0.462	[-0.471;-0.45	benign supporting
-1.151	[-1.170;-1.12	benign supporting
-1.100	[-1.108;-1.09	benign supporting
-1.084	[-1.086;-1.08	benign supporting
-0.674	[-0.691;-0.65	benign supporting
-0.233	[-0.301;-0.14	indeterminate
-1.038	[-1.054;-1.02	benign supporting
-0.449	[-0.461;-0.43	benign supporting
1.276	[1.233;1.326	pathogenic strong
-1.087	[-1.099;-1.07	benign supporting
0.851	[0.773;0.935	pathogenic moderate
1.168	[1.045;1.269	pathogenic moderate
-1.145	[-1.158;-1.13	benign supporting
-0.387	[-0.398;-0.37	benign supporting
-0.381	[-0.393;-0.36	benign supporting
-1.127	[-1.134;-1.12	benign supporting
-1.091	[-1.110;-1.07	benign supporting
-1.094	[-1.114;-1.07	benign supporting
-0.892	[-0.895;-0.88	benign supporting
-0.366	[-0.374;-0.35	benign supporting
1.099	[1.008;1.136	pathogenic moderate
-1.100	[-1.103;-1.09	benign supporting
-0.330	[-0.487;-0.08	benign supporting
-1.095	[-1.103;-1.08	benign supporting
-1.069	[-1.076;-1.05	benign supporting
-1.114	[-1.168;-1.08	benign supporting
-0.974	[-1.007;-0.95	benign supporting
0.980	[0.917;1.028	pathogenic moderate
-0.904	[-0.908;-0.90	benign supporting
-0.366	[-0.558;-0.10	benign supporting

-0.955	[-1.147;-0.36	benign supporting
-0.264	[-0.444;0.153	indeterminate
1.229	[1.205;1.255]	pathogenic moderate
-1.062	[-1.067;-1.05	benign supporting
0.372	[0.188;0.524]	pathogenic supporting
-1.140	[-1.159;-1.12	benign supporting
-1.085	[-1.168;-0.92	benign supporting
-1.094	[-1.115;-1.08	benign supporting
1.276	[1.153;1.486]	pathogenic strong
-0.137	[-0.380;0.487	indeterminate
-1.049	[-1.055;-1.04	benign supporting
-1.100	[-1.115;-0.96	benign supporting
-0.887	[-0.889;-0.88	benign supporting
1.276	[1.225;1.338]	pathogenic strong
-0.802	[-1.030;-0.57	benign supporting
-0.624	[-0.637;-0.61	benign supporting
-0.956	[-0.966;-0.94	benign supporting
1.276	[1.158;1.459]	pathogenic strong
1.184	[1.113;1.264]	pathogenic moderate
0.656	[-0.567;1.768	pathogenic moderate
-0.183	[-0.202;-0.16	indeterminate
1.276	[-0.492;5.094	pathogenic strong
1.157	[1.137;1.179]	pathogenic moderate
1.276	[1.219;1.344]	pathogenic strong
-0.648	[-2.208;4.863	benign supporting
-0.368	[-0.441;-0.28	benign supporting
-1.089	[-1.165;-1.08	benign supporting
-1.109	[-1.115;-1.06	benign supporting
0.930	[0.820;1.030]	pathogenic moderate
-1.064	[-1.068;-1.05	benign supporting
-1.086	[-1.092;-1.08	benign supporting
1.276	[-0.429;5.097	pathogenic strong
-1.074	[-1.089;-1.05	benign supporting
0.594	[-0.078;1.039	pathogenic supporting
-1.108	[-1.115;-1.09	benign supporting
0.630	[0.016;1.001]	pathogenic supporting
0.099	[-0.103;0.466	indeterminate
1.276	[1.208;1.361]	pathogenic strong
-1.043	[-1.088;-0.95	benign supporting
-0.412	[-0.621;-0.17	benign supporting
-1.068	[-1.115;-0.63	benign supporting
1.276	[1.089;1.703]	pathogenic strong
-1.020	[-1.089;-0.89	benign supporting

0.761	[0.635;0.872]	pathogenic moderate
-0.869	[-0.955;-0.76	benign supporting
-1.090	[-1.114;-0.96	benign supporting
-0.006	[-1.166;4.731	indeterminate
-0.683	[-0.706;-0.66	benign supporting
-0.984	[-1.005;-0.96	benign supporting
-0.331	[-0.376;-0.27	benign supporting
-0.994	[-3.398;1.818	benign supporting
-0.398	[-0.448;-0.34	benign supporting
-0.657	[-0.735;-0.60	benign supporting
-0.281	[-0.312;-0.24	indeterminate
-1.017	[-1.032;-1.00	benign supporting
-0.356	[-0.395;-0.31	benign supporting
-0.315	[-0.778;0.826	indeterminate
-0.819	[-0.868;-0.74	benign supporting
-0.554	[-0.580;-0.52	benign supporting
-0.311	[-0.333;-0.28	indeterminate
-0.462	[-0.505;-0.42	benign supporting
-1.067	[-1.088;-1.01	benign supporting
0.731	[0.659;0.798]	pathogenic moderate
-1.164	[-1.165;-1.16	benign supporting
0.919	[0.844;0.998]	pathogenic moderate
-0.909	[-0.928;-0.88	benign supporting
-0.672	[-0.764;-0.61	benign supporting
-0.519	[-0.574;-0.46	benign supporting
-1.006	[-1.012;-1.00	benign supporting
-0.677	[-0.691;-0.66	benign supporting
-0.216	[-0.246;-0.18	indeterminate
-1.133	[-1.168;-1.10	benign supporting
-1.044	[-1.051;-1.03	benign supporting
-0.791	[-0.809;-0.77	benign supporting
-0.549	[-0.556;-0.54	benign supporting
-0.422	[-0.470;-0.38	benign supporting
-0.632	[-0.646;-0.61	benign supporting
-0.317	[-0.344;-0.28	indeterminate
-0.696	[-0.716;-0.68	benign supporting
-0.901	[-0.914;-0.88	benign supporting
1.276	[1.162;1.454]	pathogenic strong
-0.510	[-0.525;-0.49	benign supporting
-0.745	[-0.831;-0.67	benign supporting
1.254	[0.014;5.098]	pathogenic moderate
-1.053	[-1.091;-0.96	benign supporting
-0.442	[-0.451;-0.43	benign supporting

1.276	[1.250;1.305]	pathogenic strong
-0.445	[-0.450;-0.44	benign supporting
-1.048	[-1.077;-1.00	benign supporting
-1.080	[-1.166;-0.30	benign supporting
-0.509	[-0.582;-0.43	benign supporting
-0.872	[-0.946;-0.77	benign supporting
-0.907	[-1.020;-0.74	benign supporting
-0.300	[-0.355;-0.22	indeterminate
-0.264	[-0.373;-0.10	indeterminate
1.103	[0.520;1.304]	pathogenic moderate
1.192	[1.140;1.249]	pathogenic moderate
1.276	[1.223;1.342]	pathogenic strong
-0.473	[-0.578;-0.38	benign supporting
-0.252	[-0.316;-0.17	indeterminate
1.276	[1.216;1.354]	pathogenic strong
0.000	[0.000;0.000]	indeterminate
1.276	[1.187;1.403]	pathogenic strong
-0.913	[-1.065;-0.68	benign supporting
0.424	[-0.246;1.050	pathogenic supporting
-0.470	[-1.106;1.108	benign supporting
-0.150	[-0.219;-0.08	indeterminate
1.047	[0.932;1.119]	pathogenic moderate
-0.161	[-0.483;0.778	indeterminate
-0.921	[-0.946;-0.89	benign supporting
-1.014	[-1.070;-0.93	benign supporting
-1.093	[-1.103;-1.08	benign supporting
-0.183	[-0.239;-0.12	indeterminate
-1.108	[-1.115;-1.09	benign supporting
-1.071	[-1.082;-1.05	benign supporting
-0.491	[-0.523;-0.45	benign supporting
-1.087	[-1.092;-1.08	benign supporting
-1.097	[-1.103;-1.09	benign supporting
-0.878	[-1.009;-0.70	benign supporting
-1.134	[-1.169;-1.10	benign supporting
-0.832	[-0.901;-0.73	benign supporting
-0.619	[-0.655;-0.58	benign supporting
1.276	[1.131;1.567]	pathogenic strong
-1.029	[-1.091;-0.91	benign supporting
-0.307	[-0.331;-0.27	indeterminate
-0.876	[-0.964;-0.76	benign supporting
0.916	[0.778;1.047]	pathogenic moderate
-0.837	[-0.839;-0.83	benign supporting
-0.691	[-0.806;-0.62	benign supporting

-0.823	[-0.838;-0.79	benign supporting
-0.935	[-0.960;-0.91	benign supporting
-1.086	[-1.090;-1.08	benign supporting
-1.102	[-1.105;-1.10	benign supporting
-1.090	[-1.114;-1.06	benign supporting
-0.577	[-0.587;-0.56	benign supporting
-1.127	[-1.169;-1.08	benign supporting
-0.599	[-0.635;-0.55	benign supporting
-0.614	[-0.624;-0.60	benign supporting
-1.089	[-1.096;-1.08	benign supporting
-0.923	[-0.947;-0.89	benign supporting
-0.694	[-0.728;-0.66	benign supporting
-0.612	[-0.626;-0.59	benign supporting
-1.107	[-1.111;-1.10	benign supporting
1.276	[1.202;1.374]	pathogenic strong
-1.091	[-1.099;-1.08	benign supporting
0.190	[-0.161;0.683	indeterminate
-0.927	[-0.953;-0.89	benign supporting
-0.520	[-0.533;-0.50	benign supporting
-1.094	[-1.096;-1.09	benign supporting
-1.065	[-1.089;-1.00	benign supporting
-1.099	[-1.105;-1.09	benign supporting
-0.736	[-0.875;-0.63	benign supporting
-0.736	[-0.885;-0.62	benign supporting
-0.415	[-0.475;-0.36	benign supporting
-1.091	[-1.114;-0.92	benign supporting
-1.102	[-1.114;-1.09	benign supporting
-1.123	[-3.450;1.091	benign supporting
-0.881	[-0.965;-0.77	benign supporting
-0.607	[-0.834;-0.43	benign supporting
1.276	[-0.520;5.094	pathogenic strong
-0.690	[-1.009;-0.46	benign supporting
-0.426	[-0.471;-0.38	benign supporting
-1.085	[-1.095;-1.08	benign supporting
-1.085	[-1.090;-1.08	benign supporting
1.232	[-0.341;5.092	pathogenic moderate
-0.911	[-0.988;-0.82	benign supporting
1.276	[1.216;1.348]	pathogenic strong
1.276	[0.000;5.108]	pathogenic strong
-0.955	[-0.963;-0.94	benign supporting
1.276	[1.117;1.595]	pathogenic strong
-0.669	[-0.724;-0.62	benign supporting
-0.863	[-0.933;-0.77	benign supporting

-0.025	[-0.191;0.418	indeterminate
1.254	[1.152;1.401]	pathogenic moderate
1.276	[1.245;1.312]	pathogenic strong
1.260	[1.227;1.294]	pathogenic moderate
1.276	[1.253;1.301]	pathogenic strong
1.276	[1.164;1.456]	pathogenic strong
1.188	[1.168;1.208]	pathogenic moderate
1.276	[1.216;1.349]	pathogenic strong
1.276	[1.136;1.549]	pathogenic strong
1.276	[1.230;1.333]	pathogenic strong
0.937	[0.374;1.191]	pathogenic moderate
1.276	[1.235;1.324]	pathogenic strong
1.157	[1.122;1.196]	pathogenic moderate
1.276	[1.187;1.398]	pathogenic strong
-0.400	[-0.415;-0.38	benign supporting
1.118	[0.997;1.178]	pathogenic moderate
1.224	[1.187;1.262]	pathogenic moderate
1.276	[1.251;1.303]	pathogenic strong
1.276	[1.211;1.359]	pathogenic strong
0.951	[0.894;1.005]	pathogenic moderate
-1.075	[-1.113;-0.96	benign supporting
-1.097	[-1.114;-1.08	benign supporting
-0.913	[-0.932;-0.88	benign supporting
-0.725	[-0.770;-0.68	benign supporting
-0.833	[-0.861;-0.79	benign supporting
-0.341	[-0.367;-0.31	benign supporting
-1.094	[-1.110;-1.08	benign supporting
-0.668	[-0.722;-0.62	benign supporting
-1.084	[-1.088;-1.08	benign supporting
0.757	[0.704;0.808]	pathogenic moderate
-0.525	[-0.548;-0.50	benign supporting
-0.579	[-0.621;-0.53	benign supporting
-0.875	[-0.903;-0.84	benign supporting
-0.158	[-0.258;-0.05	indeterminate
-1.018	[-1.053;-0.98	benign supporting
1.276	[1.246;1.309]	pathogenic strong
-0.589	[-0.829;-0.42	benign supporting
-1.081	[-1.085;-1.07	benign supporting
-0.960	[-0.995;-0.93	benign supporting
-1.081	[-1.089;-1.07	benign supporting
-1.112	[-1.116;-1.10	benign supporting
-1.037	[-1.107;-0.87	benign supporting
-0.399	[-0.481;-0.32	benign supporting

1.276	[1.159;1.453	pathogenic strong
1.131	[0.826;1.265	pathogenic moderate
-0.535	[-0.589;-0.47	benign supporting
1.276	[1.106;1.680	pathogenic strong
0.631	[0.509;0.733	pathogenic supporting
1.276	[1.157;1.460	pathogenic strong
1.276	[1.202;1.373	pathogenic strong
-0.112	[-0.238;0.071	indeterminate
-0.599	[-0.638;-0.55	benign supporting
-1.081	[-1.091;-1.07	benign supporting
1.276	[1.223;1.340	pathogenic strong
1.276	[0.852;5.048	pathogenic strong
-0.137	[-0.182;-0.09	indeterminate
-0.122	[-0.462;0.827	indeterminate
-0.759	[-0.849;-0.67	benign supporting
1.140	[1.126;1.155	pathogenic moderate
0.062	[-0.042;0.271	indeterminate
-1.060	[-1.092;-0.98	benign supporting
1.276	[1.176;1.426	pathogenic strong
1.276	[1.214;1.355	pathogenic strong
1.090	[0.997;1.134	pathogenic moderate
1.276	[1.250;1.304	pathogenic strong
1.276	[1.242;1.314	pathogenic strong
1.276	[1.205;1.365	pathogenic strong
1.276	[1.181;1.412	pathogenic strong
1.242	[1.215;1.270	pathogenic moderate
1.276	[1.163;1.451	pathogenic strong
1.130	[1.105;1.155	pathogenic moderate
1.276	[1.134;1.546	pathogenic strong
1.276	[1.211;1.361	pathogenic strong
1.276	[1.234;1.326	pathogenic strong
1.276	[1.194;1.389	pathogenic strong
1.276	[1.231;1.327	pathogenic strong
0.985	[0.600;1.174	pathogenic moderate
1.276	[1.140;1.528	pathogenic strong
1.276	[1.253;1.300	pathogenic strong
1.276	[1.253;1.302	pathogenic strong
1.276	[1.135;1.543	pathogenic strong
-1.085	[-1.098;-1.08	benign supporting
-0.062	[-0.173;0.195	indeterminate
1.276	[0.743;5.081	pathogenic strong
1.276	[1.120;1.581	pathogenic strong
1.121	[0.843;1.235	pathogenic moderate

-1.031	[-1.166;-0.11	benign supporting
1.276	[0.026;5.102	pathogenic strong
0.507	[-0.505;1.379	pathogenic supporting
1.276	[0.634;5.085	pathogenic strong
1.276	[-1.123;5.037	pathogenic strong
0.349	[-0.653;1.485	pathogenic supporting
1.276	[0.000;5.107	pathogenic strong
-1.091	[-2.939;1.352	benign supporting
1.276	[-0.217;5.102	pathogenic strong
1.051	[0.893;1.135	pathogenic moderate
1.276	[1.177;1.425	pathogenic strong
-0.343	[-0.598;0.177	benign supporting
-0.358	[-0.367;-0.34	benign supporting
0.799	[0.544;1.027	pathogenic moderate
-0.801	[-1.048;-0.56	benign supporting
0.962	[0.849;1.056	pathogenic moderate
1.240	[1.102;1.453	pathogenic moderate
-1.142	[-1.169;-1.10	benign supporting
1.276	[1.153;1.484	pathogenic strong
0.635	[0.604;0.666	pathogenic supporting
1.276	[1.258;1.296	pathogenic strong
1.276	[0.837;5.061	pathogenic strong
0.966	[0.444;1.201	pathogenic moderate
1.178	[1.096;1.269	pathogenic moderate
1.276	[1.195;1.384	pathogenic strong
-0.369	[-0.421;-0.30	benign supporting
1.065	[-0.196;1.652	pathogenic moderate
-1.113	[-1.165;-0.83	benign supporting
1.276	[1.238;1.321	pathogenic strong
1.143	[-0.260;5.039	pathogenic moderate
0.756	[0.726;0.788	pathogenic moderate
-0.339	[-0.421;-0.22	benign supporting
-0.221	[-0.305;-0.11	indeterminate
-0.985	[-1.029;-0.95	benign supporting
-1.093	[-1.167;-0.54	benign supporting
0.995	[-0.341;2.112	pathogenic moderate
-1.084	[-1.168;-0.99	benign supporting
0.553	[-1.150;5.018	pathogenic supporting
1.276	[0.000;5.106	pathogenic strong
1.276	[1.222;1.342	pathogenic strong
1.276	[1.139;1.530	pathogenic strong
1.276	[1.193;1.387	pathogenic strong
1.276	[1.192;1.391	pathogenic strong

1.276	[1.042;1.881	pathogenic strong
-0.965	[-0.988;-0.94	benign supporting
1.276	[-0.175;5.104	pathogenic strong
1.276	[0.905;5.033	pathogenic strong
-1.155	[-1.161;-1.14	benign supporting
-1.121	[-1.130;-1.11	benign supporting
-1.036	[-1.053;-1.02	benign supporting
0.931	[-0.209;1.430	pathogenic moderate
1.276	[1.094;1.702	pathogenic strong
1.276	[0.411;5.096	pathogenic strong
-1.165	[-1.168;-1.16	benign supporting
1.276	[-0.241;5.109	pathogenic strong
1.276	[0.649;5.098	pathogenic strong
1.276	[-0.245;5.102	pathogenic strong
1.276	[1.077;1.727	pathogenic strong
1.276	[1.139;1.529	pathogenic strong
1.276	[1.165;1.446	pathogenic strong
1.219	[1.058;1.400	pathogenic moderate
0.550	[0.452;0.628	pathogenic supporting
1.276	[0.922;5.025	pathogenic strong
-1.087	[-1.164;-0.63	benign supporting
1.276	[0.983;2.618	pathogenic strong
1.276	[1.207;1.367	pathogenic strong
1.276	[1.231;1.329	pathogenic strong
0.127	[-1.029;2.224	indeterminate
1.276	[1.054;1.851	pathogenic strong
-0.688	[-1.091;-0.35	benign supporting
-0.014	[-0.285;0.677	indeterminate
1.276	[1.229;1.333	pathogenic strong
1.276	[1.181;1.408	pathogenic strong
1.276	[1.232;1.327	pathogenic strong
1.276	[0.487;5.092	pathogenic strong
1.276	[1.198;1.381	pathogenic strong
1.115	[0.818;1.229	pathogenic moderate
1.276	[1.227;1.336	pathogenic strong
1.276	[1.104;1.688	pathogenic strong
1.079	[0.762;1.200	pathogenic moderate
1.276	[1.110;1.660	pathogenic strong
1.276	[-0.198;5.104	pathogenic strong
1.276	[0.000;5.109	pathogenic strong
1.276	[0.791;5.079	pathogenic strong
1.276	[1.223;1.342	pathogenic strong
0.889	[0.031;1.220	pathogenic moderate

1.276	[1.060;1.832	pathogenic strong
1.149	[1.135;1.163	pathogenic moderate
1.276	[1.246;1.309	pathogenic strong
0.690	[0.468;0.852	pathogenic moderate
-0.345	[-0.497;-0.11	benign supporting
-0.389	[-0.609;-0.11	benign supporting
1.276	[1.246;1.310	pathogenic strong
1.276	[1.057;1.837	pathogenic strong
1.276	[1.205;1.369	pathogenic strong
-0.948	[-1.123;-0.38	benign supporting
1.276	[1.231;1.330	pathogenic strong
-0.071	[-0.129;0.004	indeterminate
-1.085	[-1.087;-1.08	benign supporting
0.748	[0.593;0.882	pathogenic moderate
1.276	[1.099;1.713	pathogenic strong
1.276	[1.223;1.342	pathogenic strong
1.177	[0.921;1.345	pathogenic moderate
0.858	[0.507;1.120	pathogenic moderate
-0.416	[-0.621;-0.21	benign supporting
1.276	[0.862;5.051	pathogenic strong
-0.305	[-0.332;-0.27	indeterminate
1.276	[1.238;1.320	pathogenic strong
0.705	[0.491;0.870	pathogenic moderate
1.197	[1.090;1.316	pathogenic moderate
-1.047	[-1.055;-1.04	benign supporting
-1.121	[-1.169;-0.44	benign supporting
-1.090	[-1.167;-0.58	benign supporting
1.276	[-0.303;5.107	pathogenic strong
-1.057	[-1.072;-1.04	benign supporting
-0.948	[-0.950;-0.94	benign supporting
1.276	[1.150;1.476	pathogenic strong
0.760	[0.257;1.106	pathogenic moderate
1.276	[-0.125;5.102	pathogenic strong
1.276	[1.248;1.307	pathogenic strong
-1.093	[-1.105;-1.08	benign supporting
1.276	[1.241;1.316	pathogenic strong
-0.784	[-1.153;0.443	benign supporting
1.276	[1.191;1.394	pathogenic strong
1.276	[1.106;1.669	pathogenic strong
1.107	[0.648;1.280	pathogenic moderate
-1.104	[-1.104;-1.10	benign supporting
-0.788	[-0.802;-0.77	benign supporting
1.276	[1.268;1.285	pathogenic strong

1.276	[1.265;1.288	pathogenic strong
1.276	[1.197;1.380	pathogenic strong
-0.636	[-0.637;-0.63	benign supporting
1.276	[1.269;1.283	pathogenic strong
1.276	[1.273;1.279	pathogenic strong
1.276	[1.271;1.282	pathogenic strong
1.026	[1.017;1.035	pathogenic moderate
-0.726	[-0.739;-0.71	benign supporting
1.276	[1.270;1.283	pathogenic strong
-0.309	[-0.313;-0.30	indeterminate
1.276	[1.271;1.281	pathogenic strong
1.276	[1.220;1.345	pathogenic strong
-0.264	[-0.296;-0.23	indeterminate
1.276	[1.272;1.281	pathogenic strong
1.166	[1.158;1.174	pathogenic moderate
1.276	[1.275;1.278	pathogenic strong
1.276	[1.273;1.280	pathogenic strong
-0.617	[-0.623;-0.61	benign supporting
-1.077	[-1.114;-0.90	benign supporting
-0.968	[-1.065;-0.85	benign supporting
-0.236	[-0.423;0.241	indeterminate
1.276	[0.496;5.096	pathogenic strong
-1.048	[-1.065;-1.03	benign supporting
-0.819	[-0.870;-0.74	benign supporting
1.276	[1.203;1.371	pathogenic strong
-0.967	[-1.004;-0.93	benign supporting
1.276	[1.193;1.391	pathogenic strong
-1.076	[-1.114;-0.90	benign supporting
-1.088	[-1.100;-1.07	benign supporting
-1.056	[-1.069;-1.03	benign supporting
1.276	[0.435;5.102	pathogenic strong
-0.999	[-1.091;-0.82	benign supporting
-0.401	[-1.107;1.247	benign supporting
-1.084	[-1.166;-1.08	benign supporting
1.276	[0.915;4.968	pathogenic strong
0.209	[0.026;0.356	indeterminate
0.006	[-0.379;0.958	indeterminate
-0.191	[-0.350;0.063	indeterminate
0.826	[0.426;1.114	pathogenic moderate
1.276	[0.751;5.081	pathogenic strong
-0.939	[-0.942;-0.93	benign supporting
-1.056	[-1.067;-1.04	benign supporting
1.276	[1.002;2.079	pathogenic strong

0.645	[-0.566;1.64	pathogenic moderate
-1.141	[-3.443;0.00	benign supporting
-1.164	[-1.169;-1.10	benign supporting
-0.839	[-3.762;0.00	benign supporting
0.564	[-0.367;1.23	pathogenic supporting
1.276	[1.245;1.312	pathogenic strong
0.901	[0.496;1.143	pathogenic moderate
-3.347	[-3.805;0.00	benign strong
0.538	[0.367;0.677	pathogenic supporting
1.276	[1.127;1.569	pathogenic strong
-0.641	[-0.841;-0.51	benign supporting
-0.556	[-0.803;-0.36	benign supporting
1.276	[1.059;1.850	pathogenic strong
1.276	[1.182;1.409	pathogenic strong
1.276	[0.000;5.105	pathogenic strong
-0.828	[-0.865;-0.77	benign supporting
1.276	[1.198;1.382	pathogenic strong
-1.109	[-1.115;-1.06	benign supporting
-0.418	[-0.612;-0.22	benign supporting
-1.164	[-1.166;-1.16	benign supporting
1.188	[0.801;1.467	pathogenic moderate
1.276	[1.258;1.294	pathogenic strong
1.085	[0.935;1.146	pathogenic moderate
1.276	[1.006;2.065	pathogenic strong
1.276	[1.249;1.306	pathogenic strong
1.276	[0.892;5.002	pathogenic strong
1.276	[0.600;5.094	pathogenic strong
1.172	[1.036;1.284	pathogenic moderate
-0.649	[-0.692;-0.61	benign supporting
1.276	[1.253;1.302	pathogenic strong
1.276	[1.254;1.300	pathogenic strong
1.276	[1.179;1.416	pathogenic strong
0.927	[0.604;1.138	pathogenic moderate
1.154	[0.747;1.366	pathogenic moderate
1.276	[1.238;1.318	pathogenic strong
1.276	[1.256;1.299	pathogenic strong
1.276	[1.244;1.314	pathogenic strong
-0.352	[-0.518;-0.11	benign supporting
-1.108	[-1.168;-1.04	benign supporting
-1.079	[-1.136;-0.65	benign supporting
-0.598	[-1.085;-0.17	benign supporting
1.276	[1.221;1.345	pathogenic strong
1.276	[1.129;1.551	pathogenic strong

-0.852	[-1.101;-0.49	benign supporting
-0.853	[-0.925;-0.84	benign supporting
0.672	[0.454;0.833]	pathogenic moderate
1.276	[0.487;5.096]	pathogenic strong
-0.510	[-0.840;-0.25	benign supporting
1.276	[1.211;1.358]	pathogenic strong
1.276	[1.232;1.327]	pathogenic strong
1.276	[1.195;1.390]	pathogenic strong
0.000	[0.000;0.000]	indeterminate
0.392	[-0.208;0.977	pathogenic supporting
-0.902	[-1.078;-0.63	benign supporting
1.276	[1.125;1.591]	pathogenic strong
-0.845	[-1.074;-0.57	benign supporting
-1.090	[-1.094;-1.08	benign supporting
0.181	[-0.273;0.865	indeterminate
-0.338	[-0.568;0.083	benign supporting
1.235	[1.194;1.278]	pathogenic moderate
-0.271	[-0.314;-0.22	indeterminate
0.071	[-0.048;0.302	indeterminate
1.276	[1.188;1.396]	pathogenic strong
1.276	[1.236;1.323]	pathogenic strong
0.628	[-0.260;1.200	pathogenic supporting
0.672	[0.353;0.902]	pathogenic moderate
0.048	[-0.295;0.804	indeterminate
1.276	[1.052;1.854]	pathogenic strong
-1.096	[-1.104;-1.09	benign supporting
0.834	[0.371;1.131]	pathogenic moderate
1.276	[1.239;1.317]	pathogenic strong
-0.429	[-0.615;-0.25	benign supporting
1.178	[0.998;1.315]	pathogenic moderate
1.276	[1.210;1.356]	pathogenic strong
1.276	[1.015;2.163]	pathogenic strong
-0.115	[-0.360;0.530	indeterminate
-1.017	[-1.054;-0.98	benign supporting
-0.814	[-0.985;-0.62	benign supporting
-1.065	[-1.074;-1.03	benign supporting
-0.420	[-0.454;-0.38	benign supporting
-1.087	[-1.091;-1.08	benign supporting
-1.085	[-1.112;-1.08	benign supporting
-0.936	[-0.947;-0.92	benign supporting
-1.016	[-1.023;-1.00	benign supporting
-1.166	[-1.169;-1.15	benign supporting
-1.039	[-1.070;-1.00	benign supporting

1.276	[1.149;1.492	pathogenic strong
1.145	[1.129;1.161	pathogenic moderate
1.276	[-0.034;5.104	pathogenic strong
0.318	[-0.082;0.688	pathogenic supporting
-0.766	[-1.061;-0.51	benign supporting
1.136	[1.116;1.155	pathogenic moderate
-0.564	[-0.581;-0.54	benign supporting
-1.048	[-1.052;-1.04	benign supporting
1.276	[0.842;5.057	pathogenic strong
-1.095	[-1.101;-1.09	benign supporting
-1.097	[-1.114;-1.08	benign supporting
1.276	[0.979;2.124	pathogenic strong
1.167	[1.012;1.281	pathogenic moderate
0.439	[-0.414;1.232	pathogenic supporting
-1.166	[-1.170;-1.12	benign supporting
1.276	[1.226;1.337	pathogenic strong
1.276	[1.117;1.596	pathogenic strong
1.276	[1.189;1.397	pathogenic strong
-0.676	[-0.916;-0.53	benign supporting
1.253	[1.191;1.326	pathogenic moderate
1.276	[0.108;5.104	pathogenic strong
-1.057	[-1.060;-1.05	benign supporting
1.276	[1.043;1.895	pathogenic strong
-0.964	[-1.013;-0.91	benign supporting
0.974	[0.470;1.197	pathogenic moderate
1.276	[-0.049;5.106	pathogenic strong
-0.376	[-0.523;-0.18	benign supporting
-1.084	[-1.088;-1.08	benign supporting
-0.359	[-0.417;-0.28	benign supporting
-0.257	[-0.421;0.055	indeterminate
0.377	[-1.153;5.015	pathogenic supporting
-1.047	[-1.056;-1.03	benign supporting
-0.558	[-0.707;-0.41	benign supporting
-1.087	[-1.167;-0.95	benign supporting
1.276	[0.981;2.247	pathogenic strong
-1.086	[-1.088;-1.08	benign supporting
0.718	[-0.296;1.281	pathogenic moderate
1.276	[1.036;1.929	pathogenic strong
-1.057	[-1.112;-0.85	benign supporting
-0.999	[-1.078;-0.86	benign supporting
1.201	[-0.219;5.094	pathogenic moderate
-0.861	[-0.875;-0.84	benign supporting
-1.088	[-1.114;-1.03	benign supporting

-0.832	[-0.844;-0.81	benign supporting
-1.105	[-1.115;-0.98	benign supporting
1.242	[-0.069;5.103	pathogenic moderate
1.276	[0.591;5.090]	pathogenic strong
-0.200	[-0.403;0.352	indeterminate
-0.420	[-0.996;0.571	benign supporting
0.378	[-0.841;2.226	pathogenic supporting
1.224	[0.419;5.056]	pathogenic moderate
1.276	[-0.030;5.104	pathogenic strong
1.276	[1.192;1.395]	pathogenic strong
1.247	[1.112;1.463]	pathogenic moderate
0.895	[0.563;1.131]	pathogenic moderate
-0.868	[-1.053;-0.63	benign supporting
1.276	[-0.353;5.105	pathogenic strong
1.276	[1.106;1.634]	pathogenic strong
-0.216	[-0.384;0.139	indeterminate
1.127	[1.083;1.160]	pathogenic moderate
1.187	[1.157;1.217]	pathogenic moderate
1.276	[1.047;1.899]	pathogenic strong
1.276	[0.984;2.346]	pathogenic strong
1.224	[-1.084;5.065	pathogenic moderate
-0.013	[-0.285;0.679	indeterminate
-0.997	[-1.055;-0.92	benign supporting
-0.433	[-1.092;0.992	benign supporting
1.201	[0.435;4.963]	pathogenic moderate
-1.046	[-1.054;-1.03	benign supporting
1.276	[1.106;1.671]	pathogenic strong
-0.972	[-1.142;-0.89	benign supporting
0.577	[0.311;0.770]	pathogenic supporting
-0.632	[-1.108;-0.02	benign supporting
1.276	[1.217;1.350]	pathogenic strong
-0.349	[-0.361;-0.33	benign supporting
1.276	[0.781;5.081]	pathogenic strong
1.276	[1.065;1.839]	pathogenic strong
-0.963	[-1.019;-0.90	benign supporting
-0.578	[-0.612;-0.54	benign supporting
1.276	[1.129;1.550]	pathogenic strong
-0.456	[-0.541;-0.39	benign supporting
-1.112	[-1.116;-1.09	benign supporting
-1.102	[-1.115;-1.08	benign supporting
-0.982	[-1.046;-0.90	benign supporting
-0.977	[-0.999;-0.96	benign supporting
-1.099	[-1.103;-1.09	benign supporting

-1.062	[-1.074;-1.03	benign supporting
-1.167	[-1.170;-1.15	benign supporting
-1.106	[-1.110;-1.10	benign supporting
1.008	[0.179;1.282	pathogenic moderate
-0.626	[-1.071;-0.30	benign supporting
-1.101	[-1.115;-1.08	benign supporting
-1.101	[-1.120;-1.09	benign supporting
-0.321	[-0.340;-0.29	benign supporting
-0.454	[-0.630;-0.30	benign supporting
-0.037	[-0.172;0.318	indeterminate
-1.070	[-1.097;-1.01	benign supporting
1.276	[1.232;1.329	pathogenic strong
-1.091	[-1.114;-1.06	benign supporting
-0.906	[-0.924;-0.88	benign supporting
-0.660	[-0.728;-0.61	benign supporting
-0.956	[-1.021;-0.88	benign supporting
-1.116	[-1.169;-0.97	benign supporting
0.847	[-0.286;1.398	pathogenic moderate
0.747	[0.111;1.117	pathogenic moderate
-0.522	[-0.642;-0.40	benign supporting
1.143	[0.457;1.499	pathogenic moderate
-0.457	[-0.627;-0.30	benign supporting
1.276	[-1.101;5.061	pathogenic strong
1.219	[-1.084;5.074	pathogenic moderate
1.276	[0.000;5.100	pathogenic strong
1.276	[0.693;5.089	pathogenic strong
1.276	[1.118;1.585	pathogenic strong
1.276	[0.777;5.069	pathogenic strong
1.276	[-1.141;4.993	pathogenic strong
1.276	[1.011;2.012	pathogenic strong
1.276	[1.047;1.884	pathogenic strong
1.276	[-1.125;5.007	pathogenic strong
-0.390	[-0.485;-0.28	benign supporting
-0.375	[-0.407;-0.33	benign supporting
1.276	[-0.957;5.079	pathogenic strong
-1.009	[-1.032;-0.98	benign supporting
-1.116	[-1.115;-1.07	benign supporting
-0.914	[-2.831;4.754	benign supporting
-0.484	[-0.642;-0.34	benign supporting
-0.328	[-0.370;-0.27	benign supporting
-0.122	[-0.253;0.034	indeterminate
1.042	[0.871;1.135	pathogenic moderate
-1.096	[-1.102;-1.09	benign supporting

-0.640	[-0.671;-0.61	benign supporting
-1.086	[-1.129;-1.08	benign supporting
-0.440	[-0.497;-0.39	benign supporting
1.157	[1.061;1.234	pathogenic moderate
-0.740	[-0.826;-0.67	benign supporting
-1.162	[-1.170;-1.14	benign supporting
-1.084	[-1.167;-1.08	benign supporting
-1.094	[-1.108;-1.08	benign supporting
-0.631	[-0.813;-0.51	benign supporting
-0.651	[-0.690;-0.62	benign supporting
-0.069	[-0.128;0.00	indeterminate
-0.943	[-0.955;-0.93	benign supporting
-0.702	[-0.752;-0.66	benign supporting
0.374	[-0.347;1.12	pathogenic supporting
-1.097	[-1.104;-1.09	benign supporting
-1.104	[-1.109;-1.10	benign supporting
-0.821	[-0.838;-0.79	benign supporting
-1.103	[-1.107;-1.09	benign supporting
-0.644	[-0.851;-0.52	benign supporting
-1.093	[-1.099;-1.09	benign supporting
-0.702	[-0.729;-0.68	benign supporting
-0.108	[-0.139;-0.07	indeterminate
-1.084	[-1.088;-1.08	benign supporting
-1.109	[-1.114;-1.10	benign supporting
-1.090	[-1.102;-1.07	benign supporting
1.117	[1.101;1.132	pathogenic moderate
-1.106	[-1.114;-1.09	benign supporting
1.211	[1.176;1.246	pathogenic moderate
-1.166	[-1.170;-1.15	benign supporting
-1.104	[-1.108;-1.10	benign supporting
-1.084	[-1.086;-1.08	benign supporting
-0.628	[-0.664;-0.59	benign supporting
-0.673	[-0.769;-0.61	benign supporting
-0.881	[-0.940;-0.82	benign supporting
-0.553	[-0.616;-0.47	benign supporting
-0.981	[-1.102;-0.71	benign supporting
-0.778	[-1.053;-0.53	benign supporting
-1.089	[-1.110;-1.08	benign supporting
-1.098	[-1.109;-1.08	benign supporting
-1.085	[-1.168;-0.61	benign supporting
-1.020	[-1.053;-0.98	benign supporting
-0.995	[-1.037;-0.96	benign supporting
-0.584	[-0.723;-0.45	benign supporting

-1.104	[-1.115;-1.08	benign supporting
-0.480	[-0.503;-0.45	benign supporting
-0.470	[-0.502;-0.44	benign supporting
-1.064	[-1.105;-0.95	benign supporting
-1.101	[-1.115;-1.09	benign supporting
0.997	[0.818;1.114]	pathogenic moderate
-0.597	[-0.729;-0.47	benign supporting
0.115	[-0.052;0.362	indeterminate
-0.109	[-0.297;0.327	indeterminate
-0.391	[-0.429;-0.35	benign supporting
-0.005	[-0.182;0.459	indeterminate
-1.098	[-1.122;-1.08	benign supporting
-1.111	[-1.116;-1.10	benign supporting
-1.097	[-1.109;-1.08	benign supporting
-1.085	[-1.088;-1.08	benign supporting
-1.115	[-1.116;-1.10	benign supporting
-1.042	[-1.082;-0.96	benign supporting
-0.914	[-0.936;-0.88	benign supporting
-0.946	[-0.966;-0.92	benign supporting
-1.128	[-1.144;-1.11	benign supporting
-1.166	[-1.169;-1.15	benign supporting
-0.937	[-0.975;-0.89	benign supporting
-0.921	[-1.041;-0.75	benign supporting
1.276	[1.238;1.323]	pathogenic strong
-1.087	[-1.105;-1.08	benign supporting
-0.866	[-0.909;-0.82	benign supporting
0.635	[0.507;0.740]	pathogenic supporting
-0.973	[-0.979;-0.96	benign supporting
-0.872	[-1.055;-0.63	benign supporting
-1.056	[-1.098;-0.95	benign supporting
-1.121	[-1.126;-1.11	benign supporting
-0.902	[-0.948;-0.84	benign supporting
-0.088	[-0.142;-0.02	indeterminate
-0.835	[-0.852;-0.81	benign supporting
-0.771	[-0.879;-0.66	benign supporting
-1.109	[-1.115;-1.08	benign supporting
-0.738	[-0.845;-0.65	benign supporting
-1.090	[-1.098;-1.08	benign supporting
-1.084	[-1.085;-1.08	benign supporting
-1.037	[-1.047;-1.02	benign supporting
-1.050	[-1.068;-1.03	benign supporting
-1.084	[-1.134;-1.08	benign supporting
0.472	[-0.709;2.102	pathogenic supporting

-0.161	[-0.245;-0.07	indeterminate
-0.879	[-0.918;-0.84	benign supporting
-1.088	[-1.090;-1.08	benign supporting
-1.130	[-1.142;-1.11	benign supporting
0.840	[-0.018;1.205	pathogenic moderate
-1.092	[-1.169;-0.99	benign supporting
-0.496	[-0.917;-0.16	benign supporting
0.059	[-0.002;0.165	indeterminate
0.324	[-0.483;1.259	pathogenic supporting
-1.058	[-1.074;-1.02	benign supporting
-1.112	[-1.116;-1.09	benign supporting
-1.140	[-1.148;-1.13	benign supporting
-0.966	[-1.016;-0.91	benign supporting
-0.367	[-0.397;-0.33	benign supporting
-0.979	[-1.018;-0.93	benign supporting
-1.087	[-1.088;-1.08	benign supporting
-0.502	[-0.550;-0.45	benign supporting
-1.065	[-1.106;-0.95	benign supporting
-1.088	[-1.108;-1.08	benign supporting
1.224	[1.136;1.327	pathogenic moderate
-1.098	[-1.105;-1.09	benign supporting
-1.126	[-1.136;-1.11	benign supporting
-1.169	[-1.169;-1.08	benign supporting
-0.881	[-0.925;-0.83	benign supporting
-1.088	[-1.093;-1.08	benign supporting
-1.115	[-1.116;-1.10	benign supporting
1.276	[1.185;1.403	pathogenic strong
-0.354	[-0.423;-0.26	benign supporting
-1.092	[-1.164;-1.08	benign supporting
-0.848	[-0.877;-0.82	benign supporting
-0.446	[-0.493;-0.40	benign supporting
0.676	[0.603;0.737	pathogenic moderate
1.276	[1.187;1.398	pathogenic strong
-0.898	[-0.973;-0.81	benign supporting
0.025	[-0.080;0.308	indeterminate
-0.031	[-0.065;0.011	indeterminate
0.336	[0.007;0.585	pathogenic supporting
1.276	[1.182;1.411	pathogenic strong
-0.362	[-1.088;1.183	benign supporting
-1.081	[-1.114;-0.91	benign supporting
1.276	[1.235;1.325	pathogenic strong
-1.105	[-1.169;-1.01	benign supporting
1.276	[-0.229;5.103	pathogenic strong

-0.088	[-0.336;0.565	indeterminate
1.161	[1.059;1.248]	pathogenic moderate
-1.065	[-1.072;-1.04	benign supporting
-0.929	[-0.940;-0.91	benign supporting
-1.052	[-1.073;-1.01	benign supporting
0.564	[0.411;0.688]	pathogenic supporting
1.173	[0.889;1.362]	pathogenic moderate
-1.084	[-1.092;-1.07	benign supporting
0.250	[0.019;0.427]	indeterminate
-0.948	[-0.951;-0.94	benign supporting
-1.062	[-3.229;-0.33	benign supporting
1.009	[-0.423;4.921	pathogenic moderate
-0.736	[-0.990;-0.55	benign supporting
-0.048	[-0.077;-0.01	indeterminate
-1.138	[-1.162;-1.11	benign supporting
0.554	[0.051;0.851]	pathogenic supporting
-1.072	[-1.166;-0.33	benign supporting
1.276	[1.177;1.423]	pathogenic strong
-1.092	[-1.134;-1.08	benign supporting
-1.070	[-1.166;-1.01	benign supporting
0.010	[-0.380;0.965	indeterminate
-0.680	[-0.738;-0.63	benign supporting
-1.088	[-1.166;-1.08	benign supporting
-0.948	[-0.954;-0.94	benign supporting
-1.105	[-1.169;-0.87	benign supporting
-0.773	[-1.164;0.706	benign supporting
-0.431	[-0.494;-0.38	benign supporting
-1.169	[-1.169;-1.15	benign supporting
-1.082	[-1.107;-1.06	benign supporting
1.276	[1.230;1.331]	pathogenic strong
1.157	[1.086;1.225]	pathogenic moderate
-0.627	[-1.099;-0.11	benign supporting
-0.612	[-0.900;-0.41	benign supporting
-1.010	[-1.028;-0.99	benign supporting
-1.089	[-1.093;-1.08	benign supporting
1.008	[0.633;1.182]	pathogenic moderate
1.010	[0.768;1.140]	pathogenic moderate
1.276	[1.221;1.342]	pathogenic strong
-1.100	[-1.121;-1.08	benign supporting
-1.167	[-1.169;-0.50	benign supporting
1.276	[0.599;5.095]	pathogenic strong
0.540	[0.024;0.837]	pathogenic supporting
-1.105	[-1.111;-1.09	benign supporting

-0.920	[-0.922;-0.91	benign supporting
-1.089	[-1.092;-1.08	benign supporting
-0.973	[-1.071;-0.84	benign supporting
-1.069	[-1.089;-1.02	benign supporting
-1.104	[-1.111;-1.09	benign supporting
-0.949	[-1.094;-0.67	benign supporting
-1.083	[-1.114;-0.98	benign supporting
-1.163	[-1.170;-1.15	benign supporting
1.276	[1.163;1.458]	pathogenic strong
-1.165	[-1.168;-1.16	benign supporting
-0.903	[-1.077;-0.64	benign supporting
-0.484	[-0.845;-0.19	benign supporting
1.276	[1.225;1.336]	pathogenic strong
-1.163	[-1.169;-1.15	benign supporting
-1.098	[-1.168;-0.40	benign supporting
-1.164	[-3.453;1.357	benign supporting
1.276	[1.217;1.351]	pathogenic strong
0.352	[-0.174;0.863	pathogenic supporting
-1.092	[-1.094;-1.09	benign supporting
-1.129	[-1.134;-1.12	benign supporting
-1.138	[-1.148;-1.12	benign supporting
-1.092	[-1.116;-1.08	benign supporting
-1.012	[-1.022;-1.00	benign supporting
-1.090	[-1.099;-1.08	benign supporting
-1.105	[-1.108;-1.10	benign supporting
-0.938	[-1.134;-0.36	benign supporting
1.222	[1.183;1.262]	pathogenic moderate
-0.397	[-0.404;-0.39	benign supporting
1.276	[1.220;1.346]	pathogenic strong
1.276	[1.233;1.327]	pathogenic strong
-0.216	[-0.434;0.412	indeterminate
1.271	[1.119;1.581]	pathogenic moderate
1.276	[0.711;5.096]	pathogenic strong
-0.639	[-0.653;-0.62	benign supporting
-1.155	[-1.166;-1.14	benign supporting
-0.843	[-1.096;-0.50	benign supporting
0.000	[0.000;0.000]	indeterminate
-1.116	[-1.116;-1.10	benign supporting
-1.091	[-1.092;-1.09	benign supporting
-1.101	[-1.104;-1.09	benign supporting
-1.109	[-1.114;-1.10	benign supporting
-1.085	[-1.087;-1.08	benign supporting
-1.084	[-1.087;-1.08	benign supporting

-1.096	[-1.099;-1.09	benign supporting
-0.885	[-0.898;-0.87	benign supporting
-0.614	[-0.627;-0.59	benign supporting
-1.129	[-1.146;-1.11	benign supporting
-1.074	[-1.088;-1.05	benign supporting
-1.112	[-1.116;-1.10	benign supporting
-1.066	[-1.073;-1.04	benign supporting
-0.085	[-0.107;-0.06	indeterminate
-1.102	[-1.114;-1.08	benign supporting
1.219	[1.176;1.264	pathogenic moderate
-1.101	[-1.115;-1.08	benign supporting
-1.088	[-1.090;-1.08	benign supporting
-1.134	[-1.143;-1.12	benign supporting
-0.963	[-0.972;-0.95	benign supporting
-1.066	[-1.070;-1.05	benign supporting
-0.627	[-0.662;-0.59	benign supporting
1.276	[1.225;1.336	pathogenic strong
-1.085	[-1.087;-1.08	benign supporting
1.068	[0.522;1.263	pathogenic moderate
-0.966	[-1.094;-0.71	benign supporting
1.154	[1.108;1.203	pathogenic moderate
0.820	[0.726;0.921	pathogenic moderate
1.276	[1.103;1.702	pathogenic strong
-0.633	[-0.745;-0.55	benign supporting
1.276	[1.155;1.470	pathogenic strong
-0.176	[-0.213;-0.13	indeterminate
-1.091	[-1.115;-1.05	benign supporting
1.276	[1.233;1.327	pathogenic strong
0.505	[0.302;0.673	pathogenic supporting
0.953	[0.888;1.013	pathogenic moderate
-0.367	[-1.102;1.28	benign supporting
1.276	[1.239;1.318	pathogenic strong
1.239	[1.105;1.440	pathogenic moderate
-0.695	[-0.843;-0.61	benign supporting
-1.105	[-1.115;-1.09	benign supporting
1.276	[1.141;1.512	pathogenic strong
1.276	[1.229;1.333	pathogenic strong
0.822	[0.658;0.994	pathogenic moderate
0.969	[0.644;1.151	pathogenic moderate
-0.673	[-0.717;-0.63	benign supporting
1.276	[1.193;1.391	pathogenic strong
1.112	[0.957;1.177	pathogenic moderate
1.276	[1.070;1.776	pathogenic strong

1.023	[0.904;1.106]	pathogenic moderate
-0.788	[-0.853;-0.71	benign supporting
0.933	[0.745;1.103]	pathogenic moderate
0.360	[-0.538;1.328	pathogenic supporting
1.276	[1.156;1.469]	pathogenic strong
1.276	[1.211;1.359]	pathogenic strong
1.276	[1.237;1.323]	pathogenic strong
0.347	[0.076;0.546]	pathogenic supporting
1.276	[1.227;1.332]	pathogenic strong
1.148	[1.023;1.234]	pathogenic moderate
-0.919	[-1.093;-0.62	benign supporting
-1.092	[-1.108;-1.08	benign supporting
-0.750	[-1.092;-0.41	benign supporting
1.276	[0.663;5.089]	pathogenic strong
1.276	[1.142;1.519]	pathogenic strong
0.921	[0.619;1.130]	pathogenic moderate
-1.002	[-1.071;-0.90	benign supporting
0.733	[-0.247;1.252	pathogenic moderate
1.276	[-1.124;5.007	pathogenic strong
1.175	[0.360;2.076]	pathogenic moderate
1.276	[-1.093;5.048	pathogenic strong
-1.084	[-1.095;-1.08	benign supporting
1.276	[1.216;1.350]	pathogenic strong
1.276	[1.138;1.522]	pathogenic strong
1.276	[-0.308;5.104	pathogenic strong
1.276	[-1.115;5.006	pathogenic strong
1.172	[0.974;1.309]	pathogenic moderate
1.276	[0.821;5.077]	pathogenic strong
-0.284	[-0.620;0.732	indeterminate
0.272	[-1.084;4.850	indeterminate
1.276	[1.170;1.435]	pathogenic strong
1.276	[0.000;5.103]	pathogenic strong
-1.160	[-1.169;4.550	benign supporting
-0.583	[-0.961;-0.31	benign supporting
0.820	[0.612;1.023]	pathogenic moderate
-0.519	[-0.560;-0.47	benign supporting
1.276	[1.235;1.323]	pathogenic strong
-1.109	[-1.115;-1.10	benign supporting
-0.066	[-0.196;0.250	indeterminate
1.276	[1.210;1.358]	pathogenic strong
0.824	[0.758;0.895]	pathogenic moderate
-0.653	[-0.678;-0.63	benign supporting
-0.991	[-1.045;-0.92	benign supporting

1.097	[1.029;1.126]	pathogenic moderate
-1.084	[-1.087;-1.08	benign supporting
-1.096	[-1.103;-1.09	benign supporting
1.276	[1.227;1.334]	pathogenic strong
-0.362	[-0.400;-0.32	benign supporting
1.205	[1.165;1.246]	pathogenic moderate
-0.163	[-0.350;0.252	indeterminate
-1.097	[-1.114;-1.08	benign supporting
-0.413	[-0.455;-0.37	benign supporting
-0.305	[-0.554;0.310	indeterminate
-1.063	[-1.066;-1.05	benign supporting
-1.019	[-1.066;-0.96	benign supporting
1.276	[1.223;1.341]	pathogenic strong
-0.172	[-0.180;-0.16	indeterminate
0.868	[0.005;1.214]	pathogenic moderate
-0.688	[-0.712;-0.66	benign supporting
-1.088	[-1.091;-1.08	benign supporting
-1.081	[-1.084;-1.07	benign supporting
-0.011	[-0.137;0.340	indeterminate
-0.429	[-0.465;-0.39	benign supporting
-0.612	[-0.647;-0.57	benign supporting
1.160	[1.126;1.197]	pathogenic moderate
-0.460	[-0.465;-0.45	benign supporting
1.276	[1.242;1.316]	pathogenic strong
-0.126	[-0.197;-0.06	indeterminate
1.138	[1.046;1.198]	pathogenic moderate
-1.098	[-1.107;-1.09	benign supporting
1.276	[1.254;1.300]	pathogenic strong
1.174	[1.144;1.208]	pathogenic moderate
-1.025	[-1.051;-1.00	benign supporting
-0.972	[-0.989;-0.96	benign supporting
-0.530	[-0.552;-0.50	benign supporting
1.276	[1.200;1.371]	pathogenic strong
0.315	[-0.030;0.599	indeterminate
1.276	[1.142;1.509]	pathogenic strong
-0.252	[-0.302;-0.18	indeterminate
1.066	[1.006;1.112]	pathogenic moderate
1.258	[1.242;1.275]	pathogenic moderate
-0.310	[-0.324;-0.29	indeterminate
1.276	[1.222;1.344]	pathogenic strong
-0.673	[-0.720;-0.63	benign supporting
1.276	[1.260;1.294]	pathogenic strong
-0.544	[-0.549;-0.54	benign supporting

-0.715	[-0.756;-0.68	benign supporting
1.276	[1.253;1.301	pathogenic strong
-0.343	[-0.394;-0.27	benign supporting
1.270	[1.256;1.285	pathogenic moderate
0.579	[-0.394;1.288	pathogenic supporting
1.276	[1.248;1.307	pathogenic strong
1.276	[1.225;1.338	pathogenic strong
-0.678	[-0.753;-0.62	benign supporting
-0.998	[-1.015;-0.98	benign supporting
-0.531	[-0.651;-0.40	benign supporting
-0.375	[-0.540;-0.15	benign supporting
-0.827	[-0.937;-0.68	benign supporting
-1.084	[-1.088;-1.08	benign supporting
-1.090	[-1.097;-1.08	benign supporting
-1.120	[-1.167;-1.07	benign supporting
-1.165	[-1.166;-1.16	benign supporting
-1.107	[-1.113;-1.10	benign supporting
-1.115	[-1.167;-1.08	benign supporting
-1.115	[-1.168;-0.89	benign supporting
-0.774	[-0.786;-0.76	benign supporting
1.276	[-0.197;5.111	pathogenic strong
1.276	[1.115;1.628	pathogenic strong
-0.246	[-0.472;0.420	indeterminate
0.579	[0.408;0.721	pathogenic supporting
-0.965	[-1.094;-0.70	benign supporting
1.276	[-0.301;5.105	pathogenic strong
-0.881	[-0.890;-0.87	benign supporting
-1.097	[-1.103;-1.09	benign supporting
-1.089	[-1.090;-1.08	benign supporting
-0.129	[-0.132;-0.12	indeterminate
1.252	[1.238;1.265	pathogenic moderate
1.276	[1.258;1.295	pathogenic strong
0.436	[0.422;0.449	pathogenic supporting
1.276	[1.263;1.290	pathogenic strong
1.276	[1.216;1.347	pathogenic strong
1.276	[1.173;1.431	pathogenic strong
-0.420	[-0.423;-0.41	benign supporting
-1.052	[-1.056;-1.04	benign supporting
1.276	[1.271;1.281	pathogenic strong
-1.095	[-1.096;-1.09	benign supporting
-0.738	[-0.742;-0.73	benign supporting
0.530	[0.508;0.551	pathogenic supporting
1.276	[1.052;1.868	pathogenic strong

1.276	[1.124;1.595	pathogenic strong
-0.629	[-0.641;-0.61	benign supporting
-0.909	[-0.911;-0.90	benign supporting
-0.716	[-0.719;-0.71	benign supporting
-1.098	[-1.100;-1.09	benign supporting
-0.728	[-0.781;-0.68	benign supporting
-0.968	[-1.168;-0.83	benign supporting
1.017	[0.643;1.186	pathogenic moderate
-0.355	[-0.563;-0.04	benign supporting
-1.104	[-1.112;-1.09	benign supporting
1.270	[1.070;1.723	pathogenic moderate
1.276	[1.172;1.428	pathogenic strong
1.276	[1.234;1.327	pathogenic strong
-0.288	[-0.341;-0.22	indeterminate
1.276	[1.221;1.344	pathogenic strong
1.069	[0.928;1.138	pathogenic moderate
0.511	[0.411;0.602	pathogenic supporting
-1.086	[-1.096;-1.08	benign supporting
1.276	[1.173;1.431	pathogenic strong
1.276	[1.218;1.346	pathogenic strong
-0.836	[-0.946;-0.69	benign supporting
1.276	[1.081;1.737	pathogenic strong
-0.463	[-0.575;-0.37	benign supporting
-0.425	[-0.433;-0.41	benign supporting
-1.084	[-1.084;-1.08	benign supporting
-1.016	[-1.059;-0.96	benign supporting
1.230	[1.117;1.379	pathogenic moderate
1.002	[0.877;1.097	pathogenic moderate
-0.256	[-0.329;-0.15	indeterminate
1.276	[1.180;1.411	pathogenic strong
1.276	[-0.338;5.09	pathogenic strong
1.276	[0.886;5.013	pathogenic strong
1.276	[0.984;2.382	pathogenic strong
-0.998	[-1.164;-0.36	benign supporting
1.276	[0.056;5.100	pathogenic strong
0.615	[-0.704;2.71	pathogenic supporting
-1.036	[-1.164;-0.38	benign supporting
1.276	[0.725;5.084	pathogenic strong
-0.515	[-1.105;0.79	benign supporting
1.276	[-0.100;5.10	pathogenic strong
0.571	[-0.263;1.16	pathogenic supporting
0.488	[0.017;0.783	pathogenic supporting
1.276	[1.208;1.365	pathogenic strong

-0.433	[-0.641;-0.22	benign supporting
-1.104	[-1.115;-1.09	benign supporting
-1.095	[-1.102;-1.08	benign supporting
-0.940	[-0.958;-0.92	benign supporting
-0.708	[-0.759;-0.66	benign supporting
-1.097	[-1.104;-1.09	benign supporting
-1.097	[-1.105;-1.09	benign supporting
-0.452	[-0.518;-0.39	benign supporting
-0.859	[-0.883;-0.83	benign supporting
-1.016	[-1.048;-0.98	benign supporting
-1.084	[-1.118;-1.08	benign supporting
-1.085	[-1.088;-1.08	benign supporting
-0.728	[-0.775;-0.68	benign supporting
-0.406	[-0.474;-0.33	benign supporting
-0.282	[-0.368;-0.15	indeterminate
-0.637	[-0.648;-0.62	benign supporting
-0.554	[-0.564;-0.54	benign supporting
-0.386	[-0.418;-0.34	benign supporting
-0.957	[-1.077;-0.76	benign supporting
-1.059	[-1.114;-0.70	benign supporting
-0.518	[-0.539;-0.49	benign supporting
-0.566	[-0.651;-0.47	benign supporting
-1.087	[-1.102;-1.07	benign supporting
-0.976	[-0.984;-0.97	benign supporting
-1.014	[-1.028;-1.00	benign supporting
1.276	[1.185;1.407	pathogenic strong
-0.781	[-0.810;-0.75	benign supporting
-0.853	[-1.106;-0.45	benign supporting
0.092	[0.021;0.169	indeterminate
-1.030	[-1.041;-1.01	benign supporting
-1.136	[-1.169;-1.10	benign supporting
-0.300	[-0.619;0.57	indeterminate
1.276	[1.247;1.309	pathogenic strong
-1.123	[-1.145;-1.10	benign supporting
0.779	[0.528;1.001	pathogenic moderate
-1.106	[-1.115;-1.09	benign supporting
-1.101	[-1.164;-0.69	benign supporting
-1.091	[-1.124;-1.08	benign supporting
1.276	[0.000;5.102	pathogenic strong
0.526	[-0.307;1.17	pathogenic supporting
-0.230	[-0.540;0.71	indeterminate
1.104	[0.932;1.170	pathogenic moderate
1.276	[0.478;5.096	pathogenic strong

0.695	[-0.416;1.411	pathogenic moderate
-0.434	[-0.474;-0.40	benign supporting
0.012	[-0.127;0.39⁴	indeterminate
-0.912	[-0.916;-0.90	benign supporting
-0.565	[-0.593;-0.53	benign supporting
-1.126	[-1.169;-1.07	benign supporting
1.276	[0.567;5.095	pathogenic strong
-0.955	[-1.108;-0.62	benign supporting
-0.630	[-0.871;-0.47	benign supporting
-0.353	[-0.480;-0.17	benign supporting
1.276	[1.245;1.311	pathogenic strong
-0.939	[-0.956;-0.92	benign supporting
-1.169	[-1.169;-1.10	benign supporting
-1.159	[-1.168;-0.83	benign supporting
1.171	[1.150;1.193	pathogenic moderate
-0.416	[-0.807;0.01⁴	benign supporting
-0.674	[-0.681;-0.66	benign supporting
-0.397	[-0.405;-0.38	benign supporting
-1.027	[-1.035;-1.02	benign supporting
-0.971	[-0.976;-0.96	benign supporting
-0.275	[-0.296;-0.25	indeterminate
1.107	[1.019;1.146	pathogenic moderate
-0.439	[-0.444;-0.43	benign supporting
-1.166	[-1.169;-1.11	benign supporting
1.253	[1.052;1.575	pathogenic moderate
0.605	[0.566;0.642	pathogenic supporting
-0.887	[-0.895;-0.87	benign supporting
1.276	[1.156;1.466	pathogenic strong
-0.890	[-0.992;-0.75	benign supporting
0.757	[0.481;0.992	pathogenic moderate
-0.562	[-0.574;-0.55	benign supporting
-0.911	[-0.920;-0.89	benign supporting
-1.060	[-1.066;-1.05	benign supporting
-1.114	[-1.126;-1.10	benign supporting
-0.455	[-0.467;-0.44	benign supporting
-0.961	[-0.967;-0.95	benign supporting
1.276	[0.677;5.091	pathogenic strong
1.276	[1.176;1.422	pathogenic strong
1.276	[1.080;1.704	pathogenic strong
0.574	[0.429;0.700	pathogenic supporting
1.276	[1.175;1.435	pathogenic strong
0.752	[0.606;0.877	pathogenic moderate
-0.905	[-0.925;-0.88	benign supporting

-0.986	[-1.020;-0.96	benign supporting
1.276	[0.016;5.100]	pathogenic strong
1.276	[1.158;1.463]	pathogenic strong
-1.090	[-1.096;-1.08	benign supporting
1.276	[0.892;5.030]	pathogenic strong
-0.018	[-0.216;0.502	indeterminate
-0.060	[-0.077;-0.04	indeterminate
-1.166	[-1.167;-1.16	benign supporting
-0.919	[-0.932;-0.90	benign supporting
-1.002	[-1.019;-0.98	benign supporting
-1.045	[-1.052;-1.03	benign supporting
-0.573	[-0.578;-0.56	benign supporting
1.251	[1.229;1.274]	pathogenic moderate
-0.440	[-0.513;-0.38	benign supporting
-0.396	[-0.405;-0.38	benign supporting
-1.002	[-1.036;-0.95	benign supporting
1.276	[1.223;1.340]	pathogenic strong
-0.533	[-0.546;-0.52	benign supporting
-0.278	[-0.282;-0.27	indeterminate
1.276	[1.236;1.326]	pathogenic strong
-1.015	[-1.112;-0.68	benign supporting
1.276	[1.242;1.312]	pathogenic strong
-1.097	[-1.115;-1.07	benign supporting
-1.089	[-1.098;-1.08	benign supporting
-0.973	[-0.974;-0.97	benign supporting
-0.596	[-0.600;-0.59	benign supporting
-1.105	[-1.114;-1.09	benign supporting
0.710	[0.073;1.085]	pathogenic moderate
-0.349	[-1.087;1.175	benign supporting
-0.101	[-0.635;1.167	indeterminate
0.913	[0.353;1.184]	pathogenic moderate
-0.089	[-0.220;0.159	indeterminate
0.946	[0.404;1.198]	pathogenic moderate
1.276	[-0.122;5.102	pathogenic strong
1.276	[-0.065;5.098	pathogenic strong
-0.885	[-0.890;-0.88	benign supporting
1.106	[0.751;1.235]	pathogenic moderate
1.276	[0.757;5.074]	pathogenic strong
-0.643	[-1.131;0.817	benign supporting
-1.167	[-1.169;-1.03	benign supporting
1.276	[1.027;2.010]	pathogenic strong
1.076	[0.893;1.156]	pathogenic moderate
1.276	[1.216;1.351]	pathogenic strong

1.276	[1.193;1.392	pathogenic strong
-0.966	[-0.976;-0.95	benign supporting
-0.839	[-0.840;-0.83	benign supporting
-1.084	[-1.167;-1.08	benign supporting
-1.074	[-1.088;-1.05	benign supporting
-0.262	[-0.337;-0.16	indeterminate
-0.577	[-0.910;-0.35	benign supporting
-0.245	[-0.389;-0.01	indeterminate
-0.784	[-0.936;-0.63	benign supporting
-0.464	[-0.502;-0.43	benign supporting
-0.483	[-1.164;3.567	benign supporting
-0.435	[-0.514;-0.37	benign supporting
0.656	[0.561;0.740	pathogenic moderate
1.276	[0.917;4.993	pathogenic strong
-1.079	[-1.104;-1.04	benign supporting
-0.997	[-1.016;-0.96	benign supporting
-0.612	[-0.626;-0.59	benign supporting
-1.092	[-1.114;-1.08	benign supporting
-1.150	[-1.169;-0.96	benign supporting
-0.538	[-0.572;-0.50	benign supporting
-0.255	[-0.297;-0.20	indeterminate
-0.428	[-0.457;-0.40	benign supporting
0.014	[-0.051;0.174	indeterminate
-1.084	[-1.086;-1.08	benign supporting
-1.055	[-1.112;-0.82	benign supporting
-1.087	[-2.744;4.629	benign supporting
-0.385	[-0.444;-0.31	benign supporting
-0.637	[-0.652;-0.62	benign supporting
-0.694	[-0.741;-0.66	benign supporting
-0.191	[-0.238;-0.14	indeterminate
1.192	[1.137;1.252	pathogenic moderate
1.232	[1.194;1.272	pathogenic moderate
-1.127	[-1.138;-1.11	benign supporting
-0.077	[-0.117;-0.02	indeterminate
0.411	[0.299;0.511	pathogenic supporting
-0.871	[-0.905;-0.83	benign supporting
-1.067	[-1.076;-1.04	benign supporting
-0.641	[-0.661;-0.62	benign supporting
-1.115	[-1.116;-1.10	benign supporting
1.276	[1.242;1.316	pathogenic strong
-0.460	[-0.482;-0.44	benign supporting
1.276	[1.230;1.331	pathogenic strong
-0.656	[-0.693;-0.62	benign supporting

0.834	[0.771;0.902]	pathogenic moderate
-1.064	[-1.071;-1.04	benign supporting
-0.834	[-0.858;-0.80	benign supporting
-1.084	[-1.122;-1.08	benign supporting
-0.823	[-1.165;1.109	benign supporting
-1.090	[-1.122;-1.08	benign supporting
1.276	[0.971;2.806]	pathogenic strong
-0.966	[-1.088;-0.73	benign supporting
-0.111	[-0.312;0.365	indeterminate
1.276	[0.000;5.104]	pathogenic strong
-1.067	[-1.090;-1.01	benign supporting
-0.120	[-0.446;0.806	indeterminate
-1.090	[-1.113;-1.08	benign supporting
0.889	[-1.099;5.046	pathogenic moderate
-1.101	[-1.112;-1.09	benign supporting
-1.101	[-1.114;-1.08	benign supporting
-1.083	[-1.166;-0.26	benign supporting
-0.831	[-1.107;-0.41	benign supporting
1.276	[-1.124;4.968	pathogenic strong
1.276	[-0.382;5.098	pathogenic strong
-0.608	[-0.700;-0.51	benign supporting
-0.050	[-0.292;0.589	indeterminate
-0.523	[-1.065;-0.03	benign supporting
-0.988	[-1.168;-0.83	benign supporting
1.276	[1.159;1.467]	pathogenic strong
0.477	[-0.109;0.958	pathogenic supporting
-0.914	[-0.947;-0.87	benign supporting
-0.396	[-0.454;-0.33	benign supporting
-0.781	[-0.833;-0.72	benign supporting
-0.838	[-0.844;-0.83	benign supporting
-0.659	[-0.695;-0.62	benign supporting
-0.563	[-0.599;-0.52	benign supporting
-1.115	[-1.116;-1.09	benign supporting
-1.079	[-1.099;-1.05	benign supporting
-1.068	[-1.093;-1.05	benign supporting
-0.993	[-1.059;-0.91	benign supporting
-0.584	[-0.627;-0.53	benign supporting
-0.710	[-0.760;-0.67	benign supporting
1.276	[0.513;5.101]	pathogenic strong
-0.634	[-0.745;-0.55	benign supporting
-0.340	[-0.378;-0.29	benign supporting
-0.968	[-1.001;-0.93	benign supporting
-1.040	[-1.113;-0.69	benign supporting

-1.112	[-1.116;-1.09	benign supporting
1.138	[1.079;1.185]	pathogenic moderate
1.276	[1.131;1.540]	pathogenic strong
1.276	[1.163;1.462]	pathogenic strong
1.276	[1.127;1.561]	pathogenic strong
-0.918	[-0.925;-0.91	benign supporting
-0.689	[-1.096;-0.34	benign supporting
-1.095	[-1.109;-1.08	benign supporting
1.276	[1.193;1.385]	pathogenic strong
1.276	[1.146;1.501]	pathogenic strong
-1.160	[-1.169;-1.09	benign supporting
1.276	[1.117;1.619]	pathogenic strong
-0.761	[-0.787;-0.73	benign supporting
1.276	[1.186;1.397]	pathogenic strong
0.398	[-1.114;5.017	pathogenic supporting
-0.261	[-0.629;0.79(	indeterminate
1.276	[0.910;4.895]	pathogenic strong
-0.313	[-0.584;0.36:	indeterminate
0.392	[0.110;0.607]	pathogenic supporting
-1.109	[-1.115;-1.07	benign supporting
-0.531	[-0.883;-0.27	benign supporting
-0.627	[-0.644;-0.61	benign supporting
-0.607	[-0.959;-0.36	benign supporting
-0.383	[-0.484;-0.26	benign supporting
1.243	[1.186;1.305]	pathogenic moderate
0.254	[0.053;0.401]	indeterminate
-0.635	[-0.654;-0.62	benign supporting
-0.526	[-0.548;-0.50	benign supporting
0.522	[0.388;0.635]	pathogenic supporting
-0.552	[-0.605;-0.49	benign supporting
-0.882	[-0.918;-0.84	benign supporting
-1.100	[-1.102;-1.09	benign supporting
1.276	[0.978;2.214]	pathogenic strong
-1.084	[-1.086;-1.08	benign supporting
-0.825	[-0.837;-0.80	benign supporting
-0.574	[-0.616;-0.53	benign supporting
-0.820	[-0.881;-0.73	benign supporting
1.276	[1.235;1.323]	pathogenic strong
-0.133	[-0.191;-0.08	indeterminate
-0.736	[-0.779;-0.69	benign supporting
-0.767	[-0.816;-0.72	benign supporting
-1.111	[-1.116;-1.09	benign supporting
-0.468	[-0.639;-0.32	benign supporting

1.217	[1.175;1.261	pathogenic moderate
1.276	[1.214;1.356	pathogenic strong
1.276	[1.216;1.353	pathogenic strong
-0.472	[-0.498;-0.45	benign supporting
1.276	[1.001;2.359	pathogenic strong
1.137	[0.860;1.270	pathogenic moderate
0.911	[0.605;1.129	pathogenic moderate
1.276	[1.243;1.315	pathogenic strong
-0.625	[-0.664;-0.58	benign supporting
1.276	[1.119;1.598	pathogenic strong
-0.822	[-0.849;-0.78	benign supporting
-0.374	[-0.386;-0.36	benign supporting
1.017	[0.582;1.202	pathogenic moderate
1.276	[1.185;1.403	pathogenic strong
0.252	[-0.148;0.72	indeterminate
0.020	[-0.114;0.38	indeterminate
-0.138	[-0.170;-0.10	indeterminate
1.276	[1.229;1.332	pathogenic strong
-0.656	[-1.168;4.07	benign supporting
-1.109	[-1.115;-1.09	benign supporting
-0.154	[-0.262;-0.04	indeterminate
0.623	[0.280;0.860	pathogenic supporting
0.825	[0.782;0.870	pathogenic moderate
1.175	[1.142;1.210	pathogenic moderate
-0.263	[-0.274;-0.25	indeterminate
-0.023	[-0.049;0.00	indeterminate
1.262	[1.235;1.291	pathogenic moderate
1.216	[1.210;1.222	pathogenic moderate
0.330	[0.201;0.430	pathogenic supporting
-1.165	[-1.166;-1.16	benign supporting
1.276	[1.250;1.305	pathogenic strong
-1.047	[-1.052;-1.04	benign supporting
-0.403	[-0.410;-0.39	benign supporting
1.276	[1.248;1.307	pathogenic strong
-0.311	[-0.328;-0.29	indeterminate
1.276	[1.260;1.293	pathogenic strong
0.814	[0.767;0.864	pathogenic moderate
1.276	[1.262;1.292	pathogenic strong
0.829	[0.791;0.869	pathogenic moderate
-1.008	[-1.013;-1.00	benign supporting
-1.088	[-1.092;-1.08	benign supporting
1.276	[0.035;5.107	pathogenic strong
-0.704	[-0.956;-0.53	benign supporting

1.276	[-1.116;4.993	pathogenic strong
-0.318	[-0.379;-0.24	benign supporting
-0.659	[-0.725;-0.61	benign supporting
-1.044	[-1.071;-1.00	benign supporting
0.656	[-0.106;1.129	pathogenic moderate
-1.106	[-1.121;-1.09	benign supporting
0.945	[0.763;1.103	pathogenic moderate
-1.084	[-1.089;-1.08	benign supporting
-1.150	[-1.170;-1.12	benign supporting
-1.169	[-1.169;-1.14	benign supporting
-1.104	[-1.128;-1.08	benign supporting
-0.839	[-3.721;-0.83	benign supporting
-0.321	[-0.720;0.751	benign supporting
1.276	[1.229;1.334	pathogenic strong
-0.360	[-1.099;1.230	benign supporting
-0.261	[-0.305;-0.20	indeterminate
1.276	[1.237;1.322	pathogenic strong
-1.065	[-1.070;-1.05	benign supporting
-1.111	[-1.116;-1.09	benign supporting
-0.191	[-0.416;0.451	indeterminate
0.874	[0.785;0.974	pathogenic moderate
0.831	[0.760;0.907	pathogenic moderate
0.399	[0.066;0.634	pathogenic supporting
-1.110	[-1.115;-1.10	benign supporting
-0.621	[-0.656;-0.58	benign supporting
1.276	[1.257;1.298	pathogenic strong
-0.871	[-0.887;-0.85	benign supporting
-0.731	[-0.763;-0.70	benign supporting
0.168	[-0.058;0.452	indeterminate
0.533	[0.429;0.624	pathogenic supporting
-0.766	[-0.828;-0.70	benign supporting
-0.371	[-0.389;-0.35	benign supporting
1.276	[1.154;1.484	pathogenic strong
-0.261	[-0.464;0.283	indeterminate
1.276	[1.193;1.385	pathogenic strong
-0.479	[-0.539;-0.42	benign supporting
-0.351	[-0.369;-0.33	benign supporting
-0.316	[-0.337;-0.29	indeterminate
-0.878	[-0.921;-0.83	benign supporting
-1.095	[-1.112;-1.08	benign supporting
0.039	[-0.052;0.265	indeterminate
1.276	[1.189;1.396	pathogenic strong
1.105	[0.868;1.190	pathogenic moderate

1.276	[1.157;1.473	pathogenic strong
-1.116	[-1.116;-1.10	benign supporting
-1.115	[-1.116;-1.09	benign supporting
1.276	[1.223;1.341	pathogenic strong
1.002	[0.927;1.056	pathogenic moderate
-1.011	[-1.031;-0.99	benign supporting
-0.722	[-0.774;-0.68	benign supporting
-0.622	[-0.642;-0.60	benign supporting
-1.094	[-1.106;-1.08	benign supporting
-0.402	[-0.426;-0.38	benign supporting
1.276	[1.159;1.457	pathogenic strong
-0.293	[-0.331;-0.24	indeterminate
1.240	[1.213;1.270	pathogenic moderate
-0.562	[-0.627;-0.49	benign supporting
-0.359	[-0.378;-0.34	benign supporting
-1.061	[-1.070;-1.04	benign supporting
-0.422	[-0.443;-0.40	benign supporting
-1.073	[-1.082;-1.06	benign supporting
-0.400	[-0.508;-0.29	benign supporting
1.276	[1.225;1.338	pathogenic strong
-0.823	[-0.908;-0.70	benign supporting
1.276	[1.157;1.474	pathogenic strong
-0.615	[-0.655;-0.57	benign supporting
-1.098	[-1.110;-1.09	benign supporting
-0.371	[-0.418;-0.31	benign supporting
-1.099	[-1.115;-1.05	benign supporting
-0.658	[-0.861;-0.53	benign supporting
-0.840	[-1.013;-0.63	benign supporting
1.223	[1.154;1.298	pathogenic moderate
-1.087	[-1.092;-1.08	benign supporting
-1.064	[-1.074;-1.03	benign supporting
1.276	[1.236;1.322	pathogenic strong
-0.360	[-0.445;-0.24	benign supporting
1.217	[1.194;1.240	pathogenic moderate
-0.450	[-0.549;-0.37	benign supporting
1.276	[1.226;1.337	pathogenic strong
-0.972	[-0.996;-0.95	benign supporting
-0.964	[-1.008;-0.92	benign supporting
-1.110	[-1.116;-1.10	benign supporting
-0.251	[-0.319;-0.15	indeterminate
-0.567	[-0.623;-0.50	benign supporting
0.833	[0.782;0.886	pathogenic moderate
1.276	[1.257;1.298	pathogenic strong

-1.101	[-1.108;-1.09	benign supporting
-0.103	[-0.147;-0.06	indeterminate
1.076	[1.000;1.122	pathogenic moderate
0.806	[0.708;0.908	pathogenic moderate
-0.611	[-0.628;-0.59	benign supporting
-1.089	[-1.090;-1.08	benign supporting
-1.131	[-1.145;-1.11	benign supporting
-1.164	[-1.166;-1.16	benign supporting
1.276	[1.190;1.394	pathogenic strong
-0.155	[-0.189;-0.12	indeterminate
1.276	[1.242;1.316	pathogenic strong
-0.449	[-0.605;-0.32	benign supporting
-0.126	[-0.177;-0.08	indeterminate
-0.621	[-0.641;-0.60	benign supporting
-0.895	[-0.961;-0.82	benign supporting
-1.117	[-1.159;-1.09	benign supporting
0.451	[0.372;0.527	pathogenic supporting
1.116	[1.099;1.132	pathogenic moderate
-0.404	[-0.432;-0.37	benign supporting
0.992	[0.723;1.141	pathogenic moderate
-0.345	[-0.363;-0.32	benign supporting
0.506	[0.398;0.601	pathogenic supporting
1.276	[1.251;1.303	pathogenic strong
1.276	[1.253;1.301	pathogenic strong
1.276	[1.248;1.306	pathogenic strong
-0.370	[-0.400;-0.33	benign supporting
1.276	[1.235;1.325	pathogenic strong
-1.110	[-1.115;-1.10	benign supporting
0.013	[-0.025;0.08	indeterminate
1.276	[-1.111;5.02	pathogenic strong
1.276	[1.266;1.287	pathogenic strong
1.276	[1.219;1.346	pathogenic strong
-0.303	[-0.332;-0.27	indeterminate
1.276	[1.213;1.354	pathogenic strong
1.276	[1.238;1.320	pathogenic strong
-1.095	[-1.115;-1.05	benign supporting
-1.110	[-1.115;-1.09	benign supporting
-1.011	[-1.058;-0.95	benign supporting
1.276	[1.268;1.285	pathogenic strong
-0.244	[-0.358;-0.07	indeterminate
1.276	[1.229;1.333	pathogenic strong
-1.045	[-1.050;-1.04	benign supporting
-0.327	[-0.352;-0.29	benign supporting

1.276	[1.249;1.306]	pathogenic strong
-0.676	[-0.841;-0.58	benign supporting
-1.098	[-1.100;-1.09	benign supporting
1.189	[1.174;1.204]	pathogenic moderate
1.276	[1.240;1.317]	pathogenic strong
-0.587	[-0.602;-0.57	benign supporting
-0.551	[-0.564;-0.53	benign supporting
0.717	[0.553;0.849]	pathogenic moderate
-0.181	[-0.401;0.41[illegible]	indeterminate
0.751	[0.711;0.790]	pathogenic moderate
-0.957	[-1.075;-0.77	benign supporting
1.276	[1.253;1.300]	pathogenic strong
1.276	[1.225;1.337]	pathogenic strong
1.276	[1.237;1.321]	pathogenic strong
-1.082	[-1.104;-1.06	benign supporting
1.276	[1.115;1.613]	pathogenic strong
1.276	[1.264;1.289]	pathogenic strong
-0.246	[-0.305;-0.17	indeterminate
1.276	[1.244;1.310]	pathogenic strong
1.276	[1.224;1.338]	pathogenic strong
-0.364	[-0.380;-0.34	benign supporting
1.276	[1.220;1.346]	pathogenic strong
-1.090	[-1.091;-1.08	benign supporting
1.114	[1.084;1.134]	pathogenic moderate
0.461	[0.342;0.573]	pathogenic supporting
1.276	[1.218;1.346]	pathogenic strong
-1.095	[-1.098;-1.09	benign supporting
-0.179	[-0.227;-0.13	indeterminate
-0.505	[-1.088;0.51[illegible]	benign supporting
-0.530	[-0.554;-0.50	benign supporting
1.228	[1.076;1.434]	pathogenic moderate
-0.013	[-0.226;0.53[illegible]	indeterminate
1.276	[1.218;1.346]	pathogenic strong
1.276	[1.176;1.425]	pathogenic strong
0.570	[-0.027;0.95[illegible]	pathogenic supporting
-0.638	[-0.704;-0.58	benign supporting
1.276	[1.232;1.327]	pathogenic strong
1.230	[1.224;1.236]	pathogenic moderate
1.276	[1.111;1.613]	pathogenic strong
0.566	[0.518;0.607]	pathogenic supporting
-0.841	[-0.847;-0.83	benign supporting
1.194	[1.158;1.231]	pathogenic moderate
1.276	[1.259;1.294]	pathogenic strong

-0.242	[-0.302;-0.16	indeterminate
1.276	[0.864;5.015]	pathogenic strong
1.237	[1.217;1.260]	pathogenic moderate
1.276	[1.257;1.297]	pathogenic strong
-1.135	[-1.140;-1.13	benign supporting
-1.094	[-1.095;-1.09	benign supporting
0.498	[-0.355;1.196	pathogenic supporting
0.964	[0.817;1.084]	pathogenic moderate
1.276	[1.215;1.351]	pathogenic strong
-0.200	[-0.890;1.196	indeterminate
0.838	[0.821;0.855]	pathogenic moderate
1.276	[1.237;1.321]	pathogenic strong
-0.332	[-0.853;0.873	benign supporting
-0.737	[-0.922;-0.60	benign supporting
-1.128	[-1.168;-1.09	benign supporting
1.217	[1.181;1.255]	pathogenic moderate
1.270	[1.218;1.331]	pathogenic moderate
1.054	[0.831;1.154]	pathogenic moderate
-0.760	[-0.784;-0.73	benign supporting
1.276	[1.252;1.302]	pathogenic strong
1.276	[1.251;1.303]	pathogenic strong
1.156	[0.935;1.289]	pathogenic moderate
1.194	[1.171;1.218]	pathogenic moderate
1.276	[1.120;1.594]	pathogenic strong
-1.103	[-1.106;-1.10	benign supporting
-0.291	[-0.332;-0.24	indeterminate
1.276	[1.067;1.816]	pathogenic strong
-0.114	[-0.591;1.115	indeterminate
1.276	[1.237;1.321]	pathogenic strong
-1.025	[-1.087;-0.91	benign supporting
0.286	[0.183;0.369]	indeterminate
-0.660	[-0.678;-0.64	benign supporting
-1.033	[-1.067;-0.99	benign supporting
-0.906	[-0.908;-0.90	benign supporting
-0.482	[-0.617;-0.36	benign supporting
0.797	[0.200;1.136]	pathogenic moderate
-1.085	[-1.086;-1.08	benign supporting
0.010	[-0.045;0.134	indeterminate
-1.038	[-1.049;-1.02	benign supporting
1.276	[1.240;1.318]	pathogenic strong
0.930	[0.850;1.009]	pathogenic moderate
-1.100	[-1.105;-1.09	benign supporting
-0.348	[-0.362;-0.33	benign supporting

-1.101	[-1.108;-1.09	benign supporting
-1.104	[-1.107;-1.10	benign supporting
-1.067	[-1.071;-1.06	benign supporting
-1.056	[-1.072;-1.02	benign supporting
-0.103	[-0.231;0.083	indeterminate
1.083	[0.698;1.222	pathogenic moderate
-0.079	[-0.127;-0.01	indeterminate
-1.115	[-1.116;-1.11	benign supporting
-0.543	[-0.551;-0.53	benign supporting
0.540	[-0.069;0.970	pathogenic supporting
-1.140	[-1.169;-1.08	benign supporting
-1.102	[-1.115;-1.08	benign supporting
-1.143	[-1.149;-1.13	benign supporting
-0.423	[-1.099;1.104	benign supporting
-1.165	[-1.166;-1.16	benign supporting
0.769	[0.619;0.908	pathogenic moderate
-1.067	[-1.070;-1.06	benign supporting
-0.575	[-0.596;-0.55	benign supporting
1.276	[1.248;1.307	pathogenic strong
1.130	[1.103;1.155	pathogenic moderate
-0.483	[-0.521;-0.44	benign supporting
-1.086	[-1.091;-1.08	benign supporting
-0.334	[-0.346;-0.32	benign supporting
-0.457	[-0.466;-0.44	benign supporting
1.276	[1.104;1.692	pathogenic strong
1.210	[1.142;1.288	pathogenic moderate
0.696	[-0.093;1.142	pathogenic moderate
-0.212	[-0.425;0.384	indeterminate
-1.046	[-1.052;-1.04	benign supporting
1.276	[1.106;1.685	pathogenic strong
-1.093	[-1.125;-1.08	benign supporting
-1.073	[-1.083;-1.06	benign supporting
1.276	[1.048;1.807	pathogenic strong
1.276	[1.218;1.347	pathogenic strong
1.276	[0.743;5.079	pathogenic strong
-1.028	[-1.108;-0.83	benign supporting
1.144	[-0.403;5.079	pathogenic moderate
-0.418	[-0.549;-0.30	benign supporting
1.209	[1.171;1.247	pathogenic moderate
1.276	[0.921;4.971	pathogenic strong
1.276	[0.508;5.102	pathogenic strong
1.276	[-0.341;5.108	pathogenic strong
-1.088	[-1.094;-1.08	benign supporting

1.276	[1.235;1.323	pathogenic strong
-0.405	[-0.609;-0.17	benign supporting
1.109	[1.045;1.140	pathogenic moderate
1.276	[0.693;5.093	pathogenic strong
1.276	[0.357;5.100	pathogenic strong
1.145	[1.122;1.166	pathogenic moderate
-0.442	[-0.590;-0.32	benign supporting
-1.108	[-1.115;-1.09	benign supporting
1.114	[0.931;1.192	pathogenic moderate
1.156	[1.140;1.174	pathogenic moderate
0.423	[0.230;0.580	pathogenic supporting
1.276	[-0.102;5.108	pathogenic strong
-1.108	[-1.112;-1.10	benign supporting
-0.922	[-0.926;-0.91	benign supporting
1.276	[1.039;1.927	pathogenic strong
1.276	[1.168;1.438	pathogenic strong
1.163	[1.136;1.194	pathogenic moderate
1.276	[1.246;1.310	pathogenic strong
1.276	[1.218;1.350	pathogenic strong
-0.656	[-0.676;-0.63	benign supporting
1.276	[1.258;1.295	pathogenic strong
1.074	[0.826;1.175	pathogenic moderate
-1.138	[-1.168;-0.91	benign supporting
0.564	[-0.058;0.976	pathogenic supporting
-1.106	[-1.168;-0.85	benign supporting
1.276	[1.237;1.321	pathogenic strong
-1.165	[-1.166;-1.16	benign supporting
-0.718	[-1.111;-0.12	benign supporting
-1.006	[-1.107;-0.75	benign supporting
-1.139	[-1.169;-1.08	benign supporting
1.231	[1.164;1.310	pathogenic moderate
1.276	[1.133;1.536	pathogenic strong
-0.961	[-0.975;-0.94	benign supporting
-0.670	[-0.679;-0.66	benign supporting
1.160	[1.150;1.171	pathogenic moderate
0.766	[0.012;1.150	pathogenic moderate
-0.657	[-0.852;-0.54	benign supporting
-1.103	[-1.106;-1.10	benign supporting
1.072	[0.991;1.122	pathogenic moderate
-1.166	[-1.170;-1.16	benign supporting
-0.219	[-0.435;0.415	indeterminate
1.276	[0.657;5.090	pathogenic strong
1.276	[1.193;1.388	pathogenic strong

1.276	[1.207;1.367	pathogenic strong
-0.880	[-0.896;-0.86	benign supporting
-1.166	[-1.169;-1.13	benign supporting
-0.736	[-1.067;-0.47	benign supporting
-1.087	[-1.115;-0.85	benign supporting
1.276	[1.236;1.324	pathogenic strong
1.180	[-0.350;5.078	pathogenic moderate
1.276	[1.171;1.431	pathogenic strong
-1.106	[-1.115;-1.09	benign supporting
1.276	[1.022;1.965	pathogenic strong
1.276	[1.208;1.364	pathogenic strong
1.276	[1.094;1.748	pathogenic strong
-0.658	[-0.689;-0.63	benign supporting
1.276	[0.940;3.376	pathogenic strong
-0.200	[-1.161;4.975	indeterminate
-0.233	[-0.358;-0.05	indeterminate
1.276	[0.591;5.091	pathogenic strong
-1.014	[-1.018;-1.01	benign supporting
1.276	[0.000;5.107	pathogenic strong
-0.958	[-1.135;-0.36	benign supporting
0.000	[0.000;0.000	indeterminate
-0.967	[-1.169;-0.83	benign supporting
-1.103	[-1.111;-1.09	benign supporting
-1.134	[-1.169;-1.08	benign supporting
-1.083	[-1.114;-1.00	benign supporting
0.892	[0.707;1.073	pathogenic moderate
1.276	[1.229;1.333	pathogenic strong
1.276	[0.404;5.104	pathogenic strong
1.221	[1.196;1.245	pathogenic moderate
-0.974	[-1.001;-0.94	benign supporting
0.836	[-0.611;4.973	pathogenic moderate
1.265	[1.221;1.313	pathogenic moderate
0.373	[-0.283;1.067	pathogenic supporting
1.276	[1.227;1.333	pathogenic strong
0.316	[-0.109;0.739	indeterminate
1.276	[1.241;1.316	pathogenic strong
-1.167	[-1.169;-1.16	benign supporting
1.030	[1.002;1.059	pathogenic moderate
1.276	[1.003;2.725	pathogenic strong
1.256	[1.224;1.291	pathogenic moderate
1.276	[1.247;1.308	pathogenic strong
1.276	[1.011;2.022	pathogenic strong
1.276	[1.219;1.347	pathogenic strong

-1.089	[-1.092;-1.08	benign supporting
-1.085	[-1.087;-1.08	benign supporting
-1.086	[-1.096;-1.08	benign supporting
0.402	[0.317;0.482	pathogenic supporting
1.149	[1.127;1.172	pathogenic moderate
1.276	[1.231;1.329	pathogenic strong
1.276	[1.118;1.596	pathogenic strong
-1.107	[-1.114;-1.09	benign supporting
-0.948	[-0.961;-0.93	benign supporting
1.276	[1.233;1.327	pathogenic strong
-0.710	[-1.037;-0.46	benign supporting
1.241	[-0.205;5.105	pathogenic moderate
1.276	[1.250;1.305	pathogenic strong
-0.241	[-0.278;-0.19	indeterminate
-0.768	[-0.839;-0.69	benign supporting
-0.447	[-0.807;-0.10	benign supporting
-0.390	[-0.426;-0.35	benign supporting
1.276	[1.245;1.311	pathogenic strong
-0.306	[-0.639;0.645	indeterminate
1.015	[0.966;1.056	pathogenic moderate
1.276	[1.228;1.335	pathogenic strong
-0.073	[-0.261;0.412	indeterminate
-1.081	[-1.097;-1.06	benign supporting
-1.101	[-1.113;-1.09	benign supporting
-0.868	[-0.918;-0.81	benign supporting
-0.919	[-0.923;-0.91	benign supporting
-1.164	[-1.166;-1.16	benign supporting
-0.283	[-0.297;-0.27	indeterminate
-0.459	[-0.591;-0.35	benign supporting
0.725	[0.073;1.108	pathogenic moderate
-1.141	[-1.166;-1.11	benign supporting
-0.550	[-0.561;-0.53	benign supporting
-0.772	[-0.837;-0.70	benign supporting
-0.697	[-0.913;-0.56	benign supporting
-1.102	[-1.108;-1.09	benign supporting
-1.088	[-1.088;-1.08	benign supporting
-1.164	[-1.165;-1.16	benign supporting
1.276	[1.147;1.487	pathogenic strong
1.141	[0.498;1.467	pathogenic moderate
-0.849	[-0.984;-0.68	benign supporting
-0.502	[-0.517;-0.48	benign supporting
-1.131	[-1.145;-1.11	benign supporting
-0.812	[-0.872;-0.73	benign supporting

-1.083	[-1.084;-1.08	benign supporting
1.276	[1.272;1.281	pathogenic strong
1.276	[1.273;1.279	pathogenic strong
1.276	[1.269;1.283	pathogenic strong
1.276	[1.268;1.285	pathogenic strong
-0.953	[-0.954;-0.95	benign supporting
1.276	[1.264;1.289	pathogenic strong
1.276	[1.273;1.280	pathogenic strong
1.276	[1.272;1.280	pathogenic strong
1.276	[1.269;1.283	pathogenic strong
1.276	[1.274;1.278	pathogenic strong
1.276	[1.272;1.281	pathogenic strong
-0.424	[-0.425;-0.42	benign supporting
1.276	[1.270;1.282	pathogenic strong
1.276	[1.266;1.287	pathogenic strong
1.276	[1.263;1.290	pathogenic strong
1.276	[1.268;1.285	pathogenic strong
1.276	[1.272;1.280	pathogenic strong
1.276	[1.273;1.279	pathogenic strong
0.835	[0.829;0.841	pathogenic moderate
-0.495	[-0.506;-0.48	benign supporting
-1.077	[-1.114;-0.87	benign supporting
-0.336	[-1.069;1.12	benign supporting
1.016	[-0.773;5.05	pathogenic moderate
0.850	[0.718;0.997	pathogenic moderate
1.131	[1.106;1.157	pathogenic moderate
-0.913	[-0.918;-0.90	benign supporting
1.181	[1.085;1.277	pathogenic moderate
1.276	[1.211;1.360	pathogenic strong
0.608	[-0.278;1.18	pathogenic supporting
1.276	[1.213;1.357	pathogenic strong
-0.408	[-0.456;-0.36	benign supporting
-0.680	[-0.708;-0.65	benign supporting
-1.061	[-1.065;-1.05	benign supporting
-0.736	[-1.158;0.54	benign supporting
-1.103	[-1.168;-0.89	benign supporting
0.794	[-0.315;1.36	pathogenic moderate
1.234	[-0.115;5.09	pathogenic moderate
-0.892	[-0.914;-0.87	benign supporting
1.276	[1.181;1.412	pathogenic strong
1.167	[0.226;2.151	pathogenic moderate
-0.994	[-1.167;-0.88	benign supporting
-0.941	[-0.972;-0.91	benign supporting

-0.734	[-1.074;-0.44	benign supporting
1.276	[1.033;1.962	pathogenic strong
-1.126	[-1.155;-1.09	benign supporting
1.276	[1.231;1.330	pathogenic strong
-1.110	[-1.167;-0.49	benign supporting
-0.866	[-0.918;-0.83	benign supporting
-1.040	[-1.047;-1.03	benign supporting
1.276	[0.000;5.104	pathogenic strong
-1.098	[-1.115;-1.08	benign supporting
-1.082	[-1.167;0.10	benign supporting
-1.096	[-3.546;0.00	benign supporting
0.706	[0.438;0.909	pathogenic moderate
0.043	[-0.070;0.31	indeterminate
-0.952	[-0.988;-0.92	benign supporting
-0.906	[-0.914;-0.89	benign supporting
1.276	[1.187;1.401	pathogenic strong
0.000	[0.000;0.000	indeterminate
-1.086	[-1.089;-1.08	benign supporting
1.276	[1.223;1.340	pathogenic strong
-1.011	[-1.036;-0.98	benign supporting
1.276	[1.224;1.341	pathogenic strong
1.276	[1.231;1.331	pathogenic strong
-1.084	[-1.086;-1.08	benign supporting
-0.914	[-1.040;-0.73	benign supporting
-0.346	[-1.086;1.18	benign supporting
-0.381	[-0.414;-0.34	benign supporting
1.276	[0.420;5.101	pathogenic strong
-0.474	[-0.516;-0.43	benign supporting
0.118	[-0.070;0.40	indeterminate
-0.837	[-0.842;-0.83	benign supporting
-1.092	[-1.112;-1.08	benign supporting
-1.139	[-1.159;-1.12	benign supporting
1.142	[0.641;1.378	pathogenic moderate
1.276	[0.821;5.062	pathogenic strong
-0.521	[-0.556;-0.48	benign supporting
1.107	[-0.433;5.04	pathogenic moderate
1.276	[1.216;1.351	pathogenic strong
1.276	[1.183;1.411	pathogenic strong
1.203	[0.681;1.820	pathogenic moderate
-0.056	[-0.578;1.15	indeterminate
-1.116	[-1.116;-1.10	benign supporting
1.276	[0.756;5.087	pathogenic strong
-0.785	[-1.112;-0.25	benign supporting

-1.072	[-1.076;-1.06	benign supporting
1.276	[0.541;5.101	pathogenic strong
-0.669	[-0.723;-0.62	benign supporting
1.276	[1.009;2.123	pathogenic strong
1.276	[0.000;5.109	pathogenic strong
1.276	[0.455;5.102	pathogenic strong
-0.856	[-1.000;-0.67	benign supporting
1.276	[-0.356;5.10	pathogenic strong
-0.472	[-0.623;-0.34	benign supporting
-1.158	[-1.169;-1.14	benign supporting
-0.374	[-0.382;-0.36	benign supporting
-1.012	[-1.036;-0.98	benign supporting
-0.589	[-0.856;-0.40	benign supporting
-0.186	[-0.227;-0.14	indeterminate
1.276	[0.841;5.059	pathogenic strong
-1.107	[-1.111;-1.10	benign supporting
-0.968	[-1.000;-0.93	benign supporting
-0.365	[-0.400;-0.32	benign supporting
1.097	[0.702;1.234	pathogenic moderate
0.021	[-0.466;1.14	indeterminate
0.138	[-0.063;0.41	indeterminate
-1.097	[-1.115;-1.08	benign supporting
-0.557	[-0.564;-0.54	benign supporting
-0.424	[-0.495;-0.36	benign supporting
-0.933	[-1.107;-0.57	benign supporting
-1.086	[-1.090;-1.08	benign supporting
1.276	[1.255;1.299	pathogenic strong
1.276	[1.255;1.299	pathogenic strong
0.368	[0.330;0.406	pathogenic supporting
-0.537	[-0.559;-0.51	benign supporting
1.271	[1.251;1.293	pathogenic moderate
-0.056	[-0.100;0.00	indeterminate
-1.005	[-1.017;-0.99	benign supporting
-0.326	[-0.341;-0.30	benign supporting
-0.972	[-1.169;-0.83	benign supporting
1.276	[1.221;1.342	pathogenic strong
-0.518	[-0.561;-0.47	benign supporting
0.972	[0.895;1.034	pathogenic moderate
-0.461	[-0.516;-0.41	benign supporting
1.223	[1.211;1.236	pathogenic moderate
-1.096	[-1.098;-1.09	benign supporting
-0.901	[-0.924;-0.87	benign supporting
-1.085	[-1.094;-1.08	benign supporting

-0.566	[-0.960;-0.27	benign supporting
-1.103	[-1.124;-1.09	benign supporting
-1.125	[-1.133;-1.11	benign supporting
-1.165	[-1.167;-1.16	benign supporting
-0.262	[-0.283;-0.24	indeterminate
-0.361	[-0.383;-0.33	benign supporting
-0.414	[-0.432;-0.39	benign supporting
-0.832	[-0.851;-0.80	benign supporting
-1.089	[-1.091;-1.08	benign supporting
1.276	[1.244;1.312	pathogenic strong
0.396	[0.201;0.557	pathogenic supporting
-1.126	[-1.147;-1.10	benign supporting
-0.925	[-0.934;-0.91	benign supporting
0.411	[-0.415;1.224	pathogenic supporting
-0.242	[-0.381;-0.02	indeterminate
1.276	[0.818;5.070	pathogenic strong
-0.759	[-1.147;0.583	benign supporting
-0.947	[-0.952;-0.94	benign supporting
1.276	[1.193;1.388	pathogenic strong
-0.950	[-0.963;-0.93	benign supporting
0.707	[-0.041;1.123	pathogenic moderate
1.276	[0.280;5.102	pathogenic strong
-0.351	[-0.494;-0.14	benign supporting
1.276	[-0.326;5.106	pathogenic strong
-1.100	[-1.127;-1.08	benign supporting
-0.919	[-0.924;-0.91	benign supporting
-1.013	[-1.013;-1.01	benign supporting
-1.129	[-1.136;-1.12	benign supporting
1.276	[0.257;5.110	pathogenic strong
-1.029	[-1.070;-0.97	benign supporting
-0.585	[-0.613;-0.55	benign supporting
1.276	[1.146;1.501	pathogenic strong
-1.145	[-1.157;-1.13	benign supporting
-0.712	[-0.809;-0.65	benign supporting
-0.359	[-1.089;1.190	benign supporting
1.189	[1.138;1.244	pathogenic moderate
-1.094	[-1.167;-0.69	benign supporting
0.123	[0.009;0.254	indeterminate
-1.070	[-1.166;-0.09	benign supporting
0.033	[-0.194;0.599	indeterminate
-1.045	[-3.555;0.000	benign supporting
-1.091	[-1.168;-0.40	benign supporting
1.276	[0.912;4.956	pathogenic strong

-0.873	[-0.922;-0.83	benign supporting
-1.112	[-1.168;-0.96	benign supporting
-0.910	[-0.970;-0.83	benign supporting
-1.166	[-1.169;-1.16	benign supporting
-0.945	[-0.982;-0.91	benign supporting
1.276	[-0.163;5.10	pathogenic strong
-1.000	[-1.013;-0.98	benign supporting
-0.853	[-1.109;-0.40	benign supporting
-1.060	[-1.067;-1.04	benign supporting
-0.239	[-0.266;-0.20	indeterminate
1.276	[1.251;1.304	pathogenic strong
1.276	[1.222;1.340	pathogenic strong
1.276	[1.251;1.304	pathogenic strong
-0.487	[-0.498;-0.47	benign supporting
1.276	[1.215;1.351	pathogenic strong
1.222	[1.189;1.255	pathogenic moderate
1.276	[1.260;1.294	pathogenic strong
1.276	[1.234;1.324	pathogenic strong
-0.804	[-0.827;-0.78	benign supporting
1.276	[1.241;1.317	pathogenic strong
-0.594	[-0.608;-0.58	benign supporting
-0.638	[-0.648;-0.63	benign supporting
1.276	[1.253;1.301	pathogenic strong
1.276	[1.255;1.298	pathogenic strong
1.131	[1.092;1.164	pathogenic moderate
0.786	[0.727;0.848	pathogenic moderate
0.939	[0.894;0.986	pathogenic moderate
-0.365	[-0.376;-0.35	benign supporting
-1.085	[-1.088;-1.08	benign supporting
-1.111	[-1.167;-1.08	benign supporting
-0.353	[-0.603;0.05	benign supporting
1.193	[-1.111;5.04	pathogenic moderate
-0.538	[-1.069;-0.03	benign supporting
1.276	[-0.389;5.10	pathogenic strong
-1.094	[-1.107;-1.08	benign supporting
1.276	[-1.115;5.01	pathogenic strong
-1.077	[-1.165;-0.53	benign supporting
-0.935	[-0.984;-0.88	benign supporting
-0.366	[-0.509;-0.17	benign supporting
-0.439	[-0.507;-0.38	benign supporting
-0.987	[-1.014;-0.95	benign supporting
0.747	[-0.282;1.27	pathogenic moderate
-1.049	[-1.113;-0.70	benign supporting

-1.065	[-1.081;-1.02	benign supporting
0.826	[-1.144;5.02(	pathogenic moderate
-1.100	[-1.115;-1.08	benign supporting
-0.499	[-0.532;-0.46	benign supporting
-3.812	[-3.827;-0.86	benign strong
-1.086	[-1.115;-1.08	benign supporting
-1.056	[-1.114;-0.63	benign supporting
-3.228	[-3.515;-1.25	benign strong
-0.677	[-1.027;-0.43	benign supporting
-0.538	[-0.586;-0.48	benign supporting
-1.104	[-1.115;-1.06	benign supporting
-0.562	[-0.603;-0.51	benign supporting
1.276	[0.000;5.100]	pathogenic strong
-1.094	[-1.168;-1.01	benign supporting
-1.006	[-1.033;-0.97	benign supporting
-0.987	[-1.015;-0.95	benign supporting
-1.105	[-1.115;-1.08	benign supporting
-0.610	[-0.646;-0.56	benign supporting
-0.751	[-0.789;-0.71	benign supporting
-1.115	[-1.115;-1.06	benign supporting
-0.300	[-0.470;0.00:	indeterminate
1.276	[-1.089;5.075	pathogenic strong
-1.082	[-1.166;-0.36	benign supporting
-1.019	[-1.047;-0.99	benign supporting
-1.104	[-1.123;-1.09	benign supporting
0.323	[-0.348;1.111	pathogenic supporting
-1.052	[-1.067;-1.03	benign supporting
-0.959	[-1.137;-0.41	benign supporting
-1.077	[-1.114;-0.96	benign supporting
-0.166	[-0.228;-0.10	indeterminate
-0.653	[-0.702;-0.61	benign supporting
-0.759	[-0.846;-0.68	benign supporting
-0.098	[-0.175;-0.01	indeterminate
-0.401	[-0.443;-0.36	benign supporting
-0.313	[-0.372;-0.23	indeterminate
0.604	[-0.182;1.138	pathogenic supporting
-1.097	[-1.114;-1.08	benign supporting
-1.087	[-1.092;-1.08	benign supporting
-1.092	[-1.109;-1.08	benign supporting
-0.054	[-0.245;0.41:	indeterminate
-0.579	[-0.603;-0.55	benign supporting
-0.836	[-0.895;-0.75	benign supporting
0.382	[0.112;0.583]	pathogenic supporting

-0.057	[-0.148;0.14(	indeterminate
-0.449	[-0.499;-0.40	benign supporting
-0.973	[-1.015;-0.92	benign supporting
-1.069	[-1.108;-0.97	benign supporting
-1.084	[-1.097;-1.08	benign supporting
-0.185	[-0.253;-0.11	indeterminate
-1.065	[-1.072;-1.04	benign supporting
-1.077	[-1.092;-1.06	benign supporting
-1.071	[-1.084;-1.05	benign supporting
-1.166	[-1.169;-1.16	benign supporting
-0.882	[-0.913;-0.85	benign supporting
0.024	[-0.041;0.187	indeterminate
-0.786	[-1.065;-0.52	benign supporting
-0.200	[-0.292;-0.09	indeterminate
-1.113	[-1.121;-1.10	benign supporting
-1.112	[-1.115;-1.10	benign supporting
-0.392	[-0.410;-0.37	benign supporting
-0.806	[-0.849;-0.75	benign supporting
-0.968	[-1.087;-0.74	benign supporting
1.276	[1.237;1.320	pathogenic strong
-0.920	[-0.992;-0.84	benign supporting
-0.802	[-0.824;-0.78	benign supporting
-0.420	[-0.461;-0.38	benign supporting
-1.085	[-1.089;-1.08	benign supporting
-1.069	[-1.115;-0.68	benign supporting
0.992	[0.841;1.100	pathogenic moderate
1.276	[1.191;1.398	pathogenic strong
1.276	[1.188;1.395	pathogenic strong
1.132	[1.103;1.162	pathogenic moderate
-0.575	[-0.600;-0.55	benign supporting
1.276	[1.226;1.338	pathogenic strong
1.226	[1.182;1.274	pathogenic moderate
-0.455	[-0.492;-0.42	benign supporting
-0.977	[-1.016;-0.93	benign supporting
-1.093	[-1.097;-1.08	benign supporting
1.276	[1.241;1.317	pathogenic strong
-0.499	[-0.512;-0.48	benign supporting
-1.110	[-1.113;-1.10	benign supporting
-0.360	[-0.413;-0.29	benign supporting
1.220	[1.186;1.258	pathogenic moderate
1.276	[1.241;1.315	pathogenic strong
-0.812	[-0.872;-0.73	benign supporting
-0.665	[-0.688;-0.64	benign supporting

1.142	[1.084;1.193	pathogenic moderate
-0.560	[-0.579;-0.53	benign supporting
-1.129	[-1.161;-1.10	benign supporting
-0.311	[-0.396;-0.18	indeterminate
1.276	[1.233;1.325	pathogenic strong
1.276	[1.162;1.455	pathogenic strong
1.276	[1.178;1.420	pathogenic strong
-0.998	[-1.010;-0.98	benign supporting
-0.211	[-0.237;-0.18	indeterminate
1.246	[1.225;1.267	pathogenic moderate
1.276	[1.223;1.340	pathogenic strong
1.276	[1.244;1.313	pathogenic strong
-0.406	[-0.435;-0.37	benign supporting
1.184	[1.124;1.249	pathogenic moderate
-0.969	[-0.984;-0.95	benign supporting
0.935	[0.883;0.990	pathogenic moderate
1.276	[1.119;1.623	pathogenic strong
1.276	[1.259;1.294	pathogenic strong
0.535	[0.354;0.682	pathogenic supporting
-0.106	[-0.179;-0.03	indeterminate
1.276	[1.244;1.312	pathogenic strong
1.276	[1.213;1.356	pathogenic strong
-0.819	[-0.831;-0.80	benign supporting
-1.087	[-1.093;-1.08	benign supporting
-0.843	[-0.885;-0.79	benign supporting
1.276	[1.234;1.325	pathogenic strong
-1.113	[-1.115;-1.00	benign supporting
1.276	[1.103;1.678	pathogenic strong
-1.110	[-1.115;-1.09	benign supporting
1.102	[0.662;1.256	pathogenic moderate
1.276	[1.112;1.624	pathogenic strong
-0.194	[-0.262;-0.11	indeterminate
0.572	[0.484;0.647	pathogenic supporting
-1.081	[-1.091;-1.07	benign supporting
1.239	[1.198;1.282	pathogenic moderate
-1.092	[-1.097;-1.08	benign supporting
-0.848	[-0.885;-0.81	benign supporting
1.276	[0.402;5.094	pathogenic strong
0.808	[0.763;0.855	pathogenic moderate
0.209	[-0.009;0.417	indeterminate
-0.467	[-0.905;-0.08	benign supporting
1.276	[1.168;1.431	pathogenic strong
1.276	[1.234;1.326	pathogenic strong

-0.355	[-0.927;0.843	benign supporting
-1.088	[-1.096;-1.08	benign supporting
-0.937	[-0.956;-0.92	benign supporting
1.276	[1.211;1.363	pathogenic strong
0.903	[0.841;0.969	pathogenic moderate
1.276	[1.241;1.316	pathogenic strong
-0.370	[-0.407;-0.32	benign supporting
1.276	[1.237;1.322	pathogenic strong
1.103	[1.040;1.127	pathogenic moderate
1.276	[1.249;1.305	pathogenic strong
1.276	[1.232;1.326	pathogenic strong
-0.526	[-0.544;-0.50	benign supporting
0.932	[0.860;1.008	pathogenic moderate
-0.548	[-0.585;-0.50	benign supporting
-0.853	[-0.879;-0.82	benign supporting
1.276	[1.203;1.375	pathogenic strong
0.017	[-0.236;0.625	indeterminate
-0.079	[-0.278;0.406	indeterminate
-1.018	[-1.034;-1.00	benign supporting
1.276	[1.208;1.364	pathogenic strong
1.276	[1.227;1.336	pathogenic strong
-1.111	[-1.115;-1.08	benign supporting
-1.090	[-1.136;-1.08	benign supporting
-0.365	[-0.416;-0.30	benign supporting
-0.971	[-1.017;-0.92	benign supporting
-1.086	[-1.090;-1.08	benign supporting
-0.519	[-0.559;-0.48	benign supporting
-1.025	[-1.038;-1.01	benign supporting
-0.940	[-1.021;-0.84	benign supporting
-1.090	[-1.109;-1.08	benign supporting
-0.559	[-0.574;-0.54	benign supporting
-0.588	[-0.613;-0.56	benign supporting
-1.080	[-1.111;-1.03	benign supporting
-0.968	[-0.990;-0.94	benign supporting
-1.094	[-1.104;-1.08	benign supporting
-1.102	[-1.168;-1.08	benign supporting
1.180	[1.151;1.211	pathogenic moderate
-1.099	[-1.103;-1.09	benign supporting
-0.036	[-0.076;0.015	indeterminate
-0.234	[-0.259;-0.20	indeterminate
-0.718	[-0.751;-0.69	benign supporting
-1.167	[-1.169;-1.11	benign supporting
-1.059	[-1.114;-0.66	benign supporting

-0.539	[-0.620;-0.45	benign supporting
-1.104	[-1.169;-1.08	benign supporting
-0.563	[-0.593;-0.52	benign supporting
-0.628	[-0.657;-0.60	benign supporting
-0.063	[-0.208;0.295	indeterminate
-0.923	[-0.939;-0.90	benign supporting
-0.078	[-0.428;0.867	indeterminate
-1.062	[-1.106;-0.94	benign supporting
-1.085	[-1.107;-1.06	benign supporting
-1.061	[-1.110;-0.91	benign supporting
-1.074	[-1.099;-1.05	benign supporting
-0.352	[-0.426;-0.25	benign supporting
1.276	[0.860;5.024]	pathogenic strong
-0.744	[-0.873;-0.63	benign supporting
-0.277	[-0.767;0.992	indeterminate
-1.126	[-1.169;-0.83	benign supporting
-0.986	[-1.025;-0.93	benign supporting
-0.729	[-0.787;-0.68	benign supporting
-0.354	[-0.403;-0.29	benign supporting
-0.889	[-0.910;-0.86	benign supporting
-1.055	[-1.061;-1.04	benign supporting
-1.161	[-1.169;-1.05	benign supporting
-1.022	[-1.033;-1.01	benign supporting
-1.150	[-1.169;-1.13	benign supporting
-1.076	[-1.081;-1.07	benign supporting
-1.087	[-1.089;-1.08	benign supporting
-0.889	[-0.914;-0.86	benign supporting
-0.985	[-0.999;-0.97	benign supporting
-0.892	[-0.919;-0.86	benign supporting
-1.093	[-1.098;-1.09	benign supporting
-1.119	[-1.132;-1.10	benign supporting
-1.072	[-1.079;-1.06	benign supporting
-1.106	[-1.110;-1.10	benign supporting
-0.058	[-0.094;-0.01	indeterminate
-0.853	[-0.866;-0.84	benign supporting
-1.108	[-1.112;-1.10	benign supporting
-1.069	[-1.073;-1.06	benign supporting
-0.697	[-0.731;-0.67	benign supporting
-1.126	[-1.169;-1.01	benign supporting
-1.067	[-1.076;-1.04	benign supporting
-0.981	[-1.085;-0.80	benign supporting
-1.057	[-1.108;-0.92	benign supporting
-1.084	[-1.086;-1.08	benign supporting

-0.895	[-0.938;-0.84	benign supporting
-1.090	[-1.092;-1.08	benign supporting
-1.062	[-1.084;-1.00	benign supporting
-0.477	[-0.497;-0.45	benign supporting
-0.455	[-0.467;-0.44	benign supporting
-1.009	[-1.023;-0.99	benign supporting
-0.901	[-0.915;-0.88	benign supporting
-0.835	[-0.861;-0.80	benign supporting
-1.080	[-1.114;-0.95	benign supporting
-1.105	[-1.108;-1.10	benign supporting
0.480	[0.404;0.555]	pathogenic supporting
-0.865	[-0.892;-0.84	benign supporting
-1.164	[-1.166;-1.16	benign supporting
-0.632	[-0.642;-0.62	benign supporting
-0.819	[-1.066;-0.56	benign supporting
-0.955	[-0.975;-0.93	benign supporting
-1.090	[-1.092;-1.08	benign supporting
-0.964	[-1.046;-0.87	benign supporting
-1.090	[-1.114;-1.08	benign supporting
-0.557	[-0.581;-0.52	benign supporting
-1.017	[-1.034;-1.00	benign supporting
-1.099	[-1.110;-1.09	benign supporting
-0.311	[-0.361;-0.24	indeterminate
-1.076	[-1.090;-1.05	benign supporting
-1.089	[-1.093;-1.08	benign supporting
-0.863	[-0.903;-0.82	benign supporting
-1.092	[-1.096;-1.08	benign supporting
-1.097	[-1.106;-1.08	benign supporting
-0.900	[-0.925;-0.87	benign supporting
-0.530	[-0.648;-0.41	benign supporting
-1.002	[-1.027;-0.96	benign supporting
0.466	[0.337;0.585]	pathogenic supporting
-0.390	[-0.558;-0.18	benign supporting
0.396	[0.306;0.479]	pathogenic supporting
-1.088	[-1.096;-1.08	benign supporting
-0.923	[-0.942;-0.90	benign supporting
-0.901	[-0.935;-0.85	benign supporting
-0.601	[-0.910;-0.39	benign supporting
-1.043	[-1.066;-1.01	benign supporting
-0.474	[-0.507;-0.44	benign supporting
-0.652	[-0.670;-0.63	benign supporting
-1.084	[-1.087;-1.08	benign supporting
-0.618	[-0.677;-0.56	benign supporting

-0.455	[-1.005;0.287	benign supporting
-0.624	[-0.638;-0.61	benign supporting
-1.061	[-1.068;-1.04	benign supporting
-1.093	[-1.103;-1.08	benign supporting
-1.097	[-1.103;-1.09	benign supporting
-1.101	[-1.106;-1.09	benign supporting
1.276	[1.137;1.548	pathogenic strong
-0.898	[-0.932;-0.85	benign supporting
-0.127	[-0.195;-0.06	indeterminate
-0.940	[-0.955;-0.92	benign supporting
-0.862	[-0.896;-0.83	benign supporting
-0.849	[-0.861;-0.83	benign supporting
-1.027	[-1.040;-1.01	benign supporting
-0.953	[-0.958;-0.94	benign supporting
-0.488	[-0.566;-0.41	benign supporting
-0.315	[-0.334;-0.29	indeterminate
-1.135	[-1.149;-1.12	benign supporting
1.276	[0.000;5.096	pathogenic strong
-0.660	[-0.669;-0.65	benign supporting
-3.481	[-3.497;-3.46	benign strong
1.060	[1.012;1.105	pathogenic moderate
0.610	[0.512;0.702	pathogenic supporting
0.225	[-0.046;0.507	indeterminate
-1.168	[-1.169;-1.14	benign supporting
-0.178	[-0.201;-0.15	indeterminate
1.240	[1.211;1.271	pathogenic moderate
1.276	[-0.161;5.102	pathogenic strong
-0.705	[-0.791;-0.64	benign supporting
-0.188	[-0.619;1.005	indeterminate
-0.386	[-0.604;-0.09	benign supporting
-0.299	[-0.312;-0.28	indeterminate
-1.008	[-1.014;-1.00	benign supporting
1.276	[1.234;1.326	pathogenic strong
-0.915	[-0.932;-0.89	benign supporting
-0.829	[-0.963;-0.65	benign supporting
-1.164	[-1.169;-1.12	benign supporting
-0.151	[-0.178;-0.12	indeterminate
-0.671	[-1.106;-0.16	benign supporting
-0.465	[-1.166;4.799	benign supporting
1.276	[0.875;5.035	pathogenic strong
-0.514	[-0.899;-0.22	benign supporting
-1.112	[-1.116;-1.10	benign supporting
-1.116	[-1.169;-1.08	benign supporting

-0.392	[-1.168;4.932	benign supporting
-0.974	[-1.152;-0.38	benign supporting
-0.837	[-0.845;-0.83	benign supporting
1.276	[1.251;1.303]	pathogenic strong
-0.928	[-0.933;-0.92	benign supporting
-1.089	[-1.093;-1.08	benign supporting
-1.093	[-1.114;-1.08	benign supporting
-1.109	[-1.139;-1.07	benign supporting
-1.150	[-1.152;-1.14	benign supporting
-1.157	[-1.161;-1.15	benign supporting
-1.110	[-1.111;-1.10	benign supporting
-0.552	[-0.555;-0.55	benign supporting
-0.874	[-0.885;-0.86	benign supporting
-0.710	[-0.721;-0.69	benign supporting
-0.814	[-0.823;-0.80	benign supporting
-0.811	[-0.814;-0.80	benign supporting
-0.945	[-0.949;-0.94	benign supporting
-1.028	[-1.029;-1.02	benign supporting
-1.115	[-1.115;-1.11	benign supporting
-0.997	[-1.002;-0.99	benign supporting
-1.107	[-1.110;-1.10	benign supporting
1.276	[1.266;1.287]	pathogenic strong
-0.673	[-0.901;-0.53	benign supporting
-1.107	[-1.108;-1.10	benign supporting
-0.579	[-0.581;-0.57	benign supporting
-1.129	[-1.168;-1.10	benign supporting
-1.000	[-1.007;-0.99	benign supporting
-1.086	[-1.168;-0.73	benign supporting
1.138	[-0.107;4.346	pathogenic moderate
-1.055	[-1.095;-0.95	benign supporting
0.522	[0.352;0.664]	pathogenic supporting
0.449	[-0.340;1.157	pathogenic supporting
0.647	[-1.004;4.993	pathogenic moderate
1.276	[-0.319;5.102	pathogenic strong
0.346	[-0.242;0.990	pathogenic supporting
-1.086	[-1.100;-1.08	benign supporting
-1.020	[-1.116;-0.52	benign supporting
-1.110	[-1.115;-1.10	benign supporting
-0.907	[-0.911;-0.90	benign supporting
1.276	[0.630;5.097]	pathogenic strong
0.756	[0.187;1.106]	pathogenic moderate
-0.953	[-2.865;-0.83	benign supporting
-1.166	[-1.169;-1.16	benign supporting

0.133	[-0.123;0.564	indeterminate
-0.510	[-0.961;-0.12	benign supporting
-0.986	[-1.034;-0.95	benign supporting
-0.974	[-1.018;-0.95	benign supporting
-1.091	[-1.164;-0.63	benign supporting
1.276	[1.133;1.540]	pathogenic strong
-0.681	[-1.028;-0.44	benign supporting
-1.092	[-1.165;-1.02	benign supporting
-1.085	[-1.087;-1.08	benign supporting
-1.129	[-1.168;-1.08	benign supporting
-1.016	[-1.047;-0.98	benign supporting
-1.164	[-1.168;-0.92	benign supporting
0.003	[-0.036;0.077	indeterminate
-1.020	[-1.030;-1.01	benign supporting
1.231	[1.142;1.341]	pathogenic moderate
-1.131	[-1.169;-1.09	benign supporting
0.928	[0.002;1.266]	pathogenic moderate
0.487	[-0.237;1.108	pathogenic supporting
-1.084	[-1.086;-1.08	benign supporting
1.276	[1.183;1.412]	pathogenic strong
1.276	[0.935;4.763]	pathogenic strong
-0.725	[-0.752;-0.70	benign supporting
-0.737	[-0.791;-0.69	benign supporting
-0.856	[-0.903;-0.80	benign supporting
-0.990	[-1.075;-0.85	benign supporting
-0.588	[-0.608;-0.56	benign supporting
-0.979	[-1.092;-0.77	benign supporting
-0.659	[-0.793;-0.57	benign supporting
-0.722	[-0.803;-0.66	benign supporting
-0.736	[-0.762;-0.71	benign supporting
-1.110	[-1.115;-1.10	benign supporting
-0.582	[-0.629;-0.53	benign supporting
-0.615	[-0.621;-0.61	benign supporting
-0.079	[-0.131;-0.01	indeterminate
1.222	[1.170;1.279]	pathogenic moderate
-0.903	[-0.934;-0.86	benign supporting
1.103	[-0.198;2.056	pathogenic moderate
-1.112	[-1.116;-1.10	benign supporting
-0.624	[-0.632;-0.61	benign supporting
-0.509	[-0.526;-0.49	benign supporting
-1.003	[-1.023;-0.98	benign supporting
-0.619	[-0.846;-0.45	benign supporting
-1.052	[-1.166;-0.21	benign supporting

-0.531	[-0.890;-0.25	benign supporting
-1.050	[-1.065;-1.03	benign supporting
-3.544	[-3.768;-2.91	benign strong
-0.262	[-0.342;-0.14	indeterminate
-0.953	[-1.024;-0.90	benign supporting
-0.566	[-1.164;1.657	benign supporting
0.000	[-3.061;0.000	indeterminate
1.276	[1.018;2.049]	pathogenic strong
-1.068	[-1.167;-0.99	benign supporting
0.559	[-0.078;1.006	pathogenic supporting
-0.905	[-0.929;-0.87	benign supporting
-0.414	[-0.476;-0.35	benign supporting
1.276	[1.137;1.530]	pathogenic strong
-0.960	[-1.103;-0.66	benign supporting
-0.829	[-0.853;-0.79	benign supporting
1.276	[-0.205;5.108	pathogenic strong
-0.413	[-0.490;-0.34	benign supporting
-1.094	[-1.108;-1.08	benign supporting
-1.092	[-1.167;-0.63	benign supporting
-1.146	[-1.169;-1.08	benign supporting
-0.735	[-0.844;-0.65	benign supporting
-0.521	[-0.662;-0.38	benign supporting
-1.156	[-1.169;-1.13	benign supporting
-0.787	[-0.850;-0.71	benign supporting
-0.563	[-0.583;-0.54	benign supporting
-0.438	[-0.483;-0.40	benign supporting
-0.666	[-1.077;-0.36	benign supporting
-1.011	[-1.027;-0.99	benign supporting
-0.716	[-0.812;-0.64	benign supporting
-1.087	[-1.091;-1.08	benign supporting
-0.344	[-0.378;-0.30	benign supporting
-1.089	[-1.096;-1.08	benign supporting
-0.849	[-0.890;-0.80	benign supporting
-0.609	[-0.682;-0.53	benign supporting
-0.434	[-0.899;0.119	benign supporting
1.276	[-1.103;5.047	pathogenic strong
-0.588	[-0.628;-0.54	benign supporting
-0.666	[-0.686;-0.64	benign supporting
-0.869	[-0.896;-0.84	benign supporting
-1.085	[-1.168;-1.07	benign supporting
-1.109	[-1.115;-1.09	benign supporting
-0.818	[-0.840;-0.78	benign supporting
0.470	[-0.076;0.876	pathogenic supporting

-1.089	[-1.111;-1.08	benign supporting
0.327	[0.242;0.399]	pathogenic supporting
0.361	[0.247;0.459]	pathogenic supporting
-0.951	[-0.959;-0.94	benign supporting
1.276	[0.608;5.087]	pathogenic strong
-0.666	[-0.955;-0.46	benign supporting
-0.946	[-0.974;-0.92	benign supporting
-0.391	[-0.535;-0.23	benign supporting
-0.306	[-0.394;-0.17	indeterminate
1.252	[1.220;1.287]	pathogenic moderate
-0.527	[-0.552;-0.50	benign supporting
-0.294	[-0.326;-0.25	indeterminate
-0.083	[-0.411;0.848	indeterminate
1.276	[1.119;1.594]	pathogenic strong
-0.455	[-0.574;-0.36	benign supporting
1.276	[1.097;1.708]	pathogenic strong
-0.391	[-0.416;-0.36	benign supporting
1.035	[0.979;1.090]	pathogenic moderate
-0.969	[-0.987;-0.95	benign supporting
-0.853	[-0.932;-0.75	benign supporting
-0.999	[-1.072;-0.88	benign supporting
-1.098	[-1.167;-0.86	benign supporting
-0.950	[-0.965;-0.93	benign supporting
-0.931	[-0.950;-0.91	benign supporting
-0.937	[-0.956;-0.91	benign supporting
-1.081	[-1.095;-1.06	benign supporting
-1.073	[-1.085;-1.05	benign supporting
-1.099	[-1.113;-1.08	benign supporting
-0.641	[-0.667;-0.62	benign supporting
-0.578	[-0.599;-0.56	benign supporting
-0.689	[-0.741;-0.65	benign supporting
0.012	[-0.043;0.141	indeterminate
-0.573	[-0.591;-0.55	benign supporting
-1.102	[-1.105;-1.09	benign supporting
-0.290	[-0.342;-0.22	indeterminate
-0.793	[-0.833;-0.74	benign supporting
-1.068	[-1.072;-1.06	benign supporting
-1.114	[-1.116;-1.10	benign supporting
-0.256	[-0.377;-0.06	indeterminate
-1.089	[-1.103;-1.08	benign supporting
-1.082	[-1.114;-0.98	benign supporting
-1.112	[-1.116;-1.10	benign supporting
-1.089	[-3.077;4.500	benign supporting

-1.087	[-1.089;-1.08	benign supporting
-1.071	[-1.077;-1.06	benign supporting
-1.164	[-1.168;-1.16	benign supporting
-0.829	[-0.852;-0.79	benign supporting
-0.425	[-0.502;-0.36	benign supporting
-0.479	[-0.549;-0.41	benign supporting
-1.098	[-1.100;-1.09	benign supporting
-1.042	[-1.071;-1.00	benign supporting
-1.100	[-1.109;-1.09	benign supporting
-0.617	[-0.627;-0.60	benign supporting
1.276	[1.227;1.335	pathogenic strong
-1.084	[-1.085;-1.08	benign supporting
0.502	[0.091;0.774	pathogenic supporting
-1.102	[-1.111;-1.09	benign supporting
-1.110	[-1.116;-1.10	benign supporting
-1.046	[-1.053;-1.04	benign supporting
-0.338	[-0.370;-0.30	benign supporting
-0.953	[-1.052;-0.83	benign supporting
1.136	[0.988;1.221	pathogenic moderate
1.276	[1.235;1.326	pathogenic strong
-0.274	[-0.313;-0.23	indeterminate
1.276	[1.217;1.347	pathogenic strong
0.248	[0.109;0.357	indeterminate
1.276	[1.241;1.315	pathogenic strong
1.041	[-0.459;5.02	pathogenic moderate
1.276	[1.225;1.337	pathogenic strong
1.276	[1.200;1.379	pathogenic strong
0.797	[0.694;0.906	pathogenic moderate
1.276	[1.254;1.298	pathogenic strong
1.276	[1.229;1.331	pathogenic strong
1.276	[1.210;1.357	pathogenic strong
1.242	[1.159;1.342	pathogenic moderate
1.276	[1.233;1.327	pathogenic strong
1.276	[1.153;1.482	pathogenic strong
1.276	[1.196;1.383	pathogenic strong
1.276	[1.219;1.347	pathogenic strong
1.276	[1.214;1.355	pathogenic strong
1.276	[1.256;1.297	pathogenic strong
-1.105	[-1.164;-0.76	benign supporting
-0.045	[-0.251;0.50	indeterminate
-0.728	[-0.752;-0.70	benign supporting
-1.164	[-1.169;-1.15	benign supporting
-1.097	[-1.108;-1.08	benign supporting

0.741	[0.697;0.784	pathogenic moderate
-0.677	[-0.837;-0.58	benign supporting
-1.113	[-1.116;-1.11	benign supporting
-1.086	[-1.102;-1.08	benign supporting
-1.094	[-1.115;-1.04	benign supporting
0.551	[0.469;0.616	pathogenic supporting
-1.168	[-1.169;-1.16	benign supporting
1.276	[1.263;1.290	pathogenic strong
0.271	[-0.076;0.62	indeterminate
1.276	[1.229;1.331	pathogenic strong
-0.140	[-0.178;-0.10	indeterminate
0.590	[0.572;0.607	pathogenic supporting
-1.095	[-1.099;-1.09	benign supporting
-0.999	[-1.020;-0.96	benign supporting
1.276	[1.016;2.113	pathogenic strong
1.276	[1.207;1.367	pathogenic strong
1.276	[1.192;1.390	pathogenic strong
1.276	[1.114;1.614	pathogenic strong
1.276	[0.000;5.103	pathogenic strong
1.248	[1.171;1.340	pathogenic moderate
0.439	[-0.420;1.24	pathogenic supporting
-1.169	[-1.169;-1.08	benign supporting
-1.105	[-1.111;-1.09	benign supporting
-1.146	[-1.169;-1.05	benign supporting
-1.122	[-1.164;-1.09	benign supporting
1.276	[-1.098;5.04	pathogenic strong
0.000	[0.000;0.000	indeterminate
0.466	[-0.401;1.23	pathogenic supporting
-0.709	[-0.732;-0.68	benign supporting
1.276	[1.240;1.319	pathogenic strong
-0.963	[-1.066;-0.84	benign supporting
1.276	[1.241;1.317	pathogenic strong
1.276	[1.156;1.467	pathogenic strong
1.004	[-0.567;5.02	pathogenic moderate
1.171	[1.139;1.205	pathogenic moderate
-0.420	[-0.884;0.21	benign supporting
-0.469	[-0.495;-0.44	benign supporting
-1.043	[-1.062;-1.02	benign supporting
-0.251	[-0.268;-0.23	indeterminate
-0.636	[-0.667;-0.61	benign supporting
-0.901	[-0.906;-0.89	benign supporting
-1.095	[-1.101;-1.09	benign supporting
-1.049	[-1.061;-1.03	benign supporting

-1.028	[-1.059;-0.99	benign supporting
-1.115	[-1.116;-0.96	benign supporting
-0.917	[-1.166;1.04(	benign supporting
-1.084	[-1.085;-1.08	benign supporting
1.276	[1.121;1.590]	pathogenic strong
-1.101	[-1.115;-1.07	benign supporting
1.276	[1.253;1.302]	pathogenic strong
-0.236	[-0.353;-0.06	indeterminate
-0.113	[-0.254;0.10:	indeterminate
-1.112	[-1.132;-1.09	benign supporting
-0.830	[-1.160;0.14§	benign supporting
-1.109	[-1.116;-1.09	benign supporting
-1.129	[-1.152;-1.11	benign supporting
1.276	[1.252;1.303]	pathogenic strong
-0.610	[-0.618;-0.60	benign supporting
1.138	[1.126;1.149]	pathogenic moderate
0.462	[0.373;0.549]	pathogenic supporting
-0.354	[-0.362;-0.34	benign supporting
-1.116	[-1.124;-1.10	benign supporting
1.199	[1.146;1.257]	pathogenic moderate
-1.071	[-2.993;1.507	benign supporting
-1.090	[-1.110;-1.08	benign supporting
-1.081	[-1.096;-1.06	benign supporting
-0.368	[-0.383;-0.35	benign supporting
1.276	[1.228;1.333]	pathogenic strong
0.978	[0.935;1.011]	pathogenic moderate
1.276	[1.255;1.298]	pathogenic strong
-0.300	[-0.516;0.197	indeterminate
-0.734	[-0.745;-0.72	benign supporting
-0.950	[-0.961;-0.94	benign supporting
-1.085	[-1.106;-1.08	benign supporting
-1.074	[-1.165;-0.48	benign supporting
-0.592	[-0.921;-0.37	benign supporting
-0.319	[-0.384;-0.23	benign supporting
-1.097	[-1.127;-1.08	benign supporting
1.049	[0.933;1.121]	pathogenic moderate
-1.100	[-1.112;-1.09	benign supporting
-1.081	[-1.091;-1.07	benign supporting
-0.209	[-0.296;-0.10	indeterminate
-1.109	[-1.115;-1.05	benign supporting
-1.164	[-1.169;-1.09	benign supporting
-1.088	[-1.168;-0.97	benign supporting
-1.084	[-1.165;-1.08	benign supporting

0.000	[-3.388;0.00(	indeterminate
0.005	[-0.256;0.64(	indeterminate
-1.168	[-1.169;-0.96	benign supporting
1.276	[1.205;1.371]	pathogenic strong
-1.114	[-1.166;-0.64	benign supporting
-0.516	[-0.534;-0.49	benign supporting
1.276	[0.894;4.992]	pathogenic strong
-0.424	[-0.823;-0.02	benign supporting
-1.095	[-1.167;-0.45	benign supporting
1.276	[-0.330;5.10(	pathogenic strong
-1.095	[-1.168;-1.08	benign supporting
-0.607	[-0.639;-0.56	benign supporting
1.276	[1.202;1.369]	pathogenic strong
-0.828	[-1.082;-0.53	benign supporting
-0.897	[-0.916;-0.87	benign supporting
-0.847	[-0.870;-0.83	benign supporting
-0.385	[-0.855;0.49!	benign supporting
1.276	[-0.145;5.10;	pathogenic strong
-1.131	[-1.154;-1.11	benign supporting
-1.086	[-1.167;-1.08	benign supporting
1.276	[1.177;1.418]	pathogenic strong
1.276	[1.198;1.382]	pathogenic strong
-1.166	[-1.168;-0.84	benign supporting
0.509	[-0.153;1.03(	pathogenic supporting
1.276	[0.000;5.100]	pathogenic strong
-1.111	[-1.168;-0.99	benign supporting
-1.150	[-1.169;-1.13	benign supporting
-1.088	[-1.167;-0.90	benign supporting
-0.521	[-0.586;-0.45	benign supporting
-1.084	[-1.164;-1.08	benign supporting
-0.015	[-0.126;0.281	indeterminate
1.195	[1.134;1.262]	pathogenic moderate
-0.612	[-0.642;-0.57	benign supporting
1.276	[1.166;1.451]	pathogenic strong
1.276	[1.184;1.407]	pathogenic strong
0.000	[-3.779;0.00(	indeterminate
-0.357	[-0.390;-0.32	benign supporting
1.276	[1.249;1.305]	pathogenic strong
1.128	[0.312;1.492]	pathogenic moderate
-1.102	[-1.114;-1.09	benign supporting
-1.069	[-1.083;-1.04	benign supporting
1.276	[1.200;1.373]	pathogenic strong
-1.057	[-1.070;-1.03	benign supporting

-1.090	[-1.114;-1.06	benign supporting
0.996	[0.434;1.215]	pathogenic moderate
-0.123	[-0.277;0.135	indeterminate
1.131	[0.702;1.310]	pathogenic moderate
-1.083	[-1.090;-1.07	benign supporting
-0.943	[-0.952;-0.93	benign supporting
-0.791	[-0.907;-0.66	benign supporting
-0.346	[-1.165;4.931	benign supporting
-0.366	[-0.420;-0.30	benign supporting
-0.059	[-0.404;0.886	indeterminate
1.276	[0.846;5.039]	pathogenic strong
-0.567	[-0.604;-0.52	benign supporting
-0.443	[-0.497;-0.40	benign supporting
-1.085	[-1.092;-1.08	benign supporting
-0.844	[-0.883;-0.83	benign supporting
-0.518	[-0.561;-0.47	benign supporting
-0.957	[-1.025;-0.88	benign supporting
-0.494	[-1.095;0.753	benign supporting
-1.097	[-1.111;-1.08	benign supporting
0.947	[0.352;1.202]	pathogenic moderate
-1.036	[-1.072;-1.00	benign supporting
1.276	[0.755;5.079]	pathogenic strong
-0.486	[-0.602;-0.38	benign supporting
1.276	[0.987;2.255]	pathogenic strong
-0.920	[-0.989;-0.84	benign supporting
1.276	[1.075;1.760]	pathogenic strong
-1.099	[-1.114;-1.08	benign supporting
-0.768	[-0.851;-0.68	benign supporting
-1.098	[-1.109;-1.09	benign supporting
-0.768	[-0.848;-0.69	benign supporting
-0.686	[-0.844;-0.59	benign supporting
-1.111	[-1.115;-1.08	benign supporting
-0.623	[-0.712;-0.54	benign supporting
-1.107	[-1.115;-1.08	benign supporting
-0.739	[-0.814;-0.68	benign supporting
1.139	[1.117;1.160]	pathogenic moderate
-0.577	[-0.616;-0.53	benign supporting
-1.165	[-1.170;-1.16	benign supporting
-1.110	[-1.115;-1.08	benign supporting
-0.985	[-1.005;-0.96	benign supporting
-0.196	[-0.257;-0.12	indeterminate
1.276	[1.045;1.930]	pathogenic strong
-0.621	[-0.715;-0.54	benign supporting

1.129	[1.106;1.151]	pathogenic moderate
-1.090	[-1.102;-1.08	benign supporting
-1.106	[-1.110;-1.10	benign supporting
-1.088	[-1.092;-1.08	benign supporting
0.010	[-0.288;0.72(	indeterminate
-1.099	[-1.112;-1.08	benign supporting
-1.093	[-1.105;-1.08	benign supporting
-0.682	[-0.707;-0.66	benign supporting
-1.164	[-1.168;-0.85	benign supporting
1.105	[0.711;1.244]	pathogenic moderate
-0.970	[-0.989;-0.95	benign supporting
-0.867	[-0.886;-0.84	benign supporting
-0.473	[-0.489;-0.45	benign supporting
-0.617	[-0.650;-0.58	benign supporting
-0.957	[-1.019;-0.89	benign supporting
-1.092	[-1.110;-1.08	benign supporting
-1.110	[-1.155;-1.08	benign supporting
-1.017	[-1.032;-1.00	benign supporting
-1.056	[-1.068;-1.03	benign supporting
-0.554	[-0.586;-0.51	benign supporting
1.276	[1.247;1.309]	pathogenic strong
-1.130	[-1.169;-1.09	benign supporting
-1.089	[-1.092;-1.08	benign supporting
-0.951	[-1.030;-0.86	benign supporting
-1.085	[-1.090;-1.08	benign supporting
-1.113	[-1.115;-1.06	benign supporting
-0.502	[-1.168;4.873	benign supporting
-1.166	[-1.169;-1.15	benign supporting
-1.133	[-1.169;-1.08	benign supporting
-0.924	[-2.829;4.743	benign supporting
-1.099	[-1.168;-0.94	benign supporting
-0.944	[-0.945;-0.94	benign supporting
1.190	[1.141;1.243]	pathogenic moderate
1.276	[1.134;1.537]	pathogenic strong
1.276	[-0.148;5.104	pathogenic strong
1.276	[1.120;1.598]	pathogenic strong
1.276	[0.966;2.482]	pathogenic strong
1.276	[1.236;1.321]	pathogenic strong
1.276	[1.155;1.473]	pathogenic strong
1.101	[0.948;1.157]	pathogenic moderate
0.640	[0.538;0.729]	pathogenic moderate
-1.102	[-1.111;-1.09	benign supporting
-0.783	[-0.822;-0.74	benign supporting

1.276	[1.149;1.486	pathogenic strong
-1.143	[-1.151;-1.13	benign supporting
1.110	[0.687;1.267	pathogenic moderate
1.276	[1.228;1.333	pathogenic strong
1.276	[1.241;1.317	pathogenic strong
0.844	[0.678;1.014	pathogenic moderate
0.890	[0.747;1.033	pathogenic moderate
-1.100	[-1.106;-1.09	benign supporting
0.392	[-0.288;1.10	pathogenic supporting
0.059	[-1.166;4.94	indeterminate
-0.446	[-0.501;-0.40	benign supporting
1.276	[1.253;1.302	pathogenic strong
0.302	[0.095;0.453	indeterminate
-0.400	[-0.433;-0.37	benign supporting
-0.217	[-0.254;-0.17	indeterminate
1.111	[1.035;1.146	pathogenic moderate
-0.594	[-0.653;-0.53	benign supporting
-0.687	[-0.750;-0.64	benign supporting
-1.091	[-1.099;-1.08	benign supporting
-1.036	[-1.107;-0.87	benign supporting
0.312	[0.099;0.470	indeterminate
-0.724	[-0.784;-0.67	benign supporting
-1.104	[-1.115;-1.06	benign supporting
-0.246	[-0.280;-0.20	indeterminate
1.276	[1.218;1.347	pathogenic strong
-1.089	[-1.128;-1.08	benign supporting
-0.947	[-0.965;-0.93	benign supporting
-0.511	[-0.561;-0.46	benign supporting
-1.135	[-1.167;-1.11	benign supporting
1.118	[0.967;1.186	pathogenic moderate
1.276	[1.249;1.307	pathogenic strong
1.276	[1.239;1.318	pathogenic strong
1.208	[1.178;1.239	pathogenic moderate
1.109	[1.084;1.122	pathogenic moderate
1.142	[1.117;1.166	pathogenic moderate
1.276	[1.245;1.311	pathogenic strong
1.276	[1.245;1.311	pathogenic strong
1.276	[1.227;1.335	pathogenic strong
0.917	[0.761;1.059	pathogenic moderate
1.276	[1.223;1.340	pathogenic strong
1.276	[1.172;1.428	pathogenic strong
1.276	[1.228;1.335	pathogenic strong
1.276	[1.153;1.473	pathogenic strong

1.149	[0.829;1.303]	pathogenic moderate
0.963	[0.913;1.007]	pathogenic moderate
1.276	[1.189;1.397]	pathogenic strong
-0.916	[-0.925;-0.90	benign supporting
-0.608	[-0.621;-0.59	benign supporting
1.276	[1.246;1.310]	pathogenic strong
-1.081	[-1.086;-1.07	benign supporting
-1.104	[-1.115;-1.07	benign supporting
1.276	[-0.410;5.097	pathogenic strong
-0.448	[-0.575;-0.35	benign supporting
1.276	[0.891;5.029]	pathogenic strong
1.276	[1.130;1.547]	pathogenic strong
-0.570	[-0.825;-0.38	benign supporting
-0.565	[-0.592;-0.53	benign supporting
0.266	[-0.082;0.622	indeterminate
-1.115	[-1.115;-1.08	benign supporting
-0.828	[-0.869;-0.76	benign supporting
-0.318	[-0.541;0.145	indeterminate
1.276	[-1.137;4.988	pathogenic strong
0.109	[-0.375;1.042	indeterminate
-0.260	[-0.309;-0.20	indeterminate
-0.916	[-1.073;-0.66	benign supporting
1.276	[1.107;1.654]	pathogenic strong
-1.069	[-1.096;-1.00	benign supporting
1.276	[1.191;1.389]	pathogenic strong
1.276	[0.766;5.078]	pathogenic strong
-0.597	[-0.917;-0.38	benign supporting
-1.106	[-1.115;-1.08	benign supporting
-1.068	[-1.073;-1.05	benign supporting
-0.867	[-0.919;-0.81	benign supporting
-0.253	[-0.289;-0.21	indeterminate
0.371	[0.207;0.512]	pathogenic supporting
-0.039	[-0.093;0.047	indeterminate
-0.958	[-0.978;-0.93	benign supporting
-0.212	[-0.276;-0.13	indeterminate
-1.094	[-1.106;-1.08	benign supporting
-1.032	[-1.045;-1.01	benign supporting
-0.461	[-0.477;-0.44	benign supporting
-0.546	[-0.647;-0.43	benign supporting
-1.042	[-1.079;-0.97	benign supporting
-0.716	[-0.790;-0.66	benign supporting
-1.161	[-1.170;-1.15	benign supporting
-0.782	[-1.022;-0.56	benign supporting

1.273	[1.195;1.379	pathogenic strong
-1.068	[-1.098;-1.00	benign supporting
-0.581	[-0.639;-0.51	benign supporting
1.265	[1.236;1.296	pathogenic moderate
-0.142	[-0.169;-0.11	indeterminate
-1.104	[-1.115;-1.05	benign supporting
-1.077	[-1.090;-1.06	benign supporting
-1.106	[-1.112;-1.10	benign supporting
-1.096	[-1.102;-1.09	benign supporting
-1.038	[-1.056;-1.02	benign supporting
-1.088	[-1.100;-1.08	benign supporting
-1.013	[-1.051;-0.96	benign supporting
-1.152	[-1.170;-1.12	benign supporting
-0.791	[-0.911;-0.66	benign supporting
-1.100	[-1.105;-1.09	benign supporting
-0.814	[-0.848;-0.76	benign supporting
-1.113	[-1.115;-1.07	benign supporting
-1.090	[-1.101;-1.08	benign supporting
-0.072	[-0.160;0.064	indeterminate
-1.084	[-1.086;-1.08	benign supporting
-1.094	[-1.096;-1.09	benign supporting
1.116	[1.048;1.152	pathogenic moderate
-1.098	[-1.101;-1.09	benign supporting
-1.021	[-1.051;-0.99	benign supporting
-0.311	[-0.340;-0.27	indeterminate
-1.166	[-1.170;-1.14	benign supporting
-0.939	[-1.104;-0.62	benign supporting
-1.075	[-1.080;-1.07	benign supporting
0.942	[0.856;1.021	pathogenic moderate
-0.090	[-0.127;-0.05	indeterminate
1.109	[1.078;1.125	pathogenic moderate
-0.922	[-0.951;-0.88	benign supporting
-0.245	[-0.355;-0.08	indeterminate
0.536	[0.459;0.603	pathogenic supporting
-0.825	[-0.833;-0.81	benign supporting
1.276	[1.245;1.311	pathogenic strong
1.245	[1.167;1.336	pathogenic moderate
1.130	[0.755;1.295	pathogenic moderate
1.276	[1.237;1.320	pathogenic strong
-0.837	[-0.871;-0.79	benign supporting
-0.936	[-1.053;-0.79	benign supporting
1.276	[-0.901;5.081	pathogenic strong
-1.114	[-1.115;-1.07	benign supporting

-0.942	[-0.979;-0.90	benign supporting
-0.623	[-0.637;-0.61	benign supporting
-0.834	[-0.868;-0.78	benign supporting
-1.104	[-1.116;-0.90	benign supporting
-0.631	[-0.949;-0.41	benign supporting
-1.092	[-1.115;-1.08	benign supporting
1.276	[-1.130;4.992	pathogenic strong
-0.127	[-1.104;4.702	indeterminate
1.161	[1.054;1.251]	pathogenic moderate
-1.164	[-1.169;-1.07	benign supporting
-0.421	[-1.166;4.885	benign supporting
0.600	[-0.413;1.317	pathogenic supporting
-1.087	[-1.098;-1.08	benign supporting
-1.168	[-1.169;-1.02	benign supporting
-0.912	[-1.112;-0.41	benign supporting
1.183	[1.045;1.306]	pathogenic moderate
-0.924	[-1.095;-0.62	benign supporting
-0.957	[-1.096;-0.68	benign supporting
-0.547	[-0.687;-0.41	benign supporting
-1.032	[-1.113;-0.63	benign supporting
-1.143	[-1.169;-1.09	benign supporting
1.276	[-1.136;5.007	pathogenic strong
-0.381	[-0.586;-0.11	benign supporting
-1.114	[-1.116;-1.10	benign supporting
-1.088	[-1.090;-1.08	benign supporting
-0.790	[-0.897;-0.67	benign supporting
-1.047	[-1.063;-1.03	benign supporting
-0.836	[-0.863;-0.80	benign supporting
-0.900	[-0.918;-0.88	benign supporting
-1.126	[-1.146;-1.11	benign supporting
-1.089	[-1.156;-0.68	benign supporting
-0.515	[-0.542;-0.49	benign supporting
-0.737	[-0.758;-0.71	benign supporting
-0.621	[-0.697;-0.55	benign supporting
-0.914	[-0.971;-0.84	benign supporting
-0.940	[-1.046;-0.80	benign supporting
-1.086	[-1.091;-1.08	benign supporting
-0.441	[-0.456;-0.42	benign supporting
-1.035	[-1.049;-1.02	benign supporting
1.117	[0.794;1.238]	pathogenic moderate
-0.881	[-0.909;-0.85	benign supporting
0.126	[0.009;0.261]	indeterminate
-0.349	[-0.362;-0.33	benign supporting

-0.621	[-0.638;-0.60	benign supporting
-1.087	[-1.168;-1.06	benign supporting
-1.094	[-1.114;-1.07	benign supporting
-0.874	[-0.915;-0.83	benign supporting
-1.087	[-1.092;-1.08	benign supporting
-0.426	[-0.449;-0.40	benign supporting
-1.102	[-1.108;-1.09	benign supporting
-0.937	[-0.996;-0.87	benign supporting
-0.619	[-0.640;-0.59	benign supporting
-0.670	[-0.694;-0.64	benign supporting
-1.096	[-1.102;-1.09	benign supporting
-0.572	[-0.597;-0.54	benign supporting
-0.830	[-0.949;-0.67	benign supporting
-0.833	[-0.866;-0.78	benign supporting
-0.526	[-0.583;-0.46	benign supporting
-0.556	[-0.643;-0.45	benign supporting
-0.663	[-0.966;-0.46	benign supporting
0.046	[-0.078;0.338	indeterminate
-0.678	[-0.851;-0.57	benign supporting
0.604	[0.458;0.725]	pathogenic supporting
-0.149	[-0.205;-0.09	indeterminate
-1.110	[-1.169;-1.08	benign supporting
-1.063	[-1.112;-1.03	benign supporting
-0.329	[-0.425;-0.18	benign supporting
-1.100	[-1.115;-1.04	benign supporting
1.276	[1.145;1.517]	pathogenic strong
0.937	[0.850;1.017]	pathogenic moderate
1.193	[1.146;1.243]	pathogenic moderate
-0.822	[-0.845;-0.78	benign supporting
1.276	[1.213;1.356]	pathogenic strong
-1.084	[-1.168;-0.98	benign supporting
-1.056	[-1.064;-1.04	benign supporting
-1.084	[-1.085;-1.08	benign supporting
-0.896	[-0.986;-0.78	benign supporting
0.798	[0.641;0.945]	pathogenic moderate
-1.113	[-1.116;-1.10	benign supporting
-1.091	[-1.096;-1.08	benign supporting
-0.832	[-1.023;-0.61	benign supporting
1.276	[1.163;1.454]	pathogenic strong
-0.551	[-0.558;-0.54	benign supporting
-1.164	[-1.165;-1.16	benign supporting
-0.153	[-0.280;-0.01	indeterminate
-1.090	[-1.095;-1.08	benign supporting

-0.276	[-0.419;-0.04	indeterminate
1.276	[0.856;5.030	pathogenic strong
-0.405	[-0.449;-0.36	benign supporting
-1.089	[-1.102;-1.08	benign supporting
-1.098	[-1.113;-1.08	benign supporting
-0.602	[-0.726;-0.49	benign supporting
1.276	[1.130;1.576	pathogenic strong
1.276	[1.228;1.334	pathogenic strong
-0.877	[-0.883;-0.87	benign supporting
-1.145	[-1.169;-0.98	benign supporting
-0.806	[-1.164;0.878	benign supporting
-0.468	[-0.775;-0.21	benign supporting
-1.114	[-1.132;-1.10	benign supporting
1.276	[1.083;1.709	pathogenic strong
-0.313	[-1.166;4.956	indeterminate
0.945	[0.174;1.236	pathogenic moderate
1.276	[1.102;1.677	pathogenic strong
-0.766	[-0.928;-0.62	benign supporting
0.527	[0.399;0.635	pathogenic supporting
0.376	[-1.157;4.993	pathogenic supporting
1.276	[0.574;5.090	pathogenic strong
1.276	[1.052;1.810	pathogenic strong
-0.988	[-1.166;-0.89	benign supporting
-0.836	[-1.068;-0.57	benign supporting
-0.855	[-3.805;-0.83	benign supporting
1.257	[0.000;5.102	pathogenic moderate
1.276	[1.224;1.337	pathogenic strong
1.276	[0.666;5.083	pathogenic strong
-0.871	[-1.106;-0.48	benign supporting
-1.090	[-1.157;-0.68	benign supporting
0.763	[0.608;0.908	pathogenic moderate
1.276	[1.188;1.393	pathogenic strong
-1.075	[-1.081;-1.07	benign supporting
-1.049	[-1.061;-1.03	benign supporting
0.441	[0.402;0.481	pathogenic supporting
-0.922	[-0.927;-0.91	benign supporting
1.276	[1.190;1.397	pathogenic strong
-1.070	[-1.114;-0.85	benign supporting
0.192	[-0.034;0.431	indeterminate
-1.085	[-1.090;-1.08	benign supporting
0.765	[0.612;0.907	pathogenic moderate
0.058	[-0.135;0.497	indeterminate
1.276	[0.578;5.101	pathogenic strong

-1.134	[-1.169;-1.08	benign supporting
-1.068	[-1.109;-0.96	benign supporting
-0.561	[-1.087;0.008	benign supporting
-1.090	[-1.168;-0.54	benign supporting
-1.170	[-1.170;-1.13	benign supporting
-1.099	[-1.168;-1.08	benign supporting
-0.967	[-1.073;-0.81	benign supporting
-0.962	[-0.963;-0.96	benign supporting
1.018	[-0.607;5.045	pathogenic moderate
-0.865	[-0.880;-0.85	benign supporting
1.185	[1.169;1.200]	pathogenic moderate
-0.982	[-0.986;-0.97	benign supporting
-0.927	[-0.936;-0.91	benign supporting
-0.129	[-0.196;-0.07	indeterminate
-1.084	[-1.088;-1.08	benign supporting
-1.084	[-1.086;-1.08	benign supporting
1.276	[1.219;1.346]	pathogenic strong
-1.165	[-1.166;-1.16	benign supporting
-0.815	[-1.109;-0.35	benign supporting
1.276	[1.249;1.305]	pathogenic strong
-0.454	[-0.496;-0.41	benign supporting
0.060	[-0.153;0.538	indeterminate
1.276	[1.199;1.379]	pathogenic strong
-1.044	[-1.090;-1.00	benign supporting
-1.169	[-1.169;-1.09	benign supporting
-1.109	[-1.137;-1.09	benign supporting
-1.084	[-1.087;-1.08	benign supporting
-0.576	[-0.588;-0.56	benign supporting
1.244	[1.216;1.272]	pathogenic moderate
-0.280	[-0.301;-0.25	indeterminate
1.124	[0.028;1.610]	pathogenic moderate
-0.937	[-1.090;-0.66	benign supporting
-0.091	[-0.243;0.229	indeterminate
-0.959	[-0.961;-0.95	benign supporting
-1.083	[-1.147;-0.66	benign supporting
-0.595	[-0.646;-0.53	benign supporting
1.276	[0.853;5.068]	pathogenic strong
-1.169	[-1.170;-1.15	benign supporting
1.276	[1.164;1.450]	pathogenic strong
-0.442	[-0.914;0.096	benign supporting
1.268	[0.882;3.053]	pathogenic moderate
-1.038	[-1.043;-1.03	benign supporting
-1.090	[-1.100;-1.08	benign supporting

-1.094	[-1.115;-1.06	benign supporting
-0.437	[-0.656;-0.21	benign supporting
-1.100	[-1.115;-1.08	benign supporting
-0.929	[-0.934;-0.92	benign supporting
0.828	[0.718;0.948]	pathogenic moderate
-0.822	[-1.113;-0.28	benign supporting
1.201	[1.166;1.238]	pathogenic moderate
-0.266	[-0.388;-0.07	indeterminate
-1.120	[-1.127;-1.11	benign supporting
0.801	[0.596;1.000]	pathogenic moderate
-1.075	[-1.100;-1.03	benign supporting
-0.278	[-0.400;-0.08	indeterminate
1.224	[1.156;1.302]	pathogenic moderate
1.276	[1.253;1.301]	pathogenic strong
-0.614	[-1.101;-0.06	benign supporting
1.276	[0.850;5.039]	pathogenic strong
-1.096	[-1.101;-1.09	benign supporting
-0.363	[-0.376;-0.35	benign supporting
-0.675	[-1.081;-0.37	benign supporting
-1.035	[-1.071;-0.98	benign supporting
1.276	[0.585;5.088]	pathogenic strong
1.276	[0.445;5.101]	pathogenic strong
1.276	[1.179;1.415]	pathogenic strong
-1.152	[-1.169;-1.11	benign supporting
-1.086	[-1.092;-1.08	benign supporting
0.306	[-0.253;0.96(	indeterminate
0.305	[-1.143;4.97[illegible]	indeterminate
-0.459	[-1.167;4.871	benign supporting
-1.165	[-1.169;-1.09	benign supporting
-0.261	[-0.404;-0.02	indeterminate
-1.073	[-1.094;-1.03	benign supporting
-1.101	[-1.106;-1.09	benign supporting
-0.696	[-0.891;-0.57	benign supporting
-0.291	[-0.319;-0.26	indeterminate
-1.044	[-1.057;-1.03	benign supporting
1.276	[-1.083;5.07[illegible]	pathogenic strong
-0.464	[-0.506;-0.43	benign supporting
-0.878	[-0.908;-0.85	benign supporting
1.093	[1.009;1.130]	pathogenic moderate
-0.104	[-0.149;-0.06	indeterminate
-1.038	[-1.049;-1.02	benign supporting
1.276	[1.191;1.389]	pathogenic strong
-0.540	[-0.564;-0.51	benign supporting

-0.961	[-1.066;-0.83	benign supporting
1.276	[1.235;1.323]	pathogenic strong
-1.070	[-1.077;-1.06	benign supporting
0.305	[-0.026;0.578	indeterminate
-0.286	[-0.314;-0.25	indeterminate
-1.037	[-1.061;-1.01	benign supporting
-0.944	[-0.954;-0.93	benign supporting
-1.090	[-1.094;-1.08	benign supporting
-0.813	[-0.848;-0.76	benign supporting
-1.117	[-1.125;-1.11	benign supporting
-0.450	[-0.466;-0.43	benign supporting
-1.001	[-1.026;-0.96	benign supporting
-0.632	[-0.666;-0.60	benign supporting
-1.023	[-1.078;-0.93	benign supporting
-1.049	[-1.063;-1.03	benign supporting
-0.115	[-0.137;-0.09	indeterminate
-0.906	[-0.916;-0.89	benign supporting
-0.539	[-0.564;-0.51	benign supporting
-1.094	[-1.099;-1.08	benign supporting
-0.547	[-0.567;-0.52	benign supporting
1.136	[1.099;1.172]	pathogenic moderate
1.276	[1.249;1.307]	pathogenic strong
-0.706	[-0.734;-0.68	benign supporting
-0.115	[-0.149;-0.08	indeterminate
-0.508	[-0.530;-0.48	benign supporting
-0.697	[-0.715;-0.68	benign supporting
-1.038	[-1.113;-0.70	benign supporting
-0.662	[-0.864;-0.54	benign supporting
-0.941	[-1.022;-0.84	benign supporting
-1.089	[-1.115;-1.08	benign supporting
0.517	[-0.019;0.848	pathogenic supporting
0.594	[-1.100;5.030	pathogenic supporting
-1.124	[-1.154;-1.10	benign supporting
1.276	[1.243;1.313]	pathogenic strong
-1.087	[-1.104;-1.07	benign supporting
-0.232	[-0.727;1.095	indeterminate
-0.554	[-0.849;-0.34	benign supporting
0.914	[0.842;0.991]	pathogenic moderate
0.899	[0.788;1.014]	pathogenic moderate
-0.324	[-0.346;-0.29	benign supporting
-0.893	[-0.996;-0.76	benign supporting
1.120	[1.083;1.145]	pathogenic moderate
-0.106	[-0.171;-0.04	indeterminate

-0.526	[-1.094;0.442	benign supporting
-0.933	[-0.984;-0.87	benign supporting
-0.116	[-0.314;0.347	indeterminate
-0.479	[-0.569;-0.40	benign supporting
-0.919	[-1.095;-0.62	benign supporting
1.276	[-1.102;5.044	pathogenic strong
0.936	[-0.881;5.043	pathogenic moderate
1.276	[-1.112;5.028	pathogenic strong
-0.306	[-0.383;-0.18	indeterminate
0.384	[-1.109;5.022	pathogenic supporting
1.130	[0.860;1.249]	pathogenic moderate
1.276	[-1.100;5.067	pathogenic strong
1.276	[0.000;5.103]	pathogenic strong
-0.608	[-1.166;1.677	benign supporting
1.276	[-0.337;5.101	pathogenic strong
1.276	[-0.192;5.103	pathogenic strong
1.276	[-1.119;5.003	pathogenic strong
1.276	[-1.115;5.028	pathogenic strong
0.312	[-0.419;1.182	indeterminate
-0.419	[-0.903;0.340	benign supporting
1.276	[0.548;5.100]	pathogenic strong
1.276	[0.521;5.095]	pathogenic strong
-0.488	[-0.590;-0.39	benign supporting
-0.343	[-0.666;0.472	benign supporting
-0.692	[-0.775;-0.63	benign supporting
-0.949	[-0.973;-0.92	benign supporting
1.276	[1.232;1.326]	pathogenic strong
-1.100	[-1.114;-1.08	benign supporting
-1.103	[-1.115;-1.09	benign supporting
-0.303	[-0.345;-0.24	indeterminate
-1.099	[-1.115;-1.08	benign supporting
-1.034	[-1.058;-1.01	benign supporting
-0.623	[-0.645;-0.60	benign supporting
0.097	[-0.848;1.497	indeterminate
0.427	[0.186;0.614]	pathogenic supporting
-0.133	[-0.192;-0.08	indeterminate
-0.436	[-0.473;-0.40	benign supporting
-1.093	[-1.110;-1.08	benign supporting
1.276	[1.205;1.368]	pathogenic strong
-1.165	[-1.169;-1.13	benign supporting
0.342	[0.199;0.461]	pathogenic supporting
-0.889	[-0.921;-0.85	benign supporting
-0.790	[-0.899;-0.67	benign supporting

-0.901	[-3.712;0.00(	benign supporting
-1.084	[-3.364;1.53(	benign supporting
-1.104	[-1.115;-1.09	benign supporting
-0.976	[-1.032;-0.94	benign supporting
-0.645	[-1.166;1.42(	benign supporting
-1.037	[-1.096;-0.91	benign supporting
-1.159	[-1.169;-1.07	benign supporting
-0.984	[-1.067;-0.93	benign supporting
-1.068	[-1.168;-0.91	benign supporting
-0.929	[-0.941;-0.91	benign supporting
1.266	[-0.769;5.087	pathogenic moderate
-0.430	[-0.500;-0.37	benign supporting
-0.870	[-0.882;-0.86	benign supporting
-0.921	[-0.930;-0.91	benign supporting
1.217	[1.131;1.316]	pathogenic moderate
-1.115	[-1.167;-0.58	benign supporting
1.276	[0.913;4.953]	pathogenic strong
1.276	[0.000;5.104]	pathogenic strong
1.276	[0.920;4.843]	pathogenic strong
1.276	[0.467;5.099]	pathogenic strong
1.276	[0.919;4.754]	pathogenic strong
-0.714	[-0.943;-0.55	benign supporting
-0.945	[-0.973;-0.91	benign supporting
-0.837	[-0.846;-0.83	benign supporting
-1.044	[-1.077;-1.01	benign supporting
-0.878	[-1.069;-0.63	benign supporting
0.730	[0.145;1.101]	pathogenic moderate
-0.887	[-0.950;-0.82	benign supporting
-0.869	[-0.941;-0.85	benign supporting
1.276	[1.074;1.772]	pathogenic strong
-1.054	[-1.105;-0.93	benign supporting
-1.091	[-1.135;-1.05	benign supporting
1.276	[0.789;5.066]	pathogenic strong
1.276	[1.109;1.666]	pathogenic strong
-1.154	[-1.169;-0.92	benign supporting
0.360	[0.162;0.514]	pathogenic supporting
1.276	[0.947;2.464]	pathogenic strong
-0.477	[-0.927;-0.07	benign supporting
-1.091	[-1.097;-1.08	benign supporting
1.274	[1.223;1.334]	pathogenic strong
-0.409	[-0.448;-0.37	benign supporting
-1.169	[-1.170;-1.15	benign supporting
-1.006	[-1.017;-0.99	benign supporting

-1.163	[-1.170;-1.14	benign supporting
-1.164	[-1.169;-1.06	benign supporting
-1.085	[-1.090;-1.08	benign supporting
1.276	[1.256;1.298]	pathogenic strong
-1.090	[-1.112;-1.08	benign supporting
-1.085	[-1.092;-1.08	benign supporting
-0.426	[-0.468;-0.39	benign supporting
-0.997	[-1.003;-0.99	benign supporting
-0.817	[-0.837;-0.78	benign supporting
-1.075	[-1.109;-1.01	benign supporting
1.224	[1.072;1.411]	pathogenic moderate
-0.766	[-0.962;-0.60	benign supporting
-1.155	[-1.169;-1.14	benign supporting
-0.276	[-0.312;-0.23	indeterminate
-1.103	[-1.115;-1.08	benign supporting
-1.112	[-1.116;-1.10	benign supporting
-0.400	[-0.483;-0.32	benign supporting
1.276	[1.235;1.324]	pathogenic strong
1.276	[1.252;1.303]	pathogenic strong
1.276	[1.261;1.292]	pathogenic strong
1.128	[-0.233;4.96(	pathogenic moderate
1.276	[1.134;1.536]	pathogenic strong
1.276	[1.269;1.283]	pathogenic strong
-1.058	[-1.070;-1.03	benign supporting
1.276	[1.116;1.602]	pathogenic strong
1.276	[1.264;1.289]	pathogenic strong
1.276	[1.178;1.417]	pathogenic strong
1.276	[1.268;1.285]	pathogenic strong
1.276	[1.255;1.299]	pathogenic strong
1.276	[1.261;1.292]	pathogenic strong
1.276	[1.153;1.468]	pathogenic strong
1.276	[1.238;1.321]	pathogenic strong
-1.094	[-1.167;-0.85	benign supporting
1.276	[1.258;1.296]	pathogenic strong
1.276	[1.260;1.293]	pathogenic strong
1.276	[1.132;1.539]	pathogenic strong
1.276	[0.875;5.000]	pathogenic strong
1.276	[1.258;1.296]	pathogenic strong
-0.426	[-0.597;-0.27	benign supporting
-0.633	[-1.070;-0.33	benign supporting
-0.297	[-0.317;-0.27	indeterminate
0.927	[0.718;1.107]	pathogenic moderate
1.276	[0.963;2.481]	pathogenic strong

0.000	[0.000;0.000]	indeterminate
-1.080	[-1.097;-1.06	benign supporting
1.276	[1.179;1.422]	pathogenic strong
-1.045	[-1.167;-0.95	benign supporting
-0.570	[-0.947;-0.30	benign supporting
-0.373	[-0.389;-0.35	benign supporting
-1.053	[-1.063;-1.04	benign supporting
-1.096	[-1.165;-0.99	benign supporting
-0.013	[-0.697;1.305	indeterminate
1.276	[1.252;1.302]	pathogenic strong
1.276	[1.077;1.729]	pathogenic strong
-0.945	[-0.950;-0.94	benign supporting
-1.110	[-1.115;-1.10	benign supporting
-0.472	[-0.483;-0.46	benign supporting
-1.166	[-1.169;-1.15	benign supporting
-1.087	[-1.168;-1.06	benign supporting
-1.119	[-1.142;-1.09	benign supporting
-1.006	[-1.164;-0.33	benign supporting
1.276	[-0.149;5.103	pathogenic strong
-0.042	[-0.101;0.063	indeterminate
-1.066	[-1.114;-0.69	benign supporting
-0.931	[-1.013;-0.83	benign supporting
-1.088	[-1.101;-1.07	benign supporting
-0.044	[-0.399;0.916	indeterminate
0.305	[-0.053;0.630	indeterminate
1.276	[0.908;5.025]	pathogenic strong
1.268	[1.227;1.314]	pathogenic moderate
0.515	[-0.636;1.683	pathogenic supporting
-1.167	[-1.169;-1.02	benign supporting
1.276	[1.092;1.711]	pathogenic strong
-0.944	[-0.951;-0.93	benign supporting
-0.180	[-0.363;0.234	indeterminate
1.276	[1.020;1.962]	pathogenic strong
-1.017	[-1.063;-0.97	benign supporting
-1.169	[-1.168;-0.91	benign supporting
-3.197	[-3.682;0.000	benign strong
-0.657	[-0.875;-0.51	benign supporting
-0.289	[-0.476;0.146	indeterminate
-1.087	[-1.088;-1.08	benign supporting
-1.166	[-1.169;-1.10	benign supporting
-0.976	[-0.991;-0.96	benign supporting
-1.167	[-1.169;-1.16	benign supporting
1.276	[0.223;5.098]	pathogenic strong

-0.024	[-0.070;0.04	indeterminate
-1.128	[-1.168;-0.90	benign supporting
1.276	[0.440;5.099	pathogenic strong
-1.099	[-1.166;-0.91	benign supporting
1.276	[0.615;5.094	pathogenic strong
-0.482	[-0.724;-0.27	benign supporting
-0.319	[-0.340;-0.29	benign supporting
-1.098	[-1.114;-1.08	benign supporting
-1.104	[-1.168;-1.08	benign supporting
-1.000	[-1.071;-0.88	benign supporting
-1.132	[-1.155;-1.11	benign supporting
0.390	[0.182;0.558	pathogenic supporting
-1.066	[-1.071;-1.05	benign supporting
-0.926	[-0.992;-0.85	benign supporting
-0.574	[-0.603;-0.54	benign supporting
-1.101	[-1.114;-1.08	benign supporting
-1.114	[-1.116;-1.10	benign supporting
-1.070	[-1.103;-1.00	benign supporting
-1.104	[-1.108;-1.09	benign supporting
-1.168	[-1.170;-1.15	benign supporting
-1.034	[-1.045;-1.02	benign supporting
-0.217	[-0.265;-0.16	indeterminate
1.276	[1.237;1.322	pathogenic strong
-1.168	[-1.170;-1.16	benign supporting
-1.087	[-1.090;-1.08	benign supporting
0.139	[-0.044;0.37	indeterminate
-0.568	[-0.587;-0.54	benign supporting
-1.089	[-1.168;-1.08	benign supporting
-1.108	[-1.115;-1.10	benign supporting
-0.991	[-1.091;-0.79	benign supporting
-1.075	[-1.081;-1.06	benign supporting
-1.085	[-1.098;-1.07	benign supporting
-1.154	[-1.170;-1.13	benign supporting
-0.892	[-0.906;-0.87	benign supporting
-0.457	[-0.469;-0.44	benign supporting
-0.563	[-0.587;-0.53	benign supporting
-0.956	[-0.962;-0.94	benign supporting
-0.518	[-0.573;-0.46	benign supporting
-1.036	[-1.050;-1.02	benign supporting
-0.288	[-0.309;-0.26	indeterminate
-0.284	[-0.303;-0.26	indeterminate
-0.656	[-0.875;-0.52	benign supporting
0.240	[0.130;0.330	indeterminate

-1.084	[-1.085;-1.08	benign supporting
-0.941	[-0.959;-0.92	benign supporting
-1.096	[-1.102;-1.09	benign supporting
-0.715	[-0.865;-0.61	benign supporting
-1.105	[-1.128;-1.09	benign supporting
-0.124	[-0.365;0.51	indeterminate
-1.005	[-1.086;-0.85	benign supporting
0.250	[-1.160;5.00	indeterminate
-1.136	[-1.169;-1.05	benign supporting
-0.841	[-0.878;-0.79	benign supporting
-0.077	[-0.200;0.17	indeterminate
-0.643	[-0.694;-0.60	benign supporting
1.050	[0.731;1.188	pathogenic moderate
1.276	[-0.799;5.08	pathogenic strong
-0.730	[-1.112;-0.16	benign supporting
1.276	[1.158;1.465	pathogenic strong
1.276	[0.000;5.105	pathogenic strong
-1.088	[-1.168;-0.64	benign supporting
-0.137	[-1.088;1.68	indeterminate
1.147	[0.942;1.253	pathogenic moderate
-0.445	[-1.157;1.79	benign supporting
-0.807	[-0.860;-0.73	benign supporting
-0.911	[-1.074;-0.65	benign supporting
-1.084	[-1.089;-1.08	benign supporting
-0.967	[-1.051;-0.87	benign supporting
-0.577	[-0.609;-0.54	benign supporting
-1.038	[-1.052;-1.02	benign supporting
-1.093	[-1.102;-1.08	benign supporting
-0.626	[-0.649;-0.60	benign supporting
-1.090	[-1.093;-1.08	benign supporting
-1.084	[-1.110;-1.06	benign supporting
-1.114	[-1.116;-1.09	benign supporting
-0.856	[-1.026;-0.64	benign supporting
-1.104	[-1.115;-1.09	benign supporting
-1.021	[-1.065;-0.97	benign supporting
-0.621	[-0.629;-0.61	benign supporting
-1.168	[-1.170;-1.15	benign supporting
-1.170	[-1.170;-1.15	benign supporting
-0.611	[-0.644;-0.57	benign supporting
-1.087	[-1.099;-1.07	benign supporting
1.268	[1.230;1.310	pathogenic moderate
-0.746	[-0.843;-0.66	benign supporting
-0.703	[-0.732;-0.68	benign supporting

-0.856	[-0.877;-0.83	benign supporting
-0.857	[-0.876;-0.83	benign supporting
-0.597	[-0.634;-0.55	benign supporting
-1.086	[-1.092;-1.08	benign supporting
-1.079	[-1.114;-0.94	benign supporting
-1.089	[-1.169;-0.97	benign supporting
-0.262	[-0.375;-0.09	indeterminate
-1.076	[-1.168;-0.99	benign supporting
-0.644	[-0.700;-0.60	benign supporting
0.538	[-0.059;0.937	pathogenic supporting
-1.083	[-1.114;-0.99	benign supporting
-0.517	[-0.593;-0.44	benign supporting
-0.621	[-0.732;-0.53	benign supporting
-0.956	[-1.111;-0.55	benign supporting
-0.838	[-0.840;-0.83	benign supporting
-0.282	[-0.330;-0.22	indeterminate
1.276	[-0.817;5.077	pathogenic strong
-1.095	[-1.115;-1.01	benign supporting
1.276	[-1.096;5.055	pathogenic strong
-1.155	[-1.169;-1.07	benign supporting
1.276	[-0.451;5.095	pathogenic strong
-0.171	[-0.514;0.819	indeterminate
-1.108	[-1.115;-1.08	benign supporting
-1.111	[-1.115;-1.07	benign supporting
1.228	[1.054;1.453]	pathogenic moderate
1.276	[0.999;2.086]	pathogenic strong
1.268	[0.709;5.078]	pathogenic moderate
-0.376	[-0.455;-0.28	benign supporting
1.276	[1.114;1.613]	pathogenic strong
-0.843	[-1.104;-0.44	benign supporting
0.798	[0.350;1.105]	pathogenic moderate
1.276	[1.162;1.455]	pathogenic strong
1.276	[1.049;1.907]	pathogenic strong
1.276	[-0.204;5.107	pathogenic strong
0.345	[-0.197;0.890	pathogenic supporting
1.276	[-1.109;5.046	pathogenic strong
1.148	[0.746;1.359]	pathogenic moderate
1.276	[1.187;1.397]	pathogenic strong
1.276	[1.225;1.338]	pathogenic strong
1.276	[-1.096;5.059	pathogenic strong
1.108	[0.751;1.236]	pathogenic moderate
1.276	[-0.040;5.102	pathogenic strong
-0.965	[-1.070;-0.82	benign supporting

-1.074	[-1.093;-1.04	benign supporting
1.036	[-0.256;1.827	pathogenic moderate
-0.982	[-1.008;-0.95	benign supporting
-0.044	[-0.407;0.943	indeterminate
1.102	[-1.065;5.070	pathogenic moderate
-0.384	[-1.166;4.855	benign supporting
-0.636	[-0.673;-0.60	benign supporting
-1.143	[-1.169;-1.11	benign supporting
-1.104	[-1.115;-1.09	benign supporting
-0.240	[-1.167;4.921	indeterminate
1.276	[-0.632;5.093	pathogenic strong
-0.474	[-0.556;-0.40	benign supporting
1.177	[0.319;2.235]	pathogenic moderate
-0.908	[-1.065;-0.68	benign supporting
-1.086	[-1.099;-1.08	benign supporting
1.276	[-1.130;5.019	pathogenic strong
1.276	[-1.129;4.980	pathogenic strong
-1.129	[-1.169;-1.07	benign supporting
-0.008	[-0.278;0.668	indeterminate
-1.121	[-1.130;-1.11	benign supporting
-0.272	[-0.939;1.160	indeterminate
1.276	[-0.216;5.107	pathogenic strong
-0.098	[-0.468;0.941	indeterminate
1.276	[-1.123;4.983	pathogenic strong
-1.113	[-1.115;-1.06	benign supporting
-0.406	[-0.632;-0.13	benign supporting
1.250	[-0.549;5.091	pathogenic moderate
1.276	[-0.229;5.108	pathogenic strong
-0.683	[-0.801;-0.61	benign supporting
-0.359	[-0.548;-0.09	benign supporting
0.434	[-0.728;1.851	pathogenic supporting
-1.090	[-1.099;-1.08	benign supporting
-0.609	[-0.691;-0.52	benign supporting
0.814	[0.674;0.964]	pathogenic moderate
1.276	[0.340;5.098]	pathogenic strong
-0.783	[-0.957;-0.61	benign supporting
1.276	[-0.030;5.101	pathogenic strong
-0.320	[-0.394;-0.21	benign supporting
0.950	[0.683;1.131]	pathogenic moderate
-1.103	[-1.169;-0.83	benign supporting
-1.101	[-1.111;-1.09	benign supporting
-0.441	[-0.643;-0.25	benign supporting
-1.085	[-1.102;-1.08	benign supporting

-1.087	[-1.121;-1.08	benign supporting
-1.091	[-1.112;-1.08	benign supporting
-1.088	[-1.114;-1.05	benign supporting
-0.208	[-0.265;-0.14	indeterminate
-1.085	[-1.104;-1.06	benign supporting
-0.249	[-0.669;0.952	indeterminate
-0.336	[-0.459;-0.14	benign supporting
-0.715	[-0.814;-0.64	benign supporting
-0.633	[-0.869;-0.47	benign supporting
-0.656	[-0.720;-0.61	benign supporting
-1.098	[-1.105;-1.09	benign supporting
0.309	[-0.059;0.634	indeterminate
-0.038	[-0.428;1.000	indeterminate
-0.825	[-0.907;-0.71	benign supporting
1.276	[1.235;1.325]	pathogenic strong
-0.708	[-0.769;-0.66	benign supporting
-0.585	[-0.670;-0.49	benign supporting
-0.583	[-0.636;-0.52	benign supporting
-1.000	[-1.019;-0.97	benign supporting
-0.498	[-0.873;-0.18	benign supporting
-1.021	[-1.044;-1.00	benign supporting
-1.081	[-1.098;-1.06	benign supporting
-1.093	[-1.115;-1.05	benign supporting
-0.279	[-0.332;-0.21	indeterminate
-0.877	[-0.902;-0.85	benign supporting
-1.067	[-1.070;-1.06	benign supporting
-0.803	[-0.832;-0.77	benign supporting
-1.113	[-1.116;-1.09	benign supporting
-1.104	[-1.115;-1.09	benign supporting
-0.700	[-0.736;-0.67	benign supporting
-1.101	[-1.114;-1.09	benign supporting
-1.087	[-1.090;-1.08	benign supporting
-0.536	[-0.609;-0.45	benign supporting
0.615	[-0.065;1.055	pathogenic supporting
-1.090	[-1.166;-1.08	benign supporting
0.725	[0.641;0.797]	pathogenic moderate
-1.090	[-1.093;-1.08	benign supporting
-1.015	[-1.065;-0.95	benign supporting
-0.889	[-0.975;-0.77	benign supporting
-0.616	[-0.670;-0.56	benign supporting
-1.166	[-1.169;-0.95	benign supporting
0.409	[-0.046;0.759	pathogenic supporting
0.764	[-0.044;1.167	pathogenic moderate

1.276	[1.185;1.402	pathogenic strong
0.558	[-0.018;0.91	pathogenic supporting
0.995	[0.876;1.081	pathogenic moderate
1.276	[1.216;1.351	pathogenic strong
-0.307	[-0.437;-0.09	indeterminate
1.104	[0.604;1.272	pathogenic moderate
0.812	[0.520;1.078	pathogenic moderate
-0.461	[-0.524;-0.41	benign supporting
1.276	[1.248;1.307	pathogenic strong
-0.414	[-0.436;-0.39	benign supporting
-0.875	[-0.955;-0.77	benign supporting
1.276	[1.032;1.896	pathogenic strong
-1.006	[-1.067;-0.92	benign supporting
1.276	[1.234;1.326	pathogenic strong
-0.152	[-0.196;-0.10	indeterminate
1.276	[0.978;2.240	pathogenic strong
-0.879	[-1.114;-0.34	benign supporting
-1.095	[-1.098;-1.09	benign supporting
-1.102	[-1.115;-1.08	benign supporting
-0.822	[-0.839;-0.79	benign supporting
-1.101	[-1.112;-1.09	benign supporting
1.276	[1.192;1.389	pathogenic strong
-0.553	[-0.575;-0.52	benign supporting
1.276	[1.237;1.321	pathogenic strong
1.276	[1.247;1.308	pathogenic strong
-0.763	[-0.830;-0.70	benign supporting
-0.284	[-0.327;-0.23	indeterminate
-0.474	[-0.492;-0.45	benign supporting
-0.959	[-0.998;-0.92	benign supporting
-0.616	[-0.657;-0.57	benign supporting
0.242	[0.058;0.383	indeterminate
-0.791	[-0.854;-0.71	benign supporting
0.995	[0.834;1.106	pathogenic moderate
-0.797	[-0.850;-0.73	benign supporting
1.276	[1.235;1.323	pathogenic strong
0.860	[0.812;0.909	pathogenic moderate
-0.771	[-0.838;-0.70	benign supporting
-0.229	[-0.419;0.29	indeterminate
-1.093	[-1.120;-1.08	benign supporting
-1.092	[-1.114;-1.08	benign supporting
-0.412	[-0.628;-0.16	benign supporting
-0.987	[-0.991;-0.98	benign supporting
-0.626	[-0.645;-0.60	benign supporting

-1.134	[-1.169;-1.10	benign supporting
-0.847	[-3.797;0.00(	benign supporting
1.162	[0.950;1.294]	pathogenic moderate
-1.021	[-1.045;-0.99	benign supporting
-1.133	[-1.144;-1.12	benign supporting
-1.076	[-1.113;-1.05	benign supporting
-1.101	[-1.115;-1.08	benign supporting
-0.945	[-0.966;-0.92	benign supporting
1.229	[1.195;1.265]	pathogenic moderate
1.276	[0.000;5.103]	pathogenic strong
0.974	[0.563;1.173]	pathogenic moderate
1.054	[-0.321;2.873	pathogenic moderate
1.276	[0.818;5.064]	pathogenic strong
-1.095	[-1.124;-1.08	benign supporting
-0.351	[-0.363;-0.34	benign supporting
-1.050	[-1.063;-1.03	benign supporting
-1.091	[-1.168;-0.99	benign supporting
1.276	[-0.002;5.10(	pathogenic strong
0.016	[-0.257;0.683	indeterminate
-0.861	[-0.888;-0.83	benign supporting
1.276	[1.130;1.565]	pathogenic strong
-1.004	[-1.014;-0.99	benign supporting
0.343	[0.068;0.536]	pathogenic supporting
-1.062	[-1.067;-1.05	benign supporting
-0.962	[-0.978;-0.94	benign supporting
-0.510	[-0.804;-0.29	benign supporting
1.276	[1.150;1.490]	pathogenic strong
-0.270	[-0.646;0.819	indeterminate
1.276	[0.726;5.092]	pathogenic strong
1.276	[0.818;5.065]	pathogenic strong
0.149	[-0.109;0.549	indeterminate
-0.996	[-1.111;-0.62	benign supporting
1.276	[0.000;5.105]	pathogenic strong
1.276	[1.180;1.416]	pathogenic strong
1.276	[1.140;1.511]	pathogenic strong
-0.989	[-1.000;-0.97	benign supporting
-0.985	[-1.005;-0.96	benign supporting
-0.597	[-0.660;-0.52	benign supporting
-0.082	[-0.106;-0.05	indeterminate
1.276	[1.266;1.287]	pathogenic strong
-0.272	[-0.275;-0.26	indeterminate
1.231	[1.224;1.238]	pathogenic moderate
-0.713	[-0.716;-0.71	benign supporting

0.391	[0.334;0.445	pathogenic supporting
-0.954	[-0.958;-0.95	benign supporting
0.122	[-0.155;0.602	indeterminate
1.276	[1.148;1.486	pathogenic strong
0.289	[0.271;0.306	indeterminate
-0.419	[-0.420;-0.41	benign supporting
0.731	[0.723;0.738	pathogenic moderate
1.276	[1.270;1.283	pathogenic strong
-1.025	[-1.029;-1.02	benign supporting
1.276	[1.118;1.598	pathogenic strong
1.276	[1.261;1.293	pathogenic strong
1.276	[1.272;1.281	pathogenic strong
-0.073	[-0.077;-0.06	indeterminate
-1.127	[-1.169;-1.08	benign supporting
-0.668	[-0.686;-0.65	benign supporting
1.276	[1.038;1.883	pathogenic strong
-0.250	[-0.277;-0.21	indeterminate
-1.068	[-1.085;-1.05	benign supporting
1.276	[1.169;1.442	pathogenic strong
1.276	[1.200;1.376	pathogenic strong
1.276	[1.232;1.327	pathogenic strong
1.276	[0.000;5.106	pathogenic strong
1.244	[1.188;1.307	pathogenic moderate
1.276	[-0.136;5.10	pathogenic strong
0.624	[0.566;0.681	pathogenic supporting
1.276	[1.233;1.325	pathogenic strong
1.276	[0.406;5.096	pathogenic strong
1.149	[1.091;1.205	pathogenic moderate
1.228	[1.145;1.330	pathogenic moderate
1.276	[1.220;1.341	pathogenic strong
1.276	[1.205;1.370	pathogenic strong
-1.102	[-1.167;-1.08	benign supporting
-1.104	[-1.115;-1.08	benign supporting
-0.098	[-0.127;-0.06	indeterminate
-1.087	[-1.091;-1.08	benign supporting
0.833	[0.794;0.876	pathogenic moderate
1.276	[1.259;1.294	pathogenic strong
-0.616	[-0.628;-0.60	benign supporting
-0.447	[-0.472;-0.42	benign supporting
-0.776	[-0.802;-0.75	benign supporting
0.150	[0.055;0.239	indeterminate
-1.095	[-1.100;-1.09	benign supporting
-0.119	[-0.153;-0.08	indeterminate

0.914	[0.855;0.978]	pathogenic moderate
0.584	[0.542;0.625]	pathogenic supporting
-0.722	[-0.746;-0.70	benign supporting
1.167	[1.122;1.217]	pathogenic moderate
-0.918	[-0.954;-0.87	benign supporting
1.276	[1.259;1.295]	pathogenic strong
0.726	[0.606;0.829]	pathogenic moderate
-1.071	[-1.075;-1.06	benign supporting
-0.470	[-0.499;-0.44	benign supporting
-0.520	[-0.757;-0.33	benign supporting
-0.394	[-0.915;0.60	benign supporting
-0.832	[-0.854;-0.80	benign supporting
-1.164	[-1.170;-1.11	benign supporting
1.276	[0.818;5.063]	pathogenic strong
1.276	[0.713;5.084]	pathogenic strong
1.276	[0.815;5.043]	pathogenic strong
1.276	[1.059;1.771]	pathogenic strong
-1.168	[-1.170;-1.15	benign supporting
-0.837	[-0.840;-0.83	benign supporting
-1.087	[-1.089;-1.08	benign supporting
-0.631	[-0.936;-0.42	benign supporting
1.260	[1.252;1.269]	pathogenic moderate
-0.731	[-0.842;-0.64	benign supporting
-0.912	[-0.932;-0.89	benign supporting
-1.084	[-1.120;-1.08	benign supporting
1.276	[1.126;1.568]	pathogenic strong
1.276	[-0.117;5.10	pathogenic strong
-1.094	[-1.167;-0.28	benign supporting
-1.091	[-1.168;-1.08	benign supporting
-0.592	[-1.096;-0.01	benign supporting
-1.087	[-1.159;-1.08	benign supporting
-0.451	[-0.577;-0.35	benign supporting
-0.480	[-0.509;-0.45	benign supporting
-0.501	[-0.563;-0.44	benign supporting
-0.918	[-0.962;-0.86	benign supporting
1.209	[1.054;1.377]	pathogenic moderate
-0.583	[-0.604;-0.56	benign supporting
0.890	[-0.261;1.42	pathogenic moderate
-0.339	[-0.627;0.34	benign supporting
-0.658	[-0.705;-0.62	benign supporting
-0.736	[-0.761;-0.71	benign supporting
-0.300	[-0.458;-0.04	indeterminate
0.342	[-0.366;1.14	pathogenic supporting

-1.084	[-1.165;-1.08	benign supporting
1.276	[0.871;5.013	pathogenic strong
-1.109	[-1.115;-1.10	benign supporting
-0.823	[-0.886;-0.73	benign supporting
-1.098	[-1.115;-1.08	benign supporting
-0.723	[-0.947;-0.56	benign supporting
-0.572	[-0.665;-0.47	benign supporting
1.276	[1.246;1.310	pathogenic strong
0.016	[-0.029;0.11	indeterminate
-0.390	[-0.403;-0.37	benign supporting
-1.072	[-1.083;-1.05	benign supporting
1.276	[0.847;5.025	pathogenic strong
0.970	[0.836;1.076	pathogenic moderate
-0.361	[-0.593;-0.02	benign supporting
-0.981	[-0.987;-0.97	benign supporting
-0.689	[-0.707;-0.67	benign supporting
1.276	[1.202;1.374	pathogenic strong
0.707	[0.582;0.806	pathogenic moderate
1.276	[1.222;1.342	pathogenic strong
-1.013	[-1.024;-1.00	benign supporting
0.697	[0.379;0.925	pathogenic moderate
-1.008	[-1.109;-0.71	benign supporting
1.276	[-0.113;5.10	pathogenic strong
1.276	[1.230;1.328	pathogenic strong
-1.168	[-1.169;-1.15	benign supporting
1.276	[1.219;1.346	pathogenic strong
-1.109	[-1.112;-1.10	benign supporting
-0.994	[-0.994;-0.99	benign supporting
1.276	[1.272;1.280	pathogenic strong
0.860	[0.850;0.871	pathogenic moderate
1.276	[1.269;1.284	pathogenic strong
-0.508	[-0.509;-0.50	benign supporting
1.276	[1.274;1.278	pathogenic strong
1.276	[1.271;1.282	pathogenic strong
-0.944	[-0.948;-0.94	benign supporting
1.276	[1.263;1.290	pathogenic strong
1.276	[1.272;1.280	pathogenic strong
-0.255	[-0.287;-0.21	indeterminate
1.276	[1.219;1.346	pathogenic strong
-0.863	[-0.865;-0.86	benign supporting
1.276	[1.269;1.283	pathogenic strong
1.276	[1.267;1.286	pathogenic strong
-0.732	[-0.831;-0.65	benign supporting

1.276	[1.272;1.281	pathogenic strong
0.730	[0.722;0.738	pathogenic moderate
1.276	[1.269;1.283	pathogenic strong
-0.758	[-0.769;-0.74	benign supporting
-1.041	[-1.085;-0.95	benign supporting
-0.952	[-1.020;-0.87	benign supporting
-1.057	[-1.086;-0.99	benign supporting
-0.700	[-0.721;-0.68	benign supporting
-0.814	[-0.840;-0.77	benign supporting
-0.898	[-0.927;-0.86	benign supporting
-0.457	[-0.577;-0.36	benign supporting
-1.030	[-1.075;-0.96	benign supporting
-0.526	[-0.572;-0.47	benign supporting
-1.081	[-1.090;-1.07	benign supporting
-1.104	[-1.115;-0.97	benign supporting
-0.688	[-0.724;-0.65	benign supporting
-0.690	[-0.772;-0.63	benign supporting
-1.165	[-1.170;-1.15	benign supporting
-0.564	[-0.577;-0.55	benign supporting
-0.204	[-0.294;-0.09	indeterminate
-1.107	[-1.113;-1.10	benign supporting
0.449	[-0.424;1.249	pathogenic supporting
-0.581	[-0.604;-0.56	benign supporting
-0.936	[-0.954;-0.91	benign supporting
-0.024	[-0.201;0.445	indeterminate
-1.087	[-1.117;-1.08	benign supporting
-0.621	[-1.108;0.006	benign supporting
1.276	[1.222;1.341	pathogenic strong
-1.063	[-1.090;-0.99	benign supporting
1.113	[-0.527;5.064	pathogenic moderate
-1.112	[-1.115;-1.08	benign supporting
1.076	[0.705;1.217	pathogenic moderate
-0.907	[-0.953;-0.85	benign supporting
1.276	[-0.587;5.095	pathogenic strong
1.276	[0.000;5.104	pathogenic strong
0.135	[-0.442;1.148	indeterminate
0.661	[0.224;0.957	pathogenic moderate
1.276	[1.139;1.525	pathogenic strong
-1.057	[-1.076;-1.01	benign supporting
1.276	[0.462;5.103	pathogenic strong
1.202	[0.929;1.448	pathogenic moderate
-0.913	[-1.108;-0.51	benign supporting
1.276	[0.678;5.083	pathogenic strong

1.276	[-0.431;5.091	pathogenic strong
1.276	[0.901;4.861	pathogenic strong
-0.540	[-0.615;-0.45	benign supporting
-1.099	[-1.169;-1.07	benign supporting
-0.924	[-1.106;-0.57	benign supporting
-0.500	[-0.631;-0.38	benign supporting
-0.113	[-0.229;0.017	indeterminate
-1.107	[-1.167;-1.08	benign supporting
-1.096	[-1.115;-1.08	benign supporting
-0.399	[-0.594;-0.17	benign supporting
-0.921	[-0.986;-0.84	benign supporting
-0.113	[-0.588;1.126	indeterminate
-0.310	[-1.148;4.893	indeterminate
-0.466	[-0.937;-0.00	benign supporting
-1.111	[-1.115;-1.09	benign supporting
-1.000	[-1.044;-0.96	benign supporting
-1.077	[-1.165;-0.45	benign supporting
-1.119	[-1.167;-1.09	benign supporting
0.372	[-1.164;4.981	pathogenic supporting
1.130	[0.962;1.214	pathogenic moderate
-0.446	[-0.637;-0.27	benign supporting
-1.106	[-1.167;-0.50	benign supporting
-1.097	[-1.135;-1.08	benign supporting
-0.755	[-0.794;-0.71	benign supporting
-0.878	[-0.983;-0.74	benign supporting
0.624	[0.367;0.814	pathogenic supporting
-0.268	[-0.350;-0.14	indeterminate
-1.107	[-1.133;-1.09	benign supporting
-1.069	[-1.095;-1.01	benign supporting
-0.565	[-0.601;-0.52	benign supporting
-1.141	[-1.166;-1.12	benign supporting
-0.751	[-0.878;-0.64	benign supporting
1.276	[1.193;1.389	pathogenic strong
-0.945	[-0.974;-0.91	benign supporting
-0.679	[-0.716;-0.64	benign supporting
-1.102	[-1.115;-0.99	benign supporting
-1.084	[-1.092;-1.08	benign supporting
0.798	[0.693;0.909	pathogenic moderate
-0.612	[-0.650;-0.57	benign supporting
-0.106	[-0.176;-0.03	indeterminate
-1.167	[-1.169;-1.14	benign supporting
-1.105	[-1.115;-1.09	benign supporting
-0.341	[-0.352;-0.32	benign supporting

1.276	[1.241;1.316	pathogenic strong
-0.819	[-0.958;-0.65	benign supporting
-0.060	[-0.136;0.08	indeterminate
-1.101	[-1.134;-1.08	benign supporting
-0.764	[-0.867;-0.66	benign supporting
1.276	[1.203;1.371	pathogenic strong
-1.058	[-1.068;-1.04	benign supporting
0.612	[-0.053;1.04	pathogenic supporting
-1.164	[-1.167;-1.16	benign supporting
-0.813	[-1.098;-0.44	benign supporting
-1.102	[-1.115;-1.08	benign supporting
1.078	[0.898;1.153	pathogenic moderate
1.276	[0.942;4.931	pathogenic strong
1.276	[1.180;1.415	pathogenic strong
-0.527	[-0.547;-0.50	benign supporting
1.276	[1.208;1.363	pathogenic strong
1.121	[-0.117;2.12	pathogenic moderate
-0.125	[-0.252;0.01	indeterminate
-0.858	[-0.907;-0.80	benign supporting
-1.089	[-1.091;-1.08	benign supporting
-0.595	[-0.622;-0.56	benign supporting
-1.109	[-1.111;-1.10	benign supporting
-0.611	[-0.617;-0.60	benign supporting
-1.088	[-1.097;-1.08	benign supporting
-0.310	[-0.319;-0.30	indeterminate
-1.113	[-1.115;-1.11	benign supporting
-0.558	[-0.611;-0.50	benign supporting
-0.970	[-0.983;-0.95	benign supporting
-0.437	[-0.511;-0.38	benign supporting
-1.103	[-1.109;-1.09	benign supporting
-1.034	[-1.040;-1.02	benign supporting
-1.115	[-1.119;-1.11	benign supporting
-1.027	[-1.032;-1.02	benign supporting
-0.173	[-0.402;0.45	indeterminate
-0.830	[-0.990;-0.64	benign supporting
1.276	[1.034;1.953	pathogenic strong
-0.045	[-0.288;0.58	indeterminate
-1.024	[-1.027;-1.02	benign supporting
-0.811	[-0.831;-0.78	benign supporting
1.151	[-0.198;5.03	pathogenic moderate
-0.422	[-0.487;-0.36	benign supporting
-1.105	[-1.112;-1.09	benign supporting
-0.353	[-0.374;-0.33	benign supporting

-1.011	[-1.047;-0.96	benign supporting
1.276	[1.208;1.365	pathogenic strong
-1.102	[-1.164;-0.95	benign supporting
0.047	[-0.002;0.14	indeterminate
0.972	[-0.205;1.45	pathogenic moderate
1.276	[1.235;1.322	pathogenic strong
1.276	[1.214;1.356	pathogenic strong
1.276	[1.259;1.294	pathogenic strong
-0.620	[-1.083;-0.25	benign supporting
-1.088	[-1.092;-1.08	benign supporting
0.998	[-1.101;5.05	pathogenic moderate
-0.012	[-0.265;0.63	indeterminate
-1.088	[-1.090;-1.08	benign supporting
1.276	[1.142;1.520	pathogenic strong
-1.166	[-3.481;1.22	benign supporting
1.276	[1.192;1.390	pathogenic strong
1.276	[1.208;1.364	pathogenic strong
-0.909	[-0.932;-0.87	benign supporting
1.276	[1.237;1.322	pathogenic strong
-0.334	[-0.382;-0.27	benign supporting
-0.163	[-0.716;1.15	indeterminate
-1.090	[-1.094;-1.08	benign supporting
-0.679	[-0.855;-0.57	benign supporting
-1.156	[-1.170;-1.13	benign supporting
1.276	[1.245;1.312	pathogenic strong
1.204	[1.130;1.288	pathogenic moderate
1.276	[1.240;1.317	pathogenic strong
0.856	[0.817;0.898	pathogenic moderate
-0.557	[-1.026;-0.17	benign supporting
0.920	[0.627;1.125	pathogenic moderate
-0.509	[-0.962;-0.13	benign supporting
1.276	[1.259;1.295	pathogenic strong
1.276	[1.260;1.294	pathogenic strong
-1.088	[-1.100;-1.08	benign supporting
-1.099	[-1.114;-1.08	benign supporting
-1.073	[-1.114;-0.77	benign supporting
-0.849	[-1.016;-0.64	benign supporting
-0.456	[-0.545;-0.38	benign supporting
-0.525	[-0.546;-0.50	benign supporting
1.276	[1.178;1.418	pathogenic strong
1.181	[1.154;1.210	pathogenic moderate
1.119	[0.946;1.197	pathogenic moderate
-0.919	[-3.551;1.18	benign supporting

1.260	[0.380;5.091]	pathogenic moderate
-1.093	[-1.146;-0.74	benign supporting
0.781	[0.729;0.835]	pathogenic moderate
-1.067	[-1.072;-1.05	benign supporting
-1.164	[-3.476;0.012	benign supporting
1.276	[1.250;1.305]	pathogenic strong
-0.589	[-1.099;0.078	benign supporting
1.130	[0.952;1.213]	pathogenic moderate
-0.109	[-0.341;0.476	indeterminate
1.276	[1.209;1.364]	pathogenic strong
1.111	[0.967;1.171]	pathogenic moderate
-1.090	[-1.091;-1.08	benign supporting
-0.478	[-0.624;-0.35	benign supporting
-0.522	[-0.586;-0.45	benign supporting
-1.130	[-1.147;-1.11	benign supporting
-0.852	[-0.908;-0.78	benign supporting
-0.737	[-0.876;-0.63	benign supporting
-0.277	[-0.302;-0.25	indeterminate
-1.095	[-1.107;-1.08	benign supporting
-0.593	[-0.674;-0.50	benign supporting
-0.475	[-0.491;-0.46	benign supporting
-0.800	[-0.833;-0.76	benign supporting
-1.092	[-1.095;-1.09	benign supporting
-1.101	[-1.115;-0.96	benign supporting
1.276	[1.128;1.570]	pathogenic strong
-0.628	[-0.659;-0.60	benign supporting
-0.284	[-0.358;-0.17	indeterminate
-0.829	[-0.892;-0.74	benign supporting
-1.095	[-1.097;-1.09	benign supporting
-1.095	[-1.168;-0.96	benign supporting
-1.130	[-1.153;-1.11	benign supporting
-0.435	[-0.571;-0.31	benign supporting
-1.043	[-1.113;-0.70	benign supporting
-0.742	[-0.909;-0.61	benign supporting
-0.828	[-0.882;-0.75	benign supporting
-0.916	[-0.923;-0.90	benign supporting
-1.096	[-1.164;-1.04	benign supporting
-0.760	[-0.808;-0.71	benign supporting
-1.073	[-1.083;-1.06	benign supporting
1.276	[-0.539;5.099	pathogenic strong
-0.566	[-0.707;-0.43	benign supporting
-0.527	[-0.606;-0.44	benign supporting
-1.096	[-1.106;-1.08	benign supporting

-1.064	[-1.073;-1.03	benign supporting
-0.668	[-0.809;-0.58	benign supporting
-1.030	[-1.110;-0.83	benign supporting
-0.949	[-1.068;-0.78	benign supporting
1.276	[1.212;1.356	pathogenic strong
-1.113	[-3.182;-0.22	benign supporting
-1.159	[-1.169;-1.09	benign supporting
1.276	[0.625;5.093	pathogenic strong
-1.085	[-1.097;-1.08	benign supporting
-1.031	[-1.056;-1.00	benign supporting
1.276	[-0.186;5.10	pathogenic strong
1.276	[0.347;5.104	pathogenic strong
0.141	[-1.092;4.92	indeterminate
-0.365	[-0.406;-0.31	benign supporting
1.224	[1.131;1.341	pathogenic moderate
1.276	[1.224;1.339	pathogenic strong
-0.388	[-0.423;-0.34	benign supporting
1.260	[1.078;1.612	pathogenic moderate
-1.088	[-1.091;-1.08	benign supporting
-1.037	[-1.112;-0.74	benign supporting
1.276	[0.540;5.094	pathogenic strong
1.116	[0.351;1.420	pathogenic moderate
0.396	[0.136;0.592	pathogenic supporting
-0.608	[-0.622;-0.58	benign supporting
1.276	[1.171;1.434	pathogenic strong
-1.096	[-1.109;-1.08	benign supporting
1.276	[0.765;5.092	pathogenic strong
1.276	[1.138;1.523	pathogenic strong
-1.084	[-1.086;-1.08	benign supporting
-1.073	[-1.082;-1.06	benign supporting
1.175	[1.150;1.203	pathogenic moderate
1.153	[1.113;1.198	pathogenic moderate
1.276	[1.212;1.358	pathogenic strong
-0.621	[-0.652;-0.59	benign supporting
1.080	[0.873;1.167	pathogenic moderate
1.017	[0.980;1.053	pathogenic moderate
0.421	[0.009;0.705	pathogenic supporting
1.276	[1.092;1.717	pathogenic strong
-1.045	[-1.097;-0.93	benign supporting
-0.786	[-0.904;-0.66	benign supporting
0.672	[0.526;0.789	pathogenic moderate
-1.100	[-1.104;-1.09	benign supporting
-0.322	[-0.344;-0.29	benign supporting

1.260	[1.233;1.289]	pathogenic moderate
-0.310	[-0.365;-0.24	indeterminate
1.276	[1.236;1.322]	pathogenic strong
-0.119	[-0.235;0.007	indeterminate
0.566	[0.423;0.687]	pathogenic supporting
1.276	[1.156;1.476]	pathogenic strong
-0.995	[-1.089;-0.82	benign supporting
-1.065	[-1.088;-1.00	benign supporting
-1.166	[-1.169;-1.10	benign supporting
-1.114	[-1.115;-1.06	benign supporting
-0.910	[-0.932;-0.88	benign supporting
-1.146	[-1.170;-1.11	benign supporting
-0.980	[-1.036;-0.91	benign supporting
-0.879	[-3.811;0.000	benign supporting
-0.391	[-0.559;-0.20	benign supporting
-1.098	[-1.114;-1.08	benign supporting
-1.087	[-1.093;-1.08	benign supporting
-0.450	[-0.898;-0.04	benign supporting
-0.354	[-0.657;0.338	benign supporting
-0.656	[-1.106;-0.16	benign supporting
-0.580	[-0.624;-0.53	benign supporting
-1.089	[-1.108;-1.07	benign supporting
0.870	[0.026;1.210]	pathogenic moderate
1.240	[0.793;2.206]	pathogenic moderate
-0.659	[-0.719;-0.61	benign supporting
-0.295	[-0.744;0.852	indeterminate
-1.110	[-1.166;-1.08	benign supporting
-1.166	[-1.169;-1.13	benign supporting
-1.092	[-1.099;-1.08	benign supporting
-1.105	[-1.108;-1.10	benign supporting
-1.031	[-1.048;-1.01	benign supporting
-0.620	[-0.634;-0.60	benign supporting
-1.102	[-1.105;-1.09	benign supporting
-0.241	[-0.260;-0.21	indeterminate
-1.040	[-1.067;-1.01	benign supporting
-0.791	[-0.808;-0.77	benign supporting
-0.884	[-0.902;-0.86	benign supporting
-0.920	[-0.931;-0.90	benign supporting
-0.928	[-0.942;-0.91	benign supporting
-0.918	[-0.930;-0.90	benign supporting
-0.461	[-0.476;-0.44	benign supporting
-0.289	[-0.310;-0.26	indeterminate
-0.892	[-0.920;-0.86	benign supporting

1.276	[1.231;1.329	pathogenic strong
-0.941	[-0.958;-0.92	benign supporting
-0.734	[-0.755;-0.71	benign supporting
-0.947	[-0.959;-0.93	benign supporting
-1.084	[-1.086;-1.08	benign supporting
-1.084	[-1.087;-1.08	benign supporting
1.276	[1.262;1.291	pathogenic strong
1.253	[1.228;1.281	pathogenic moderate
1.115	[1.045;1.150	pathogenic moderate
-1.165	[-1.166;-1.16	benign supporting
1.078	[1.004;1.122	pathogenic moderate
0.869	[0.806;0.937	pathogenic moderate
1.070	[1.041;1.100	pathogenic moderate
1.218	[1.195;1.242	pathogenic moderate
-1.103	[-1.107;-1.09	benign supporting
-0.983	[-0.990;-0.97	benign supporting
1.099	[1.047;1.119	pathogenic moderate
-0.835	[-0.851;-0.81	benign supporting
1.199	[1.184;1.214	pathogenic moderate
1.257	[1.240;1.276	pathogenic moderate
1.276	[1.260;1.293	pathogenic strong
-0.067	[-0.089;-0.04	indeterminate
1.267	[1.252;1.282	pathogenic moderate
-0.216	[-0.227;-0.20	indeterminate
-1.097	[-1.115;-1.07	benign supporting
-0.855	[-1.054;-0.62	benign supporting
-0.666	[-1.054;-0.40	benign supporting
1.276	[1.219;1.348	pathogenic strong
-0.766	[-1.077;-0.46	benign supporting
-1.098	[-1.102;-1.09	benign supporting
-0.201	[-0.271;-0.11	indeterminate
-0.950	[-0.962;-0.93	benign supporting
-0.804	[-0.813;-0.79	benign supporting
1.276	[1.215;1.354	pathogenic strong
-1.064	[-1.085;-1.00	benign supporting
-0.405	[-0.466;-0.34	benign supporting
-1.110	[-1.115;-1.10	benign supporting
1.276	[1.108;1.684	pathogenic strong
-0.952	[-0.954;-0.94	benign supporting
-0.275	[-0.985;1.18(	indeterminate
1.276	[1.143;1.507	pathogenic strong
-1.164	[-1.169;-0.97	benign supporting
0.541	[0.430;0.633	pathogenic supporting

-0.531	[-0.568;-0.49	benign supporting
-1.066	[-1.069;-1.06	benign supporting
-0.726	[-0.789;-0.67	benign supporting
-1.073	[-1.083;-1.06	benign supporting
0.512	[0.496;0.528	pathogenic supporting
-1.090	[-1.093;-1.08	benign supporting
-0.298	[-0.304;-0.29	indeterminate
-0.451	[-0.453;-0.44	benign supporting
-0.914	[-0.920;-0.90	benign supporting
-0.513	[-0.557;-0.46	benign supporting
-1.169	[-1.170;-1.16	benign supporting
-1.066	[-1.068;-1.06	benign supporting
-0.734	[-0.741;-0.72	benign supporting
-0.947	[-0.952;-0.94	benign supporting
-1.051	[-1.054;-1.04	benign supporting
1.276	[1.259;1.294	pathogenic strong
-1.106	[-1.108;-1.10	benign supporting
-0.558	[-0.560;-0.55	benign supporting
-1.068	[-1.085;-1.02	benign supporting
-0.685	[-1.100;-0.29	benign supporting
0.926	[0.481;1.166	pathogenic moderate
1.276	[1.232;1.327	pathogenic strong
1.276	[1.256;1.298	pathogenic strong
-0.973	[-0.977;-0.97	benign supporting
-0.548	[-1.168;4.758	benign supporting
-1.168	[-1.169;-1.16	benign supporting
1.042	[0.847;1.142	pathogenic moderate
-0.350	[-0.649;0.389	benign supporting
1.276	[1.178;1.408	pathogenic strong
-0.844	[-0.948;-0.70	benign supporting
0.670	[-0.425;1.391	pathogenic moderate
-0.366	[-0.380;-0.35	benign supporting
-1.110	[-1.168;-0.61	benign supporting
1.276	[1.106;1.694	pathogenic strong
1.276	[0.685;5.091	pathogenic strong
-1.104	[-1.107;-1.10	benign supporting
-0.940	[-1.036;-0.82	benign supporting
1.276	[1.243;1.313	pathogenic strong
1.276	[1.184;1.403	pathogenic strong
1.276	[1.143;1.509	pathogenic strong
-0.829	[-0.838;-0.81	benign supporting
1.276	[1.253;1.300	pathogenic strong
0.003	[-1.090;4.819	indeterminate

1.276	[1.266;1.287	pathogenic strong
1.276	[1.066;1.774	pathogenic strong
-1.002	[-1.005;-0.99	benign supporting
-1.164	[-1.169;-1.16	benign supporting
1.276	[1.258;1.296	pathogenic strong
-0.564	[-0.583;-0.54	benign supporting
1.276	[1.247;1.308	pathogenic strong
1.276	[1.180;1.407	pathogenic strong
1.276	[1.247;1.309	pathogenic strong
1.276	[1.044;1.936	pathogenic strong
1.276	[1.141;1.511	pathogenic strong
1.276	[1.233;1.325	pathogenic strong
1.276	[1.258;1.296	pathogenic strong
-1.113	[-1.167;-0.32	benign supporting
-0.463	[-1.262;4.894	benign supporting
1.212	[0.829;1.585	pathogenic moderate
1.276	[1.112;1.631	pathogenic strong
-1.095	[-1.099;-1.09	benign supporting
0.435	[0.236;0.601	pathogenic supporting
0.389	[0.017;0.650	pathogenic supporting
1.276	[0.647;5.091	pathogenic strong
-0.947	[-0.956;-0.93	benign supporting
-1.039	[-1.049;-1.02	benign supporting
-1.093	[-1.168;-1.07	benign supporting
0.674	[-0.262;1.217	pathogenic moderate
-0.894	[-0.899;-0.88	benign supporting
1.276	[1.239;1.319	pathogenic strong
-0.942	[-0.945;-0.93	benign supporting
1.193	[1.166;1.222	pathogenic moderate
1.116	[1.016;1.164	pathogenic moderate
-1.088	[-1.114;-1.05	benign supporting
1.183	[0.718;1.532	pathogenic moderate
1.276	[1.105;1.666	pathogenic strong
1.276	[1.238;1.321	pathogenic strong
1.276	[1.263;1.289	pathogenic strong
-0.090	[-0.155;-0.01	indeterminate
1.019	[0.758;1.153	pathogenic moderate
1.276	[0.996;2.188	pathogenic strong
1.257	[1.192;1.337	pathogenic moderate
-0.840	[-0.916;-0.74	benign supporting
1.276	[1.200;1.376	pathogenic strong
-1.067	[-1.069;-1.06	benign supporting
1.276	[0.898;5.004	pathogenic strong

1.276	[1.182;1.411	pathogenic strong
1.276	[1.171;1.436	pathogenic strong
1.276	[1.088;1.728	pathogenic strong
1.276	[1.200;1.376	pathogenic strong
1.276	[1.256;1.297	pathogenic strong
0.833	[0.763;0.907	pathogenic moderate
-1.105	[-1.115;-1.08	benign supporting
1.276	[0.000;5.103	pathogenic strong
-0.898	[-0.956;-0.83	benign supporting
1.276	[1.149;1.488	pathogenic strong
1.111	[0.557;1.310	pathogenic moderate
1.108	[0.880;1.193	pathogenic moderate
1.039	[0.819;1.149	pathogenic moderate
0.659	[0.338;0.881	pathogenic moderate
1.276	[1.135;1.527	pathogenic strong
-1.084	[-1.091;-1.08	benign supporting
-0.561	[-0.678;-0.43	benign supporting
-0.402	[-0.641;-0.09	benign supporting
-1.126	[-1.132;-1.12	benign supporting
-1.057	[-1.113;-0.79	benign supporting
0.945	[0.768;1.103	pathogenic moderate
1.276	[1.251;1.305	pathogenic strong
0.831	[0.652;1.017	pathogenic moderate
-1.106	[-1.107;-1.10	benign supporting
-1.040	[-1.146;-0.97	benign supporting
1.276	[1.103;1.764	pathogenic strong
1.276	[1.262;1.292	pathogenic strong
1.276	[1.252;1.303	pathogenic strong
-0.034	[-0.040;-0.02	indeterminate
1.276	[1.203;1.369	pathogenic strong
1.276	[1.240;1.316	pathogenic strong
0.890	[0.810;0.978	pathogenic moderate
1.115	[1.108;1.123	pathogenic moderate
-0.905	[-0.911;-0.89	benign supporting
1.276	[1.261;1.293	pathogenic strong
-1.093	[-1.094;-1.09	benign supporting
-1.089	[-1.090;-1.08	benign supporting
1.276	[1.144;1.495	pathogenic strong
1.276	[1.202;1.377	pathogenic strong
1.276	[0.929;2.920	pathogenic strong
1.199	[1.043;1.355	pathogenic moderate
1.276	[0.117;5.098	pathogenic strong
0.728	[0.681;0.771	pathogenic moderate

-0.896	[-0.940;-0.84	benign supporting
-0.956	[-0.962;-0.95	benign supporting
0.286	[-0.481;1.232	indeterminate
1.167	[0.458;1.724	pathogenic moderate
-0.877	[-0.886;-0.86	benign supporting
-1.084	[-1.092;-1.08	benign supporting
1.276	[-0.092;5.105	pathogenic strong
1.175	[1.127;1.230	pathogenic moderate
-1.092	[-1.115;-0.89	benign supporting
-0.923	[-0.929;-0.91	benign supporting
-0.268	[-0.409;-0.03	indeterminate
-0.467	[-0.635;-0.32	benign supporting
-0.618	[-0.640;-0.59	benign supporting
1.261	[1.208;1.322	pathogenic moderate
1.276	[1.217;1.347	pathogenic strong
-1.087	[-1.168;-0.68	benign supporting
-1.136	[-1.168;-0.86	benign supporting
1.276	[1.180;1.412	pathogenic strong
1.276	[1.242;1.314	pathogenic strong
1.276	[1.202;1.373	pathogenic strong
1.276	[1.172;1.435	pathogenic strong
0.814	[0.728;0.907	pathogenic moderate
1.276	[1.022;2.087	pathogenic strong
-1.011	[-1.164;-0.38	benign supporting
-0.021	[-0.506;1.127	indeterminate
1.138	[1.106;1.171	pathogenic moderate
1.276	[1.201;1.374	pathogenic strong
0.319	[0.118;0.474	pathogenic supporting
1.276	[1.127;1.566	pathogenic strong
1.276	[1.180;1.414	pathogenic strong
0.650	[0.352;0.864	pathogenic moderate
-0.844	[-1.085;-0.83	benign supporting
0.204	[-0.293;0.921	indeterminate
1.276	[1.103;1.687	pathogenic strong
-1.017	[-1.090;-0.96	benign supporting
1.276	[0.464;5.097	pathogenic strong
-1.166	[-1.170;-1.16	benign supporting
1.276	[0.931;4.749	pathogenic strong
0.615	[-1.085;5.014	pathogenic supporting
-0.072	[-0.136;0.011	indeterminate
0.756	[-0.069;1.165	pathogenic moderate
-0.965	[-1.039;-0.88	benign supporting
-0.468	[-0.562;-0.39	benign supporting

-0.976	[-1.108;-0.91	benign supporting
-1.108	[-1.167;-1.08	benign supporting
1.276	[1.176;1.432	pathogenic strong
0.544	[-0.051;0.93	pathogenic supporting
1.276	[1.255;1.299	pathogenic strong
-0.880	[-0.888;-0.87	benign supporting
1.276	[1.240;1.317	pathogenic strong
1.276	[1.199;1.382	pathogenic strong
-1.075	[-1.081;-1.07	benign supporting
-0.744	[-1.075;-0.44	benign supporting
1.276	[1.227;1.334	pathogenic strong
1.110	[1.018;1.152	pathogenic moderate
1.125	[0.428;1.425	pathogenic moderate
-0.506	[-0.573;-0.43	benign supporting
0.727	[0.645;0.796	pathogenic moderate
1.276	[1.246;1.310	pathogenic strong
-0.395	[-0.514;-0.27	benign supporting
0.677	[0.536;0.791	pathogenic moderate
-0.118	[-0.192;-0.04	indeterminate
-0.268	[-0.417;-0.00	indeterminate
0.959	[0.889;1.020	pathogenic moderate
-0.553	[-0.564;-0.53	benign supporting
-0.703	[-0.796;-0.64	benign supporting
1.276	[1.222;1.342	pathogenic strong
0.388	[-0.020;0.70	pathogenic supporting
1.276	[1.131;1.546	pathogenic strong
-0.456	[-0.940;0.07	benign supporting
1.218	[1.149;1.291	pathogenic moderate
-1.090	[-1.114;-0.98	benign supporting
-1.095	[-1.115;-1.00	benign supporting
-0.776	[-1.090;-0.44	benign supporting
1.276	[1.212;1.354	pathogenic strong
-1.087	[-1.103;-1.07	benign supporting
-0.488	[-0.520;-0.45	benign supporting
-1.115	[-1.134;-0.92	benign supporting
1.231	[-0.383;5.09	pathogenic moderate
-1.104	[-1.168;0.16	benign supporting
1.276	[-0.563;5.09	pathogenic strong
1.276	[1.024;2.043	pathogenic strong
0.331	[-0.218;0.891	pathogenic supporting
-1.100	[-1.111;-1.09	benign supporting
1.276	[1.205;1.375	pathogenic strong
-0.302	[-0.685;0.76	indeterminate

1.276	[1.184;1.401	pathogenic strong
-0.644	[-0.682;-0.61	benign supporting
-1.058	[-1.100;-0.95	benign supporting
-0.983	[-1.019;-0.94	benign supporting
-1.110	[-1.167;-0.51	benign supporting
1.276	[1.197;1.380	pathogenic strong
-0.920	[-0.940;-0.89	benign supporting
1.276	[1.103;1.709	pathogenic strong
0.470	[-0.602;1.507	pathogenic supporting
-0.594	[-0.705;-0.48	benign supporting
1.136	[1.000;1.215	pathogenic moderate
-0.851	[-1.164;0.680	benign supporting
-1.106	[-1.115;-1.08	benign supporting
1.276	[-0.231;5.099	pathogenic strong
1.276	[0.626;5.080	pathogenic strong
-1.102	[-1.109;-1.09	benign supporting
-0.086	[-0.208;0.162	indeterminate
-0.634	[-1.108;0.024	benign supporting
1.233	[0.727;2.624	pathogenic moderate
1.187	[1.090;1.294	pathogenic moderate
1.276	[1.256;1.299	pathogenic strong
-1.154	[-1.169;-1.11	benign supporting
-0.645	[-0.889;-0.49	benign supporting
-0.294	[-0.348;-0.22	indeterminate
-1.063	[-1.068;-1.05	benign supporting
-0.937	[-3.467;-0.83	benign supporting
1.276	[1.087;1.748	pathogenic strong
-0.940	[-0.945;-0.93	benign supporting
-0.405	[-0.477;-0.33	benign supporting
-0.935	[-0.943;-0.92	benign supporting
-0.199	[-0.273;-0.11	indeterminate
-0.268	[-0.389;-0.08	indeterminate
1.276	[0.799;5.066	pathogenic strong
-1.169	[-1.170;-1.15	benign supporting
1.276	[-0.055;5.108	pathogenic strong
-0.491	[-1.107;1.005	benign supporting
-0.825	[-1.094;-0.48	benign supporting
1.276	[0.673;5.078	pathogenic strong
-0.943	[-0.946;-0.94	benign supporting
-1.113	[-1.115;-1.07	benign supporting
-0.616	[-0.748;-0.50	benign supporting
1.162	[1.025;1.266	pathogenic moderate
1.079	[0.852;1.168	pathogenic moderate

1.276	[1.225;1.336	pathogenic strong
-0.627	[-0.654;-0.60	benign supporting
1.276	[1.131;1.530	pathogenic strong
1.019	[-0.102;1.421	pathogenic moderate
1.276	[1.241;1.316	pathogenic strong
1.177	[0.525;1.734	pathogenic moderate
-1.167	[-1.169;-1.13	benign supporting
-0.537	[-0.561;-0.51	benign supporting
1.276	[1.207;1.368	pathogenic strong
-0.347	[-0.421;-0.24	benign supporting
0.174	[0.009;0.336	indeterminate
1.276	[0.902;4.958	pathogenic strong
-0.045	[-0.333;0.69	indeterminate
1.276	[1.242;1.316	pathogenic strong
-1.109	[-1.115;-1.10	benign supporting
1.276	[1.226;1.335	pathogenic strong
1.276	[1.038;1.925	pathogenic strong
-1.014	[-1.068;-0.94	benign supporting
-1.103	[-1.115;-1.08	benign supporting
-0.530	[-0.584;-0.47	benign supporting
-0.601	[-0.626;-0.57	benign supporting
1.276	[1.241;1.314	pathogenic strong
-0.902	[-0.915;-0.88	benign supporting
-0.471	[-0.493;-0.45	benign supporting
-0.423	[-0.440;-0.40	benign supporting
-0.384	[-0.403;-0.36	benign supporting
-0.167	[-0.201;-0.13	indeterminate
-0.161	[-0.229;-0.09	indeterminate
-0.669	[-0.679;-0.66	benign supporting
-0.441	[-0.461;-0.42	benign supporting
-0.429	[-0.453;-0.40	benign supporting
-0.356	[-0.383;-0.32	benign supporting
1.276	[1.238;1.320	pathogenic strong
-0.493	[-0.575;-0.41	benign supporting
1.276	[1.254;1.300	pathogenic strong
-0.522	[-0.573;-0.46	benign supporting
-0.232	[-0.252;-0.20	indeterminate
-0.867	[-0.889;-0.84	benign supporting
-1.092	[-1.094;-1.09	benign supporting
-0.347	[-0.387;-0.30	benign supporting
1.276	[1.262;1.291	pathogenic strong
1.115	[0.901;1.201	pathogenic moderate
1.276	[1.216;1.351	pathogenic strong

-0.996	[-1.007;-0.97	benign supporting
0.382	[0.309;0.451]	pathogenic supporting
-0.088	[-0.131;-0.03	indeterminate
1.231	[1.189;1.275]	pathogenic moderate
-0.058	[-0.147;0.115	indeterminate
-0.997	[-1.012;-0.97	benign supporting
-1.109	[-1.115;-1.09	benign supporting
1.276	[1.212;1.356]	pathogenic strong
-0.760	[-0.793;-0.73	benign supporting
0.493	[0.331;0.633]	pathogenic supporting
1.276	[1.192;1.393]	pathogenic strong
-0.340	[-0.356;-0.32	benign supporting
1.276	[1.241;1.317]	pathogenic strong
-0.398	[-0.409;-0.38	benign supporting
0.868	[0.822;0.916]	pathogenic moderate
0.068	[-0.077;0.361	indeterminate
-1.151	[-1.168;-1.13	benign supporting
-1.041	[-1.063;-1.02	benign supporting
-0.957	[-1.165;0.067	benign supporting
-0.563	[-0.601;-0.52	benign supporting
-0.342	[-0.563;-0.00	benign supporting
-0.592	[-0.619;-0.56	benign supporting
1.276	[1.123;1.568]	pathogenic strong
-0.472	[-0.509;-0.44	benign supporting
-1.094	[-1.100;-1.08	benign supporting
-1.167	[-1.170;-1.15	benign supporting
-0.631	[-0.773;-0.53	benign supporting
1.276	[-1.148;4.983	pathogenic strong
-0.672	[-0.915;-0.52	benign supporting
-0.142	[-0.195;-0.09	indeterminate
1.230	[0.870;1.766]	pathogenic moderate
0.981	[-0.353;2.541	pathogenic moderate
1.276	[1.196;1.382]	pathogenic strong
-0.135	[-0.243;-0.02	indeterminate
1.276	[1.170;1.437]	pathogenic strong
1.247	[1.200;1.298]	pathogenic moderate
-1.089	[-1.114;-1.05	benign supporting
1.276	[0.452;5.098]	pathogenic strong
1.276	[1.122;1.575]	pathogenic strong
1.115	[-0.171;2.963	pathogenic moderate
1.167	[-1.164;4.838	pathogenic moderate
-1.101	[-1.116;-1.09	benign supporting
0.068	[-0.178;0.587	indeterminate

1.156	[1.107;1.211	pathogenic moderate
0.972	[0.837;1.083	pathogenic moderate
-0.285	[-1.109;1.951	indeterminate
-0.391	[-0.592;-0.14	benign supporting
-0.478	[-0.671;-0.31	benign supporting
1.219	[0.555;4.963	pathogenic moderate
1.276	[-0.359;5.101	pathogenic strong
0.905	[0.765;1.040	pathogenic moderate
-0.019	[-0.488;1.12	indeterminate
1.276	[1.125;1.564	pathogenic strong
-0.340	[-0.483;-0.11	benign supporting
-0.278	[-0.527;0.41	indeterminate
1.276	[1.177;1.415	pathogenic strong
1.191	[0.583;1.898	pathogenic moderate
