# Supplementary material for: Systematically testing human HMBS missense variants to reveal mechanism and pathogenic variation
Source: bioRxiv. 2023 Feb 6:2023.02.06.527353. Preprint. [Version 1] doi: 10.1101/2023.02.06.527353 (PMC9934555; doi:10.1101/2023.02.06.527353)
Supplement: Supplement 5 [file NIHPP2023.02.06.527353v1-supplement-5.pdf]

bioRxiv preprint doi: https://doi.org/10.1101/2023.02.06.527353; this version posted February 6, 2023. The copyright holder for this preprint (which was not certified by peer review) is the author/funder, who has granted bioRxiv a license to display the preprint in perpetuity. It is made available under aCC-BY 4.0 International license.

Supplemental Figures

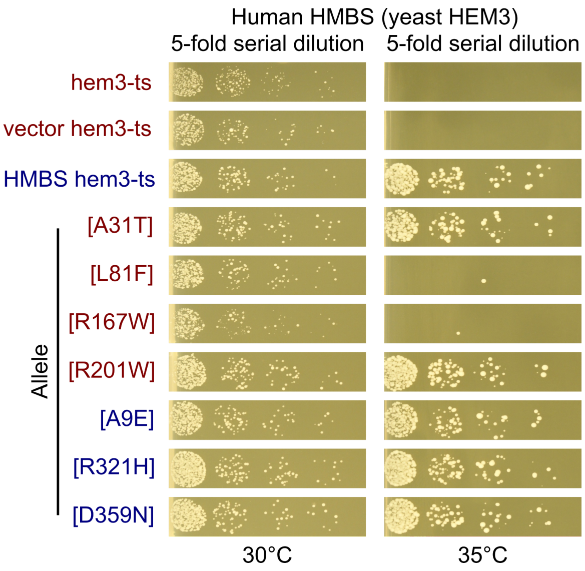

Supplemental Figure S1. Functional complementation assay results showing whether expression of human HMBS protein variants can rescue growth of a yeast strain bearing a temperature-sensitive mutation in the essential gene *HEM3*.

Pathogenic variants A31T, L81F, R167W and R201W and negative controls are indicated in red text, while benign variants A9E, R321H and D359N and a wild-type HMBS control are indicated in blue text. Fivefold serial dilutions of yeast cells were spotted onto plates, with growth evaluation after 48 hours of incubation at either permissive (30°C) or non-permissive (35°C) temperature.

bioRxiv preprint doi: https://doi.org/10.1101/2023.02.06.527353; this version posted February 6, 2023. The copyright holder for this preprint (which was not certified by peer review) is the author/funder, who has granted bioRxiv a license to display the preprint in perpetuity. It is made available under aCC-BY 4.0 International license.

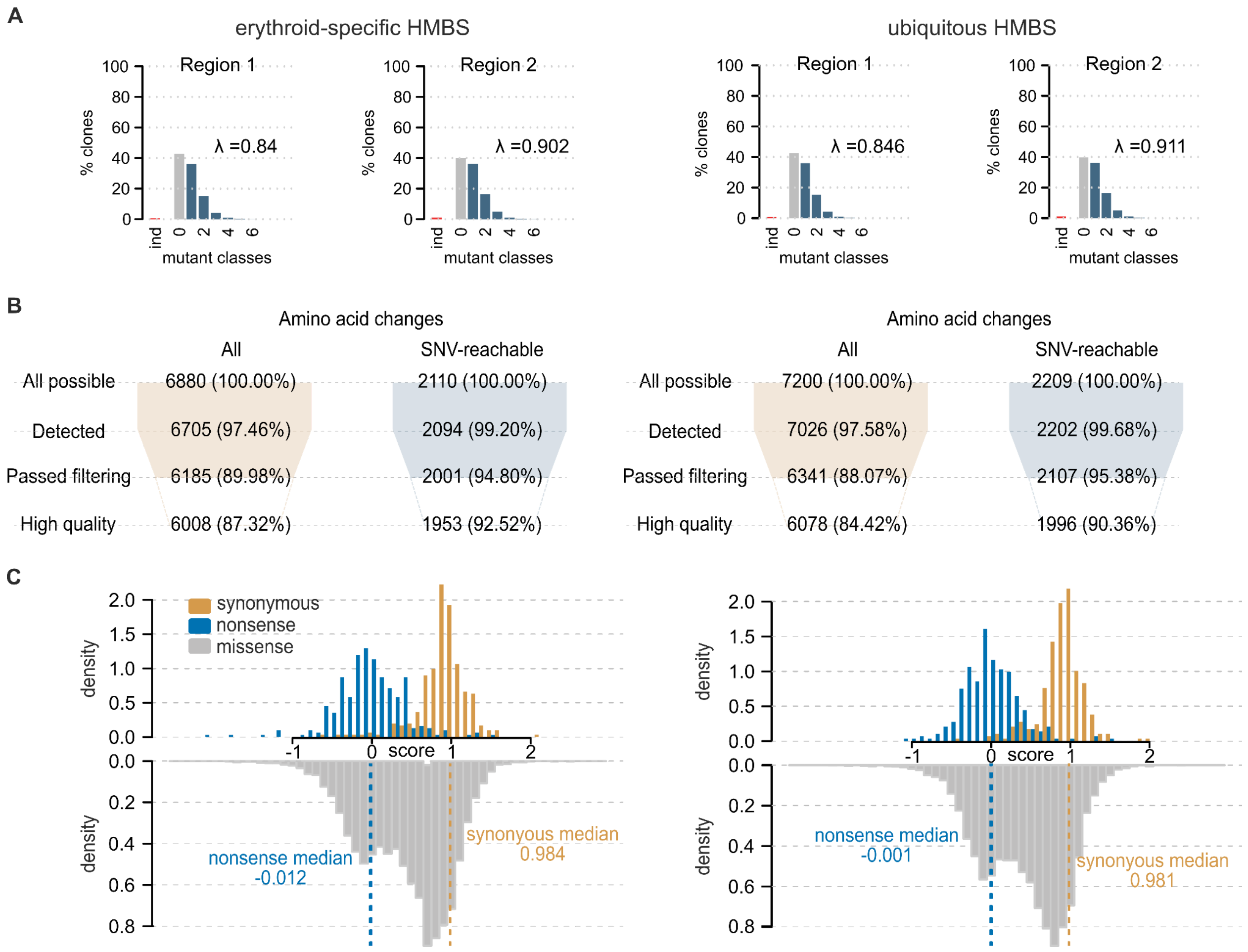

Supplemental Figure S2. HMBS variant libraries underlying the combined variant effect map

(A) Distribution of the number of missense variants in clones from the erythroid-specific (left) and ubiquitous (right) HMBS mutagenized libraries, and the fraction of clones carrying small indels ("ind"). The average number of amino acid changes per clone (λ) is also estimated (see Methods).

(B) Fractions of variants detected and passing after each of two levels of quality control are shown for each HMBS isoform. For each isoform, the number of synonymous, nonsense, and missense substitutions (up to 19 possible) are shown across all residue positions, both before (left) and after (right) restricting to substitutions that are possible given a single nucleotide change. The four rows correspond to: 1) theoretically possible substitutions; 2) substitutions detected in the non-selective condition; 3) substitutions above a threshold pre-selection frequency; and 4) substitutions with a sufficiently low regularized standard error.

(C) Distributions of measured functional impact scores for nonsense, synonymous, and missense variants for the erythroid-specific (left) and ubiquitous (right) HMBS isoforms.

bioRxiv preprint doi: https://doi.org/10.1101/2023.02.06.527353; this version posted February 6, 2023. The copyright holder for this preprint (which was not certified by peer review) is the author/funder, who has granted bioRxiv a license to display the preprint in perpetuity. It is made available under aCC-BY 4.0 International license.

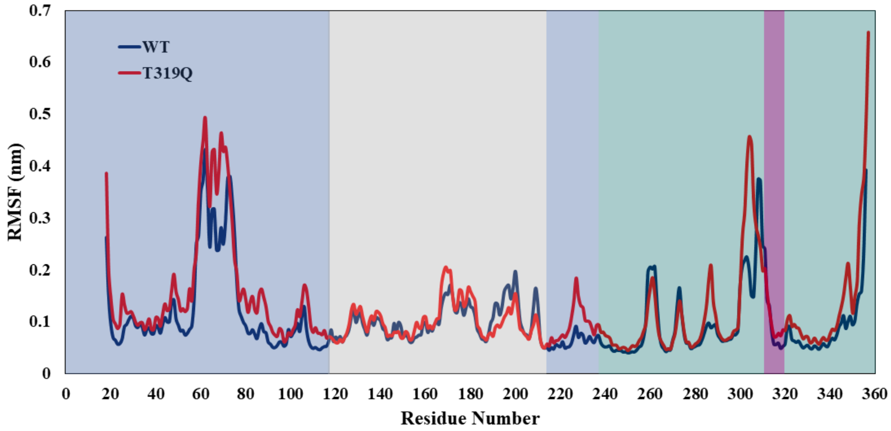

Supplemental Figure S3. Root-mean-square fluctuation (RMSF) of Cα atoms of WT HMBS and the T319Q variant.

RMSF reveals the average deviation of atoms throughout the simulation with respect to the initial structure. Domains 1, 2, and 3 are highlighted in light blue, gray, and green, respectively. The 316-319 positions at the interface of domains 1 and 3 are highlighted in purple.

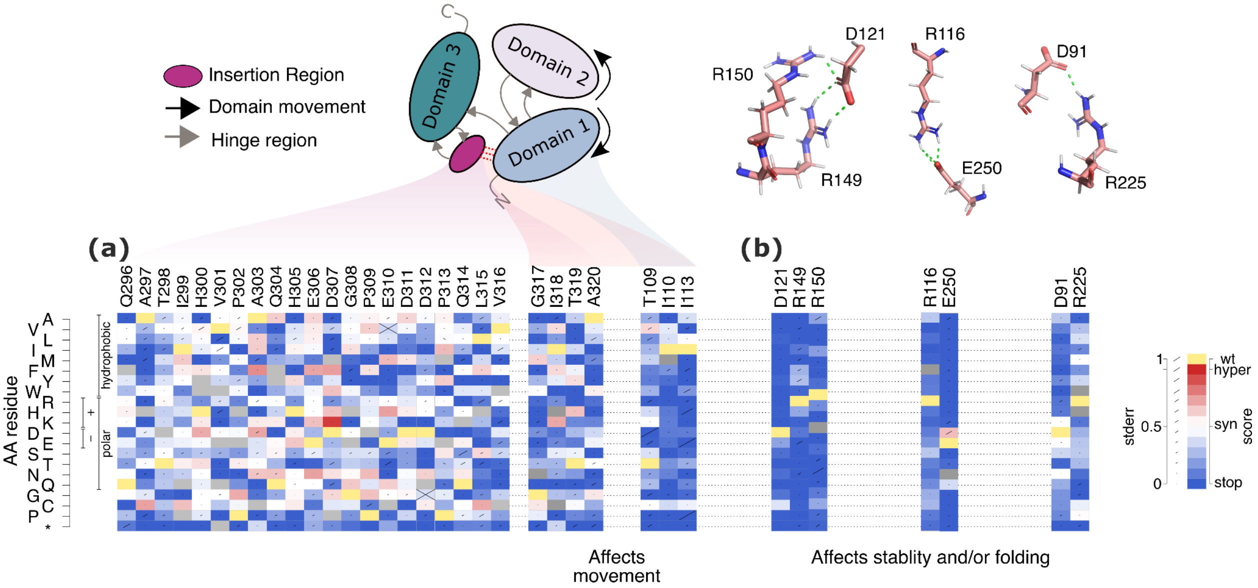

Supplemental Figure S4. Placing functional impact scores in the context of residue roles in moderating HMBS fluctuation, protein stability or folding.

Functional scores for each possible substituted amino acid (*y*-axis) at each residue position outside the active site (*x*-axis) participating in: (a) constraining HMBS domain movement, or (b) salt bridges. For each substitution, diagonal bar sizes convey estimated measurement error in the corresponding functional score. Box color either indicates the wild-type residue (yellow), or a substitution with damaging (blue), tolerated (white), or above-wildtype ('hyper-complementing', red) functional score, or missing data (gray).

bioRxiv preprint doi: https://doi.org/10.1101/2023.02.06.527353; this version posted February 6, 2023. The copyright holder for this preprint (which was not certified by peer review) is the author/funder, who has granted bioRxiv a license to display the preprint in perpetuity. It is made available under aCC-BY 4.0 International license.

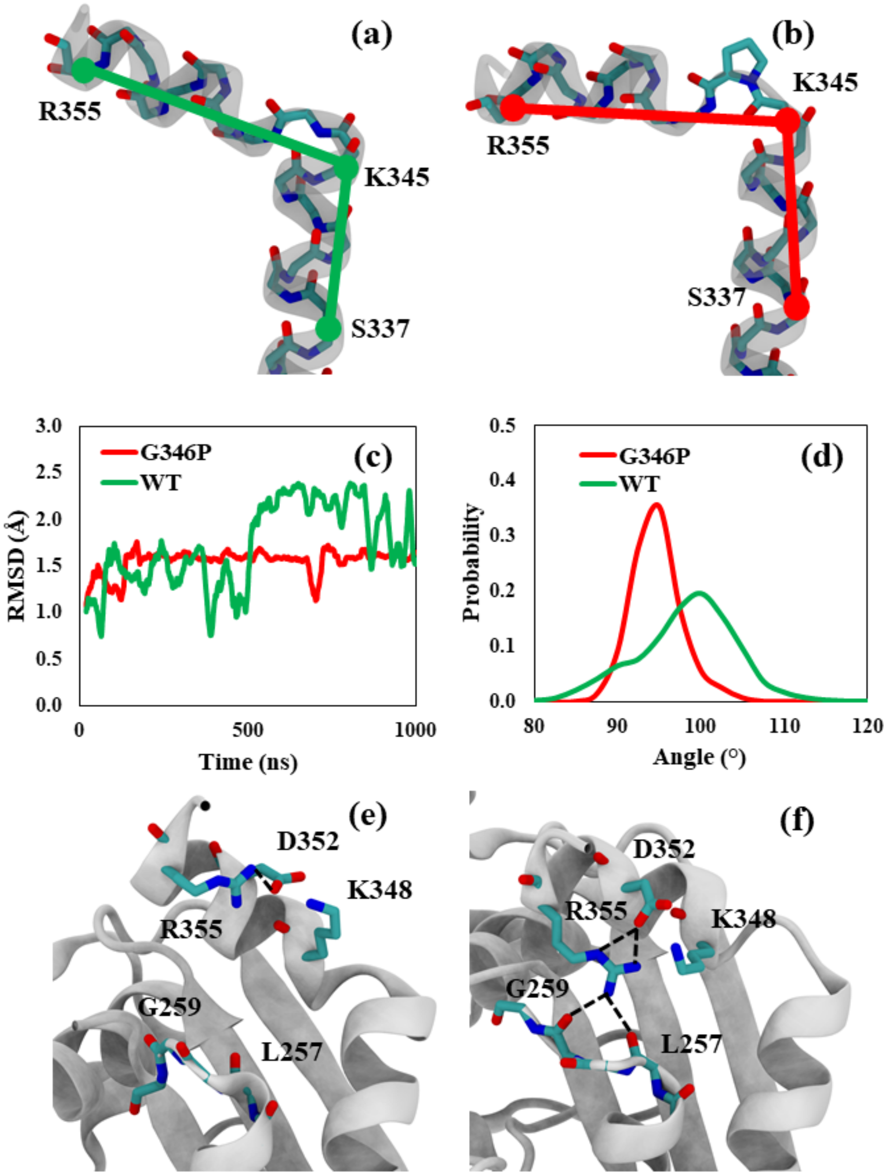

Supplemental Figure S5. Comparison between WT and the G346P clinical variant showing a widespread impact on protein flexibility.

Backbone atoms of S337, K345, and R355 were selected to evaluate the angle between the two helices in (a) WT and (b) G346P. For clarity only the C-terminal helices are shown. (c) The roost-mean-squared deviation (RMSD) of the backbone atoms in the C-terminal helix (K345-L357). (d) Angle distribution for WT (average: 100° ± 6) and G346 (average: 96° ± 3). Interactions of R355 with neighboring residues in (e) WT and (f) G346P

bioRxiv preprint doi: https://doi.org/10.1101/2023.02.06.527353; this version posted February 6, 2023. The copyright holder for this preprint (which was not certified by peer review) is the author/funder, who has granted bioRxiv a license to display the preprint in perpetuity. It is made available under aCC-BY 4.0 International license.

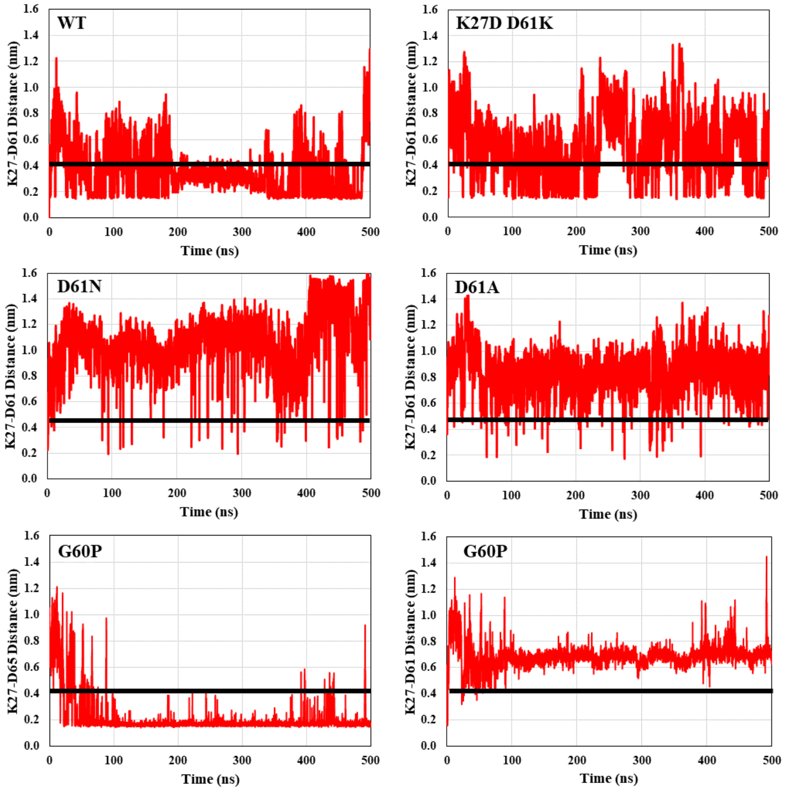

Supplemental Figure S6. Monitoring open vs. closed active site loop status based on average distance between salt bridge residues 27 and 61 over time: WT (top left), K27D D61K (top right), D61N (mid left), D61A (mid right), and G60P (bottom). Bottom right shows the average distance between an alternative salt bridge between residues 27 and 65 in the G60P mutant. The salt bridge formation threshold (4 Å) is shown by a black solid line.

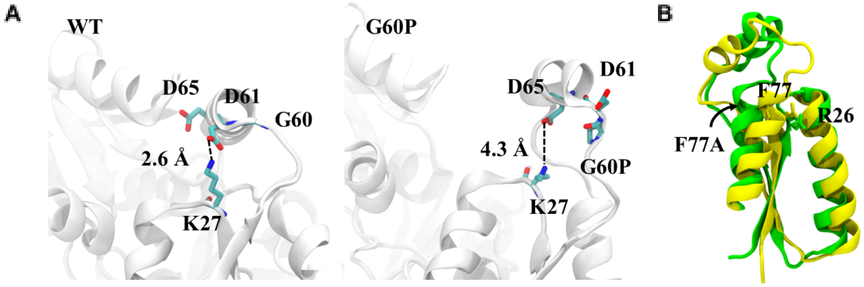

Supplemental Figure S7. Modeling the effect of G60P and F77A variants on active site loop conformation.

(A) Comparison of salt bridge distances (Å) in WT HMBS (left) and the G60P variant (right). The D61-K27 interaction is lost with the G60P variant, and instead replaced by a D65-K27 salt bridge that retains the active site loop in the "open" state.
(B) The F77A variant (green) results in a dislocation of the active-site loop compared to the WT (yellow), exposing the active site.

bioRxiv preprint doi: https://doi.org/10.1101/2023.02.06.527353; this version posted February 6, 2023. The copyright holder for this preprint (which was not certified by peer review) is the author/funder, who has granted bioRxiv a license to display the preprint in perpetuity. It is made available under aCC-BY 4.0 International license.

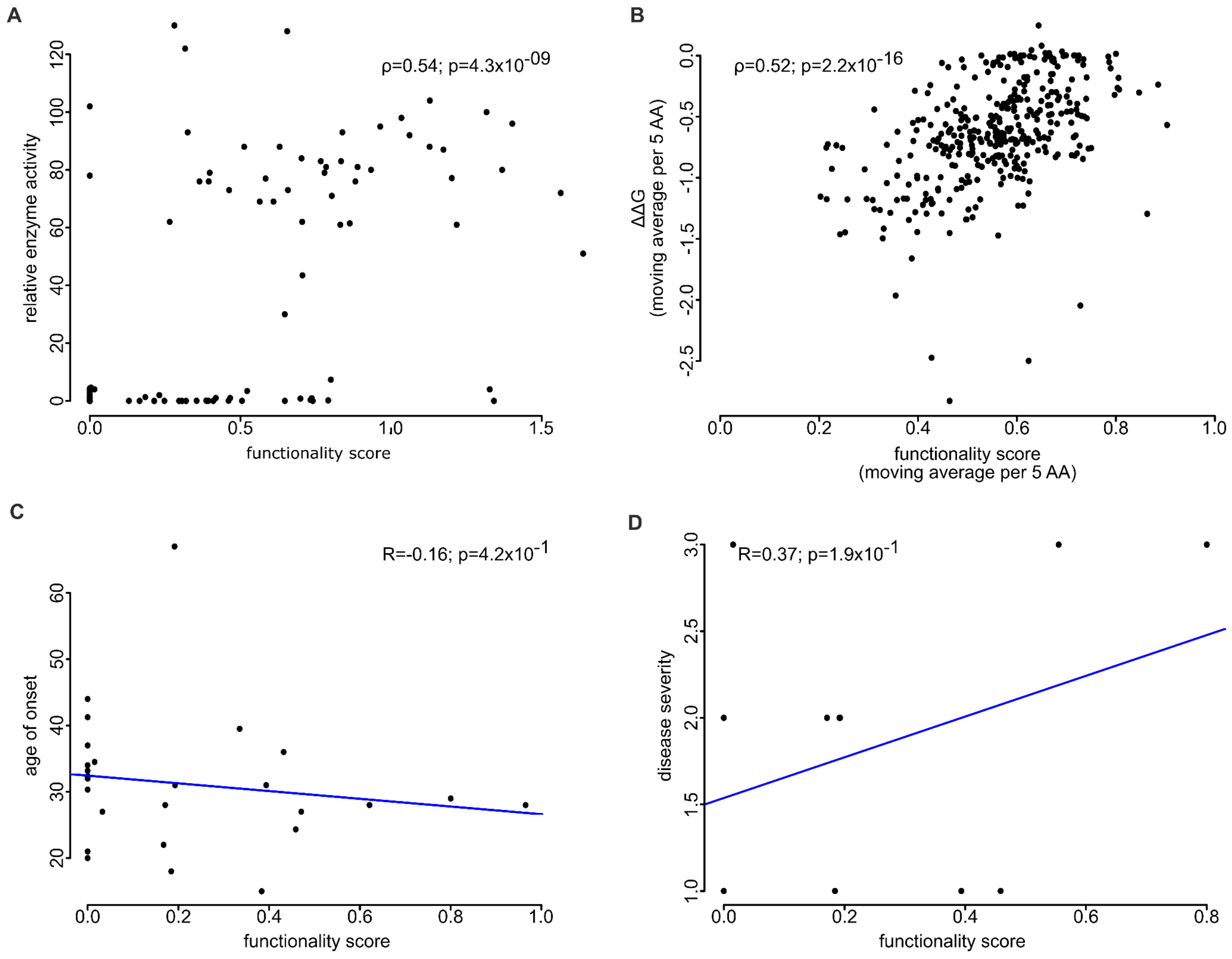

Supplemental Figure S8. Correlation of functional impact scores from the combined map with other variant properties. Either Spearman's rank correlation (ρ) with significance (p) of correlation, or Pearson correlation (R) with significance (p) are shown.

(A) Correlation (ρ = 0.54, p= 4 x10^{-9}) with HMBS relative enzyme activity (variant activity divided by wild type activity).

(B) Correlation (ρ= 0.52, p= 0.2) of running medians (interval size of 5 amino acids) of the combined map scores and predicted folding free energy change (ΔΔG) values.

(C) Correlation (R=-0.16; P = 0.4) with age of AIP onset.

(D) Correlation (R=0.37; P = 0.2) with AIP severity scores (3 = mild disease, 2 = moderate disease, 1 = severe disease).

bioRxiv preprint doi: https://doi.org/10.1101/2023.02.06.527353; this version posted February 6, 2023. The copyright holder for this preprint (which was not certified by peer review) is the author/funder, who has granted bioRxiv a license to display the preprint in perpetuity. It is made available under aCC-BY 4.0 International license.

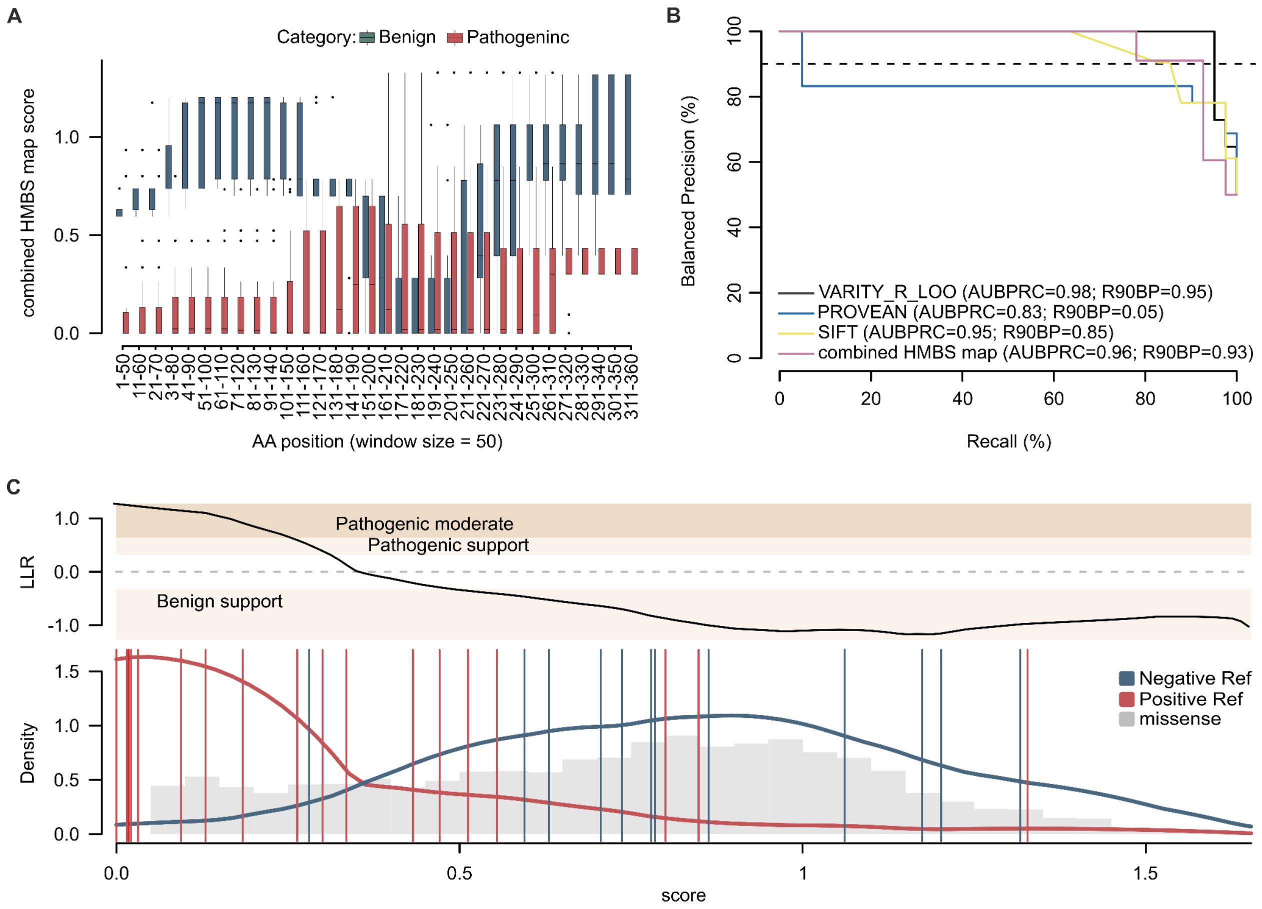

Supplemental Figure S9. Evaluating the ability of the combined HMBS map, as well as computational predictors, to distinguish positive from random reference variants and interpret clinically-relevant missense variants from the ClinVar database.

(A) Moving window analysis of scores from the combined map, as part of the positive (red) or negative (blue) reference variant sets. Plotted values are averages within windows of 50 amino acid (AA) positions.

(B) Evaluation of precision (fraction of variants scoring below each threshold functional impact score that are in the positive reference set containing pathogenic variants) vs recall (fraction of positive reference variants with functionality scores below threshold). Here, precision has been balanced to reflect performance in a balanced test setting where positive and negative sets contain the same number of variants, excluding all at residue positions 160-215. Balanced precision-recall curves are shown for the combined map (pink), computational predictors PROVEAN (green), SIFT (yellow), and VARITY (black). Performance is also described in terms of area under the balanced precision vs recall curve (AUBPRC) and recall at a balanced precision of 90% (R90BP).

(C) Transformation functions represent variant effects in terms of the strength of evidence for and against pathogenicity, that is, a log-likelihood ratio (LLR) of pathogenicity. The functions (top) express the log ratio between the likelihood of observing a given score in the score distribution of the positive reference (red) set as opposed to that of the negative reference set (blue). Gray histogram bars show the distribution of missense variants for comparison.

bioRxiv preprint doi: https://doi.org/10.1101/2023.02.06.527353; this version posted February 6, 2023. The copyright holder for this preprint (which was not certified by peer review) is the author/funder, who has granted bioRxiv a license to display the preprint in perpetuity. It is made available under aCC-BY 4.0 International license.

Supplemental Figure S10. Full-sized HMBS variant effect maps.

(A) Complete functional maps for erythroid-specific and ubiquitous HMBS isoforms, and the weighted average score values. Colors and labels are as in Figure 2.
(B) Delta map, measuring ubiquitous − erythroid-specific functional impact. Substitutions were coloured red if the score is positive and blue if negative.

Supplemental Tables

Table S1. Comparing different models for effects of activity enhancing mutations.

**Model**	**ΔAIC relative to best model**
Penalize enhancing mutations	0
Cap score of enhancing mutations at wild type level	24.1
Enhancing mutations are beneficial	38.0

Table S2. Average RMSD (standard deviation in parentheses) of backbone atoms for the wild-type (WT) or enzyme substrate complex 2 (ES2) structures.

**System**	**RMSD (nm)**
Apo-enzyme WT	0.21 (0.02)
Apo-enzyme G60P	0.33 (0.05)
Apo-enzyme D61N	0.19 (0.04)
Apo-enzyme D61A	0.19 (0.04)
Apo-enzyme K27D D61K	0.31 (0.03)
Apo-enzyme R26P	0.27 (0.04)
ES2 WT	0.23 (0.03)
ES2 E250R	0.22 (0.02)
ES2 G346P	0.23 (0.02)

bioRxiv preprint doi: https://doi.org/10.1101/2023.02.06.527353; this version posted February 6, 2023. The copyright holder for this preprint (which was not certified by peer review) is the author/funder, who has granted bioRxiv a license to display the preprint in perpetuity. It is made available under aCC-BY 4.0 International license.

Table S3. Hydrogen bond occupancy (%) between HMBS important residues and the substrate in different systems. For residues with more than one interaction with PBG, only the more persistent interaction is reported.

		DPM-PBG	
**Residue**	**WT**	**E250R**	**G346P**
R149	100	82	87
R150	100	85	100
G218	100	-	95
S147	99	57	65
S96	99	55	65
A189	98	56	73
R173	98	95	42
T102	100	58	70
L148	72	-	-
K98	100	100	100
S146	68	99	54
R195	57	76	50
R117	-	93	86
T145	68	85	38
D99*	94, 80	20, 4	7, 6

*Numbers correspond to hydrogen bonds with the 4th (second PBG) and 3rd (first PBG) pyrrole ring, respectively.

bioRxiv preprint doi: https://doi.org/10.1101/2023.02.06.527353; this version posted February 6, 2023. The copyright holder for this preprint (which was not certified by peer review) is the author/funder, who has granted bioRxiv a license to display the preprint in perpetuity. It is made available under aCC-BY 4.0 International license.

Table S4. Hydrogen bond occupancy (%) in the C-terminal helix region for WT and the G346P variant.

		Occupancy (%)	
**Backbone Hydrogen Bonds**		**G346P**	**WT**
L342 (O)	K345 (NH)	80	-
L342 (O)	A347 (NH)	-	87
L342 (O)	G346 (NH)	-	57
L342 (NH)	L338 (O)	99	100
L343(O)	G346 (O)	-	46
L343 (NH)	A339 (O)	92	97
S344 (NH)	L341 (O)	62	20
S344 (NH)	N340 (O)	-	78
K345 (O)	A347 (NH)	91	-
K345 (NH)	L342 (O)	80	-
K345 (NH)	L341 (O)	-	90
G346 (O)	N349 (NH)	-	55
G346 (NH)	L342 (O)	-	57
G346 (NH)	L343 (O)	-	46
P346 (O)	N349 (NH)	47	-
P346 (O)	I350 (NH)	32	-
A347 (O)	I350 (NH)	49	65
A347 (O)	L351 (NH)	92	87
A347 (NH)	K345 (O)	91	-
A347 (NH)	L342 (O)	-	87

bioRxiv preprint doi: https://doi.org/10.1101/2023.02.06.527353; this version posted February 6, 2023. The copyright holder for this preprint (which was not certified by peer review) is the author/funder, who has granted bioRxiv a license to display the preprint in perpetuity. It is made available under aCC-BY 4.0 International license.

Table S5. Hydrogen bond occupancy (%) for R355 in WT and the G346P variant.

G346P

**Hydrogen Bond**		**Occupancy (%)**
R355 (NH1)	G259 (O)	82
R355 (NH2)	L257 (O)	63
R355 (NH22)	D352 (Oδ2)	59
R355 (NH21)	D352 (Oδ1)	32

WT

**Hydrogen Bond**		**Occupancy (%)**
R355 (NH1)	D352 (Oδ1)	19
R355 (NH)	D352 (O)	16

Table S6. Average distance (Å) between selected residue pairs in WT and variants.

	WT	K27D D61K	D61N	D61A	G60P	R26P
R26-D61	7.6	8.6	12	11	14	13
K27-D61	3.5	5.4	11	8.5	6.9	10
G60-Q34	17	18	20	20	21	20
G60-R26	9.1	8.9	10	11	13	12

Table S7. Curated reference variant sets for validation
Table S8. HMBS missense variants and corresponding scores, log likelihood ratios of pathogenicity and calibrated evidence strength labels
Table S9. List of TileSeq primers and POPcode mutagenesis oligos
Table S10. Ensembl homologs having at least 85% sequence identity to the human HMBS protein
